# Leaf morphology, rather than plant water status, underlies genetic variation of rice leaf rolling under drought

**DOI:** 10.1111/pce.13514

**Published:** 2019-02-20

**Authors:** Andrew J. Cal, Millicent Sanciangco, Maria Camila Rebolledo, Delphine Luquet, Rolando O. Torres, Kenneth L. McNally, Amelia Henry

**Affiliations:** ^1^ Strategic Innovation Platform International Rice Research Institute Metro Manila 1301 Philippines; ^2^ AGAP, Univ Montpellier, CIRAD, INRA, Montpellier SupAgro Montpellier France; ^3^ Agrobiodiversity International Center for Tropical Agriculture Cali AA6713 Colombia

**Keywords:** aus, drought, leaf rolling, rice, tropical japonica

## Abstract

Soil drying causes leaf rolling in rice, but the relationship between leaf rolling and drought tolerance has historically confounded selection of drought‐tolerant genotypes. In this study on tropical japonica and aus diversity panels (170–220 genotypes), the degree of leaf rolling under drought was more affected by leaf morphology than by stomatal conductance, leaf water status, or maintenance of shoot biomass and grain yield. A range of canopy temperature and leaf rolling (measured as change in normalized difference vegetation index [ΔNDVI]) combinations were observed among aus genotypes, indicating that some genotypes continued transpiration while rolled. Association mapping indicated colocation of genomic regions for leaf rolling score and ΔNDVI under drought with previously reported leaf rolling genes and gene networks related to leaf anatomy. The relatively subtle variation across these large diversity panels may explain the lack of agreement of this study with earlier reports that used small numbers of genotypes that were highly divergent in hydraulic traits driving leaf rolling differences. This study highlights the large range of physiological responses to drought among rice genotypes and emphasizes that drought response processes should be understood in detail before incorporating them into a varietal selection programme.

## Abstract 2

Leaf rolling was characterized in two rice diversity panels (aus and tropical japonica) under drought stress, either by visual scoring or by change in NDVI over successive dates. The phenotypic variation and identified candidate genes/networks related to leaf rolling under drought in two rice (Oryza sativa L.) diversity panels (aus and tropical japonica) showed stronger relationships with leaf morphology than with plant growth or grain yield under drought. These results help explain why selection for leaf rolling has historically confounded selection of genotypes with higher yield under drought.

**Abbreviations**
CT: canopy temperature
NDVI: normalized difference vegetation index

## 1 INTRODUCTION

Rice leaves typically show increasing degrees of leaf rolling in response to increasing severity of drought stress (DS). Leaf rolling score is a visual evaluation considered to be a rapid and economical measurement that was historically recommended as a screening parameter for drought tolerance in rice (Loresto, Chang, & Tagumpay, 1976). However, leaf rolling score is no longer recommended as a key trait for drought screening because it is not well correlated with yield under drought (Lafitte, Blum, & Atlin, 2003) and was reported to show less sensitivity to drought than other physiological parameters (Turner, O'Toole, Cruz, Namuco, & Ahmad, 1986). A better understanding of the physiology and genetics behind leaf rolling under drought may help elucidate how this trait could be used most effectively to distinguish between drought resistant and susceptible rice genotypes.

A number of studies have characterized leaf rolling under drought in rice as related to other measures of leaf water status. O'Toole and Cruz (1979) reported that transpiration rate per unit leaf area decreased then increased when rice leaves were manually rolled and then unrolled, leading the authors to conclude that the boundary layer formed upon rolling had a stronger effect than stomatal closure on transpiration of rolled leaves. This “modified microclimate” created by the leaf upon rolling was reported to be linked to differential stomatal resistance between the abaxial and adaxial sides of the leaf (O'Toole & Cruz, 1980), although Henson (1982) reported that stomata closed before leaf rolling occurred. Leaf rolling improved water use efficiency by affecting transpiration more than CO_2_ assimilation (Dingkuhn, Cruz, O'Toole, & Dörffling, 1989). Leaf rolling was reported to be directly related to leaf water potential (LWP), but the LWP threshold differed among Oryza sativa and Oryza glaberrima genotypes (Dingkuhn et al., 1989; Dingkuhn, Audebert, Jones, Etienne, & Sow, 1999; Henson, 1982; Hsiao, O'Toole, Yambao, & Turner, 1984; and Turner et al., 1986). Excised leaves with higher osmotic potentials rolled at more negative LWPs (Hsiao et al., 1984). O'Toole and Cruz (1980) emphasized that because multiple parameters change together with increasing DS, it is difficult to disentangle the roles of these parameters in leaf rolling.

In addition to affecting leaf water status under drought, leaf morphological parameters have been reported as criteria affecting the susceptibility of a rice genotype to exhibit leaf rolling under drought. Turner et al. (1986) observed that dryland cultivars (which tend to be tall) rolled earlier in the DS treatment than wetland cultivars. Tall genotypes have typically shown higher leaf rolling scores than dwarf genotypes regardless of their drought susceptibility (Chang & Loresto, 1986; Dingkuhn et al., 1989, 1999). In 39 diverse grass species (not including rice), small leaf width was correlated with high drought resistance index (Redmann, 1985). Biomechanics have also been hypothesized to play an important role in rice leaf rolling under drought (Price, Young, & Tomos, 1997), particularly the turgor of bulliform cells (Hsiao et al., 1984).

Given the complex interactions among leaf rolling under drought, LWP, stomatal conductance, and leaf morphology, many questions remain about the role leaf rolling plays in drought resistance: Is leaf rolling in rice a symptom of drought susceptibility or a mode of conserving water? Are stomata closed when rice leaves are rolled? Do genetic differences in leaf anatomy affect leaf rolling under drought? Furthermore, despite the strong genotypic effect reported on rice leaf rolling in response to drought, little is known about the genetics related to this trait, and previous physiology studies have included only 1–7 genotypes. However, Singh and Mackill (1991) observed transgressive segregation for diurnal changes in leaf rolling, suggesting that major genes—likely at multiple loci—might be detected for susceptibility to leaf rolling. A better understanding of the genetics behind rice leaf rolling under drought may help define the physiology of this trait, so that its potential value in plant breeding can be determined. Studying large numbers of diverse genotypes may facilitate both the physiological and genetic characterization of rice leaf rolling under drought. In this study, we characterized leaf rolling under drought in three field experiments and one greenhouse lysimeter experiment using 220 rice genotypes from the aus subgroup. Results from a separate experiment on 172 japonica genotypes under vegetative‐stage dry‐down in the greenhouse (Rebolledo et al., 2013) were also evaluated. This study allowed the comparison of multiple physiological drought response parameters, and because the aus and tropical japonica panels have been genotyped for single nucleotide polymorphism (SNPs), genomic regions were associated with leaf rolling under drought.

## 2 MATERIALS AND METHODS

Two hundred and forty‐two genotypes (Table S1) were selected to comprise the aus panel in this study based on information from the Generation Challenge Program (GCP) composite collection genotyping (GCP, 2005), as well as additional entries known to be in the aus subgroup based on molecular characterization (McCouch et al., 2016). The panel of 176 tropical japonica genotypes (Table S2) for the greenhouse dry‐down study was compiled from the International Rice Research Institute (IRRI) Genebank, CIRAD (France), and collections in Senegal and Mali as described by Rebolledo et al. (2013).

### 2.1 Characterization of the aus panel for drought response in the field

Two hundred and twenty aus genotypes were evaluated in three field studies conducted at the experimental farm of the IRRI, Los Baños, Philippines (14°30′N, 121°15′E) during the dry seasons (January to May) of 2010, 2011, and 2012. The soil was classified as an Isohyperthermic Typic Hapludalf, with an average bulk density of 1.09 and 1.08 g cm^−3^ at depths of 5–10 and 25–30 cm. The experimental design was alpha lattice, with three replicates per genotype in each treatment. In 2012, the genotypes were separated into three groups—early (51–62 days to flowering [DTF], 68 genotypes), mid (62–72 DTF, 144 genotypes), and late flowering (73–83 DTF, 34 genotypes)—to target the timing of DS to occur at reproductive stage in each genotype. Each study included a DS treatment and an irrigated control treatment located in an adjacent field on a lower terrace. Field preparation and crop management were carried out according to Henry, Gowda, Torres, McNally, and Serraj (2011), where the DS fields were maintained flooded until 54 days after sowing (DAS) in 2010, 49 DAS in 2011, and 39, 47, and, 54 DAS in the early, mid, and late flowering groups, respectively, in 2012. In each experiment, three to four seedlings per hill were transplanted with 0.2 m between hills and 0.25 m between rows of 3 m in length, with three rows per plot. Soil moisture was monitored in the drought treatment for soil water potential with 2–6 tensiometers per trial (Soilmoisture Equipment Co., CA, USA) installed at a depth of 30 cm. Volumetric soil moisture at 10‐cm increments to a depth of 70 cm (Diviner 2000, Sentek Sensor Technologies, Stepney, SA, Australia) was measured through 3–6 PVC access tubes installed in the field in 2010 and 2011, and through a total of 86 PVC tubes installed in 2012, to monitor general soil moisture levels as well as the effects of the 26 selected genotypes. The DS treatment was most severe in 2010 as indicated by rainfall, soil water potential, and volumetric water content (Figure S1).

Plots in the DS treatment were monitored for canopy temperature (CT; IR Thermometer 8872 Spectrum Technologies, Inc., Plainfield, IL, USA, 2010, and Model 62 Mini Infrared Thermometer, FLUKE, Everett, WA, USA, 2011–2012), normalized difference vegetation index (NDVI; Greenseeker Hand‐held Sensor, NTech Industries, CA, USA), leaf rolling score (according to IRRI, 1996, in 2010 and 2012 only), days to 50% flowering, and plant height. CT and NDVI measurements are considered as high‐throughput proxies for transpiration and leaf area index, respectively (as reviewed by White et al., 2012). Leaf rolling score is a visual assessment using classifications of 0 (open leaf), 1 (shallow V‐shaped), 3 (deep V‐shaped), 5 (U‐shaped, fully cupped), 7 (O‐shaped, leaf margins touching), and 9 (tightly rolled). The CT and NDVI measurements, as well as the leaf rolling score, were conducted from 10–12 hr on sunny days; each measurement was conducted on a plot‐by‐plot basis, and the CT and NDVI measurements were taken at a distance of ~0.5 m above the canopy. Leaf rolling scores were determined at 74 DAS in 2010 and 73, 74, and 86 DAS in the early, mid, and late groups in 2012. CT was measured on 76 DAS in 2010, 89 DAS in 2011, and 70, 74, and 86 DAS in the early, mid, and late groups in 2012. The change in NDVI (ΔNDVI) between dates represented the reduction in NDVI due to DS (earlier date NDVI–later date NDVI) and was used to represent leaf rolling score (as previously described by Lu et al., 2011) between 68 and 74 DAS in 2010, 83–89 DAS in 2011, and 64–70 DAS in the 2012 early maturing group; 71–74 and 71–81 DAS in the 2012 medium maturing group; and 85–88 DAS in the 2012 late maturing group. In all field studies, grain yield was determined in all plots at maturity by harvesting an area of 1.5 m^2^ and normalizing the grain weight to a 14% moisture content.

### 2.2 Greenhouse lysimeter study to monitor water uptake rates of the aus panel

Two hundred and twenty‐six aus genotypes were grown in both drought and well‐watered (WW) treatments from August to November 2011 in the IRRI lysimeter facility as described by Kijoji et al. (2012). Briefly, lysimeters were constructed from PVC cylinders (18 cm in diameter and 95 cm in height) filled with upland soil (bulk density = 1.1 g cm^−3^), with 20 cm of lowland paddy soil on top. Lysimeters were covered with a plastic sheet around the base of each plant to reduce nontranspirational loss of water, drained at 32 days after germination, and weighed weekly thereafter to determine water uptake rates. Digital images of the shoot of each plant were acquired at the time of weighing to normalize water uptake rates for plant size. Shoots were harvested at 88 days after germination in the WW treatment and 89–91 days after germination (by replicate) in the drought treatment.

### 2.3 Detailed characterization of selected aus genotypes

Twenty‐six genotypes that represented the range of CT and ΔNDVI values were chosen for additional characterization. In both the DS and WW treatments, LWP (three leaves per field plot and one leaf per greenhouse plant; 3000HGBL Plant Water Status Console, Soilmoisture Equipment Corp., CA, USA), leaf rolling score (IRRI, 1996), and stomatal conductance measurements (two leaves per plant/plot; AP4 Porometer, Delta‐T Devices, Cambridge, UK), as well as sampling for abscisic acid (ABA) (two leaves per plant/plot; collected into liquid N_2_ and stored at −80°C until extraction), were conducted concurrently on the individual plots/plants from the subset of 26 selected genotypes at 73, 80, and 106 DAS in the early, mid, and late groups in the field and at 94, 95, and 108 days after germination in the greenhouse. In many cases, leaves were manually unrolled in order to conduct the stomatal conductance measurements. Leaf length, leaf width, leaf area from individual leaves, and shoot dry biomass were determined in drought treatments of the field studies by sampling at 78 DAS in 2010, 86 DAS in 2011, and at 72, 80, and 106 DAS in the early, medium, and late duration groups in 2012. Leaf length, leaf width, and leaf area from individual leaves were determined in the WW and drought treatments of the greenhouse study at 71 DAS. In the field DS treatments, PVC tubes were installed in individual plots of the subset of 26 genotypes to monitor soil water uptake as determined by changes in volumetric soil moisture (Diviner 2000, Sentek Sensor Technologies, Stepney, SA, Australia). Analysis of leaf ABA concentrations was conducted on leaf tissue from the 2012 early duration group and from the greenhouse study by enzyme‐linked immunosorbent assay using plates produced by the Phytohormones Research Institute, China Agricultural University, China, according to the methods described by He (1993). After washing the plates and diluting the samples, absorbance was read on a spectrophotometer at 490 nm. All parameters measured on the 26 selected genotypes were compared with leaf rolling score by Spearman's rho correlation, and direct relationships between physiological parameters were compared by linear regression in R (R Core Team, 2016).

Based on the results from the first three field seasons, a fourth field study (2012WS) was conducted on the eight most contrasting aus genotypes for different combinations of leaf rolling and CT response (Lakhsnikajal, IC27525, UPRB56, Brown Gora, Tak Siah, Dangar, ARC 14088, and Goai). Four replicates of each genotype were planted in two‐row plots in an open field (WW treatment) and in a rolling rainout shelter (DS treatment) at IRRI in the 2012 wet season. Fully expanded leaves were collected at 85 DAS and stored in 70% ethanol until hand sectioning and imaging at 200× with a Zeiss Axioplan 2 compound microscope. Leaf anatomical parameters were measured in ImageJ (see Table S3). Stomatal density was determined from epidermal imprints taken at the midpoint of the leaf blade using clear nail polish and cellophane tape and imaged at 10×. The number of stomata was counted in an area of about 0.01 mm^2^ between small veins.

Finally, leaf samples from a fifth field study (2018DS) were collected from six genotypes (Lakhsnikajal, IC27525, UPRB56, Brown Gora, Dangar, and Goai). Six replicates of each genotype were planted in four‐row plots in an open field (WW treatment) at IRRI in the 2018 dry season. Fully expanded leaves were collected at 110 DAS and stored in 70% ethanol until dehydration and infiltration in a series of ethanol concentrations, embedding in Spurr's resin, sectioning with a microtome, staining with 0.05% Toluidine blue and imaging a bright field microscope (Olympus BX51)  as described by Chatterjee et al. (2016). Total abaxial and adaxial sclerenchyma cell area and number were determined in ImageJ.

### 2.4 Greenhouse dry‐down study of tropical japonica genotypes

Except for leaf rolling scores, the results of the greenhouse dry‐down study of tropical japonica genotypes were previously reported by Rebolledo et al. (2013). Briefly, single rice plants were grown in pots containing 930 g of soil (36.2% clay, 22.5% sand, and 41.5% silt). Each genotype was replicated three times in a randomized complete block design in the greenhouse at IRRI from September to November 2010. The DS treatment was initiated by stopping irrigation when the plants reached the six‐leaf stage and continued until the soil dried to a soil moisture level of 20% of transpirable soil water (FTSW = 0.2), at which time leaf rolling score was observed and plants were harvested. Plants in the WW treatment were maintained flooded throughout the study. Other measurements at the end of the study included last ligulated leaf area (blade width × blade length × 0.725), leaf blade width and length, length of the sheath, specific leaf area, shoot biomass, and plant leaf area. The level of drought tolerance was evaluated in terms of ability to maintain growth of several parameters in the DS treatment compared with the WW treatment ([DS‐WW]/WW), including maintenance of specific leaf area of Leaf Number 7, maintenance of shoot biomass, and maintenance of plant leaf area.

### 2.5 Association analysis

Using the genotype data from the high‐density rice array (McCouch et al., 2016), we performed genome‐wide association study (GWAS) to identify markers/loci associated with leaf rolling scores. For the aus panel data, we filtered the genotype data based on sample and marker call rates (>20%) and minor allele frequency (>0.05) and generated a linkage disequilibrium (LD) pruned dataset, which was used to compute the kinship matrix and principal components and account for possible stratification in the mixed model analysis. We implemented the efficient mixed‐model association expedited (EMMAX; Kang et al., 2010) model in SNP & Variation Suite v8.4.0 (Golden Helix, Inc., Bozeman, MT, USA, www.goldenhelix.com) on the filtered dataset, with the computed kinship matrix as random‐effect component. We computed the Bonferroni correction (BC) and false discovery rate (FDR), which is the ratio of false positives over total rejected multiplied by the probability of making at least one Type I error, from the original *P* values to adjust for multiple testing comparisons, as implemented in SVS (SNP & Variation Suite v8.4.0). We generated the quantile‐quantile and Manhattan plots from the EMMAX *P* values using a custom *qqman* script (Turner, 2014) implemented in the R package. There were no principal components assigned as fixed effect covariates in the final dataset because the data only included the aus population, although the top principal components were investigated.

For the tropical japonica panel data, we used the genotype data from Courtois et al. (2012) and Rebolledo et al. (2015). A total of 16,444 SNPs and a kinship matrix generated with TASSEL V5 (Bradbury et al., 2007) were used to perform the mixed linear model GWAS association model for all phenotypic variables.

Further, we annotated the associated markers from the EMMAX using gene models from the Rice Genome Annotation Project (Kawahara et al., 2013), which were then treated as guide genes in RiceNetv2 (Lee et al., 2015) to explore possible connections between published known genes (also as guide genes).

## 3 RESULTS

### 3.1 Aus genotypes: Leaf rolling under vegetative and reproductive stage drought was not correlated with drought tolerance (CT, maintenance of biomass, or grain yield) but was correlated with leaf morphology

**Figure 1:**
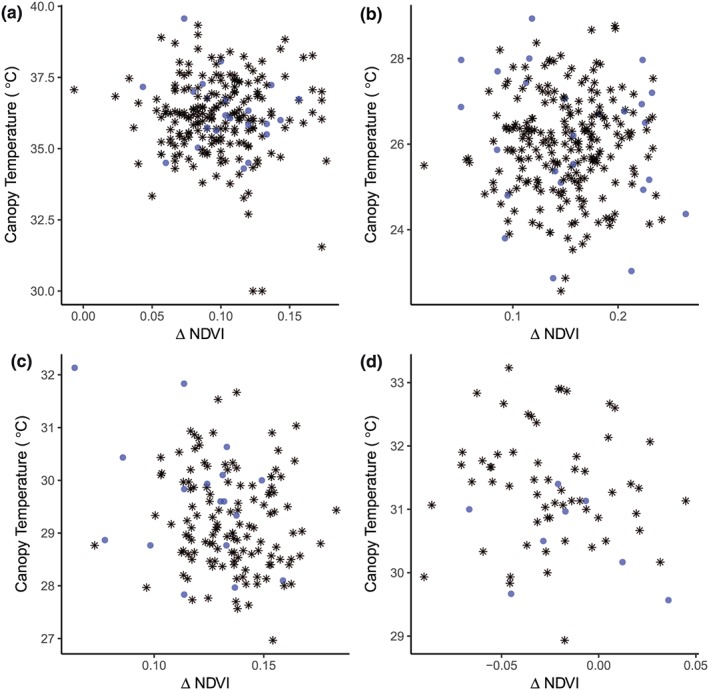
Relationship between ΔNDVI and CT within a panel of ~220 aus rice genotypes in three field dry seasons under drought stress: (a) 2010 (ΔNDVI 68–74 DAS, CT 76 DAS); (b) 2011 (ΔNDVI 83–89 DAS, CT 89 DAS), (c) 2012 early maturing group (ΔNDVI 64–70 DAS, CT 70 DAS); (d) 2012 medium maturing group (ΔNDVI 71–74 DAS, CT 74 DAS). Twenty‐six genotypes selected for detailed measurements from the first field season are indicated by blue circles. Values shown are genotypic means

To test the hypothesis that leaf rolling is related to leaf water status under drought, we compared decreases in canopy cover, measured by NDVI, with CT measurements over three field seasons using a panel of ~220 genotypes from the rice subgroup aus (Table 1). Leaf rolling score (Figure S2) was significantly correlated with ΔNDVI (Table S4). We observed that ΔNDVI was not correlated with CT, except for some dates in 2012 when experiments were grouped by phenology (Table S5). Genotypes separated into four quadrants representing four separate responses (low ΔNDVI + low CT, low ΔNDVI + high CT, high ΔNDVI + low CT, and high ΔNDVI + high CT; Figure 1). The relationship between CT and ΔNDVI was independent of shoot biomass (Table S5). No consistent relationships across seasons were observed between biomass or grain yield reduction by drought and leaf rolling in terms of leaf rolling score or ΔNDVI (Table S6).

**Table 1:** Summary of experiments included in this study, the measurements conducted in each experiment that are reported here, and the relative degree of severity of the drought stress treatment as indicated by the maintenance of biomass or grain yield compared with the well‐watered (WW) control treatment

			Drought stress compared with WW control (DS‐WW)/WW × 100	
Experiment	Number of genotypes	Measurements reported	Biomass	Grain yield
Aus—field				
2010	220	Leaf rolling score, midseason shoot biomass, NDVI, CT, DTF, yield, and biomass at harvest	−10%	−20%
2011	248	Midseason shoot biomass, NDVI, CT, DTF, yield, and straw biomass at harvest	−42%	−50%
Early 2012	68	All genotypes: leaf rolling score, NDVI, CT, DTF, yield, and straw biomass at harvest Selected genotypes: midseason biomass, leaf dimensions, stomatal conductance, leaf water potential, [ABA]_leaf_, volumetric soil moisture	−33%	−30%
Mid 2012	144		−30%	−53%
Late 2012	34		−29%	−63%
2012WS	8	Leaf anatomy, stomatal density, yield, and biomass at harvest	−52%	−84%
2018DS	6	Leaf anatomy	n/a	n/a
Aus—greenhouse lysimeters	226	All genotypes: leaf rolling score Selected genotypes: soil moisture dry‐down, leaf dimensions, stomatal conductance, leaf water potential, [ABA]_leaf_	−38%	n/a
Tropical japonica—greenhouse pots	172	Leaf rolling score, leaf dimensions, specific leaf area, shoot biomass, soil dry‐down rate	−45%	n/a

To further investigate the leaf structural and functional parameters related to leaf rolling, we selected 26 genotypes (denoted by blue circles in Figure 1) representing the diversity of the ΔNDVI/CT response across the four quadrants for more detailed measurements among all the genotypes planted in one field and one greenhouse lysimeter study.

**Figure 2:**
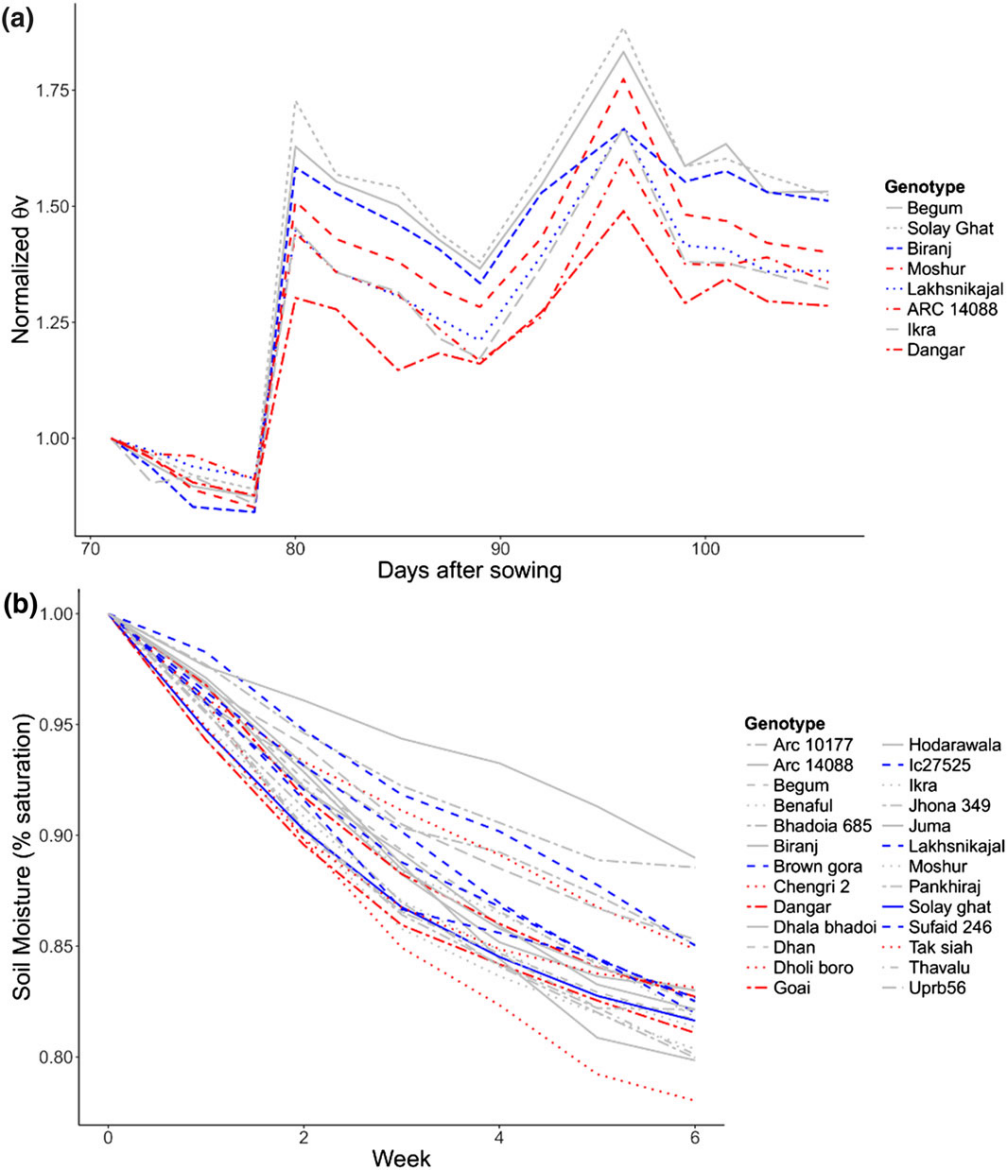
Volumetric soil moisture (θ_v_) in the 2012 early field drought stress treatment (30 cm in depth), normalized for the initial reading in each plot (a), and gravimetric soil moisture (b) in the drought stress treatment of greenhouse lysimeters for selected aus genotypes belonging to different leaf rolling groups (low = blue, high = red) selected based on previous leaf rolling scores and ΔNDVI values. In the field study, genotypes with a greater degree of leaf rolling under drought were ARC 14088, Dangar, and Moshur, and those showing a lesser degree of leaf rolling were Biranj and Lakhsnikajal. In the greenhouse lysimeter study, genotypes with a greater degree of leaf rolling under drought in the greenhouse included Chengri 2, Dhala Bhadoi, Dangar, Goai, and Tak Siah. Genotypes with a lesser degree of leaf rolling in the greenhouse lysimeter study included Brown Gora, IC27525, Lakhsnikajal, Sufaid 246, and Solay Ghat

In a comparison of multiple parameters potentially related to leaf rolling (Figure S3), leaf morphology—particularly leaf width—appeared to be most closely related to leaf rolling score in both the field and greenhouse (Table 2). Narrow leaves tended to roll at less negative LWPs than wider leaves (i.e., narrow leaves rolled earlier over the course of the DS treatment). LWP was related to leaf rolling in the medium maturing group in the field (Table 2B) but not in the greenhouse (Table 2C). In addition to LWP and leaf width, we observed a weak correlation between leaf length and leaf rolling in the greenhouse, although genetic variation for plant height in the aus panel was relatively low. No relationships between leaf rolling (LRS or ΔNDVI) and CT were observed (Figure 1), except for a negative relationship in the 2012 early maturing group among selected contrasting genotypes (Table S5). Furthermore, no relationships between leaf rolling and stomatal conductance or [ABA] were observed. In general, water uptake in the field and greenhouse was less in genotypes with the highest degree of leaf rolling under drought (Figure 2). One exception was genotype Dangar, which showed the highest levels of water uptake and showed the highest degree of leaf rolling in the field and greenhouse. Dangar also showed the lowest CT in the field among selected genotypes.

**Table 2:** Correlation matrices by Spearman's rho correlation of leaf rolling and potentially associated traits under drought stress in (A, B) field and (C) greenhouse lysimeter experiments conducted on ~220 aus genotypes

(A) 2012DS field—early maturing group—measurements taken on 70 DAS									
	LRS	NDVI	ΔNDVI 64–70 DAS	LWP	θ_v_ 30 cm	Leaf width	Leaf length	g_s_	CT
NDVI	−0.1								
ΔNDVI 64–70 DAS	−0.47*	0.26							
LWP	−0.1	0.25	0.02						
θ_v_ 30 cm	−0.17	0.13	0.2	0.13					
Leaf width	−0.52***	0.19	0.23	0.46**	0.16				
Leaf length	0.59***	0.26	−0.27	−0.28	−0.14	−0.2			
g_s_	−0.3	0	0.16	0.08	−0.02	0.14	−0.31		
CT	−0.09	0.18	−0.14	0.71***	0.07	0.5	−0.23	0.27	
[ABA]	−0.06	−0.28	0.11	0.03	−0.19	−0.07	−0.05	−0.12	−0.24

(B) 2012DS field—medium maturing group—measurements taken on 81 DAS								
	LRS	NDVI	ΔNDVI 74–81 DAS	LWP	θ_v_ 30 cm	Leaf width	Leaf length	g_s_
NDVI	−0.17							
ΔNDVI 74–81 DAS	−0.6***	0.35***						
LWP	−0.26*	0.29	0.27					
θ_v_ 30 cm	−0.01	−0.01	0.01	−0.2				
Leaf width	−0.22	−0.02	−0.05	0.09	−0.12			
Leaf length	0.33*	−0.19	−0.05	−0.15	−0.18	0.04		
g_s_	−0.15	0.35***	0.23	0.13	0.05	0.13	−0.25	
CT	−0.01	−0.21	−0.01	0.08	0.16	0.18	−0.05	0.12

(C) Greenhouse lysimeter study—108 DAP												
	LRS	LWP	Soil moisture	Normalized Tr	Absolute Tr	Leaf width	Leaf width ratio	Leaf length	Leaf length ratio	Leaf area	Leaf area ratio	g_s_
LWP	0.04											
Soil Moisture	−0.44*	0.27										
Normalized Tr	−0.26	0.04	0.55***									
Absolute Tr	0.13	0.04	−0.31	0.48*								
Leaf width	−0.4*	−0.19	0.19	0.11	0.01							
Leaf width ratio	0.3	0.03	−0.23	0	0.02	−0.47*						
Leaf length	−0.04	−0.27	−0.2	−0.31	−0.06	0.12	−0.41					
Leaf length ratio	0.07	0.19	0.41	0.54***	−0.09	−0.13	0.27	−0.58				
Leaf Area	−0.28	−0.35	0.06	−0.06	−0.02	0.65***	−0.64***	0.73***	−0.42*			
Leaf area ratio	0.29	0.22	−0.09	0.09	−0.06	−0.31	0.8***	−0.65***	0.64***	−0.69***		
g_s_	−0.23	−0.42	0.3	0.22	−0.3	−0.02	−0.06	0.07	0.22	0.06	−0.06	
[ABA]	0.07	0.07	−0.33	−0.28	−0.13	−0.39	0.35	−0.17	−0.28	−0.32	0.09	0.05

*Note*. Absolute Tr: absolute weekly transpiration rate at 99 DAP; CT: canopy temperature; g_s_: stomatal conductance; LRS: leaf rolling score; LWP: leaf water potential; NDVI: normalized difference vegetation index; Normalized Tr: weekly transpiration rate at 99 DAP normalized to water uptake of the first week of stress; SM: soil moisture level relative to initial soil moisture at the start of the stress treatment; ratio: WW‐DS/WW; θ_v_ 30 cm: volumetric water content at 30 cm depth.

Significant correlations are indicated by

* *P* < 0.05;

** *P* < 0.01;

*** *P* < 0.001.

**Figure 3:**
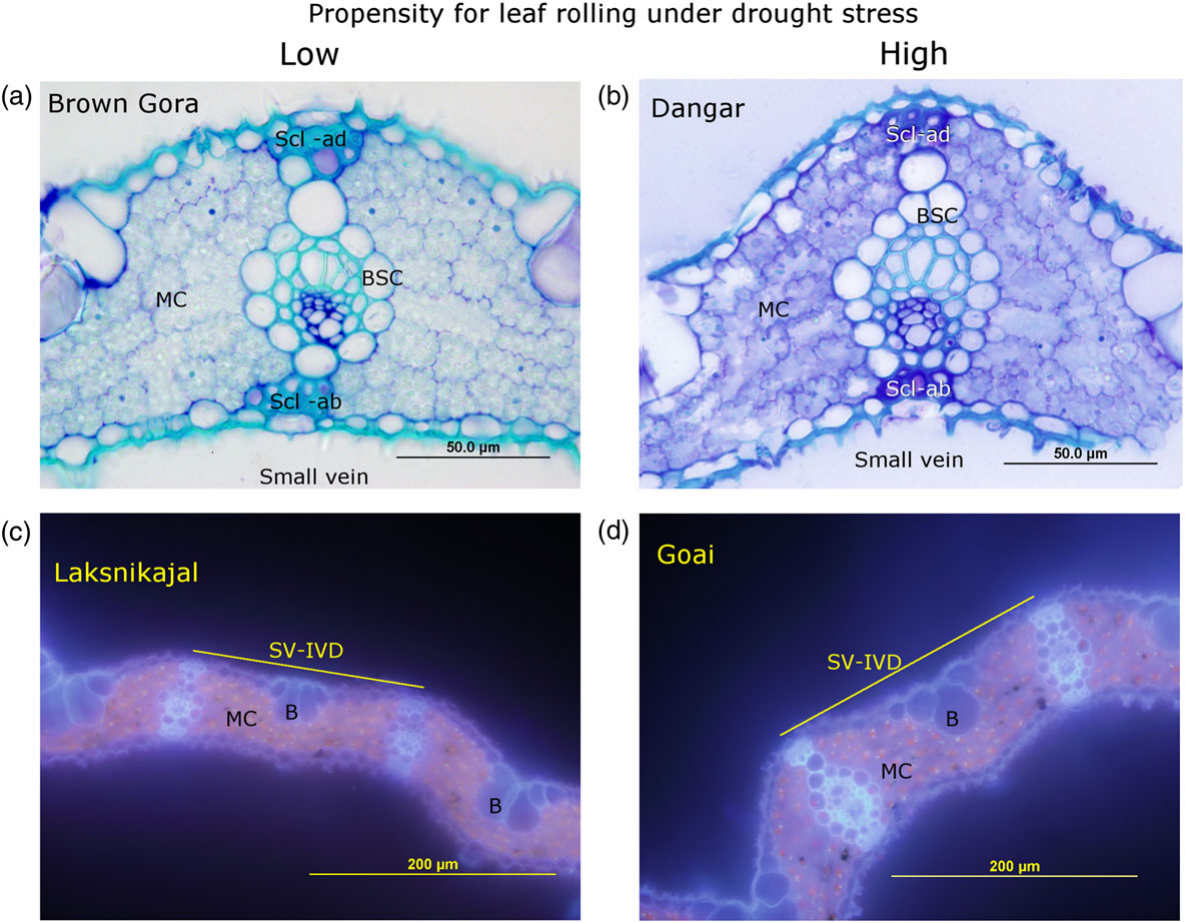
Leaf anatomy of selected aus genotypes contrasting in their propensity for leaf rolling under drought stress. (a,b) Genotypes varying in sclerenchyma cell area and number as measured in the 2018DS well‐watered field study. (c,d) Genotypes varying bulliform cell height and small vein parameters as measured in the drought stress treatment of the 2012WS field study. B, bulliform cell; BSC, bundle sheath cell; Scl‐ab, abaxial sclerenchyma cell; Scl‐ad, adaxial sclerenchyma cell; SV‐IVD, interveinal distance between small veins. Quantitative values are shown in Tables S3, S7, and S8

Significant differences in leaf anatomy were observed among the selected genotypes contrasting for rolling and leaf water status grown in the 2012WS and 2018DS experiments (Figure 3 and Tables S3 and S7), and these genotypic differences depended on the treatment. Bulliform cell height was related to leaf rolling propensity in the WW control treatment only; no significant differences among genotypes were observed between bulliform cell number or size and leaf rolling propensity under drought (Table S8). Stomatal density did not differ significantly among the eight genotypes under DS in the 2012WS experiment (Table S8). Genotype Dangar (which stood out for high transpiration rates despite a high degree of leaf rolling) showed the significantly highest values for a number of anatomical traits including mesophyll cell width and small vein width under drought, as well as low adaxial sclerenchyma cell area (Figure 3 and Tables S3 and S7). In contrast, genotype Brown Gora that showed low leaf rolling scores stood out for having small bulliform cell height and high adaxial sclerenchyma cell area and number (Figure 3 and Tables S3 and S7). However, these trends in leaf anatomy were not observed across all genotypes with either high or low degrees of leaf rolling under drought.

### 3.2 Tropical japonica genotypes: Leaf rolling under seedling stage drought was not correlated with drought tolerance (maintenance of biomass) but was correlated with leaf morphology

In a greenhouse dry‐down study of 172 tropical japonica genotypes, a large distribution in leaf rolling scores was observed (Figure S4). Leaf rolling did not show any significant relationship with measures of drought tolerance, including maintenance of biomass and leaf area under stress compared with the WW control (Table 3). Leaf rolling was not directly related to plant size as indicated by leaf area. However, leaf rolling in the stress treatment was significantly correlated with leaf dimensions (Figure S5) including positively with leaf blade and sheath length and negatively with leaf blade width and specific leaf area (Table 3). No correlation between leaf rolling score and dry‐down rate (time to reach FTSW = 0.2) was observed (Figure S6). Japonica genotypes with the highest leaf rolling scores were Tres Meses and Padi Kasalle, whereas genotypes with the lowest ability to maintain shoot biomass under drought were Kuroka and Yunlu 7 (Table S9).

**Table 3:** Greenhouse dry‐down study conducted on 172 japonica genotypes

	Rolling DS	LLLdim DS	Blade L DS	Blade W DS	Sheath L DS	SLA DS	PLA DS	SDW ratio	LLLdim ratio	Blade L ratio	Blade W ratio	SLA ratio
LLLdim DS	0.09											
Blade L DS	0.29***	0.71***										
Blade W DS	−0.15*	0.76***	0.23**									
Sheath L DS	0.26***	0.51***	0.58***	0.24**								
SLA DS	−0.33***	0.03	−0.11	0.25**	−0.23**							
PLA DS	−0.17*	0.36***	0.12	0.42***	0.02	0.19*						
SDW ratio	−0.14	−0.47***	−0.51***	−0.25***	−0.25***	0.08	−0.35***					
LLLdim ratio	0.12	−0.64***	−0.41***	−0.53***	−0.11	−0.16*	−0.35***	0.57***				
Blade L ratio	0.11	0.54***	0.71***	0.19*	0.25**	−0.08	0.19*	−0.61***	−0.68***			
Blade W ratio	−0.27***	0.36***	0	0.6***	−0.04	0.29***	0.4***	−0.39***	−0.73***	0.23**		
SLA ratio	0.26***	−0.1	0.13	−0.27***	0.15	−0.67***	−0.16*	−0.07	0.25**	0.01	−0.35***	
PLA ratio	0.07	−0.31***	−0.25**	−0.25***	−0.06	−0.02	−0.56***	0.7***	0.53***	−0.45***	−0.48***	0.11

*Note*. Spearman's rho correlation matrix for variables measured in drought‐stressed plants (DS) and calculated variables from both the DS and well‐watered (WW) treatments (ratio: WW‐DS/WW). Blade L DS: length of the blade in DS plants at the end of the experiment; Blade L ratio: reduction in length of the blade (WW‐DS/WW); Blade W DS: width of the blade in DS plants at the end of the experiment; Blade W ratio: reduction in width of the blade (WW‐DS/WW); LLL dim ratio: reduction in last ligulated leaf dimensions (blade width*blade length*0.725) (WW‐DS/WW); LLLdim DS: last ligulated leaf dimensions (blade width*blade length*0.725) measured in DS plants at the end of the experiment; PLA DS: plant leaf area measured in DS plants at the end of the stress treatments FTSW 0.2; Rolling DS: leaf rolling score measured in stressed plants at the end of the experiment (FTSW 0.2); SDWratio: reduction of shoot biomass (WW‐DS/WW); Sheath L DS: length of the sheath in DS plants at the end of the experiment; SLA DS: specific leaf area of Leaf Number 7 measured at the end of the experiment in DS plants; SLA ratio: reduction in specific leaf area leaf area (WW‐DS/WW); SLA ratio: reduction of specific leaf area of Leaf Number 7 (WW‐DS/WW).

*** *P* < 0.001.

** *P* < 0.01.

* *P* < 0.05.

### 3.3 Association analysis revealed genetic regions related to rice (aus and tropical japonica) leaf rolling under drought

**Figure 4:**
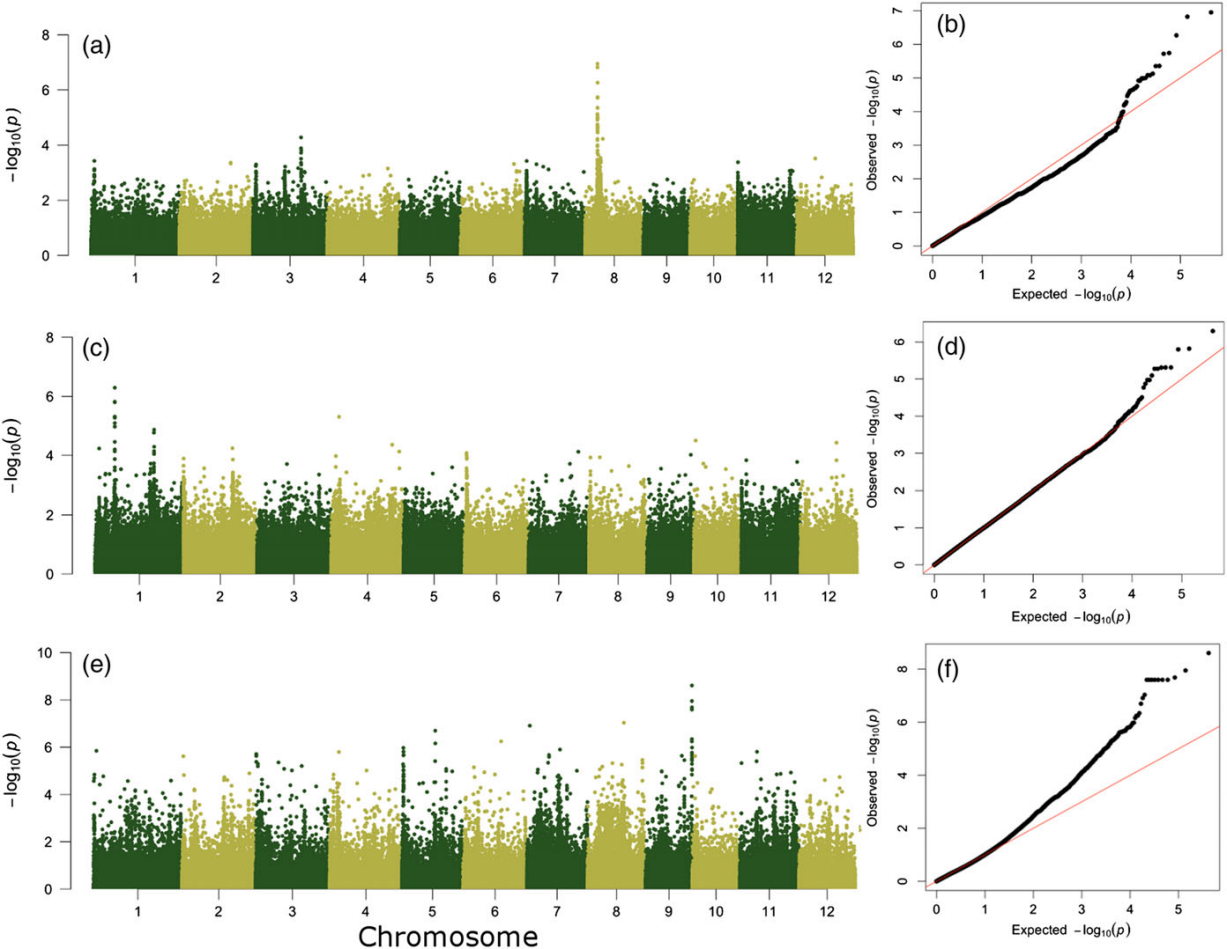
Manhattan plots and quantile‐quantile plots of genome‐wide association analysis in the aus panel for (a,b) leaf rolling score in the greenhouse lysimeter study, (c,d) change in NDVI in the 2011 field drought trial, and (e,f) leaf rolling score in the 2012 field drought trial on medium‐duration genotypes (106 DAS)

Association analysis of leaf rolling under drought was conducted using the available genotypes in the aus panel (41–93 genotypes) for leaf rolling score in the field, lysimeter experiments and ΔNDVI in the field, and in the japonica panel (172 genotypes) for leaf rolling score in the greenhouse (Figures 4 and S7–S8 and Table S10–S12).

The Manhattan plots from EMMAX analysis showed interesting peaks for at least three measurements for leaf rolling, including ΔNDVI from the 2011 field trial, leaf rolling score from the lysimeter study, and leaf rolling score from 106 DAS in the 2012 medium‐duration field trial. The results for ΔNDVI in the 2011 field trial showed heightened peaks on Chromosome 1, with the top markers explaining 0.22–0.24 of the genetic variance (Table S11), with an h^2^ of 0.23, explained by relationship matrix (Table S10), although nonsignificant based on BC and FDR corrections. The signals for leaf rolling score in the lysimeter study were on Chromosomes 3 and 8, with the top markers contributing about 0.42–0.51 of the genetic variance and h^2^ of 4.54 × 10^−5^ from the random‐effect component; only the top few markers on Chromosome 8 were significant based on multiple testing corrections (Table S12). For leaf rolling score from 106 DAS in the 2012 medium‐duration field trial, associated peaks were on Chromosomes 2, 5, 8, and 9, with the top markers explaining about 0.36–0.45 of the genetic variance and an h^2^ of 0.99. Both BC and FDR for leaf rolling score from the 2012 field trial support the significant associations (<0.01–0.005) for the top markers (Table S12). Within these most significant peaks, a list of seven candidate loci for leaf rolling under drought in the aus panel was identified (Table S13). The effects of marker density and correlation were also investigated for the traits, with only the measurement of leaf rolling score from the 2012 field trial showing an improved quantile‐quantile plot for marker‐trait association (Figure S9). Several other peaks were also observed for the 2010 field ΔNDVI and 2012 medium‐duration field trial leaf rolling score at 74 DAS; however, the −log10(*P* value) of these peaks was generally <5.

A total of 172 tropical japonica genotypes were analyzed using GWAS, of which the genetic data were previously described (Courtois et al., 2012; Rebolledo et al., 2015). Using TASSEL, the mixed linear model using a kinship correction was selected to identify significant GWAS associations. There was not a colocation between rolling and other morphological leaf‐related variables (leaf area and leaf dimensions) or biomass according to the SNP map analysis within a LD of ±20 kb as described by Rebolledo et al. (2015). The highest GWAS associations (*P* > 4.1E‐04) were observed in Chromosome 2 position 5,749,994, Chromosome 6 position 8,345,115, and Chromosome 10 position 5,809,774. A total of seven genes were in the LD region (20 Kb) for the GWAS peak on Chromosome 2, three genes were in the LD region for GWAS peak on Chromosome 10, and only one gene for GWAS peak on Chromosome 6 (Table S14).

We explored how previously reported genes from the literature might be linked to the candidate genes from the aus and tropical japonica GWAS using RiceNet v2 (Lee et al., 2015) using the identified candidate genes from each panel and loci previously reported to be involved in leaf rolling as seed loci (Tables S15 and S16). For the aus study, this investigation of annotated markers (Table S17) revealed that LOC_Os09g23200 (SLL; SHALLOT‐LIKE1, Zhang, Xu, Zhu, & Xue, 2009) and LOC_Os12g36430 (2010 field ΔNDVI, −log10 [*P* value] = 5.73) are involved in regulation of transcription, multicellular organismal development, and abaxial cell fate specification. LOC_Os02g45250 (ROC5, Zou et al., 2011), LOC_Os04g04020 (protein transport protein Sec24‐like, putative, expressed; 2012 Med field leaf rolling score at 74 DAS), LOC_Os03g57300 (tetratricopeptide repeat‐like superfamily protein, likely component of TRAPP complex, TRS85; 2012 Med field leaf rolling score at 74 DAS), LOC_Os06g36850 (cysteine synthase, putative, expressed; 2010 field ΔNDVI), and LOC_Os09g39670 (oxidoreductase, short chain dehydrogenase/reductase family domain containing family, expressed; 2012 medium‐duration field trial field leaf rolling score at 106 DAS) are involved in intracellular protein transport, ER to Golgi vesicle‐mediated transport, and ER body organization. For the tropical japonica study, annotated markers (Table S18) showed that LOC_Os02g10800 is involved in regulation of transcription, leaf development, and response to stress; LOC_Os02g108010 in translation, adaxial/abaxial pattern formation, leaf morphogenesis, and response to stress; LOC_Os02g108030 in response to stress and protein/amino acid metabolism; and LOC_Os02g108050 in protein folding and stress response. The locus on Chromosome 6, LOC_Os06g14750 is involved in cytoskeleton organization and response to stress. Expression data from RNA‐Seq on drought experiments conducted on Nipponbare curated in the TENOR (Kawahara et al., 2016) and IC4R‐RED (Xia et al., 2017) databases were examined at the candidate gene with expression evidence given in Table S13 (aus) and Table S14 (tropical japonica). Although LOC_Os09g23200 (Shallot like 1) showed low expression in roots and shoots, it was found to be down regulated under drought. Other loci showed differential expression between tissues in the wild type and between stress and control treatments, consistent with the possible involvement in drought responses we have observed.

## 4 DISCUSSION

Soil drying causes leaf rolling in rice, but results from this study on two diversity panels of 170–220 genotypes indicate that rice genetic variation in leaf rolling under drought is more correlated with leaf morphology than with leaf water status or with drought tolerance as defined by grain yield or maintenance of shoot biomass.

The results from the field and greenhouse lysimeter studies of the aus panel suggest that stomata can remain open in rolled rice leaves under DS and that leaf rolling is not consistently directly related to other drought response parameters. No correlations between stomatal conductance and leaf rolling were observed (Table 2), and some plants maintained moderate levels of stomatal conductance/low CT despite a high degree of leaf rolling (Figure 1). In the aus panel, drought response parameters such as CT, LWP, and ABA levels were not consistently correlated with the degree of leaf rolling (Table 2). Likewise, in the japonica panel, drought tolerance (as reflected by the ability to maintain shoot biomass, leaf area, and specific leaf area under drought as compared with WW conditions) was not correlated with the degree of leaf rolling. These results point to the predominance of constitutive traits (such as plant type) rather than responsive traits (such as leaf water status) in controlling leaf rolling under drought. The lack of agreement between this study and previous reports of a strong relationship between leaf rolling and leaf water status may be due to the fewer number of genotypes used in those prior studies that were highly divergent in hydraulic traits driving leaf rolling differences, although the relative variation in hydraulics across the large diversity panels used here were more subtle.

The leaf morphological parameters of length and width were highly correlated with leaf rolling in the DS treatments of both the aus and japonica panels (Tables 2 and 3). The absolute values of the leaf dimensions showed the strongest relationships with leaf rolling under drought, rather than the relative change in leaf dimensions due to DS (Tables 2C and 3). In the aus panel leaf anatomical analysis, genotypic differences in bulliform cell height, small vein parameters, and sclerenchyma cell area were observed but did not appear to be consistently related to leaf rolling under drought. Mutations in all of these cell types have been previously reported to affect leaf rolling in rice (Zou, Zhang, Qi, Peng, & Lu, 2014). Therefore, it is possible that the propensity of a given genotype to show leaf rolling under drought results from the combined effects of leaf morphological and anatomical traits in different combinations.

The use of ΔNDVI has been used previously to represent leaf rolling in maize (Lu et al., 2011) and was an effective proxy for leaf rolling score, although our approach differed from that of Lu et al. (2011) in that their ΔNDVI calculations compared morning and afternoon NDVI measurements on the same day, whereas our ΔNDVI values were calculated across 3–10 days at the same time of day. In the case of the present study on rice diversity panels, we found this approach to be appropriate because the most drought sensitive genotypes tended not to unroll in the morning, as described by Singh and Mackill (1991) and therefore would not have correlated with ΔNDVI when measured across multiple times on the same day.

The use of ΔNDVI resulted in more resolution for statistical analyses as it is a continuous variable rather than a categorical variable such as leaf rolling score. In some cases when leaves rolled early in the DS period, smaller changes in NDVI were observed in subsequent measurements, which resulted in a negative relationship between ΔNDVI and LRS (for example, as observed in the late‐maturing group of the 2012DS field study; Table S4). Furthermore, because our ΔNDVI measurements were conducted at slightly different growth stages in each experiment, the values reflect a combination of DS and growth effects, as indicated by the negative ΔNDVI values in 2012 (Figure 1). Senescence effects related with maturity were less likely because the ΔNDVI measurements were not conducted past early reproductive stage.

Although many functional drought response parameters in this study were not correlated with leaf rolling, a strong negative relationship was observed between leaf rolling and cumulative water uptake in the 2012DS field study and in the greenhouse lysimeter study (Figure 2), indicating that although stomates may be open when leaves are rolled as detected by instantaneous measurements, the boundary layer created by rolling may effectively reduce water uptake over the long term. However, not all genotypes followed this trend, for example, in the case of genotype Dangar. Furthermore, the lack of correlation between leaf rolling score and dry‐down rate in the japonica study may be due to the short duration of that study in which leaves were rolled for shorter lengths of time. A previous examination of the relationship between leaf rolling and water uptake in the field did not detect differences in depletion of soil water among genotypes with different leaf rolling using a neutron probe (Turner et al., 1986), but this may have been due to methodological limitations in the measurements or spatial variation in the field. Interestingly, root growth has been suggested to be related to stomatal conductance and not leaf rolling (Dingkuhn et al., 1999). Clearly, differences in root structure and function may explain a large proportion of genetic variation in drought response and are the subject of future analyses of the aus and tropical japonica panels.

Because leaf rolling is a biomechanical response to drought, it is logical that morphological properties affecting the force required to roll a leaf will affect that response. Although the onset of DS signalling in rice that may precede leaf rolling is highly complex (Shinozaki & Yamaguchi‐Shinozaki, 2007), different genotypes may send a similar signal to the bulliform cells to initiate leaf rolling, and the force of the response to that signal will be mechanically impeded to different degrees depending on leaf morphology. Rice molecular genetic studies using mutants have identified several genes involved in leaf rolling in rice; all of which appear to influence aspects of leaf morphology and anatomy, including bulliform cells (Hu et al., 2010; Li et al., 2010; Zhang et al., 2009). However, the lack of genetic variation in bulliform cell size and number among the most contrasting aus genotypes in this study suggests that this parameter is not the strongest factor controlling natural genetic variation in rice leaf rolling under drought.

Although the genes related to leaf rolling based on mutant studies have been identified under WW conditions, it is possible that these genes underlie susceptibility to drought‐induced leaf rolling. Two known loci, qLRC‐1 (You et al., 2006) and qRL7 (Xu, Zhong, Yu, Luo, & Li, 1999), associated with leaf rolling were recovered from our analysis (Figure S7 and Table S12). qLRC‐1 was consistently identified for all three measurements of ΔNDVI, although the −log10 (*P* value) ranged from 4.13 to 4.87. qRL7, on the other hand, was recovered from leaf rolling score in the 2012 field trial at 74 DAS and ΔNDVI from the 2011 experiment, with −log10 (*P* value) of 4.81 and 4.12, respectively. The relaxed threshold allowed detection of more markers possibly associated with leaf rolling. Although the number of samples included for GWA was limited in the greenhouse lysimeter study, we could identify marker–trait associations and corroborate previous findings due to the selection of contrasting lines for that measurement (i.e., only those genotypes observed to be most contrasting for leaf rolling under drought in the field were scored in the lysimeter study). Moreover, annotations for markers for all trait measures above the −log10 (*P* value) = 4 threshold for leaf rolling included quantitative trait loci (QTLs) previously reported for root descriptors, chlorophyll content, osmotic adjustment, relative water content, number of vascular bundles, and drought response (Table S12). Cross‐referencing linked loci from RiceNet v2 between the two studies (Tables S17 and S18) revealed common functions the candidate genes may play a role in such as stress response, microtubule cytoskeleton organization, translation, adaxial/abaxial pattern formation, and leaf morphogenesis. Genes important for adaxial leaf rolling were identified from the RiceNet analysis, consistent with the observation that all leaf rolling observed in these experiments was adaxial leaf rolling. In summary, identifying common inferred interactions support the roles of leaf morphology and cell development in the physiological response of leaf rolling.

Although the number of individuals included in our GWA (<100) was too limited to provide more statistical power to detect small effects, our results show QTLs and loci linked to genes directly involved in rice leaf rolling. Increasing the number of samples and adding evidence from transcriptomics will be vital in capturing signals of expressed genes during rolling and enable detection of small additive effects of complex traits with low heritability, such as is the case of leaf rolling under DS in the field. Nevertheless, the candidate genes and biochemical pathways (for example, number of vascular bundles and abaxial cell fate specification) identified to be genetically associated with leaf rolling under drought in this study may further support the conclusion based on physiological observations that leaf morphology, rather than plant water status, underlies the genetic variation of rice leaf rolling under drought.

## CONFLICT OF INTEREST

The authors have no conflict of interest to declare.

## Supporting information

Table S1. Genotypes included in the aus experiments in the field and greenhouse.
Table S2. Genotypes included in the tropical japonica greenhouse experiment.
Table S3. Leaf anatomical parameters measured in eight selected aus genotypes in field drought stress and well‐watered treatments.
Table S4. Correlations between the change in normalized difference vegetation index (ΔNDVI) and leaf rolling score (LRS) in the 2010 and 2012 field experiments, based on Spearman's rank correlation.
Table S5. Relationships among canopy temperature (CT) and the change in normalized difference vegetation index (ΔNDVI) or and shoot biomass, based on ANOVA on a panel of 226 aus rice genotypes under drought conditions in 3 field studies during the dry season of 2010, 2011, 2012.
Table S6. Relationships among leaf rolling (leaf rolling score ΔNDVI) with maintenance of biomass and grain yield under drought in the aus field and greenhouse experiments, based on correlation (Spearman's for leaf rolling traits, Pearson for ΔNDVI).
Table S7. Sclerenchyma cell area and number in six selected aus genotypes in the 2018DS field well‐watered treatment. Letter groups indicate significant differences among genotypes (*p* < 0.05).
Table S8. Bulliform cell size and number, as well as stomatal density, in eight selected aus genotypes in field drought stress and well‐watered treatments.
Table S9. The most contrasting genotypes from the japonica panel in terms of leaf rolling score and maintenance of shoot biomass under drought as compared to that under well‐watered conditions (SDWratio, calculated as (DS‐WW/WW)).
Table S10. Traits for which association analysis was conducted on genotypes with available sequence data.
Table S11. List of top markers (−log10(P‐value) > 4.0) from association mapping using EMMAX model for leaf rolling scores and ΔNDVI from different experiments.
Table S12. List of markers with annotations from gene models following the Rice Genome Annotation Project (Kawahara *et al*., 2013) and OGRO/Q‐TARO database (Yamamoto *et al*., 2012).
Table S13. Aus candidate loci for leaf rolling under drought and their annotations.
Table S14. Tropical japonica candidate loci for leaf rolling under drought and their annotations.
Table S15. Aus seed loci used for identification of genes in networks for leaf rolling under drought.
Table S16. Aus seed loci used for identification of genes in networks for leaf rolling under drought.
Table S17. Loci from the aus panel predicted by RiceNet2 to be involved in networks related leaf rolling under drought.
Table S18. Loci from the tropical japonica panel predicted to be involved in networks related leaf rolling under drought.
Figure S1. Rainfall and soil water potential (30 cm depth) from the drought treatments in the aus field studies.
Figure S2. Distributions of mean leaf rolling scores of aus genotypes in the field and greenhouse lysimeter experiments.
Figure S3. Leaf morphology distributions among 26 selected genotypes in the aus panel grown in the greenhouse lysimeter study. The youngest fully expanded leaf at 71 DAS was measured to determine A) leaf area, B) leaf length, C) leaf width, and D) specific leaf area.
Figure S4. Distribution of mean leaf rolling score of 172 tropical japonica genotypes at the end of the greenhouse study, when the soil moisture level reached FTSW of 0.2.
Figure S5. Leaf morphology distributions among tropical japonical panel genotypes grown in the greenhouse. The last ligulated leaf was measured when the soil dried to FTSW = 0.2 to determine A) leaf area, B) leaf length, C) leaf width, and D) specific leaf area.
Figure S6. Relationship between mean leaf rolling score of 156 tropical japonica genotypes at the end of the greenhouse study and the time required for the soil moisture level of each genotype to reach FTSW of 0.2.
Figure S7. Manhattan plots and Quantile‐Quantile plots of genome‐wide association analysis in the aus panel for A‐B) leaf rolling score in the 2010 field drought trial; C‐D) leaf rolling score in the 2012 field drought trial on medium‐duration genotypes (74 DAS); E‐F) change in NDVI in the 2010 field drought trial; G‐H) change in NDVI in the 2012 field drought trial on medium‐duration genotypes.
Figure S8. Manhattan plot and Quantile‐Quantile plot of genome‐wide association analysis of leaf rolling score in the greenhouse study of 172 tropical japonica genotypes.
Figure S9. Quantile‐Quantile plot of genome‐wide association analysis in the aus panel leaf rolling score in the 2012 field drought trial on medium‐duration genotypes (106 DAS), adjusted for effects of marker density and correlation.
