# Supplementary material for: Leaf morphology, rather than plant water status, underlies genetic variation of rice leaf rolling under drought
Source: Plant Cell Environ. 2019 Feb 20;42(5):1532–44. doi: 10.1111/pce.13514 (PMC6487826; doi:10.1111/pce.13514)
Supplement: Supplementary file 1 — Table S1. Genotypes included in the aus experiments in the field and greenhouse. Table S2. Genotypes included in the tropical japonica greenhouse experiment. Table S3. Leaf anatomical parameters measured in eight selected aus genotypes in field drought stress and well‐watered treatments. Table S4. Correlations between the change in normalized difference vegetation index (ΔNDVI) and leaf rolling score (LRS) in the 2010 and 2012 field experiments, based on Spearman's rank correlation. Table S5. Relationships among canopy temperature (CT) and the change in normalized difference vegetation index (ΔNDVI) or and shoot biomass, based on ANOVA on a panel of 226 aus rice genotypes under drought conditions in 3 field studies during the dry season of 2010, 2011, 2012. Table S6. Relationships among leaf rolling (leaf rolling score ΔNDVI) with maintenance of biomass and grain yield under drought in the aus field and greenhouse experiments, based on correlation (Spearman's for leaf rolling traits, Pearson for ΔNDVI). Table S7. Sclerenchyma cell area and number in six selected aus genotypes in the 2018DS field well‐watered treatment. Letter groups indicate significant differences among genotypes (p < 0.05). Table S8. Bulliform cell size and number, as well as stomatal density, in eight selected aus genotypes in field drought stress and well‐watered treatments. Table S9. The most contrasting genotypes from the japonica panel in terms of leaf rolling score and maintenance of shoot biomass under drought as compared to that under well‐watered conditions (SDWratio, calculated as (DS‐WW/WW)). Table S10. Traits for which association analysis was conducted on genotypes with available sequence data. Table S11. List of top markers (−log10(P‐value) > 4.0) from association mapping using EMMAX model for leaf rolling scores and ΔNDVI from different experiments. Table S12. List of markers with annotations from gene models following the Rice Genome Annotation Project (Kawahara et al., 2013) and O [file PCE-42-1532-s001.zip › pce13514.pdf]

Supp. Table S1. Genotypes included in the aus experiments in the field and greenhouse

**Genetic St**	**GS variety**	**Country o**	**GS source**	**From studies**
IRGC 1172	DULAR	India	IRGC 3256	GCP, 2005; McCouch et al., 2016
IRGC 1172	FR 13 A	India	IRGC 6144	GCP, 2005; McCouch et al., 2016
IRGC 1172	N 22	India	IRGC 4819	GCP, 2005; McCouch et al., 2016
IRGC 1172	RAYADA	Banglades	IRGC 7721	GCP, 2005; McCouch et al., 2016
IRGC 1174	ARC 1031	India	IRGC 1243	GCP, 2005; McCouch et al., 2016
IRGC 1174	AUS JOTA	Banglades	IRGC 6676	GCP, 2005; McCouch et al., 2016
IRGC 1174	BARAN BO	Banglades	IRGC 2750	GCP, 2005; McCouch et al., 2016
IRGC 1174	CHUAN 4:	Taiwan	IRGC 1705	GCP, 2005; McCouch et al., 2016
IRGC 1174	DA 16::IRC	Banglades	IRGC 6245	GCP, 2005; McCouch et al., 2016
IRGC 1174	DA 28::IRC	Banglades	IRGC 6246	GCP, 2005; McCouch et al., 2016
IRGC 1174	DA 8::IRG	Banglades	IRGC 6422	GCP, 2005; McCouch et al., 2016
IRGC 1174	GOAI::IRG	Banglades	IRGC 4918	GCP, 2005; related sample in McCouch et al., 2016
IRGC 1174	JC 178::IR	India	IRGC 9080	GCP, 2005
IRGC 1175	JHONA 26	Pakistan	IRGC 2796	GCP, 2005; McCouch et al., 2016
IRGC 1175	JHONA 34	India	IRGC 6307	GCP, 2005; related sample in McCouch et al., 2016
IRGC 1175	KALAMKA	India	IRGC 4597	GCP, 2005; McCouch et al., 2016
IRGC 1175	KALUKAN	Sri Lanka	IRGC 7755	GCP, 2005
IRGC 1175	MIRITI::IR	Banglades	IRGC 2590	GCP, 2005; McCouch et al., 2016
IRGC 1175	PETA::IRG	Indonesia	IRGC 3257	GCP, 2005; McCouch et al., 2016
IRGC 1175	TCHAMPA	Iran	IRGC 3236	GCP, 2005; McCouch et al., 2016
IRGC 1175	TEPI BORO	Banglades	IRGC 2751	GCP, 2005; McCouch et al., 2016
IRGC 1208	AUS 196::	Banglades	IRGC 2901	GCP, 2005; McCouch et al., 2016
IRGC 1208	DHAL KAC	Banglades	IRGC 6677	GCP, 2005; McCouch et al., 2016
IRGC 1208	DHALA BH	Banglades	IRGC 6677	GCP, 2005; McCouch et al., 2016
IRGC 1208	MERY::IRC	Banglades	IRGC 3472	GCP, 2005; McCouch et al., 2016
IRGC 1208	NARIKEL J	Banglades	IRGC 6479	GCP, 2005; McCouch et al., 2016
IRGC 1208	SHANKAR	India	IRGC 7477	GCP, 2005; McCouch et al., 2016
IRGC 1208	182::IRGC	Pakistan	IRGC 2842	GCP, 2005; McCouch et al., 2016
IRGC 1208	29 A 2::IR	Pakistan	IRGC 2836	GCP, 2005; McCouch et al., 2016
IRGC 1208	ANGALAIS	Senegal	IRGC 5656	GCP, 2005; McCouch et al., 2016
IRGC 1208	ARC 1031	India	IRGC 4257	GCP, 2005; McCouch et al., 2016
IRGC 1208	ARC 1037	India	IRGC 2088	GCP, 2005; related sample in McCouch et al., 2016
IRGC 1208	ARC 1095	India	IRGC 1268	GCP, 2005; McCouch et al., 2016
IRGC 1208	ARC 1175	India	IRGC 2161	GCP, 2005; McCouch et al., 2016
IRGC 1208	ARC 1207	India	IRGC 4100	GCP, 2005; McCouch et al., 2016
IRGC 1208	ARC 1534	India	IRGC 4201	GCP, 2005; McCouch et al., 2016
IRGC 1208	ASWINA 3	Banglades	IRGC 6591	related sample in McCouch et al., 2016
IRGC 1208	AUS 257::	Banglades	IRGC 2904	GCP, 2005; McCouch et al., 2016
IRGC 1208	AUS 41::IF	Banglades	IRGC 2890	GCP, 2005; McCouch et al., 2016
IRGC 1208	AUS 439::	Banglades	IRGC 2922	GCP, 2005; McCouch et al., 2016
IRGC 1208	AUS 55::IF	Banglades	IRGC 2891	GCP, 2005; McCouch et al., 2016
IRGC 1208	BAILAM::I	Banglades	IRGC 2583	GCP, 2005; McCouch et al., 2016

Do not distribute

IRGC 1208	BAMOIA 3	Banglades	IRGC 6538	GCP, 2005; McCouch et al., 2016
IRGC 1208	BANS 4::IF	India	IRGC 7472	GCP, 2005; McCouch et al., 2016
IRGC 1208	BATHURI:	Banglades	IRGC 2583	GCP, 2005; McCouch et al., 2016
IRGC 1208	BAULAN::	Banglades	IRGC 6476	GCP, 2005; McCouch et al., 2016
IRGC 1208	BAWOI::IF	Banglades	IRGC 3473	GCP, 2005; McCouch et al., 2016
IRGC 1208	BENAFUL:	Banglades	IRGC 2583	GCP, 2005; McCouch et al., 2016
IRGC 1208	BENAMUF	Banglades	IRGC 2584	GCP, 2005; McCouch et al., 2016
IRGC 1208	BHADOIA	Banglades	IRGC 6588	GCP, 2005; McCouch et al., 2016
IRGC 1208	BIRANJ::IF	India	IRGC 7473	GCP, 2005; McCouch et al., 2016
IRGC 1208	BJ 1::IRGC	India	IRGC 3711	GCP, 2005; related sample in McCouch et al., 2016
IRGC 1208	BLACK GO	India	IRGC 4027	GCP, 2005; related sample in McCouch et al., 2016
IRGC 1208	BOILAN::I	Banglades	IRGC 4902	GCP, 2005; McCouch et al., 2016
IRGC 1209	BOLIUM::I	Banglades	IRGC 3157	GCP, 2005; McCouch et al., 2016
IRGC 1209	BOTESHA\	Banglades	IRGC 3468	GCP, 2005; McCouch et al., 2016
IRGC 1209	CHENGRI	Banglades	IRGC 7722	GCP, 2005; McCouch et al., 2016
IRGC 1209	CHENGRI	Banglades	IRGC 6677	GCP, 2005; McCouch et al., 2016
IRGC 1209	CHIADI N/	India	IRGC 5210	GCP, 2005; McCouch et al., 2016
IRGC 1209	CHIKON S	Banglades	IRGC 6477	GCP, 2005; McCouch et al., 2016
IRGC 1209	CHUNGUF	Banglades	IRGC 2585	GCP, 2005; McCouch et al., 2016
IRGC 1209	CODE NO	Japan	IRGC 4537	GCP, 2005; McCouch et al., 2016
IRGC 1209	CROSS 4-2	India	IRGC 4533	GCP, 2005; McCouch et al., 2016
IRGC 1209	CTG 1516:	Banglades	IRGC 8704	related sample in McCouch et al., 2016
IRGC 1209	DAINY::IR	Nepal	IRGC 5894	GCP, 2005; McCouch et al., 2016
IRGC 1209	DANGAR::	India	IRGC 7629	GCP, 2005; McCouch et al., 2016
IRGC 1209	DD 62::IR	Banglades	IRGC 8626	related sample in McCouch et al., 2016
IRGC 1209	DHALA BA	Banglades	IRGC 7723	GCP, 2005; McCouch et al., 2016
IRGC 1209	DHALI KH/	Banglades	IRGC 6053	GCP, 2005; McCouch et al., 2016
IRGC 1209	DHARIA B	Banglades	IRGC 6477	GCP, 2005; McCouch et al., 2016
IRGC 1209	DHARIA::I	Banglades	IRGC 6477	GCP, 2005; McCouch et al., 2016
IRGC 1209	DHEKI SH/	Banglades	IRGC 6477	GCP, 2005; McCouch et al., 2016
IRGC 1209	DJ 123::IR	Banglades	IRGC 8455	related sample in McCouch et al., 2016
IRGC 1209	DJ 24::IRG	Banglades	IRGC 8506	related sample in McCouch et al., 2016
IRGC 1209	DK 12::IR	Banglades	IRGC 8581	related sample in McCouch et al., 2016
IRGC 1209	DM 43::IR	Banglades	IRGC 8769	related sample in McCouch et al., 2016
IRGC 1209	DNJ 140::I	Banglades	IRGC 8428	related sample in McCouch et al., 2016
IRGC 1209	DUDHI SA	Nepal	IRGC 1626	GCP, 2005; McCouch et al., 2016
IRGC 1209	DV 123::IF	Banglades	IRGC 8861	related sample in McCouch et al., 2016
IRGC 1209	DZ 78::IRG	Banglades	IRGC 8555	related sample in McCouch et al., 2016
IRGC 1209	FULKATI::I	Banglades	IRGC 6678	GCP, 2005; McCouch et al., 2016
IRGC 1209	GADRA::IF	Pakistan	IRGC 7309	GCP, 2005; McCouch et al., 2016
IRGC 1209	GARIA::IR	Banglades	IRGC 2585	GCP, 2005; McCouch et al., 2016
IRGC 1209	GERDEH::	Iran	IRGC 3230	GCP, 2005; related sample in McCouch et al., 2016
IRGC 1209	GOCHI BO	Banglades	IRGC 6678	GCP, 2005; McCouch et al., 2016
IRGC 1209	GORBAI::I	Banglades	IRGC 6679	GCP, 2005; McCouch et al., 2016

Do not distribute

IRGC 1209	GUL MUR	Banglades	IRGC 6679	GCP, 2005; McCouch et al., 2016
IRGC 1209	HABIGON	Banglades	IRGC 1087	GCP, 2005; McCouch et al., 2016
IRGC 1209	HARBHOC	India	IRGC 7474	GCP, 2005; McCouch et al., 2016
IRGC 1209	HEENBAL	Sri Lanka	IRGC 8942	GCP, 2005; McCouch et al., 2016
IRGC 1209	HEGRA::IF	India	IRGC 5244	GCP, 2005; McCouch et al., 2016
IRGC 1209	HODARAV	Sri Lanka	IRGC 6763	GCP, 2005; McCouch et al., 2016
IRGC 1209	HOLOI BA	Banglades	IRGC 6477	GCP, 2005; McCouch et al., 2016
IRGC 1209	IKRA::IRG	Banglades	IRGC 7725	GCP, 2005; McCouch et al., 2016
IRGC 1209	JAMBALI E	Pakistan	IRGC 7310	GCP, 2005; McCouch et al., 2016
IRGC 1209	JAMBALI::	Pakistan	IRGC 7310	GCP, 2005
IRGC 1209	JAMRI::IR	Banglades	IRGC 3711	GCP, 2005; McCouch et al., 2016
IRGC 1209	JHUM BE	Banglades	IRGC 2586	GCP, 2005; McCouch et al., 2016
IRGC 1210	JHUM FUI	Banglades	IRGC 2586	GCP, 2005; McCouch et al., 2016
IRGC 1210	KAL SHON	Banglades	IRGC 6478	GCP, 2005; McCouch et al., 2016
IRGC 1210	KALIA::IRG	Banglades	IRGC 3469	GCP, 2005; McCouch et al., 2016
IRGC 1210	KALIBORC	Banglades	IRGC 7720	GCP, 2005; McCouch et al., 2016
IRGC 1210	KALO KUC	Banglades	IRGC 6680	GCP, 2005; McCouch et al., 2016
IRGC 1210	KALU A 30	Sri Lanka	IRGC 4974	GCP, 2005; McCouch et al., 2016
IRGC 1210	KANGRO::	Pakistan	IRGC 7310	GCP, 2005; McCouch et al., 2016
IRGC 1210	KARKATI 8	Banglades	IRGC 6618	GCP, 2005; related sample in McCouch et al., 2016
IRGC 1210	KASAPUR:	India	IRGC 7475	GCP, 2005; McCouch et al., 2016
IRGC 1210	KAT GIMI:	Banglades	IRGC 6478	GCP, 2005; McCouch et al., 2016
IRGC 1210	KELE BARI	India	IRGC 3498	GCP, 2005; McCouch et al., 2016
IRGC 1210	KELEE::IRG	Banglades	IRGC 6680	GCP, 2005; McCouch et al., 2016
IRGC 1210	KHARAI M	Banglades	IRGC 6680	GCP, 2005; McCouch et al., 2016
IRGC 1210	KHARSU 8	Pakistan	IRGC 2801	GCP, 2005; McCouch et al., 2016
IRGC 1210	KOI MURA	Banglades	IRGC 6681	GCP, 2005; McCouch et al., 2016
IRGC 1210	KOYRA::IR	Banglades	IRGC 7726	GCP, 2005; McCouch et al., 2016
IRGC 1210	KURULU V	Sri Lanka	IRGC 6651	GCP, 2005; McCouch et al., 2016
IRGC 1210	LAKHI PUF	Banglades	IRGC 3471	GCP, 2005; McCouch et al., 2016
IRGC 1210	LAKHSNIK	Banglades	IRGC 2588	GCP, 2005; McCouch et al., 2016
IRGC 1210	LALSAITA:	Banglades	IRGC 4391	GCP, 2005; McCouch et al., 2016
IRGC 1210	LENJA MU	Banglades	IRGC 6681	GCP, 2005; McCouch et al., 2016
IRGC 1210	MADHABS	Banglades	IRGC 2937	GCP, 2005; McCouch et al., 2016
IRGC 1210	MAHLAR 3	Banglades	IRGC 6427	GCP, 2005; McCouch et al., 2016
IRGC 1210	MAHSURI	Malaysia	IRGC 5269	GCP, 2005
IRGC 1210	MI TIMBR	Banglades	IRGC 2757	GCP, 2005; McCouch et al., 2016
IRGC 1210	MICOCHU	Banglades	IRGC 2588	GCP, 2005; McCouch et al., 2016
IRGC 1210	MIKOTCH	Banglades	IRGC 2589	GCP, 2005; McCouch et al., 2016
IRGC 1210	MOSHIA E	Banglades	IRGC 6681	GCP, 2005; McCouch et al., 2016
IRGC 1210	MOSHUR:	Banglades	IRGC 6478	GCP, 2005; McCouch et al., 2016
IRGC 1210	MOYNA M	Banglades	IRGC 6681	GCP, 2005; McCouch et al., 2016
IRGC 1210	MUNSHI N	Banglades	IRGC 6681	GCP, 2005; McCouch et al., 2016
IRGC 1210	NCS 160::	India	IRGC 5190	GCP, 2005; McCouch et al., 2016

Do not distribute

IRGC 1210	NCS 183::	India	IRGC 5192	GCP, 2005; McCouch et al., 2016
IRGC 1210	NOROI::IR	Banglades	IRGC 3161	GCP, 2005; McCouch et al., 2016
IRGC 1210	NP 125::IF	India	IRGC 3255	GCP, 2005; McCouch et al., 2016
IRGC 1210	PANKHIRA	Banglades	IRGC 2591	GCP, 2005; McCouch et al., 2016
IRGC 1210	PHUDUGE	Bhutan	IRGC 3239	GCP, 2005; McCouch et al., 2016
IRGC 1210	RAKHOIL::	Banglades	IRGC 6479	GCP, 2005; McCouch et al., 2016
IRGC 1210	RANGPUR	Banglades	IRGC 6682	GCP, 2005; McCouch et al., 2016
IRGC 1211	SADA DAN	Banglades	IRGC 7729	GCP, 2005; McCouch et al., 2016
IRGC 1211	SADA SOL	Pakistan	IRGC 7311	GCP, 2005; McCouch et al., 2016
IRGC 1211	SAFED MA	India	IRGC 7477	GCP, 2005; McCouch et al., 2016
IRGC 1211	SAMPATT	Banglades	IRGC 2592	GCP, 2005; McCouch et al., 2016
IRGC 1211	SANTHI SU	Pakistan	IRGC 2821	GCP, 2005; McCouch et al., 2016
IRGC 1211	SATHI::IRC	Pakistan	IRGC 2822	GCP, 2005; related sample in McCouch et al., 2016
IRGC 1211	SEENETTI:	Sri Lanka	IRGC 4741	GCP, 2005; McCouch et al., 2016
IRGC 1211	SERETY::IF	Banglades	IRGC 3473	GCP, 2005; McCouch et al., 2016
IRGC 1211	SHADA SH	Banglades	IRGC 6479	GCP, 2005; McCouch et al., 2016
IRGC 1211	SHIRKATI:	Afghanista	IRGC 1452	GCP, 2005; McCouch et al., 2016
IRGC 1211	SIMUL KH	India	IRGC 3515	GCP, 2005; McCouch et al., 2016
IRGC 1211	SLO 16::IR	India	IRGC 63	GCP, 2005; McCouch et al., 2016
IRGC 1211	SOLOI::IRC	Banglades	IRGC 3759	GCP, 2005; McCouch et al., 2016
IRGC 1211	SREERAM	Banglades	IRGC 6479	GCP, 2005; McCouch et al., 2016
IRGC 1211	SUFAID 24	Pakistan	IRGC 2830	GCP, 2005; McCouch et al., 2016
IRGC 1211	SULTANJA	Banglades	IRGC 2592	GCP, 2005; McCouch et al., 2016
IRGC 1211	T 1::IRGC	India	IRGC 6294	GCP, 2005; related sample in McCouch et al., 2016
IRGC 1211	T 26::IRGC	India	IRGC 4676	GCP, 2005; McCouch et al., 2016
IRGC 1211	TAK SUFA	Pakistan	IRGC 7312	GCP, 2005; McCouch et al., 2016
IRGC 1211	TAOTHAB	India	IRGC 1374	GCP, 2005; McCouch et al., 2016
IRGC 1211	UPRB 56::	India	IRGC 4422	GCP, 2005; McCouch et al., 2016
IRGC 1211	UPRH 184	India	IRGC 6163	GCP, 2005; McCouch et al., 2016
IRGC 1211	URYEE BO	India	IRGC 7471	GCP, 2005; McCouch et al., 2016
IRGC 1211	WEDA HEI	Sri Lanka	IRGC 6766	GCP, 2005; McCouch et al., 2016
IRGC 1211	CA 902-B-	Chad	IRGC 6349	related sample in McCouch et al., 2016
IRGC 1211	PANNITTI:	Sri Lanka	IRGC 6652	GCP, 2005; McCouch et al., 2016
IRGC 1211	SATHRA 2	Pakistan	IRGC 2823	GCP, 2005; McCouch et al., 2016
IRGC 1211	SAYARI::IF	India	IRGC 7471	GCP, 2005; McCouch et al., 2016
IRGC 1211	ARC 6578	India	IRGC 1228	related sample in McCouch et al., 2016
IRGC 1211	DHALA SH	India	IRGC 3686	related sample in McCouch et al., 2016
IRGC 1211	KAUKAU::	Mali	IRGC 9007	related sample in McCouch et al., 2016
IRGC 1211	PAUNGM/	Myanmar	IRGC 6252	related sample in McCouch et al., 2016
IRGC 1211	SURJA MU	India	IRGC 3516	GCP, 2005; McCouch et al., 2016
IRGC 1211	ARC 1010	India	IRGC 2070	GCP, 2005; McCouch et al., 2016
IRGC 1211	ARC 1155	India	IRGC 2147	GCP, 2005; McCouch et al., 2016
IRGC 1211	ARC 1177	India	IRGC 2163	GCP, 2005; McCouch et al., 2016
IRGC 1211	ARC 1195	India	IRGC 2178	GCP, 2005; McCouch et al., 2016

Do not distribute

IRGC 1211	ARC 7099	India	IRGC 2049	GCP, 2005; McCouch et al., 2016
IRGC 1211	DAL KASH	Banglades	IRGC 2584	GCP, 2005; McCouch et al., 2016
IRGC 1211	DUMAI::IF	Banglades	IRGC 2585	GCP, 2005; McCouch et al., 2016
IRGC 1212	IC 27525::	India	IRGC 5398	GCP, 2005; McCouch et al., 2016
IRGC 1212	JHUM SOI	Banglades	IRGC 2587	GCP, 2005; McCouch et al., 2016
IRGC 1212	KALA SAN	India	IRGC 3495	GCP, 2005; McCouch et al., 2016
IRGC 1212	PANNITI::	Sri Lanka	IRGC 6652	GCP, 2005; McCouch et al., 2016
IRGC 1212	SOKANA::	India	IRGC 5276	GCP, 2005; McCouch et al., 2016
IRGC 1212	TAK SIAH:	Pakistan	IRGC 7312	GCP, 2005; McCouch et al., 2016
IRGC 1212	ARC 1035	India	IRGC 1244	GCP, 2005; McCouch et al., 2016
IRGC 1212	DHOLI BO	Banglades	IRGC 2751	GCP, 2005; McCouch et al., 2016
IRGC 1212	JAGLI BOF	Banglades	IRGC 2751	GCP, 2005; McCouch et al., 2016
IRGC 1212	JC 148::IR	India	IRGC 9069	GCP, 2005; McCouch et al., 2016
IRGC 1212	KHAO DA	Thailand	IRGC 2774	GCP, 2005; McCouch et al., 2016
IRGC 1212	KAMOZ::I	Pakistan	IRGC 6701	GCP, 2005; McCouch et al., 2016
IRGC 1212	ANDIKULA	Sri Lanka	IRGC 7738	GCP, 2005; McCouch et al., 2016
IRGC 1212	ASAHI::IR	Japan	IRGC 3398	GCP, 2005; McCouch et al., 2016
IRGC 1212	AUS 342::	Banglades	IRGC 2912	GCP, 2005; McCouch et al., 2016
IRGC 1212	AUS JHAR	Banglades	IRGC 2583	GCP, 2005; McCouch et al., 2016
IRGC 1212	BAGETAU	Nepal	IRGC 6190	GCP, 2005; McCouch et al., 2016
IRGC 1212	BALA (CR	India	IRGC 1288	GCP, 2005; McCouch et al., 2016
IRGC 1212	BROWN G	India	IRGC 4523	GCP, 2005; McCouch et al., 2016
IRGC 1213	DEHULA::	India	IRGC 7473	GCP, 2005; McCouch et al., 2016
IRGC 1213	ETYM::IRG	Soviet Uni	IRGC 3614	GCP, 2005; McCouch et al., 2016
IRGC 1213	JUMA::IR	Banglades	IRGC 6680	GCP, 2005; McCouch et al., 2016
IRGC 1213	KARUTHA	Sri Lanka	IRGC 4738	GCP, 2005; McCouch et al., 2016
IRGC 1213	KHADASIY	India	IRGC 5407	GCP, 2005; McCouch et al., 2016
IRGC 1214	MAHA PA	Sri Lanka	IRGC 5102	GCP, 2005; McCouch et al., 2016
IRGC 1214	MUTTU S	Sri Lanka	IRGC 3633	GCP, 2005; McCouch et al., 2016
IRGC 1214	P 660::IRG	Pakistan	IRGC 2813	GCP, 2005; McCouch et al., 2016
IRGC 1214	PODI HEE	Sri Lanka	IRGC 3634	GCP, 2005; McCouch et al., 2016
IRGC 1214	SAREINA::	India	IRGC 6775	GCP, 2005; McCouch et al., 2016
IRGC 1215	ARC 1408	India	IRGC 4139	GCP, 2005; McCouch et al., 2016
IRGC 1215	BHADOIA	Banglades	IRGC 6551	GCP, 2005; McCouch et al., 2016
IRGC 1215	TAK::IRGC	Pakistan	IRGC 7312	GCP, 2005; McCouch et al., 2016
IRGC 1215	JABARSHA	Banglades	IRGC 2586	GCP, 2005; McCouch et al., 2016
IRGC 1215	PTB 30::IR	India	IRGC 6304	GCP, 2005; McCouch et al., 2016
IRGC 1215	ARC 1017	India	IRGC 1238	GCP, 2005; McCouch et al., 2016
IRGC 1215	AUS 71::IF	Banglades	IRGC 2893	GCP, 2005; McCouch et al., 2016
IRGC 1216	CHANDAR	Banglades	IRGC 2584	GCP, 2005; McCouch et al., 2016
IRGC 1216	CHAWAL::	Pakistan	IRGC 7309	GCP, 2005; McCouch et al., 2016
IRGC 1216	JABOR SA	Banglades	IRGC 6683	GCP, 2005; McCouch et al., 2016
IRGC 1216	MEHR::IR	Iran	IRGC 1288	GCP, 2005; McCouch et al., 2016
IRGC 1218	H 4::IRGC	Sri Lanka	IRGC 8961	GCP, 2005; McCouch et al., 2016

Do not distribute

IRGC 1244	DM 56::IR	Banglades	IRGC 8787	related sample in McCouch et al., 2016
IRGC 1244	BRE JER::I	Thailand	IRGC 7823	GCP, 2005; McCouch et al., 2016
IRGC 1256	BEGUM::I	India	IRGC 3399	GCP, 2005
IRGC 1256	JHONA 34	India	IRGC 3742	GCP, 2005; related sample in McCouch et al., 2016
IRGC 1256	THAVALU	Sri Lanka	IRGC 1532	GCP, 2005; related sample in McCouch et al., 2016
IRGC 1259	KALUBAL/	Sri Lanka	IRGC 7702	GCP, 2005; related sample in McCouch et al., 2016
IRGC 1260	SINTANE I	Burkina Fa	IRGC 5418	GCP, 2005; related sample in McCouch et al., 2016
IRGC 1260	BASMATI	Pakistan	IRGC 2779	GCP, 2005
IRGC 1261	BAGHLAN	Afghanista	IRGC 5826	GCP, 2005; related sample in McCouch et al., 2016
IRGC 1261	ARC 7229	India	IRGC 1233	GCP, 2005
IRGC 1262	DV 85::IR(	Banglades	IRGC 8839	GCP, 2005; related sample in McCouch et al., 2016
IRGC 1262	NCS 840::	India	IRGC 6253	GCP, 2005
IRGC 1262	TEPAKAIN	Banglades	IRGC 2592	GCP, 2005
IRGC 1263	MOLLADI(	Banglades	IRGC 2590	GCP, 2005
IRGC 1269	HARLAN N	Nigeria	IRGC 1473	GCP, 2005
IRGC 1270	DHAN::IR(	India	IRGC 3681	GCP, 2005; McCouch et al., 2016
IRGC 1285	NS 1254::I	Madagasc	IRGC 6892	GCP, 2005
IRGC 1285	LEUANG P	Thailand	IRGC 2776	GCP, 2005; related sample in McCouch et al., 2016
IRGC 1319	SOLAY GH	Pakistan	IRGC 7312	GCP, 2005; McCouch et al., 2016
IRGC 1319	DZ 193::IR	Banglades	IRGC 8517	GCP, 2005; McCouch et al., 2016
IRGC 1357	MEKRA BH	India	IRGC 6101	GCP, 2005
IRGC 1378	KURKARU	Sri Lanka	IRGC 1544	GCP, 2005
IRGC 1379	PULUTHI I	India	IRGC 7753	GCP, 2005
Not assigr	P 737::IRG	Pakistan	IRGC 2817	GCP, 2005; related sample in McCouch et al., 2016

Do not distribute

Supp. Table S2. Genotypes included in the tropical japonica greenhouse experiment

ATPd	germplasm	Country	Seed source	Specified in	IRGC Gen	Designation
250	62667	SENEGAL	CRBT_174	Courtois et	121949	62667::C1
251	63-104	SENEGAL	IRGC_151	Courtois et	121700	63-104::IRGC 15100-C1
1	APO	PHILIPPIN	Wk Col		121705	APO::C1
252	ARAGUAI	BRAZIL	INGER_17	Courtois et	121706	ARAGUAIA::IRTP 17399-C1
253	ARIAS	INDONESI	IRGC_433	Courtois et	126994	ARIAS::IRGC 43325-C1
254	ARROZ CE	VENEZUEI	IRGC_656	Courtois et	121709	ARROZ CEBADA::IRGC 65646-C1
2	ASD 1	INDIA	IRGC_6267		121969	ASD 1::IRGC 6267-C1
4	AZUCENA	PHILIPPIN	IRGC_328	Courtois et	121973	AZUCENA::IRGC 328-C1
255	BABER	INDIA	IRGC_339	Courtois et	121975	BABER::IRGC 33984-C1
256	BAGAN AS	MALAYSIA	IRGC_715	Courtois et	121976	BAGANAN ASALAO::IRGC 71503-C1
257	BAKUNG H	MALAYSIA	IRGC_602	Courtois et	121977	BAKUNG H::IRGC 60220-C1
6	BASMATI :	INDIA	IRGC_4895		121979	BASMATI 370::IRGC 4895-C1
258	BELOHAKI	MADAGAS	CRBT_8342		121714	BELOHAKILA 119::GERVEX 8342-C1
259	BENGALY	MADAGAS	CRBT_475	Courtois et	121715	BENGALY VAKARINA::GERVEX 4750-C1
389	BESHI	TAIWAN	IRGC_826	Courtois et al.,2014		
260	BICO BRA	BRAZIL	IRGC_389	Courtois et	121982	BICO BRANCO::IRGC 38994-C1
261	BINULAW	PHILIPPIN	IRGC_268	Courtois et	121983	BINULAWAN::IRGC 26872-C1
262	BODA 148	MADAGAS	GRBT_8258		121985	BODA 148-3::GERVEX 8258-C1
263	BOTRA FC	MADAGAS	GRBT_8320		121989	BOTRA FOTSY::GERVEX 8320-C1
7	BULU PAN	INDONESI	IRGC_136	Courtois et	124382	BULU PANDAK::IRGC 13614-C1
282	CAN 7\BO\	COLOMBI	CRB_1000	Courtois et	122018	CNA 7\BO\1\1>33-13-6-1::C1
267	CANA RO)	BRAZIL	IRGC_259	Courtois et	121997	CANA ROXA::IRGC 25966-C1
265	CAAWA/F(	PHILIPPIN	IRGC_544	Courtois et	121993	CAAWA/FORTUNA 6::IRGC 5441-C1
266	CAIAPO	BRAZIL	CRB_1000	Courtois et	121994	CAIAPO::C1
268	CANELA D	BRAZIL	IRGC_504	Courtois et	135520	CANELA DE FERRO::IRGC 50448-C1
269	CHA LOY (	THAILAND	CRB_1000	Courtois et	122004	CHA LOY OE::C1
270	CHA PHU	THAILAND	CRB_1000	Courtois et	121724	CHA PHU MA::C1
273	CICIH BET	INDONESI	IRGC_433	Courtois et	122012	CICIH BETON::IRGC 43372-C1
276	CIRAD 392	MADAGAS	CRBT_904	Courtois et	121727	CIRAD 392::C1
277	CIRAD 394	MADAGAS	CRBT_904	Courtois et	121728	CIRAD 394::C1
278	CIRAD 402	BRAZIL	CRBT_903	Courtois et	121729	CIRAD 402::C1
279	CIRAD 403	Unknown	CRBT_903	Courtois et	122014	CIRAD 403::GERVEX 9039-C1
280	CIRAD 409	Unknown	CRBT_904	Courtois et	122015	CIRAD 409::C1
281	CIRAD 488	MADAGAS	CRBT_917	Courtois et	121730	CIRAD 488::GERVEX 9172-C1
283	COLOMBI	COLOMBI	IRTP_394	Courtois et	122020	COLOMBIA 1::IRTP 394-C1
284	CT13582-1	COLOMBI	CRB_1000	Courtois et	122021	CT 13582-15-5-M::C1
289	CUBA 65	CUBA	IRGC_106	Courtois et	122026	CUBA 65::IRGC 10658-C1
290	CUIABAN	BRAZIL	IRTP_174	Courtois et	122027	CUIABANA::IRTP 17401-C1
291	CURINCA	BRAZIL	CRB_1000	Courtois et	121696	CURINCA::C1
292	DAM	THAILAND	IRGC_237	Courtois et	122028	DAM::IRGC 23710-C1
294	DAVAO	PHILIPPIN	IRGC_824	Courtois et	122031	DAVAO::IRGC 8244-C1
296	DINORAD	PHILIPPIN	IRTP_125	Courtois et al.,2014		
8	DOM SOFI	IRAN	IRGC_12880		122036	DOM SOFID::IRGC 12880-C1
297	DOURADC	BRAZIL	IRGC_329	Courtois et	122038	DOURADO AGULHA::IRGC 3297-C1
9	DULAR	INDIA	IRGC_32561		122040	DULAR::IRGC 32561-C1
999	EARLY MU	BRAZIL	CRBT_850	Courtois et	121735	EARLY MUTANT IAC 165::GERVEX 8508-C1
300	ESPERAN	BOLIVIA	CRB_1000	Courtois et	121736	ESPERANZA::C1
10	FANDRAP	MADAGAS	IRGC_10984		122047	FANDRAPOTSY 104::IRGC 10984-C1
302	FOSSA H\	BURKI FA	IRGC_160	Courtois et	137954	FOSSA HV::IRGC 16069-C1
303	GANIGI	INDONESI	IRGC_486	Courtois et	122052	GANIGI::IRGC 48698-C1
304	GEMJYA J	BHUTAN	IRGC_324	Courtois et	121739	GEMJYA JYANAM::IRGC 32411-C1
13	GIZA 171	EGYPT	IRGC_507	Courtois et	122058	GIZA 171::IRGC 50750-C1
305	GOGO	INDONESI	IRGC_433	Courtois et	122063	GOGO::IRGC 43390-C1

307	GOGO LEI	INDONESI	IRGC_433	Courtois et	121741	GOGO LEMPAK::IRGC 43392-C1
306	GOGO LEI	INDONESI	IRGC_433	Courtois et	122064	GOGO LEMPUK::IRGC 43394-C1
308	GOMPA 2	INDIA	IRGC_128	Courtois et	122066	GOMPA 2::IRGC 12894-C1
310	GRAZI	IVORY CO	IRGC_508	Courtois et	122072	GRAZI::IRGC 50836-C1
311	GUARANI	BRAZIL	CRBT_850	Courtois et	121742	GUARANI::GERVEX 8506-C1
313	HAIFUGO	TAIWAN	IRGC_17054			
315	HD 1-4	FRANCE	CRBT_882	Courtois et	121744	HD 1-4::C1
14	IAC 165	BRAZIL	CRBT_850	Courtois et	122080	IAC 165::GERVEX 8508-C1
320	IAC 47	BRAZIL	CRBT_113	Courtois et	122084	IAC 47::C1
321	IDSA 77	IVORY CO	CRBT_870	Courtois et	121746	IDSA 77::C1
322	IGUAPE C.	BRAZIL	IRGC_412	Courtois et	122086	IGUAPE CATETO::IRGC 4122-C1
323	INDANE	MYANMAR	IRGC_331	Courtois et	122087	INDANE::IRGC 33130-C1
458	IR47684-0	PHILIPPIN	CRB_1000	Courtois et	121749	IR 47684-05-1-B::C1
446	IR47686-0	PHILIPPIN	CRB_1000	Courtois et	122095	IR 47686-09-01-B-1::C1
447	IR53236-2	PHILIPPIN	CRB_1001	Courtois et	122096	IR 53236-275-1::C1
325	IR60080-4	PHILIPPIN	IRTP_2101	Courtois et	122100	IR 60080-46 A::IRTP 21016-C1
326	IR63371-3	PHILIPPIN	Wk col		121754	IR 63371-38::C1
328	IR63372-0	PHILIPPIN	CRB_1001	Courtois et	121755	IR 63372-8::C1
327	IR63380-0	PHILIPPIN	CRB_1001	Courtois et	122101	IR 63380-16::C1
15	IR64	PHILIPPIN	IRGC_669	Courtois et	122102	IR 64::IRGC 66970-C1
449	IR65261-1	PHILIPPIN	CRB_1001	Courtois et	122103	IR 65261-19-1-B::C1
330	IR65907-1	PHILIPPIN	Wk col		121698	IR 65907-116-1-B::C1
451	IR65907-1	PHILIPPIN	Wk col		122104	IR 65907-173-1-B::C1
331	IR65907-1	PHILIPPIN	IRTP_2107	Courtois et	122105	IR 65907-188-1-B::IRTP 21075-C1
452	IR65907-2	PHILIPPIN	CRB_1001	Courtois et	122106	IR 65907-206-4-B::C1
332	IR66421-0	PHILIPPIN	CRB_1001	Courtois et	121757	IR 66421-096-2-1-1::C1
453	IR66421-1	PHILIPPIN	CRB_1001	Courtois et	122107	IR 66421-105-1-1::C1
334	IR68704-1	PHILIPPIN	CRB_1001	Courtois et	122108	IR 68704-145-1-1-B::C1
335	IR70758-1	PHILIPPIN	Wk col		121758	IR 70758-17-2-1::C1
454	IR71524-4	PHILIPPIN	CRB_1001	Courtois et	122109	IR 71524-44-1-1::C1
336	IR71525-1	PHILIPPIN	CRB_1001	Courtois et	122110	IR 71525-19-1-1::C1
337	IR71676-9	PHILIPPIN	Wk col (=NPT)		121759	IR 71676-90-2-2::C1
338	IR72967-1	PHILIPPIN	Wk col (=NPT)		122111	IR 72967-12-2-3::C1
340	IRAT 109	IVORY CO	CRBT_498	Courtois et	121761	IRAT 109::GERVEX 4988-C1
341	IRAT 112	IVORY CO	CRBT_499	Courtois et	122115	IRAT 112::C1
343	IRAT 144	GHA	IRGC_556	Courtois et	121762	IRAT 144::IRGC 55685-C1
344	IRAT 170	IVORY CO	CRBT_743	Courtois et	122117	IRAT 170::C1
345	IRAT 177	FRENCH (	CRBT_741	Courtois et	122118	IRAT 177::C1
347	IRAT 212	IVORY CO	CRBT_769	Courtois et	121763	IRAT 212::GERVEX 7698-C1
348	IRAT 216	IVORY CO	CRBT_770	Courtois et	121764	IRAT 216::GERVEX 7702-C1
349	IRAT 234	GUF-BRA	CRBT_740	Courtois et	122119	IRAT 234::C1
350	IRAT 257	BRAZIL	CRBT_775	Courtois et	121765	IRAT 257::C1
352	IRAT 362	NICARAGU	CRBT_871	Courtois et	121767	IRAT 362::GERVEX 8712-C1
353	IRAT 364	NICARAGU	CRBT_871	Courtois et	121768	IRAT 364::GERVEX 8714-C1
354	IRAT 366	NICARAGU	CRBT_871	Courtois et	121769	IRAT 366::GERVEX 8716-C1
355	IRAT 380	MADAGAS	CRBT_879	Courtois et	121770	IRAT 380::C1
356	JAO HAW	THAILAND	CRB_1002	Courtois et	122123	JAO HAW::C1
357	JIMBRUK .	INDONESI	IRGC_434	Courtois et	121772	JIMBRUK JOLOWORO::IRGC 43420-C1
358	JUMALI	NEPAL	IRGC_954	Courtois et	121773	JUMALI::IRGC 9542-C1
360	KAKANI 2	NEPAL	IRGC_133	Courtois et	122128	KAKANI 2::IRGC 13373-C1
361	KANIRAN(	INDONESI	IRGC_914	Courtois et	122129	KANIRANGA::IRGC 9145-C1
362	KARASUK.	TAIWAN	IRGC_819	Courtois et	122130	KARASUKARA SURANKASU::IRGC 8196-C
363	KEDAYAN	MALAYSIA	IRGC_715	Courtois et	122133	KEDAYAN::IRGC 71537-C1
365	KENDINGA	MALAYSIA	IRGC_603	Courtois et	122134	KENDINGA 5 H::IRGC 60310-C1
366	KERITING	Unknown	IRGC_19972		124402	KERITING TINGGI::IRGC 19972-C1

Do not distribute

368	KETAN LU	INDONESI	IRGC_179	Courtois et	121776	KETAN LUMBU::IRGC 17906-C1
370	KETAN SE	INDONESI	IRGC_43448		132380	KETAN SELEM::IRGC 43448-C1
17	KHAO DAI	THAILAND	IRGC_233	Courtois et	124403	KHAO DAM::IRGC 23385-C1
371	KHAO KAF	LAOS	IRGC_234	Courtois et	122137	KHAO KAP XANG::IRGC 23423-C1
372	KINDANG	PHILIPPIN	IRGC_233	Courtois et	122139	KINANDANG PATONG::IRGC 23364-C1
373	KOMOJAM	MADAGAS	CRBT_845	Courtois et	121779	KOMOJAMANITRA::GERVEX 8453-C1
374	KU 115	THAILAND	CRBT_340	Courtois et	122141	KU 115::GERVEX 3401-C1
375	KUROKA	JAPAN	IRGC_745	Courtois et	121781	KUROKA::IRGC 74556-C1
376	LAC 23	LIBERIA	IRGC_14957		122145	LAC 23::IRGC 14957-C1
377	LAMBAYQ	PERU	IRGC_107	Courtois et	122148	LAMBAYQUE 1::IRGC 10769-C1
378	LOHAMBIT	MADAGAS	CRBT_8308		121786	LOHAMBITRO 3670::GERVEX 8238-C1
379	LUDAN	PHILIPPIN	IRGC_641	Courtois et	121787	LUDAN::IRGC 64189-C1
19	M 202	USA	IRGC_771	Courtois et	122155	M 202::IRGC 77142-C1
380	MA HAE	THAILAND	IRGC_237	Courtois et	122157	MA HAE::IRGC 23754-C1
381	MAINTIMC	MADAGAS	IRGC_110	Courtois et	121791	MAINTIMOLOTSY 1226::IRGC 11010-C1
382	MALAGKIT	PHILIPPIN	IRGC_818	Courtois et	122158	MALAGKIT PIRURUTONG::IRGC 8182-C1
383	MANELATI	MADAGAS	CRBT_844	Courtois et	121793	MANANELATRA 520::GERVEX 8445-C1
385	MARAVILH	BRAZIL	CRB_1002	Courtois et	122161	MARAVILHA::C1
386	MED NOI	THAILAND	CRBT_2570		121795	MED NOI::GERVEX 2570-C1
387	MITSANG	MADAGAS	CRBT_831	Courtois et	122172	MITSANGANAHIJERY::GERVEX 8316-C1
388	MOLOK	INDONESI	IRGC_434	Courtois et	121796	MOLOK::IRGC 43485-C1
21	MOROBEF	GUINEA	IRGC_120	Courtois et	122173	MOROBEREKAN::IRGC 12048-C1
22	N 22	INDIA	IRGC_4819		122176	N 22::IRGC 4819-C1
390	NEP HOA	VIETM	IRGC_407	Courtois et	127003	NEP HOA VANG::IRGC 40748-C1
391	NHTA 10	INDIA	IRGC_191	Courtois et	121798	NHTA 10::IRGC 191-C1
392	NHTA 5	INDIA	IRGC_186	Courtois et	122305	NHTA 5::IRGC 186-C1
23	NIPPONB	JAPAN	IRGC_127	Courtois et	124410	NIPPONBARE::IRGC 12731-C1
393	NPE 253	PAKISTAN	IRGC_386	Courtois et	127004	NPE 826::IRGC 38694-C1
397	ORYZICA	COLOMBI	CRB_1002	Courtois et	121801	ORYZICA SABANA 6::C1
398	OS 4	ZAIRE	IRGC_113	Courtois et	122185	OS 4::IRGC 11335-C1
399	OS 6	ZAIRE	CRBT_6	Courtois et	122186	OS 6::GERVEX 6-C1
400	P5589-1-1	COLOMBI	CRB_1002	Courtois et	122188	P 5589-1-1-3-P::C1
401	PACHOLIN	BRAZIL	IRGC_505	Courtois et	122189	PACHOLINHA::IRGC 50531-C1
403	PADI BOE	INDONESI	IRGC_914	Courtois et	121802	PADI BOENAR::IRGC 9147-C1
404	PADI KAS	INDONESI	IRGC_826	Courtois et	122190	PADI KASALLE::IRGC 8261-C1
406	PADI ROT	INDONESI	IRGC_110452		124413	PADI ROTAN::C1
407	PALAWAN	PHILIPPIN	CRB_1002	Courtois et	121803	PALAWAN::C1
409	PATE BLA	IVORY CO	IRGC_160	Courtois et	122192	PATE BLANC MAN 1::IRGC 16073-C1
410	PCT11\0\0	COLOMBI	CRB_1002	Courtois et	121804	PCT 11\0\0\2,BO 1>55-1-3-1::C1
412	PCT4\SA\4	COLOMBI	CRB_1002	Courtois et	121805	PCT 4\SA\4\1>1076-2-4-1-5::C1
413	PEH PI NU	CHI	IRGC_826	Courtois et	121807	PEH PI NUO::IRGC 8266-C1
414	POENOET	INDONESI	IRGC_819	Courtois et	124415	POENOET HITAM::IRGC 8193-C1
459	PRIMAVEF	BRAZIL	CRB_1002	Courtois et	121809	PRIMAVERA::C1
417	RATHAL	SRI LANK	IRGC_315	Courtois et	122212	RATHAL::IRGC 31524-C1
418	REKET M	INDONESI	IRGC_435	Courtois et	122215	REKET MAUN::IRGC 43570-C1
419	RT 1031-6	ZAIRE	IRGC_150	Courtois et	122224	RT 1031-69::IRGC 15092-C1
421	SEBOTA 6	BRAZIL	Wk col (=CIRAD 402)		122242	SEBOTA 65::C1
422	SENG	THAILAND	IRGC_237	Courtois et	122245	SENG::IRGC 23776-C1
424	SPEAKER	PHILIPPIN	CRB_1003	Courtois et	121825	SPEAKER::C1
427	TANDUI	MALAYSIA	IRGC_716	Courtois et	122262	TANDUI::IRGC 71635-C1
25	TEQUING	CHINA	IRGC_81093		122263	TE QING::IRGC 81093-C1
428	TREMBES	INDONESI	IRGC_436	Courtois et	122269	TREMBESE::IRGC 43675-C1
429	TRES ME	BRAZIL	IRGC_646	Courtois et	122270	TRES MESES::IRGC 6464-C1
430	TSIPALA 8	MADAGAS	CRBT_838	Courtois et	121833	TSIPALA 89::GERVEX 8385-C1
432	VARY LAV	MADAGAS	CRBT_825	Courtois et	122275	VARY LAVA 90::GERVEX 8254-C1

434	VARY MAI	MADAGAS	CRBT_844	Courtois et	122277	VARY MADINIKA 3566::GERVEX 8448-C1
435	VARY MAL	MADAGAS	CRBT_832	Courtois et	122278	VARY MALADY::GERVEX 8325-C1
437	VARY SON	MADAGAS	CRBT_832	Courtois et	121838	VARY SOMOTRA SIHANAKA::GERVEX 832
455	VIETNAM	VIETM	CRB_1003	Courtois et	122281	VIETNAM 1::C1
456	VIETNAM	VIETM	Wk col		121841	VIETNAM 2::C1
457	VIETNAM	VIETM	CRB_1003	Courtois et	121842	VIETNAM 3::C1
438	WAB 56-12	IVORY CO	IRTP_1977	Courtois et	121843	WAB 56-125::IRTP 19771-C1
439	WAB 56-50	IVORY CO	IRTP_1946	Courtois et	122282	WAB 56-50::IRTP 19464-C1
440	WAB706-3	IVORY CO	CRB_1003	Courtois et	121844	WAB 706-3-4-K4-KB-1::C1
443	YANCAOU	IVORY CO	IRGC_160	Courtois et	121859	YANCAOUSSA::IRGC 16071-C1
444	YANGKUM	BHUTAN	IRGC_324	Courtois et	122299	YANGKUM RED::IRGC 32406-C1
445	YUNLU 7	CHINA	CRB_1003	Courtois et	121861	YUNLU 7::C1

Do not distribute

Do not distribute

Do not distribute

1

Do not distribute

1-C1

Do not distribute

Supplementary Files

Leaf morphology, rather than plant water status, underlies genetic variation of rice leaf rolling under drought

Supp. Table S3a. Leaf anatomical parameters measured in eight selected aus genotypes in field drought stress and well-watered treatments (mesophyll and small-vein parameters; MCNO = mesophyll cell number, MCWD = mesophyll cell width, MCTA = mesophyll cell total area, SVWD = small vein width, SVHT = small vein height, SV-LFTH = small vein leaf thickness, IVD-SV = interveinal distance small vein, SV-BSCN = small vein bundle sheath cell number, SV-BSCA = small vein bundle sheath cell area). Letter groups indicate significant differences among genotypes ($p<0.05$).

Do not distribute

genotype	MCNO	Total MCWD (µm)	Ave. MCWD (µm)	MCTA (µm²)	SVWD (µm)	SVHT (µm)	SV-LFTH (µm)	IVD-SV (µm)	SV-BSCN	SV-BSCA (µm²)
Drought stress										
ARC 14088	9.5	154 e	16.5	8011 c	45.7 b	46.4 cd	93	189 cd	10.6 c	77
Brown Gora	11.0	174 bcd	16.1	8579 bc	47.5 b	48.6 bcd	100	208 abcd	11.0 bc	85
Dangar	10.6	186 ab	17.7	14716 a	57.3 a	57.4 a	118	244 a	12.6 a	116
Goai	9.6	166 cde	17.6	8477 bc	47.2 b	47.7 cd	98	203 bcd	10.8 bc	82
IC27525	11.0	193 a	17.7	12118 ab	55.2 a	57.2 ab	109	234 ab	11.3 bc	110
Lakhsnikajal	10.6	170 bcde	16.2	8827 bc	46.7 b	44.3 d	103	181 d	11.6 b	82
Tak Siah	9.7	161 de	16.7	9631 bc	52.5 ab	52.3 abcd	108	204 bcd	11.5 b	109
UPRB56	10.2	182 abc	18.2	10645 abc	52.5 ab	53.6 abc	115	220 abc	11.3 bc	98
Well-watered										
ARC 14088	10.1	162 c	16.1	8818 c	46.9 bc	46.2 cd	100 bc	182 c	10.5 cd	71.4
Brown Gora	9.4	172 bc	18.9	7812 c	46.5 c	46.2 cd	92 c	198 bc	10.5 cd	70.7
Dangar	9.5	181 bc	19.6	9544 bc	52.6 ab	53.8 abc	98 bc	215 b	11.5 b	74.9
Goai	9.7	176 bc	18.8	8462 c	45.6 c	47.8 bcd	93 c	186 c	10.4 d	72.4
IC27525	11.0	206 a	19.2	14417 a	58.1 a	59.4 a	121 a	244 a	12.6 a	91.6
Lakhsnikajal	9.6	188 ab	20.3	11181 b	53.6 a	55.6 ab	109 ab	216 b	11.6 b	85.4
Tak Siah	10.4	169 bc	16.6	7946 c	42.8 c	43.3 d	90 c	197 bc	9.8 d	72.7
UPRB56	8.9	188 ab	21.5	11170 b	54.4 a	58.8 a	110 ab	216 b	11.3 bc	82.1

Supp. Table S3b. Leaf anatomical parameters measured in eight selected aus genotypes in field drought stress and well-watered treatments (large-vein parameters; LVHT = large vein height, LV-LFTH = large vein leaf thickness, LVWD = large vein width, LV-IVD = interveinal distance large vein, LV-BSCN = large vein bundle sheath cell number, LV-BSCA = large vein bundle sheath cell area. Letter groups indicate significant differences among genotypes ($p<0.05$).

Genotype	LVHT (µm)	LV-LFTH (µm)	LVWD (µm)	LV-IVD (µm)	LV-BSCN	LV-BSCA (µm^2)
Drought stress						
ARC 14088	111	204 b	128	213 c	19.0	172 cd
Brown Gora	114	214 b	135	233 bc	19.9	176 bcd
Dangar	124	233 ab	149	253 ab	20.1	224 abc
Goai	115	231 ab	139	235 bc	19.6	179 bcd
IC27525	131	255 a	153	270 a	20.2	227 ab
Lakhsnikajal	114	231 ab	138	235 bc	20.2	159 d
Tak Siah	115	215 b	137	226 c	21.0	166 d
UPRB56	120	251 a	144	259 ab	18.9	234 a
Well-watered						
ARC 14088	99 cd	196 cd	123 de	226	18.2 bc	143
Brown Gora	112 bcd	217 bc	135 cde	227	18.9 b	151
Dangar	119 abc	220 bc	139 abcd	233	18.8 bc	174
Goai	108 bcd	221 bc	136 bcde	220	18.2 bc	165
IC27525	129 ab	256 a	154 a	274	21.9 a	180
Lakhsnikajal	141 a	255 a	152 ab	248	20.4 ab	173
Tak Siah	94 d	189 d	121 e	228	16.6 c	151
UPRB56	117 bcd	235 ab	142 abc	236	19.0 d	164

Do not distribute

Supp. Table S4. Correlations between the change in normalized difference vegetation index (ΔNDVI) and leaf rolling score (LRS) in the 2010 and 2012 field experiments, based on Spearman's rank correlation.

Season	rho	P value	Measurements compared
2010	0.27	<0.001	ΔNDVI 68-74 DAS and LRS on 74 DAS
2012E	0.15	0.03	ΔNDVI 64-70 DAS and LRS on 73 DAS
2012M	0.2	<0.001	ΔNDVI 71-74 DAS and LRS on 74 DAS
2012M	0.33	<0.001	ΔNDVI 71-81 DAS and LRS on 74 DAS
2012L	0.21	0.03	ΔNDVI 85-88 DAS and LRS on 86 DAS

Do not distribute

Supp. Table S5. Relationships among canopy temperature (CT) and the change in normalized difference vegetation index (ΔNDVI) or and shoot biomass, based on ANOVA on a panel of 226 aus rice genotypes under drought conditions in 3 field studies during the dry season of 2010, 2011, 2012. In 2012, genotypes were grouped depending on their phenology: early (E), medium (M) and Late (L) and shoot biomass was only collected for 26 selected genotypes contrasting for leaf rolling.

Season	ΔNDVI	Shoot biomass	Measurements compared
	Relationship with CT (P-value)		
2010	0.5214	0.9539	ΔNDVI 68-74 DAS, shoot biomass on 78 DAS, and CT on 76 DAS
2011	0.7765	<0.001	ΔNDVI 83-89 DAS, shoot biomass on 86 DAS, and CT on 89 DAS
2012E	0.003	0.580	ΔNDVI 64-70 DAS, shoot biomass on 73 DAS, and CT on 70 DAS
2012M	0.993	0.328	ΔNDVI 71-74 DAS, shoot biomass on 81 DAS, and CT on 74 DAS
2012L	0.331	0.437	ΔNDVI 85-88 DAS, shoot biomass on 106 DAS, and CT on 86 DAS

Supplementary Table S6. Relationships among leaf rolling (leaf rolling score ΔNDVI) with maintenance of biomass and grain yield under drought in the aus field and greenhouse experiments, based on correlation (Spearman's for leaf rolling traits, Pearson for ΔNDVI). DS: drought stress treatment, WW: well-watered treatment.

	Maintenance of biomass ((DS - WW)/WW)		Maintenance of grain yield ((DS - WW)/WW)	
	LRS	ΔNDVI	LRS	ΔNDVI
**Field**				
2010	-0.15*	0.11	-0.19**	0.08
2011	n/a	0.11	n/a	0.11
2012 early	0.03	-0.10	-0.09	-0.012
2012 med	0.33***	0.11	-0.16	-0.13
2012 late	-0.17	0.35*	-0.26	0.03
**Greenhouse**				
BG02	0.1	n/a	n/a	n/a

Do not distribute

Supp. Table S7. Sclerenchyma cell area and number in six selected aus genotypes in the 2018DS field well-watered treatment. Letter groups indicate significant differences among genotypes ($p<0.05$).

	Adaxial		Abaxial	
	Total sclerenchyma cell area (μm^2)	Number of sclerenchyma cells	Total sclerenchyma cell area (μm^2)	Number of sclerenchyma cells
Brown Gora	298.4 a	12.0 a	315.3 a	14.50 a
Dangar	228.5 cd	8.50 b	240.6 b	9.64 b
Goai	239.5 bc	8.11 b	259.6 b	8.80 b
IC 27525	200.6 d	9.50 b	260.3 b	11.56 ab
Lakhsnikajal	266.5 ab	8.83 b	244.2 b	10.97 b
UPRB 56	219.1 cd	8.81 b	233.0 b	9.39 b

Do not distribute

Supp. Table S8. Bulliform cell size and number, as well as stomatal density, in eight selected aus genotypes in 2012WS field drought stress and well-watered treatments. Letter groups indicate significant differences among genotypes ($p<0.05$).

Treatment		Bulliform cell number	Width of all bulliform cells (μm)	Height of largest bulliform cell (μm)	Stomatal density (stomata/μm^2)
Drought stress	ARC 14088	7.02	92.9	45.3	0.00406
	Brown Gora	5.83	85.5	45.9	0.00482
	Dangar	6.41	97.9	50.5	0.00514
	Goai	6.80	90.2	44.9	0.00448
	IC27525	5.88	94.8	51.9	0.00532
	Lakhsnikajal	6.80	94.2	49.5	0.00534
	Tak Siah	6.25	84.7	47.9	0.00458
	UPRB56	6.78	97.7	48.6	0.00539
Well-watered					
	ARC 14088	5.50	78.4	43.7 d	
	Brown Gora	5.45	78.1	42.4 d	
	Dangar	5.31	83.2	44.8 cd	
	Goai	6.32	94.3	43.8 cd	
	IC27525	5.35	91.0	53.3 a	
	Lakhsnikajal	5.05	80.0	48.9 b	
	Tak Siah	5.55	82.0	44.2 cd	
	UPRB56	5.69	86.7	47.6 bc	

Do not distribute

Supp. Table S9. The most contrasting genotypes from the japonica panel in terms of leaf rolling score and maintenance of shoot biomass under drought as compared to that under well-watered conditions (SDWratio, calculated as (DS-WW/WW)).

**Genotype**	**Mean leaf rolling score**	**Genotype**	**SDWratio**
**Highest values**		**Most negative values**	
CAAWA/FORTU 6	7	KUROKA	-0.8090
CANELA DE FERRO	7	YUNLU 7	-0.7460
IAC 47	7	PADI KASALLE	-0.7290
IGUAPE CATETO	7	GRAZI	-0.6920
IRAT 212	7	VIETM2	-0.6820
IRAT 257	7	OS 6	-0.6780
VIETM2	7	TSIPALA 89	-0.6780
DAVAO	7.66667	CIRAD 409	-0.6690
GOGO	7.66667	IRAT 170	-0.6670
WAB706-3-4-K-KB-1	7.66667	PCT4\SA\4\1>1076-2-4-1-5	-0.6620
TRES MESES	8		
PADI KASALLE	9		
**Lowest values**		**Least negative values**	
GUARANI	0	DOURADO AGULHA	-0.2580
IR66421-096-2-1-1	0	PALAWAN	-0.2580
IRAT 112	0	SPEAKER	-0.2520
KARASUKARA SURANKASU	0	ESPERANZA	-0.2380
KETAN LUMBU	0	BABER	-0.2360
BESHI	0	RATHAL	-0.2310
WAB 56-125	0	BICO BRANCO	-0.2300
CIRAD 403	0.33333	BAKUNG H	-0.2280
NEP HOA VANG	0.33333	VIETM3	-0.2160
P5589-1-1-3-P	0.33333	ARROZ CEBADA	-0.1510
POENOET HITAM	0.33333	PCT11\0\0\2,BO\1>55-1-3-1	-0.1390
		IR68704-145-1-1-B	-0.0899
		VARY MALADY	0.0569

Do not distribute

Supp. Table S10. Traits for which association analysis was conducted on genotypes with available sequence data. Vg (additive genetic variance), Ve (residual phenotypic variance, environmental).

	aus							tropical japonica
Experiment	Lysimeter	Field 2010DS	Field 2012DS (Med)	Field 2012DS (Med)	Field 2010DS	Field 2011DS	Field 2012DS (Med)	Greenhouse
Measurement	LRS 84 DAS	LRS 74 DAS	LRS 74 DAS	LRS 106 DAS	ΔNDVI 68-74 DAS	ΔNDVI 83-89 DAS	ΔNDVI 74-81 DAS	LRS FTSW 0.2
Samples scanned	41	87	61	61	86	93	60	146
Markers analyzed	206611	210540	209205	209205	212267	212407	210911	16444
Pseudo-heritability	0.00	0.17	0.62	1.00	1.00	0.23	1.00	0.28
Variance of heritability	0.00	0.03	0.28	1.63	3080.53	2742.85	350692.00	7.71
Std Error of heritability	0.00	0.16	0.53	1.28	55.50	52.37	592.19	2.78
Vg	0.00	0.17	0.92	0.63	0.00	0.00	0.00	2.14
Ve	5.93	0.86	0.56	0.00	0.00	0.00	0.00	5.57

Do not distribute

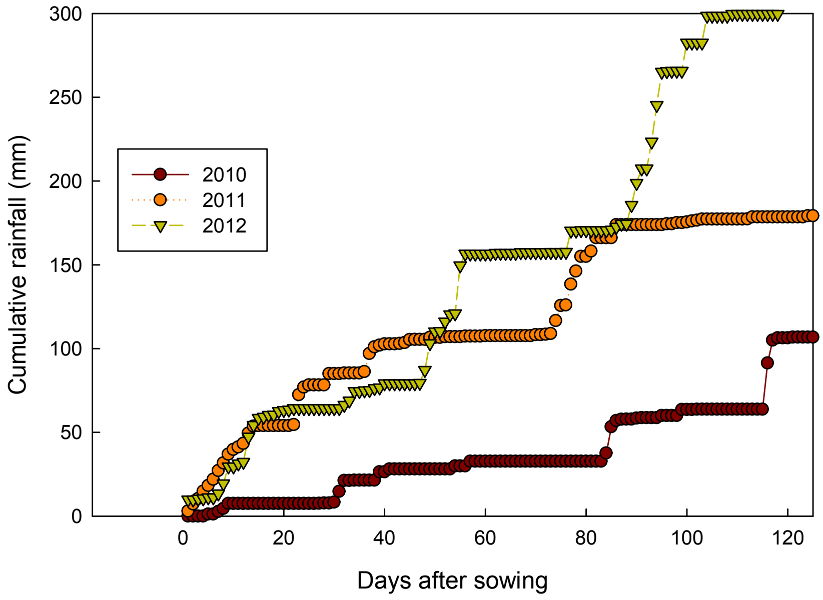

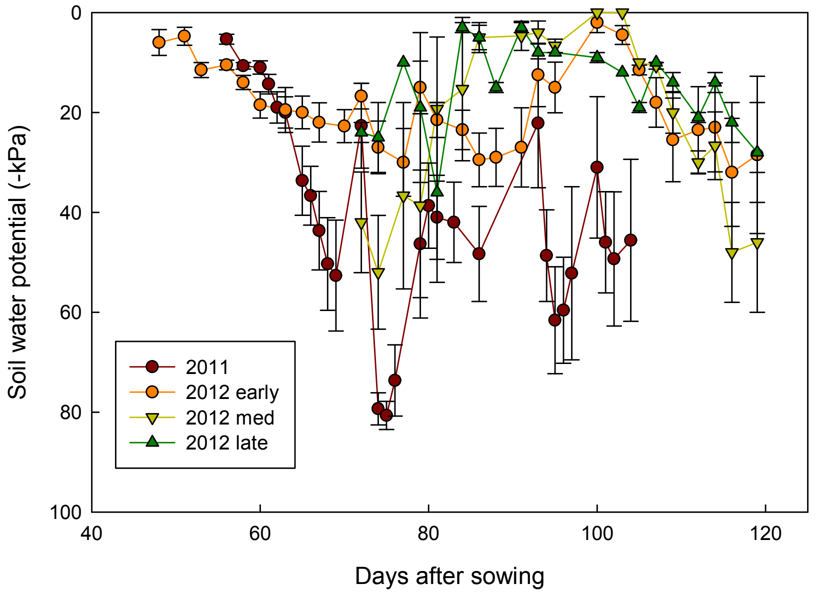

Supp Fig S1. Rainfall and soil water potential in the drought treatment (30 cm depth) from the drought treatments in the aus field studies.

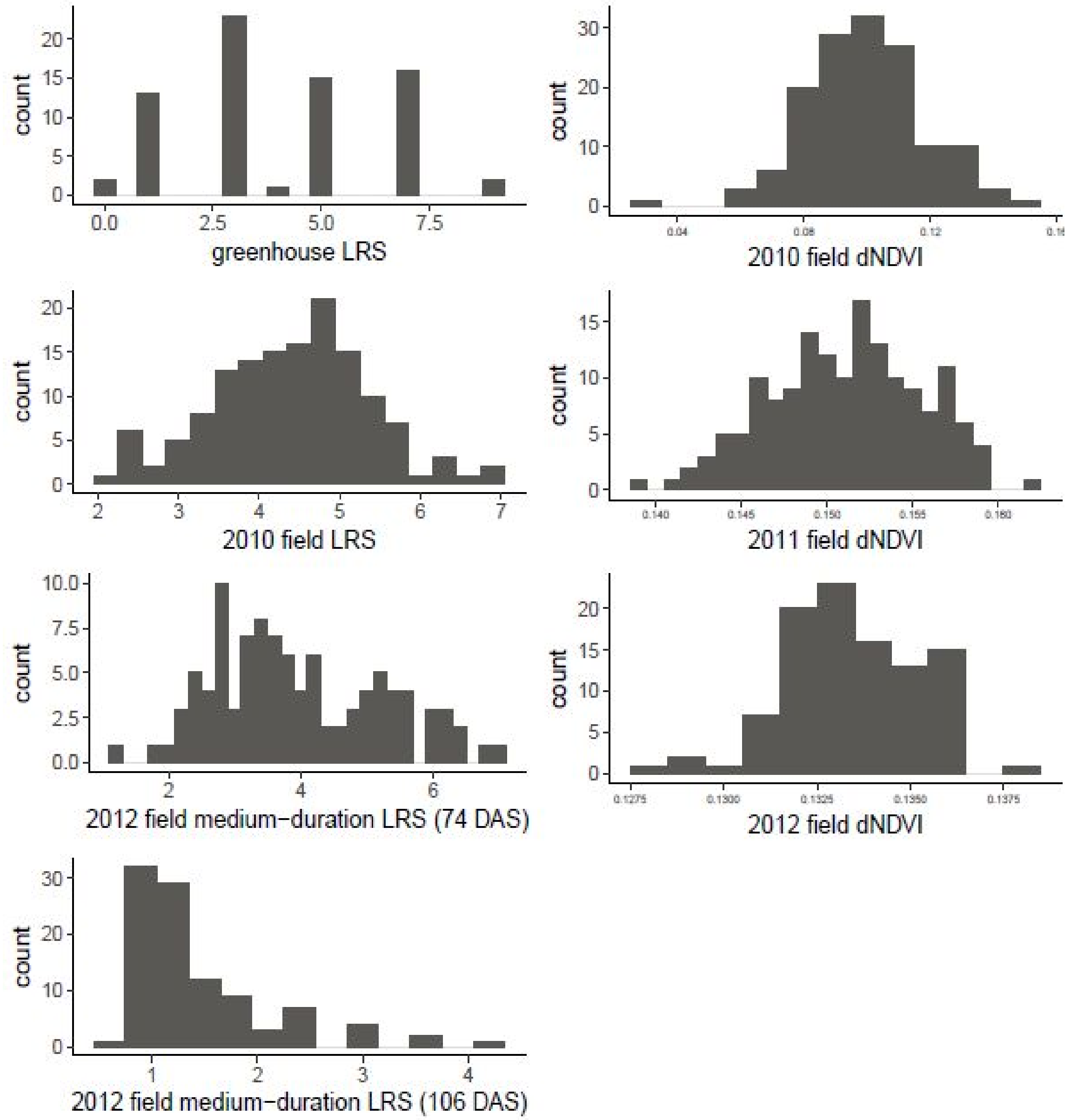

Supp. Fig. S2. Distributions of mean leaf rolling scores and ΔNDVI values of aus genotypes in the field and greenhouse lysimeter experiments. The ΔNDVI values shown are BLUPs fitted from a mixed model with replication as a random effect.

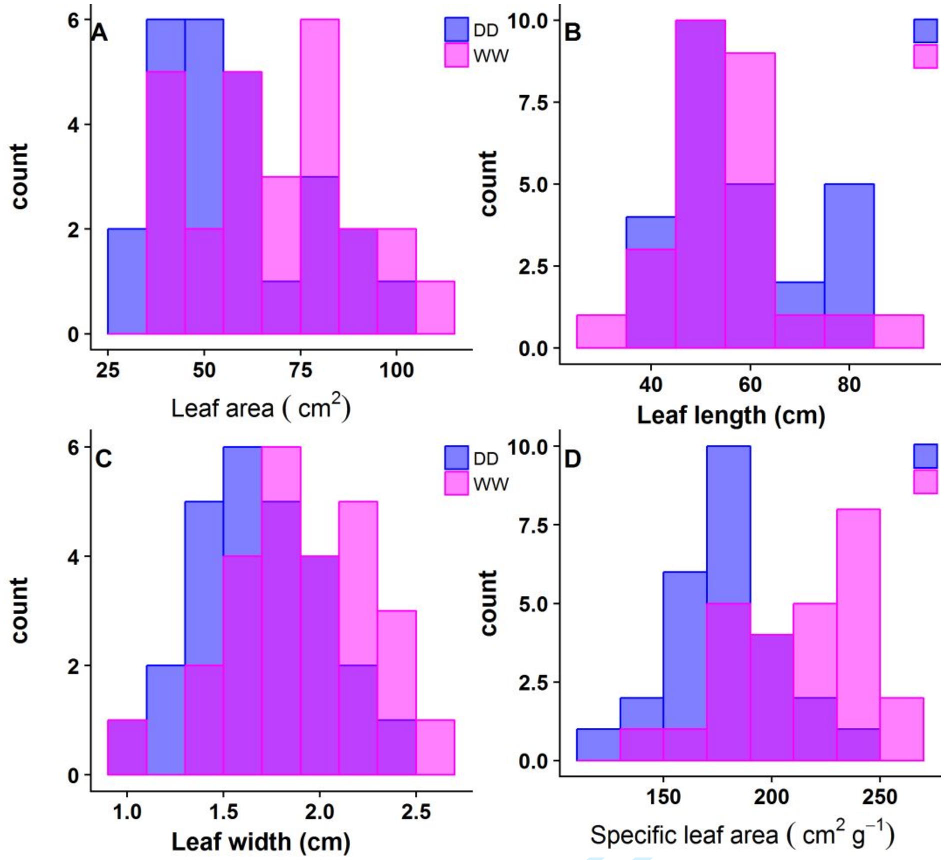

Supp. Fig. S3. Leaf morphology distributions among 26 selected genotypes in the aus panel grown in the greenhouse lysimeter study.The youngest fully expanded leaf at 71 DAS was measured to determine A) leaf area, B) leaf length, C) leaf width, and D) specific leaf area.

istribute

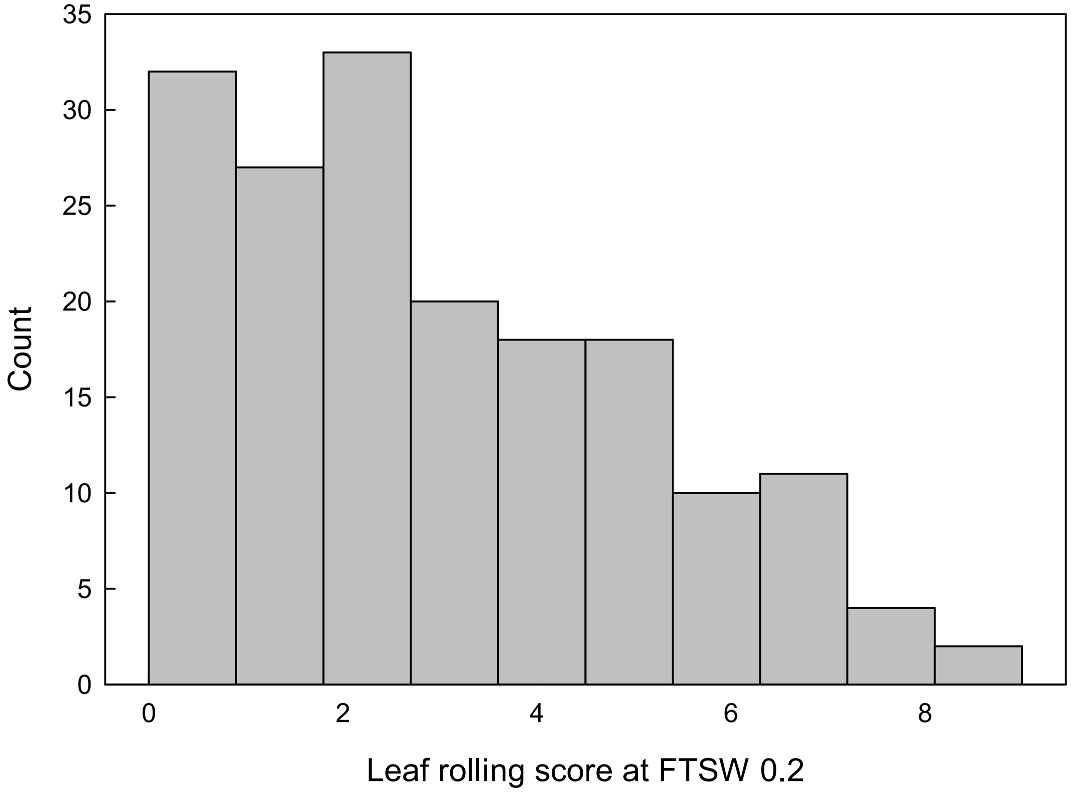

Supp. Fig. S4. Distribution of mean leaf rolling score of 172 tropical japonica genotypes at the end of the greenhouse study, when the soil moisture level reached FTSW of 0.2.

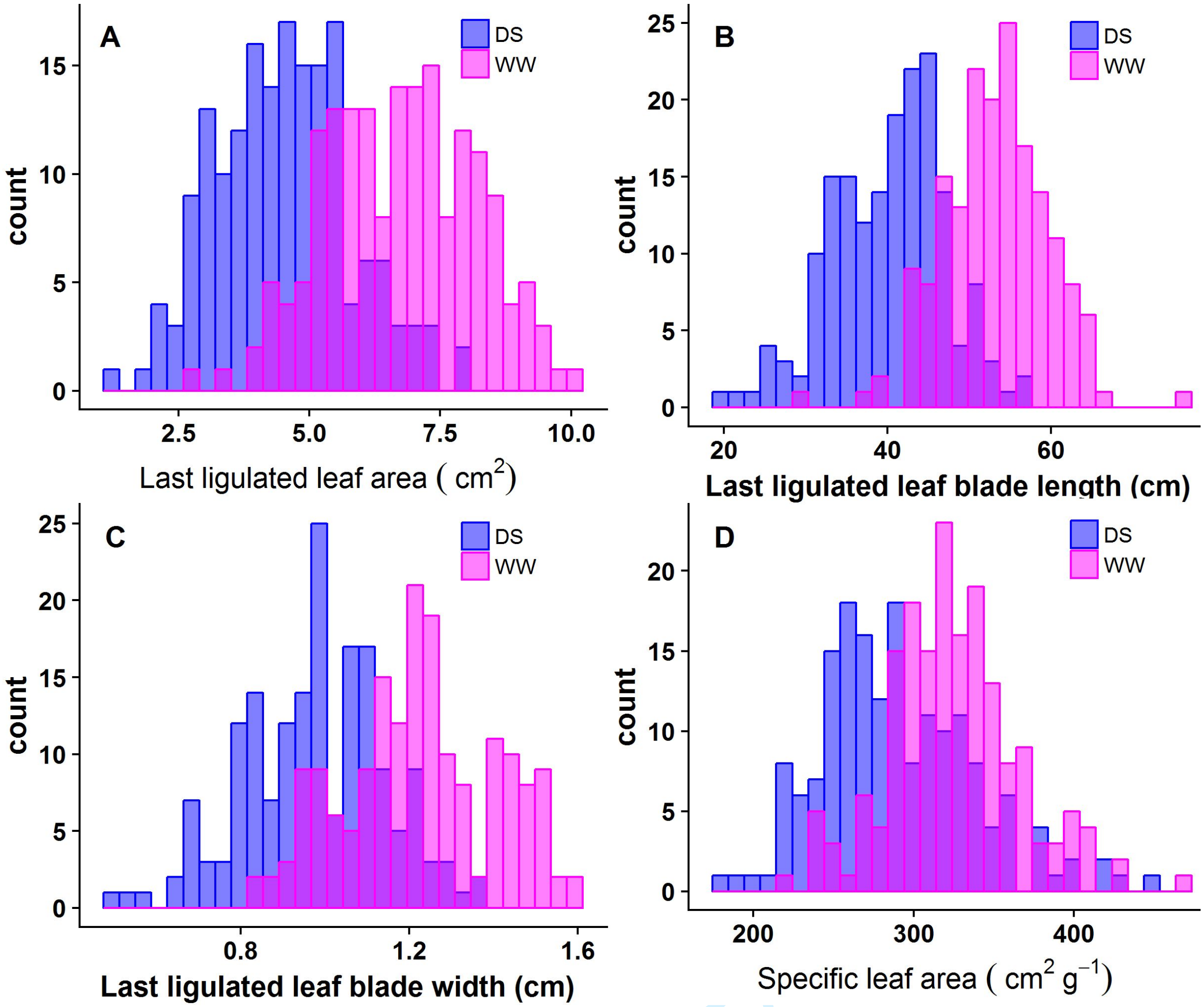

Supp. Fig. S5. Leaf morphology distributions among tropical japonical panel genotypes grown in the greenhouse. The last ligulated leaf was measured when the soil dried to FTSW=0.2 to determine A) leaf area, B) leaf length, C) leaf width, and D) specific leaf area.

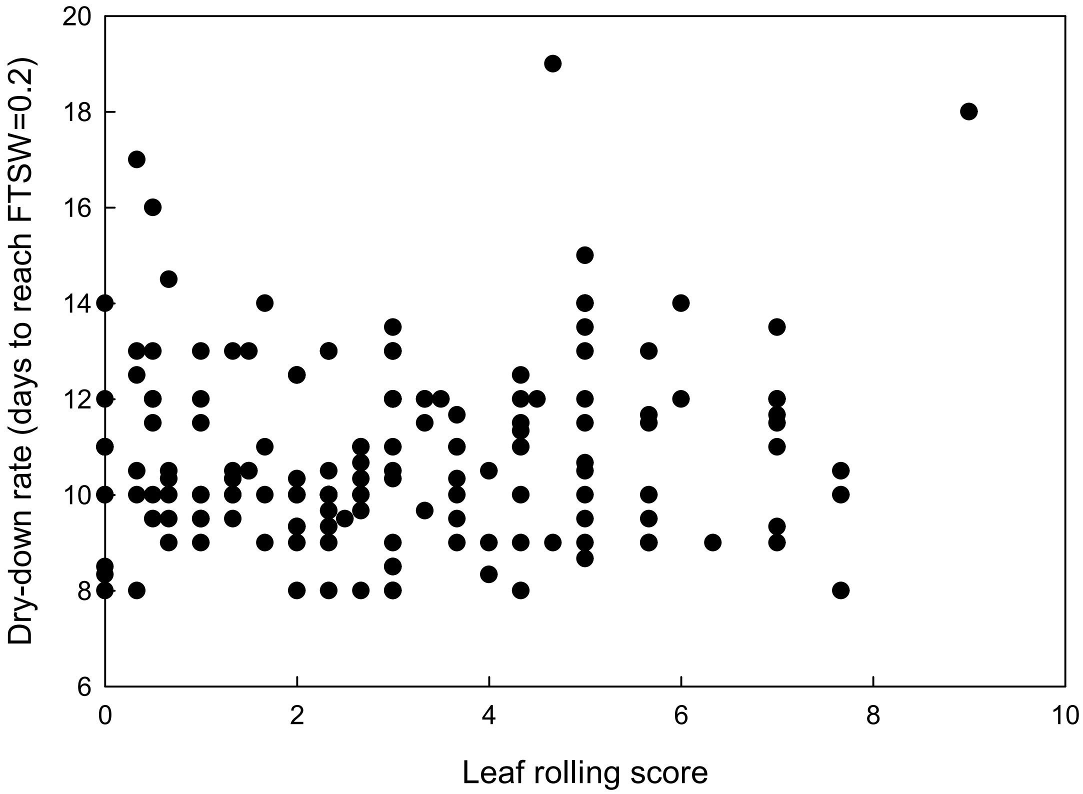

Supp. Fig. S6. Relationship between mean leaf rolling score of 156 japonica genotypes at the end of the greenhouse study and the time required for the soil moisture level of each genotype to reach FTSW of 0.2.

stribute

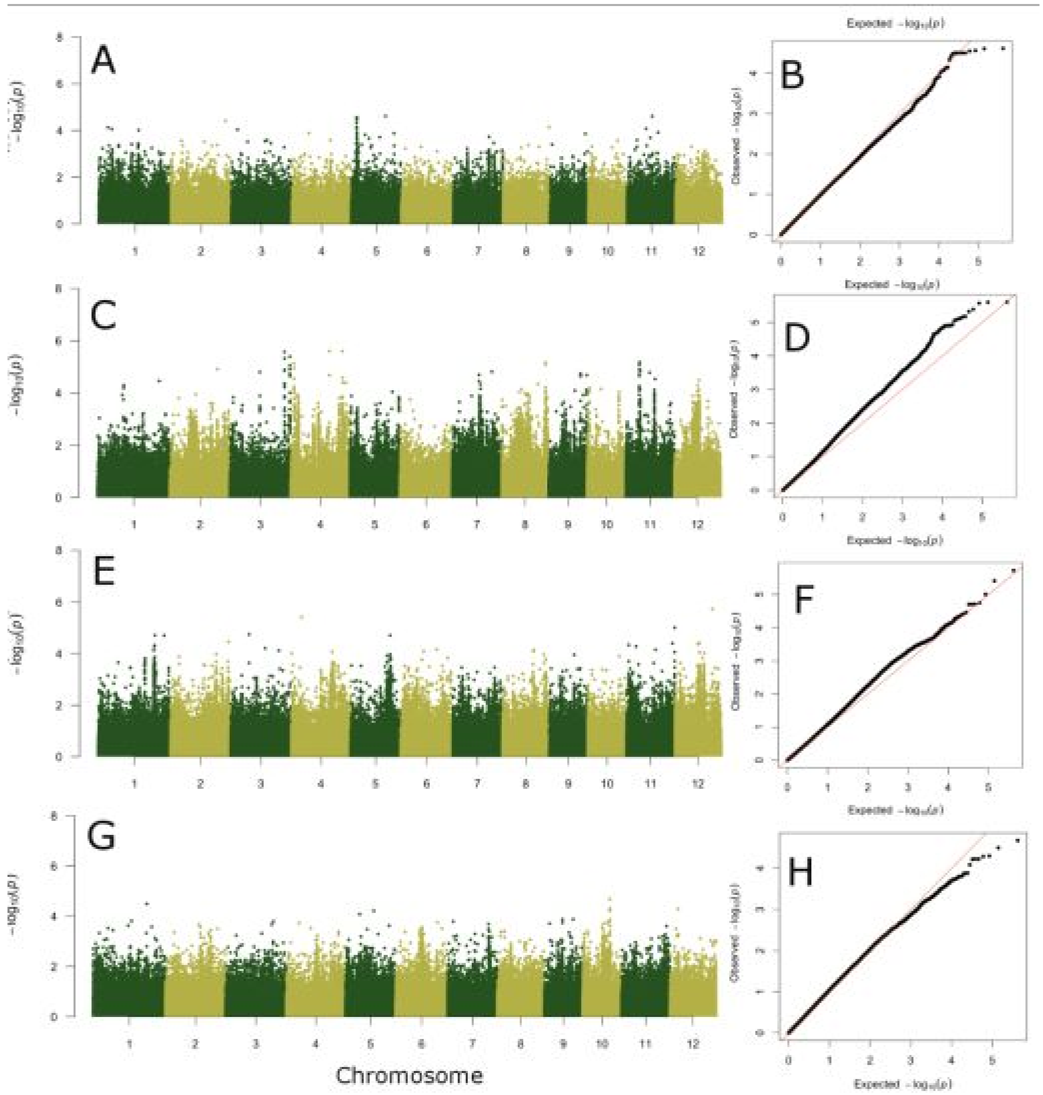

Supp. Fig. S7. Manhattan plots and Quantile-Quantile plots of genome-wide association analysis in the aus panel for A-B) leaf rolling score in the 2010 field drought trial; C-D) leaf rolling score in the 2012 field drought trial on medium-duration genotypes (74 DAS); E-F) change in NDVI in the 2010 field drought trial; G-H) change in NDVI in the 2012 field drought trial on medium-duration genotypes.

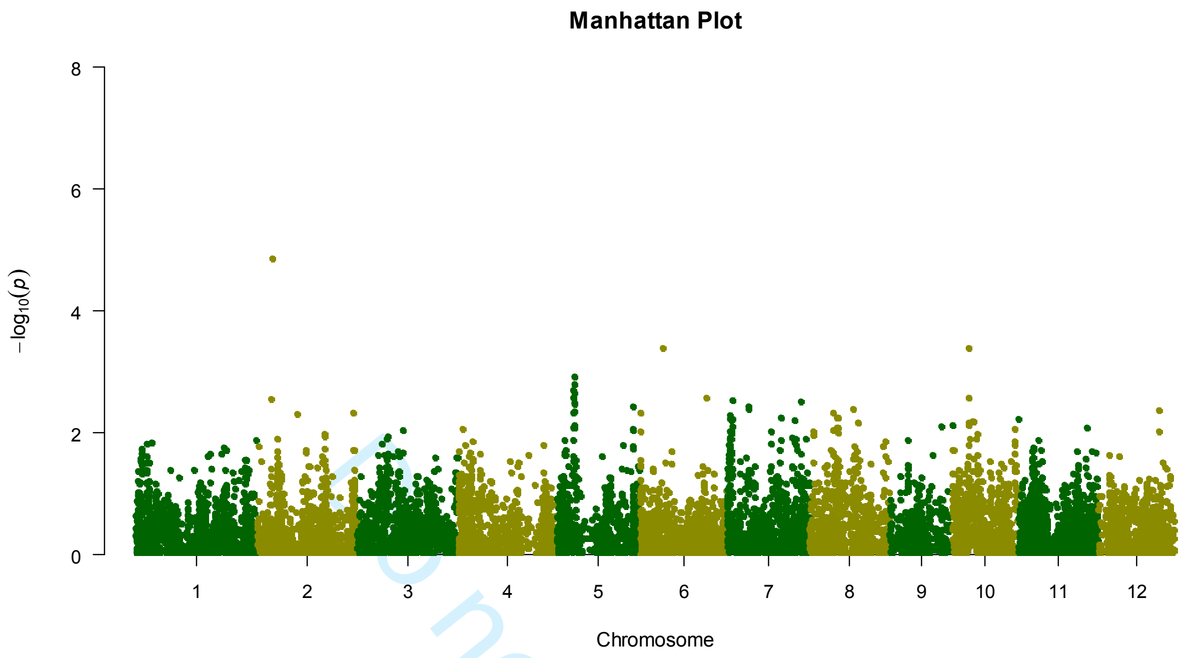

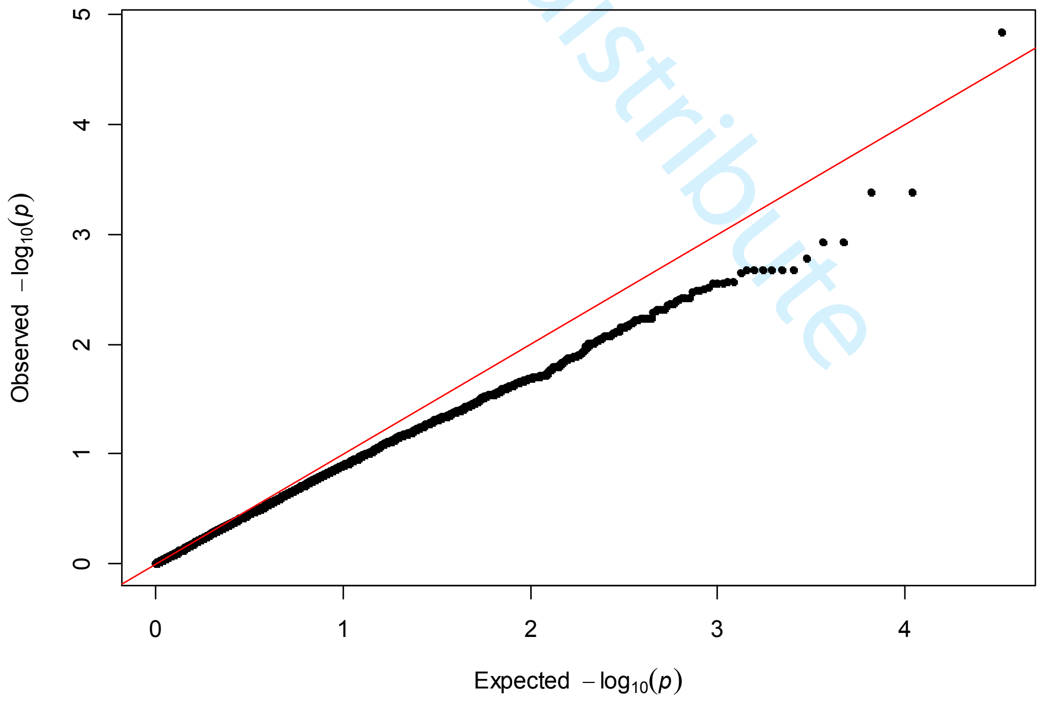

Do not distribute

Supp Fig S8. Manhattan plot and Quantile-Quantile plot of genome-wide association analysis of leaf rolling score in the greenhouse study of 172 tropical japonica genotypes.

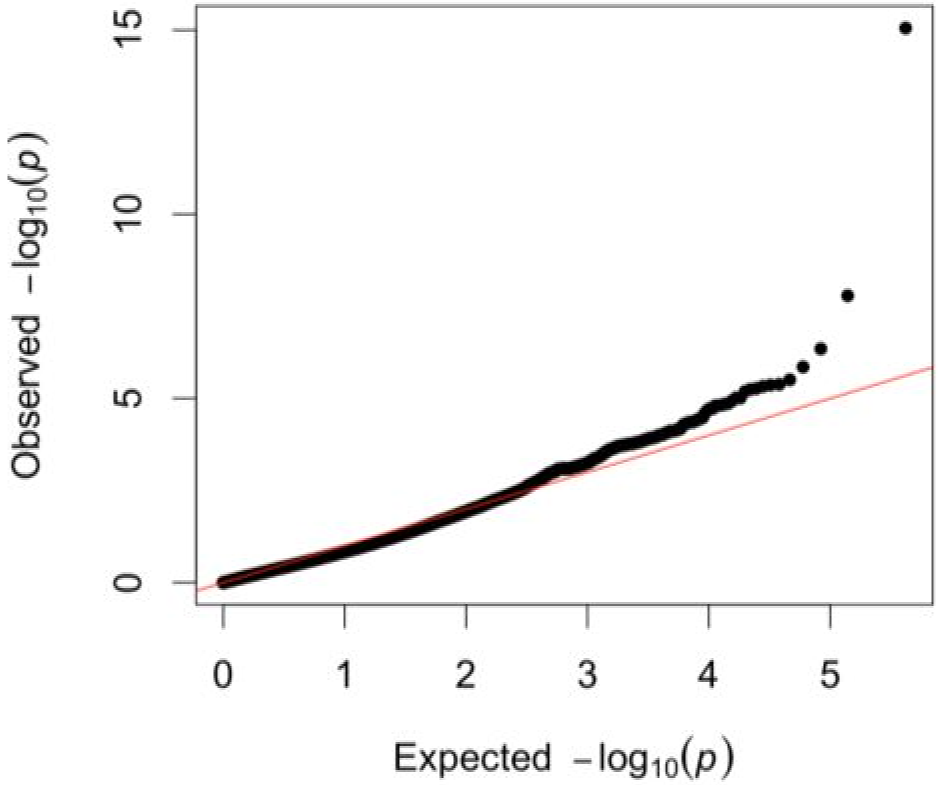

Supp. Fig. S9. Quantile-Quantile plot of genome-wide association analysis in the aus panel leaf rolling score in the 2012 field drought trial on medium-duration genotypes (106 DAS), adjusted for effects of marker density and correlation.

Supp. Table S11. List of top markers (−log10(P-value) > 4.0) from association mapping using

Trait	Marker	Chromosome	Position	P-Value
BG02_drought_LRS_11	SNP-8.6037049.	8	6038045	1.13E-07
BG02_drought_LRS_11	SNP-8.6028753.	8	6029749	1.52E-07
BG02_drought_LRS_11	SNP-8.6084695.	8	6085691	5.46E-07
BG02_drought_LRS_11	SNP-8.6060024.	8	6061020	1.83E-06
BG02_drought_LRS_11	SNP-8.6076486.	8	6077482	1.93E-06
BG02_drought_LRS_11	SNP-8.6065682.	8	6066678	4.44E-06
BG02_drought_LRS_11	SNP-8.6032002.	8	6032998	4.49E-06
BG02_drought_LRS_11	SNP-8.5966904.	8	5967902	7.55E-06
BG02_drought_LRS_11	SNP-8.6007841.	8	6008839	8.39E-06
BG02_drought_LRS_11	SNP-8.6009143.	8	6010141	8.39E-06
BG02_drought_LRS_11	SNP-8.6037501.	8	6038497	1.01E-05
BG02_drought_LRS_11	SNP-8.6112578.	8	6113574	1.03E-05
BG02_drought_LRS_11	SNP-8.5986389.	8	5987387	1.04E-05
BG02_drought_LRS_11	SNP-8.5958671.	8	5959669	1.21E-05
BG02_drought_LRS_11	SNP-8.5985610.	8	5986608	1.21E-05
BG02_drought_LRS_11	SNP-8.6007631.	8	6008629	1.78E-05
BG02_drought_LRS_11	SNP-8.6045764.	8	6046760	1.98E-05
BG02_drought_LRS_11	SNP-8.6019884.	8	6020882	2.05E-05
BG02_drought_LRS_11	SNP-8.5987048.	8	5988046	2.23E-05
BG02_drought_LRS_11	SNP-8.6097384.	8	6098380	2.28E-05
BG02_drought_LRS_11	SNP-8.6046730.	8	6047726	2.39E-05
BG02_drought_LRS_11	SNP-8.6036179.	8	6037175	2.44E-05
BG02_drought_LRS_11	SNP-8.6047080.	8	6048076	2.77E-05
BG02_drought_LRS_11	SNP-8.6037290.	8	6038286	3.08E-05
BG02_drought_LRS_11	SNP-8.6101543.	8	6102539	3.47E-05
BG02_drought_LRS_11	SNP-3.23618157.	3	23619984	5.27E-05
BG02_drought_LRS_11	SNP-8.8607335.	8	8608332	5.96E-05
BG02_drought_LRS_11	SNP-8.6023073.	8	6024071	6.02E-05
BG02_drought_LRS_11	SNP-8.5986335.	8	5987333	6.62E-05
LRI_2010DSstress_3220	SNP-5.20416723.	5	20479253	2.41E-05
LRI_2010DSstress_3220	SNP-11.14571305.	11	15034992	2.45E-05
LRI_2010DSstress_3220	SNP-5.3181430.	5	3181455	2.73E-05
LRI_2010DSstress_3220	SNP-5.3300703.	5	3300728	2.82E-05
LRI_2010DSstress_3220	SNP-5.3244692.	5	3244717	3.13E-05
LRI_2010DSstress_3220	SNP-5.3332632.	5	3332658	3.13E-05
LRI_2010DSstress_3220	SNP-5.3332728.	5	3332754	3.13E-05
LRI_2010DSstress_3220	SNP-5.3341514.	5	3341540	3.13E-05
LRI_2010DSstress_3220	SNP-5.3343208.	5	3343234	3.13E-05
LRI_2010DSstress_3220	SNP-5.3201962.	5	3201987	3.31E-05
LRI_2010DSstress_3220	SNP-2.32442548.	2	32448418	3.82E-05
LRI_2010DSstress_3220	SNP-5.3184658.	5	3184683	4.60E-05
LRI_2010DSstress_3220	SNP-5.3203564.	5	3203589	7.15E-05

Do not distribute

LRI_2010DSstress_3220	SNP-8.27763141.	8	27765856	7.18E-05
LRI_2010DSstress_3220	SNP-1.5354222.	1	5355223	7.42E-05
LRI_2010DSstress_3220	SNP-5.3203805.	5	3203830	8.32E-05
LRI_2010DSstress_3220	SNP-11.11262377.	11	11268061	8.38E-05
LRI_2010DSstress_3220	SNP-1.7745250.	1	7746251	8.63E-05
LRI_2010DSstress_3220	SNP-3.3761482.	3	3762487	9.16E-05
LRI_2010DSstress_3220	SNP-1.23807197.	1	23808242	9.60E-05
LRI_2012DS9M_39	SNP-4.22897111.	4	23082258	2.48E-06
LRI_2012DS9M_39	SNP-4.30734517.	4	30919639	2.52E-06
LRI_2012DS9M_39	SNP-3.32623574.	3	32630704	2.68E-06
LRI_2012DS9M_39	SNP-3.35883593.	3	35890725	4.06E-06
LRI_2012DS9M_39	SNP-3.32623501.	3	32630631	4.75E-06
LRI_2012DS9M_39	SNP-11.8283396.	11	8288787	6.65E-06
LRI_2012DS9M_39	SNP-8.26425296.	8	26428011	6.90E-06
LRI_2012DS9M_39	SNP-4.1941121.	4	1945526	7.58E-06
LRI_2012DS9M_39	SNP-8.26408114.	8	26410829	7.96E-06
LRI_2012DS9M_39	SNP-11.8030743.	11	8034999	8.41E-06
LRI_2012DS9M_39	SNP-3.35864108.	3	35871240	8.83E-06
LRI_2012DS9M_39	SNP-4.1857526.	4	1861931	1.15E-05
LRI_2012DS9M_39	SNP-2.28362463.	2	28368333	1.22E-05
LRI_2012DS9M_39	SNP-3.35875761.	3	35882893	1.23E-05
LRI_2012DS9M_39	SNP-11.8094798.	11	8099054	1.25E-05
LRI_2012DS9M_39	SNP-11.8177273.	11	8181529	1.25E-05
LRI_2012DS9M_39	SNP-11.8186598.	11	8190854	1.25E-05
LRI_2012DS9M_39	SNP-11.8195315.	11	8200706	1.25E-05
LRI_2012DS9M_39	SNP-3.32664804.	3	32671934	1.30E-05
LRI_2012DS9M_39	SNP-3.32632974.	3	32640104	1.40E-05
LRI_2012DS9M_39	SNP-11.8392473.	11	8397864	1.42E-05
LRI_2012DS9M_39	SNP-11.8401660.	11	8407051	1.46E-05
LRI_2012DS9M_39	SNP-7.23737864.	7	23738858	1.54E-05
LRI_2012DS9M_39	SNP-3.17895386.	3	17896605	1.59E-05
LRI_2012DS9M_39	SNP-11.13736585.	11	14200265	1.67E-05
LRI_2012DS9M_39	SNP-9.18971485.	9	18972486	1.82E-05
LRI_2012DS9M_39	SNP-3.35896791.	3	35903923	1.88E-05
LRI_2012DS9M_39	SNP-11.8043271.	11	8047527	1.98E-05
LRI_2012DS9M_39	SNP-7.16111463.	7	16112457	2.03E-05
LRI_2012DS9M_39	SNP-9.22815231.	9	22815713	2.09E-05
LRI_2012DS9M_39	SNP-4.22858472.	4	23043581	2.11E-05
LRI_2012DS9M_39	SNP-9.19031926.	9	19032927	2.17E-05
LRI_2012DS9M_39	SNP-11.8060472.	11	8064728	2.21E-05
LRI_2012DS9M_39	SNP-3.35895034.	3	35902166	2.25E-05
LRI_2012DS9M_39	SNP-9.19026790.	9	19027791	2.27E-05
LRI_2012DS9M_39	SNP-4.30753140.	4	30938261	2.65E-05
LRI_2012DS9M_39	SNP-4.1880540.	4	1884945	2.79E-05

Do not distribute

LRI_2012DS9M_39	SNP-11.17010168.	11	17476314	2.90E-05
LRI_2012DS9M_39	SNP-12.14734414.	12	14737074	3.25E-05
LRI_2012DS9M_39	SNP-1.36788409.	1	36789453	3.50E-05
LRI_2012DS9M_39	SNP-7.16131292.	7	16132286	3.53E-05
LRI_2012DS9M_39	SNP-4.30974019.	4	31159129	3.97E-05
LRI_2012DS9M_39	SNP-4.32863705.	4	33048819	4.14E-05
LRI_2012DS9M_39	SNP-3.32629719.	3	32636849	4.36E-05
LRI_2012DS9M_39	SNP-8.22049363.	8	22052077	4.56E-05
LRI_2012DS9M_39	SNP-3.32641603.	3	32648733	4.60E-05
LRI_2012DS9M_39	SNP-3.32681977.	3	32689107	4.60E-05
LRI_2012DS9M_39	SNP-7.16907502.	7	16908496	4.66E-05
LRI_2012DS9M_39	SNP-1.15685372.	1	15686399	5.12E-05
LRI_2012DS9M_39	SNP-12.13975318.	12	13977979	5.15E-05
LRI_2012DS9M_39	SNP-11.8029594.	11	8033850	5.54E-05
LRI_2012DS9M_39	SNP-4.1992357.	4	1996762	5.77E-05
LRI_2012DS9M_39	SNP-3.32628291.	3	32635421	6.01E-05
LRI_2012DS9M_39	SNP-8.26408505.	8	26411220	6.20E-05
LRI_2012DS9M_39	SNP-10.1359883.	10	1360907	6.41E-05
LRI_2012DS9M_39	SNP-1.15441262.	1	15442289	6.44E-05
LRI_2012DS9M_39	SNP-11.8029703.	11	8033959	6.67E-05
LRI_2012DS9M_39	SNP-7.16898583.	7	16899577	6.91E-05
LRI_2012DS9M_39	SNP-10.8250333.	10	8321472	7.02E-05
LRI_2012DS9M_39	SNP-8.11945227.	8	11946223	7.09E-05
LRI_2012DS9M_39	SNP-4.1878688.	4	1883093	7.18E-05
LRI_2012DS9M_39	SNP-4.1909815.	4	1914220	7.18E-05
LRI_2012DS9M_39	SNP-11.8431318.	11	8436709	7.30E-05
LRI_2012DS9M_39	SNP-12.14731896.	12	14734556	7.52E-05
LRI_2012DS9M_39	SNP-9.22450186.	9	22450668	7.64E-05
LRI_2012DS9M_39	SNP-11.16540542.	11	17006691	7.67E-05
LRI_2012DS9M_39	SNP-4.1907177.	4	1911582	7.85E-05
LRI_2012DS9M_39	SNP-11.8032085.	11	8036341	8.02E-05
LRI_2012DS9M_39	SNP-9.15651094.	9	15652096	8.08E-05
LRI_2012DS9M_39	SNP-10.1353241.	10	1354265	8.35E-05
LRI_2012DS9M_39	SNP-8.26494615.	8	26497330	8.54E-05
LRI_2012DS9M_39	SNP-12.13761047.	12	13763710	8.57E-05
LRI_2012DS9M_39	SNP-5.25277479.	5	25340059	9.06E-05
LRI_2012DS9M_39	SNP-8.10661359.	8	10662356	9.23E-05
LRI_2012DS9M_39	SNP-10.19001674.	10	19073118	9.28E-05
LRI_2012DS9M_39	SNP-3.35910927.	3	35918059	9.92E-05
LRI_2012DS9M_39	SNP-9.22814711.	9	22815193	9.93E-05
LRI_2012DS9M_39	SNP-8.26432403.	8	26435118	9.99E-05
LRI_2012DS9M_410	SNP-9.22747168.	9	22747650	2.47E-09
LRI_2012DS9M_410	SNP-9.22739510.	9	22739992	1.12E-08
LRI_2012DS9M_410	SNP-9.22764425.	9	22764907	2.07E-08

Do not distribute

LRI_2012DS9M_410	SNP-9.22715928.	9	22716410	2.53E-08
LRI_2012DS9M_410	SNP-9.22730307.	9	22730789	2.53E-08
LRI_2012DS9M_410	SNP-9.22748888.	9	22749370	2.53E-08
LRI_2012DS9M_410	SNP-9.22755366.	9	22755848	2.53E-08
LRI_2012DS9M_410	SNP-9.22755877.	9	22756359	2.53E-08
LRI_2012DS9M_410	SNP-9.22771900.	9	22772382	2.53E-08
LRI_2012DS9M_410	SNP-9.22781961.	9	22782443	2.53E-08
LRI_2012DS9M_410	SNP-8.17845916.	8	17848630	9.28E-08
LRI_2012DS9M_410	SNP-7.1447398.	7	1448399	1.23E-07
LRI_2012DS9M_410	SNP-5.16218247.	5	16275766	2.01E-07
LRI_2012DS9M_410	SNP-9.22743768.	9	22744250	4.57E-07
LRI_2012DS9M_410	SNP-6.18621788.	6	18622786	5.65E-07
LRI_2012DS9M_410	SNP-9.22712391.	9	22712873	5.71E-07
LRI_2012DS9M_410	SNP-5.16244228.	5	16301747	6.90E-07
LRI_2012DS9M_410	SNP-9.22814711.	9	22815193	1.04E-06
LRI_2012DS9M_410	SNP-5.668559.	5	668560	1.07E-06
LRI_2012DS9M_410	SNP-7.16249268.	7	16250262	1.26E-06
LRI_2012DS9M_410	SNP-1.1349206.	1	1350207	1.43E-06
LRI_2012DS9M_410	SNP-5.710311.	5	710312	1.55E-06
LRI_2012DS9M_410	SNP-11.8462793.	11	8468184	1.56E-06
LRI_2012DS9M_410	SNP-4.4550572.	4	4554970	1.60E-06
LRI_2012DS9M_410	SNP-9.22775795.	9	22776277	1.71E-06
LRI_2012DS9M_410	SNP-3.467282.	3	468285	1.95E-06
LRI_2012DS9M_410	SNP-5.759988.	5	760007	2.12E-06
LRI_2012DS9M_410	SNP-9.22804298.	9	22804780	2.14E-06
LRI_2012DS9M_410	SNP-7.10838197.	7	10839192	2.14E-06
LRI_2012DS9M_410	SNP-5.706340.	5	706341	2.23E-06
LRI_2012DS9M_410	SNP-9.17801317.	9	17802319	2.31E-06
LRI_2012DS9M_410	SNP-9.22815133.	9	22815615	2.32E-06
LRI_2012DS9M_410	SNP-3.470581.	3	471584	2.34E-06
LRI_2012DS9M_410	SNP-10.1351817.	10	1352841	2.38E-06
LRI_2012DS9M_410	SNP-2.697617.	2	697618	2.41E-06
LRI_2012DS9M_410	SNP-7.10878827.	7	10879822	2.58E-06
LRI_2012DS9M_410	SNP-9.22802209.	9	22802691	3.00E-06
LRI_2012DS9M_410	SNP-9.22734093.	9	22734575	3.13E-06
LRI_2012DS9M_410	SNP-8.26892872.	8	26895587	3.47E-06
LRI_2012DS9M_410	SNP-9.19027230.	9	19028231	3.54E-06
LRI_2012DS9M_410	SNP-9.22789525.	9	22790007	3.65E-06
LRI_2012DS9M_410	SNP-5.16171672.	5	16229191	3.90E-06
LRI_2012DS9M_410	SNP-11.8265704.	11	8271095	3.93E-06
LRI_2012DS9M_410	SNP-3.485459.	3	486462	4.15E-06
LRI_2012DS9M_410	SNP-3.11352559.	3	11353842	4.42E-06
LRI_2012DS9M_410	SNP-7.10435568.	7	10436563	4.54E-06
LRI_2012DS9M_410	SNP-8.26947729.	8	26950444	4.70E-06

Do not distribute

LRI_2012DS9M_410	SNP-11.884294.	11	885294	4.70E-06
LRI_2012DS9M_410	SNP-8.12258202.	8	12259198	4.74E-06
LRI_2012DS9M_410	SNP-3.1249820.	3	1250823	5.09E-06
LRI_2012DS9M_410	SNP-9.22760033.	9	22760515	5.13E-06
LRI_2012DS9M_410	SNP-5.535778.	5	535779	5.27E-06
LRI_2012DS9M_410	SNP-8.16803942.	8	16806657	5.45E-06
LRI_2012DS9M_410	SNP-3.22734125.	3	22735637	6.27E-06
LRI_2012DS9M_410	SNP-3.1233888.	3	1234891	6.43E-06
LRI_2012DS9M_410	SNP-5.723539.	5	723540	6.57E-06
LRI_2012DS9M_410	SNP-8.7294372tc.	8	7295368	6.63E-06
LRI_2012DS9M_410	SNP-6.5290712.	6	5291712	7.12E-06
LRI_2012DS9M_410	SNP-8.26894162.	8	26896877	7.24E-06
LRI_2012DS9M_410	SNP-3.14121543.	3	14122855	8.23E-06
LRI_2012DS9M_410	SNP-5.21560117.	5	21622676	8.36E-06
LRI_2012DS9M_410	SNP-9.22807391.	9	22807873	8.53E-06
LRI_2012DS9M_410	SNP-8.6085166.	8	6086162	8.64E-06
LRI_2012DS9M_410	SNP-7.5410421.	7	5411421	8.85E-06
LRI_2012DS9M_410	SNP-9.22815231.	9	22815713	9.27E-06
LRI_2012DS9M_410	SNP-5.696105.	5	696106	9.28E-06
LRI_2012DS9M_410	SNP-3.17931695.	3	17932914	9.61E-06
LRI_2012DS9M_410	SNP-4.17899849.	4	18071809	9.69E-06
LRI_2012DS9M_410	SNP-5.692901.	5	692902	9.90E-06
LRI_2012DS9M_410	SNP-7.24500602.	7	24501597	9.95E-06
LRI_2012DS9M_410	SNP-5.21563942.	5	21626501	1.01E-05
LRI_2012DS9M_410	SNP-9.7952056.	9	7953057	1.04E-05
LRI_2012DS9M_410	SNP-5.24532943.	5	24595524	1.05E-05
LRI_2012DS9M_410	SNP-9.7958331.	9	7959332	1.08E-05
LRI_2012DS9M_410	SNP-6.10431671.	6	10432671	1.15E-05
LRI_2012DS9M_410	SNP-11.24593374.	11	25059535	1.18E-05
LRI_2012DS9M_410	SNP-7.5264335.	7	5265335	1.19E-05
LRI_2012DS9M_410	SNP-2.33020521.	2	33026391	1.28E-05
LRI_2012DS9M_410	SNP-11.19583023.	11	20049101	1.34E-05
LRI_2012DS9M_410	SNP-5.762144.	5	762163	1.39E-05
LRI_2012DS9M_410	SNP-11.17663613.	11	18129756	1.42E-05
LRI_2012DS9M_410	SNP-6.15588618.	6	15589617	1.43E-05
LRI_2012DS9M_410	SNP-4.1903154.	4	1907559	1.44E-05
LRI_2012DS9M_410	SNP-1.382274.	1	383275	1.45E-05
LRI_2012DS9M_410	SNP-9.22741924.	9	22742406	1.45E-05
LRI_2012DS9M_410	SNP-2.969013.	2	969014	1.52E-05
LRI_2012DS9M_410	SNP-7.15518610.	7	15519604	1.58E-05
LRI_2012DS9M_410	SNP-1.4714542.	1	4715543	1.71E-05
LRI_2012DS9M_410	SNP-8.5620515.	8	5621513	1.74E-05
LRI_2012DS9M_410	SNP-5.658349.	5	658350	1.75E-05
LRI_2012DS9M_410	SNP-5.658472.	5	658473	1.75E-05

Do not distribute

LRI_2012DS9M_410	SNP-5.757571.	5	757572	1.75E-05
LRI_2012DS9M_410	SNP-9.10042163.	9	10043165	1.76E-05
LRI_2012DS9M_410	SNP-7.15008730.	7	15009724	1.76E-05
LRI_2012DS9M_410	SNP-8.12269463.	8	12270459	1.82E-05
LRI_2012DS9M_410	SNP-6.5675174.	6	5676174	1.83E-05
LRI_2012DS9M_410	SNP-12.19905955.	12	19934486	1.83E-05
LRI_2012DS9M_410	SNP-2.20783970.	2	20789839	1.85E-05
LRI_2012DS9M_410	SNP-8.4078059.	8	4079057	1.89E-05
LRI_2012DS9M_410	SNP-7.8526197.	7	8527193	1.89E-05
LRI_2012DS9M_410	SNP-2.20700225.	2	20706094	1.97E-05
LRI_2012DS9M_410	SNP-2.20783642.	2	20789511	1.97E-05
LRI_2012DS9M_410	SNP-1.309100.	1	310101	2.03E-05
LRI_2012DS9M_410	SNP-7.16408098.	7	16409092	2.09E-05
LRI_2012DS9M_410	SNP-4.1822146.	4	1826552	2.15E-05
LRI_2012DS9M_410	SNP-9.19026790.	9	19027791	2.17E-05
LRI_2012DS9M_410	SNP-11.10431465.	11	10436921	2.29E-05
LRI_2012DS9M_410	SNP-3.3265257.	3	3266262	2.34E-05
LRI_2012DS9M_410	SNP-5.684424.	5	684425	2.42E-05
LRI_2012DS9M_410	SNP-2.22196750.	2	22202620	2.46E-05
LRI_2012DS9M_410	SNP-12.12685827.	12	12688489	2.48E-05
LRI_2012DS9M_410	SNP-8.17393008.	8	17395723	2.49E-05
LRI_2012DS9M_410	SNP-2.20537389.	2	20543258	2.49E-05
LRI_2012DS9M_410	SNP-7.15630189.	7	15631183	2.59E-05
LRI_2012DS9M_410	SNP-1.37873789.	1	37874833	2.59E-05
LRI_2012DS9M_410	SNP-8.26918427.	8	26921142	2.61E-05
LRI_2012DS9M_410	SNP-1.16730474.	1	16731501	2.70E-05
LRI_2012DS9M_410	SNP-1.56031.	1	57032	2.71E-05
LRI_2012DS9M_410	SNP-5.738504.	5	738505	2.79E-05
LRI_2012DS9M_410	SNP-5.692270.	5	692271	2.87E-05
LRI_2012DS9M_410	SNP-5.21575740.	5	21638299	2.88E-05
LRI_2012DS9M_410	SNP-11.26453660.	11	26925274	3.01E-05
LRI_2012DS9M_410	SNP-5.16248343.	5	16305862	3.02E-05
LRI_2012DS9M_410	SNP-5.15753483.	5	15810943	3.05E-05
LRI_2012DS9M_410	SNP-8.16889348.	8	16892063	3.17E-05
LRI_2012DS9M_410	SNP-9.17554761.	9	17555763	3.19E-05
LRI_2012DS9M_410	SNP-10.2480329.	10	2481353	3.24E-05
LRI_2012DS9M_410	SNP-5.10306322.	5	10306394	3.26E-05
LRI_2012DS9M_410	SNP-4.3678390.	4	3682785	3.33E-05
LRI_2012DS9M_410	SNP-4.13486071.	4	13495358	3.38E-05
LRI_2012DS9M_410	SNP-8.10287128.	8	10288125	3.39E-05
LRI_2012DS9M_410	SNP-9.8200833.	9	8201834	3.40E-05
LRI_2012DS9M_410	SNP-5.8419615.	5	8419688	3.52E-05
LRI_2012DS9M_410	SNP-8.16901067.	8	16903782	3.53E-05
LRI_2012DS9M_410	SNP-4.4643132.	4	4647693	3.56E-05

Do not distribute

LRI_2012DS9M_410	SNP-3.3262776.	3	3263781	3.63E-05
LRI_2012DS9M_410	SNP-7.15646764.	7	15647758	3.66E-05
LRI_2012DS9M_410	SNP-5.684336.	5	684337	3.81E-05
LRI_2012DS9M_410	SNP-8.15016852.	8	15019567	3.82E-05
LRI_2012DS9M_410	SNP-7.16065417.	7	16066411	3.82E-05
LRI_2012DS9M_410	SNP-1.2970211.	1	2971212	3.83E-05
LRI_2012DS9M_410	SNP-8.17064811.	8	17067526	3.98E-05
LRI_2012DS9M_410	SNP-3.17157082.	3	17158273	4.07E-05
LRI_2012DS9M_410	SNP-7.19651280.	7	19652274	4.19E-05
LRI_2012DS9M_410	SNP-9.16263546.	9	16264548	4.21E-05
LRI_2012DS9M_410	SNP-5.721300.	5	721301	4.22E-05
LRI_2012DS9M_410	SNP-2.24313691.	2	24319561	4.29E-05
LRI_2012DS9M_410	SNP-8.17270971.	8	17273686	4.38E-05
LRI_2012DS9M_410	SNP-9.16400352.	9	16401354	4.51E-05
LRI_2012DS9M_410	SNP-8.12055099.	8	12056095	4.51E-05
LRI_2012DS9M_410	SNP-7.14682372.	7	14683366	4.54E-05
LRI_2012DS9M_410	SNP-3.29801362.	3	29808323	4.56E-05
LRI_2012DS9M_410	SNP-10.525804.	10	526829	4.70E-05
LRI_2012DS9M_410	SNP-7.15575440.	7	15576434	4.72E-05
LRI_2012DS9M_410	SNP-5.758772.	5	758773	4.73E-05
LRI_2012DS9M_410	SNP-3.6115018.	3	6116019	4.75E-05
LRI_2012DS9M_410	SNP-4.4040190.	4	4044584	4.83E-05
LRI_2012DS9M_410	SNP-8.18080367.	8	18083081	4.87E-05
LRI_2012DS9M_410	SNP-9.19031406.	9	19032407	5.01E-05
LRI_2012DS9M_410	SNP-6.8585898.	6	8586898	5.20E-05
LRI_2012DS9M_410	SNP-4.3683035.	4	3687430	5.20E-05
LRI_2012DS9M_410	SNP-7.19735018.	7	19736012	5.32E-05
LRI_2012DS9M_410	SNP-7.15408301.	7	15409295	5.41E-05
LRI_2012DS9M_410	SNP-7.15547252.	7	15548246	5.41E-05
LRI_2012DS9M_410	SNP-7.15552081.	7	15553075	5.41E-05
LRI_2012DS9M_410	SNP-7.15552494.	7	15553488	5.41E-05
LRI_2012DS9M_410	SNP-7.15626492.	7	15627486	5.41E-05
LRI_2012DS9M_410	SNP-8.11869527.	8	11870523	5.43E-05
LRI_2012DS9M_410	SNP-9.19017815.	9	19018816	5.47E-05
LRI_2012DS9M_410	SNP-11.18271437.	11	18737630	5.50E-05
LRI_2012DS9M_410	SNP-12.19926431.	12	19954962	5.52E-05
LRI_2012DS9M_410	SNP-11.10378147.	11	10383603	5.52E-05
LRI_2012DS9M_410	SNP-5.743262.	5	743263	5.54E-05
LRI_2012DS9M_410	SNP-9.19022407.	9	19023408	5.67E-05
LRI_2012DS9M_410	SNP-1.15135730.	1	15136757	5.87E-05
LRI_2012DS9M_410	SNP-5.745088.	5	745089	5.90E-05
LRI_2012DS9M_410	SNP-8.7795388.	8	7796385	5.92E-05
LRI_2012DS9M_410	SNP-7.8867065.	7	8868060	5.94E-05
LRI_2012DS9M_410	SNP-4.32000845.	4	32185953	5.95E-05

Do not distribute

LRI_2012DS9M_410	SNP-10.9511276.	10	9582456	5.95E-05
LRI_2012DS9M_410	SNP-2.6070090.	2	6070091	6.18E-05
LRI_2012DS9M_410	SNP-5.21591739.	5	21654298	6.23E-05
LRI_2012DS9M_410	SNP-2.33424278.	2	33430148	6.54E-05
LRI_2012DS9M_410	SNP-7.19653698.	7	19654692	6.59E-05
LRI_2012DS9M_410	SNP-4.356283.	4	357284	6.66E-05
LRI_2012DS9M_410	SNP-1.20399608.	1	20400654	6.67E-05
LRI_2012DS9M_410	SNP-2.6074811.	2	6074812	6.74E-05
LRI_2012DS9M_410	SNP-2.6077991.	2	6077992	6.74E-05
LRI_2012DS9M_410	SNP-2.6097956.	2	6097957	6.74E-05
LRI_2012DS9M_410	SNP-2.6111390.	2	6111391	6.74E-05
LRI_2012DS9M_410	SNP-6.13489062.	6	13490062	6.98E-05
LRI_2012DS9M_410	SNP-5.546275.	5	546276	6.99E-05
LRI_2012DS9M_410	SNP-1.20359524.	1	20360570	7.02E-05
LRI_2012DS9M_410	SNP-1.20412575.	1	20413621	7.02E-05
LRI_2012DS9M_410	SNP-3.1232437.	3	1233440	7.03E-05
LRI_2012DS9M_410	SNP-4.29064173.	4	29249317	7.03E-05
LRI_2012DS9M_410	SNP-3.24060747.	3	24062575	7.14E-05
LRI_2012DS9M_410	SNP-7.9336866.	7	9337861	7.29E-05
LRI_2012DS9M_410	SNP-5.541490.	5	541491	7.30E-05
LRI_2012DS9M_410	SNP-4.1880540.	4	1884945	7.34E-05
LRI_2012DS9M_410	SNP-6.29541712.	6	29542711	7.35E-05
LRI_2012DS9M_410	SNP-8.16642062.	8	16644777	7.37E-05
LRI_2012DS9M_410	SNP-11.22729505.	11	23195635	7.46E-05
LRI_2012DS9M_410	SNP-12.21087646.	12	21121097	7.69E-05
LRI_2012DS9M_410	SNP-1.25479473.	1	25480518	7.69E-05
LRI_2012DS9M_410	SNP-3.3187857.	3	3188862	7.87E-05
LRI_2012DS9M_410	SNP-11.13739090.	11	14202770	7.91E-05
LRI_2012DS9M_410	SNP-5.12248648.	5	12248719	7.91E-05
LRI_2012DS9M_410	SNP-4.3680408.	4	3684803	7.92E-05
LRI_2012DS9M_410	SNP-6.7381972.	6	7382972	8.00E-05
LRI_2012DS9M_410	SNP-7.3792591.	7	3793591	8.01E-05
LRI_2012DS9M_410	SNP-7.15536998.	7	15537992	8.02E-05
LRI_2012DS9M_410	SNP-10.8705676.	10	8776815	8.04E-05
LRI_2012DS9M_410	SNP-8.24772010.	8	24774725	8.07E-05
LRI_2012DS9M_410	SNP-8.3687063.	8	3688061	8.10E-05
LRI_2012DS9M_410	SNP-7.15661969.	7	15662963	8.37E-05
LRI_2012DS9M_410	SNP-4.1857526.	4	1861931	8.50E-05
LRI_2012DS9M_410	SNP-1.33862904.	1	33863949	8.80E-05
LRI_2012DS9M_410	SNP-6.22179562.	6	22180560	9.04E-05
LRI_2012DS9M_410	SNP-3.3265341.	3	3266346	9.18E-05
LRI_2012DS9M_410	SNP-7.21276037.	7	21277031	9.20E-05
LRI_2012DS9M_410	SNP-4.21777846.	4	21949796	9.22E-05
LRI_2012DS9M_410	SNP-3.3261948.	3	3262953	9.33E-05

Do not distribute

LRI_2012DS9M_410	SNP-3.3265533.	3	3266538	9.33E-05
LRI_2012DS9M_410	SNP-3.3267583.	3	3268588	9.33E-05
LRI_2012DS9M_410	SNP-3.3268511.	3	3269516	9.33E-05
LRI_2012DS9M_410	SNP-3.3270032.	3	3271037	9.33E-05
LRI_2012DS9M_410	SNP-3.3308351.	3	3309356	9.33E-05
LRI_2012DS9M_410	SNP-3.3280686.	3	3281691	9.41E-05
LRI_2012DS9M_410	SNP-11.26007964.	11	26479551	9.48E-05
LRI_2012DS9M_410	SNP-7.6716645.	7	6717641	9.52E-05
LRI_2012DS9M_410	SNP-1.42589572.	1	42590617	9.61E-05
LRI_2012DS9M_410	SNP-8.26914674.	8	26917389	9.71E-05
LRI_2012DS9M_410	SNP-11.16540542.	11	17006691	9.80E-05
LRI_2012DS9M_410	SNP-6.5058678.	6	5059678	9.85E-05
LRI_2012DS9M_410	SNP-8.13025239.	8	13027415	9.97E-05
NDVI_2010	SNP-12.22260614.	12	22294068	1.85E-06
NDVI_2010	SNP-4.6022995.	4	6027557	3.84E-06
NDVI_2010	SNP-11.28313594.	11	28836816	9.75E-06
NDVI_2010	SNP-3.11061115.	3	11062398	1.78E-05
NDVI_2010	SNP-1.33866589.	1	33867634	1.93E-05
NDVI_2010	SNP-5.23520041.	5	23582621	1.94E-05
NDVI_2010	SNP-1.39424951.	1	39425995	1.97E-05
NDVI_2010	SNP-2.34651145.	2	34657015	3.42E-05
NDVI_2010	SNP-12.14653220.	12	14655881	3.78E-05
NDVI_2010	SNP-11.26976127.	11	27447741	3.98E-05
NDVI_2010	SNP-12.13647132.	12	13649795	4.31E-05
NDVI_2010	SNP-11.1363182.	11	1364182	4.64E-05
NDVI_2010	SNP-1.33637662.	1	33638707	4.77E-05
NDVI_2010	SNP-11.5755836.	11	5760085	5.22E-05
NDVI_2010	SNP-1.33593652.	1	33594697	5.26E-05
NDVI_2010	SNP-3.20692745.	3	20694236	6.12E-05
NDVI_2010	SNP-6.21711969.	6	21712967	6.82E-05
NDVI_2010	SNP-11.16922298.	11	17388446	7.09E-05
NDVI_2010	SNP-8.19184314.	8	19187028	7.13E-05
NDVI_2010	SNP-1.33228301.	1	33229346	7.36E-05
NDVI_2010	SNP-3.28929496.	3	28936445	7.57E-05
NDVI_2010	SNP-6.13749087.	6	13750087	8.02E-05
NDVI_2010	SNP-8.18629180.	8	18631894	8.03E-05
NDVI_2010	SNP-4.24155492.	4	24340632	8.50E-05
NDVI_2010	SNP-11.28303653.	11	28826875	8.68E-05
NDVI_2010	SNP-12.14739527.	12	14742187	8.84E-05
NDVI_2010	SNP-12.17408642.	12	17414688	9.52E-05
NDVI_2010	SNP-4.147798.	4	148799	9.72E-05
NDVI_2011	SNP-1.9594078.	1	9595103	5.10E-07
NDVI_2011	SNP-1.9584809.	1	9585834	1.52E-06
NDVI_2011	SNP-1.9586957.	1	9587982	1.59E-06

Do not distribute

NDVI_2011	SNP-1.9583015.	1	9584040	4.87E-06
NDVI_2011	SNP-1.9597974.	1	9598999	4.88E-06
NDVI_2011	SNP-4.3851360.	4	3855757	4.93E-06
NDVI_2011	SNP-1.9599235.	1	9600260	5.29E-06
NDVI_2011	SNP-1.9606745.	1	9607770	5.29E-06
NDVI_2011	SNP-1.9603371.	1	9604396	8.04E-06
NDVI_2011	SNP-1.9585183.	1	9586208	1.06E-05
NDVI_2011	SNP-1.9624453.	1	9625478	1.06E-05
NDVI_2011	SNP-1.28847110.	1	28848156	1.35E-05
NDVI_2011	SNP-1.28742560.	1	28743606	1.70E-05
NDVI_2011	SNP-10.877381.	10	878406	3.13E-05
NDVI_2011	SNP-1.9599594.	1	9600619	3.48E-05
NDVI_2011	SNP-12.17619717.	12	17625763	3.71E-05
NDVI_2011	SNP-4.29695396.	4	29880527	4.37E-05
NDVI_2011	SNP-1.28868530.	1	28869576	5.10E-05
NDVI_2011	SNP-2.23945763.	2	23951633	5.73E-05
NDVI_2011	SNP-1.1911845.	1	1912846	5.82E-05
NDVI_2011	SNP-1.9600413.	1	9601438	6.29E-05
NDVI_2011	SNP-1.28810345.	1	28811391	7.16E-05
NDVI_2011	SNP-1.9581788.	1	9582813	7.29E-05
NDVI_2011	SNP-4.33138757.	4	33323868	7.40E-05
NDVI_2011	SNP-7.24448331.	7	24449326	7.53E-05
NDVI_2011	SNP-6.947949.	6	948949	8.33E-05
NDVI_2011	SNP-1.28792217.	1	28793263	8.48E-05
NDVI_2011	SNP-1.28811414.	1	28812460	8.98E-05
NDVI_2011	SNP-6.932310.	6	933310	9.42E-05
NDVI_2011	SNP-9.21418329.	9	21418811	9.51E-05
NDVI_2012	SNP-10.16200979.	10	16272204	2.11E-05
NDVI_2012	SNP-1.32079967.	1	32081012	3.19E-05
NDVI_2012	SNP-10.16306343.	10	16377568	5.02E-05
NDVI_2012	SNP-12.4965353.	12	4966368	5.19E-05
NDVI_2012	SNP-10.16171182.	10	16242409	5.98E-05
NDVI_2012	SNP-10.16255769.	10	16326994	5.98E-05
NDVI_2012	SNP-5.16819141.	5	16879501	6.06E-05
NDVI_2012	SNP-5.8428315.	5	8428388	8.26E-05

Do not distribute

; EMMAX model for leaf rolling scores and ΔNDVI from different experiments.

–log10 (P-valı	Bonferroni P	FDR	Proportion of Variance Explained
6.9472569	0.0238159	0.0238159	0.51820928
6.81914507	0.0319874	0.0159937	0.51102556
6.26310016	0.11508634	0.03836211	0.47864101
5.73705785	0.38642578	0.09660645	0.44613548
5.71548694	0.40610375	0.08122075	0.44476258
5.35280225	0.9360988	0.15601647	0.42119608
5.34779588	0.94695221	0.13527889	0.42086433
5.12184699	1	0.19915305	0.40570683
5.07625787	1	0.19661813	0.40260435
5.07625787	1	0.17695631	0.40260435
4.99620442	1	0.19343134	0.39712031
4.98826855	1	0.18058187	0.39657415
4.98340111	1	0.16856968	0.39623893
4.91801298	1	0.18196293	0.39171915
4.91689448	1	0.17027003	0.39164156
4.74863926	1	0.23516067	0.37986713
4.70378621	1	0.24540805	0.37669342
4.68829806	1	0.24018914	0.37559408
4.65236894	1	0.24717321	0.37303708
4.64254816	1	0.24018495	0.3723365
4.62196808	1	0.23984826	0.3708661
4.61327269	1	0.23357619	0.37024389
4.55780827	1	0.25385623	0.36626198
4.51143647	1	0.27069251	0.3629154
4.45989056	1	0.29261283	0.35917675
4.27787823	1	0.42783111	0.34581748
4.22456363	1	0.46579685	0.3418576
4.22014568	1	0.45375375	0.34152851
4.17945044	1	0.48114448	0.33849029
4.61807033	1	1	0.1902548
4.61020188	1	1	0.18992445
4.5644122	1	1	0.18799991
4.54949077	1	1	0.18737199
4.50426201	1	1	0.18546637
4.50426201	1	1	0.18546637
4.50426201	1	0.94354777	0.18546637
4.50426201	1	0.8256043	0.18546637
4.50426201	1	0.73387049	0.18546637
4.47971736	1	0.69888639	0.18443077
4.41803583	1	0.73231078	0.18182378
4.33719467	1	0.8086266	0.17839731
4.14579756	1	1	0.17024169

Do not distribute

4.14404775	1	1	0.17016685
4.12962546	1	1	0.16954983
4.08013377	1	1	0.16742991
4.07685561	1	1	0.16728935
4.0638845	1	1	0.16673303
4.03829044	1	1	0.16563452
4.01756564	1	1	0.16474423
5.60614026	0.52237655	0.52237655	0.31552734
5.59841106	0.53175659	0.2658783	0.31512768
5.57208089	0.56499298	0.18833099	0.3137646
5.39153492	0.85622721	0.2140568	0.30435202
5.32366648	1	0.20021078	0.30078395
5.17713018	1	0.23379893	0.29302442
5.16084903	1	0.20805438	0.29215758
5.12029203	1	0.19986738	0.28999417
5.09900503	1	0.18658486	0.28885634
5.07546039	1	0.17728155	0.28759596
5.05414141	1	0.16927384	0.28645302
4.93933632	1	0.20211864	0.28027046
4.91297783	1	0.19824524	0.27884439
4.90930485	1	0.18564834	0.27864547
4.90445936	1	0.17521582	0.27838298
4.90445936	1	0.16426483	0.27838298
4.90445936	1	0.1546022	0.27838298
4.90445936	1	0.14601319	0.27838298
4.8850264	1	0.14465849	0.27732944
4.85472955	1	0.14735484	0.27568424
4.84669197	1	0.14295939	0.27524723
4.8350319	1	0.14017461	0.27461286
4.81127061	1	0.1416203	0.27331862
4.79763133	1	0.14004944	0.27257481
4.77684923	1	0.14103753	0.2714402
4.74064128	1	0.14740401	0.26945976
4.7259574	1	0.14682594	0.26865528
4.70314773	1	0.14921696	0.26740411
4.69155008	1	0.14797075	0.26676724
4.67952274	1	0.14705505	0.26610627
4.67653707	1	0.14329307	0.26594211
4.66425436	1	0.14279717	0.26526645
4.65486319	1	0.14149686	0.26474949
4.64696058	1	0.13985708	0.26431423
4.64322337	1	0.13703532	0.26410832
4.57647924	1	0.15536107	0.26042251
4.55468458	1	0.15894162	0.25921554

Do not distribute

4.53734001	1	0.16106469	0.25825382
4.48872942	1	0.17552133	0.2555528
4.45624275	1	0.1844256	0.25374307
4.45251274	1	0.1814794	0.25353504
4.40155304	1	0.19921481	0.25068809
4.38348731	1	0.20284681	0.24967662
4.36047434	1	0.20902437	0.24838652
4.34115172	1	0.21367795	0.24730186
4.33717147	1	0.21095734	0.24707827
4.33717147	1	0.20646888	0.24707827
4.33126865	1	0.20493402	0.24674658
4.29101916	1	0.22024634	0.24448163
4.28791254	1	0.21739092	0.24430658
4.25683178	1	0.22894016	0.24255337
4.23891666	1	0.23399358	0.24154128
4.22084673	1	0.23933231	0.24051932
4.20732081	1	0.24233122	0.2397536
4.19343021	1	0.24565807	0.23896658
4.19090814	1	0.24267653	0.23882361
4.17580752	1	0.24685479	0.23796713
4.16068284	1	0.25119623	0.2371085
4.15366108	1	0.25096367	0.2367096
4.14906224	1	0.24940804	0.23644826
4.14385943	1	0.24827596	0.2361525
4.14385943	1	0.24427151	0.2361525
4.13686545	1	0.24429689	0.23575477
4.12363705	1	0.24791735	0.23500205
4.11670385	1	0.24803144	0.2346073
4.11546237	1	0.24497267	0.2345366
4.10505128	1	0.24717119	0.23394347
4.0957432	1	0.24881229	0.23341286
4.09271646	1	0.24692121	0.23324026
4.07851334	1	0.25148527	0.23242988
4.06849562	1	0.25372892	0.23185789
4.0670168	1	0.25105834	0.23177343
4.04308423	1	0.26164765	0.23040541
4.03482204	1	0.26306931	0.22993268
4.03264528	1	0.26086595	0.22980809
4.00362546	1	0.27522311	0.2281456
4.00298347	1	0.27205063	0.22810879
4.00035653	1	0.2701922	0.22795815
8.60745745	0.00052079	0.00052079	0.45536163
7.94992873	0.00236698	0.00118349	0.42725645
7.6841772	0.00436462	0.00145487	0.41550881

Do not distribute

7.59729251	0.00533128	0.00133282	0.41161861
7.59729251	0.00533128	0.00106626	0.41161861
7.59729251	0.00533128	0.00088855	0.41161861
7.59729251	0.00533128	0.00076161	0.41161861
7.59729251	0.00533128	0.00066641	0.41161861
7.59729251	0.00533128	0.00059236	0.41161861
7.59729251	0.00533128	0.00053313	0.41161861
7.03238528	0.01957666	0.0017797	0.38572146
6.91068264	0.02590844	0.00215904	0.38000339
6.69758396	0.04231945	0.00325534	0.36987092
6.34023314	0.09635867	0.00688276	0.35253241
6.24767107	0.11924849	0.0079499	0.34796978
6.24358789	0.12037493	0.00752343	0.34776782
6.16121049	0.14551691	0.00855982	0.34368113
5.98209614	0.21980006	0.01221111	0.334714
5.97187582	0.22503399	0.01184389	0.33419896
5.89975958	0.26568336	0.01328417	0.33055439
5.84543653	0.30108382	0.01433732	0.32779703
5.81088837	0.32601349	0.01481879	0.32603803
5.80561902	0.32999314	0.01434753	0.32576937
5.79677523	0.33678188	0.01403258	0.32531826
5.76632084	0.36124604	0.01444984	0.32376271
5.70993459	0.41132902	0.01582035	0.32087402
5.6742067	0.44659848	0.01654068	0.31903788
5.67012724	0.45081328	0.01610047	0.31882794
5.66944437	0.45152268	0.01556975	0.31879279
5.65126389	0.47082558	0.01569419	0.31785642
5.63561136	0.48810424	0.0157453	0.31704932
5.63429021	0.48959134	0.01529973	0.31698116
5.63062268	0.49374335	0.01496192	0.31679191
5.62412862	0.50118183	0.01474064	0.31645668
5.61879856	0.5073707	0.01449631	0.31618143
5.5890315	0.54336597	0.0150935	0.31464239
5.52345359	0.631932	0.01707924	0.31124079
5.50394516	0.66096548	0.01739383	0.31022594
5.45937734	0.73239707	0.01877941	0.30790242
5.45107225	0.7465376	0.01866344	0.30746867
5.43792054	0.76949074	0.01876807	0.30678128
5.40897899	0.82251713	0.01958374	0.30526647
5.40561602	0.82891103	0.019277	0.30509026
5.38151215	0.87621722	0.01991403	0.30382611
5.35478528	0.93183422	0.02070743	0.30242199
5.34330006	0.95680597	0.02080013	0.30181783
5.3278913	0.99136284	0.02109283	0.30100654

Do not distribute

5.3278913	0.99136284	0.02065339	0.30100654
5.32465079	0.99878762	0.02038342	0.30083582
5.29350151	1	0.02146112	0.29919285
5.28966266	1	0.02122712	0.29899013
5.27830707	1	0.02137044	0.29839017
5.26353716	1	0.02169256	0.29760913
5.20247881	1	0.02450481	0.29437215
5.19161012	1	0.02466898	0.29379456
5.18244086	1	0.02474543	0.29330696
5.17875953	1	0.02451825	0.29311111
5.1475887	1	0.02588852	0.29145088
5.14015272	1	0.02588923	0.29105432
5.08476403	1	0.02892069	0.28809423
5.07771984	1	0.02891174	0.28771699
5.06917117	1	0.02901089	0.28725895
5.06347326	1	0.02892745	0.28695351
5.05309926	1	0.02916384	0.28639711
5.03291743	1	0.03008106	0.28531357
5.03235923	1	0.02966339	0.28528359
5.01741131	1	0.0302439	0.28448009
5.01389185	1	0.03004161	0.2842908
5.00451724	1	0.03025225	0.28378636
5.00228314	1	0.02997387	0.2836661
4.9953357	1	0.03002824	0.28329202
4.98160937	1	0.03056202	0.28255241
4.97735418	1	0.03044016	0.282323
4.9671534	1	0.03074247	0.28177278
4.93758487	1	0.03246966	0.28017578
4.92781291	1	0.03277158	0.27964731
4.92614913	1	0.03247013	0.2795573
4.89387166	1	0.03452691	0.27780914
4.87181702	1	0.03586574	0.27661253
4.8564354	1	0.0366943	0.27577696
4.84806758	1	0.03694633	0.27532204
4.84597657	1	0.03667191	0.27520832
4.84217411	1	0.03654868	0.27500149
4.83876601	1	0.03639809	0.27481607
4.83812282	1	0.03602319	0.27478107
4.81734375	1	0.03734924	0.27364961
4.80234906	1	0.03821692	0.27283217
4.76577191	1	0.04110261	0.27083481
4.75965277	1	0.04121745	0.2705002
4.75673086	1	0.04103463	0.27034037
4.75673086	1	0.0405837	0.27034037

Do not distribute

4.75673086	1	0.04014258	0.27034037
4.75405746	1	0.03995614	0.27019412
4.75399238	1	0.039537	0.27019056
4.73992716	1	0.04040854	0.26942065
4.73775496	1	0.04018813	0.26930169
4.73690939	1	0.03985133	0.26925537
4.73300461	1	0.03980093	0.26904147
4.72459473	1	0.04016928	0.26858059
4.72265903	1	0.03994523	0.26847447
4.70594417	1	0.04110157	0.2675576
4.70594417	1	0.04069861	0.2675576
4.69322763	1	0.04150105	0.26685939
4.67987949	1	0.0423849	0.26612588
4.6674283	1	0.04320225	0.2654411
4.66424352	1	0.04310966	0.26526585
4.64081328	1	0.04507408	0.2639755
4.63099202	1	0.04567811	0.26343404
4.61531677	1	0.04692245	0.26256915
4.60930209	1	0.0471443	0.26223705
4.60468776	1	0.04721861	0.26198219
4.60405573	1	0.04686517	0.26194727
4.60320941	1	0.04654104	0.26190052
4.58722225	1	0.04786266	0.26101683
4.58678098	1	0.0474947	0.26099243
4.5835502	1	0.04743684	0.26081374
4.56795269	1	0.04875121	0.25995052
4.56669017	1	0.04847879	0.25988061
4.55400979	1	0.04949567	0.25917815
4.54159869	1	0.05050612	0.25849005
4.541111	1	0.05014499	0.258463
4.52165158	1	0.05201308	0.25738301
4.52029759	1	0.0517513	0.25730781
4.51575227	1	0.05187403	0.25705534
4.49834322	1	0.05356372	0.25608765
4.49612053	1	0.05341127	0.25596402
4.49007487	1	0.05373353	0.25562767
4.48638812	1	0.05376824	0.2554225
4.47811832	1	0.05437708	0.2549621
4.47139717	1	0.05480036	0.25458774
4.46966587	1	0.05459926	0.25449128
4.46806968	1	0.05438515	0.25440234
4.45318691	1	0.055858	0.25357264
4.45254066	1	0.05552371	0.2535366
4.4481103	1	0.05567752	0.25328945

Do not distribute

4.4398836	1	0.05632503	0.25283034
4.43675609	1	0.05631801	0.25265574
4.4192704	1	0.0582069	0.25167893
4.41818229	1	0.05793311	0.25161811
4.41751987	1	0.0576071	0.25158108
4.41679755	1	0.05729375	0.2515407
4.39971191	1	0.05917301	0.25058506
4.38991512	1	0.06009977	0.25003663
4.37729928	1	0.06144155	0.24932991
4.37605103	1	0.06119345	0.24925995
4.37452986	1	0.06098756	0.24917469
4.36715492	1	0.06161007	0.24876122
4.35814394	1	0.06247673	0.24825577
4.34626094	1	0.06377886	0.24758879
4.34555559	1	0.06345664	0.24754918
4.34282531	1	0.06343394	0.24739586
4.34113193	1	0.0632628	0.24730075
4.32752027	1	0.06485034	0.24653589
4.3263188	1	0.06460772	0.24646834
4.32505003	1	0.0643787	0.24639701
4.3232961	1	0.06422487	0.24629839
4.31584028	1	0.06492082	0.24587905
4.31277741	1	0.0649665	0.24570673
4.2999249	1	0.06649698	0.24498327
4.2839727	1	0.06855376	0.24408452
4.2837661	1	0.06816037	0.24407288
4.2736956	1	0.06932874	0.24350504
4.26643635	1	0.07006482	0.24309551
4.26643635	1	0.06963759	0.24309551
4.26643635	1	0.06921555	0.24309551
4.26643635	1	0.06879858	0.24309551
4.26643635	1	0.06838662	0.24309551
4.26512065	1	0.06818581	0.24302126
4.26240891	1	0.0682069	0.24286821
4.25994215	1	0.06819191	0.24272897
4.25799315	1	0.06809805	0.24261894
4.25791852	1	0.06771377	0.24261473
4.25655741	1	0.06753368	0.24253788
4.2463369	1	0.06874447	0.24196061
4.2310217	1	0.07080504	0.24109491
4.22938484	1	0.07066859	0.24100234
4.22757155	1	0.07056334	0.24089978
4.22584964	1	0.07044567	0.24080238
4.22549075	1	0.07011003	0.24078207

Do not distribute

4.2253315	1	0.0697461	0.24077306
4.20866217	1	0.07207476	0.23982956
4.20533819	1	0.07222946	0.23964131
4.18473553	1	0.07532468	0.2384736
4.18126919	1	0.07551564	0.238277
4.1762219	1	0.07598543	0.23799065
4.17573875	1	0.07566103	0.23796323
4.17128492	1	0.07603218	0.23771047
4.17128492	1	0.07562775	0.23771047
4.17128492	1	0.0752276	0.23771047
4.17128492	1	0.07483167	0.23771047
4.1562258	1	0.07706635	0.23685532
4.15558402	1	0.07677834	0.23681886
4.15363635	1	0.07672383	0.2367082
4.15363635	1	0.07632835	0.2367082
4.1529962	1	0.07604893	0.23667182
4.15275511	1	0.07570294	0.23665812
4.1461075	1	0.07648041	0.2362803
4.13750295	1	0.07761681	0.23579103
4.13666666	1	0.07737563	0.23574347
4.13448394	1	0.07737666	0.23561931
4.13357541	1	0.07715294	0.23556762
4.1325703	1	0.07694887	0.23551044
4.12733516	1	0.0774984	0.23521254
4.11425077	1	0.07947728	0.23446759
4.11412166	1	0.0791131	0.23446024
4.10416478	1	0.0805549	0.23389295
4.10200121	1	0.08056611	0.23376963
4.10163286	1	0.0802468	0.23374863
4.10125161	1	0.07993299	0.2337269
4.0970274	1	0.0803299	0.23348608
4.09614932	1	0.080111	0.23343602
4.09577297	1	0.07980225	0.23341456
4.09454852	1	0.07965184	0.23334474
4.09294723	1	0.07957249	0.23325342
4.09142135	1	0.07948115	0.23316639
4.07724661	1	0.08173792	0.23235757
4.07070713	1	0.08259563	0.2319842
4.05540459	1	0.08516533	0.2311099
4.04381991	1	0.08706827	0.23044749
4.03735782	1	0.08797179	0.23007779
4.0360277	1	0.08784235	0.23000167
4.03521871	1	0.08760971	0.22995538
4.02998599	1	0.08827406	0.22965586

Do not distribute

4.02998599	1	0.08787998	0.22965586
4.02998599	1	0.0874894	0.22965586
4.02998599	1	0.08710228	0.22965586
4.02998599	1	0.08671857	0.22965586
4.02998599	1	0.08633822	0.22965586
4.02647676	1	0.08665861	0.22945495
4.02330271	1	0.08691473	0.22927319
4.02136304	1	0.08692585	0.22916209
4.01742914	1	0.08733872	0.22893675
4.01266975	1	0.08792214	0.22866404
4.00897554	1	0.08829428	0.22845231
4.00655929	1	0.08840906	0.2283138
4.00113281	1	0.08914133	0.22800266
5.73382574	0.39263104	0.39263104	0.23845369
5.4158406	0.81652552	0.40826276	0.22553123
5.01114362	1	0.69110731	0.20881637
4.74857247	1	0.94880705	0.19781289
4.71451709	1	0.82096241	0.19637672
4.71272021	1	0.6869718	0.19630088
4.70616799	1	0.59778407	0.1960243
4.46639894	1	0.90849269	0.1858514
4.42222441	1	0.8940122	0.18396622
4.3997066	1	0.84742987	0.18300396
4.36584804	1	0.83285546	0.18155543
4.33344991	1	0.82258202	0.18016753
4.32132598	1	0.78080221	0.1796477
4.28224023	1	0.79330849	0.17797012
4.27860432	1	0.74664607	0.17781393
4.21349927	1	0.81318829	0.17501345
4.1663381	1	0.85314597	0.17298036
4.14921329	1	0.83815544	0.17224119
4.14669329	1	0.79866284	0.17213238
4.13334109	1	0.78241881	0.17155566
4.12091606	1	0.76678748	0.17101872
4.09589232	1	0.77534566	0.16993656
4.09516844	1	0.74287217	0.16990524
4.07053072	1	0.75347422	0.16883874
4.06153527	1	0.73847379	0.1684491
4.05352057	1	0.72329664	0.16810183
4.02123596	1	0.75025802	0.16670189
4.01243115	1	0.73828014	0.1663198
6.29241593	0.10849118	0.10849118	0.24322973
5.81872084	0.32291597	0.16145798	0.225464
5.79867795	0.33816793	0.11272264	0.2247044

Do not distribute

5.31252337	1	0.25895661	0.20608458
5.31157242	1	0.20761941	0.2060478
5.30735289	1	0.17470536	0.20588455
5.27680817	1	0.16065866	0.204702
5.27680817	1	0.14057633	0.204702
5.09491651	1	0.18995527	0.19762988
4.97407551	1	0.22580568	0.19290313
4.97393631	1	0.20534369	0.19289767
4.87100089	1	0.23857681	0.18885359
4.76975989	1	0.2780399	0.18486034
4.50502991	1	0.47495495	0.17434575
4.45844781	1	0.49348198	0.17248485
4.43067604	1	0.49319009	0.17137388
4.35979278	1	0.54647295	0.16853323
4.29237245	1	0.60278913	0.16582463
4.24188821	1	0.64145849	0.16379217
4.23509808	1	0.61898808	0.16351853
4.20136072	1	0.63713336	0.16215794
4.14483022	1	0.69271958	0.15987455
4.13726285	1	0.67424802	0.15956855
4.13087648	1	0.65572636	0.15931024
4.12340984	1	0.64041355	0.15900817
4.07958506	1	0.68116443	0.15723361
4.07149323	1	0.66827217	0.15690567
4.04680458	1	0.68209953	0.15590453
4.02576762	1	0.69126528	0.15505081
4.02170682	1	0.67450053	0.15488594
4.67514188	1	1	0.26979872
4.49631476	1	1	0.25978906
4.29942122	1	1	0.24863371
4.28446787	1	1	0.24778077
4.22358159	1	1	0.24429949
4.22358159	1	1	0.24429949
4.217538	1	1	0.24395321
4.08298006	1	1	0.23620948

Do not distribute

Supp. Table S12. List of markers with annotations from gene models following the Rice G

**Trait**	**CONTIG, POS, -LOGP**	**GENE MODELS**
BG02_drought_LRS_1124	(chr03, 23619984, 4.28)	LOC_Os03g42440 chr03 [23616737-23620260] -expressed protein (Os03g0622300, OsNippo03g242100)
BG02_drought_LRS_1124	(chr08, 5986608, 4.92); (chr08, 5987333, 4.18); (chr08, 5987387, 4.98); (chr08, 5988046, 4.65)	LOC_Os08g10290 chr08 [5980927-5988963] -SHR5-receptor-like kinase, putative, expressed (Os08g0203100, Os08g0203201, OsNippo08g069450)
BG02_drought_LRS_1124	(chr08, 6020882, 4.69)	LOC_Os08g10310 chr08 [6015744-6023280] -SHR5-receptor-like kinase, putative, expressed (Os08g0203400, OsNippo08g069750)
BG02_drought_LRS_1124	(chr08, 6032998, 5.35); (chr08, 6037175, 4.61); (chr08, 6038045, 6.95); (chr08, 6038286, 4.51);	LOC_Os08g10320 chr08 [6032170-6039181] -SHR5-receptor-like kinase, putative, expressed (Os08g0203600, OsNippo08g069850)
BG02_drought_LRS_1124	(chr08, 6046760, 4.7); (chr08, 6047726, 4.62); (chr08, 6048076, 4.56)	LOC_Os08g10330 chr08 [6040525-6048616] -SHR5-receptor-like kinase, putative, expressed (Os08g0203700, OsNippo08g069950)
BG02_drought_LRS_1124	(chr08, 6077482, 5.72)	LOC_Os08g10360 chr08 [6071547-6078861] -retrotransposon protein, putative, unclassified, expressed (OsNippo08g070150)
BG02_drought_LRS_1124	(chr08, 6085691, 6.26)	LOC_Os08g10380 chr08 [6085434-6086181] -expressed protein (OsNippo08g070250)
BG02_drought_LRS_1124	(chr08, 6098380, 4.64)	LOC_Os08g10390 chr08 [6098375-6100816] -retrotransposon protein, putative, unclassified (OsNippo08g070300)
BG02_drought_LRS_1124	(chr08, 6113574, 4.99)	LOC_Os08g10410 chr08 [6112374-6115664] -expressed protein (Os08g0204800, OsNippo08g070400)

Do not distribute

LRI_2010DSstress_322010	(chr01, 7746251, 4.06)	LOC_Os01g13810 chr01 [7744242-7747746] -expressed protein (Os01g0239800, OsNippo01g090500)
LRI_2010DSstress_322010	(chr01, 23808242, 4.02)	LOC_Os01g41980 chr01 [23806123-23808853] -retrotransposon protein, putative, unclassified, expressed (OsNippo01g243900)
LRI_2010DSstress_322010	(chr02, 32448418, 4.42)	LOC_Os02g53020 chr02 [32447892-32451281] -transposon protein, putative, CACTA, En/Spm sub-class, expressed (OsNippo02g325050)
LRI_2010DSstress_322010	(chr05, 3184683, 4.34)	LOC_Os05g06260 chr05 [3181565-3188216] -Spc97 / Spc98 family protein, putative, expressed (Os05g0154500, OsNippo05g043300)
LRI_2010DSstress_322010	(chr05, 3203589, 4.15); (chr05, 3203830, 4.08)	LOC_Os05g06280 chr05 [3203041-3214116] -ATKINESIN-13A/KINESIN-13A, putative, expressed (Os05g0154700, Os05g0154800, OsNippo05g043400)
LRI_2010DSstress_322010	(chr05, 3244717, 4.5)	LOC_Os05g06330 chr05 [3241298-3245201] -nifU, putative, expressed (Os05g0155300, OsNippo05g043800)
LRI_2010DSstress_322010	(chr05, 3332658, 4.5); (chr05, 3332754, 4.5)	LOC_Os05g06470 chr05 [3323281-3334849] -suppressor of Mek, putative, expressed (Os05g0156800, OsNippo05g044750)
LRI_2010DSstress_322010	(chr05, 3343234, 4.5)	LOC_Os05g06490 chr05 [3342053-3344599] -RNA polymerase Rpb7, N-terminal domain containing protein, expressed (Os05g0157100, OsNippo05g044900)
LRI_2010DSstress_322010	(chr05, 20479253, 4.62)	LOC_Os05g34540 chr05 [20477180-20483634] -rab GDP dissociation inhibitor alpha, putative, expressed (Os05g0418000, OsNippo05g201100)

Do not distribute

LRI_2010DSstress_322010	(chr11, 11268061, 4.08)	LOC_Os11g19550 chr11 [11265058-11268700] -transposon protein, putative, CACTA, En/Spm sub-class, expressed (OsNippo11g117250)
LRI_2012DS9M_39	(chr01, 15686399, 4.29)	LOC_Os01g28040 chr01 [15681230-15686522] -transposon protein, putative, unclassified, expressed (OsNippo01g167650)
LRI_2012DS9M_39	(chr03, 32630631, 5.32); (chr03, 32630704, 5.57)	LOC_Os03g57220 chr03 [32628527-32632535] -hydroxyacid oxidase 1, putative, expressed (Os03g0786100, OsNippo03g328850)
LRI_2012DS9M_39	(chr03, 32689107, 4.34)	LOC_Os03g57300 chr03 [32679833-32691070] -expressed protein (Os03g0786900, OsNippo03g329300)
LRI_2012DS9M_39	(chr03, 35871240, 5.05)	LOC_Os03g63500 chr03 [35868697-35875491] -ATPase, AFG1 family domain containing protein, expressed (Os03g0851900, OsNippo03g366200)
LRI_2012DS9M_39	(chr03, 35882893, 4.91)	LOC_Os03g63510 chr03 [35877541-35882905] -pentatricopeptide, putative, expressed (Os03g0852000, OsNippo03g366150)
LRI_2012DS9M_39	(chr03, 35890725, 5.39)	LOC_Os03g63530 chr03 [35890201-35894181] -histone-like transcription factor and archaeal histone, putative, expressed (Os03g0852300, OsNippo03g366350)
LRI_2012DS9M_39	(chr03, 35902166, 4.65)	LOC_Os03g63560 chr03 [35900685-35903066] -pentatricopeptide, putative, expressed (Os03g0852700, OsNippo03g366550)
LRI_2012DS9M_39	(chr04, 1861931, 4.94)	LOC_Os04g04020 chr04 [1857914-1865238] -protein transport protein Sec24-like, putative, expressed (Os04g0129500, OsNippo04g027750)

Do not distribute

LRI_2012DS9M_39	(chr04, 1883093, 4.14)	LOC_Os04g04080 chr04 [1882946-1884718] -transposon protein, putative, CACTA, En/Spm sub-class, expressed (OsNippo04g028100)
LRI_2012DS9M_39	(chr04, 1945526, 5.12)	LOC_Os04g04180 chr04 [1943611-1948016] -transposon protein, putative, CACTA, En/Spm sub-class, expressed (OsNippo04g028600)
LRI_2012DS9M_39	(chr04, 1996762, 4.24)	LOC_Os04g04254 chr04 [1985633-1997948] -sterol 3-beta-glucosyltransferase, putative, expressed (Os04g0131900, OsNippo04g029000)
LRI_2012DS9M_39	(chr07, 16112457, 4.69)	LOC_Os07g27590 chr07 [16111827-16112848] -retrotransposon protein, putative, unclassified, expressed (OsNippo07g158950)
LRI_2012DS9M_39	(chr07, 16899577, 4.16)	LOC_Os07g28850 chr07 [16893629-16900205] -retrotransposon protein, putative, unclassified, expressed (Os07g0471300, OsNippo07g166050)
LRI_2012DS9M_39	(chr07, 23738858, 4.81)	LOC_Os07g39610 chr07 [23737765-23740989] -transposon protein, putative, CACTA, En/Spm sub-class, expressed (OsNippo07g233800)
LRI_2012DS9M_39	(chr08, 11946223, 4.15)	LOC_Os08g19960 chr08 [11946209-11946506] -expressed protein (OsNippo08g125450)
LRI_2012DS9M_39	(chr08, 26435118, 4)	LOC_Os08g41860 chr08 [26434674-26435130] -expressed protein (OsNippo08g258500)
LRI_2012DS9M_39	(chr09, 22450668, 4.12)	LOC_Os09g39110 chr09 [22450379-22453266] -vignain precursor, putative, expressed (Os09g0564600, OsNippo09g222200)

Do not distribute

LRI_2012DS9M_39	(chr10, 8321472, 4.15)	LOC_Os10g16690 chr10 [8320661-8323519] -retrotransposon protein, putative, unclassified, expressed (OsNippo10g088650)
LRI_2012DS9M_39	(chr10, 19073118, 4.03)	LOC_Os10g35660 chr10 [19071211-19073156] -MYB family transcription factor, putative, expressed (OsNippo10g193800)
LRI_2012DS9M_39	(chr11, 8036341, 4.1)	LOC_Os11g14320 chr11 [8035250-8037227] -expressed protein (OsNippo11g090800)
LRI_2012DS9M_39	(chr11, 8064728, 4.65)	LOC_Os11g14360 chr11 [8063067-8065101] -retrotransposon, putative, centromere-specific (Os11g0248732, OsNippo11g091000)
LRI_2012DS9M_39	(chr11, 8099054, 4.9)	LOC_Os11g14420 chr11 [8095868-8099597] -inactive receptor kinase At1g27190 precursor, putative, expressed (Os11g0249900, OsNippo11g091350)
LRI_2012DS9M_39	(chr11, 8181529, 4.9)	LOC_Os11g14560 chr11 [8176413-8187419] -transposon protein, putative, CACTA, En/Spm sub-class, expressed (OsNippo11g092100)
LRI_2012DS9M_39	(chr11, 8200706, 4.9)	LOC_Os11g14570 chr11 [8198056-8201966] -expressed protein (Os11g0252400, OsNippo11g092250)
LRI_2012DS9M_39	(chr11, 14200265, 4.78)	LOC_Os11g24930 chr11 [14194532-14200495] -retrotransposon protein, putative, Ty3-gypsy subclass, expressed (OsNippo11g142300)
LRI_2012DS9M_39	(chr12, 13763710, 4.07)	LOC_Os12g24170 chr12 [13761533-13782378] -beta-galactosidase precursor, putative, expressed (Os12g0429200, Os12g0429300, OsNippo12g141350)

Do not distribute

LRI_2012DS9M_39	(chr12, 13977979, 4.29)	LOC_Os12g24480 chr12 [13977580-13979961] -expressed protein (OsNippo12g142900)
LRI_2012DS9M_410	(chr01, 310101, 4.69)	LOC_Os01g01620 chr01 [309458-313170] -kinase, pfkB family, putative, expressed (Os01g0105900, OsNippo01g013900)
LRI_2012DS9M_410	(chr01, 383275, 4.84)	LOC_Os01g01720 chr01 [383046-386701] -pex14, putative, expressed (Os01g0107000, OsNippo01g014400)
LRI_2012DS9M_410	(chr01, 2971212, 4.42)	LOC_Os01g06270 chr01 [2971124-2979313] -expressed protein (Os01g0155400, OsNippo01g044600)
LRI_2012DS9M_410	(chr01, 4715543, 4.77)	LOC_Os01g09280 chr01 [4714467-4718746] -myb-related transcription activator, putative, expressed (Os01g0187900, OsNippo01g063300)
LRI_2012DS9M_410	(chr01, 15136757, 4.23)	LOC_Os01g27150 chr01 [15130856-15139604] -cullin, putative, expressed (Os01g0369000, OsNippo01g162300)
LRI_2012DS9M_410	(chr01, 20360570, 4.15)	LOC_Os01g36670 chr01 [20351523-20364454] -expressed protein (Os01g0547200, OsNippo01g211350)
LRI_2012DS9M_410	(chr01, 25480518, 4.11)	LOC_Os01g44430 chr01 [25476743-25482973] -retrotransposon protein, putative, unclassified, expressed (Os01g0635550, Os01g0635600, OsNippo01g259800)
LRI_2012DS9M_410	(chr01, 37874833, 4.59)	LOC_Os01g65270 chr01 [37874455-37875289] -retrotransposon protein, putative, unclassified (OsNippo01g391100)

Do not distribute

LRI_2012DS9M_410	(chr02, 969014, 4.82)	LOC_Os02g02640 chr02 [967171-974497] -NBS-LRR disease resistance protein, putative, expressed (OsNippo02g020400)
LRI_2012DS9M_410	(chr02, 6070091, 4.21); (chr02, 6074812, 4.17)	LOC_Os02g11760 chr02 [6069030-6075807] -pleiotropic drug resistance protein, putative, expressed (Os02g0208300, OsNippo02g073350)
LRI_2012DS9M_410	(chr02, 6111391, 4.17)	LOC_Os02g11820 chr02 [6110091-6118738] -GTPase-activating protein, putative, expressed (Os02g0208900, OsNippo02g073800)
LRI_2012DS9M_410	(chr02, 20543258, 4.6)	LOC_Os02g34330 chr02 [20542156-20543511] -retrotransposon protein, putative, unclassified, expressed (OsNippo02g204500)
LRI_2012DS9M_410	(chr02, 33430148, 4.18)	LOC_Os02g54580 chr02 [33429948-33433753] -serine esterase family protein, putative, expressed (Os02g0787100, OsNippo02g335450)
LRI_2012DS9M_410	(chr03, 471584, 5.63)	LOC_Os03g01750 chr03 [471452-476739] -dual specificity protein phosphatase, putative, expressed (Os03g0107800, OsNippo03g015550)
LRI_2012DS9M_410	(chr03, 486462, 5.38)	LOC_Os03g01780 chr03 [485270-486556] -transposon protein, putative, unclassified, expressed (OsNippo03g015750)
LRI_2012DS9M_410	(chr03, 1233440, 4.15)	LOC_Os03g03020 chr03 [1232213-1234476] -L11 domain containing ribosomal protein, putative, expressed (Os03g0122200, OsNippo03g023600)
LRI_2012DS9M_410	(chr03, 3188862, 4.1)	LOC_Os03g06370 chr03 [3186738-3190051] -PPR repeat domain containing protein, putative, expressed (Os03g0159700, OsNippo03g044450)

Do not distribute

LRI_2012DS9M_410	(chr03, 3262953, 4.03); (chr03, 3263781, 4.44); (chr03, 3266262, 4.63); (chr03, 3266346, 4.04);	LOC_Os03g06510 chr03 [3258436-3270596] -KIP1, putative, expressed (Os03g0161100, OsNippo03g045150)
LRI_2012DS9M_410	(chr03, 6116019, 4.32)	LOC_Os03g11690 chr03 [6115337-6118762] -PPR repeat containing protein, expressed (Os03g0216300, OsNippo03g075450)
LRI_2012DS9M_410	(chr03, 11353842, 5.35)	LOC_Os03g20100 chr03 [11351430-11355773] -S1 RNA binding domain containing protein, expressed (Os03g0315800, OsNippo03g128250)
LRI_2012DS9M_410	(chr04, 1861931, 4.07)	LOC_Os04g04020 chr04 [1857914-1865238] -protein transport protein Sec24-like, putative, expressed (Os04g0129500, OsNippo04g027750)
LRI_2012DS9M_410	(chr04, 3682785, 4.48)	LOC_Os04g06990 chr04 [3682117-3684160] -expressed protein (Os04g0154000, OsNippo04g043700)
LRI_2012DS9M_410	(chr04, 13495358, 4.47)	LOC_Os04g23580 chr04 [13493210-13498065] -xylosyltransferase, putative, expressed (Os04g0301700, OsNippo04g136150)
LRI_2012DS9M_410	(chr04, 18071809, 5.01)	LOC_Os04g30270 chr04 [18069356-18072567] -protein kinase domain containing protein, expressed (Os04g0371200, OsNippo04g173500)
LRI_2012DS9M_410	(chr04, 21949796, 4.04)	LOC_Os04g36000 chr04 [21948795-21951830] -OsFBX137 - F-box domain containing protein, expressed (Os04g0440901, OsNippo04g208600)
LRI_2012DS9M_410	(chr04, 29249317, 4.15)	LOC_Os04g49060 chr04 [29247373-29249631] -H-BTB3 - Bric-a-Brac, Tramtrack, Broad Complex BTB domain with H family conserved sequence, expressed (Os04g0579700, OsNippo04g286000)

Do not distribute

LRI_2012DS9M_410	(chr05, 658350, 4.76); (chr05, 658473, 4.76)	LOC_Os05g02150 chr05 [658105-660255] -expressed protein (Os05g0112200, OsNippo05g017450)
LRI_2012DS9M_410	(chr05, 696106, 5.03)	LOC_Os05g02210 chr05 [694388-696374] -pentatricopeptide, putative, expressed (Os05g0112900, OsNippo05g017750)
LRI_2012DS9M_410	(chr05, 721301, 4.37)	LOC_Os05g02260 chr05 [719131-721868] -expressed protein (Os05g0113500, OsNippo05g018050)
LRI_2012DS9M_410	(chr05, 738505, 4.55)	LOC_Os05g02300 chr05 [738203-739079] -Core histone H2A/H2B/H3/H4 domain containing protein, putative, expressed (Os05g0113900, OsNippo05g018250)
LRI_2012DS9M_410	(chr05, 760007, 5.67); (chr05, 762163, 4.86)	LOC_Os05g02350 chr05 [759007-763806] -endonuclease/exonuclease/phosphatase family domain containing protein, expressed (Os05g0114100, OsNippo05g018600)
LRI_2012DS9M_410	(chr05, 8419688, 4.45)	LOC_Os05g14810 chr05 [8418033-8419930] -retrotransposon protein, putative, Ty1-copia subclass (OsNippo05g090300)
LRI_2012DS9M_410	(chr05, 10306394, 4.49)	LOC_Os05g17920 chr05 [10305339-10306577] -retrotransposon protein, putative, unclassified (OsNippo05g108200)
LRI_2012DS9M_410	(chr05, 15810943, 4.52)	LOC_Os05g27170 chr05 [15807141-15811008] -retrotransposon, putative, centromere-specific, expressed (OsNippo05g158000)
LRI_2012DS9M_410	(chr05, 16305862, 4.52)	LOC_Os05g27940 chr05 [16302688-16306135] -40S ribosomal protein S7, putative, expressed (Os05g0346300, OsNippo05g162500)

Do not distribute

LRI_2012DS9M_410	(chr05, 21654298, 4.21)	LOC_Os05g37060 chr05 [21654181-21655380] -MYB family transcription factor, putative, expressed (Os05g0442400, OsNippo05g213850)
LRI_2012DS9M_410	(chr05, 24595524, 4.98)	LOC_Os05g42020 chr05 [24594917-24597258] -UDP-glucoronosyl and UDP-glucosyl transferase domain containing protein, expressed (Os05g0499600, OsNippo05g245250)
LRI_2012DS9M_410	(chr06, 5291712, 5.15)	LOC_Os06g10290 chr06 [5288460-5293460] -OsFBX191 - F-box domain containing protein, expressed (Os06g0204500, OsNippo06g069900)
LRI_2012DS9M_410	(chr06, 8586898, 4.28)	LOC_Os06g15150 chr06 [8584737-8590595] -retrotransposon protein, putative, Ty3-gypsy subclass, expressed (OsNippo06g100500)
LRI_2012DS9M_410	(chr06, 13490062, 4.16)	LOC_Os06g23130 chr06 [13488226-13490947] -retrotransposon protein, putative, unclassified, expressed (OsNippo06g147650)
LRI_2012DS9M_410	(chr06, 18622786, 6.25)	LOC_Os06g32030 chr06 [18622233-18623038] -transposon protein, putative, CACTA, En/Spm sub-class (OsNippo06g193800)
LRI_2012DS9M_410	(chr07, 5265335, 4.93)	LOC_Os07g09900 chr07 [5263408-5267310] -disease resistance protein RPM1, putative, expressed (Os07g0197300, OsNippo07g063750)
LRI_2012DS9M_410	(chr07, 5411421, 5.05)	LOC_Os07g10100 chr07 [5410900-5412979] -retrotransposon protein, putative, Ty3-gypsy subclass, expressed (OsNippo07g064950)
LRI_2012DS9M_410	(chr07, 8527193, 4.72)	LOC_Os07g14900 chr07 [8526649-8528020] -expressed protein (Os07g0252900, OsNippo07g094250)

Do not distribute

LRI_2012DS9M_410	(chr07, 10839192, 5.67)	LOC_Os07g18290 chr07 [10837668-10839449] -transposon protein, putative, unclassified, expressed (OsNippo07g113800)
LRI_2012DS9M_410	(chr07, 10879822, 5.59)	LOC_Os07g18360 chr07 [10878501-10883369] -retrotransposon protein, putative, Ty3-gypsy subclass, expressed (OsNippo07g114250)
LRI_2012DS9M_410	(chr07, 15409295, 4.27)	LOC_Os07g26690 chr07 [15406441-15409801] -aquaporin protein, putative, expressed (Os07g0448800, OsNippo07g153350)
LRI_2012DS9M_410	(chr07, 15627486, 4.27); (chr07, 15631183, 4.59)	LOC_Os07g27000 chr07 [15626794-15631685] -retrotransposon protein, putative, unclassified, expressed (OsNippo07g155200)
LRI_2012DS9M_410	(chr07, 15647758, 4.44)	LOC_Os07g27010 chr07 [15644781-15647996] -retrotransposon protein, putative, unclassified, expressed (OsNippo07g155300)
LRI_2012DS9M_410	(chr08, 3688061, 4.09)	LOC_Os08g06500 chr08 [3680924-3688728] -PPR repeat domain containing protein, putative, expressed (Os08g0162250, OsNippo08g046000)
LRI_2012DS9M_410	(chr08, 4079057, 4.72)	LOC_Os08g07300 chr08 [4078664-4088954] -haloacid dehalogenase-like hydrolase domain-containing protein 3, putative, expressed (Os08g0169800, OsNippo08g050700)
LRI_2012DS9M_410	(chr08, 5621513, 4.76)	LOC_Os08g09720 chr08 [5618540-5621555] -OsFBX272 - F-box domain containing protein, expressed (Os08g0197100, OsNippo08g065800)
LRI_2012DS9M_410	(chr08, 6086162, 5.06)	LOC_Os08g10380 chr08 [6085434-6086181] -expressed protein (OsNippo08g070250)

Do not distribute

LRI_2012DS9M_410	(chr08, 7295368, 5.18)	LOC_Os08g12380 chr08 [7294102-7295938] -retrotransposon protein, putative, Ty3-gypsy subclass, expressed (OsNippo08g082200)
LRI_2012DS9M_410	(chr08, 11870523, 4.27)	LOC_Os08g19830 chr08 [11869979-11879530] -ubiquitin family protein, putative, expressed (Os08g0295000, Os08g0295100, OsNippo08g124700)
LRI_2012DS9M_410	(chr08, 12056095, 4.35)	LOC_Os08g20110 chr08 [12052929-12057408] -retrotransposon protein, putative, Ty3-gypsy subclass, expressed (OsNippo08g126300)
LRI_2012DS9M_410	(chr08, 12259198, 5.32)	LOC_Os08g20410 chr08 [12256989-12259738] -expressed protein (Os08g0299300, OsNippo08g128250)
LRI_2012DS9M_410	(chr08, 16806657, 5.26)	LOC_Os08g27570 chr08 [16804845-16808858] -retrotransposon protein, putative, unclassified, expressed (OsNippo08g169500)
LRI_2012DS9M_410	(chr08, 17848630, 7.03)	LOC_Os08g29150 chr08 [17844188-17850916] -phospholipid-transporting ATPase, putative, expressed (Os08g0379200, OsNippo08g178550)
LRI_2012DS9M_410	(chr08, 24774725, 4.09)	LOC_Os08g39220 chr08 [24772960-24775745] -OsWAK75 - OsWAK receptor-like protein kinase, expressed (Os08g0501600, OsNippo08g242200)
LRI_2012DS9M_410	(chr08, 26921142, 4.58)	LOC_Os08g42590 chr08 [26918193-26921575] -mtN19, putative, expressed (Os08g0538600, OsNippo08g263000)
LRI_2012DS9M_410	(chr08, 26950444, 5.33)	LOC_Os08g42640 chr08 [26948571-26953515] -zinc finger, C3HC4 type domain containing protein, expressed (Os08g0539300, OsNippo08g263300)

Do not distribute

LRI_2012DS9M_410	(chr09, 7953057, 4.98)	LOC_Os09g13640 chr09 [7952822-7953923] -retrotransposon protein, putative, unclassified (OsNippo09g081200)
LRI_2012DS9M_410	(chr09, 8201834, 4.47)	LOC_Os09g13940 chr09 [8199883-8203350] -AP2 domain containing protein, expressed (Os09g0309700, OsNippo09g083000)
LRI_2012DS9M_410	(chr09, 10043165, 4.75)	LOC_Os09g16430 chr09 [10043019-10049365] -retrotransposon protein, putative, Ty3-gypsy subclass, expressed (OsNippo09g098100)
LRI_2012DS9M_410	(chr09, 16264548, 4.38)	LOC_Os09g26770 chr09 [16259677-16265914] -ribosomal L18p/L5e family protein, putative, expressed (Os09g0439050, Os09g0439100, OsNippo09g155850)
LRI_2012DS9M_410	(chr09, 17802319, 5.64)	LOC_Os09g29330 chr09 [17799761-17805714] -retrotransposon protein, putative, unclassified, expressed (OsNippo09g171750)
LRI_2012DS9M_410	(chr09, 19023408, 4.25)	LOC_Os09g31514 chr09 [19020419-19024392] -dihydroflavonol-4-reductase, putative, expressed (Os09g0491852, OsNippo09g184150)
LRI_2012DS9M_410	(chr09, 22716410, 7.6)	LOC_Os09g39570 chr09 [22713279-22717110] -beta-amylase, putative, expressed (Os09g0569200, OsNippo09g225350)
LRI_2012DS9M_410	(chr09, 22716410, 7.6)	LOC_Os09g39580 chr09 [22716053-22719930] -calmodulin-binding heat-shock protein, putative, expressed (Os09g0569300, OsNippo09g225450)
LRI_2012DS9M_410	(chr09, 22739992, 7.95)	LOC_Os09g39620 chr09 [22736558-22741162] -protein kinase family protein, putative, expressed (Os09g0569800, OsNippo09g225800)

Do not distribute

LRI_2012DS9M_410	(chr09, 22742406, 4.84)	LOC_Os09g39630 chr09 [22742143-22743177] -conserved hypothetical protein (OsNippo09g225900)
LRI_2012DS9M_410	(chr09, 22747650, 8.61); (chr09, 22749370, 7.6)	LOC_Os09g39640 chr09 [22747356-22752211] -protein kinase, putative, expressed (Os09g0570000, OsNippo09g225950)
LRI_2012DS9M_410	(chr09, 22755848, 7.6); (chr09, 22756359, 7.6)	LOC_Os09g39650 chr09 [22753303-22757508] -protein kinase family protein, putative, expressed (Os09g0570100, OsNippo09g226000)
LRI_2012DS9M_410	(chr09, 22760515, 5.29)	LOC_Os09g39660 chr09 [22758930-22762537] -ZOS9-21 - C2H2 zinc finger protein, expressed (Os09g0570200, OsNippo09g226100)
LRI_2012DS9M_410	(chr09, 22764907, 7.68)	LOC_Os09g39670 chr09 [22762864-22766969] -oxidoreductase, short chain dehydrogenase/reductase family domain containing family, expressed (Os09g0570300, OsNippo09g226150)
LRI_2012DS9M_410	(chr09, 22776277, 5.77)	LOC_Os09g39690 chr09 [22773514-22777592] -protein binding protein, putative, expressed (Os09g0570500, OsNippo09g226250)
LRI_2012DS9M_410	(chr09, 22782443, 7.6)	LOC_Os09g39700 chr09 [22778757-22783735] -expressed protein (Os09g0570600, OsNippo09g226300)
LRI_2012DS9M_410	(chr09, 22804780, 5.67)	LOC_Os09g39760 chr09 [22804667-22807049] -pectinesterase, putative, expressed (Os09g0571100, OsNippo09g226700)
LRI_2012DS9M_410	(chr10, 526829, 4.33)	LOC_Os10g01800 chr10 [526146-527331] -transferase family protein, putative, expressed (Os10g0107400, OsNippo10g014950)

Do not distribute

LRI_2012DS9M_410	(chr10, 1352841, 5.62)	LOC_Os10g03200 chr10 [1352639-1353952] -retrotransposon protein, putative, unclassified (OsNippo10g023100)
LRI_2012DS9M_410	(chr10, 2481353, 4.49)	LOC_Os10g05069 chr10 [2477205-2489779] -lysosomal alpha-mannosidase precursor, putative, expressed (Os10g0140200, OsNippo10g033950)
LRI_2012DS9M_410	(chr10, 8776815, 4.09)	LOC_Os10g17410 chr10 [8775159-8779157] -retrotransposon protein, putative, unclassified, expressed (OsNippo10g093100)
LRI_2012DS9M_410	(chr11, 885294, 5.33)	LOC_Os11g02720 chr11 [882109-885904] -expressed protein (Os11g0119900, OsNippo11g021300)
LRI_2012DS9M_410	(chr11, 8468184, 5.81)	LOC_Os11g15040 chr11 [8466831-8469354] -S-adenosyl-L-methionine:benzoic acid/salicylic acid carboxyl methyltransferase, putative, expressed (OsNippo11g094750)
LRI_2012DS9M_410	(chr11, 10436921, 4.64)	LOC_Os11g18510 chr11 [10435429-10438555] -transposon protein, putative, CACTA, En/Spm sub-class, expressed (OsNippo11g111150)
LRI_2012DS9M_410	(chr11, 18129756, 4.85)	LOC_Os11g31140 chr11 [18128029-18129811] -expressed protein (OsNippo11g176150)
LRI_2012DS9M_410	(chr11, 23195635, 4.13)	LOC_Os11g38980 chr11 [23194768-23197394] -F-box/Kelch-repeat protein, putative, expressed (Os11g0602800, OsNippo11g222200)
LRI_2012DS9M_410	(chr11, 25059535, 4.93)	LOC_Os11g41730 chr11 [25058023-25062198] -transposon protein, putative, CACTA, En/Spm sub-class, expressed (OsNippo11g238900)

Do not distribute

LRI_2012DS9M_410	(chr12, 19954962, 4.26)	LOC_Os12g33030 chr12 [19954177-19955202] -expressed protein (OsNippo12g191050)
NDVI_2010	(chr01, 33229346, 4.13)	LOC_Os01g57510 chr01 [33227651-33231771] -receptor protein kinase, putative, expressed (Os01g0784200, OsNippo01g339550)
NDVI_2010	(chr01, 33594697, 4.28)	LOC_Os01g58080 chr01 [33593743-33597339] -membrane-associated salt-inducible protein, putative, expressed (Os01g0793200, OsNippo01g344500)
NDVI_2010	(chr01, 33638707, 4.32)	LOC_Os01g58170 chr01 [33638608-33639501] -expressed protein (OsNippo01g345000)
NDVI_2010	(chr02, 34657015, 4.47)	LOC_Os02g56570 chr02 [34654197-34658863] -TBC domain containing protein, expressed (Os02g0810500, OsNippo02g348150)
NDVI_2010	(chr03, 28936445, 4.12)	LOC_Os03g50670 chr03 [28931767-28940039] -retrotransposon protein, putative, Ty3-gypsy subclass, expressed (Os03g0714750, OsNippo03g290900)
NDVI_2010	(chr04, 148799, 4.01)	LOC_Os04g01210 chr04 [147419-149785] -retrotransposon protein, putative, Ty3-gypsy subclass, expressed (OsNippo04g011450)
NDVI_2010	(chr04, 24340632, 4.07)	LOC_Os04g41020 chr04 [24339608-24343784] -kelch repeat protein, putative, expressed (Os04g0487100, OsNippo04g235550)
NDVI_2010	(chr05, 23582621, 4.71)	LOC_Os05g40150 chr05 [23580974-23585400] -RGH2B, putative, expressed (Os05g0479700, OsNippo05g233850)

Do not distribute

NDVI_2010	(chr06, 21712967, 4.17)	LOC_Os06g36850 chr06 [21708950-21714106] -cysteine synthase, putative, expressed (Os06g0564600, OsNippo06g223700)
NDVI_2010	(chr11, 17388446, 4.15)	LOC_Os11g29920 chr11 [17385301-17393700] -NB-ARC domain containing protein, expressed (Os11g0491600, Os11g0493700, OsNippo11g169800)
NDVI_2010	(chr12, 22294068, 5.73)	LOC_Os12g36430 chr12 [22291141-22294802] -expressed protein (Os12g0550800, OsNippo12g212700)
NDVI_2011	(chr01, 1912846, 4.24)	LOC_Os01g04300 chr01 [1912560-1915094] -glycosyl hydrolase family 10 protein, putative, expressed (Os01g0134900, OsNippo01g031350)
NDVI_2011	(chr01, 28743606, 4.77)	LOC_Os01g50030 chr01 [28739986-28744762] -CPuORF25 - conserved peptide uORF-containing transcript, expressed (Os01g0695100, OsNippo01g292350)
NDVI_2011	(chr01, 28743606, 4.77)	LOC_Os01g50032 chr01 [28740235-28744474] -expressed protein (Os01g0695100, OsNippo01g292350)
NDVI_2011	(chr01, 28793263, 4.07)	LOC_Os01g50100 chr01 [28786108-28793300] -ABC transporter, ATP-binding protein, putative, expressed (Os01g0695800, OsNippo01g292650)
NDVI_2011	(chr01, 28848156, 4.87)	LOC_Os01g50200 chr01 [28847956-28849824] -UDP-glucoronosyl and UDP-glucosyl transferase domain containing protein, expressed (Os01g0697100, OsNippo01g293450)
NDVI_2011	(chr04, 3855757, 5.31)	LOC_Os04g07260 chr04 [3853319-3857891] -expressed protein (Os04g0156050, Os04g0156100, OsNippo04g045200)

Do not distribute

NDVI_2011	(chr04, 29880527, 4.36)	LOC_Os04g50090 chr04 [29877889-29881295] -helix-loop-helix DNA-binding protein, putative, expressed (Os04g0590800, OsNippo04g292950)
NDVI_2011	(chr06, 933310, 4.03)	LOC_Os06g02630 chr06 [933129-936449] -retrotransposon protein, putative, Ty3-gypsy subclass, expressed (Os06g0116900, OsNippo06g019600)
NDVI_2011	(chr06, 948949, 4.08)	LOC_Os06g02660 chr06 [947956-949606] -hypothetical protein (OsNippo06g019850)
NDVI_2011	(chr07, 24449326, 4.12)	LOC_Os07g40790 chr07 [24446288-24451350] -COBW domain containing protein, putative, expressed (Os07g0598900, OsNippo07g241450)
NDVI_2011	(chr12, 17625763, 4.43)	LOC_Os12g29570 chr12 [17624458-17626192] -expressed protein (Os12g0480100, OsNippo12g172250)
NDVI_2012	(chr01, 32081012, 4.5)	LOC_Os01g55700 chr01 [32072448-32082488] -NLI interacting factor-like phosphatase, putative, expressed (Os01g0762400, OsNippo01g328250)
NDVI_2012	(chr05, 8428388, 4.08)	LOC_Os05g14820 chr05 [8427408-8429407] -transmembrane amino acid transporter protein, putative, expressed (Os05g0237800, OsNippo05g090350)
NDVI_2012	(chr05, 16879501, 4.22)	LOC_Os05g28790 chr05 [16879337-16879862] -retrotransposon protein, putative, LINE subclass, expressed (OsNippo05g167400)
NDVI_2012	(chr10, 16242409, 4.22)	LOC_Os10g31050 chr10 [16239754-16242730] -expressed protein (Os10g0448000, OsNippo10g167750)

Do not distribute

NDVI_2012	(chr10, 16272204, 4.68)	LOC_Os10g31090 chr10 [16271956-16272426] -retrotransposon protein, putative, unclassified (OsNippo10g168000)
NDVI_2012	(chr10, 16326994, 4.22)	LOC_Os10g31170 chr10 [16323422-16327210] -plant protein of unknown function domain containing protein, expressed (Os10g0449532, Os10g0449600, OsNippo10g168450)

References

Kawahara, Y., de la Bastide, M., Hamilton J. P., Kanamori, H., McCombie, W. R., Ouyang, S.,
Yamamoto E, Yonemaru J, Yamamoto T, Yano M (2012) Rice OGRO: The Overview of functi

Do not distribute

ienome Annotation Project (Kawahara et al, 2013) and QTARO database (Yamamoto et al, 2012).

QTARO QTL

ccfs3 chr03[12459327-23887965] - qtaro-qtl:Mean of cumulative chlorophyll contents of the flag and second leaves (source activity - physiological trait) (id 1019; ref no. 911; year 2007; marker Mixture; cross SNU-SG1xMilyang 23); - chr03[16018787-23850248] - qtaro-qtl:Shoot length (shoot/seedling - morphological trait) (id 954; ref no. 836; year 1996; marker RFLP; cross LabellexBlack Gora); qSV-3-1 chr03[18350854-28859103] - qtaro-qtl:Shoot length (shoot/seedling - morphological trait) (id 608; ref no. 177; year 2005; marker SSR; cross LemontxTeqing)

qDSR 8 chr08[3927309-15529036] - qtaro-qtl:Dead seedling rate at 62 days (other soil stress tolerance - resistance or tolerance) (id 947; ref no. 827; year 2008; marker SSR; cross Gaochan 106xChangbai 9); qALRR-8 chr08[5326640-17528755] - qtaro-qtl:Root length ratio (other soil stress tolerance - resistance or tolerance) (id 222; ref no. 488; year 2002; marker Mixture; cross CT9993-5-10-1-MxIR62266-42-6-2)

qDSR 8 chr08[3927309-15529036] - qtaro-qtl:Dead seedling rate at 62 days (other soil stress tolerance - resistance or tolerance) (id 947; ref no. 827; year 2008; marker SSR; cross Gaochan 106xChangbai 9); qALRR-8 chr08[5326640-17528755] - qtaro-qtl:Root length ratio (other soil stress tolerance - resistance or tolerance) (id 222; ref no. 488; year 2002; marker Mixture; cross CT9993-5-10-1-MxIR62266-42-6-2)

qDSR 8 chr08[3927309-15529036] - qtaro-qtl:Dead seedling rate at 62 days (other soil stress tolerance - resistance or tolerance) (id 947; ref no. 827; year 2008; marker SSR; cross Gaochan 106xChangbai 9); qALRR-8 chr08[5326640-17528755] - qtaro-qtl:Root length ratio (other soil stress tolerance - resistance or tolerance) (id 222; ref no. 488; year 2002; marker Mixture; cross CT9993-5-10-1-MxIR62266-42-6-2)

qDSR 8 chr08[3927309-15529036] - qtaro-qtl:Dead seedling rate at 62 days (other soil stress tolerance - resistance or tolerance) (id 947; ref no. 827; year 2008; marker SSR; cross Gaochan 106xChangbai 9); qALRR-8 chr08[5326640-17528755] - qtaro-qtl:Root length ratio (other soil stress tolerance - resistance or tolerance) (id 222; ref no. 488; year 2002; marker Mixture; cross CT9993-5-10-1-MxIR62266-42-6-2)

qDSR 8 chr08[3927309-15529036] - qtaro-qtl:Dead seedling rate at 62 days (other soil stress tolerance - resistance or tolerance) (id 947; ref no. 827; year 2008; marker SSR; cross Gaochan 106xChangbai 9); qALRR-8 chr08[5326640-17528755] - qtaro-qtl:Root length ratio (other soil stress tolerance - resistance or tolerance) (id 222; ref no. 488; year 2002; marker Mixture; cross CT9993-5-10-1-MxIR62266-42-6-2)

qDSR 8 chr08[3927309-15529036] - qtaro-qtl:Dead seedling rate at 62 days (other soil stress tolerance - resistance or tolerance) (id 947; ref no. 827; year 2008; marker SSR; cross Gaochan 106xChangbai 9); qALRR-8 chr08[5326640-17528755] - qtaro-qtl:Root length ratio (other soil stress tolerance - resistance or tolerance) (id 222; ref no. 488; year 2002; marker Mixture; cross CT9993-5-10-1-MxIR62266-42-6-2);

qDSR 8 chr08[3927309-15529036] - qtaro-qtl:Dead seedling rate at 62 days (other soil stress tolerance - resistance or tolerance) (id 947; ref no. 827; year 2008; marker SSR; cross Gaochan 106xChangbai 9); qALRR-8 chr08[5326640-17528755] - qtaro-qtl:Root length ratio (other soil stress tolerance - resistance or tolerance) (id 222; ref no. 488; year 2002; marker Mixture; cross CT9993-5-10-1-MxIR62266-42-6-2)

qDSR 8 chr08[3927309-15529036] - qtaro-qtl:Dead seedling rate at 62 days (other soil stress tolerance - resistance or tolerance) (id 947; ref no. 827; year 2008; marker SSR; cross Gaochan 106xChangbai 9); qALRR-8 chr08[5326640-17528755] - qtaro-qtl:Root length ratio (other soil stress tolerance - resistance or tolerance) (id 222; ref no. 488; year 2002; marker Mixture; cross CT9993-5-10-1-MxIR62266-42-6-2)

Do not distribute

chr01[3483439-7970819] - qtaro-qtl:Root dry weight (root - morphological trait) (id 828; ref no. 717; year 2006; marker SSR; cross AkihikarixIRAT109); - chr01[3483439-10753685] - qtaro-qtl:Root axis length (root - morphological trait) (id 829; ref no. 717; year 2006; marker SSR; cross AkihikarixIRAT109); - chr01[4635793-25629404] - qtaro-qtl:K+1 concentration (salinity tolerance - resistance or tolerance) (id 196; ref no. 466; year 2001; marker Mixture; cross IR4630-xIR15324-); - chr01[4635793-27950354] - qtaro-qtl:Na+ uptake (salinity tolerance - resistance or tolerance) (id 195; ref no. 466; year 2001; marker Mixture; cross IR4630-xIR15324-)+B11+G12+G12+12:13+A12+12:13; rfw1b chr01[7476658-35715948] - qtaro-qtl:Root fresh weight (drought tolerance - chr01[4635793-25629404] - qtaro-qtl:K+1 concentration (salinity tolerance - resistance or tolerance) (id 196; ref no. 466; year 2001; marker Mixture; cross IR4630-xIR15324-); - chr01[4635793-27950354] - qtaro-qtl:Na+ uptake (salinity tolerance - resistance or tolerance) (id 195; ref no. 466; year 2001; marker Mixture; cross IR4630-xIR15324-); - rfw1b chr01[7476658-35715948] - qtaro-qtl:Root fresh weight (drought tolerance - resistance or tolerance) (id 97; ref no. 140; year 2005; marker RFLP; cross IRAT109xYuefu); brt1d chr01[14045859-42715593] - qtaro-qtl:Basal root thickness (drought tolerance - resistance or tolerance) (id 93; ref no. 140; year 2005; marker RFLP; cross IRAT109xYuefu); - chr01[20666582-25629404] - qtaro-qtl:Canopy temperature (CT) (drought tolerance - resistance or tolerance) (id 604; ref no. 176; year 2005; marker SSR; cross Zhenshan 97xIRAT109); qVB-1 chr01[22256683-24776333] - qtaro-qtl:No. of vascular bundles in peduncle (c+G11ulm/leaf - morphological trait) (id 521; ref no. qDSR 8 chr08[3927309-15529036] - qtaro-qtl:Dead seedling rate at 62 days (other soil stress tolerance - resistance or tolerance) (id 947; ref no. 827; year 2008; marker SSR; cross Gaochan 106xChangbai 9); qALRR-8 chr08[5326640-17528755] - qtaro-qtl:Root length ratio (other soil stress tolerance - resistance or tolerance) (id 222; ref no. 488; year 2002; marker Mixture; cross CT9993-5-10-1-MxIR62266-42-6-2)

CORN5 chr05[238143-3285751] - qtaro-qtl:The ratio of Rubisco to total leaf N content at 5days after heading (source activity - physiological trait) (id 194; ref no. 465; year 2001; marker RFLP; cross NipponbarexKasalath)

CORN5 chr05[238143-3285751] - qtaro-qtl:The ratio of Rubisco to total leaf N content at 5days after heading (source activity - physiological trait) (id 194; ref no. 465; year 2001; marker RFLP; cross NipponbarexKasalath)

CORN5 chr05[238143-3285751] - qtaro-qtl:The ratio of Rubisco to total leaf N content at 5days after heading (source activity - physiological trait) (id 194; ref no. 465; year 2001; marker RFLP; cross NipponbarexKasalath)

chr05[3320155-3597128] - qtaro-qtl:Rooting depth (root - morphological trait) (id 498; ref no. 208; year 2002; marker Mixture; cross CT9993xIR62266)

chr05[3320155-3597128] - qtaro-qtl:Rooting depth (root - morphological trait) (id 498; ref no. 208; year 2002; marker Mixture; cross CT9993xIR62266)

Do not distribute

qDLR11 chr11[383711-21835946] - qtaro-qtl:Dead leaf rate at 20 days (other soil stress tolerance - resistance or tolerance) (id 940; ref no. 827; year 2008; marker SSR; cross Gaochan 106xChangbai 9)

chr01[4635793-27950354] - qtaro-qtl:Na+ uptake (salinity tolerance - resistance or tolerance) (id 195; ref no. 466; year 2001; marker Mixture; cross IR4630-xIR15324-); rfw1b chr01[7476658-35715948] - qtaro-qtl:Root fresh weight (drought tolerance - resistance or tolerance) (id 97; ref no. 140; year 2005; marker RFLP; cross IRAT109xYuefu); brt1d chr01[14045859-42715593] - qtaro-qtl:Basal root thickness (drought tolerance - resistance or tolerance) (id 93; ref no. 140; year 2005; marker RFLP; cross IRAT109xYuefu)

chr03[31490136-35089139] - qtaro-qtl:Panicle length (drought tolerance - resistance or tolerance) (id 7; ref no. 22; year 2004; marker RFLP; cross AzucenaxBala)

chr03[31490136-35089139] - qtaro-qtl:Panicle length (drought tolerance - resistance or tolerance) (id 7; ref no. 22; year 2004; marker RFLP; cross AzucenaxBala)

Do not distribute

Qsbn3b chr03[35783061-37199035] - qtaro-qtl:Secondary branch number per panicle (panicle/flower - morphological trait) (id 471; ref no. 196; year 2001; marker Mixture; cross LemontxTeqing)

chr04[608084-4435752] - qtaro-qtl:P-use eficiency (other soil stress tolerance - resistance or tolerance) (id 999; ref no. 883; year 1998; marker RFLP; cross NipponbarexKasalath); qCTS4-1 chr04[679929-6571458] - qtaro-qtl:Cold tolerance (cold tolerance - resistance or tolerance) (id 543; ref no. 274; year 2003; marker SSR; cross M-202xIR50)

chr04[608084-4435752] - qtaro-qtl:P-use eficiency (other soil stress tolerance - resistance or tolerance) (id 999; ref no. 883; year 1998; marker RFLP; cross NipponbarexKasalath); qCTS4-1 chr04[679929-6571458] - qtaro-qtl:Cold tolerance (cold tolerance - resistance or tolerance) (id 543; ref no. 274; year 2003; marker SSR; cross M-202xIR50)

chr04[608084-4435752] - qtaro-qtl:P-use eficiency (other soil stress tolerance - resistance or tolerance) (id 999; ref no. 883; year 1998; marker RFLP; cross NipponbarexKasalath); qCTS4-1 chr04[679929-6571458] - qtaro-qtl:Cold tolerance (cold tolerance - resistance or tolerance) (id 543; ref no. 274; year 2003; marker SSR; cross M-202xIR50)

chr04[608084-4435752] - qtaro-qtl:P-use eficiency (other soil stress tolerance - resistance or tolerance) (id 999; ref no. 883; year 1998; marker RFLP; cross NipponbarexKasalath); qCTS4-1 chr04[679929-6571458] - qtaro-qtl:Cold tolerance (cold tolerance - resistance or tolerance) (id 543; ref no. 274; year 2003; marker SSR; cross M-202xIR50)

chr07[4085041-21393981] - qtaro-qtl:Days to wilt (DW4) (insect resistance - resistance or tolerance) (id 27; ref no. 38; year 2004; marker RFLP; cross IR64xAzucena);

chr07[4085041-21393981] - qtaro-qtl:Days to wilt (DW4) (insect resistance - resistance or tolerance) (id 27; ref no. 38; year 2004; marker RFLP; cross IR64xAzucena);

Do not distribute

QRl7 chr07[18481047-25763098] - qtaro-qtl:Leaf rolling (culm/leaf - morphological trait) (id 565; ref no. 112; year 1999; marker Mixture; cross LemontxTeqing); chr07[21001164-24959020] - qtaro-qtl:Root dry weight (other soil stress tolerance - resistance or tolerance) (id 416; ref no. 329; year 2003; marker RFLP; cross AsominorixIR24); qCIVG7-2 chr07[22532352-27297913] - qtaro-qtl:Cold response index for vigor of germination (cold tolerance - resistance or tolerance) (id 727; ref no. 542; year 2006; marker SSR; cross Milyang 25xJileng 1); qCDS-7 chr07[23518753-24685302] - qtaro-qtl:Chlorophyll degradation speed (source activity - physiological trait) (id 1034; ref no. 929; year 2007; marker RFLP; cross IR24xAsominori)

qDSR 8 chr08[3927309-15529036] - qtaro-qtl:Dead seedling rate at 62 days (other soil stress tolerance - resistance or tolerance) (id 947; ref no. 827; year 2008; marker SSR; cross Gaochan 106xChangbai 9); qALRR-8 chr08[5326640-17528755] - qtaro-qtl:Root length ratio (other soil stress tolerance - resistance or tolerance) (id 222; ref no. 488; year 2002; marker Mixture; cross CT9993-5-10-1-MxIR62266-42-6-2); chr08[6762708-12383755] - qtaro-qtl:Delay in flowering time by drought (DFT) (drought tolerance - resistance or tolerance) (id 599; ref no. 176; year 2005; marker SSR; cross Zhenshan 97xIRAT109)

chr08[24274675-27825769] - qtaro-qtl:root to shoot ratio,maximum root length (root - morphological trait) (id 933; ref no. 824; year 2002; marker RFLP; cross BalaxAzucena)

yldp9.1 chr09[19464779-23552932] - qtaro-qtl:Yield per plant (seed - morphological trait) (id 117; ref no. 147; year 2005; marker RFLP; cross IR 58025AxO. rufipogon (IC 22015)); Spk(t) chr09[21109960-22690613] - qtaro-qtl:Spread stub (culm/leaf - morphological trait) (id 553; ref no. 80; year 1997; marker RFLP; cross NipponbarexKasalath); - chr09[21313734-23027968] - qtaro-qtl:Maximum root length (root - morphological trait) (id 255; ref no. 513; year 2003; marker RFLP; cross IAC165xCo39); qTN-9-2 chr09[22021233-23027968] - qtaro-qtl:Tiller number (culm/leaf - morphological trait) (id 675; ref no. 643; year 2006; marker Mixture; cross Zhenshan 97xMinghui 63); - chr09[22021233-23552932] - qtaro-qtl:Maximum new root length NF-C (root -

qDLR11 chr11[383711-21835946] - qtaro-qtl:Dead leaf rate at 20 days (other soil stress tolerance - resistance or tolerance) (id 940; ref no. 827; year 2008; marker SSR; cross Gaochan 106xChangbai 9); fer11 chr11[4830321-8107623] - qtaro-qtl:Spikelet fertility (cold tolerance - resistance or tolerance) (id 651; ref no. 25; year 2004; marker SSR; cross Milyang 23xHapcheonaengmi 3); Tid1 chr11[6171645-8107623] - qtaro-qtl:Tid1 (dwarf - morphological trait) (id 328; ref no. 231; year 2004; marker SSR; cross Tid1-mutantxKasalath)

qDLR11 chr11[383711-21835946] - qtaro-qtl:Dead leaf rate at 20 days (other soil stress tolerance - resistance or tolerance) (id 940; ref no. 827; year 2008; marker SSR; cross Gaochan 106xChangbai 9); fer11 chr11[4830321-8107623] - qtaro-qtl:Spikelet fertility (cold tolerance - resistance or tolerance) (id 651; ref no. 25; year 2004; marker SSR; cross Milyang 23xHapcheonaengmi 3); Tid1 chr11[6171645-8107623] - qtaro-qtl:Tid1 (dwarf - morphological trait) (id 328; ref no. 231; year 2004; marker SSR; cross Tid1-mutantxKasalath)

qDLR11 chr11[383711-21835946] - qtaro-qtl:Dead leaf rate at 20 days (other soil stress tolerance - resistance or tolerance) (id 940; ref no. 827; year 2008; marker SSR; cross Gaochan 106xChangbai 9); fer11 chr11[4830321-8107623] - qtaro-qtl:Spikelet fertility (cold tolerance - resistance or tolerance) (id 651; ref no. 25; year 2004; marker SSR; cross Milyang 23xHapcheonaengmi 3); Tid1 chr11[6171645-8107623] - qtaro-qtl:Tid1 (dwarf - morphological trait) (id 328; ref no. 231; year 2004; marker SSR; cross Tid1-mutantxKasalath)

qDLR11 chr11[383711-21835946] - qtaro-qtl:Dead leaf rate at 20 days (other soil stress tolerance - resistance or tolerance) (id 940; ref no. 827; year 2008; marker SSR; cross Gaochan 106xChangbai 9); elr11-1 chr11[2391119-20101064] - qtaro-qtl:Cooked kernel elongation ratio (eating quality - physiological trait) (id 767; ref no. 615; year 2007; marker SSR; cross Pusa 1121xPusa 1342); - chr11[2827521-20702000] - qtaro-qtl:Grain length (seed - morphological trait) (id 154; ref no. 17; year 2002; marker SSR; cross ReihoxYamadanishiki)

qDLR11 chr11[383711-21835946] - qtaro-qtl:Dead leaf rate at 20 days (other soil stress tolerance - resistance or tolerance) (id 940; ref no. 827; year 2008; marker SSR; cross Gaochan 106xChangbai 9)

qDLR11 chr11[383711-21835946] - qtaro-qtl:Dead leaf rate at 20 days (other soil stress tolerance - resistance or tolerance) (id 940; ref no. 827; year 2008; marker SSR; cross Gaochan 106xChangbai 9)

chr12[1548039-18160498] - qtaro-qtl:Relative tillering ability (other soil stress tolerance - resistance or tolerance) (id 912; ref no. 810; year 1998; marker RFLP; cross IR20xIR55178-3B-9-3); qtl12.1 chr12[9895474-17758636] - qtaro-qtl:Flowering delay (drought tolerance - resistance or tolerance) (id 734; ref no. 544; year 2007; marker SSR; cross Way RaremxVandana)

Do not distribute

chr12[1548039-18160498] - qtaro-qtl:Relative tillering ability (other soil stress tolerance - resistance or tolerance) (id 912; ref no. 810; year 1998; marker RFLP; cross IR20xIR55178-3B-9-3); qtl12.1 chr12[9895474-17758636] - qtaro-qtl:Flowering delay (drought tolerance - resistance or tolerance) (id 734; ref no. 544; year 2007; marker SSR; cross Way RaremxVandana)

Gc chr01[84323-1058644] - qtaro-qtl:Chlorophyll content (others - others) (id 681; ref no. 567; year 2007; marker SSR; cross Chongqing 2xZhenshan 97B)

Gc chr01[84323-1058644] - qtaro-qtl:Chlorophyll content (others - others) (id 681; ref no. 567; year 2007; marker SSR; cross Chongqing 2xZhenshan 97B)

qFLL-1 chr01[1405980-5759851] - qtaro-qtl:First leaf length (culm/leaf - morphological trait) (id 397; ref no. 321; year 2003; marker RFLP; cross Zhenshan 97BxMilyang 46)

Do not distribute

qFLL-1 chr01[1405980-5759851] - qtaro-qtl:First leaf length (culm/leaf - morphological trait) (id 397; ref no. 321; year 2003; marker RFLP; cross Zhenshan 97BxMilyang 46); - chr01[3483439-7970819] - qtaro-qtl:Root dry weight (root - morphological trait) (id 828; ref no. 717; year 2006; marker SSR; cross AkihikarixIRAT109); - chr01[3483439-10753685] - qtaro-qtl:Root axis length (root - morphological trait) (id 829; ref no. 717; year 2006; marker SSR; cross AkihikarixIRAT109); chr01[4541296-7481804] - qtaro-qtl:Stem diameter (lodging resistance - resistance or tolerance) (id 712; ref no. 19; year 2004; marker RFLP; cross NipponbarexKasalath); qTNSP-1-1 chr01[4635793-5759851] - qtaro-qtl:Total number of spikelets per panicle (panicle/flower - morphological trait) (id 237; chr01[4635793-25629404] - qtaro-qtl:K+1 concentration (salinity tolerance - resistance or tolerance) (id 196; ref no. 466; year 2001; marker Mixture; cross IR4630-xIR15324-); - chr01[4635793-27950354] - qtaro-qtl:Na+:K+1 ratio (salinity tolerance - resistance or tolerance) (id 197; ref no. 466; year 2001; marker Mixture; cross IR4630-xIR15324-); rfw1b chr01[7476658-35715948] - qtaro-qtl:Root fresh weight (drought tolerance - resistance or tolerance) (id 97; ref no. 140; year 2005; marker RFLP; cross IRAT109xYuefu); - chr01[10753550-26104011] - qtaro-qtl:Branching index (root - morphological trait) (id 830; ref no. 717; year 2006; marker SSR; cross AkihikarixIRAT109); brt1d chr01[14045859-42715593] - qtaro-qtl:Basal root thickness (drought tolerance - chr01[4635793-25629404] - qtaro-qtl:K+1 concentration (salinity tolerance - resistance or tolerance) (id 196; ref no. 466; year 2001; marker Mixture; cross IR4630-xIR15324-); - chr01[4635793-27950354] - qtaro-qtl:Na+:K+1 ratio (salinity tolerance - resistance or tolerance) (id 197; ref no. 466; year 2001; marker Mixture; cross IR4630-xIR15324-); rfw1b chr01[7476658-35715948] - qtaro-qtl:Root fresh weight (drought tolerance - resistance or tolerance) (id 97; ref no. 140; year 2005; marker RFLP; cross IRAT109xYuefu); - chr01[10753550-26104011] - qtaro-qtl:Branching index (root - morphological trait) (id 830; ref no. 717; year 2006; marker SSR; cross AkihikarixIRAT109); brt1d chr01[14045859-42715593] - qtaro-qtl:Basal root thickness (drought tolerance - chr01[4635793-25629404] - qtaro-qtl:K+1 concentration (salinity tolerance - resistance or tolerance) (id 196; ref no. 466; year 2001; marker Mixture; cross IR4630-xIR15324-); - chr01[4635793-27950354] - qtaro-qtl:Na+:K+1 ratio (salinity tolerance - resistance or tolerance) (id 197; ref no. 466; year 2001; marker Mixture; cross IR4630-xIR15324-); rfw1b chr01[7476658-35715948] - qtaro-qtl:Root fresh weight (drought tolerance - resistance or tolerance) (id 97; ref no. 140; year 2005; marker RFLP; cross IRAT109xYuefu); - chr01[10753550-26104011] - qtaro-qtl:Branching index (root - morphological trait) (id 830; ref no. 717; year 2006; marker SSR; cross AkihikarixIRAT109); brt1d chr01[14045859-42715593] - qtaro-qtl:Basal root thickness (drought tolerance - chr01[5759741-39646396] - qtaro-qtl:Number of filled grains per panicle (source activity - physiological trait) (id 206; ref no. 476; year 2001; marker RFLP; cross Zhenshan 97BxMilyang 46); brt1d chr01[14045859-42715593] - qtaro-qtl:Basal root thickness (drought tolerance - resistance or tolerance) (id 93; ref no. 140; year 2005; marker RFLP; cross IRAT109xYuefu); chr01[34067651-42495953] - qtaro-qtl:Panicles/m2 (drought tolerance - resistance or tolerance) (id 863; ref no. 760; year 2002; marker RFLP; cross IR64xAzucena); - chr01[34539279-39646396] - qtaro-qtl:Length of the flag leaf (insect resistance - resistance or tolerance) (id 965; ref no. 844; year 2007; marker RFLP; cross IR36xTNAULFR831311); sf1.1 chr01[34810383-38491404] - qtaro-qtl:Spikelet fertility

chr02[5263536-18269395] - qtaro-qtl:Spikelet fertility (drought tolerance - resistance or tolerance) (id 11; ref no. 22; year 2004; marker RFLP; cross AzucenaxBala); tms5 chr02[6035616-6762680] - qtaro-qtl:Thermo-sensitive genic male sterile (sterility - physiological trait) (id 682; ref no. 569; year 2007; marker SSR; cross Y58SxGuanghui122)

chr02[5263536-18269395] - qtaro-qtl:Spikelet fertility (drought tolerance - resistance or tolerance) (id 11; ref no. 22; year 2004; marker RFLP; cross AzucenaxBala); tms5 chr02[6035616-6762680] - qtaro-qtl:Thermo-sensitive genic male sterile (sterility - physiological trait) (id 682; ref no. 569; year 2007; marker SSR; cross Y58SxGuanghui122)

qPH-2 chr02[5263536-30654749] - qtaro-qtl:Plant height (dwarf - morphological trait) (id 375; ref no. 308; year 2003; marker RFLP; cross Gui630x2428); np2.2 chr02[11418741-21658842] - qtaro-qtl:Number of panicles per plant (culm/leaf - morphological trait) (id 109; ref no. 147; year 2005; marker SSR; cross IR 58025AxO. rufipogon (IC 22015)); qSS-2 chr02[12245463-26758298] - qtaro-qtl:Spikelet fertility (sterility - physiological trait) (id 586; ref no. 167; year 2005; marker SSR; cross CPSLO17xW207-2); - chr02[18269869-20729299] - qtaro-qtl:Deep root weight per tiller (root - morphological trait) (id 261; ref no. 513; year 2003; marker RFLP; cross IAC165xCo39); qSTA-2 chr02[20199878-21658842] - qtaro-qtl:Stele transversal area (root - morphological trait) qCHR-2 chr02[26758064-36554631] - qtaro-qtl:Chlorate sensitivity (others - others) (id 292; ref no. 524; year 2006; marker Mixture; cross ZYQ8xJingxi 17); qDLR2-1 chr02[29160264-36128950] - qtaro-qtl:Dead leaf rate at 34 days (other soil stress tolerance - resistance or tolerance) (id 942; ref no. 827; year 2008; marker SSR; cross Gaochan 106xChangbai 9)

Do not distribute

chr03[464682-1429520] - qtaro-qtl:Total root number (root - morphological trait) (id 530; ref no. 251; year 2000; marker Mixture; cross IR64xAzucena)

chr03[464682-1429520] - qtaro-qtl:Total root number (root - morphological trait) (id 530; ref no. 251; year 2000; marker Mixture; cross IR64xAzucena)

chr03[464682-1429520] - qtaro-qtl:Total root number (root - morphological trait) (id 530; ref no. 251; year 2000; marker Mixture; cross IR64xAzucena)

qDEF-3 chr03[1423343-4098191] - qtaro-qtl:Days to emergence of flag-leaf (flowering - physiological trait) (id 268; ref no. 515; year 2004; marker RFLP; cross AsominorixIR24); QSs3 chr03[1429107-3215610] - qtaro-qtl:Spikelet sterility (sterility - physiological trait) (id 536; ref no. 265; year 1997; marker RFLP; cross LemontxTeqing); chr03[1772253-3475138] - qtaro-qtl:Deep root mass (root - morphological trait) (id 496; ref no. 208; year 2002; marker Mixture; cross CT9993xIR62266); chr03[2432425-3509693] - qtaro-qtl:Root volume (low moisture stress 85 days) (drought tolerance - resistance or tolerance) (id 303; ref no. 528; year 2002; marker Mixture; cross IR64xAzucena); rn3 chr03[2432425-3845280] - qtaro-qtl:Root number (drought tolerance - resistance or tolerance)

qDEF-3 chr03[1423343-4098191] - qtaro-qtl:Days to emergence of flag-leaf (flowering - physiological trait) (id 268; ref no. 515; year 2004; marker RFLP; cross AsominorixIR24); chr03[1772253-3475138] - qtaro-qtl:Deep root mass (root - morphological trait) (id 496; ref no. 208; year 2002; marker Mixture; cross CT9993xIR62266); chr03[2432425-3509693] - qtaro-qtl:Root volume (low moisture stress 85 days) (drought tolerance - resistance or tolerance) (id 303; ref no. 528; year 2002; marker Mixture; cross IR64xAzucena); rn3 chr03[2432425-3845280] - qtaro-qtl:Root number (drought tolerance - resistance or tolerance) (id 949; ref no. 831; year 2008; marker SSR; cross IRAT109xYuefu)
qTNL-3 chr03[4072902-6784194] - qtaro-qtl:Total number of leaves (culm/leaf - morphological trait) (id 267; ref no. 515; year 2004; marker RFLP; cross AsominorixIR24); Rhz2 chr03[6088558-7176458] - qtaro-qtl:Rhizome branching degree (others - others) (id 361; ref no. 288; year 2003; marker SSR; cross RD23xO. longistaminata accession with long and strong rhizomes)

dcf3 chr03[10000519-12459455] - qtaro-qtl:Degree of chlorophyll content of flag leaf at the heading date (source activity - physiological trait) (id 1016; ref no. 911; year 2007; marker Mixture; cross SNU-SG1xMilyang 23); qLEI3 chr03[10708808-12954471] - qtaro-qtl:Lowest elongated internode (submergency tolerance - resistance or tolerance) (id 1035; ref no. 132; year 2008; marker Mixture; cross T65xC9285)

chr04[608084-4435752] - qtaro-qtl:P-use eficiency (other soil stress tolerance - resistance or tolerance) (id 999; ref no. 883; year 1998; marker RFLP; cross NipponbarexKasalath); qCTS4-1 chr04[679929-6571458] - qtaro-qtl:Cold tolerance (cold tolerance - resistance or tolerance) (id 543; ref no. 274; year 2003; marker SSR; cross M-202xIR50); qSNP-4a chr04[679929-13211273] - qtaro-qtl:Spikelet number per panicle (panicle/flower - morphological trait) (id 49; ref no. 84; year 2006; marker SSR; cross LemontxTeqing); spp4-2 chr04[1137976-17888842] - qtaro-qtl:Spikelets per panicle (panicle/flower - morphological trait) (id 1005; ref no. 890; year 1996; marker RFLP; cross 9024xLH422)
chr04[608084-4435752] - qtaro-qtl:P-use eficiency (other soil stress tolerance - resistance or tolerance) (id 999; ref no. 883; year 1998; marker RFLP; cross NipponbarexKasalath); qCTS4-1 chr04[679929-6571458] - qtaro-qtl:Cold tolerance (cold tolerance - resistance or tolerance) (id 543; ref no. 274; year 2003; marker SSR; cross M-202xIR50); qSNP-4a chr04[679929-13211273] - qtaro-qtl:Spikelet number per panicle (panicle/flower - morphological trait) (id 49; ref no. 84; year 2006; marker SSR; cross LemontxTeqing); spp4-2 chr04[1137976-17888842] - qtaro-qtl:Spikelets per panicle (panicle/flower - morphological trait) (id 1005; ref no. 890; year 1996; marker RFLP; cross 9024xLH422)
spp4-2 chr04[1137976-17888842] - qtaro-qtl:Spikelets per panicle (panicle/flower - morphological trait) (id 1005; ref no. 890; year 1996; marker RFLP; cross 9024xLH422); qCCFJ-4 chr04[6571336-24646318] - qtaro-qtl:Leaf chlorophyll content at flowering (SPAD) (source activity - physiological trait) (id 81; ref no. 115; year 2005; marker SSR; cross IR36xNekken2); qPEN-4 chr04[8667374-13693920] - qtaro-qtl:Panicle exsertion (panicle/flower - morphological trait) (id 274; ref no. 517; year 2002; marker RFLP; cross IR64xAzucena); chr04[11283287-17888842] - qtaro-qtl:Deep root weight (root - morphological trait) (id 259; ref no. 513; year 2003; marker RFLP; cross IAC165xCo39); - chr04[11283287-20281368] - qtaro-qtl:Panicle length (PAL) (panicle/flower - morphological
qCCFJ-4 chr04[6571336-24646318] - qtaro-qtl:Leaf chlorophyll content at flowering (SPAD) (source activity - physiological trait) (id 81; ref no. 115; year 2005; marker SSR; cross IR36xNekken2); chr04[11283287-20281368] - qtaro-qtl:Panicle length (PAL) (panicle/flower - morphological trait) (id 645; ref no. 72; year 1996; marker RFLP; cross TesanaixCB)

qCCFJ-4 chr04[6571336-24646318] - qtaro-qtl:Leaf chlorophyll content at flowering (SPAD) (source activity - physiological trait) (id 81; ref no. 115; year 2005; marker SSR; cross IR36xNekken2); chr04[19921036-29158662] - qtaro-qtl:Boron tolerance (other soil stress tolerance - resistance or tolerance) (id 917; ref no. 815; year 2008; marker SSR; cross Nekken-1xIR36); orl1 chr04[20518311-24585809] - qtaro-qtl:- (culm/leaf - morphological trait) (id 460; ref no. 149; year 2001; marker RFLP; cross ORL1 heterozygous plantsx-)

rfw4a chr04[25626203-33083265] - qtaro-qtl:Root fresh weight (drought tolerance - resistance or tolerance) (id 951; ref no. 831; year 2008; marker SSR; cross IRAT109xYuefu); qCTS4-3 chr04[27441052-29644770] - qtaro-qtl:Cold-induced yellowing tolerance (cold tolerance - resistance or tolerance) (id 545; ref no. 274; year 2003; marker SSR; cross M-202xIR50); chr04[27941367-30762314] - qtaro-qtl:Maximum new root length Average (root - morphological trait) (id 68; ref no. 95; year 2006; marker SSR; cross OtomemochixYumenohatamochi); chr04[29158445-31356186] - qtaro-qtl:Spikelet number per panicle(35 d after heading) (panicle/flower - morphological trait) (id 574; ref no. 159; year 2005; marker SSR; cross Milyang 23xAkihikari)

Do not distribute

CORN5 chr05[238143-3285751] - qtaro-qtl:The ratio of Rubisco to total leaf N content at 5days after heading (source activity - physiological trait) (id 194; ref no. 465; year 2001; marker RFLP; cross NipponbarexKasalath)

CORN5 chr05[238143-3285751] - qtaro-qtl:The ratio of Rubisco to total leaf N content at 5days after heading (source activity - physiological trait) (id 194; ref no. 465; year 2001; marker RFLP; cross NipponbarexKasalath)

CORN5 chr05[238143-3285751] - qtaro-qtl:The ratio of Rubisco to total leaf N content at 5days after heading (source activity - physiological trait) (id 194; ref no. 465; year 2001; marker RFLP; cross NipponbarexKasalath)

CORN5 chr05[238143-3285751] - qtaro-qtl:The ratio of Rubisco to total leaf N content at 5days after heading (source activity - physiological trait) (id 194; ref no. 465; year 2001; marker RFLP; cross NipponbarexKasalath)

CORN5 chr05[238143-3285751] - qtaro-qtl:The ratio of Rubisco to total leaf N content at 5days after heading (source activity - physiological trait) (id 194; ref no. 465; year 2001; marker RFLP; cross NipponbarexKasalath)

nt5.1 chr05[7509091-10787755] - qtaro-qtl:Number of tillers per plant (culm/leaf - morphological trait) (id 110; ref no. 147; year 2005; marker SSR; cross IR 58025AxO. rufipogon (IC 22015)); yl(t) chr05[7509091-16531609] - qtaro-qtl:Yellowish-green leaf (shoot/seedling - morphological trait) (id 180; ref no. 191; year 2003; marker SSR; cross yl mutantxW002)

nt5.1 chr05[7509091-10787755] - qtaro-qtl:Number of tillers per plant (culm/leaf - morphological trait) (id 110; ref no. 147; year 2005; marker SSR; cross IR 58025AxO. rufipogon (IC 22015)); yl(t) chr05[7509091-16531609] - qtaro-qtl:Yellowish-green leaf (shoot/seedling - morphological trait) (id 180; ref no. 191; year 2003; marker SSR; cross yl mutantxW002)

yl(t) chr05[7509091-16531609] - qtaro-qtl:Yellowish-green leaf (shoot/seedling - morphological trait) (id 180; ref no. 191; year 2003; marker SSR; cross yl mutantxW002); qLTG-5 chr05[10787462-18944887] - qtaro-qtl:Germinability at low temperature (15ï¿½Å½) (cold tolerance - resistance or tolerance) (id 566; ref no. 113; year 1999; marker Mixture; cross Milyang 23xMilyang 23)

yl(t) chr05[7509091-16531609] - qtaro-qtl:Yellowish-green leaf (shoot/seedling - morphological trait) (id 180; ref no. 191; year 2003; marker SSR; cross yl mutantxW002); qLTG-5 chr05[10787462-18944887] - qtaro-qtl:Germinability at low temperature (15ï¿½Å½) (cold tolerance - resistance or tolerance) (id 566; ref no. 113; year 1999; marker Mixture; cross Milyang 23xMilyang 23)

Do not distribute

QFla5 chr05[23597602-29429411] - qtaro-qtl:Flag leaf angle (culm/leaf - morphological trait) (id 875; ref no. 773; year 1999; marker RFLP; cross LemontxTeqing); qRGR-5 chr05[24086072-27457701] - qtaro-qtl:Relative germination rate(RGR) (germination/dormancy - physiological trait) (id 126; ref no. 373; year 2006; marker SSR; cross Zhenshan 97xIRAT109)

alk6-1 chr06[1764586-5425602] - qtaro-qtl:Alkali-spread.score (eating quality - physiological trait) (id 4; ref no. 5; year 2004; marker SSR; cross CaiapoxOryza glaberrima (accession IRGC No. 103544)); AsTol chr06[2561213-6283916] - qtaro-qtl:Root length (other soil stress tolerance - resistance or tolerance) (id 817; ref no. 689; year 2004; marker RFLP; cross BalaxAzucena); qRCRJ-6 chr06[4234080-6399814] - qtaro-qtl:â€šâ€™eduction in chlorophyll content (source activity - physiological trait) (id 83; ref no. 115; year 1999; marker SSR; cross IR36xNekken2); "qLDLJ-6-1,2,3" chr06[4234080-28130383] - qtaro-qtl:Number of late-discoloring leaves per panicle at 25 DAF (source activity - physiological trait) (id 84; ref no. 115; year 1999; marker SSR; cross IR36xNekken2); "qLDLJ-6-1,2,3" chr06[4234080-28130383] - qtaro-qtl:Number of late-discoloring leaves per panicle at 25 DAF (source activity - physiological trait) (id 84; ref no. 115; year 1999; marker SSR; cross IR36xNekken2); tns6 chr06[6023974-9537572] - qtaro-qtl:Total number of spikelets/panicle (seed - morphological trait) (id 885; ref no. 777; year 1996; marker RFLP; cross Waiyin 2xCB); S5 chr06[6283432-9284248] - qtaro-qtl:Spikelet fertility (sterility - physiological trait) (id 529; ref no. 249; year 2000; marker -; cross Nanjing11xDuiai)

"qLDLJ-6-1,2,3" chr06[4234080-28130383] - qtaro-qtl:Number of late-discoloring leaves per panicle at 25 DAF (source activity - physiological trait) (id 84; ref no. 115; year 1999; marker SSR; cross IR36xNekken2)

"qLDLJ-6-1,2,3" chr06[4234080-28130383] - qtaro-qtl:Number of late-discoloring leaves per panicle at 25 DAF (source activity - physiological trait) (id 84; ref no. 115; year 1999; marker SSR; cross IR36xNekken2)

tp7b chr07[2349842-17535483] - qtaro-qtl:Tillers/plant (culm/leaf - morphological trait) (id 870; ref no. 767; year 2000; marker RFLP; cross Zhenshan 97xMinghui 6); - chr07[4085041-21393981] - qtaro-qtl:Days to wilt (DW4) (insect resistance - resistance or tolerance) (id 27; ref no. 38; year 2004; marker RFLP; cross IR64xAzucena); QHd7 chr07[4606397-6812968] - qtaro-qtl:Heading date (flowering - physiological trait) (id 371; ref no. 302; year 2003; marker RFLP; cross IR64xAzucena); qah7 chr07[4606397-16264722] - qtaro-qtl:Length of the heading period (flowering - physiological trait) (id 246; ref no. 508; year 2004; marker RFLP; cross NipponbarexKasalath); - chr07[4606397-17535483] - qtaro-qtl:Heading date (flowering - physiological trait) (id 915; ref no. 813; tp7b chr07[2349842-17535483] - qtaro-qtl:Tillers/plant (culm/leaf - morphological trait) (id 870; ref no. 767; year 2000; marker RFLP; cross Zhenshan 97xMinghui 6); - chr07[4085041-21393981] - qtaro-qtl:Days to wilt (DW4) (insect resistance - resistance or tolerance) (id 27; ref no. 38; year 2004; marker RFLP; cross IR64xAzucena); QHd7 chr07[4606397-6812968] - qtaro-qtl:Heading date (flowering - physiological trait) (id 371; ref no. 302; year 2003; marker RFLP; cross IR64xAzucena); qah7 chr07[4606397-16264722] - qtaro-qtl:Length of the heading period (flowering - physiological trait) (id 246; ref no. 508; year 2004; marker RFLP; cross NipponbarexKasalath); - chr07[4606397-17535483] - qtaro-qtl:Heading date (flowering - physiological trait) (id 915; ref no. 813; tp7b chr07[2349842-17535483] - qtaro-qtl:Tillers/plant (culm/leaf - morphological trait) (id 870; ref no. 767; year 2000; marker RFLP; cross Zhenshan 97xMinghui 6); - chr07[4085041-21393981] - qtaro-qtl:Days to wilt (DW4) (insect resistance - resistance or tolerance) (id 27; ref no. 38; year 2004; marker RFLP; cross IR64xAzucena); qah7 chr07[4606397-16264722] - qtaro-qtl:Length of the heading period (flowering - physiological trait) (id 246; ref no. 508; year 2004; marker RFLP; cross NipponbarexKasalath); - chr07[4606397-17535483] - qtaro-qtl:Heading date (flowering - physiological trait) (id 915; ref no. 813; year 2008; marker RFLP; cross HoshinoyumexKasalath); qHd-7 chr07[5512628-19619933] - qtaro-qtl:Heading date (flowering - physiological trait) (id 723; ref

Do not distribute

tp7b chr07[2349842-17535483] - qtaro-qtl:Tillers/plant (culm/leaf - morphological trait) (id 870; ref no. 767; year 2000; marker RFLP; cross Zhenshan 97xMinghui 6); - chr07[4085041-21393981] - qtaro-qtl:Days to wilt (DW4) (insect resistance - resistance or tolerance) (id 27; ref no. 38; year 2004; marker RFLP; cross IR64xAzucena); qah7 chr07[4606397-16264722] - qtaro-qtl:Length of the heading period (flowering - physiological trait) (id 246; ref no. 508; year 2004; marker RFLP; cross NipponbarexKasalath); - chr07[4606397-17535483] - qtaro-qtl:Heading date (flowering - physiological trait) (id 915; ref no. 813; year 2008; marker RFLP; cross HoshinoyumexKasalath); qHd-7 chr07[5512628-19619933] - qtaro-qtl:Heading date (flowering - physiological trait) (id 723; ref

tp7b chr07[2349842-17535483] - qtaro-qtl:Tillers/plant (culm/leaf - morphological trait) (id 870; ref no. 767; year 2000; marker RFLP; cross Zhenshan 97xMinghui 6); - chr07[4085041-21393981] - qtaro-qtl:Days to wilt (DW4) (insect resistance - resistance or tolerance) (id 27; ref no. 38; year 2004; marker RFLP; cross IR64xAzucena); qah7 chr07[4606397-16264722] - qtaro-qtl:Length of the heading period (flowering - physiological trait) (id 246; ref no. 508; year 2004; marker RFLP; cross NipponbarexKasalath); - chr07[4606397-17535483] - qtaro-qtl:Heading date (flowering - physiological trait) (id 915; ref no. 813; year 2008; marker RFLP; cross HoshinoyumexKasalath); qHd-7 chr07[5512628-19619933] - qtaro-qtl:Heading date (flowering - physiological trait) (id 723; ref

tp7b chr07[2349842-17535483] - qtaro-qtl:Tillers/plant (culm/leaf - morphological trait) (id 870; ref no. 767; year 2000; marker RFLP; cross Zhenshan 97xMinghui 6); - chr07[4085041-21393981] - qtaro-qtl:Days to wilt (DW4) (insect resistance - resistance or tolerance) (id 27; ref no. 38; year 2004; marker RFLP; cross IR64xAzucena); qah7 chr07[4606397-16264722] - qtaro-qtl:Length of the heading period (flowering - physiological trait) (id 246; ref no. 508; year 2004; marker RFLP; cross NipponbarexKasalath); - chr07[4606397-17535483] - qtaro-qtl:Heading date (flowering - physiological trait) (id 915; ref no. 813; year 2008; marker RFLP; cross HoshinoyumexKasalath); qHd-7 chr07[5512628-19619933] - qtaro-qtl:Heading date (flowering - physiological trait) (id 723; ref

tp7b chr07[2349842-17535483] - qtaro-qtl:Tillers/plant (culm/leaf - morphological trait) (id 870; ref no. 767; year 2000; marker RFLP; cross Zhenshan 97xMinghui 6); - chr07[4085041-21393981] - qtaro-qtl:Days to wilt (DW4) (insect resistance - resistance or tolerance) (id 27; ref no. 38; year 2004; marker RFLP; cross IR64xAzucena); qah7 chr07[4606397-16264722] - qtaro-qtl:Length of the heading period (flowering - physiological trait) (id 246; ref no. 508; year 2004; marker RFLP; cross NipponbarexKasalath); - chr07[4606397-17535483] - qtaro-qtl:Heading date (flowering - physiological trait) (id 915; ref no. 813; year 2008; marker RFLP; cross HoshinoyumexKasalath); qHd-7 chr07[5512628-19619933] - qtaro-qtl:Heading date (flowering - physiological trait) (id 723; ref

tp7b chr07[2349842-17535483] - qtaro-qtl:Tillers/plant (culm/leaf - morphological trait) (id 870; ref no. 767; year 2000; marker RFLP; cross Zhenshan 97xMinghui 6); - chr07[4085041-21393981] - qtaro-qtl:Days to wilt (DW4) (insect resistance - resistance or tolerance) (id 27; ref no. 38; year 2004; marker RFLP; cross IR64xAzucena); qah7 chr07[4606397-16264722] - qtaro-qtl:Length of the heading period (flowering - physiological trait) (id 246; ref no. 508; year 2004; marker RFLP; cross NipponbarexKasalath); - chr07[4606397-17535483] - qtaro-qtl:Heading date (flowering - physiological trait) (id 915; ref no. 813; year 2008; marker RFLP; cross HoshinoyumexKasalath); qHd-7 chr07[5512628-19619933] - qtaro-qtl:Heading date (flowering - physiological trait) (id 723; ref

qDTH8 chr08[360155-4446617] - qtaro-qtl:Days-to-heading (flowering - physiological trait) (id 749; ref no. 607; year 2007; marker Mixture; cross 98SQ1496xKoshihikari); - chr08[1584134-5333855] - qtaro-qtl:Cd concentration in brown rice (other soil stress tolerance - resistance or tolerance) (id 91; ref no. 130; year 2005; marker RFLP; cross KoshihikarixKasalath); - chr08[3170545-5333855] - qtaro-qtl:Heading date (flowering - physiological trait) (id 516; ref no. 223; year 2002; marker RFLP; cross Taichung 65xBhadua)

qDTH8 chr08[360155-4446617] - qtaro-qtl:Days-to-heading (flowering - physiological trait) (id 749; ref no. 607; year 2007; marker Mixture; cross 98SQ1496xKoshihikari); - chr08[1584134-5333855] - qtaro-qtl:Cd concentration in brown rice (other soil stress tolerance - resistance or tolerance) (id 91; ref no. 130; year 2005; marker RFLP; cross KoshihikarixKasalath); - chr08[3170545-5333855] - qtaro-qtl:Heading date (flowering - physiological trait) (id 516; ref no. 223; year 2002; marker RFLP; cross Taichung 65xBhadua); qDSR 8 chr08[3927309-15529036] - qtaro-qtl:Dead seedling rate at 62 days (other soil stress tolerance - resistance or tolerance) (id 947; ref no. 827; year 2008; marker SSR; cross Gaochan 106xChangbai 9)

qDSR 8 chr08[3927309-15529036] - qtaro-qtl:Dead seedling rate at 62 days (other soil stress tolerance - resistance or tolerance) (id 947; ref no. 827; year 2008; marker SSR; cross Gaochan 106xChangbai 9); dth8 chr08[4105519-25684949] - qtaro-qtl:Days to heading (flowering - physiological trait) (id 1007; ref no. 890; year 1996; marker RFLP; cross 9024xLH422); qALRR-8 chr08[5326640-17528755] - qtaro-qtl:Root length ratio (other soil stress tolerance - resistance or tolerance) (id 222; ref no. 488; year 2002; marker Mixture; cross CT9993-5-10-1-MxIR62266-42-6-2); nfb8 chr08[5421262-7568613] - qtaro-qtl:Number of first branches / main panicle (seed - morphological trait) (id 883; ref no. 777; year 1996; marker RFLP; cross Waiyin 2xCB); hd8 chr08[5421262-17528755]

qDSR 8 chr08[3927309-15529036] - qtaro-qtl:Dead seedling rate at 62 days (other soil stress tolerance - resistance or tolerance) (id 947; ref no. 827; year 2008; marker SSR; cross Gaochan 106xChangbai 9); dth8 chr08[4105519-25684949] - qtaro-qtl:Days to heading (flowering - physiological trait) (id 1007; ref no. 890; year 1996; marker RFLP; cross 9024xLH422); qALRR-8 chr08[5326640-17528755] - qtaro-qtl:Root length ratio (other soil stress tolerance - resistance or tolerance) (id 222; ref no. 488; year 2002; marker Mixture; cross CT9993-5-10-1-MxIR62266-42-6-2); nfb8 chr08[5421262-7568613] - qtaro-qtl:Number of first branches / main panicle (seed - morphological trait) (id 883; ref no. 777; year 1996; marker RFLP; cross Waiyin 2xCB); hd8 chr08[5421262-17528755]

Do not distribute

qDSR 8 chr08[3927309-15529036] - qtaro-qtl:Dead seedling rate at 62 days (other soil stress tolerance - resistance or tolerance) (id 947; ref no. 827; year 2008; marker SSR; cross Gaochan 106xChangbai 9); dth8 chr08[4105519-25684949] - qtaro-qtl:Days to heading (flowering - physiological trait) (id 1007; ref no. 890; year 1996; marker RFLP; cross 9024xLH422); qALRR-8 chr08[5326640-17528755] - qtaro-qtl:Root length ratio (other soil stress tolerance - resistance or tolerance) (id 222; ref no. 488; year 2002; marker Mixture; cross CT9993-5-10-1-MxIR62266-42-6-2); nfb8 chr08[5421262-7568613] - qtaro-qtl:Number of first branches / main panicle (seed - morphological trait) (id 883; ref no. 777; year 1996; marker RFLP; cross Waiyin 2xCB); hd8 chr08[5421262-17528755]

qDSR 8 chr08[3927309-15529036] - qtaro-qtl:Dead seedling rate at 62 days (other soil stress tolerance - resistance or tolerance) (id 947; ref no. 827; year 2008; marker SSR; cross Gaochan 106xChangbai 9); dth8 chr08[4105519-25684949] - qtaro-qtl:Days to heading (flowering - physiological trait) (id 1007; ref no. 890; year 1996; marker RFLP; cross 9024xLH422); qALRR-8 chr08[5326640-17528755] - qtaro-qtl:Root length ratio (other soil stress tolerance - resistance or tolerance) (id 222; ref no. 488; year 2002; marker Mixture; cross CT9993-5-10-1-MxIR62266-42-6-2); hd8 chr08[5421262-17528755] - qtaro-qtl:Heading date (flowering - physiological trait) (id 639; ref no. 66; year 1995; marker RFLP; cross Tesanai 2xCB); - chr08[6762708-12383755] - qtaro-qtl:Delay in flowering

qDSR 8 chr08[3927309-15529036] - qtaro-qtl:Dead seedling rate at 62 days (other soil stress tolerance - resistance or tolerance) (id 947; ref no. 827; year 2008; marker SSR; cross Gaochan 106xChangbai 9); dth8 chr08[4105519-25684949] - qtaro-qtl:Days to heading (flowering - physiological trait) (id 1007; ref no. 890; year 1996; marker RFLP; cross 9024xLH422); qALRR-8 chr08[5326640-17528755] - qtaro-qtl:Root length ratio (other soil stress tolerance - resistance or tolerance) (id 222; ref no. 488; year 2002; marker Mixture; cross CT9993-5-10-1-MxIR62266-42-6-2); hd8 chr08[5421262-17528755] - qtaro-qtl:Heading date (flowering - physiological trait) (id 639; ref no. 66; year 1995; marker RFLP; cross Tesanai 2xCB); - chr08[6762708-12383755] - qtaro-qtl:Delay in flowering

qDSR 8 chr08[3927309-15529036] - qtaro-qtl:Dead seedling rate at 62 days (other soil stress tolerance - resistance or tolerance) (id 947; ref no. 827; year 2008; marker SSR; cross Gaochan 106xChangbai 9); dth8 chr08[4105519-25684949] - qtaro-qtl:Days to heading (flowering - physiological trait) (id 1007; ref no. 890; year 1996; marker RFLP; cross 9024xLH422); qALRR-8 chr08[5326640-17528755] - qtaro-qtl:Root length ratio (other soil stress tolerance - resistance or tolerance) (id 222; ref no. 488; year 2002; marker Mixture; cross CT9993-5-10-1-MxIR62266-42-6-2); hd8 chr08[5421262-17528755] - qtaro-qtl:Heading date (flowering - physiological trait) (id 639; ref no. 66; year 1995; marker RFLP; cross Tesanai 2xCB); - chr08[6762708-12383755] - qtaro-qtl:Delay in flowering

dth8 chr08[4105519-25684949] - qtaro-qtl:Days to heading (flowering - physiological trait) (id 1007; ref no. 890; year 1996; marker RFLP; cross 9024xLH422); qALRR-8 chr08[5326640-17528755] - qtaro-qtl:Root length ratio (other soil stress tolerance - resistance or tolerance) (id 222; ref no. 488; year 2002; marker Mixture; cross CT9993-5-10-1-MxIR62266-42-6-2); hd8 chr08[5421262-17528755] - qtaro-qtl:Heading date (flowering - physiological trait) (id 639; ref no. 66; year 1995; marker RFLP; cross Tesanai 2xCB); qCRE-8 chr08[16626364-19399182] - qtaro-qtl:Control root elongation (other soil stress tolerance - resistance or tolerance) (id 252; ref no. 511; year 2006; marker RFLP; cross NipponbarexKasalath)

dth8 chr08[4105519-25684949] - qtaro-qtl:Days to heading (flowering - physiological trait) (id 1007; ref no. 890; year 1996; marker RFLP; cross 9024xLH422); qCRE-8 chr08[16626364-19399182] - qtaro-qtl:Control root elongation (other soil stress tolerance - resistance or tolerance) (id 252; ref no. 511; year 2006; marker RFLP; cross NipponbarexKasalath); - chr08[17528232-25684949] - qtaro-qtl:Osmotic adjustment at 70 % RWC (drought tolerance - resistance or tolerance) (id 616; ref no. 184; year 1996; marker RFLP; cross Moroberekanx C039)

dth8 chr08[4105519-25684949] - qtaro-qtl:Days to heading (flowering - physiological trait) (id 1007; ref no. 890; year 1996; marker RFLP; cross 9024xLH422); - chr08[17528232-25684949] - qtaro-qtl:Osmotic adjustment at 70 % RWC (drought tolerance - resistance or tolerance) (id 616; ref no. 184; year 1996; marker RFLP; cross Moroberekanx C039); 04-Aug chr08[24274675-27825769] - qtaro-qtl:root to shoot ratio,maximum root length (root - morphological trait) (id 933; ref no. 824; year 2002; marker RFLP; cross BalaxAzucena)

chr08[24274675-27825769] - qtaro-qtl:root to shoot ratio,maximum root length (root - morphological trait) (id 933; ref no. 824; year 2002; marker RFLP; cross BalaxAzucena); qRGV-8 chr08[26582935-28166213] - qtaro-qtl:Relative germination vigor(RGV) (germination/dormancy - physiological trait) (id 125; ref no. 373; year 2006; marker SSR; cross Zhenshan 97xIRAT109)

chr08[24274675-27825769] - qtaro-qtl:root to shoot ratio,maximum root length (root - morphological trait) (id 933; ref no. 824; year 2002; marker RFLP; cross BalaxAzucena); qRGV-8 chr08[26582935-28166213] - qtaro-qtl:Relative germination vigor(RGV) (germination/dormancy - physiological trait) (id 125; ref no. 373; year 2006; marker SSR; cross Zhenshan 97xIRAT109)

Do not distribute

qPC9 chr09[1119992-12373242] - qtaro-qtl:Protein content (eating quality - physiological trait) (id 621; ref no. 52; year 2006; marker SSR; cross MoritawasexKoshihikari); chr09[1159676-11367960] - qtaro-qtl:Maximum Root Length (root - morphological trait) (id 467; ref no. 195; year 2001; marker RFLP; cross ZYQ8xJingxi 17); clr9 chr09[2658483-16317550] - qtaro-qtl:Ratio of culm length reduction (cold tolerance - resistance or tolerance) (id 650; ref no. 25; year 2004; marker SSR; cross Milyang 23xHapcheonaengmi 3); chr09[6582100-12413787] - qtaro-qtl:Relative water content (drought tolerance - resistance or tolerance) (id 509; ref no. 215; year 2002; marker Mixture; cross BalaxAzucena)
qPC9 chr09[1119992-12373242] - qtaro-qtl:Protein content (eating quality - physiological trait) (id 621; ref no. 52; year 2006; marker SSR; cross MoritawasexKoshihikari); chr09[1159676-11367960] - qtaro-qtl:Maximum Root Length (root - morphological trait) (id 467; ref no. 195; year 2001; marker RFLP; cross ZYQ8xJingxi 17); clr9 chr09[2658483-16317550] - qtaro-qtl:Ratio of culm length reduction (cold tolerance - resistance or tolerance) (id 650; ref no. 25; year 2004; marker SSR; cross Milyang 23xHapcheonaengmi 3); chr09[6582100-12413787] - qtaro-qtl:Relative water content (drought tolerance - resistance or tolerance) (id 509; ref no. 215; year 2002; marker Mixture; cross BalaxAzucena)
qPC9 chr09[1119992-12373242] - qtaro-qtl:Protein content (eating quality - physiological trait) (id 621; ref no. 52; year 2006; marker SSR; cross MoritawasexKoshihikari); chr09[1159676-11367960] - qtaro-qtl:Maximum Root Length (root - morphological trait) (id 467; ref no. 195; year 2001; marker RFLP; cross ZYQ8xJingxi 17); clr9 chr09[2658483-16317550] - qtaro-qtl:Ratio of culm length reduction (cold tolerance - resistance or tolerance) (id 650; ref no. 25; year 2004; marker SSR; cross Milyang 23xHapcheonaengmi 3); chr09[6582100-12413787] - qtaro-qtl:Relative water content (drought tolerance - resistance or tolerance) (id 509; ref no. 215; year 2002; marker Mixture; cross BalaxAzucena); - chr09[9783058-12413337] - qtaro-qtl:Shoot dry weight
clr9 chr09[2658483-16317550] - qtaro-qtl:Ratio of culm length reduction (cold tolerance - resistance or tolerance) (id 650; ref no. 25; year 2004; marker SSR; cross Milyang 23xHapcheonaengmi 3); rdw9 chr09[12410287-18491736] - qtaro-qtl:Root dry weight (drought tolerance - resistance or tolerance) (id 95; ref no. 140; year 2005; marker Mixture; cross IRAT109xYuefu); chr09[15359476-18374564] - qtaro-qtl:The panicle length (PL) (panicle/flower - morphological trait) (id 709; ref no. 18; year 2004; marker SSR; cross Zhenshan 97xWuyujing 2)

rdw9 chr09[12410287-18491736] - qtaro-qtl:Root dry weight (drought tolerance - resistance or tolerance) (id 95; ref no. 140; year 2005; marker Mixture; cross IRAT109xYuefu); chr09[15359476-18374564] - qtaro-qtl:The panicle length (PL) (panicle/flower - morphological trait) (id 709; ref no. 18; year 2004; marker SSR; cross Zhenshan 97xWuyujing 2)

mrl9a chr09[18297411-20828859] - qtaro-qtl:Maximum root length (drought tolerance - resistance or tolerance) (id 950; ref no. 831; year 2008; marker SSR; cross IRAT109xYuefu); qCCAI-9 chr09[18374393-20828859] - qtaro-qtl:Leaf chlorophyll content 25 days after flowering (source activity - physiological trait) (id 82; ref no. 115; year 1999; marker SSR; cross IR36xNekken2); - chr09[18489616-21113859] - qtaro-qtl:Leaf bronzing index (other soil stress tolerance - resistance or tolerance) (id 414; ref no. 329; year 2003; marker RFLP; cross AsominorixIR24)

yldp9.1 chr09[19464779-23552932] - qtaro-qtl:Yield per plant (seed - morphological trait) (id 117; ref no. 147; year 2005; marker RFLP; cross IR 58025AxO. rufipogon (IC 22015)); - chr09[21313734-23027968] - qtaro-qtl:Maximum root length (root - morphological trait) (id 255; ref no. 513; year 2003; marker RFLP; cross IAC165xCo39); qTN-9-2 chr09[22021233-23027968] - qtaro-qtl:Tiller number (culm/leaf - morphological trait) (id 675; ref no. 643; year 2006; marker Mixture; cross Zhenshan 97xMinghui 63); - chr09[22021233-23552932] - qtaro-qtl:Maximum new root length NF-C (root - morphological trait) (id 66; ref no. 95; year 2006; marker SSR; cross OtomemochixYumenohatamochi)
yldp9.1 chr09[19464779-23552932] - qtaro-qtl:Yield per plant (seed - morphological trait) (id 117; ref no. 147; year 2005; marker RFLP; cross IR 58025AxO. rufipogon (IC 22015)); - chr09[21313734-23027968] - qtaro-qtl:Maximum root length (root - morphological trait) (id 255; ref no. 513; year 2003; marker RFLP; cross IAC165xCo39); qTN-9-2 chr09[22021233-23027968] - qtaro-qtl:Tiller number (culm/leaf - morphological trait) (id 675; ref no. 643; year 2006; marker Mixture; cross Zhenshan 97xMinghui 63); - chr09[22021233-23552932] - qtaro-qtl:Maximum new root length NF-C (root - morphological trait) (id 66; ref no. 95; year 2006; marker SSR; cross OtomemochixYumenohatamochi)
yldp9.1 chr09[19464779-23552932] - qtaro-qtl:Yield per plant (seed - morphological trait) (id 117; ref no. 147; year 2005; marker RFLP; cross IR 58025AxO. rufipogon (IC 22015)); - chr09[21313734-23027968] - qtaro-qtl:Maximum root length (root - morphological trait) (id 255; ref no. 513; year 2003; marker RFLP; cross IAC165xCo39); qTN-9-2 chr09[22021233-23027968] - qtaro-qtl:Tiller number (culm/leaf - morphological trait) (id 675; ref no. 643; year 2006; marker Mixture; cross Zhenshan 97xMinghui 63); - chr09[22021233-23552932] - qtaro-qtl:Maximum new root length NF-C (root - morphological trait) (id 66; ref no. 95; year 2006; marker SSR; cross OtomemochixYumenohatamochi)

Do not distribute

yldp9.1 chr09[19464779-23552932] - qtaro-qtl:Yield per plant (seed - morphological trait) (id 117; ref no. 147; year 2005; marker RFLP; cross IR 58025AxO. rufipogon (IC 22015)); - chr09[21313734-23027968] - qtaro-qtl:Maximum root length (root - morphological trait) (id 255; ref no. 513; year 2003; marker RFLP; cross IAC165xCo39); qTN-9-2 chr09[22021233-23027968] - qtaro-qtl:Tiller number (culm/leaf - morphological trait) (id 675; ref no. 643; year 2006; marker Mixture; cross Zhenshan 97xMinghui 63); - chr09[22021233-23552932] - qtaro-qtl:Maximum new root length NF-C (root - morphological trait) (id 66; ref no. 95; year 2006; marker SSR; cross OtomemochixYumenohatamochi)

yldp9.1 chr09[19464779-23552932] - qtaro-qtl:Yield per plant (seed - morphological trait) (id 117; ref no. 147; year 2005; marker RFLP; cross IR 58025AxO. rufipogon (IC 22015)); - chr09[21313734-23027968] - qtaro-qtl:Maximum root length (root - morphological trait) (id 255; ref no. 513; year 2003; marker RFLP; cross IAC165xCo39); qTN-9-2 chr09[22021233-23027968] - qtaro-qtl:Tiller number (culm/leaf - morphological trait) (id 675; ref no. 643; year 2006; marker Mixture; cross Zhenshan 97xMinghui 63); - chr09[22021233-23552932] - qtaro-qtl:Maximum new root length NF-C (root - morphological trait) (id 66; ref no. 95; year 2006; marker SSR; cross OtomemochixYumenohatamochi)
yldp9.1 chr09[19464779-23552932] - qtaro-qtl:Yield per plant (seed - morphological trait) (id 117; ref no. 147; year 2005; marker RFLP; cross IR 58025AxO. rufipogon (IC 22015)); - chr09[21313734-23027968] - qtaro-qtl:Maximum root length (root - morphological trait) (id 255; ref no. 513; year 2003; marker RFLP; cross IAC165xCo39); qTN-9-2 chr09[22021233-23027968] - qtaro-qtl:Tiller number (culm/leaf - morphological trait) (id 675; ref no. 643; year 2006; marker Mixture; cross Zhenshan 97xMinghui 63); - chr09[22021233-23552932] - qtaro-qtl:Maximum new root length NF-C (root - morphological trait) (id 66; ref no. 95; year 2006; marker SSR; cross OtomemochixYumenohatamochi)
yldp9.1 chr09[19464779-23552932] - qtaro-qtl:Yield per plant (seed - morphological trait) (id 117; ref no. 147; year 2005; marker RFLP; cross IR 58025AxO. rufipogon (IC 22015)); - chr09[21313734-23027968] - qtaro-qtl:Maximum root length (root - morphological trait) (id 255; ref no. 513; year 2003; marker RFLP; cross IAC165xCo39); qTN-9-2 chr09[22021233-23027968] - qtaro-qtl:Tiller number (culm/leaf - morphological trait) (id 675; ref no. 643; year 2006; marker Mixture; cross Zhenshan 97xMinghui 63); - chr09[22021233-23552932] - qtaro-qtl:Maximum new root length NF-C (root - morphological trait) (id 66; ref no. 95; year 2006; marker SSR; cross OtomemochixYumenohatamochi)
yldp9.1 chr09[19464779-23552932] - qtaro-qtl:Yield per plant (seed - morphological trait) (id 117; ref no. 147; year 2005; marker RFLP; cross IR 58025AxO. rufipogon (IC 22015)); - chr09[21313734-23027968] - qtaro-qtl:Maximum root length (root - morphological trait) (id 255; ref no. 513; year 2003; marker RFLP; cross IAC165xCo39); qTN-9-2 chr09[22021233-23027968] - qtaro-qtl:Tiller number (culm/leaf - morphological trait) (id 675; ref no. 643; year 2006; marker Mixture; cross Zhenshan 97xMinghui 63); - chr09[22021233-23552932] - qtaro-qtl:Maximum new root length NF-C (root - morphological trait) (id 66; ref no. 95; year 2006; marker SSR; cross OtomemochixYumenohatamochi)
yldp9.1 chr09[19464779-23552932] - qtaro-qtl:Yield per plant (seed - morphological trait) (id 117; ref no. 147; year 2005; marker RFLP; cross IR 58025AxO. rufipogon (IC 22015)); - chr09[21313734-23027968] - qtaro-qtl:Maximum root length (root - morphological trait) (id 255; ref no. 513; year 2003; marker RFLP; cross IAC165xCo39); qTN-9-2 chr09[22021233-23027968] - qtaro-qtl:Tiller number (culm/leaf - morphological trait) (id 675; ref no. 643; year 2006; marker Mixture; cross Zhenshan 97xMinghui 63); - chr09[22021233-23552932] - qtaro-qtl:Maximum new root length NF-C (root - morphological trait) (id 66; ref no. 95; year 2006; marker SSR; cross OtomemochixYumenohatamochi)
yldp9.1 chr09[19464779-23552932] - qtaro-qtl:Yield per plant (seed - morphological trait) (id 117; ref no. 147; year 2005; marker RFLP; cross IR 58025AxO. rufipogon (IC 22015)); - chr09[21313734-23027968] - qtaro-qtl:Maximum root length (root - morphological trait) (id 255; ref no. 513; year 2003; marker RFLP; cross IAC165xCo39); qTN-9-2 chr09[22021233-23027968] - qtaro-qtl:Tiller number (culm/leaf - morphological trait) (id 675; ref no. 643; year 2006; marker Mixture; cross Zhenshan 97xMinghui 63); - chr09[22021233-23552932] - qtaro-qtl:Maximum new root length NF-C (root - morphological trait) (id 66; ref no. 95; year 2006; marker SSR; cross OtomemochixYumenohatamochi)
yldp9.1 chr09[19464779-23552932] - qtaro-qtl:Yield per plant (seed - morphological trait) (id 117; ref no. 147; year 2005; marker RFLP; cross IR 58025AxO. rufipogon (IC 22015)); - chr09[21313734-23027968] - qtaro-qtl:Maximum root length (root - morphological trait) (id 255; ref no. 513; year 2003; marker RFLP; cross IAC165xCo39); qTN-9-2 chr09[22021233-23027968] - qtaro-qtl:Tiller number (culm/leaf - morphological trait) (id 675; ref no. 643; year 2006; marker Mixture; cross Zhenshan 97xMinghui 63); - chr09[22021233-23552932] - qtaro-qtl:Maximum new root length NF-C (root - morphological trait) (id 66; ref no. 95; year 2006; marker SSR; cross OtomemochixYumenohatamochi)

Do not distribute

chr10[3758658-13115650] - qtaro-qtl:Relative fresh weight (other stress resistance - resistance or tolerance) (id 384; ref no. 318; year 2003; marker RFLP; cross NipponbarexKasalath); qPBN-10 chr10[5016823-10014985] - qtaro-qtl:Primary branch number (panicle/flower - morphological trait) (id 48; ref no. 84; year 2006; marker SSR; cross LemontxTeqing)

qDLR11 chr11[383711-21835946] - qtaro-qtl:Dead leaf rate at 20 days (other soil stress tolerance - resistance or tolerance) (id 940; ref no. 827; year 2008; marker SSR; cross Gaochan 106xChangbai 9)

qDLR11 chr11[383711-21835946] - qtaro-qtl:Dead leaf rate at 20 days (other soil stress tolerance - resistance or tolerance) (id 940; ref no. 827; year 2008; marker SSR; cross Gaochan 106xChangbai 9)

qDLR11 chr11[383711-21835946] - qtaro-qtl:Dead leaf rate at 20 days (other soil stress tolerance - resistance or tolerance) (id 940; ref no. 827; year 2008; marker SSR; cross Gaochan 106xChangbai 9)

qDLR11 chr11[383711-21835946] - qtaro-qtl:Dead leaf rate at 20 days (other soil stress tolerance - resistance or tolerance) (id 940; ref no. 827; year 2008; marker SSR; cross Gaochan 106xChangbai 9)

Do not distribute

rfw1b chr01[7476658-35715948] - qtaro-qtl:Root fresh weight (drought tolerance - resistance or tolerance) (id 97; ref no. 140; year 2005; marker RFLP; cross IRAT109xYuefu); brt1d chr01[14045859-42715593] - qtaro-qtl:Basal root thickness (drought tolerance - resistance or tolerance) (id 93; ref no. 140; year 2005; marker RFLP; cross IRAT109xYuefu); qLRC-1 chr01[28571547-34744337] - qtaro-qtl:Leaf rolling of culturing (LRC) (drought tolerance - resistance or tolerance) (id 128; ref no. 373; year 2006; marker SSR; cross Zhenshan 97xIRAT109); gpy1 chr01[31822907-33817439] - qtaro-qtl:Green plantlet yield frequency (others - others) (id 748; ref no. 606; year 1998; marker RFLP; cross ZYQ8xJingxi 17); bc7(t) chr01[33168525-33249565] - qtaro-qtl:Brittle culm (culm/leaf -
rfw1b chr01[7476658-35715948] - qtaro-qtl:Root fresh weight (drought tolerance - resistance or tolerance) (id 97; ref no. 140; year 2005; marker RFLP; cross IRAT109xYuefu); brt1d chr01[14045859-42715593] - qtaro-qtl:Basal root thickness (drought tolerance - resistance or tolerance) (id 93; ref no. 140; year 2005; marker RFLP; cross IRAT109xYuefu); qLRC-1 chr01[28571547-34744337] - qtaro-qtl:Leaf rolling of culturing (LRC) (drought tolerance - resistance or tolerance) (id 128; ref no. 373; year 2006; marker SSR; cross Zhenshan 97xIRAT109); gpy1 chr01[31822907-33817439] - qtaro-qtl:Green plantlet yield frequency (others - others) (id 748; ref no. 606; year 1998; marker RFLP; cross ZYQ8xJingxi 17); - chr01[33178792-34810544] - qtaro-qtl:Deep root per tiller (root -
rfw1b chr01[7476658-35715948] - qtaro-qtl:Root fresh weight (drought tolerance - resistance or tolerance) (id 97; ref no. 140; year 2005; marker RFLP; cross IRAT109xYuefu); brt1d chr01[14045859-42715593] - qtaro-qtl:Basal root thickness (drought tolerance - resistance or tolerance) (id 93; ref no. 140; year 2005; marker RFLP; cross IRAT109xYuefu); qLRC-1 chr01[28571547-34744337] - qtaro-qtl:Leaf rolling of culturing (LRC) (drought tolerance - resistance or tolerance) (id 128; ref no. 373; year 2006; marker SSR; cross Zhenshan 97xIRAT109); gpy1 chr01[31822907-33817439] - qtaro-qtl:Green plantlet yield frequency (others - others) (id 748; ref no. 606; year 1998; marker RFLP; cross ZYQ8xJingxi 17); - chr01[33178792-34810544] - qtaro-qtl:Deep root per tiller (root -
qCHR-2 chr02[26758064-36554631] - qtaro-qtl:Chlorate sensitivity (others - others) (id 292; ref no. 524; year 2006; marker Mixture; cross ZYQ8xJingxi 17); qDLR2-1 chr02[29160264-36128950] - qtaro-qtl:Dead leaf rate at 34 days (other soil stress tolerance - resistance or tolerance) (id 942; ref no. 827; year 2008; marker SSR; cross Gaochan 106xChangbai 9); Hd7 chr02[29242392-35959447] - qtaro-qtl:Heading date (flowering - physiological trait) (id 528; ref no. 248; year 2000; marker RFLP; cross NipponbarexKasalath); chr02[33667096-34831889] - qtaro-qtl:Shoot length (shoot/seedling - morphological trait) (id 704; ref no. 15; year 2004; marker Mixture; cross LemontxTeqing); sn2.1 chr02[33667096-36028798] - qtaro-qtl:Spikelet number per ppanicle
Yd3-13 chr03[23850041-28999468] - qtaro-qtl:Yield (seed - morphological trait) (id 678; ref no. 562; year 2007; marker RFLP; cross IR64xAzucena); - chr03[23850041-29580503] - qtaro-qtl:Panicle size (panicle/flower - morphological trait) (id 957; ref no. 837; year 1998; marker RFLP; cross LabellexBlack Gora); - chr03[23850041-31298638] - qtaro-qtl:Coleoptile length (shoot/seedling - morphological trait) (id 955; ref no. 836; year 1996; marker RFLP; cross LabellexBlack Gora); - chr03[27494415-31496678] - qtaro-qtl:Carbon isotope discrimination(Æ'Â¢ 13C) in IRRI 1996 (drought tolerance - resistance or tolerance) (id 223; ref no. 489; year 2002; marker Mixture; cross BalaxAzucena); qRFWd3-2 chr03[27894595-29301670] - qtaro-qtl:Root fresh weight (Water deficit)

Do not distribute

qCCFJ-4 chr04[6571336-24646318] - qtaro-qtl:Leaf chlorophyll content at flowering (SPAD) (source activity - physiological trait) (id 81; ref no. 115; year 2005; marker SSR; cross IR36xNekken2); - chr04[19921036-29158662] - qtaro-qtl:Boron tolerance (other soil stress tolerance - resistance or tolerance) (id 917; ref no. 815; year 2008; marker SSR; cross Nekken-1xIR36); orl1 chr04[20518311-24585809] - qtaro-qtl:- (culm/leaf - morphological trait) (id 460; ref no. 149; year 2001; marker RFLP; cross ORL1 heterozygous plantsx-); qChla4-1 chr04[22859833-27441315] - qtaro-qtl:Chlorophyll a (shoot/seedling - morphological trait) (id 811; ref no. 688; year 2002; marker Mixture; cross Zhenshan 97AxMinghui 63)
QPl5 chr05[18944261-29429411] - qtaro-qtl:Spikelet density (panicle/flower - morphological trait) (id 669; ref no. 621; year 1998; marker RFLP; cross LemontxTeqing)

"qLDLJ-6-1,2,3" chr06[4234080-28130383] - qtaro-qtl:Number of late-discoloring leaves per panicle at 25 DAF (source activity - physiological trait) (id 84; ref no. 115; year 1999; marker SSR; cross IR36xNekken2)

qDLR11 chr11[383711-21835946] - qtaro-qtl:Dead leaf rate at 20 days (other soil stress tolerance - resistance or tolerance) (id 940; ref no. 827; year 2008; marker SSR; cross Gaochan 106xChangbai 9); hbd3 chr11[15492181-19497296] - qtaro-qtl:The number of tillers (lethality - physiological trait) (id 670; ref no. 622; year 2007; marker Mixture; cross KoshihikarixHabataki); Hbd3 chr11[16351679-19497296] - qtaro-qtl:No. of tiller (lethality - physiological trait) (id 761; ref no. 611; year 2007; marker SSR; cross SL-407 (Sasanishiki/Habataki//Sasanishiki)xHabataki)

Do not distribute

qFLL-1 chr01[1405980-5759851] - qtaro-qtl:First leaf length (culm/leaf - morphological trait) (id 397; ref no. 321; year 2003; marker RFLP; cross Zhenshan 97BxMilyang 46)

rfw1b chr01[7476658-35715948] - qtaro-qtl:Root fresh weight (drought tolerance - resistance or tolerance) (id 97; ref no. 140; year 2005; marker RFLP; cross IRAT109xYuefu); brt1d chr01[14045859-42715593] - qtaro-qtl:Basal root thickness (drought tolerance - resistance or tolerance) (id 93; ref no. 140; year 2005; marker RFLP; cross IRAT109xYuefu); chr01[27797914-30720282] - qtaro-qtl:Root-to-shoot ratio Average (root - morphological trait) (id 70; ref no. 95; year 2006; marker SSR; cross OtomemochixYumenohatamochi); qLRC-1 chr01[28571547-34744337] - qtaro-qtl:Leaf rolling of culturing (LRC) (drought tolerance - resistance or tolerance) (id 128; ref no. 373; year 2006; marker SSR; cross Zhenshan 97xIRAT109); qCC-1 chr01[28628063-
rfw1b chr01[7476658-35715948] - qtaro-qtl:Root fresh weight (drought tolerance - resistance or tolerance) (id 97; ref no. 140; year 2005; marker RFLP; cross IRAT109xYuefu); brt1d chr01[14045859-42715593] - qtaro-qtl:Basal root thickness (drought tolerance - resistance or tolerance) (id 93; ref no. 140; year 2005; marker RFLP; cross IRAT109xYuefu); chr01[27797914-30720282] - qtaro-qtl:Root-to-shoot ratio Average (root - morphological trait) (id 70; ref no. 95; year 2006; marker SSR; cross OtomemochixYumenohatamochi); qLRC-1 chr01[28571547-34744337] - qtaro-qtl:Leaf rolling of culturing (LRC) (drought tolerance - resistance or tolerance) (id 128; ref no. 373; year 2006; marker SSR; cross Zhenshan 97xIRAT109); qCC-1 chr01[28628063-
rfw1b chr01[7476658-35715948] - qtaro-qtl:Root fresh weight (drought tolerance - resistance or tolerance) (id 97; ref no. 140; year 2005; marker RFLP; cross IRAT109xYuefu); brt1d chr01[14045859-42715593] - qtaro-qtl:Basal root thickness (drought tolerance - resistance or tolerance) (id 93; ref no. 140; year 2005; marker RFLP; cross IRAT109xYuefu); chr01[27797914-30720282] - qtaro-qtl:Root-to-shoot ratio Average (root - morphological trait) (id 70; ref no. 95; year 2006; marker SSR; cross OtomemochixYumenohatamochi); qLRC-1 chr01[28571547-34744337] - qtaro-qtl:Leaf rolling of culturing (LRC) (drought tolerance - resistance or tolerance) (id 128; ref no. 373; year 2006; marker SSR; cross Zhenshan 97xIRAT109); qCC-1 chr01[28628063-
rfw1b chr01[7476658-35715948] - qtaro-qtl:Root fresh weight (drought tolerance - resistance or tolerance) (id 97; ref no. 140; year 2005; marker RFLP; cross IRAT109xYuefu); brt1d chr01[14045859-42715593] - qtaro-qtl:Basal root thickness (drought tolerance - resistance or tolerance) (id 93; ref no. 140; year 2005; marker RFLP; cross IRAT109xYuefu); chr01[27797914-30720282] - qtaro-qtl:Root-to-shoot ratio Average (root - morphological trait) (id 70; ref no. 95; year 2006; marker SSR; cross OtomemochixYumenohatamochi); qLRC-1 chr01[28571547-34744337] - qtaro-qtl:Leaf rolling of culturing (LRC) (drought tolerance - resistance or tolerance) (id 128; ref no. 373; year 2006; marker SSR; cross Zhenshan 97xIRAT109); qCC-1 chr01[28628063-
chr04[608084-4435752] - qtaro-qtl:P-use eficiency (other soil stress tolerance - resistance or tolerance) (id 999; ref no. 883; year 1998; marker RFLP; cross NipponbarexKasalath); qCTS4-1 chr04[679929-6571458] - qtaro-qtl:Cold tolerance (cold tolerance - resistance or tolerance) (id 543; ref no. 274; year 2003; marker SSR; cross M-202xIR50); qSNP-4a chr04[679929-13211273] - qtaro-qtl:Spikelet number per panicle (panicle/flower - morphological trait) (id 49; ref no. 84; year 2006; marker SSR; cross LemontxTeqing); spp4-2 chr04[1137976-17888842] - qtaro-qtl:Spikelets per panicle (panicle/flower - morphological trait) (id 1005; ref no. 890; year 1996; marker RFLP; cross 9024xLH422)

rfw4a chr04[25626203-33083265] - qtaro-qtl:Root fresh weight (drought tolerance - resistance or tolerance) (id 951; ref no. 831; year 2008; marker SSR; cross IRAT109xYuefu); - chr04[27941367-30762314] - qtaro-qtl:Maximum new root length Average (root - morphological trait) (id 68; ref no. 95; year 2006; marker SSR; cross OtomemochixYumenohatamochi); - chr04[29158445-31356186] - qtaro-qtl:Spikelet number per panicle(35 d after heading) (panicle/flower - morphological trait) (id 574; ref no. 159; year 2005; marker SSR; cross Milyang 23xAkihikari); OsAOX1a chr04[29737978-33358474] - qtaro-qtl:Alternative oxidase (cold tolerance - resistance or tolerance) (id 212; ref no. 477; year 2002; marker RFLP; cross Hokkai241xSilewah)

qHD6 chr06[483009-1562787] - qtaro-qtl:Heading date (flowering - physiological trait) (id 620; ref no. 52; year 2006; marker SSR; cross MoritawasexKoshihikari)

qHD6 chr06[483009-1562787] - qtaro-qtl:Heading date (flowering - physiological trait) (id 620; ref no. 52; year 2006; marker SSR; cross MoritawasexKoshihikari)

QRl7 chr07[18481047-25763098] - qtaro-qtl:Leaf rolling (culm/leaf - morphological trait) (id 565; ref no. 112; year 1999; marker Mixture; cross LemontxTeqing); qSDS-7-2 chr07[19354374-26133946] - qtaro-qtl:Seed â€šâ€žormancy (germination/dormancy - physiological trait) (id 700; ref no. 12; year 2004; marker SSR; cross EM93-1xSS18-2); - chr07[21001164-24959020] - qtaro-qtl:Root dry weight (other soil stress tolerance - resistance or tolerance) (id 416; ref no. 329; year 2003; marker RFLP; cross AsominorixIR24); qCIVG7-2 chr07[22532352-27297913] - qtaro-qtl:Cold response index for vigor of germination (cold tolerance - resistance or tolerance) (id 727; ref no. 542; year 2006; marker SSR; cross Milyang 25xJileng 1); - chr07[23326303-25284593] - qtaro-

chr12[1548039-18160498] - qtaro-qtl:Relative shoot dry weight (other soil stress tolerance - resistance or tolerance) (id 913; ref no. 810; year 1998; marker AFLP; cross IR20xIR55178-3B-9-3)

Do not distribute

rfw1b chr01[7476658-35715948] - qtaro-qtl:Root fresh weight (drought tolerance - resistance or tolerance) (id 97; ref no. 140; year 2005; marker RFLP; cross IRAT109xYuefu); brt1d chr01[14045859-42715593] - qtaro-qtl:Basal root thickness (drought tolerance - resistance or tolerance) (id 93; ref no. 140; year 2005; marker RFLP; cross IRAT109xYuefu); qLRC-1 chr01[28571547-34744337] - qtaro-qtl:Leaf rolling of culturing (LRC) (drought tolerance - resistance or tolerance) (id 128; ref no. 373; year 2006; marker SSR; cross Zhenshan 97xIRAT109); qCC-1 chr01[31822907-33817439] - qtaro-qtl:Chlorophyll content (source activity - physiological trait) (id 286; ref no. 521; year 2004; marker Mixture; cross ZYQ8xJingxi 17)

snp5.1 chr05[5311917-10787755] - qtaro-qtl:Spikelet number per plant (panicle/flower - morphological trait) (id 116; ref no. 147; year 2005; marker SSR; cross IR 58025AxO. rufipogon (IC 22015)); yl(t) chr05[7509091-16531609] - qtaro-qtl:Yellowish-green leaf (shoot/seedling - morphological trait) (id 180; ref no. 191; year 2003; marker SSR; cross yl mutantxW002)

QKw5 chr05[6115238-18944887] - qtaro-qtl:1000 kernel weight (seed - morphological trait) (id 533; ref no. 264; year 1997; marker RFLP; cross LemontxTeqing); qLTG-5 chr05[10787462-18944887] - qtaro-qtl:Germinability at low temperature (15ï¿½Å½) (cold tolerance - resistance or tolerance) (id 566; ref no. 113; year 1999; marker Mixture; cross Milyang 23xMilyang 23)

ssd10 chr10[12044545-19623828] - qtaro-qtl:Spikelets setting density (panicle/flower - morphological trait) (id 1008; ref no. 890; year 1996; marker RFLP; cross 9024xLH422); - chr10[13330314-19551633] - qtaro-qtl:Root penetration index(Sum.) (drought tolerance - resistance or tolerance) (id 783; ref no. 664; year 2000; marker Mixture; cross IR58821-23-B-1-2-1xIR52561-UBN-1-1-2); - chr10[15737354-17165630] - qtaro-qtl:Drought respond index (DRI) (drought tolerance - resistance or tolerance) (id 601; ref no. 176; year 2005; marker SSR; cross Zhenshan 97xIRAT109)

chr10[13330314-19551633] - qtaro-qtl:Root penetration index(Sum.) (drought tolerance - resistance or tolerance) (id 783; ref no. 664; year 2000; marker Mixture; cross IR58821-23-B-1-2-1xIR52561-UBN-1-1-2); - chr10[15737354-17165630] - qtaro-qtl:Drought respond index (DRI) (drought tolerance - resistance or tolerance) (id 601; ref no. 176; year 2005; marker SSR; cross Zhenshan 97xIRAT109)

chr10[13330314-19551633] - qtaro-qtl:Root penetration index(Sum.) (drought tolerance - resistance or tolerance) (id 783; ref no. 664; year 2000; marker Mixture; cross IR58821-23-B-1-2-1xIR52561-UBN-1-1-2); - chr10[15737354-17165630] - qtaro-qtl:Drought respond index (DRI) (drought tolerance - resistance or tolerance) (id 601; ref no. 176; year 2005; marker SSR; cross Zhenshan 97xIRAT109); - chr10[16326502-16330772] - qtaro-qtl:Plant regeneration ability in anther culture (others - others) (id 220; ref no. 483; year 2002; marker RFLP; cross Milyang 23xGihobyeo)

, Schwartz, D. C., Tanaka, T., Wu, J., Zhou, S., Childs, K. L., Davidson, R. M., Lin, H., Quesada-Ocampo, L., Vaillancourt, B., Sakai, H., Lee,
ionally characterized Genes in Rice online database. Rice 5:26 doi;10.1186/1939-8433-5-26

Do not distribute

Do not distribute

Do not distribute

Do not distribute

Do not distribute

Do not distribute

Do not distribute

Do not distribute

Do not distribute

Do not distribute

Do not distribute

Do not distribute

Do not distribute

Do not distribute

Do not distribute

Do not distribute

Do not distribute

Do not distribute

Do not distribute

a Nipponbare reference genome using next generation sequence and optical map data.*Rice* 6:4.

Do not distribute

Supp. Table S13. Aus candidate loci for leaf rolling under drought and their annotations

Candidate Locus	Annotation
LOC_Os09g23200	KANADI1, putative, expressed; SHALLOT-LIKE1 controls leaf rolling
LOC_Os12g36430	no Pfam or other hits
LOC_Os02g45250	homeobox and START domains containing protein, putative, expressed; Isolation and characterization of rl(t), a gene that controls leaf rolling in rice. Li, L., Xue, X., Chen, Z. et al. Chin. Sci. Bull. (2014) 59:3142. DOI:10.1007/s11434-014-0357-8.
LOC_Os04g04020.1	Gene Product Name: protein transport protein Sec24-like, putative, expressed
LOC_Os03g57300	Tetratricopeptide repeat (TPR)-like superfamily protein, likely component of TRAPP complex, TRS85
LOC_Os06g36850	cysteine synthase, putative, expressed
LOC_Os09g39670	oxidoreductase, short chain dehydrogenase/reductase family domain containing family, expressed

Do not distribute

Supp. Table S14. Tropical japonica candidate loci for leaf rolling under drought and their annotati

**Locus**	**Annotation**
LOC_Os02g10794	expressed protein
LOC_Os02g10800	mitochondrial carrier protein, putative, expressed
LOC_Os02g10810	protein of unknown function domain containing protein, expressed
LOC_Os02g10820	Sel1 repeat domain containing protein, putative, expressed
LOC_Os02g10830	serine acetyltransferase protein, putative, expressed
LOC_Os02g10850	OsFBK3 - F-box domain and kelch repeat containing protein, expressed
LOC_Os02g10860	bZIP transcription factor domain containing protein, expressed
LOC_Os02g10870	hypothetical protein
LOC_Os06g14750	phosphatidylinositol-4-phosphate 5-Kinase family protein, putative, expressec
LOC_Os10g10510	expressed protein
LOC_Os10g10530	expressed protein
LOC_Os10g10540	cysteine-rich repeat secretory protein precursor, putative, expressed

Do not distribute

ions

d

Do not distribute

Supp. Table S15. Aus seed loci used for identification of genes in networks for leaf rolling under d

[Rank]	[ORF_ID]	[Symbol]	[Score]	[Evidences]	[#_linked_(	[Linked_query]
1	LOC_Os04g	Os04g0129	11.25	DM-CX:0.3	4/7	LOC_Os02g45250 LOC_Os03g5
2	LOC_Os06g	Os06g0564	3.74	SC-LC:0.67	1/7	LOC_Os04g04020
3	LOC_Os03g	Os03g0786	3.68	DM-CX:1.0	1/7	LOC_Os04g04020
4	LOC_Os12g	Os12g0550	2.73	OS-CX:1.0(	1/7	LOC_Os09g23200
5	LOC_Os09g	Os09g0395	2.73	OS-CX:1.0(	1/7	LOC_Os12g36430
6	LOC_Os09g	Os09g0570	1.91	SC-GT:1.0(	1/7	LOC_Os04g04020
7	LOC_Os02g	Os02g0674	1.91	AT-CX:1.0(	1/7	LOC_Os04g04020

Do not distribute

drought

[GO_descriptions]
protein amino acid glycosylation;protein amino acid N-linked glycosylation;transport;intracellular protein transport;ER to Golgi vesicle-mediated transport;positive regulation of cell proliferation;protein transport;ER body organization
na
vegetative to reproductive phase transition of meristem;protein desumoylation;hydrogen peroxide biosynthetic process
na
transcription, DNA-dependent;regulation of transcription, DNA-dependent;multicellular organismal development;abaxial cell fate specification;inflorescence development;cell differentiation
metabolic process;oxidation reduction
regulation of transcription, DNA-dependent;anthocyanin accumulation in tissues in response to UV light;root development;transcription, DNA-dependent

Do not distribute

Supp. Table S16. Aus seed loci used for identification of genes in networks for leaf rolling

[Code]	[Rank]	[ORF_ID]	[Symbol]	[Score]	[Evidences]	[#_linked_c	[Linked_qu
LOC_Os02g	N	LOC_Os02g	Os02g0202300				
LOC_Os02g	N	LOC_Os02g	Os02g0202400				
LOC_Os02g	N	LOC_Os02g	Os02g0202500				
LOC_Os02g	N	LOC_Os02g	Os02g0202600				
LOC_Os02g	N	LOC_Os02g	na				
LOC_Os02g	N	LOC_Os02g	Os02g0202900				
LOC_Os02g	N	LOC_Os02g	Os02g0202950,Os02g0203000				
LOC_Os02g10870							
LOC_Os06g	N	LOC_Os06g	Os06g0259000				
LOC_Os10g	V	na	na				
LOC_Os10g	V	LOC_Os10g	na				
LOC_Os10g	V	LOC_Os10g	Os10g0184871				

Do not distribute

g under drought

[GO_descriptions]

na

transport;transmembrane transport;nucleotide transport

na

na

na

na

regulation of transcription, DNA-dependent

cellular protein metabolic process;phosphatidylinositol metabolic process;phosphoinositide phosphorylation;phosphorylation

na

protein amino acid phosphorylation

Do not distribute

Supp. Table S17. Loci from the aus panel predicted by RiceNet2 to be involved in networ

[Rank]	[ORF_ID]	[Paralogs]	[Symbol]	[score]	[Evidences(	[#_linked_query/#_val
1	LOC_Os11g	no_paralog	Os11g0433	25.59	SC-HT:0.19	1/7
2	LOC_Os11g	no_paralog	Os11g0482	21.94	HS-HT:0.23	2/7
3	LOC_Os08g	LOC_Os09g	Os08g0474	20.95	OS-CX:0.20	2/7
4	LOC_Os09g	LOC_Os08g	Os09g0460	16.58	SC-CC:0.24	1/7
5	LOC_Os07g	no_paralog	Os07g0246	12.35	SC-CC:0.33	1/7
6	LOC_Os01g	no_paralog	Os01g0179	12.01	SC-CC:0.30	1/7
7	LOC_Os02g	no_paralog	Os02g0135	11.73	SC-CC:0.39	1/7
8	LOC_Os01g	no_paralog	Os01g0616	11.44	OS-CX:0.51	2/7
9	LOC_Os07g	no_paralog	Os07g0657	11.37	SC-CC:0.41	1/7
10	LOC_Os01g	no_paralog	Os01g0254	11.14	SC-CC:0.41	1/7
11	LOC_Os01g	no_paralog	Os01g0338	11.06	SC-CC:0.42	1/7
12	LOC_Os12g	no_paralog	Os12g0560	10.99	SC-CC:0.42	1/7
13	LOC_Os11g	no_paralog	Os11g0174	10.92	HS-CX:0.30	2/7
14	LOC_Os06g	no_paralog	Os06g0568	10.24	SC-GT:0.47	3/7
15	LOC_Os03g	LOC_Os07g	Os03g0831	9.5	SC-CC:0.42	1/7
16	LOC_Os11g	no_paralog	Os11g0707	9.35	SC-GT:0.49	2/7
17	LOC_Os07g	no_paralog	Os07g0101	9.35	SC-GT:0.49	2/7
18	LOC_Os06g	no_paralog	na	9.12	OS-GN:0.5	1/7
19	LOC_Os06g	no_paralog	Os06g0175	9.12	OS-GN:0.5	1/7
20	LOC_Os01g	no_paralog	Os01g0513	9.04	SC-GT:0.51	2/7
21	LOC_Os09g	no_paralog	na	8.93	DM-CX:1.0	2/7
22	LOC_Os09g	no_paralog	Os09g0326	8.93	DM-CX:1.0	2/7
23	LOC_Os06g	no_paralog	na	8.93	DM-CX:1.0	2/7
24	LOC_Os06g	no_paralog	Os06g0698	8.93	DM-CX:1.0	2/7
25	LOC_Os02g	no_paralog	Os02g0714	8.91	HS-LC:0.53	2/7
26	LOC_Os07g	no_paralog	Os07g0201	8.83	AT-CC:0.46	2/7
27	LOC_Os01g	no_paralog	Os01g0711	8.77	SC-GT:0.51	3/7
28	LOC_Os07g	no_paralog	Os07g0512	8.55	HS-LC:0.69	2/7
29	LOC_Os08g	no_paralog	Os08g0110	8.35	DM-CX:1.0	2/7
30	LOC_Os08g	LOC_Os04g	Os08g0191	8.34	HS-LC:0.74	2/7
31	LOC_Os02g	no_paralog	Os02g0209	8.28	SC-LC:0.32	3/7
32	LOC_Os08g	LOC_Os04g	Os08g0224	8.27	HS-HT:0.56	1/7
33	LOC_Os03g	no_paralog	Os03g0596	8.25	DM-CX:0.7	2/7
34	LOC_Os02g	no_paralog	Os02g0179	8.24	DM-CX:1.0	2/7
35	LOC_Os04g	LOC_Os08g	Os04g0624	8.2	HS-LC:0.73	2/7
36	LOC_Os02g	no_paralog	Os02g0524	8.15	DM-CX:1.0	2/7
37	LOC_Os12g	no_paralog	Os12g0535	8.13	DM-CX:1.0	2/7
38	LOC_Os08g	no_paralog	Os08g0517	7.9	DM-CX:1.0	2/7
39	LOC_Os06g	LOC_Os02g	Os06g0698	7.9	DM-CX:1.0	2/7
40	LOC_Os02g	no_paralog	Os02g0209	7.88	SC-GT:0.42	2/7
41	LOC_Os07g	no_paralog	Os07g0642	7.82	HS-LC:0.60	2/7
42	LOC_Os03g	no_paralog	Os03g0401	7.82	HS-LC:0.60	2/7
43	LOC_Os08g	no_paralog	Os08g0154	7.82	DM-CX:0.5	2/7
44	LOC_Os03g	no_paralog	Os03g0227	7.78	AT-CC:0.43	2/7

Do not distribute

45	LOC_Os02gLOC_Os04gOs02g0613	7.66	HS-LC:0.62	2/7
46	LOC_Os06gno_paralogOs06g0116	7.56	SC-GT:0.74	2/7
47	LOC_Os02gno_paralogOs02g0470	7.56	SC-GT:0.74	3/7
48	LOC_Os04gno_paralogOs04g0656	7.55	SC-CC:0.57	1/7
49	LOC_Os03gno_paralogOs03g0856	7.54	SC-GT:0.67	3/7
50	LOC_Os03gLOC_Os03gOs03g0276	7.45	SC-LC:0.75	2/7
51	LOC_Os05gLOC_Os01gOs05g0460	7.45	SC-LC:0.75	2/7
52	LOC_Os07gno_paralogOs07g0661	7.38	SC-GT:0.62	2/7
53	LOC_Os04gLOC_Os02gOs04g0507	7.34	DM-CX:1.0	2/7
54	LOC_Os01gLOC_Os05gOs01g0840	7.33	SC-LC:0.76	2/7
55	LOC_Os01gno_paralogOs01g0371	7.26	CE-CC:0.42	2/7
56	LOC_Os04gLOC_Os02gOs04g0503	7.23	HS-LC:0.64	2/7
57	LOC_Os06gno_paralogOs06g0225	7.23	SC-CC:0.59	1/7
58	LOC_Os11gLOC_Os12gOs11g0703	7.23	SC-LC:0.76	2/7
59	LOC_Os12gLOC_Os03gOs12g0638	7.21	SC-CC:0.58	1/7
60	LOC_Os03gLOC_Os03gOs03g0821	7.21	SC-LC:0.76	2/7
61	LOC_Os04gno_paralogOs04g0117	7.19	CE-CX:0.36	2/7
62	LOC_Os07gno_paralogOs07g0191	7.19	SC-CC:0.58	1/7
63	LOC_Os03gLOC_Os12gOs03g0689	7.18	SC-CC:0.58	1/7
64	LOC_Os03gno_paralogOs03g0118	7.18	SC-HT:0.66	2/7
65	LOC_Os05gno_paralogOs05g0319	7.15	SC-CC:0.58	1/7
66	LOC_Os01gno_paralogOs01g0370	7.11	CE-CC:0.43	2/7
67	LOC_Os01gno_paralogOs01g0281	7.09	HS-CX:0.40	1/7
68	LOC_Os03gno_paralogOs03g0277	7.07	SC-LC:0.76	2/7
69	LOC_Os03gno_paralogOs03g0780	7.07	HS-HT:0.49	2/7
70	LOC_Os06gLOC_Os02gOs06g0671	7.06	HS-HT:0.49	2/7
71	LOC_Os02gLOC_Os06gOs02g0797	7.06	SC-CC:0.58	1/7
72	LOC_Os02gLOC_Os06gOs02g0167	7.06	HS-HT:0.49	2/7
73	LOC_Os03gno_paralogOs03g0399	7.03	HS-CX:0.72	2/7
74	LOC_Os02gno_paralogOs02g0497	7.02	DM-CX:1.0	2/7
75	LOC_Os03gno_paralogOs03g0661	7.01	HS-HT:0.47	2/7
76	LOC_Os03gno_paralogOs03g0100	7	SC-CC:0.58	1/7
77	LOC_Os07gLOC_Os03gOs07g0626	6.98	DM-CX:1.0	2/7
78	LOC_Os01gno_paralogOs01g0805	6.97	HS-HT:0.48	2/7
79	LOC_Os01gno_paralogOs01g0282	6.97	HS-HT:0.48	2/7
80	LOC_Os05gno_paralogOs05g0413	6.96	HS-HT:0.48	2/7
81	LOC_Os03gno_paralogOs03g0105	6.96	HS-HT:0.48	2/7
82	LOC_Os06gLOC_Os02gOs06g0181	6.94	SC-CC:0.58	1/7
83	LOC_Os01gLOC_Os05gOs01g0369	6.94	CE-CC:0.44	2/7
84	LOC_Os03gno_paralogOs03g0183	6.91	SC-CC:0.58	1/7
85	LOC_Os06gLOC_Os02gOs06g0562	6.88	OS-CX:0.54	3/7
86	LOC_Os03gno_paralogOs03g0791	6.78	SC-CC:0.50	1/7
87	LOC_Os06gno_paralogOs06g0198	6.78	SC-CC:0.50	1/7
88	LOC_Os04gLOC_Os08gOs04g0608	6.77	HS-HT:0.58	1/7
89	LOC_Os09gno_paralogOs09g0515	6.74	SC-GT:1.00	2/7
90	LOC_Os06gno_paralogOs06g0319	6.66	DM-CX:1.0	2/7

Do not distribute

91	LOC_Os12g	no_paralog	Os12g0165	6.57	SC-LC:0.51	1/7
92	LOC_Os01g	no_paralog	Os01g0902	6.55	AT-CX:1.00	3/7
93	LOC_Os03g	no_paralog	Os03g0646	6.49	DM-HT:0.4	2/7
94	LOC_Os10g	no_paralog	Os10g0575	6.49	DM-CX:1.0	2/7
95	LOC_Os04g	LOC_Os01g	Os04g0627	6.38	AT-CC:0.71	2/7
96	LOC_Os02g	LOC_Os06g	Os02g0768	6.23	SC-LC:0.53	1/7
97	LOC_Os08g	no_paralog	Os08g0320	6.2	OS-CX:1.00	2/7
98	LOC_Os03g	no_paralog	Os03g0747	6.17	SC-LC:0.56	1/7
99	LOC_Os03g	no_paralog	Os03g0644	6.17	SC-GT:1.00	2/7
100	LOC_Os03g	no_paralog	Os03g0289	6.16	SC-CC:0.60	1/7
101	LOC_Os09g	no_paralog	na	6.14	DM-CX:1.0	2/7
102	LOC_Os05g	no_paralog	Os05g0519	6.07	SC-CC:0.70	2/7
103	LOC_Os01g	no_paralog	Os01g0862	6.06	SC-GT:1.00	2/7
104	LOC_Os03g	LOC_Os12g	Os03g0654	6.03	SC-LC:0.60	1/7
105	LOC_Os10g	no_paralog	Os10g0437	6.03	SC-LC:0.60	1/7
106	LOC_Os12g	no_paralog	Os12g0166	6.02	SC-LC:0.52	1/7
107	LOC_Os02g	no_paralog	Os02g0741	6.02	DM-CX:1.0	2/7
108	LOC_Os07g	no_paralog	Os07g0564	5.98	OS-CX:0.58	2/7
109	LOC_Os10g	no_paralog	Os10g0399	5.88	OS-GN:0.6	2/7
110	LOC_Os09g	no_paralog	Os09g0127	5.87	OS-CX:0.53	1/7
111	LOC_Os01g	no_paralog	Os01g0681	5.87	SC-GT:1.00	2/7
112	LOC_Os09g	no_paralog	Os09g0552	5.87	DM-CX:1.0	2/7
113	LOC_Os03g	no_paralog	Os03g0748	5.87	DM-CX:1.0	2/7
114	LOC_Os03g	no_paralog	Os03g0826	5.86	SC-GT:1.00	2/7
115	LOC_Os05g	no_paralog	Os05g0163	5.83	DM-CX:1.0	2/7
116	LOC_Os10g	no_paralog	Os10g0399	5.82	OS-GN:0.6	2/7
117	LOC_Os12g	no_paralog	Os12g0625	5.82	SC-LC:0.56	1/7
118	LOC_Os06g	LOC_Os02g	Os06g0210	5.8	SC-LC:0.53	1/7
119	LOC_Os01g	no_paralog	Os01g0585	5.78	SC-LC:0.57	1/7
120	LOC_Os03g	no_paralog	Os03g0258	5.74	SC-GT:1.00	2/7
121	LOC_Os05g	no_paralog	Os05g0436	5.73	OS-CX:1.00	2/7
122	LOC_Os02g	no_paralog	Os02g0749	5.72	DM-CX:0.5	2/7
123	LOC_Os04g	LOC_Os08g	Os04g0605	5.71	SC-LC:0.51	1/7
124	LOC_Os10g	no_paralog	Os10g0363	5.69	DM-CX:1.0	2/7
125	LOC_Os12g	LOC_Os03g	Os12g0623	5.67	SC-LC:0.60	1/7
126	LOC_Os07g	no_paralog	Os07g0209	5.64	SC-GT:1.00	2/7
127	LOC_Os01g	no_paralog	Os01g0234	5.62	SC-LC:0.51	1/7
128	LOC_Os10g	LOC_Os04g	Os10g0561	5.61	AT-CC:0.67	1/7
129	LOC_Os03g	LOC_Os12g	Os03g0616	5.6	DM-HT:0.5	2/7
130	LOC_Os03g	no_paralog	Os03g0203	5.6	DM-HT:0.5	2/7
131	LOC_Os01g	no_paralog	Os01g0708	5.58	SC-LC:1.00	2/7
132	LOC_Os11g	no_paralog	Os11g0247	5.58	SC-HT:0.56	2/7
133	LOC_Os02g	LOC_Os02g	Os02g0617	5.53	DM-CX:1.0	2/7
134	LOC_Os01g	no_paralog	Os01g0978	5.52	SC-LC:0.56	1/7
135	LOC_Os08g	LOC_Os04g	Os08g0234	5.51	SC-LC:0.51	1/7
136	LOC_Os08g	no_paralog	na	5.5	SC-LC:0.51	1/7

Do not distribute

137	LOC_Os05ɡno_paralogOs05g0527	5.48	CE-CC:0.51	1/7
138	LOC_Os01ɡno_paralogOs01g0734	5.48	CE-CC:0.51	1/7
139	LOC_Os04ɡLOC_Os04ɡOs04g0319	5.48	CE-CC:0.51	1/7
140	LOC_Os01ɡno_paralogOs01g0735	5.48	CE-CC:0.51	1/7
141	LOC_Os01ɡno_paralogOs01g0638	5.48	CE-CC:0.51	1/7
142	LOC_Os10ɡno_paralogOs10g0442	5.48	CE-CC:0.51	1/7
143	LOC_Os09ɡno_paralogOs09g0482	5.48	CE-CC:0.51	1/7
144	LOC_Os02ɡLOC_Os04ɡOs02g0634	5.48	CE-CC:0.51	1/7
145	LOC_Os04ɡno_paralogOs04g0206	5.48	CE-CC:0.51	1/7
146	LOC_Os11ɡno_paralogOs11g0461	5.48	CE-CC:0.51	1/7
147	LOC_Os02ɡno_paralogOs02g0578	5.48	CE-CC:0.51	1/7
148	LOC_Os05ɡno_paralogOs05g0527	5.48	CE-CC:0.51	1/7
149	LOC_Os07ɡno_paralogOs07g0489	5.48	CE-CC:0.51	1/7
150	LOC_Os02ɡno_paralogOs02g0242	5.48	CE-CC:0.51	1/7
151	LOC_Os03ɡno_paralogOs03g0726	5.48	SC-HT:0.5[illegible]	2/7
152	LOC_Os01ɡno_paralogOs01g0374	5.44	CE-CC:0.5[illegible]	2/7
153	LOC_Os04ɡno_paralogOs04g0326	5.44	CE-CC:0.51	1/7
154	LOC_Os02ɡno_paralogOs02g0767	5.38	AT-CX:0.6[illegible]	2/7
155	LOC_Os08ɡno_paralogOs08g0564	5.36	OS-CX:1.0[illegible]	2/7
156	LOC_Os11ɡno_paralogOs11g0220	5.36	SC-LC:0.54	1/7
157	LOC_Os06ɡLOC_Os02ɡOs06g0699	5.35	HS-HT:0.7[illegible]	1/7
158	LOC_Os07ɡno_paralogOs07g0574	5.35	SC-HT:0.5[illegible]	2/7
159	LOC_Os02ɡno_paralogOs02g0137	5.34	DM-CX:0.6	2/7
160	LOC_Os05ɡno_paralogOs05g0527	5.31	CE-CC:0.5[illegible]	1/7
161	LOC_Os03ɡno_paralogOs03g0290	5.29	SC-GT:1.0[illegible]	2/7
162	LOC_Os11ɡno_paralogOs11g0309	5.29	SC-GT:1.0[illegible]	2/7
163	LOC_Os09ɡno_paralogOs09g0326	5.29	HS-HT:0.7[illegible]	1/7
164	LOC_Os02ɡLOC_Os06ɡOs02g0146	5.29	HS-HT:0.6[illegible]	1/7
165	LOC_Os04ɡno_paralogOs04g0509	5.27	HS-CX:0.5[illegible]	1/7
166	LOC_Os12ɡno_paralogOs12g0207	5.27	OS-CX:1.0[illegible]	2/7
167	LOC_Os04ɡno_paralogOs04g0319	5.23	CE-CC:0.5[illegible]	1/7
168	LOC_Os04ɡLOC_Os04ɡOs04g0326	5.22	CE-CC:0.5[illegible]	1/7
169	LOC_Os03ɡno_paralogOs03g0841	5.21	CE-CC:0.5[illegible]	1/7
170	LOC_Os02ɡno_paralogOs02g0243	5.19	CE-CC:0.5[illegible]	1/7
171	LOC_Os01ɡno_paralogOs01g0915	5.19	DM-CX:0.5	2/7
172	LOC_Os03ɡno_paralogOs03g0101	5.17	SC-GT:1.0[illegible]	2/7
173	LOC_Os09ɡLOC_Os02ɡOs09g0453	5.16	SC-LC:0.55	1/7
174	LOC_Os09ɡno_paralogOs09g0491	5.16	SC-LC:0.48	1/7
175	LOC_Os07ɡno_paralog	na	5.16	CE-CC:0.5[illegible]	1/7
176	LOC_Os07ɡno_paralog	na	5.16	CE-CC:0.5[illegible]	1/7
177	LOC_Os05ɡno_paralogOs05g0530	5.16	SC-GT:1.0[illegible]	2/7
178	LOC_Os12ɡLOC_Os03ɡOs12g0586	5.13	DM-HT:0.[illegible]	2/7
179	LOC_Os04ɡno_paralogOs04g0324	5.12	CE-CC:0.5[illegible]	1/7
180	LOC_Os04ɡno_paralogOs04g0165	5.12	SC-LC:0.57	1/7
181	LOC_Os11ɡno_paralogOs11g0457	5.11	CE-CC:0.5[illegible]	1/7
182	LOC_Os01ɡLOC_Os01ɡOs01g0754	5.11	OS-CX:0.5[illegible]	1/7

Do not distribute

183	LOC_Os09g no_paralog Os09g0557	5.11	OS-CX:0.55	1/7
184	LOC_Os02g no_paralog Os02g0126	5.11	SC-GT:0.50	2/7
185	LOC_Os05g no_paralog Os05g0535	5.1	SC-HT:0.63	2/7
186	LOC_Os08g no_paralog Os08g0450	5.09	HS-CX:1.00	1/7
187	LOC_Os04g no_paralog Os04g0314	5.07	CE-CC:0.54	1/7
188	LOC_Os07g no_paralog Os07g0486	5.06	CE-CC:0.54	1/7
189	LOC_Os12g no_paralog Os12g0633	5.06	SC-LC:0.62	1/7
190	LOC_Os01g no_paralog Os01g0814	5.05	SC-LC:0.57	1/7
191	LOC_Os02g no_paralog na	5.05	CE-CC:0.54	1/7
192	LOC_Os07g no_paralog Os07g0489	5.02	CE-CC:0.54	1/7
193	LOC_Os01g no_paralog Os01g0371	5.02	CE-CC:0.57	2/7
194	LOC_Os04g LOC_Os06g Os04g0451	5.02	CE-CC:0.54	1/7
195	LOC_Os05g no_paralog Os05g0169	5.01	SC-LC:0.55	1/7
196	LOC_Os01g no_paralog Os01g0638	5.01	CE-CC:0.54	1/7
197	LOC_Os03g no_paralog Os03g0197	5.01	SC-GT:0.58	1/7
198	LOC_Os07g no_paralog Os07g0489	5.01	CE-CC:0.54	1/7
199	LOC_Os07g no_paralog Os07g0490	4.99	CE-CC:0.55	1/7
200	LOC_Os01g no_paralog Os01g0370	4.99	CE-CC:0.57	2/7
201	LOC_Os02g no_paralog Os02g0577	4.97	CE-CC:0.55	1/7
202	LOC_Os05g no_paralog Os05g0494	4.97	SC-LC:0.60	1/7
203	LOC_Os01g no_paralog Os01g0597	4.97	CE-CC:0.55	1/7
204	LOC_Os03g no_paralog Os03g0306	4.96	SC-GT:1.00	2/7
205	LOC_Os01g no_paralog Os01g0764	4.94	CE-CC:0.56	2/7
206	LOC_Os08g no_paralog Os08g0404	4.94	CE-CC:0.55	1/7
207	LOC_Os04g LOC_Os02g Os04g0569	4.93	AT-CC:0.63	1/7
208	LOC_Os01g no_paralog Os01g0772	4.93	DM-CX:1.0	1/7
209	LOC_Os08g no_paralog Os08g0528	4.92	CE-CX:1.00	1/7
210	LOC_Os08g LOC_Os09g Os08g0470	4.92	HS-LC:0.53	1/7
211	LOC_Os02g LOC_Os06g Os02g0149	4.91	SC-GT:1.00	2/7
212	LOC_Os02g no_paralog Os02g0554	4.9	SC-GT:1.00	2/7
213	LOC_Os01g no_paralog Os01g0371	4.9	CE-CC:0.57	2/7
214	LOC_Os06g no_paralog Os06g0608	4.87	SC-GT:1.00	1/7
215	LOC_Os07g no_paralog Os07g0277	4.85	OS-CX:1.00	2/7
216	LOC_Os10g no_paralog Os10g0578	4.85	SC-GT:0.60	1/7
217	LOC_Os02g LOC_Os06g Os02g0149	4.84	DM-CX:1.0	2/7
218	LOC_Os01g no_paralog Os01g0512	4.83	SC-GT:1.00	1/7
219	LOC_Os03g LOC_Os10g Os03g0209	4.82	SC-GT:1.00	1/7
220	LOC_Os06g no_paralog Os06g0564	4.81	SC-LC:0.58	1/7
221	LOC_Os11g LOC_Os12g Os11g0169	4.79	SC-GT:1.00	2/7
222	LOC_Os02g LOC_Os11g Os02g0550	4.79	SC-GT:1.00	2/7
223	LOC_Os12g LOC_Os11g Os12g0168	4.79	SC-GT:1.00	2/7
224	LOC_Os02g no_paralog Os02g0633	4.78	HS-LC:0.58	2/7
225	LOC_Os06g no_paralog Os06g0149	4.78	SC-LC:0.58	1/7
226	LOC_Os11g LOC_Os12g Os11g0137	4.77	SC-LC:0.64	1/7
227	LOC_Os01g no_paralog Os01g0777	4.76	DM-CX:0.5	2/7
228	LOC_Os01g no_paralog Os01g0973	4.76	SC-GT:1.00	1/7

Do not distribute

229	LOC_Os03gno_paralogOs03g0228	4.76	SC-GT:1.0C	1/7
230	LOC_Os11gno_paralogOs11g0439	4.76	SC-GT:1.0C	1/7
231	LOC_Os10gLOC_Os03gOs10g0337	4.74	SC-GT:1.0C	1/7
232	LOC_Os05gno_paralogOs05g0302	4.73	HS-LC:0.61	1/7
233	LOC_Os05gLOC_Os01gOs05g0148	4.73	CE-CC:0.58	2/7
234	LOC_Os06gno_paralogOs06g0564	4.73	SC-LC:0.58	1/7
235	LOC_Os12gno_paralogOs12g0554	4.72	HS-LC:1.0C	1/7
236	LOC_Os07gno_paralogOs07g0454	4.72	HS-LC:1.0C	1/7
237	LOC_Os06gno_paralogOs06g0151	4.72	HS-LC:1.0C	1/7
238	LOC_Os10gno_paralogOs10g0498	4.72	HS-LC:1.0C	1/7
239	LOC_Os05gLOC_Os01gOs05g0106	4.72	SC-GT:1.0C	2/7
240	LOC_Os06gno_paralogOs06g0149	4.72	SC-LC:0.58	1/7
241	LOC_Os12gno_paralogOs12g0613	4.71	DM-CX:0.5	1/7
242	LOC_Os10gLOC_Os03gOs10g0170	4.71	HS-HT:1.0(	1/7
243	LOC_Os11gno_paralogOs11g0682	4.7	OS-CX:1.0(	2/7
244	LOC_Os10gno_paralogOs10g0400	4.69	HS-CX:0.59	2/7
245	LOC_Os02gLOC_Os04gOs02g0537	4.69	SC-CC:0.53	1/7
246	LOC_Os01gno_paralogOs01g0372	4.68	CE-CC:0.59	2/7
247	LOC_Os05gLOC_Os01gOs05g0437	4.68	HS-LC:0.58	2/7
248	LOC_Os09gno_paralogOs09g0244	4.67	HS-LC:1.0C	1/7
249	LOC_Os07gLOC_Os03gOs07g0603	4.67	SC-GT:1.0C	2/7
250	LOC_Os02gLOC_Os06gOs02g0222	4.67	SC-LC:0.59	1/7
251	LOC_Os04gno_paralogOs04g0162	4.66	DM-CX:1.0	1/7
252	LOC_Os07gLOC_Os03gOs07g0632	4.64	SC-LC:0.58	1/7
253	LOC_Os02gLOC_Os04gOs02g0646	4.64	SC-LC:0.57	2/7
254	LOC_Os03gno_paralogOs03g0177	4.63	SC-LC:0.53	2/7
255	LOC_Os03gno_paralogOs03g0177	4.63	SC-LC:0.53	2/7
256	LOC_Os03gno_paralogOs03g0177	4.63	SC-LC:0.53	2/7
257	LOC_Os01gno_paralogOs01g0353	4.63	CE-CC:0.6C	2/7
258	LOC_Os01gno_paralogOs01g0570	4.62	HS-LC:1.0C	1/7
259	LOC_Os02gno_paralogOs02g0635	4.62	CE-CC:1.0C	1/7
260	LOC_Os01gno_paralogOs01g0567	4.61	SC-CC:0.6C	1/7
261	LOC_Os03gno_paralogOs03g0594	4.61	SC-CC:0.6C	1/7
262	LOC_Os06gno_paralogOs06g0135	4.61	SC-CC:1.0C	1/7
263	LOC_Os08gLOC_Os09gOs08g0430	4.6	DM-CX:1.0	2/7
264	LOC_Os07gno_paralogOs07g0291	4.6	HS-LC:1.0C	1/7
265	LOC_Os11gno_paralogOs11g0472	4.6	CE-CX:1.0C	1/7
266	LOC_Os03gno_paralogOs03g0417	4.59	SC-GT:1.0C	2/7
267	LOC_Os06gno_paralogOs06g0264	4.59	DM-CX:1.0	2/7
268	LOC_Os02gno_paralogOs02g0313	4.57	DM-CX:0.6	2/7
269	LOC_Os07gno_paralogOs07g0171	4.57	DM-HT:0.6	1/7
270	LOC_Os01gno_paralogOs01g0916	4.56	DM-CX:1.0	1/7
271	LOC_Os01gno_paralogOs01g0933	4.54	CE-CC:0.6C	2/7
272	LOC_Os08gno_paralogOs08g0169	4.53	HS-HT:1.0(	1/7
273	LOC_Os11gno_paralogOs11g0440	4.53	SC-GT:1.0C	1/7
274	LOC_Os01gLOC_Os05gOs01g0109	4.53	OS-CX:0.53	2/7

Do not distribute

275	LOC_Os03gno_paralogOs03g0711	4.52	CE-CX:0.60	1/7
276	LOC_Os03gLOC_Os07gOs03g0376	4.52	SC-LC:0.59	1/7
277	LOC_Os02gno_paralogOs02g0718	4.52	HS-LC:0.68	1/7
278	LOC_Os01gno_paralogOs01g0977	4.52	HS-CX:1.00	1/7
279	LOC_Os01gno_paralogOs01g0166	4.51	HS-LC:1.00	1/7
280	LOC_Os02gno_paralogOs02g0172	4.51	OS-CX:1.00	2/7
281	LOC_Os09gLOC_Os08gOs09g0407	4.5	DM-CX:1.0	2/7
282	LOC_Os11gLOC_Os12gOs11g0158	4.5	AT-CC:1.00	1/7
283	LOC_Os01gno_paralogOs01g0974	4.49	OS-CX:1.00	1/7
284	LOC_Os06gno_paralogOs06g0300	4.49	SC-GT:1.00	1/7
285	LOC_Os01gno_paralogOs01g0511	4.48	AT-CC:1.00	1/7
286	LOC_Os04gno_paralogOs04g0502	4.48	HS-LC:1.00	1/7
287	LOC_Os03gLOC_Os06gOs03g0410	4.47	AT-CC:1.00	1/7
288	LOC_Os05gno_paralogOs05g0295	4.46	DM-CX:1.0	2/7
289	LOC_Os01gno_paralogOs01g0666	4.45	DM-CX:1.0	1/7
290	LOC_Os03gno_paralogOs03g0744	4.45	HS-LC:1.00	1/7
291	LOC_Os01gno_paralogOs01g0567	4.42	SC-CC:0.60	1/7
292	LOC_Os08gno_paralogOs08g0470	4.42	HS-LC:0.59	1/7
293	LOC_Os05gno_paralogOs05g0188	4.41	HS-CX:0.56	1/7
294	LOC_Os04gno_paralogOs04g0643	4.41	SC-GT:1.00	2/7
295	LOC_Os06gno_paralogOs06g0344	4.41	OS-CX:1.00	2/7
296	LOC_Os05gLOC_Os01gOs05g0438	4.4	AT-CX:1.00	1/7
297	LOC_Os06gno_paralogOs06g0159	4.4	AT-CX:1.00	1/7
298	LOC_Os06gno_paralogOs06g0160	4.4	AT-CX:1.00	1/7
299	LOC_Os11gno_paralogOs11g0155	4.4	AT-CX:1.00	1/7
300	LOC_Os01gLOC_Os04gOs01g0866	4.4	AT-CX:1.00	1/7
301	LOC_Os01gLOC_Os05gOs01g0308	4.37	AT-CC:1.00	1/7
302	LOC_Os03gno_paralogOs03g0593	4.37	OS-CX:1.00	2/7
303	LOC_Os07gno_paralogOs07g0237	4.36	SC-LC:0.63	1/7
304	LOC_Os01gno_paralogOs01g0267	4.35	SC-LC:0.62	1/7
305	LOC_Os05gno_paralogOs05g0371	4.34	SC-LC:0.62	1/7
306	LOC_Os08gLOC_Os04gOs08g0170	4.33	HS-LC:0.70	1/7
307	LOC_Os01gno_paralogOs01g0963	4.32	SC-GT:1.00	1/7
308	LOC_Os08gno_paralogOs08g0323	4.32	OS-CX:1.00	1/7
309	LOC_Os06gLOC_Os02gOs06g0625	4.31	HS-HT:1.00	1/7
310	LOC_Os02gno_paralogOs02g0452	4.31	SC-CC:1.00	1/7
311	LOC_Os07gno_paralogOs07g0555	4.31	DM-CX:1.0	1/7
312	LOC_Os08gno_paralogOs08g0328	4.29	OS-CX:1.00	2/7
313	LOC_Os02gno_paralogOs02g0194	4.28	DR-CX:0.56	2/7
314	LOC_Os06gno_paralogOs06g0163	4.28	OS-CX:1.00	2/7
315	LOC_Os07gno_paralogOs07g0598	4.27	AT-CC:1.00	1/7
316	LOC_Os01gno_paralogOs01g0749	4.27	AT-CC:1.00	1/7
317	LOC_Os08gLOC_Os09gOs08g0465	4.27	AT-CC:1.00	1/7
318	LOC_Os07gno_paralogOs07g0417	4.27	AT-CC:1.00	1/7
319	LOC_Os07gno_paralogOs07g0661	4.27	AT-CC:1.00	1/7
320	LOC_Os11gLOC_Os12gOs11g0141	4.27	HS-HT:1.00	1/7

Do not distribute

321	LOC_Os12gLOC_Os11gOs12g0137	4.27	HS-HT:1.0(	1/7
322	LOC_Os06gno_paralogOs06g0242	4.26	OS-GN:1.0	1/7
323	LOC_Os11gno_paralogOs11g0126	4.25	SC-GT:1.0(	1/7
324	LOC_Os01gno_paralogOs01g0273	4.25	HS-HT:1.0(	1/7
325	LOC_Os03gLOC_Os10gOs03g0249	4.24	HS-HT:1.0(	1/7
326	LOC_Os06gno_paralogOs06g0205	4.23	HS-LC:1.00	1/7
327	LOC_Os12gLOC_Os11gOs12g0123	4.23	SC-GT:1.0(	1/7
328	LOC_Os09gLOC_Os08gOs09g0449	4.22	DM-CX:1.0	1/7
329	LOC_Os03gno_paralogOs03g0663	4.22	OS-CX:1.0(	2/7
330	LOC_Os11gno_paralogOs11g0126	4.22	SC-GT:1.0(	1/7
331	LOC_Os10gno_paralogOs10g0492	4.22	AT-LC:1.00	1/7
332	LOC_Os04gno_paralogOs04g0439	4.22	AT-LC:1.00	1/7
333	LOC_Os07gno_paralogOs07g0290	4.22	AT-LC:1.00	1/7
334	LOC_Os09gno_paralogOs09g0541	4.21	HS-LC:1.00	1/7
335	LOC_Os07gno_paralogOs07g0540	4.18	OS-CX:1.0(	2/7
336	LOC_Os11gno_paralogOs11g0538	4.18	DR-CX:0.5(	2/7
337	LOC_Os04gLOC_Os02gOs04g0252	4.17	SC-CC:1.0(	1/7
338	LOC_Os03gno_paralogOs03g0239	4.16	AT-CC:1.0(	1/7
339	LOC_Os01gLOC_Os02gOs01g0203	4.16	AT-CC:1.0(	1/7
340	LOC_Os08gno_paralogOs08g0383	4.16	AT-CC:1.0(	1/7
341	LOC_Os10gLOC_Os02gOs10g0555	4.16	AT-CC:1.0(	1/7
342	LOC_Os08gno_paralogOs08g0513	4.16	AT-CC:1.0(	1/7
343	LOC_Os03gLOC_Os07gOs03g0336	4.16	AT-CC:1.0(	1/7
344	LOC_Os10gno_paralogOs10g0575	4.16	AT-CC:1.0(	1/7
345	LOC_Os02gno_paralogOs02g0496	4.16	AT-CC:1.0(	1/7
346	LOC_Os09gno_paralogOs09g0527	4.16	AT-CC:1.0(	1/7
347	LOC_Os07gno_paralogOs07g0679	4.16	AT-CC:1.0(	1/7
348	LOC_Os04gno_paralogOs04g0162	4.16	AT-CC:1.0(	1/7
349	LOC_Os06gno_paralogOs06g0130	4.16	AT-CC:1.0(	1/7
350	LOC_Os06gno_paralogOs06g0207	4.16	AT-CC:1.0(	1/7
351	LOC_Os02gno_paralogOs02g0635	4.16	CE-CC:1.0(	1/7
352	LOC_Os08gLOC_Os04gOs08g0187	4.14	AT-CC:1.0(	1/7
353	LOC_Os03gno_paralogOs03g0181	4.14	AT-CC:1.0(	1/7
354	LOC_Os05gno_paralogOs05g0230	4.12	OS-CX:0.5:	2/7
355	LOC_Os02gLOC_Os04gOs02g0663	4.12	HS-HT:1.0(	1/7
356	LOC_Os08gno_paralogOs08g0557	4.12	HS-HT:1.0(	1/7
357	LOC_Os12gno_paralogOs12g0628	4.12	HS-HT:1.0(	1/7
358	LOC_Os07gno_paralogOs07g0559	4.11	SC-CC:0.6(	1/7
359	LOC_Os08gno_paralogOs08g0178	4.1	SC-CC:0.6(	1/7
360	LOC_Os11gLOC_Os12gOs11g0137	4.1	HS-CX:1.0(	1/7
361	LOC_Os12gLOC_Os11gOs12g0134	4.09	SC-LC:0.66	1/7
362	LOC_Os03gLOC_Os12gOs03g0642	4.08	DM-CX:1.0	1/7
363	LOC_Os07gno_paralogOs07g0106	4.08	SC-CC:0.6(	1/7
364	LOC_Os02gno_paralogOs02g0117	4.08	HS-LC:1.00	1/7
365	LOC_Os01gno_paralogOs01g0695	4.08	AT-CC:1.0(	1/7
366	LOC_Os01gno_paralogOs01g0131	4.08	AT-CC:1.0(	1/7

Do not distribute

367	LOC_Os02gno_paralogOs02g0228	4.08	AT-CC:1.0(	1/7
368	LOC_Os03gLOC_Os10gOs03g0231	4.08	AT-CC:1.0(	1/7
369	LOC_Os02gno_paralogOs02g0823	4.08	AT-CC:1.0(	1/7
370	LOC_Os05gno_paralogOs05g0408	4.08	AT-CC:1.0(	1/7
371	LOC_Os12gLOC_Os03gOs12g0636	4.08	AT-CC:1.0(	1/7
372	LOC_Os03gno_paralogOs03g0445	4.08	AT-CC:1.0(	1/7
373	LOC_Os09gLOC_Os08gOs09g0558	4.08	AT-CC:1.0(	1/7
374	LOC_Os04gno_paralogOs04g0669	4.08	AT-CC:1.0(	1/7
375	LOC_Os01gno_paralogOs01g0288	4.08	AT-CC:1.0(	1/7
376	LOC_Os03gLOC_Os03gOs03g0680	4.08	AT-CC:1.0(	1/7
377	LOC_Os04gno_paralogOs04g0675	4.07	DR-CX:0.5:	1/7
378	LOC_Os10gno_paralogOs10g0486	4.06	HS-HT:1.0(	1/7
379	LOC_Os01gno_paralogOs01g0200	4.06	HS-LC:1.00	1/7
380	LOC_Os08gLOC_Os02gOs08g0469	4.04	SC-LC:0.63	1/7
381	LOC_Os07gLOC_Os03gOs07g0166	4.03	HS-HT:1.0(	1/7
382	LOC_Os02gLOC_Os06gOs02g0102	4.02	OS-GN:1.0	2/7
383	LOC_Os12gno_paralogOs12g0277	4.02	OS-GN:1.0	2/7
384	LOC_Os10gLOC_Os03gOs10g0462	4.02	OS-GN:1.0	2/7
385	LOC_Os09gno_paralogOs09g0563	4.02	OS-GN:1.0	2/7
386	LOC_Os03gLOC_Os10gOs03g0143	4.02	OS-GN:1.0	2/7
387	LOC_Os05gno_paralogOs05g0540	4.02	OS-GN:1.0	2/7
388	LOC_Os06gLOC_Os02gOs06g0114	4.02	OS-GN:1.0	2/7
389	LOC_Os03gno_paralogOs03g0859	4.02	OS-GN:1.0	2/7
390	LOC_Os08gno_paralogOs08g0107	4.01	SC-GT:1.0(	2/7
391	LOC_Os03gLOC_Os07gOs03g0791	4.01	HS-HT:1.0(	1/7
392	LOC_Os03gno_paralogOs03g0328	4.01	SC-LC:0.63	1/7
393	LOC_Os07gLOC_Os03gOs07g0640	4	AT-CC:1.0(	1/7
394	LOC_Os09gLOC_Os08gOs09g0532	4	AT-CC:1.0(	1/7
395	LOC_Os02gLOC_Os04gOs02g0170	4	AT-CC:1.0(	1/7
396	LOC_Os01gno_paralogOs01g0286	4	AT-CC:1.0(	1/7
397	LOC_Os05gno_paralogOs05g0286	4	AT-CC:1.0(	1/7
398	LOC_Os05gno_paralogOs05g0182	3.98	HS-HT:1.0(	1/7
399	LOC_Os10gno_paralogOs10g0399	3.97	OS-GN:1.0	1/7
400	LOC_Os10gno_paralogOs10g0546	3.97	AT-CX:1.0(	2/7
401	LOC_Os03gno_paralogOs03g0702	3.96	OS-PG:0.6(	1/7
402	LOC_Os02gLOC_Os06gOs02g0192	3.96	HS-HT:1.0(	1/7
403	LOC_Os09gno_paralogOs09g0297	3.96	SC-CC:0.61	1/7
404	LOC_Os11gno_paralogOs11g0168	3.96	SC-GT:1.0(	1/7
405	LOC_Os01gno_paralogOs01g0782	3.95	SC-GT:0.54	1/7
406	LOC_Os05gno_paralogOs05g0104	3.95	HS-HT:1.0(	1/7
407	LOC_Os08gno_paralogOs08g0161	3.95	SC-GT:0.58	1/7
408	LOC_Os11gLOC_Os12gOs11g0133	3.94	OS-CX:1.0(	2/7
409	LOC_Os02gLOC_Os04gOs02g0610	3.93	AT-CC:1.0(	1/7
410	LOC_Os04gLOC_Os02gOs04g0497	3.93	AT-CC:1.0(	1/7
411	LOC_Os06gLOC_Os01gOs06g0335	3.93	AT-CC:1.0(	1/7
412	LOC_Os02gLOC_Os06gOs02g0775	3.93	AT-CC:1.0(	1/7

Do not distribute

413	LOC_Os01gLOC_Os03gOs01g0838	3.93	AT-CC:1.0(	1/7
414	LOC_Os02gno_paralogOs02g0189	3.93	AT-CC:1.0(	1/7
415	LOC_Os07gno_paralogOs07g0580	3.93	AT-CC:1.0(	1/7
416	LOC_Os02gLOC_Os10gOs02g0659	3.93	AT-CC:1.0(	1/7
417	LOC_Os04gLOC_Os08gOs04g0637	3.93	AT-CC:1.0(	1/7
418	LOC_Os04gno_paralogOs04g0618	3.93	AT-CC:1.0(	1/7
419	LOC_Os05gLOC_Os01gOs05g0497	3.93	AT-CC:1.0(	1/7
420	LOC_Os02gno_paralogOs02g0817	3.93	AT-CC:1.0(	1/7
421	LOC_Os03gLOC_Os03gOs03g0732	3.93	AT-CC:1.0(	1/7
422	LOC_Os06gLOC_Os02gOs06g0624	3.93	AT-CC:1.0(	1/7
423	LOC_Os04gLOC_Os02gOs04g0555	3.91	HS-HT:1.0(	1/7
424	LOC_Os01gno_paralogOs01g0916	3.91	AT-CX:1.0(	2/7
425	LOC_Os03gno_paralogOs03g0780	3.9	HS-HT:1.0(	1/7
426	LOC_Os03gno_paralogOs03g0214	3.9	OS-CX:0.5(	1/7
427	LOC_Os07gLOC_Os03gOs07g0682	3.9	SC-GT:1.0(	1/7
428	LOC_Os05gLOC_Os01gOs05g0358	3.89	AT-CC:1.0(	1/7
429	LOC_Os02gLOC_Os01gOs02g0235	3.89	CE-CX:1.0(	1/7
430	LOC_Os02gno_paralogOs02g0160	3.88	SC-CC:0.61	1/7
431	LOC_Os02gno_paralogOs02g0774	3.87	SC-LC:0.64	1/7
432	LOC_Os03gno_paralogOs03g0113	3.87	SC-LC:0.64	1/7
433	LOC_Os12gLOC_Os11gOs12g0133	3.87	HS-CX:1.0(	1/7
434	LOC_Os02gLOC_Os06gOs02g0226	3.87	AT-CC:1.0(	1/7
435	LOC_Os05gno_paralogOs05g0411	3.87	AT-CC:1.0(	1/7
436	LOC_Os06gLOC_Os01gOs06g0550	3.85	CE-CX:1.0(	1/7
437	LOC_Os05gLOC_Os01gOs05g0207	3.85	CE-CX:1.0(	1/7
438	LOC_Os03gno_paralogOs03g0376	3.85	OS-GN:1.0	1/7
439	LOC_Os01gno_paralogOs01g0383	3.85	SC-LC:0.64	1/7
440	LOC_Os08gno_paralogOs08g0248	3.85	SC-GT:1.0(	1/7
441	LOC_Os01gLOC_Os02gOs01g0205	3.84	CE-CX:1.0(	1/7
442	LOC_Os03gLOC_Os11gOs03g0328	3.83	SC-GT:1.0(	1/7
443	LOC_Os03gno_paralogOs03g0218	3.83	SC-CC:0.61	1/7
444	LOC_Os09gno_paralogOs09g0416	3.83	SC-CC:0.61	1/7
445	LOC_Os01gLOC_Os05gOs01g0649	3.82	SC-GT:1.0(	2/7
446	LOC_Os09gLOC_Os08gOs09g0447	3.82	AT-CC:1.0(	1/7
447	LOC_Os01gno_paralogOs01g0854	3.81	AT-CC:1.0(	1/7
448	LOC_Os04gno_paralogOs04g0648	3.81	AT-CC:1.0(	1/7
449	LOC_Os01gno_paralogOs01g0129	3.81	AT-CC:1.0(	1/7
450	LOC_Os02gno_paralogOs02g0167	3.81	DM-CX:1.0	1/7
451	LOC_Os03gLOC_Os10gOs03g0101	3.8	SC-CC:0.61	1/7
452	LOC_Os02gno_paralogOs02g0173	3.78	SC-GT:0.5(	1/7
453	LOC_Os01gno_paralogOs01g0177	3.78	HS-HT:1.0(	1/7
454	LOC_Os01gno_paralogOs01g0916	3.78	OS-CX:1.0(	1/7
455	LOC_Os10gLOC_Os03gOs10g0561	3.77	SC-CC:0.61	1/7
456	LOC_Os03gno_paralogOs03g0765	3.75	SC-GT:0.51	2/7
457	LOC_Os07gLOC_Os03gOs07g0679	3.75	AT-CC:1.0(	1/7
458	LOC_Os06gno_paralogOs06g0193	3.75	AT-CC:1.0(	1/7

Do not distribute

459	LOC_Os01gno_paralogOs01g0895	3.74	SC-CX:0.52	1/7
460	LOC_Os06gno_paralogOs06g0141	3.73	SC-CC:0.61	1/7
461	LOC_Os10gno_paralogOs10g0521	3.73	HS-HT:1.0(	1/7
462	LOC_Os01gLOC_Os05gOs01g0720	3.73	OS-GN:1.0	1/7
463	LOC_Os02gno_paralogOs02g0557	3.72	OS-CX:1.0(	1/7
464	LOC_Os09gno_paralogOs09g0268	3.72	SC-CC:0.61	1/7
465	LOC_Os05gno_paralogOs05g0497	3.72	AT-CC:1.0(	1/7
466	LOC_Os11gno_paralogOs11g0525	3.72	SC-LC:0.67	1/7
467	LOC_Os08gLOC_Os02gOs08g0452	3.71	HS-CX:1.0(	1/7
468	LOC_Os03gLOC_Os07gOs03g0333	3.71	HS-CX:1.0(	1/7
469	LOC_Os11gLOC_Os12gOs11g0125	3.71	SC-GT:1.0(	1/7
470	LOC_Os04gno_paralogOs04g0481	3.71	HS-HT:1.0(	1/7
471	LOC_Os03gno_paralogOs03g0669	3.69	OS-CX:1.0(	1/7
472	LOC_Os04gno_paralogOs04g0321	3.69	OS-CX:1.0(	1/7
473	LOC_Os03gLOC_Os07gOs03g0777	3.69	AT-CC:1.0(	1/7
474	LOC_Os12gLOC_Os03gOs12g0601	3.69	AT-CC:1.0(	1/7
475	LOC_Os03gLOC_Os12gOs03g0639	3.69	AT-CC:1.0(	1/7
476	LOC_Os01gno_paralogOs01g0707	3.69	SC-CC:1.0C	1/7
477	LOC_Os01gno_paralogOs01g0560	3.69	SC-CC:1.0C	1/7
478	LOC_Os07gno_paralogOs07g0131	3.69	SC-CC:0.61	1/7
479	LOC_Os07gno_paralogOs07g0206	3.67	SC-CC:0.61	1/7
480	LOC_Os03gno_paralogOs03g0711	3.67	OS-CX:1.0(	1/7
481	LOC_Os03gno_paralogOs03g0689	3.67	DM-CX:1.0	1/7
482	LOC_Os03gno_paralogOs03g0125	3.66	AT-CX:1.0(	1/7
483	LOC_Os03gno_paralogOs03g0243	3.66	HS-HT:1.0(	1/7
484	LOC_Os10gno_paralogOs10g0400	3.66	HS-LC:1.0C	1/7
485	LOC_Os04gno_paralogOs04g0454	3.66	SC-CC:0.61	1/7
486	LOC_Os03gno_paralogOs03g0278	3.64	SC-LC:0.69	1/7
487	LOC_Os12gno_paralogOs12g0601	3.64	AT-CC:1.0(	1/7
488	LOC_Os05gLOC_Os01gOs05g0455	3.63	AT-CC:1.0(	1/7
489	LOC_Os12gLOC_Os03gOs12g0583	3.63	AT-CC:1.0(	1/7
490	LOC_Os12gLOC_Os11gOs12g0100	3.63	SC-LC:0.69	1/7
491	LOC_Os06gno_paralogOs06g0331	3.63	OS-CX:1.0(	1/7
492	LOC_Os05gLOC_Os01gOs05g0574	3.63	SC-GT:1.0(	2/7
493	LOC_Os02gno_paralogOs02g0190	3.63	HS-LC:1.0C	1/7
494	LOC_Os07gno_paralogOs07g0297	3.62	HS-HT:1.0(	1/7
495	LOC_Os11gLOC_Os12gOs11g0100	3.62	SC-LC:0.69	1/7
496	LOC_Os03gLOC_Os10gOs03g0143	3.61	OS-CX:1.0(	1/7
497	LOC_Os03gno_paralogOs03g0427	3.61	OS-CX:0.5!	1/7
498	LOC_Os07gno_paralogOs07g0484	3.61	HS-HT:1.0(	1/7
499	LOC_Os02gLOC_Os04gOs02g0639	3.59	DM-CX:1.0	1/7
500	LOC_Os05gLOC_Os01gOs05g0533	3.59	OS-GN:1.0	1/7
501	LOC_Os03gno_paralogOs03g0820	3.58	HS-HT:1.0(	1/7
502	LOC_Os06gno_paralogOs06g0724	3.58	OS-CX:1.0(	1/7
503	LOC_Os01gLOC_Os05gOs01g0867	3.57	AT-CC:1.0(	1/7
504	LOC_Os04gno_paralogOs04g0557	3.57	AT-CX:1.0(	1/7

Do not distribute

505	LOC_Os02g	no_paralog	Os02g0525	3.56	OS-GN:1.0	1/7
506	LOC_Os01g	no_paralog	Os01g0659	3.56	SC-CC:0.66	1/7
507	LOC_Os03g	no_paralog	Os03g0219	3.55	SC-LC:0.69	1/7
508	LOC_Os08g	no_paralog	Os08g0427	3.54	SC-LC:0.70	1/7
509	LOC_Os02g	no_paralog	Os02g0139	3.54	DM-CX:1.0	1/7
510	LOC_Os09g	no_paralog	Os09g0521	3.53	SC-GT:1.00	1/7
511	LOC_Os01g	no_paralog	Os01g0675	3.52	HS-HT:1.00	1/7
512	LOC_Os02g	no_paralog	Os02g0602	3.51	SC-GT:1.00	1/7
513	LOC_Os06g	LOC_Os01g	Os06g0552	3.51	AT-CC:1.00	1/7
514	LOC_Os06g	no_paralog	Os06g0256	3.51	SC-LC:0.71	1/7
515	LOC_Os04g	LOC_Os02g	Os04g0552	3.5	AT-CC:1.00	1/7
516	LOC_Os10g	no_paralog	na	3.48	HS-LC:1.00	1/7
517	LOC_Os02g	LOC_Os06g	Os02g0137	3.47	AT-CX:1.00	1/7
518	LOC_Os09g	no_paralog	Os09g0306	3.46	DM-CX:1.0	1/7
519	LOC_Os05g	no_paralog	Os05g0125	3.45	OS-CX:1.00	1/7
520	LOC_Os02g	no_paralog	Os02g0480	3.45	DM-CX:1.0	1/7
521	LOC_Os06g	no_paralog	Os06g0111	3.44	OS-GN:0.5	1/7
522	LOC_Os11g	no_paralog	Os11g0484	3.44	OS-GN:0.5	1/7
523	LOC_Os03g	no_paralog	Os03g0152	3.44	CE-CX:1.00	1/7
524	LOC_Os01g	LOC_Os05g	Os01g0797	3.43	AT-CC:1.00	1/7
525	LOC_Os02g	no_paralog	Os02g0179	3.43	CE-CX:1.00	1/7
526	LOC_Os03g	no_paralog	Os03g0780	3.43	SC-GT:1.00	1/7
527	LOC_Os07g	no_paralog	Os07g0589	3.42	AT-CC:1.00	1/7
528	LOC_Os02g	LOC_Os06g	Os02g0110	3.42	DM-CX:1.0	1/7
529	LOC_Os02g	no_paralog	Os02g0829	3.41	HS-LC:1.00	1/7
530	LOC_Os02g	no_paralog	Os02g0709	3.4	SC-GT:1.00	1/7
531	LOC_Os06g	no_paralog	Os06g0103	3.4	CE-CX:0.55	1/7
532	LOC_Os03g	LOC_Os07g	Os03g0392	3.38	SC-GT:1.00	1/7
533	LOC_Os01g	LOC_Os05g	Os01g0848	3.38	AT-CC:1.00	1/7
534	LOC_Os03g	no_paralog	Os03g0246	3.38	SC-LC:1.00	1/7
535	LOC_Os04g	LOC_Os02g	Os04g0566	3.38	DM-CX:1.0	1/7
536	LOC_Os03g	no_paralog	Os03g0345	3.37	OS-CX:1.00	1/7
537	LOC_Os05g	no_paralog	Os05g0595	3.37	DR-CX:1.00	1/7
538	LOC_Os11g	no_paralog	Os11g0191	3.36	HS-LC:1.00	1/7
539	LOC_Os07g	no_paralog	Os07g0472	3.36	OS-CX:1.00	1/7
540	LOC_Os02g	LOC_Os03g	na	3.33	AT-CC:1.00	1/7
541	LOC_Os02g	no_paralog	Os02g0776	3.33	HS-LC:1.00	1/7
542	LOC_Os02g	no_paralog	Os02g0120	3.33	HS-CX:1.00	1/7
543	LOC_Os09g	no_paralog	Os09g0338	3.31	OS-GN:1.0	1/7
544	LOC_Os02g	no_paralog	Os02g0746	3.3	HS-LC:1.00	1/7
545	LOC_Os01g	no_paralog	Os01g0678	3.3	DM-CX:1.0	1/7
546	LOC_Os01g	no_paralog	Os01g0271	3.3	OS-GN:0.5	1/7
547	LOC_Os05g	no_paralog	Os05g0387	3.3	AT-HT:1.00	1/7
548	LOC_Os03g	no_paralog	Os03g0425	3.3	AT-CC:1.00	1/7
549	LOC_Os07g	no_paralog	Os07g0105	3.28	AT-HT:1.00	1/7
550	LOC_Os07g	no_paralog	Os07g0495	3.27	OS-CX:1.00	1/7

Do not distribute

551	LOC_Os11gLOC_Os12gOs11g0110	3.27	AT-HT:1.0(	1/7
552	LOC_Os12gLOC_Os11gOs12g0110	3.27	AT-HT:1.0(	1/7
553	LOC_Os01gLOC_Os05gOs01g0873	3.25	CE-CX:1.0(	1/7
554	LOC_Os12gno_paralogOs12g0597	3.24	AT-HT:1.0(	1/7
555	LOC_Os06gno_paralogOs06g0127	3.23	AT-CC:1.0(	1/7
556	LOC_Os08gLOC_Os02gOs08g0433	3.23	AT-CC:1.0(	1/7
557	LOC_Os02gLOC_Os04gOs02g0641	3.23	AT-CC:1.0(	1/7
558	LOC_Os03gno_paralogOs03g0266	3.23	AT-CC:1.0(	1/7
559	LOC_Os08gno_paralogOs08g0512	3.22	SC-GT:1.0(	1/7
560	LOC_Os02gLOC_Os01gOs02g0232	3.22	OS-CX:1.0(	1/7
561	LOC_Os08gno_paralogOs08g0123	3.22	OS-CX:1.0(	1/7
562	LOC_Os03gLOC_Os07gOs03g0758	3.21	AT-CX:1.0(	1/7
563	LOC_Os05gno_paralogOs05g0168	3.21	AT-HT:1.0(	1/7
564	LOC_Os10gno_paralogOs10g0369	3.2	OS-GN:0.5	1/7
565	LOC_Os06gno_paralogOs06g0136	3.2	SC-GT:1.0(	1/7
566	LOC_Os04gLOC_Os02gOs04g0530	3.19	DM-CX:1.0	1/7
567	LOC_Os02gno_paralogOs02g0723	3.19	AT-CC:1.0(	1/7
568	LOC_Os11gLOC_Os12gOs11g0186	3.19	SC-LC:1.00	1/7
569	LOC_Os03gno_paralogOs03g0397	3.19	OS-CX:1.0(	1/7
570	LOC_Os12gno_paralogOs12g0507	3.18	AT-CC:1.0(	1/7
571	LOC_Os03gLOC_Os07gOs03g0366	3.16	AT-CC:1.0(	1/7
572	LOC_Os09gno_paralogOs09g0106	3.15	AT-CC:1.0(	1/7
573	LOC_Os02gno_paralogOs02g0120	3.15	OS-CX:1.0(	1/7
574	LOC_Os01gno_paralogOs01g0918	3.15	HS-LC:1.0(	1/7
575	LOC_Os04gLOC_Os02gOs04g0614	3.14	AT-CC:1.0(	1/7
576	LOC_Os02gno_paralogOs02g0315	3.14	HS-CX:1.0(	1/7
577	LOC_Os08gLOC_Os09gOs08g0555	3.14	AT-CC:1.0(	1/7
578	LOC_Os12gno_paralogOs12g0209	3.14	HS-CX:1.0(	1/7
579	LOC_Os08gno_paralogOs08g0560	3.13	AT-HT:1.0(	1/7
580	LOC_Os10gno_paralogOs10g0560	3.12	HS-LC:1.0(	1/7
581	LOC_Os02gno_paralogOs02g0169	3.12	OS-CX:1.0(	1/7
582	LOC_Os03gno_paralogOs03g0213	3.12	SC-CC:1.0(	1/7
583	LOC_Os07gno_paralogOs07g0222	3.11	OS-CX:1.0(	1/7
584	LOC_Os03gno_paralogna	3.1	AT-CC:1.0(	1/7
585	LOC_Os01gno_paralogOs01g0558	3.1	OS-CX:1.0(	1/7
586	LOC_Os09gno_paralogOs09g0411	3.1	AT-CX:1.0(	1/7
587	LOC_Os03gno_paralogOs03g0851	3.1	SC-GT:1.0(	1/7
588	LOC_Os05gLOC_Os01gOs05g0437	3.1	AT-CC:1.0(	1/7
589	LOC_Os09gno_paralogOs09g0347	3.1	SC-CC:1.0(	1/7
590	LOC_Os09gno_paralogOs09g0347	3.1	SC-CC:1.0(	1/7
591	LOC_Os08gLOC_Os09gOs08g0467	3.09	DM-CX:1.0	1/7
592	LOC_Os02gLOC_Os06gOs02g0180	3.09	DM-CX:1.0	1/7
593	LOC_Os08gLOC_Os09gOs08g0532	3.09	HS-LC:1.0(	1/7
594	LOC_Os02gno_paralogOs02g0682	3.09	OS-CX:1.0(	1/7
595	LOC_Os03gno_paralogOs03g0108	3.09	SC-HT:1.0(	1/7
596	LOC_Os03gno_paralogOs03g0633	3.08	AT-CC:1.0(	1/7

Do not distribute

597	LOC_Os12gLOC_Os03gOs12g0610	3.08	AT-CC:1.0(	1/7
598	LOC_Os02gLOC_Os04gOs02g0693	3.07	DM-HT:1.(	1/7
599	LOC_Os04gLOC_Os02gOs04g0592	3.07	DM-HT:1.(	1/7
600	LOC_Os11gno_paralogOs11g0432	3.07	OS-CX:1.0(	1/7
601	LOC_Os03gno_paralogOs03g0165	3.06	AT-CC:1.0(	1/7
602	LOC_Os07gno_paralogOs07g0685	3.06	AT-CC:1.0(	1/7
603	LOC_Os06gno_paralogOs06g0154	3.06	OS-CX:1.0(	1/7
604	LOC_Os07gLOC_Os03gOs07g0138	3.05	AT-CC:1.0(	1/7
605	LOC_Os02gno_paralogOs02g0732	3.05	OS-CX:1.0(	1/7
606	LOC_Os01gno_paralogOs01g0918	3.05	HS-LC:1.0C	1/7
607	LOC_Os02gno_paralogOs02g0564	3.05	AT-CC:1.0(	1/7
608	LOC_Os04gno_paralogOs04g0643	3.04	HS-LC:1.0C	1/7
609	LOC_Os06gLOC_Os02gOs06g0667	3.03	AT-CC:1.0(	1/7
610	LOC_Os05gLOC_Os01gOs05g0230	3.03	AT-CC:1.0(	1/7
611	LOC_Os02gLOC_Os10gOs02g0782	3.03	AT-CC:1.0(	1/7
612	LOC_Os05gno_paralogOs05g0144	3.03	SC-GT:1.0(	1/7
613	LOC_Os02gLOC_Os04gOs02g0673	3.03	SC-GT:1.0(	1/7
614	LOC_Os09gno_paralogOs09g0509	3.03	OS-CX:1.0(	1/7
615	LOC_Os03gLOC_Os12gOs03g0610	3.02	AT-CC:1.0(	1/7
616	LOC_Os09gLOC_Os02gOs09g0445	3.02	AT-CC:1.0(	1/7
617	LOC_Os07gLOC_Os03gOs07g0620	3.02	SC-GT:1.0(	1/7
618	LOC_Os02gno_paralogOs02g0833	3.01	HS-LC:1.0C	1/7
619	LOC_Os06gno_paralogOs06g0151	3	OS-GN:1.0	1/7
620	LOC_Os12gno_paralogOs12g0628	3	SC-GT:1.0(	1/7
621	LOC_Os05gno_paralogOs05g0461	3	AT-CC:1.0(	1/7
622	LOC_Os10gno_paralogOs10g0478	2.99	OS-CX:1.0(	1/7
623	LOC_Os07gLOC_Os03gOs07g0680	2.98	HS-CX:1.0(	1/7
624	LOC_Os02gno_paralogOs02g0293	2.98	AT-CC:1.0(	1/7
625	LOC_Os03gLOC_Os03gOs03g0710	2.97	AT-CC:1.0(	1/7
626	LOC_Os06gLOC_Os02gOs06g0653	2.97	CE-CC:1.0(	1/7
627	LOC_Os01gno_paralogna	2.97	AT-CC:1.0(	1/7
628	LOC_Os08gLOC_Os02gOs08g0463	2.96	AT-CC:1.0(	1/7
629	LOC_Os06gno_paralogOs06g0111	2.96	DM-CX:1.0	1/7
630	LOC_Os09gLOC_Os08gOs09g0507	2.95	HS-LC:1.0C	1/7
631	LOC_Os02gLOC_Os04gOs02g0650	2.94	OS-GN:1.0	1/7
632	LOC_Os03gno_paralogOs03g0794	2.94	OS-GN:1.0	1/7
633	LOC_Os03gno_paralogOs03g0196	2.94	OS-GN:1.0	1/7
634	LOC_Os03gno_paralogOs03g0829	2.93	DM-HT:1.(	1/7
635	LOC_Os03gLOC_Os10gOs03g0252	2.92	OS-CX:1.0(	1/7
636	LOC_Os02gLOC_Os06gOs02g0148	2.92	DM-CX:1.0	1/7
637	LOC_Os04gLOC_Os02gOs04g0533	2.92	AT-CC:1.0(	1/7
638	LOC_Os03gLOC_Os07gOs03g0764	2.92	AT-CC:1.0(	1/7
639	LOC_Os02gno_paralogOs02g0179	2.91	HS-CX:1.0(	1/7
640	LOC_Os05gno_paralogOs05g0289	2.91	OS-CX:1.0(	1/7
641	LOC_Os12gLOC_Os11gOs12g0177	2.91	SC-LC:1.00	1/7
642	LOC_Os04gLOC_Os02gOs04g0567	2.9	SC-GT:1.0(	1/7

Do not distribute

643	LOC_Os02gLOC_Os06gOs02g0178	2.9	CE-CC:1.00	1/7
644	LOC_Os02gLOC_Os04gOs02g0608	2.89	DM-CX:1.0	1/7
645	LOC_Os04gno_paralogOs04g0690	2.89	OS-CX:1.00	1/7
646	LOC_Os03gno_paralogOs03g0216	2.89	AT-CC:1.00	1/7
647	LOC_Os01gno_paralogOs01g0172	2.87	SC-CX:1.00	1/7
648	LOC_Os02gno_paralog na	2.87	OS-GN:1.0	1/7
649	LOC_Os05gLOC_Os01gOs05g0430	2.87	CE-CX:1.00	1/7
650	LOC_Os05gno_paralogOs05g0593	2.86	SC-LC:1.00	1/7
651	LOC_Os01gno_paralogOs01g0570	2.86	SC-LC:1.00	1/7
652	LOC_Os01gno_paralogOs01g0147	2.86	OS-CX:1.00	1/7
653	LOC_Os10gno_paralogOs10g0415	2.86	SC-LC:1.00	1/7
654	LOC_Os07gno_paralogOs07g0214	2.86	OS-CX:1.00	1/7
655	LOC_Os04gLOC_Os02gOs04g0555	2.85	AT-CC:1.00	1/7
656	LOC_Os01gLOC_Os01gOs01g0626	2.85	AT-CC:1.00	1/7
657	LOC_Os09gno_paralogOs09g0372	2.84	DM-CX:1.0	1/7
658	LOC_Os03gLOC_Os12gOs03g0633	2.83	AT-CC:1.00	1/7
659	LOC_Os01gno_paralogOs01g0657	2.82	OS-CX:1.00	1/7
660	LOC_Os01gno_paralogOs01g0549	2.82	OS-CX:1.00	1/7
661	LOC_Os08gno_paralogOs08g0250	2.82	OS-CX:1.00	1/7
662	LOC_Os02gLOC_Os04gOs02g0672	2.81	DM-CX:1.0	1/7
663	LOC_Os04gLOC_Os02gOs04g0543	2.81	OS-GN:1.0	1/7
664	LOC_Os01gLOC_Os02gOs01g0229	2.81	HS-LC:1.00	1/7
665	LOC_Os09gLOC_Os08gOs09g0439	2.81	OS-CX:1.00	1/7
666	LOC_Os01gLOC_Os05gOs01g0867	2.8	HS-LC:1.00	1/7
667	LOC_Os06gLOC_Os02gOs06g0194	2.8	AT-CC:1.00	1/7
668	LOC_Os02gLOC_Os11gOs02g0533	2.79	HS-LC:1.00	1/7
669	LOC_Os08gno_paralogOs08g0424	2.79	HS-LC:1.00	1/7
670	LOC_Os08gno_paralogOs08g0423	2.79	HS-LC:1.00	1/7
671	LOC_Os03gno_paralogOs03g0859	2.79	OS-CX:1.00	1/7
672	LOC_Os02gno_paralogOs02g0768	2.79	SC-CC:1.00	1/7
673	LOC_Os05gno_paralogOs05g0115	2.78	AT-CX:1.00	1/7
674	LOC_Os02gLOC_Os06gOs02g0139	2.78	AT-CC:1.00	1/7
675	LOC_Os03gno_paralogOs03g0176	2.78	OS-CX:1.00	1/7
676	LOC_Os12gno_paralogOs12g0564	2.77	AT-CX:1.00	1/7
677	LOC_Os05gLOC_Os01gOs05g0100	2.77	DM-CX:1.0	1/7
678	LOC_Os07gno_paralogOs07g0496	2.77	SC-GT:1.00	1/7
679	LOC_Os03gno_paralogOs03g0191	2.77	SC-GT:1.00	1/7
680	LOC_Os06gno_paralogOs06g0693	2.77	HS-LC:1.00	1/7
681	LOC_Os08gno_paralogOs08g0423	2.77	HS-LC:1.00	1/7
682	LOC_Os07gLOC_Os03gOs07g0566	2.77	AT-CC:1.00	1/7
683	LOC_Os12gLOC_Os03gOs12g0614	2.76	DM-CX:1.0	1/7
684	LOC_Os01gno_paralog na	2.76	AT-CC:1.00	1/7
685	LOC_Os12gno_paralogOs12g0528	2.75	HS-CX:1.00	1/7
686	LOC_Os02gno_paralogOs02g0663	2.75	AT-CC:1.00	1/7
687	LOC_Os02gno_paralogOs02g0308	2.75	OS-CX:1.00	1/7
688	LOC_Os02gno_paralogOs02g0552	2.75	HS-CX:1.00	1/7

Do not distribute

689	LOC_Os06gLOC_Os02gOs06g0699	2.74	DM-CX:1.0	1/7
690	LOC_Os05gno_paralogOs05g0111	2.74	HS-LC:1.0(	1/7
691	LOC_Os01gno_paralogOs01g0587	2.74	CE-CC:1.0(	1/7
692	LOC_Os03gno_paralogOs03g0179	2.74	CE-CC:1.0(	1/7
693	LOC_Os02gLOC_Os10gOs02g0662	2.74	AT-CC:1.0(	1/7
694	LOC_Os12gno_paralogOs12g0609	2.73	CE-CC:1.0(	1/7
695	LOC_Os03gno_paralogOs03g0586	2.73	AT-CX:1.0(	1/7
696	LOC_Os07gno_paralogOs07g0218	2.73	CE-CC:1.0(	1/7
697	LOC_Os07gno_paralogOs07g0635	2.73	CE-CC:1.0(	1/7
698	LOC_Os06gno_paralogOs06g0191	2.73	CE-CC:1.0(	1/7
699	LOC_Os01gno_paralogOs01g0628	2.73	CE-CC:1.0(	1/7
700	LOC_Os07gLOC_Os03gOs07g0635	2.73	CE-CC:1.0(	1/7
701	LOC_Os03gno_paralogOs03g0711	2.72	CE-CX:1.0(	1/7
702	LOC_Os03gno_paralogOs03g0582	2.72	OS-CX:1.0(	1/7
703	LOC_Os11gLOC_Os12gOs11g0195	2.72	OS-CX:1.0(	1/7
704	LOC_Os06gno_paralogOs06g0670	2.72	OS-CX:1.0(	1/7
705	LOC_Os07gno_paralogOs07g0452	2.72	SC-GT:1.0(	1/7
706	LOC_Os06gLOC_Os02gOs06g0698	2.72	SC-GT:1.0(	1/7
707	LOC_Os10gno_paralogOs10g0483	2.71	SC-GT:1.0(	1/7
708	LOC_Os06gno_paralogOs06g0705	2.71	DM-CX:1.0	1/7
709	LOC_Os04gno_paralogOs04g0609	2.71	DM-CX:1.0	1/7
710	LOC_Os12gno_paralogOs12g0562	2.71	HS-LC:1.0(	1/7
711	LOC_Os07gLOC_Os03gOs07g0694	2.71	HS-LC:1.0(	1/7
712	LOC_Os01gno_paralogOs01g0174	2.7	OS-CX:1.0(	1/7
713	LOC_Os10gno_paralogOs10g0497	2.7	OS-CX:1.0(	1/7
714	LOC_Os04gLOC_Os11gOs04g0688	2.7	OS-CX:1.0(	1/7
715	LOC_Os01gno_paralogOs01g0264	2.69	AT-CC:1.0(	1/7
716	LOC_Os07gno_paralogOs07g0618	2.69	OS-GN:1.0	1/7
717	LOC_Os01gno_paralogOs01g0708	2.69	SC-GT:1.0(	1/7
718	LOC_Os02gno_paralogOs02g0655	2.69	SC-LC:1.00	1/7
719	LOC_Os02gLOC_Os06gOs02g0187	2.69	OS-CX:1.0(	1/7
720	LOC_Os12gLOC_Os11gOs12g0136	2.68	DM-HT:1.0	1/7
721	LOC_Os08gno_paralogOs08g0396	2.68	OS-CX:1.0(	1/7
722	LOC_Os05gLOC_Os01gOs05g0169	2.67	OS-CX:1.0(	1/7
723	LOC_Os08gLOC_Os09gOs08g0531	2.67	OS-CX:1.0(	1/7
724	LOC_Os02gno_paralogOs02g0123	2.67	SC-HT:1.0(	1/7
725	LOC_Os02gLOC_Os10gOs02g0810	2.67	SC-LC:1.00	1/7
726	LOC_Os11gLOC_Os12gOs11g0142	2.66	OS-CX:1.0(	1/7
727	LOC_Os05gno_paralogOs05g0540	2.66	OS-CX:1.0(	1/7
728	LOC_Os08gno_paralogOs08g0309	2.66	SC-GT:1.0(	1/7
729	LOC_Os02gLOC_Os06gOs02g0217	2.66	OS-CX:1.0(	1/7
730	LOC_Os03gno_paralogOs03g0109	2.65	SC-GT:1.0(	1/7
731	LOC_Os05gLOC_Os01gOs05g0176	2.65	OS-CX:1.0(	1/7
732	LOC_Os04gLOC_Os02gOs04g0540	2.65	SC-LC:1.00	1/7
733	LOC_Os06gno_paralogOs06g0708	2.65	OS-CX:1.0(	1/7
734	LOC_Os07gno_paralogOs07g0179	2.65	HS-CX:1.0(	1/7

Do not distribute

735	LOC_Os08gLOC_Os04gOs08g0192	2.64	AT-CX:1.0(	1/7
736	LOC_Os11gLOC_Os12gOs11g0140	2.64	DM-HT:1.(	1/7
737	LOC_Os06gLOC_Os02gOs06g0703	2.64	AT-CC:1.0(	1/7
738	LOC_Os05gLOC_Os01gOs05g0475	2.64	OS-CX:1.0(	1/7
739	LOC_Os04gno_paralogOs04g0268	2.63	OS-CX:1.0(	1/7
740	LOC_Os03gno_paralogOs03g0133	2.63	OS-GN:1.0	1/7
741	LOC_Os03gno_paralogOs03g0801	2.63	SC-HT:1.0(	1/7
742	LOC_Os06gLOC_Os02gOs06g0651	2.63	DM-CX:1.0	1/7
743	LOC_Os11gno_paralogOs11g0414	2.63	HS-CX:1.0(	1/7
744	LOC_Os02gLOC_Os10gOs02g0622	2.63	SC-LC:1.00	1/7
745	LOC_Os05gno_paralogOs05g0276	2.63	SC-GT:1.0(	1/7
746	LOC_Os10gno_paralogOs10g0412	2.63	OS-CX:1.0(	1/7
747	LOC_Os02gno_paralogOs02g0598	2.63	OS-CX:1.0(	1/7
748	LOC_Os04gLOC_Os02gOs04g0514	2.63	SC-LC:1.00	1/7
749	LOC_Os04gLOC_Os02gOs04g0412	2.62	HS-LC:1.0(	1/7
750	LOC_Os02gno_paralogOs02g0647	2.62	SC-LC:1.00	1/7
751	LOC_Os03gno_paralogOs03g0152	2.62	OS-CX:1.0(	1/7
752	LOC_Os09gno_paralogOs09g0249	2.62	OS-CX:1.0(	1/7
753	LOC_Os09gLOC_Os02gOs09g0434	2.62	AT-CC:1.0(	1/7
754	LOC_Os03gno_paralogOs03g0738	2.62	OS-CX:1.0(	1/7
755	LOC_Os01gno_paralogOs01g0939	2.62	DM-HT:1.(	1/7
756	LOC_Os11gno_paralogOs11g0661	2.62	HS-LC:1.0(	1/7
757	LOC_Os11gno_paralogOs11g0648	2.61	OS-CX:1.0(	1/7
758	LOC_Os04gLOC_Os06gOs04g0659	2.61	SC-GT:1.0(	1/7
759	LOC_Os07gno_paralogOs07g0421	2.61	OS-PG:1.0(	1/7
760	LOC_Os04gno_paralogOs04g0560	2.6	DM-CX:1.0	1/7
761	LOC_Os12gno_paralogOs12g0635	2.6	OS-CX:1.0(	1/7
762	LOC_Os03gLOC_Os07gOs03g0422	2.6	OS-CX:1.0(	1/7
763	LOC_Os03gno_paralogOs03g0225	2.59	OS-CX:1.0(	1/7
764	LOC_Os02gLOC_Os06gOs02g0161	2.59	SC-LC:1.00	1/7
765	LOC_Os06gLOC_Os02gOs06g0681	2.59	SC-LC:1.00	1/7
766	LOC_Os05gno_paralogna	2.59	SC-LC:1.00	1/7
767	LOC_Os07gno_paralogOs07g0245	2.59	CE-CX:1.0(	1/7
768	LOC_Os05gno_paralogOs05g0558	2.59	CE-CX:1.0(	1/7
769	LOC_Os01gno_paralogOs01g0619	2.58	AT-CC:1.0(	1/7
770	LOC_Os01gno_paralogOs01g0277	2.58	AT-CC:1.0(	1/7
771	LOC_Os06gno_paralogOs06g0699	2.58	SC-GT:1.0(	1/7
772	LOC_Os04gno_paralogOs04g0175	2.57	OS-CX:1.0(	1/7
773	LOC_Os03gLOC_Os05gna	2.57	HS-LC:1.0(	1/7
774	LOC_Os11gno_paralogOs11g0181	2.57	SC-GT:1.0(	1/7
775	LOC_Os11gno_paralogOs11g0181	2.57	SC-GT:1.0(	1/7
776	LOC_Os03gno_paralogOs03g0773	2.57	SC-GT:1.0(	1/7
777	LOC_Os10gno_paralogOs10g0517	2.57	OS-GN:1.0	1/7
778	LOC_Os05gLOC_Os01gOs05g0583	2.57	AT-CC:1.0(	1/7
779	LOC_Os04gno_paralogOs04g0407	2.56	OS-CX:1.0(	1/7
780	LOC_Os09gno_paralogOs09g0281	2.56	DM-CX:1.0	1/7

Do not distribute

781	LOC_Os10gLOC_Os02gOs10g0476	2.56	SC-LC:1.00	1/7
782	LOC_Os01gLOC_Os05gOs01g0812	2.55	AT-CX:1.0(	1/7
783	LOC_Os01gno_paralogOs01g0104	2.55	OS-CX:1.0(	1/7
784	LOC_Os12gno_paralogOs12g0538	2.55	HS-CX:1.0(	1/7
785	LOC_Os02gno_paralogOs02g0686	2.55	SC-CC:1.0(	1/7
786	LOC_Os10gLOC_Os03gOs10g0556	2.55	DM-CX:1.0	1/7
787	LOC_Os06gno_paralogOs06g0473	2.54	OS-CX:1.0(	1/7
788	LOC_Os01gLOC_Os05gOs01g0958	2.54	SC-GT:1.0(	1/7
789	LOC_Os02gLOC_Os01gOs02g0280	2.54	SC-LC:1.00	1/7
790	LOC_Os08gLOC_Os02gOs08g0473	2.54	OS-CX:1.0(	1/7
791	LOC_Os02gno_paralogOs02g0208	2.54	OS-CX:1.0(	1/7
792	LOC_Os08gno_paralogOs08g0484	2.54	SC-GT:1.0(	1/7
793	LOC_Os09gno_paralogOs09g0553	2.54	SC-HT:1.0(	1/7
794	LOC_Os01gLOC_Os05gOs01g0835	2.54	SC-HT:1.0(	1/7
795	LOC_Os03gLOC_Os02gOs03g0119	2.54	SC-HT:1.0(	1/7
796	LOC_Os09gno_paralogOs09g0433	2.54	SC-HT:1.0(	1/7
797	LOC_Os04gLOC_Os02gOs04g0583	2.54	SC-HT:1.0(	1/7
798	LOC_Os10gLOC_Os02gOs10g0539	2.54	SC-HT:1.0(	1/7
799	LOC_Os02gLOC_Os10gOs02g0684	2.54	SC-HT:1.0(	1/7
800	LOC_Os07gno_paralogOs07g0549	2.54	SC-HT:1.0(	1/7
801	LOC_Os05gLOC_Os01gOs05g0466	2.54	SC-HT:1.0(	1/7
802	LOC_Os05gLOC_Os01gOs05g0462	2.54	SC-HT:1.0(	1/7
803	LOC_Os04gno_paralogOs04g0628	2.53	SC-LC:1.00	1/7
804	LOC_Os06gno_paralogOs06g0521	2.53	OS-CX:1.0(	1/7
805	LOC_Os02gno_paralogOs02g0106	2.53	AT-CX:1.0(	1/7
806	LOC_Os05gLOC_Os03gOs05g0127	2.53	HS-LC:1.0(	1/7
807	LOC_Os01gLOC_Os02gOs01g0231	2.53	SC-LC:1.00	1/7
808	LOC_Os05gno_paralogOs05g0507	2.53	AT-CX:1.0(	1/7
809	LOC_Os12gno_paralogOs12g0443	2.52	AT-CX:1.0(	1/7
810	LOC_Os05gno_paralogOs05g0540	2.52	OS-CX:1.0(	1/7
811	LOC_Os08gLOC_Os09gOs08g0374	2.52	DR-CX:1.0(	1/7
812	LOC_Os09gno_paralogOs09g0286	2.51	SC-GT:1.0(	1/7
813	LOC_Os02gno_paralogOs02g0506	2.51	CE-CX:1.0(	1/7
814	LOC_Os06gno_paralogOs06g0714	2.51	SC-CX:1.0(	1/7
815	LOC_Os08gno_paralogOs08g0538	2.51	CE-CX:1.0(	1/7
816	LOC_Os03gno_paralogOs03g0405	2.5	OS-CX:1.0(	1/7
817	LOC_Os02gno_paralogOs02g0575	2.5	DM-CX:1.0	1/7
818	LOC_Os09gno_paralogOs09g0528	2.5	AT-CX:1.0(	1/7
819	LOC_Os03gno_paralogOs03g0265	2.5	OS-CX:1.0(	1/7
820	LOC_Os07gno_paralogOs07g0499	2.5	HS-CX:1.0(	1/7
821	LOC_Os04gno_paralogOs04g0252	2.5	OS-CX:1.0(	1/7
822	LOC_Os09gLOC_Os08gOs09g0538	2.5	CE-CX:1.0(	1/7
823	LOC_Os05gno_paralogOs05g0132	2.49	OS-CX:1.0(	1/7
824	LOC_Os01gLOC_Os01gOs01g0629	2.49	DM-CX:1.0	1/7
825	LOC_Os03gno_paralogOs03g0259	2.49	SC-CX:1.0(	1/7
826	LOC_Os08gno_paralogOs08g0155	2.49	SC-GT:1.0(	1/7

Do not distribute

827	LOC_Os10gno_paralogOs10g0549	2.49	SC-GT:1.0(	1/7
828	LOC_Os04gno_paralogOs04g0557	2.49	AT-CX:1.0(	1/7
829	LOC_Os03gno_paralogOs03g0620	2.49	OS-CX:1.0(	1/7
830	LOC_Os05gno_paralogOs05g0402	2.49	SC-GT:1.0(	1/7
831	LOC_Os09gno_paralog na	2.49	SC-CX:1.0(	1/7
832	LOC_Os05gno_paralogOs05g0495	2.49	DM-HT:1.(	1/7
833	LOC_Os12gno_paralogOs12g0613	2.49	SC-LC:1.00	1/7
834	LOC_Os03gno_paralogOs03g0289	2.49	SC-GT:1.0(	1/7
835	LOC_Os02gno_paralogOs02g0805	2.49	SC-LC:1.00	1/7
836	LOC_Os08gno_paralogOs08g0406	2.48	OS-CX:1.0(	1/7
837	LOC_Os10gno_paralogOs10g0573	2.48	SC-LC:1.00	1/7
838	LOC_Os05gLOC_Os01gOs05g0383	2.48	SC-GT:1.0(	1/7
839	LOC_Os02gno_paralogOs02g0121	2.48	OS-GN:1.0	1/7
840	LOC_Os07gno_paralogOs07g0522	2.48	SC-LC:1.00	1/7
841	LOC_Os01gLOC_Os05gOs01g0962	2.47	SC-GT:1.0(	1/7
842	LOC_Os09gLOC_Os08gOs09g0323	2.47	DR-CX:1.0(	1/7
843	LOC_Os01gLOC_Os05gOs01g0180	2.47	SC-LC:1.00	1/7
844	LOC_Os11gno_paralogOs11g0456	2.47	SC-LC:1.00	1/7
845	LOC_Os06gLOC_Os02gOs06g0168	2.47	SC-LC:1.00	1/7
846	LOC_Os08gno_paralogOs08g0547	2.47	SC-GT:1.0(	1/7
847	LOC_Os04gno_paralogOs04g0636	2.47	OS-CX:1.0(	1/7
848	LOC_Os03gno_paralogOs03g0833	2.46	CE-CX:1.0(	1/7
849	LOC_Os02gLOC_Os06gOs02g0804	2.46	SC-LC:1.00	1/7
850	LOC_Os05gLOC_Os01gOs05g0181	2.46	SC-LC:1.00	1/7
851	LOC_Os07gno_paralogOs07g0571	2.46	OS-CX:1.0(	1/7
852	LOC_Os07gno_paralogOs07g0693	2.46	DM-CX:1.0	1/7
853	LOC_Os09gno_paralogOs09g0375	2.46	SC-LC:1.00	1/7
854	LOC_Os05gno_paralogOs05g0117	2.46	HS-CX:1.0(	1/7
855	LOC_Os04gno_paralogOs04g0334	2.46	OS-CX:1.0(	1/7
856	LOC_Os02gno_paralogOs02g0219	2.45	SC-GT:1.0(	1/7
857	LOC_Os09gno_paralogOs09g0503	2.45	OS-CX:1.0(	1/7
858	LOC_Os10gno_paralogOs10g0554	2.45	SC-GT:1.0(	1/7
859	LOC_Os03gno_paralogOs03g0704	2.45	SC-GT:1.0(	1/7
860	LOC_Os08gLOC_Os09gOs08g0548	2.45	CE-CX:1.0(	1/7
861	LOC_Os01gno_paralogOs01g0232	2.44	OS-CX:1.0(	1/7
862	LOC_Os04gLOC_Os02gOs04g0496	2.44	DM-CX:1.0	1/7
863	LOC_Os02gLOC_Os06gOs02g0105	2.44	OS-CX:1.0(	1/7
864	LOC_Os11gno_paralogOs11g0212	2.43	DM-CX:1.0	1/7
865	LOC_Os01gno_paralogOs01g0165	2.43	OS-CX:1.0(	1/7
866	LOC_Os03gno_paralogOs03g0703	2.43	DM-HT:1.(	1/7
867	LOC_Os05gno_paralogOs05g0387	2.43	DM-CX:1.0	1/7
868	LOC_Os04gno_paralogOs04g0550	2.42	SC-GT:1.0(	1/7
869	LOC_Os08gno_paralogOs08g0232	2.42	OS-CX:1.0(	1/7
870	LOC_Os02gno_paralogOs02g0157	2.42	OS-CX:1.0(	1/7
871	LOC_Os02gno_paralogOs02g0218	2.42	DR-CX:1.0(	1/7
872	LOC_Os09gno_paralogOs09g0362	2.42	DR-CX:1.0(	1/7

Do not distribute

873 LOC_Os05g LOC_Os01g Os05g0485	2.42	OS-CX:1.0(	1/7
874 LOC_Os02g no_paralog Os02g0288	2.41	OS-CX:1.0(	1/7
875 LOC_Os12g no_paralog Os12g0188	2.41	DM-CX:1.0	1/7
876 LOC_Os06g no_paralog Os06g0489	2.41	OS-CX:1.0(	1/7
877 LOC_Os12g no_paralog Os12g0233	2.41	AT-CX:1.0(	1/7
878 LOC_Os02g no_paralog Os02g0119	2.4	CE-CX:1.0(	1/7
879 LOC_Os09g no_paralog Os09g0514	2.4	DR-CX:1.0(	1/7
880 LOC_Os01g no_paralog Os01g0911	2.4	OS-CX:1.0(	1/7
881 LOC_Os03g LOC_Os05g Os03g0286	2.4	SC-HT:1.0(	1/7
882 LOC_Os11g LOC_Os12g Os11g0634	2.39	DM-HT:1.0	1/7
883 LOC_Os12g LOC_Os11g Os12g0508	2.39	DM-HT:1.0	1/7
884 LOC_Os03g no_paralog Os03g0773	2.39	DM-HT:1.0	1/7
885 LOC_Os05g no_paralog Os05g0477	2.39	OS-CX:1.0(	1/7
886 LOC_Os06g no_paralog Os06g0729	2.38	DM-CX:1.0	1/7
887 LOC_Os01g LOC_Os03g Os01g0160	2.38	SC-HT:1.0(	1/7
888 LOC_Os11g LOC_Os12g Os11g0135	2.38	SC-HT:1.0(	1/7
889 LOC_Os01g no_paralog Os01g0110	2.37	AT-CX:1.0(	1/7
890 LOC_Os10g no_paralog Os10g0545	2.37	OS-CX:1.0(	1/7
891 LOC_Os05g LOC_Os01g Os05g0389	2.36	SC-GT:1.0(	1/7
892 LOC_Os04g no_paralog Os04g0561	2.36	OS-GN:1.0	1/7
893 LOC_Os02g no_paralog Os02g0831	2.36	DM-CX:1.0	1/7
894 LOC_Os12g LOC_Os11g Os12g0133	2.36	SC-HT:1.0(	1/7
895 LOC_Os03g no_paralog Os03g0237	2.36	DM-CX:1.0	1/7
896 LOC_Os06g no_paralog Os06g0699	2.36	OS-GN:1.0	1/7
897 LOC_Os01g no_paralog Os01g0108	2.36	OS-GN:1.0	1/7
898 LOC_Os02g no_paralog Os02g0157	2.36	OS-CX:1.0(	1/7
899 LOC_Os08g no_paralog Os08g0559	2.36	AT-CX:1.0(	1/7
900 LOC_Os03g LOC_Os07g Os03g0383	2.36	DM-CX:1.0	1/7
901 LOC_Os08g no_paralog Os08g0130	2.35	SC-HT:1.0(	1/7
902 LOC_Os08g LOC_Os09g Os08g0474	2.34	OS-CX:1.0(	1/7
903 LOC_Os01g LOC_Os01g Os01g0711	2.34	SC-CC:1.0(	1/7
904 LOC_Os03g no_paralog Os03g0269	2.34	OS-CX:1.0(	1/7
905 LOC_Os09g LOC_Os08g Os09g0338	2.34	AT-CX:1.0(	1/7
906 LOC_Os10g no_paralog Os10g0456	2.34	OS-CX:1.0(	1/7
907 LOC_Os01g LOC_Os05g Os01g0772	2.34	SC-GT:1.0(	1/7
908 LOC_Os07g no_paralog Os07g0544	2.33	DR-CX:1.0(	1/7
909 LOC_Os03g LOC_Os07g Os03g0764	2.33	OS-CX:1.0(	1/7
910 LOC_Os11g no_paralog Os11g0598	2.32	OS-CX:1.0(	1/7
911 LOC_Os01g no_paralog Os01g0935	2.31	OS-GN:1.0	1/7
912 LOC_Os09g no_paralog Os09g0442	2.31	OS-CX:1.0(	1/7
913 LOC_Os05g LOC_Os01g Os05g0458	2.3	SC-GT:1.0(	1/7
914 LOC_Os02g LOC_Os02g Os02g0816	2.3	DM-CX:1.0	1/7
915 LOC_Os11g no_paralog na	2.3	HS-LC:1.0(	1/7
916 LOC_Os03g no_paralog Os03g0669	2.3	HS-LC:1.0(	1/7
917 LOC_Os02g LOC_Os12g Os02g0537	2.3	HS-LC:1.0(	1/7
918 LOC_Os12g LOC_Os11g Os12g0153	2.3	HS-LC:1.0(	1/7

Do not distribute

919	LOC_Os01gLOC_Os02gOs01g0742	2.3	AT-CX:1.0(	1/7
920	LOC_Os05gno_paralogOs05g0170	2.3	AT-CX:1.0(	1/7
921	LOC_Os08gno_paralogOs08g0322	2.3	AT-CX:1.0(	1/7
922	LOC_Os03gLOC_Os07gOs03g0773	2.3	AT-CX:1.0(	1/7
923	LOC_Os08gno_paralogOs08g0541	2.3	AT-CX:1.0(	1/7
924	LOC_Os02gno_paralogOs02g0635	2.3	AT-CX:1.0(	1/7
925	LOC_Os06gLOC_Os02gOs06g0692	2.3	AT-CX:1.0(	1/7
926	LOC_Os07gno_paralogOs07g0187	2.3	DR-CX:1.0(	1/7
927	LOC_Os12gno_paralogOs12g0595	2.3	SC-GT:1.0(	1/7
928	LOC_Os05gno_paralogOs05g0570	2.29	OS-CX:1.0(	1/7
929	LOC_Os05gLOC_Os01gOs05g0587	2.29	SC-GT:1.0(	1/7
930	LOC_Os11gno_paralogOs11g0303	2.29	OS-CX:1.0(	1/7
931	LOC_Os07gno_paralogOs07g0161	2.29	OS-CX:1.0(	1/7
932	LOC_Os03gno_paralogOs03g0624	2.28	OS-CX:1.0(	1/7
933	LOC_Os02gno_paralogOs02g0257	2.28	OS-CX:1.0(	1/7
934	LOC_Os07gno_paralogOs07g0205	2.28	CE-CX:1.0(	1/7
935	LOC_Os07gno_paralogOs07g0155	2.28	AT-CX:1.0(	1/7
936	LOC_Os07gno_paralogOs07g0609	2.28	OS-CX:1.0(	1/7
937	LOC_Os03gno_paralogOs03g0129	2.28	OS-CX:1.0(	1/7
938	LOC_Os01gno_paralogOs01g0728	2.28	OS-CX:1.0(	1/7
939	LOC_Os04gno_paralogOs04g0604	2.28	OS-CX:1.0(	1/7
940	LOC_Os05gno_paralogOs05g0310	2.27	CE-CX:1.0(	1/7
941	LOC_Os02gLOC_Os06gOs02g0255	2.26	SC-GT:1.0(	1/7
942	LOC_Os07gno_paralogOs07g0648	2.26	OS-CX:1.0(	1/7
943	LOC_Os01gno_paralogOs01g0265	2.26	OS-CX:1.0(	1/7
944	LOC_Os12gno_paralogOs12g0538	2.26	OS-CX:1.0(	1/7
945	LOC_Os08gno_paralogOs08g0104	2.26	DR-CX:1.0(	1/7
946	LOC_Os03gno_paralogOs03g0832	2.25	OS-CX:1.0(	1/7
947	LOC_Os07gno_paralogOs07g0414	2.25	DM-HT:1.(	1/7
948	LOC_Os03gno_paralogOs03g0146	2.25	AT-CX:1.0(	1/7
949	LOC_Os11gno_paralogOs11g0521	2.24	OS-CX:1.0(	1/7
950	LOC_Os02gno_paralogOs02g0197	2.23	SC-GT:1.0(	1/7
951	LOC_Os06gLOC_Os05gOs06g0566	2.23	OS-CX:1.0(	1/7
952	LOC_Os08gno_paralogOs08g0109	2.23	OS-CX:1.0(	1/7
953	LOC_Os06gno_paralogOs06g0597	2.22	DM-CX:1.0	1/7
954	LOC_Os01gLOC_Os05gOs01g0951	2.22	SC-GT:1.0(	1/7
955	LOC_Os07gno_paralogOs07g0550	2.22	OS-CX:1.0(	1/7
956	LOC_Os04gno_paralogOs04g0465	2.22	OS-CX:1.0(	1/7
957	LOC_Os02gno_paralogOs02g0822	2.22	OS-CX:1.0(	1/7
958	LOC_Os01gno_paralogOs01g0185	2.22	OS-CX:1.0(	1/7
959	LOC_Os06gno_paralogOs06g0601	2.22	AT-CX:1.0(	1/7
960	LOC_Os01gLOC_Os05gOs01g0580	2.21	AT-CX:1.0(	1/7
961	LOC_Os09gLOC_Os04gOs09g0526	2.21	OS-CX:1.0(	1/7
962	LOC_Os01gno_paralogOs01g0706	2.21	AT-CX:1.0(	1/7
963	LOC_Os07gno_paralogOs07g0689	2.21	SC-GT:1.0(	1/7
964	LOC_Os04gno_paralogOs04g0320	2.21	CE-CX:1.0(	1/7

Do not distribute

965	LOC_Os03gno_paralogOs03g0210	2.2	OS-CX:1.0(	1/7
966	LOC_Os02gno_paralogOs02g0652	2.2	SC-GT:1.0(	1/7
967	LOC_Os08gno_paralogOs08g0295	2.2	SC-GT:1.0(	1/7
968	LOC_Os02gno_paralogOs02g0825	2.2	OS-CX:1.0(	1/7
969	LOC_Os02gno_paralogOs02g0119	2.2	OS-CX:1.0(	1/7
970	LOC_Os01gno_paralogOs01g0114	2.2	OS-CX:1.0(	1/7
971	LOC_Os12gLOC_Os01gOs12g0548	2.2	OS-CX:1.0(	1/7
972	LOC_Os05gLOC_Os03gOs05g0122	2.19	SC-HT:1.0(	1/7
973	LOC_Os06gno_paralogOs06g0720	2.19	OS-CX:1.0(	1/7
974	LOC_Os04gno_paralogOs04g0206	2.19	CE-CX:1.0(	1/7
975	LOC_Os01gLOC_Os05gOs01g0964	2.19	SC-CX:1.0(	1/7
976	LOC_Os03gno_paralogOs03g0216	2.19	OS-CX:1.0(	1/7
977	LOC_Os10gno_paralogOs10g0488	2.18	SC-GT:1.0(	1/7
978	LOC_Os06gno_paralogOs06g0685	2.18	OS-CX:1.0(	1/7
979	LOC_Os04gno_paralogOs04g0206	2.18	CE-CX:1.0(	1/7
980	LOC_Os05gLOC_Os01gOs05g0155	2.18	OS-CX:1.0(	1/7
981	LOC_Os02gno_paralogOs02g0185	2.18	OS-CX:1.0(	1/7
982	LOC_Os04gno_paralogOs04g0402	2.18	DR-CX:1.0(	1/7
983	LOC_Os12gno_paralogOs12g0190	2.18	OS-GN:1.0	1/7
984	LOC_Os05gno_paralogOs05g0469	2.18	SC-HT:1.0(	1/7
985	LOC_Os05gno_paralogOs05g0469	2.18	SC-HT:1.0(	1/7
986	LOC_Os10gno_paralogOs10g0350	2.17	OS-CX:1.0(	1/7
987	LOC_Os01gno_paralogOs01g0772	2.17	OS-CX:1.0(	1/7
988	LOC_Os05gno_paralogOs05g0400	2.17	SC-GT:1.0(	1/7
989	LOC_Os03gLOC_Os01gOs03g0293	2.17	SC-HT:1.0(	1/7
990	LOC_Os05gno_paralogOs05g0176	2.17	SC-HT:1.0(	1/7
991	LOC_Os05gLOC_Os01gOs05g0106	2.17	SC-CX:1.0(	1/7
992	LOC_Os05gno_paralogOs05g0220	2.17	SC-GT:1.0(	1/7
993	LOC_Os04gLOC_Os02gOs04g0485	2.16	OS-GN:1.0	1/7
994	LOC_Os02gLOC_Os04gOs02g0600	2.16	OS-GN:1.0	1/7
995	LOC_Os03gno_paralogOs03g0412	2.16	OS-GN:1.0	1/7
996	LOC_Os03gno_paralogOs03g0318	2.16	OS-GN:1.0	1/7
997	LOC_Os07gno_paralogOs07g0406	2.16	OS-GN:1.0	1/7
998	LOC_Os04gno_paralogOs04g0544	2.16	SC-CX:1.0(	1/7
999	LOC_Os01gno_paralogOs01g0342	2.16	CE-CX:1.0(	1/7
1000	LOC_Os08gno_paralogOs08g0149	2.16	OS-CX:1.0(	1/7
1001	LOC_Os05gno_paralogOs05g0524	2.16	OS-CX:1.0(	1/7
1002	LOC_Os02gno_paralogOs02g0217	2.16	OS-CX:1.0(	1/7
1003	LOC_Os06gno_paralogOs06g0182	2.16	SC-CX:1.0(	1/7
1004	LOC_Os09gno_paralogOs09g0104	2.16	DR-CX:1.0(	1/7
1005	LOC_Os03gLOC_Os05gOs03g0356	2.16	SC-GT:1.0(	1/7
1006	LOC_Os08gno_paralogOs08g0351	2.15	OS-CX:1.0(	1/7
1007	LOC_Os12gno_paralogOs12g0616	2.15	SC-GT:1.0(	1/7
1008	LOC_Os10gno_paralogOs10g0477	2.15	AT-CX:1.0(	1/7
1009	LOC_Os01gno_paralogOs01g0743	2.15	OS-GN:1.0	1/7
1010	LOC_Os05gno_paralogOs05g0558	2.15	OS-CX:1.0(	1/7

Do not distribute

1011	LOC_Os02g no_paralog Os02g0242	2.15	CE-CX:1.0C	1/7
1012	LOC_Os03g no_paralog Os03g0174	2.15	OS-GN:1.0	1/7
1013	LOC_Os01g no_paralog Os01g0665	2.15	CE-CX:1.0C	1/7
1014	LOC_Os08g no_paralog Os08g0180	2.14	OS-CX:1.0(	1/7
1015	LOC_Os04g no_paralog Os04g0321	2.14	CE-CX:1.0C	1/7
1016	LOC_Os03g no_paralog Os03g0349	2.14	DR-CX:1.0(	1/7
1017	LOC_Os02g no_paralog Os02g0457	2.14	DM-CX:1.0	1/7
1018	LOC_Os04g no_paralog na	2.13	CE-CX:1.0C	1/7
1019	LOC_Os04g no_paralog Os04g0117	2.13	OS-CX:1.0(	1/7
1020	LOC_Os01g LOC_Os05g Os01g0844	2.13	SC-GT:1.0C	1/7
1021	LOC_Os02g no_paralog Os02g0242	2.13	CE-CX:1.0C	1/7
1022	LOC_Os05g no_paralog Os05g0214	2.12	OS-CX:1.0(	1/7
1023	LOC_Os03g LOC_Os10g Os03g0101	2.12	OS-GN:1.0	1/7
1024	LOC_Os10g LOC_Os03g Os10g0561	2.12	OS-GN:1.0	1/7
1025	LOC_Os12g no_paralog Os12g0422	2.12	OS-CX:1.0(	1/7
1026	LOC_Os04g no_paralog Os04g0596	2.12	AT-CX:1.0C	1/7
1027	LOC_Os06g no_paralog Os06g0132	2.12	DR-CX:1.0(	1/7
1028	LOC_Os07g LOC_Os03g Os07g0693	2.12	SC-HT:1.0C	1/7
1029	LOC_Os01g no_paralog Os01g0729	2.12	OS-GN:1.0	1/7
1030	LOC_Os07g no_paralog Os07g0477	2.12	OS-CX:1.0(	1/7
1031	LOC_Os05g no_paralog Os05g0506	2.12	OS-CX:1.0(	1/7
1032	LOC_Os02g no_paralog Os02g0827	2.11	OS-CX:1.0(	1/7
1033	LOC_Os03g no_paralog Os03g0604	2.11	OS-CX:1.0(	1/7
1034	LOC_Os05g no_paralog Os05g0196	2.11	AT-CX:1.0C	1/7
1035	LOC_Os09g no_paralog Os09g0119	2.11	CE-CX:1.0C	1/7
1036	LOC_Os03g no_paralog Os03g0781	2.11	OS-GN:1.0	1/7
1037	LOC_Os04g LOC_Os02g Os04g0394	2.11	AT-CX:1.0C	1/7
1038	LOC_Os12g no_paralog Os12g0191	2.1	OS-GN:1.0	1/7
1039	LOC_Os02g no_paralog Os02g0755	2.1	CE-CX:1.0C	1/7
1040	LOC_Os01g no_paralog Os01g0896	2.1	SC-HT:1.0C	1/7
1041	LOC_Os08g no_paralog Os08g0564	2.1	AT-CX:1.0C	1/7
1042	LOC_Os09g LOC_Os08g Os09g0474	2.1	OS-CX:1.0(	1/7
1043	LOC_Os04g no_paralog Os04g0204	2.1	CE-CX:1.0C	1/7
1044	LOC_Os10g no_paralog Os10g0432	2.1	OS-CX:1.0(	1/7
1045	LOC_Os05g no_paralog Os05g0117	2.1	AT-CX:1.0C	1/7
1046	LOC_Os04g no_paralog Os04g0442	2.1	OS-CX:1.0(	1/7
1047	LOC_Os04g no_paralog Os04g0617	2.1	OS-GN:1.0	1/7
1048	LOC_Os03g no_paralog Os03g0114	2.1	OS-CX:1.0(	1/7
1049	LOC_Os11g no_paralog Os11g0298	2.1	DR-CX:1.0(	1/7
1050	LOC_Os05g no_paralog Os05g0144	2.09	OS-CX:1.0(	1/7
1051	LOC_Os07g no_paralog Os07g0197	2.09	DR-CX:1.0(	1/7
1052	LOC_Os01g LOC_Os01g Os01g0742	2.09	DR-CX:1.0(	1/7
1053	LOC_Os01g LOC_Os05g Os01g0940	2.09	DR-CX:1.0(	1/7
1054	LOC_Os08g no_paralog Os08g0199	2.09	OS-GN:1.0	1/7
1055	LOC_Os01g no_paralog Os01g0740	2.09	OS-CX:1.0(	1/7
1056	LOC_Os02g no_paralog Os02g0186	2.09	OS-CX:1.0(	1/7

Do not distribute

1057	LOC_Os12gno_paralogOs12g0560	2.09	AT-CX:1.0(	1/7
1058	LOC_Os01gno_paralogOs01g0174	2.09	DM-CX:1.0	1/7
1059	LOC_Os06gno_paralogOs06g0330	2.09	OS-CX:1.0(	1/7
1060	LOC_Os02gno_paralogOs02g0741	2.09	OS-CX:1.0(	1/7
1061	LOC_Os03gno_paralogOs03g0205	2.09	OS-CX:1.0(	1/7
1062	LOC_Os04gno_paralogOs04g0455	2.09	OS-GN:1.0	1/7
1063	LOC_Os05gno_paralogOs05g0511	2.09	OS-GN:1.0	1/7
1064	LOC_Os01gno_paralogOs01g0769	2.09	OS-GN:1.0	1/7
1065	LOC_Os04gno_paralogOs04g0592	2.08	DR-CX:1.0(	1/7
1066	LOC_Os02gno_paralogOs02g0293	2.08	AT-CX:1.0(	1/7
1067	LOC_Os01gno_paralogOs01g0116	2.08	AT-CX:1.0(	1/7
1068	LOC_Os03gno_paralogOs03g0828	2.08	SC-GT:1.0(	1/7
1069	LOC_Os01gno_paralogOs01g0736	2.08	CE-CX:1.0(	1/7
1070	LOC_Os07gno_paralogOs07g0509	2.08	OS-GN:1.0	1/7
1071	LOC_Os09gno_paralogOs09g0558	2.08	DM-CX:1.0	1/7
1072	LOC_Os02gno_paralogOs02g0821	2.08	SC-HT:1.0(	1/7
1073	LOC_Os02gno_paralogOs02g0511	2.08	SC-GT:1.0(	1/7
1074	LOC_Os11gno_paralogOs11g0621	2.08	OS-CX:1.0(	1/7
1075	LOC_Os04gno_paralogOs04g0668	2.07	OS-CX:1.0(	1/7
1076	LOC_Os02gno_paralogOs02g0791	2.07	CE-CX:1.0(	1/7
1077	LOC_Os07gno_paralogOs07g0296	2.07	OS-CX:1.0(	1/7
1078	LOC_Os02gno_paralogOs02g0623	2.07	OS-GN:1.0	1/7
1079	LOC_Os03gno_paralogOs03g0626	2.06	CE-CX:1.0(	1/7
1080	LOC_Os10gno_paralogOs10g0162	2.06	CE-CX:1.0(	1/7
1081	LOC_Os05gno_paralogOs05g0210	2.06	OS-CX:1.0(	1/7
1082	LOC_Os03gno_paralogOs03g0287	2.06	SC-CX:1.0(	1/7
1083	LOC_Os04gno_paralogOs04g0564	2.06	SC-GT:1.0(	1/7
1084	LOC_Os11gLOC_Os01gOs11g0163	2.06	SC-CX:1.0(	1/7
1085	LOC_Os03gno_paralogOs03g0679	2.06	OS-GN:1.0	1/7
1086	LOC_Os12gno_paralogOs12g0480	2.06	DM-CX:1.0	1/7
1087	LOC_Os02gno_paralogOs02g0294	2.06	SC-CX:1.0(	1/7
1088	LOC_Os07gno_paralogOs07g0250	2.06	CE-CX:1.0(	1/7
1089	LOC_Os03gno_paralogOs03g0678	2.06	OS-CX:1.0(	1/7
1090	LOC_Os12gno_paralogOs12g0242	2.06	AT-CX:1.0(	1/7
1091	LOC_Os12gno_paralogOs12g0431	2.06	AT-CX:1.0(	1/7
1092	LOC_Os05gLOC_Os01gOs05g0526	2.06	CE-CX:1.0(	1/7
1093	LOC_Os11gno_paralogOs11g0602	2.05	OS-CX:1.0(	1/7
1094	LOC_Os03gno_paralogOs03g0169	2.05	OS-GN:1.0	1/7
1095	LOC_Os09gno_paralogOs09g0258	2.05	SC-HT:1.0(	1/7
1096	LOC_Os03gno_paralogOs03g0337	2.05	OS-GN:1.0	1/7
1097	LOC_Os06gno_paralogOs06g0258	2.05	OS-PG:1.0(	1/7
1098	LOC_Os04gno_paralogOs04g0206	2.05	CE-CX:1.0(	1/7
1099	LOC_Os02gno_paralogOs02g0793	2.05	OS-CX:1.0(	1/7
1100	LOC_Os03gno_paralogOs03g0586	2.05	OS-GN:1.0	1/7
1101	LOC_Os02gLOC_Os04gOs02g0437	2.04	SC-HT:1.0(	1/7
1102	LOC_Os05gno_paralogOs05g0520	2.04	SC-HT:1.0(	1/7

Do not distribute

1103	LOC_Os12g	no_paralog	Os12g0640	2.04	SC-HT:1.00	1/7
1104	LOC_Os02g	LOC_Os06g	Os02g0755	2.04	CE-CX:1.00	1/7
1105	LOC_Os05g	no_paralog	Os05g0399	2.04	OS-CX:1.00	1/7
1106	LOC_Os08g	no_paralog	Os08g0485	2.04	OS-CX:1.00	1/7
1107	LOC_Os05g	no_paralog	Os05g0274	2.04	AT-CX:1.00	1/7
1108	LOC_Os04g	LOC_Os02g	Os04g0525	2.04	CE-CX:1.00	1/7
1109	LOC_Os01g	no_paralog	Os01g0301	2.04	OS-CX:1.00	1/7
1110	LOC_Os10g	no_paralog	Os10g0577	2.04	OS-CX:1.00	1/7
1111	LOC_Os08g	no_paralog	na	2.03	AT-CX:1.00	1/7
1112	LOC_Os02g	no_paralog	Os02g0602	2.03	OS-CX:1.00	1/7
1113	LOC_Os03g	no_paralog	Os03g0110	2.03	OS-CX:1.00	1/7
1114	LOC_Os06g	no_paralog	Os06g0143	2.03	SC-CX:1.00	1/7
1115	LOC_Os09g	no_paralog	Os09g0240	2.03	SC-CX:1.00	1/7
1116	LOC_Os03g	no_paralog	Os03g0666	2.03	AT-CX:1.00	1/7
1117	LOC_Os01g	no_paralog	Os01g0539	2.03	OS-CX:1.00	1/7
1118	LOC_Os10g	no_paralog	Os10g0411	2.02	SC-HT:1.00	1/7
1119	LOC_Os03g	no_paralog	Os03g0836	2.02	SC-CX:1.00	1/7
1120	LOC_Os01g	no_paralog	Os01g0153	2.02	AT-CX:1.00	1/7
1121	LOC_Os01g	no_paralog	Os01g0736	2.02	CE-CX:1.00	1/7
1122	LOC_Os01g	LOC_Os01g	Os01g0276	2.02	DR-CX:1.00	1/7
1123	LOC_Os06g	no_paralog	Os06g0526	2.02	SC-GT:1.00	1/7
1124	LOC_Os02g	no_paralog	Os02g0755	2.02	CE-CX:1.00	1/7
1125	LOC_Os01g	LOC_Os12g	Os01g0355	2.02	OS-CX:1.00	1/7
1126	LOC_Os03g	no_paralog	Os03g0165	2.02	AT-CX:1.00	1/7
1127	LOC_Os05g	no_paralog	Os05g0526	2.02	CE-CX:1.00	1/7
1128	LOC_Os03g	no_paralog	Os03g0812	2.02	AT-CX:1.00	1/7
1129	LOC_Os02g	no_paralog	Os02g0100	2.02	AT-CX:1.00	1/7
1130	LOC_Os05g	LOC_Os01g	Os05g0489	2.01	SC-CX:1.00	1/7
1131	LOC_Os02g	no_paralog	Os02g0704	2.01	OS-GN:1.0	1/7
1132	LOC_Os10g	no_paralog	Os10g0510	2.01	SC-CX:1.00	1/7
1133	LOC_Os02g	no_paralog	Os02g0198	2.01	OS-CX:1.00	1/7
1134	LOC_Os03g	no_paralog	Os03g0253	2.01	DM-CX:1.0	1/7
1135	LOC_Os10g	LOC_Os04g	Os10g0495	2.01	OS-CX:1.00	1/7
1136	LOC_Os04g	LOC_Os02g	Os04g0394	2.01	SC-GT:1.00	1/7
1137	LOC_Os04g	LOC_Os08g	Os04g0635	2.01	DM-HT:1.0	1/7
1138	LOC_Os05g	no_paralog	Os05g0223	2.01	AT-CX:1.00	1/7
1139	LOC_Os01g	no_paralog	Os01g0869	2.01	CE-CX:1.00	1/7
1140	LOC_Os05g	LOC_Os05g	Os05g0463	2	OS-CX:1.00	1/7
1141	LOC_Os03g	no_paralog	Os03g0644	2	AT-CX:1.00	1/7
1142	LOC_Os08g	LOC_Os06g	Os08g0471	2	AT-CX:1.00	1/7
1143	LOC_Os01g	no_paralog	Os01g0321	2	OS-CX:1.00	1/7
1144	LOC_Os11g	LOC_Os12g	Os11g0150	2	DM-CX:1.0	1/7
1145	LOC_Os03g	no_paralog	Os03g0146	2	OS-CX:1.00	1/7
1146	LOC_Os10g	no_paralog	Os10g0559	2	AT-CX:1.00	1/7
1147	LOC_Os04g	no_paralog	Os04g0640	1.99	AT-CX:1.00	1/7
1148	LOC_Os03g	no_paralog	Os03g0715	1.99	OS-CX:1.00	1/7

Do not distribute

1149	LOC_Os09gLOC_Os08gOs09g0484	1.99	SC-CX:1.0C	1/7
1150	LOC_Os03gno_paralogOs03g0655	1.99	OS-CX:1.0(	1/7
1151	LOC_Os12gno_paralogOs12g0628	1.99	SC-CX:1.0C	1/7
1152	LOC_Os12gno_paralogOs12g0236	1.99	OS-CX:1.0(	1/7
1153	LOC_Os01gno_paralogOs01g0896	1.99	AT-CX:1.0C	1/7
1154	LOC_Os04gno_paralogOs04g0208	1.98	OS-CX:1.0(	1/7
1155	LOC_Os03gno_paralogOs03g0744	1.98	AT-CX:1.0C	1/7
1156	LOC_Os10gno_paralogOs10g0508	1.98	DR-CX:1.0(	1/7
1157	LOC_Os05gLOC_Os01gOs05g0509	1.98	SC-GT:1.0C	1/7
1158	LOC_Os08gno_paralogOs08g0140	1.98	OS-CX:1.0(	1/7
1159	LOC_Os04gno_paralogOs04g0398	1.98	CE-CX:1.0C	1/7
1160	LOC_Os04gno_paralogOs04g0512	1.97	AT-CX:1.0C	1/7
1161	LOC_Os12gno_paralogOs12g0182	1.97	OS-GN:1.0	1/7
1162	LOC_Os12gLOC_Os11gOs12g0163	1.97	SC-CX:1.0C	1/7
1163	LOC_Os07gno_paralogOs07g0627	1.97	OS-GN:1.0	1/7
1164	LOC_Os03gno_paralogOs03g0681	1.97	SC-HT:1.0C	1/7
1165	LOC_Os01gLOC_Os05gOs01g0618	1.97	SC-GT:1.0C	1/7
1166	LOC_Os01gno_paralogOs01g0377	1.96	OS-PG:1.0(	1/7
1167	LOC_Os01gLOC_Os05gOs01g0839	1.96	SC-CX:1.0C	1/7
1168	LOC_Os05gLOC_Os01gOs05g0460	1.96	SC-CX:1.0C	1/7
1169	LOC_Os07gno_paralogOs07g0211	1.96	AT-CX:1.0C	1/7
1170	LOC_Os12gno_paralogOs12g0141	1.96	OS-CX:1.0(	1/7
1171	LOC_Os01gLOC_Os05gOs01g0734	1.96	CE-CX:1.0C	1/7
1172	LOC_Os09gno_paralogOs09g0530	1.96	OS-CX:1.0(	1/7
1173	LOC_Os10gno_paralogOs10g0419	1.96	DM-CX:1.0	1/7
1174	LOC_Os03gno_paralogOs03g0826	1.96	AT-CX:1.0C	1/7
1175	LOC_Os03gno_paralogOs03g0711	1.96	OS-CX:1.0(	1/7
1176	LOC_Os03gno_paralogOs03g0718	1.96	SC-CX:1.0C	1/7
1177	LOC_Os08gno_paralogOs08g0559	1.96	OS-CX:1.0(	1/7
1178	LOC_Os02gno_paralogna	1.95	CE-CX:1.0C	1/7
1179	LOC_Os08gno_paralogOs08g0545	1.95	CE-CX:1.0C	1/7
1180	LOC_Os01gno_paralogOs01g0369	1.95	OS-CX:1.0(	1/7
1181	LOC_Os03gno_paralogOs03g0255	1.95	AT-CX:1.0C	1/7
1182	LOC_Os07gno_paralogOs07g0134	1.95	SC-GT:1.0C	1/7
1183	LOC_Os08gno_paralogOs08g0406	1.95	OS-CX:1.0(	1/7
1184	LOC_Os12gno_paralogOs12g0541	1.95	OS-GN:1.0	1/7
1185	LOC_Os01gLOC_Os05gOs01g0823	1.95	DR-CX:1.0(	1/7
1186	LOC_Os03gno_paralogOs03g0628	1.95	SC-GT:1.0C	1/7
1187	LOC_Os08gLOC_Os09gOs08g0431	1.95	OS-GN:1.0	1/7
1188	LOC_Os02gno_paralogOs02g0750	1.95	DR-CX:1.0(	1/7
1189	LOC_Os07gno_paralogOs07g0154	1.95	OS-CX:1.0(	1/7
1190	LOC_Os05gno_paralogOs05g0314	1.95	OS-CX:1.0(	1/7
1191	LOC_Os09gno_paralogOs09g0470	1.95	AT-CX:1.0C	1/7
1192	LOC_Os08gno_paralogOs08g0327	1.94	OS-CX:1.0(	1/7
1193	LOC_Os03gno_paralogOs03g0819	1.94	CE-CX:1.0C	1/7
1194	LOC_Os02gno_paralogOs02g0110	1.94	OS-CX:1.0(	1/7

Do not distribute

1195	LOC_Os03g	no_paralog	Os03g0200	1.94	SC-HT:1.00	1/7
1196	LOC_Os04g	no_paralog	Os04g0485	1.94	SC-GT:1.00	1/7
1197	LOC_Os01g	no_paralog	Os01g0199	1.94	SC-HT:1.00	1/7
1198	LOC_Os09g	no_paralog	Os09g0517	1.94	CE-CX:1.00	1/7
1199	LOC_Os07g	no_paralog	Os07g0156	1.94	AT-CX:1.00	1/7
1200	LOC_Os07g	no_paralog	Os07g0157	1.94	AT-CX:1.00	1/7
1201	LOC_Os01g	no_paralog	Os01g0151	1.94	SC-GT:1.00	1/7
1202	LOC_Os03g	no_paralog	Os03g0305	1.94	SC-HT:1.00	1/7
1203	LOC_Os01g	no_paralog	Os01g0948	1.94	SC-HT:1.00	1/7
1204	LOC_Os02g	LOC_Os06g	Os02g0739	1.94	SC-HT:1.00	1/7
1205	LOC_Os01g	no_paralog	Os01g0833	1.94	SC-HT:1.00	1/7
1206	LOC_Os09g	no_paralog	Os09g0266	1.93	OS-CX:1.00	1/7
1207	LOC_Os01g	LOC_Os05g	Os01g0813	1.93	SC-CX:1.00	1/7
1208	LOC_Os03g	no_paralog	Os03g0824	1.93	SC-CX:1.00	1/7
1209	LOC_Os09g	no_paralog	Os09g0518	1.93	CE-CX:1.00	1/7
1210	LOC_Os11g	LOC_Os12g	Os11g0181	1.93	OS-CX:1.00	1/7
1211	LOC_Os09g	LOC_Os08g	Os09g0480	1.93	OS-CX:1.00	1/7
1212	LOC_Os02g	no_paralog	Os02g0729	1.93	OS-CX:1.00	1/7
1213	LOC_Os04g	no_paralog	Os04g0401	1.93	SC-GT:1.00	1/7
1214	LOC_Os09g	no_paralog	Os09g0469	1.92	OS-GN:1.0	1/7
1215	LOC_Os03g	no_paralog	Os03g0133	1.92	CE-CX:1.00	1/7
1216	LOC_Os01g	no_paralog	Os01g0277	1.92	OS-CX:1.00	1/7
1217	LOC_Os01g	no_paralog	Os01g0598	1.92	CE-CX:1.00	1/7
1218	LOC_Os04g	no_paralog	Os04g0555	1.92	AT-CX:1.00	1/7
1219	LOC_Os02g	no_paralog	Os02g0755	1.92	AT-CX:1.00	1/7
1220	LOC_Os12g	no_paralog	Os12g0152	1.92	OS-CX:1.00	1/7
1221	LOC_Os10g	no_paralog	Os10g0560	1.92	SC-CX:1.00	1/7
1222	LOC_Os11g	no_paralog	Os11g0479	1.91	AT-CX:1.00	1/7
1223	LOC_Os05g	no_paralog	Os05g0133	1.91	AT-CX:1.00	1/7
1224	LOC_Os02g	LOC_Os04g	Os02g0601	1.91	OS-CX:1.00	1/7
1225	LOC_Os01g	no_paralog	Os01g0740	1.91	OS-CX:1.00	1/7
1226	LOC_Os10g	no_paralog	Os10g0127	1.91	AT-CX:1.00	1/7
1227	LOC_Os05g	no_paralog	Os05g0392	1.91	OS-CX:1.00	1/7
1228	LOC_Os04g	no_paralog	Os04g0398	1.91	OS-CX:1.00	1/7
1229	LOC_Os04g	no_paralog	Os04g0549	1.91	OS-CX:1.00	1/7
1230	LOC_Os11g	LOC_Os12g	Os11g0114	1.91	OS-CX:1.00	1/7
1231	LOC_Os11g	no_paralog	Os11g0558	1.91	OS-CX:1.00	1/7
1232	LOC_Os08g	no_paralog	Os08g0115	1.91	AT-CX:1.00	1/7
1233	LOC_Os01g	no_paralog	Os01g0771	1.9	OS-GN:1.0	1/7
1234	LOC_Os02g	no_paralog	Os02g0131	1.9	OS-GN:1.0	1/7
1235	LOC_Os05g	no_paralog	Os05g0449	1.9	OS-GN:1.0	1/7
1236	LOC_Os03g	no_paralog	Os03g0749	1.9	OS-GN:1.0	1/7
1237	LOC_Os03g	no_paralog	Os03g0749	1.9	OS-GN:1.0	1/7
1238	LOC_Os03g	no_paralog	Os03g0749	1.9	OS-GN:1.0	1/7
1239	LOC_Os07g	LOC_Os03g	Os07g0206	1.9	SC-GT:1.00	1/7
1240	LOC_Os07g	LOC_Os03g	Os07g0673	1.9	AT-CX:1.00	1/7

Do not distribute

1241	LOC_Os04gno_paralogOs04g0392	1.9	OS-CX:1.0(	1/7
1242	LOC_Os04gno_paralogOs04g0645	1.9	OS-PG:1.0(	1/7
1243	LOC_Os11gno_paralogOs11g0148	1.9	OS-CX:1.0(	1/7
1244	LOC_Os05gno_paralogOs05g0140	1.9	AT-CX:1.0(	1/7
1245	LOC_Os08gno_paralogOs08g0385	1.9	AT-CX:1.0(	1/7
1246	LOC_Os08gno_paralogOs08g0486	1.9	AT-CX:1.0(	1/7
1247	LOC_Os06gno_paralogOs06g0691	1.9	OS-CX:1.0(	1/7
1248	LOC_Os01gno_paralogOs01g0191	1.9	OS-CX:1.0(	1/7
1249	LOC_Os04gno_paralogOs04g0658	1.9	OS-CX:1.0(	1/7
1250	LOC_Os01gno_paralogOs01g0620	1.89	CE-CX:1.0(	1/7
1251	LOC_Os03gno_paralogOs03g0129	1.89	AT-CX:1.0(	1/7
1252	LOC_Os02gno_paralogOs02g0168	1.89	CE-CX:1.0(	1/7
1253	LOC_Os02gno_paralogOs02g0490	1.89	CE-CX:1.0(	1/7
1254	LOC_Os03gno_paralogOs03g0733	1.89	SC-CX:1.0(	1/7
1255	LOC_Os04gno_paralogOs04g0444	1.89	OS-CX:1.0(	1/7
1256	LOC_Os04gLOC_Os02gOs04g0605	1.89	DM-HT:1.(	1/7
1257	LOC_Os10gno_paralogOs10g0488	1.89	SC-GT:1.0(	1/7
1258	LOC_Os03gno_paralogOs03g0722	1.89	OS-CX:1.0(	1/7
1259	LOC_Os01gLOC_Os06gOs01g0581	1.89	OS-CX:1.0(	1/7
1260	LOC_Os03gno_paralogOs03g0563	1.89	OS-GN:1.0	1/7
1261	LOC_Os05gLOC_Os01gOs05g0592	1.89	AT-CX:1.0(	1/7
1262	LOC_Os05gno_paralogOs05g0306	1.89	AT-CX:1.0(	1/7
1263	LOC_Os03gLOC_Os02gOs03g0120	1.89	AT-CX:1.0(	1/7
1264	LOC_Os12gno_paralogOs12g0488	1.88	OS-CX:1.0(	1/7
1265	LOC_Os03gno_paralogOs03g0133	1.88	CE-CX:1.0(	1/7
1266	LOC_Os12gno_paralogOs12g0498	1.88	SC-GT:1.0(	1/7
1267	LOC_Os12gno_paralogOs12g0406	1.88	SC-HT:1.0(	1/7
1268	LOC_Os04gno_paralogOs04g0623	1.88	AT-CX:1.0(	1/7
1269	LOC_Os01gLOC_Os05gOs01g0220	1.88	OS-CX:1.0(	1/7
1270	LOC_Os06gno_paralogOs06g0152	1.88	OS-GN:1.0	1/7
1271	LOC_Os05gLOC_Os01gOs05g0522	1.88	DR-CX:1.0(	1/7
1272	LOC_Os10gno_paralogOs10g0547	1.87	SC-GT:1.0(	1/7
1273	LOC_Os03gno_paralogOs03g0356	1.87	AT-CX:1.0(	1/7
1274	LOC_Os01gno_paralog na	1.87	CE-CX:1.0(	1/7
1275	LOC_Os05gno_paralogOs05g0527	1.87	CE-CX:1.0(	1/7
1276	LOC_Os07gLOC_Os03gOs07g0137	1.87	OS-CX:1.0(	1/7
1277	LOC_Os06gno_paralogOs06g0240	1.87	OS-CX:1.0(	1/7
1278	LOC_Os01gno_paralogOs01g0510	1.87	OS-CX:1.0(	1/7
1279	LOC_Os08gno_paralogOs08g0156	1.87	SC-GT:1.0(	1/7
1280	LOC_Os10gno_paralogOs10g0495	1.87	OS-CX:1.0(	1/7
1281	LOC_Os12gLOC_Os01gOs12g0158	1.87	AT-CX:1.0(	1/7
1282	LOC_Os03gno_paralogOs03g0209	1.87	OS-GN:1.0	1/7
1283	LOC_Os11gno_paralogOs11g0512	1.87	OS-CX:1.0(	1/7
1284	LOC_Os10gno_paralogOs10g0548	1.87	SC-GT:1.0(	1/7
1285	LOC_Os08gno_paralogOs08g0113	1.87	OS-CX:1.0(	1/7
1286	LOC_Os05gno_paralogOs05g0113	1.87	SC-GT:1.0(	1/7

Do not distribute

1287	LOC_Os03g	no_paralog	Os03g0833	1.87	OS-CX:1.0(	1/7
1288	LOC_Os05g	no_paralog	Os05g0362	1.86	OS-CX:1.0(	1/7
1289	LOC_Os08g	no_paralog	Os08g0530	1.86	SC-HT:1.0(	1/7
1290	LOC_Os09g	no_paralog	Os09g0518	1.86	CE-CX:1.0(	1/7
1291	LOC_Os02g	LOC_Os06g	Os02g0803	1.86	AT-CX:1.0(	1/7
1292	LOC_Os03g	no_paralog	Os03g0281	1.86	SC-GT:1.0(	1/7
1293	LOC_Os01g	LOC_Os05g	Os01g0662	1.86	OS-GN:1.0	1/7
1294	LOC_Os07g	no_paralog	Os07g0481	1.86	OS-CX:1.0(	1/7
1295	LOC_Os08g	no_paralog	Os08g0547	1.86	AT-CX:1.0(	1/7
1296	LOC_Os02g	no_paralog	Os02g0492	1.85	OS-CX:1.0(	1/7
1297	LOC_Os04g	no_paralog	Os04g0625	1.85	SC-GT:1.0(	1/7
1298	LOC_Os02g	no_paralog	Os02g0664	1.85	OS-CX:1.0(	1/7
1299	LOC_Os08g	no_paralog	Os08g0160	1.85	OS-CX:1.0(	1/7
1300	LOC_Os05g	no_paralog	Os05g0316	1.85	OS-CX:1.0(	1/7
1301	LOC_Os01g	no_paralog	Os01g0879	1.85	OS-CX:1.0(	1/7
1302	LOC_Os01g	LOC_Os01g	Os01g0183	1.85	SC-GT:1.0(	1/7
1303	LOC_Os05g	LOC_Os01g	Os05g0535	1.85	SC-GT:1.0(	1/7
1304	LOC_Os02g	no_paralog	Os02g0788	1.85	SC-HT:1.0(	1/7
1305	LOC_Os01g	LOC_Os01g	Os01g0743	1.85	SC-HT:1.0(	1/7
1306	LOC_Os05g	LOC_Os01g	Os05g0490	1.85	SC-HT:1.0(	1/7
1307	LOC_Os04g	no_paralog	Os04g0652	1.85	DR-CX:1.0(	1/7
1308	LOC_Os01g	LOC_Os05g	Os01g0695	1.85	CE-CX:1.0(	1/7
1309	LOC_Os02g	no_paralog	Os02g0287	1.85	SC-HT:1.0(	1/7
1310	LOC_Os03g	no_paralog	Os03g0347	1.84	OS-GN:1.0	1/7
1311	LOC_Os07g	LOC_Os03g	Os07g0173	1.84	OS-CX:1.0(	1/7
1312	LOC_Os05g	no_paralog	Os05g0264	1.84	AT-CX:1.0(	1/7
1313	LOC_Os09g	no_paralog	na	1.84	OS-GN:1.0	1/7
1314	LOC_Os05g	no_paralog	Os05g0155	1.83	SC-GT:1.0(	1/7
1315	LOC_Os06g	no_paralog	Os06g0343	1.83	CE-CX:1.0(	1/7
1316	LOC_Os07g	no_paralog	Os07g0486	1.83	SC-GT:1.0(	1/7
1317	LOC_Os06g	LOC_Os01g	Os06g0551	1.83	SC-GT:1.0(	1/7
1318	LOC_Os03g	no_paralog	Os03g0254	1.83	OS-CX:1.0(	1/7
1319	LOC_Os09g	no_paralog	Os09g0497	1.83	SC-GT:1.0(	1/7
1320	LOC_Os03g	no_paralog	Os03g0111	1.83	OS-GN:1.0	1/7
1321	LOC_Os07g	no_paralog	Os07g0163	1.83	OS-GN:1.0	1/7
1322	LOC_Os10g	no_paralog	Os10g0506	1.83	OS-GN:1.0	1/7
1323	LOC_Os03g	no_paralog	Os03g0335	1.83	OS-GN:1.0	1/7
1324	LOC_Os03g	no_paralog	Os03g0694	1.83	OS-GN:1.0	1/7
1325	LOC_Os01g	no_paralog	na	1.83	CE-CX:1.0(	1/7
1326	LOC_Os03g	LOC_Os03g	Os03g0743	1.83	SC-GT:1.0(	1/7
1327	LOC_Os04g	no_paralog	Os04g0291	1.83	SC-GT:1.0(	1/7
1328	LOC_Os01g	no_paralog	Os01g0697	1.82	CE-CX:1.0(	1/7
1329	LOC_Os12g	no_paralog	Os12g0485	1.82	SC-GT:1.0(	1/7
1330	LOC_Os09g	LOC_Os08g	Os09g0284	1.82	OS-GN:1.0	1/7
1331	LOC_Os03g	LOC_Os12g	Os03g0648	1.82	SC-GT:1.0(	1/7
1332	LOC_Os05g	LOC_Os01g	Os05g0182	1.82	OS-GN:1.0	1/7

Do not distribute

1333	LOC_Os01g	LOC_Os05g	Os01g0185	1.82	OS-GN:1.0	1/7
1334	LOC_Os04g	no_paralog	Os04g0169	1.82	OS-CX:1.0(	1/7
1335	LOC_Os04g	no_paralog	Os04g0291	1.82	SC-GT:1.0(	1/7
1336	LOC_Os01g	no_paralog	Os01g0776	1.81	OS-CX:1.0(	1/7
1337	LOC_Os04g	no_paralog	Os04g0577	1.81	SC-GT:1.0(	1/7
1338	LOC_Os09g	no_paralog	Os09g0558	1.81	SC-HT:1.0(	1/7
1339	LOC_Os08g	LOC_Os09g	Os08g0398	1.81	OS-GN:1.0	1/7
1340	LOC_Os06g	LOC_Os02g	Os06g0526	1.81	SC-GT:1.0(	1/7
1341	LOC_Os12g	no_paralog	Os12g0506	1.81	SC-GT:1.0(	1/7
1342	LOC_Os07g	no_paralog	Os07g0685	1.81	OS-CX:1.0(	1/7
1343	LOC_Os03g	no_paralog	Os03g0710	1.81	SC-GT:1.0(	1/7
1344	LOC_Os03g	no_paralog	na	1.81	OS-GN:1.0	1/7
1345	LOC_Os03g	LOC_Os03g	Os03g0669	1.81	OS-CX:1.0(	1/7
1346	LOC_Os05g	no_paralog	Os05g0311	1.81	SC-HT:1.0(	1/7
1347	LOC_Os01g	no_paralog	Os01g0887	1.81	OS-CX:1.0(	1/7
1348	LOC_Os09g	no_paralog	Os09g0529	1.81	SC-GT:1.0(	1/7
1349	LOC_Os12g	no_paralog	Os12g0285	1.8	SC-GT:1.0(	1/7
1350	LOC_Os05g	LOC_Os01g	Os05g0419	1.8	OS-CX:1.0(	1/7
1351	LOC_Os07g	no_paralog	Os07g0128	1.8	OS-CX:1.0(	1/7
1352	LOC_Os08g	no_paralog	Os08g0557	1.8	OS-CX:1.0(	1/7
1353	LOC_Os10g	no_paralog	Os10g0131	1.8	OS-CX:1.0(	1/7
1354	LOC_Os03g	no_paralog	Os03g0721	1.8	OS-CX:1.0(	1/7
1355	LOC_Os12g	no_paralog	Os12g0626	1.8	SC-HT:1.0(	1/7
1356	LOC_Os02g	LOC_Os06g	Os02g0722	1.79	DR-CX:1.0(	1/7
1357	LOC_Os08g	no_paralog	Os08g0196	1.79	OS-CX:1.0(	1/7
1358	LOC_Os05g	LOC_Os01g	Os05g0477	1.79	DR-CX:1.0(	1/7
1359	LOC_Os06g	no_paralog	Os06g0505	1.79	OS-CX:1.0(	1/7
1360	LOC_Os02g	no_paralog	Os02g0822	1.79	OS-CX:1.0(	1/7
1361	LOC_Os11g	no_paralog	Os11g0615	1.79	OS-CX:1.0(	1/7
1362	LOC_Os01g	LOC_Os05g	Os01g0811	1.79	SC-HT:1.0(	1/7
1363	LOC_Os12g	no_paralog	Os12g0288	1.79	OS-CX:1.0(	1/7
1364	LOC_Os03g	no_paralog	Os03g0669	1.79	OS-GN:1.0	1/7
1365	LOC_Os04g	no_paralog	Os04g0317	1.78	OS-CX:1.0(	1/7
1366	LOC_Os05g	LOC_Os01g	Os05g0568	1.78	OS-GN:1.0	1/7
1367	LOC_Os01g	no_paralog	Os01g0276	1.78	OS-CX:1.0(	1/7
1368	LOC_Os02g	no_paralog	Os02g0144	1.78	OS-CX:1.0(	1/7
1369	LOC_Os06g	no_paralog	Os06g0116	1.78	OS-CX:1.0(	1/7
1370	LOC_Os10g	no_paralog	Os10g0498	1.78	OS-CX:1.0(	1/7
1371	LOC_Os06g	no_paralog	Os06g0252	1.77	SC-CX:1.0(	1/7
1372	LOC_Os03g	no_paralog	Os03g0172	1.77	OS-CX:1.0(	1/7
1373	LOC_Os02g	LOC_Os06g	Os02g0784	1.77	OS-GN:1.0	1/7
1374	LOC_Os06g	LOC_Os02g	Os06g0173	1.77	OS-GN:1.0	1/7
1375	LOC_Os06g	LOC_Os02g	Os06g0192	1.77	OS-GN:1.0	1/7
1376	LOC_Os05g	LOC_Os01g	Os05g0458	1.77	OS-GN:1.0	1/7
1377	LOC_Os01g	no_paralog	Os01g0618	1.77	OS-GN:1.0	1/7
1378	LOC_Os06g	no_paralog	Os06g0725	1.77	OS-GN:1.0	1/7

Do not distribute

1379	LOC_Os02gLOC_Os06gOs02g0199	1.77	OS-GN:1.0	1/7
1380	LOC_Os04gno_paralogOs04g0466	1.77	OS-GN:1.0	1/7
1381	LOC_Os06gLOC_Os02gOs06g0600	1.77	OS-GN:1.0	1/7
1382	LOC_Os01gno_paralogOs01g0574	1.77	OS-GN:1.0	1/7
1383	LOC_Os02gno_paralogOs02g0649	1.77	OS-GN:1.0	1/7
1384	LOC_Os03gLOC_Os07gOs03g0298	1.77	OS-GN:1.0	1/7
1385	LOC_Os06gno_paralogOs06g0229	1.77	OS-GN:1.0	1/7
1386	LOC_Os01gLOC_Os05gOs01g0842	1.77	OS-GN:1.0	1/7
1387	LOC_Os02gLOC_Os06gOs02g0205	1.77	OS-GN:1.0	1/7
1388	LOC_Os06gno_paralogOs06g0669	1.77	OS-GN:1.0	1/7
1389	LOC_Os02gno_paralogOs02g0325	1.77	OS-GN:1.0	1/7
1390	LOC_Os02gLOC_Os06gOs02g0803	1.77	OS-GN:1.0	1/7
1391	LOC_Os07gLOC_Os03gOs07g0691	1.77	OS-GN:1.0	1/7
1392	LOC_Os01gno_paralogOs01g0574	1.77	OS-GN:1.0	1/7
1393	LOC_Os05gLOC_Os01gOs05g0485	1.76	SC-GT:1.0(	1/7
1394	LOC_Os10gLOC_Os04gOs10g0516	1.76	OS-CX:1.0(	1/7
1395	LOC_Os09gno_paralogOs09g0123	1.76	OS-CX:1.0(	1/7
1396	LOC_Os07gLOC_Os03gOs07g0635	1.76	SC-HT:1.0(	1/7
1397	LOC_Os05gno_paralogOs05g0193	1.76	OS-CX:1.0(	1/7
1398	LOC_Os01gno_paralogOs01g0246	1.76	OS-CX:1.0(	1/7
1399	LOC_Os03gno_paralogOs03g0109	1.76	SC-HT:1.0(	1/7
1400	LOC_Os06gLOC_Os02gOs06g0527	1.76	OS-CX:1.0(	1/7
1401	LOC_Os02gLOC_Os10gOs02g0643	1.76	SC-GT:1.0(	1/7
1402	LOC_Os08gno_paralogOs08g0308	1.76	SC-HT:1.0(	1/7
1403	LOC_Os01gLOC_Os05gOs01g0315	1.76	OS-CX:1.0(	1/7
1404	LOC_Os03gno_paralogOs03g0783	1.75	OS-CX:1.0(	1/7
1405	LOC_Os02gno_paralogOs02g0571	1.75	OS-CX:1.0(	1/7
1406	LOC_Os01gno_paralogOs01g0106	1.75	SC-GT:1.0(	1/7
1407	LOC_Os10gno_paralogOs10g0524	1.75	SC-GT:1.0(	1/7
1408	LOC_Os03gno_paralogOs03g0826	1.75	OS-CX:1.0(	1/7
1409	LOC_Os10gno_paralogOs10g0135	1.75	OS-CX:1.0(	1/7
1410	LOC_Os10gno_paralogOs10g0569	1.75	SC-HT:1.0(	1/7
1411	LOC_Os01gLOC_Os04gOs01g0920	1.75	OS-CX:1.0(	1/7
1412	LOC_Os07gLOC_Os03gOs07g0655	1.75	OS-CX:1.0(	1/7
1413	LOC_Os01gLOC_Os11gOs01g0817	1.75	SC-GT:1.0(	1/7
1414	LOC_Os06gLOC_Os02gOs06g0246	1.75	SC-HT:1.0(	1/7
1415	LOC_Os04gno_paralogOs04g0272	1.75	OS-CX:1.0(	1/7
1416	LOC_Os09gno_paralogOs09g0405	1.74	OS-CX:1.0(	1/7
1417	LOC_Os10gno_paralogOs10g0148	1.74	OS-CX:1.0(	1/7
1418	LOC_Os06gno_paralogOs06g0129	1.74	SC-GT:1.0(	1/7
1419	LOC_Os06gno_paralogOs06g0708	1.74	SC-GT:1.0(	1/7
1420	LOC_Os10gno_paralogOs10g0414	1.74	OS-GN:1.0	1/7
1421	LOC_Os05gno_paralogOs05g0256	1.74	SC-HT:1.0(	1/7

Do not distribute

ks related leaf rolling under drought

[Linked_query]	[GO_descriptions]
LOC_Os04g04020	transport;intracellular protein transport;ER to Golgi vesicle-mediate
LOC_Os03g57300 LOC	transport;intracellular protein transport;ER to Golgi vesicle-mediate
LOC_Os03g57300 LOC	intracellular protein transport;ER to Golgi vesicle-mediated transpo
LOC_Os04g04020	intracellular protein transport;ER to Golgi vesicle-mediated transpo
LOC_Os04g04020	na
LOC_Os04g04020	intracellular protein transport;vesicle-mediated transport
LOC_Os04g04020	na
LOC_Os03g57300 LOC	protein amino acid phosphorylation;phosphorylation
LOC_Os04g04020	na
LOC_Os04g04020	transport;intracellular protein transport;protein transport;vesicle-m
LOC_Os04g04020	transport;intracellular protein transport;protein transport;vesicle-m
LOC_Os04g04020	intracellular protein transport;transport;protein transport;vesicle-m
LOC_Os04g04020 LOC	transport;intracellular protein transport;protein transport;vesicle-m
LOC_Os04g04020 LOC	transport;ion transport;glucose mediated signaling pathway;ATP hy
LOC_Os04g04020	na
LOC_Os04g04020 LOC	fatty acid biosynthetic process;sterol biosynthetic process;oxidation
LOC_Os04g04020 LOC	fatty acid biosynthetic process;sterol biosynthetic process;oxidation
LOC_Os06g36850	na
LOC_Os06g36850	'de novo' L-methionine biosynthetic process
LOC_Os03g57300 LOC	transport;ER to Golgi vesicle-mediated transport
LOC_Os03g57300 LOC	na
LOC_Os03g57300 LOC	translational initiation;RNA metabolic process
LOC_Os03g57300 LOC	na
LOC_Os03g57300 LOC	translational initiation;RNA metabolic process
LOC_Os03g57300 LOC	protein amino acid glycosylation;cellulose biosynthetic process;Golg
LOC_Os04g04020 LOC	transport;intracellular protein transport;protein transport;vesicle-m
LOC_Os04g04020 LOC	transport;ion transport;ATP hydrolysis coupled proton transport;pro
LOC_Os04g04020 LOC	autophagy;transport;protein transport;defense response to fungus
LOC_Os03g57300 LOC	detection of gravity;gravitropism;amyloplast organization;negative
LOC_Os04g04020 LOC	autophagy;transport;protein transport
LOC_Os03g57300 LOC	intracellular protein transport;vesicle-mediated transport
LOC_Os04g04020	regulation of protein catabolic process;regulation of catalytic activit
LOC_Os03g57300 LOC	na
LOC_Os03g57300 LOC	nucleotide-excision repair;proteasomal ubiquitin-dependent protei
LOC_Os04g04020 LOC	autophagy;transport;protein transport
LOC_Os03g57300 LOC	entrainment of circadian clock by photoperiod;photoperiodism, flov
LOC_Os03g57300 LOC	nuclear mRNA splicing, via spliceosome;transcription initiation;regu
LOC_Os03g57300 LOC	production of ta-siRNAs involved in RNA interference;primary micro
LOC_Os03g57300 LOC	production of ta-siRNAs involved in RNA interference;primary micro
LOC_Os04g04020 LOC	intracellular protein transport;vesicle-mediated transport
LOC_Os03g57300 LOC	na
LOC_Os03g57300 LOC	na
LOC_Os03g57300 LOC	DNA replication;DNA topological change;chromatin remodeling;chr
LOC_Os04g04020 LOC	transport;intracellular protein transport;protein transport;vesicle-m

Do not distribute

LOC_Os03g57300 LOC	transport;ER to Golgi vesicle-mediated transport
LOC_Os04g04020 LOC	intracellular protein transport;vesicle-mediated transport;vacuolar t
LOC_Os03g57300 LOC	na
LOC_Os04g04020	ATP catabolic process;ATP biosynthetic process;cation transport;res
LOC_Os03g57300 LOC	phosphorylation;cellular response to glucose starvation;positive reg
LOC_Os04g04020 LOC	response to stress;response to virus;response to hydrogen peroxide
LOC_Os04g04020 LOC	na
LOC_Os04g04020 LOC	proteolysis;ubiquitin-dependent protein catabolic process
LOC_Os03g57300 LOC	mitosis;cell growth;protein stabilization
LOC_Os04g04020 LOC	response to stress
LOC_Os04g04020 LOC	na
LOC_Os03g57300 LOC	transport;ER to Golgi vesicle-mediated transport
LOC_Os04g04020	transport;intracellular protein transport;protein transport;vesicle-m
LOC_Os04g04020 LOC	response to stress;response to heat;response to cadmium ion
LOC_Os04g04020	ATP catabolic process;ATP biosynthetic process;cation transport;res
LOC_Os04g04020 LOC	response to stress;response to virus;response to heat;response to c
LOC_Os04g04020 LOC	tRNA processing;oxidation reduction
LOC_Os04g04020	ATP catabolic process;ATP biosynthetic process;cation transport;res
LOC_Os04g04020	ATP catabolic process;ATP biosynthetic process;cation transport;res
LOC_Os06g36850 LOC	protein amino acid phosphorylation
LOC_Os04g04020	ATP catabolic process;ATP biosynthetic process;cation transport;res
LOC_Os04g04020 LOC	na
LOC_Os04g04020	transport;intracellular protein transport;protein transport;vesicle-m
LOC_Os04g04020 LOC	response to stress;response to virus;response to heat;response to b
LOC_Os04g04020 LOC	GTP catabolic process;microtubule-based process;response to light
LOC_Os04g04020 LOC	GTP catabolic process;microtubule-based process;response to cadm
LOC_Os04g04020	ATP catabolic process;ATP biosynthetic process;cation transport;res
LOC_Os04g04020 LOC	GTP catabolic process;microtubule-based process;response to cadm
LOC_Os03g57300 LOC	vacuole organization;pollen development;cellular protein metabolic
LOC_Os03g57300 LOC	DNA repair;microtubule cytoskeleton organization;mitotic cell cycle
LOC_Os04g04020 LOC	GTP catabolic process;microtubule-based process;protein polymeriz
LOC_Os04g04020	ATP catabolic process;ATP biosynthetic process;cation transport;me
LOC_Os03g57300 LOC	na
LOC_Os04g04020 LOC	GTP catabolic process;microtubule-based process;protein polymeriz
LOC_Os04g04020 LOC	GTP catabolic process;microtubule-based process;protein polymeriz
LOC_Os04g04020 LOC	GTP catabolic process;microtubule-based process;response to cold;
LOC_Os04g04020 LOC	gluconeogenesis;GTP catabolic process;cytoskeleton organization;m
LOC_Os04g04020	ATP catabolic process;ATP biosynthetic process;cation transport;res
LOC_Os04g04020 LOC	na
LOC_Os04g04020	cation transport;proanthocyanidin biosynthetic process;vacuolar ac
LOC_Os02g45250 LOC	regulation of transcription, DNA-dependent
LOC_Os04g04020	transport;vesicle-mediated transport
LOC_Os04g04020	transport
LOC_Os04g04020	na
LOC_Os04g04020 LOC	regulation of Rab GTPase activity;positive regulation of Rab GTPase
LOC_Os03g57300 LOC	RNA polyadenylation

Do not distribute

LOC_Os04g04020	transport
LOC_Os02g45250 LOC	proteolysis;chromosome segregation;synapsis;reciprocal meiotic re
LOC_Os04g04020 LOC	transcription, DNA-dependent
LOC_Os03g57300 LOC	protein folding;protein targeting to vacuole;endocytosis;endosome
LOC_Os02g45250 LOC	transcription, DNA-dependent;regulation of transcription, DNA-dep
LOC_Os04g04020	transport
LOC_Os09g23200 LOC	oligopeptide transport;transmembrane transport
LOC_Os04g04020	cysteine biosynthetic process from serine;cellular amino acid biosyr
LOC_Os04g04020 LOC	autophagy
LOC_Os04g04020	na
LOC_Os03g57300 LOC	na
LOC_Os04g04020 LOC	na
LOC_Os04g04020 LOC	ubiquitin-dependent protein catabolic process;protein targeting to
LOC_Os04g04020	amino acid transmembrane transport
LOC_Os04g04020	amino acid transmembrane transport
LOC_Os04g04020	na
LOC_Os03g57300 LOC	protein amino acid N-linked glycosylation;regulation of mitotic cell
LOC_Os03g57300 LOC	regulation of ARF protein signal transduction;organ morphogenesis;
LOC_Os06g36850 LOC	methionine biosynthetic process
LOC_Os04g04020	transport;intracellular protein transport;protein transport;vesicle-m
LOC_Os04g04020 LOC	autophagy;pollen germination
LOC_Os03g57300 LOC	protein amino acid phosphorylation;phosphorylation
LOC_Os03g57300 LOC	protein amino acid phosphorylation;phosphorylation;response to o:
LOC_Os04g04020 LOC	nucleotide-sugar transport;GDP-mannose transport;transmembran
LOC_Os03g57300 LOC	nuclear mRNA splicing, via spliceosome
LOC_Os06g36850 LOC	metabolic process;methionine biosynthetic process
LOC_Os04g04020	cysteine biosynthetic process from serine;aging;cellular amino acid
LOC_Os04g04020	transport
LOC_Os09g39670	na
LOC_Os04g04020 LOC	autophagy;pollen germination;autophagic vacuole assembly;proteir
LOC_Os03g57300 LOC	na
LOC_Os03g57300 LOC	exocytosis
LOC_Os04g04020	na
LOC_Os03g57300 LOC	lipid metabolic process;fatty acid metabolic process;fatty acid biosy
LOC_Os04g04020	amino acid transmembrane transport
LOC_Os04g04020 LOC	RNA methylation;plant-type cell wall organization;unidimensional c
LOC_Os04g04020	transport
LOC_Os02g45250	transcription, DNA-dependent;regulation of transcription, DNA-dep
LOC_Os03g57300 LOC	cation transport;calcium ion transmembrane transport;transport;io
LOC_Os03g57300 LOC	cation transport;calcium ion transmembrane transport;pollen devel
LOC_Os04g04020 LOC	na
LOC_Os04g04020 LOC	GTP catabolic process;microtubule-based process;protein polymeri
LOC_Os03g57300 LOC	negative regulation of microtubule depolymerization;protein stabili
LOC_Os04g04020	cysteine biosynthetic process from serine;cellular amino acid biosyr
LOC_Os04g04020	na
LOC_Os04g04020	na

Do not distribute

LOC_Os09g39670	metabolic process
LOC_Os09g39670	metabolic process
LOC_Os09g39670	metabolic process
LOC_Os09g39670	metabolic process
LOC_Os09g39670	metabolic process
LOC_Os09g39670	metabolic process
LOC_Os09g39670	metabolic process
LOC_Os09g39670	metabolic process
LOC_Os09g39670	metabolic process
LOC_Os09g39670	metabolic process
LOC_Os09g39670	metabolic process
LOC_Os09g39670	metabolic process
LOC_Os09g39670	metabolic process
LOC_Os09g39670	metabolic process
LOC_Os04g04020 LOC	GTP catabolic process;microtubule-based process;protein polymeriz
LOC_Os04g04020 LOC	na
LOC_Os09g39670	na
LOC_Os03g57300 LOC	na
LOC_Os03g57300 LOC	regulation of transcription, DNA-dependent
LOC_Os04g04020	translation;response to UV-B;developmental process;cellular respor
LOC_Os04g04020	regulation of protein catabolic process;regulation of catalytic activit
LOC_Os04g04020 LOC	GTP catabolic process;microtubule-based process;response to cadm
LOC_Os03g57300 LOC	pollen germination;ovule development;pollen development
LOC_Os09g39670	metabolic process
LOC_Os04g04020 LOC	inositol trisphosphate metabolic process;inositol phosphate dephos
LOC_Os04g04020 LOC	root hair cell tip growth;response to salt stress
LOC_Os04g04020	regulation of protein catabolic process;regulation of catalytic activit
LOC_Os04g04020	regulation of protein catabolic process;regulation of catalytic activit
LOC_Os03g57300	RNA processing;polarity specification of adaxial/abaxial axis;produc
LOC_Os09g23200 LOC	transcription, DNA-dependent;regulation of transcription, DNA-dep
LOC_Os09g39670	metabolic process
LOC_Os09g39670	metabolic process
LOC_Os09g39670	metabolic process
LOC_Os09g39670	xenobiotic metabolic process;metabolic process;response to toxin;r
LOC_Os04g04020 LOC	na
LOC_Os04g04020 LOC	phospholipid biosynthetic process
LOC_Os04g04020	transport
LOC_Os06g36850	protein folding;response to salt stress;response to cadmium ion;res
LOC_Os09g39670	na
LOC_Os09g39670	na
LOC_Os04g04020 LOC	protein amino acid phosphorylation;phosphorylation;detection of n
LOC_Os03g57300 LOC	transport;ion transport;cation transport;calcium ion transport;calciu
LOC_Os09g39670	metabolic process
LOC_Os04g04020	cysteine biosynthetic process from serine;cyanide catabolic process
LOC_Os09g39670	metabolic process
LOC_Os04g04020	reproduction;1,3-beta-glucan biosynthetic process;defense respons

Do not distribute

LOC_Os04g04020	na
LOC_Os03g57300 LOC	transport;ER to Golgi vesicle-mediated transport;intra-Golgi vesicle-
LOC_Os03g57300 LOC	tRNA modification
LOC_Os03g57300	vacuole organization;pollen development;cellular protein metabolic
LOC_Os09g39670	metabolic process
LOC_Os09g39670	metabolic process
LOC_Os09g39670	metabolic process;oxidation reduction
LOC_Os04g04020	cysteine biosynthetic process from serine;cellular amino acid biosyr
LOC_Os09g39670	na
LOC_Os09g39670	metabolic process
LOC_Os04g04020 LOC	na
LOC_Os09g39670	metabolic process
LOC_Os04g04020	translation;response to UV-B;developmental process;cellular respor
LOC_Os09g39670	metabolic process
LOC_Os04g04020	na
LOC_Os09g39670	metabolic process
LOC_Os09g39670	metabolic process
LOC_Os04g04020 LOC	na
LOC_Os09g39670	metabolic process
LOC_Os04g04020	nucleobase, nucleoside, nucleotide and nucleic acid metabolic proc
LOC_Os09g39670	metabolic process
LOC_Os04g04020 LOC	na
LOC_Os04g04020 LOC	na
LOC_Os09g39670	metabolic process
LOC_Os02g45250	transcription, DNA-dependent;regulation of transcription, DNA-dep
LOC_Os04g04020	na
LOC_Os09g39670	na
LOC_Os09g39670	na
LOC_Os04g04020 LOC	protein amino acid dephosphorylation;defense response to bacteriu
LOC_Os04g04020 LOC	na
LOC_Os04g04020 LOC	na
LOC_Os04g04020	transport;vesicle-mediated transport
LOC_Os09g23200 LOC	na
LOC_Os09g39670	biosynthetic process
LOC_Os03g57300 LOC	production of ta-siRNAs involved in RNA interference;primary micro
LOC_Os04g04020	intracellular protein transport;vesicle-mediated transport;endosom
LOC_Os04g04020	vesicle-mediated transport
LOC_Os04g04020	cysteine biosynthetic process from serine
LOC_Os04g04020 LOC	transport;ion transport;ATP hydrolysis coupled proton transport;pro
LOC_Os04g04020 LOC	transport;ion transport;ATP hydrolysis coupled proton transport
LOC_Os04g04020 LOC	transport;ion transport;ATP hydrolysis coupled proton transport
LOC_Os02g45250 LOC	DNA replication;DNA repair;DNA recombination;response to DNA d
LOC_Os04g04020	cysteine biosynthetic process from serine;cysteine homeostasis
LOC_Os04g04020	na
LOC_Os04g04020 LOC	na
LOC_Os04g04020	intracellular protein transport;Golgi to vacuole transport;vesicle-me

Do not distribute

LOC_Os04g04020	transport;intracellular protein transport;protein transport;vesicle-m
LOC_Os04g04020	na
LOC_Os04g04020	vesicle-mediated transport
LOC_Os09g39670	transport;purine nucleotide transport;transmembrane transport
LOC_Os04g04020 LOC	na
LOC_Os04g04020	cysteine biosynthetic process from serine
LOC_Os03g57300	cytokinesis;cell plate assembly
LOC_Os03g57300	transport;ER to Golgi vesicle-mediated transport
LOC_Os03g57300	transport;ER to Golgi vesicle-mediated transport
LOC_Os03g57300	fatty acid beta-oxidation;protein import into peroxisome matrix
LOC_Os04g04020 LOC	transport;ion transport;ATP hydrolysis coupled proton transport
LOC_Os04g04020	cysteine biosynthetic process from serine
LOC_Os04g04020	na
LOC_Os04g04020	chloride transport;cell volume homeostasis
LOC_Os09g23200 LOC	na
LOC_Os04g04020 LOC	tRNA aminoacylation for protein translation;methionyl-tRNA amino
LOC_Os06g36850	response to cold;defense response to bacterium;oxidation reductio
LOC_Os04g04020 LOC	na
LOC_Os03g57300 LOC	mRNA processing;regulation of translation;regulation of RNA splicin
LOC_Os03g57300	response to abscisic acid stimulus;pollen tube development
LOC_Os04g04020 LOC	transport
LOC_Os04g04020	na
LOC_Os04g04020	regulation of gene expression by genetic imprinting;negative regula
LOC_Os04g04020	transport
LOC_Os09g39670 LOC	cellular aldehyde metabolic process;metabolic process;oxidation re
LOC_Os04g04020 LOC	GTP catabolic process;translational elongation;response to cadmiur
LOC_Os04g04020 LOC	GTP catabolic process;translational elongation;response to cadmiur
LOC_Os04g04020 LOC	GTP catabolic process;translational elongation;response to cadmiur
LOC_Os04g04020 LOC	na
LOC_Os03g57300	gluconeogenesis;pyrimidine nucleotide biosynthetic process;chrom
LOC_Os09g39670	nitrogen compound metabolic process;response to bacterium;cellul
LOC_Os04g04020	transport;transmembrane transport
LOC_Os04g04020	transport;response to nematode;monosaccharide transport;transm
LOC_Os04g04020	vesicle docking during exocytosis;vesicle-mediated transport;protei
LOC_Os03g57300 LOC	nucleotide-excision repair;response to cold;proteasomal ubiquitin-c
LOC_Os03g57300	response to abscisic acid stimulus;pollen tube development
LOC_Os09g39670	glucose catabolic process;cellulose biosynthetic process;Golgi vesicl
LOC_Os04g04020 LOC	pollen tube growth;sexual reproduction
LOC_Os03g57300 LOC	nucleotide-excision repair;response to cold;proteasomal ubiquitin-c
LOC_Os03g57300 LOC	na
LOC_Os04g04020	carbohydrate metabolic process;galactose metabolic process;positi
LOC_Os04g04020	na
LOC_Os04g04020 LOC	toxin catabolic process
LOC_Os04g04020	na
LOC_Os04g04020	na
LOC_Os03g57300 LOC	regulation of transcription, DNA-dependent;response to abscisic aci

Do not distribute

LOC_Os04g04020	intracellular protein transport;vesicle-mediated transport
LOC_Os04g04020	transport
LOC_Os09g39670	transport;purine nucleotide transport;transmembrane transport
LOC_Os03g57300	na
LOC_Os03g57300	postreplication repair;sister chromatid cohesion
LOC_Os09g23200 LOC	defense response
LOC_Os03g57300 LOC	nucleotide-excision repair;proteasomal ubiquitin-dependent protei
LOC_Os02g45250	regulation of transcription, DNA-dependent
LOC_Os03g57300	na
LOC_Os04g04020	transport;vesicle-mediated transport
LOC_Os02g45250	response to gibberellin stimulus;regulation of transcription, DNA-de
LOC_Os03g57300	cytokinesis;cell plate assembly
LOC_Os02g45250	na
LOC_Os03g57300 LOC	lipid metabolic process;fatty acid metabolic process;fatty acid biosy
LOC_Os04g04020	intracellular protein transport
LOC_Os04g04020	transport;sterol biosynthetic process;sphingoid biosynthetic proces
LOC_Os04g04020	transport;response to nematode;monosaccharide transport;transm
LOC_Os09g39670	one-carbon metabolic process
LOC_Os03g57300	reproduction;microtubule cytoskeleton organization;mitotic cell cyc
LOC_Os04g04020 LOC	glucose catabolic process;protein targeting to vacuole;calcium ion t
LOC_Os09g23200 LOC	regulation of transcription, DNA-dependent
LOC_Os02g45250	na
LOC_Os02g45250	na
LOC_Os02g45250	nucleosome assembly
LOC_Os02g45250	nucleosome assembly
LOC_Os02g45250	na
LOC_Os09g39670	na
LOC_Os09g23200 LOC	oxidation reduction
LOC_Os04g04020	na
LOC_Os04g04020	response to salt stress
LOC_Os04g04020	response to salt stress
LOC_Os04g04020	ubiquitin-dependent protein catabolic process;response to red or fa
LOC_Os04g04020	vesicle-mediated transport;Golgi vesicle transport
LOC_Os04g04020	na
LOC_Os04g04020	defense response to bacterium;oxidation reduction
LOC_Os04g04020	vesicle docking during exocytosis;vesicle-mediated transport
LOC_Os04g04020	microtubule cytoskeleton organization;cytokinesis by cell plate form
LOC_Os09g23200 LOC	na
LOC_Os04g04020 LOC	tricarboxylic acid cycle;cellular carbohydrate metabolic process;resp
LOC_Os03g57300 LOC	protein amino acid glycosylation;proteolysis;lipid metabolic process
LOC_Os02g45250	na
LOC_Os02g45250	regulation of transcription, DNA-dependent;response to heat;transc
LOC_Os02g45250	na
LOC_Os02g45250	na
LOC_Os02g45250	nuclear-transcribed mRNA catabolic process;regulation of transcript
LOC_Os04g04020	regulation of signal transduction;response to cold

Do not distribute

LOC_Os04g04020	response to cold;regulation of signal transduction
LOC_Os06g36850	na
LOC_Os04g04020	na
LOC_Os04g04020	ubiquitin-dependent protein catabolic process;cell proliferation
LOC_Os04g04020	chloride transport;cell volume homeostasis
LOC_Os03g57300	autophagic vacuole assembly;transport;autophagy;protein transpor
LOC_Os04g04020	na
LOC_Os04g04020	na
LOC_Os09g23200 LOC	metabolic process
LOC_Os04g04020	na
LOC_Os09g39670	chloroplast organization;protein import into chloroplast stroma
LOC_Os09g39670	rRNA processing;tRNA metabolic process;chloroplast organization;c
LOC_Os09g39670	na
LOC_Os03g57300	transport;ER to Golgi vesicle-mediated transport
LOC_Os09g23200 LOC	na
LOC_Os04g04020 LOC	tricarboxylic acid cycle;cellular carbohydrate metabolic process;resp
LOC_Os04g04020	vesicle docking during exocytosis;vesicle-mediated transport
LOC_Os02g45250	regulation of transcription, DNA-dependent
LOC_Os02g45250	na
LOC_Os02g45250	na
LOC_Os02g45250	na
LOC_Os02g45250	transcription, DNA-dependent;regulation of transcription, DNA-dep
LOC_Os02g45250	regulation of transcription, DNA-dependent;transcription, DNA-dep
LOC_Os02g45250	regulation of transcription, DNA-dependent;transcription, DNA-dep
LOC_Os02g45250	nuclear-transcribed mRNA catabolic process;regulation of transcript
LOC_Os02g45250	na
LOC_Os02g45250	transcription, DNA-dependent;regulation of transcription, DNA-dep
LOC_Os02g45250	na
LOC_Os02g45250	regulation of transcription, DNA-dependent;transcription, DNA-dep
LOC_Os02g45250	trichome branching;regulation of transcription, DNA-dependent;tra
LOC_Os09g39670	nitrogen compound metabolic process;response to bacterium;cellul
LOC_Os02g45250	regulation of transcription, DNA-dependent
LOC_Os03g57300	fatty acid biosynthetic process;metabolic process;lipid biosynthetic
LOC_Os03g57300 LOC	ER to Golgi vesicle-mediated transport;metabolic process
LOC_Os04g04020	actin filament depolymerization
LOC_Os04g04020	protein modification process;response to salt stress;aging;seed gerr
LOC_Os04g04020	actin filament depolymerization
LOC_Os04g04020	transport;response to nematode;monosaccharide transport;transm
LOC_Os04g04020	transport;response to nematode;monosaccharide transport;transm
LOC_Os04g04020	rRNA processing
LOC_Os04g04020	na
LOC_Os04g04020	protein amino acid phosphorylation;response to auxin stimulus;aux
LOC_Os04g04020	transport;response to nematode;monosaccharide transport;transm
LOC_Os03g57300	transport;autophagy;leaf senescence;response to starvation;protein
LOC_Os02g45250	mucilage biosynthetic process during seed coat development;seed (
LOC_Os02g45250	transcription, DNA-dependent;regulation of transcription, DNA-dep

Do not distribute

LOC_Os02g45250	transcription, DNA-dependent;regulation of transcription, DNA-dep
LOC_Os02g45250	transcription, DNA-dependent;regulation of transcription, DNA-dep
LOC_Os02g45250	na
LOC_Os02g45250	cell morphogenesis;regulation of transcription, DNA-dependent;cell
LOC_Os02g45250	regulation of transcription, DNA-dependent
LOC_Os02g45250	na
LOC_Os02g45250	na
LOC_Os02g45250	transcription, DNA-dependent;regulation of transcription, DNA-dep
LOC_Os02g45250	na
LOC_Os02g45250	regulation of transcription, DNA-dependent
LOC_Os04g04020	protein amino acid glycosylation
LOC_Os04g04020	na
LOC_Os03g57300	transport;autophagy;protein transport
LOC_Os04g04020	transport
LOC_Os04g04020	UV protection;negative regulation of flower development;leaf morp
LOC_Os06g36850 LOC	protein folding;cell death;response to cold;systemic acquired resista
LOC_Os06g36850 LOC	protein folding;chloroplast organization;embryonic development;pr
LOC_Os06g36850 LOC	protein folding;response to heat;protein refolding;cellular protein m
LOC_Os06g36850 LOC	protein folding;pyrimidine ribonucleotide biosynthetic process;resp
LOC_Os06g36850 LOC	protein folding;response to heat;protein refolding;cellular protein m
LOC_Os06g36850 LOC	protein folding;protein refolding;cellular protein metabolic process;
LOC_Os06g36850 LOC	cell death;response to cold;systemic acquired resistance;cellular pro
LOC_Os06g36850 LOC	cellular protein metabolic process;protein folding;chloroplast organ
LOC_Os04g04020 LOC	GDP-mannose transport
LOC_Os04g04020	ubiquitin-dependent protein catabolic process;UV protection;negat
LOC_Os04g04020	transport
LOC_Os02g45250	regulation of transcription, DNA-dependent;response to heat;transc
LOC_Os02g45250	positive regulation of transcription, DNA-dependent
LOC_Os02g45250	transcription, DNA-dependent;regulation of transcription, DNA-dep
LOC_Os02g45250	transcription, DNA-dependent;regulation of transcription, DNA-dep
LOC_Os02g45250	regulation of transcription, DNA-dependent;regulation of meristem
LOC_Os04g04020	UV protection;negative regulation of flower development;leaf morp
LOC_Os06g36850	metabolic process;selenium metabolic process
LOC_Os03g57300 LOC	protein amino acid glycosylation
LOC_Os03g57300	na
LOC_Os04g04020	defense response to bacterium;oxidation reduction
LOC_Os04g04020	transport;response to nematode;monosaccharide transport;transm
LOC_Os04g04020	vesicle-mediated transport;Golgi vesicle transport
LOC_Os04g04020	activation of protein kinase C activity by G-protein coupled receptor
LOC_Os04g04020	response to salt stress;response to temperature stimulus;response
LOC_Os09g39670	biosynthetic process
LOC_Os03g57300 LOC	na
LOC_Os02g45250	na
LOC_Os02g45250	na
LOC_Os02g45250	transcription, DNA-dependent;regulation of transcription, DNA-dep
LOC_Os02g45250	na

Do not distribute

LOC_Os02g45250	na
LOC_Os02g45250	na
LOC_Os02g45250	brassinosteroid mediated signaling pathway;negative regulation of
LOC_Os02g45250	na
LOC_Os02g45250	transcription, DNA-dependent;regulation of transcription, DNA-dep
LOC_Os02g45250	na
LOC_Os02g45250	transcription, DNA-dependent;regulation of transcription, DNA-dep
LOC_Os02g45250	transcription, DNA-dependent;regulation of transcription, DNA-dep
LOC_Os02g45250	regulation of transcription, DNA-dependent
LOC_Os02g45250	na
LOC_Os04g04020	actin filament depolymerization;regulation of transcription, DNA-de
LOC_Os02g45250 LOC	glucose catabolic process;protein amino acid N-linked glycosylation,
LOC_Os04g04020	actin filament depolymerization
LOC_Os04g04020	na
LOC_Os04g04020	ATP catabolic process;pollen germination
LOC_Os09g39670	na
LOC_Os09g39670	translation
LOC_Os04g04020	transport;response to nematode;monosaccharide transport;transm
LOC_Os06g36850	protein folding
LOC_Os06g36850	protein folding;response to stress;response to salt stress;response t
LOC_Os04g04020	rRNA processing
LOC_Os02g45250	regulation of transcription, DNA-dependent
LOC_Os02g45250	regulation of transcription, DNA-dependent
LOC_Os09g39670	translation
LOC_Os09g39670	translation
LOC_Os06g36850	methionine biosynthetic process;metabolic process
LOC_Os04g04020	transport
LOC_Os04g04020	small GTPase mediated signal transduction;intracellular protein trar
LOC_Os09g39670	translation
LOC_Os04g04020	ATP catabolic process;pollen germination
LOC_Os04g04020	transport;transmembrane transport;monosaccharide transport;carl
LOC_Os04g04020	transport;carbohydrate transport;carbohydrate transmembrane tra
LOC_Os04g04020 LOC	carbohydrate metabolic process;tricarboxylic acid cycle;malate met
LOC_Os02g45250	regulation of transcription, DNA-dependent
LOC_Os02g45250	transcription, DNA-dependent;regulation of transcription, DNA-dep
LOC_Os02g45250	transcription, DNA-dependent;regulation of transcription, DNA-dep
LOC_Os02g45250	multicellular organismal development;negative regulation of cell pr
LOC_Os04g04020	nuclear mRNA splicing, via spliceosome;nuclear-transcribed mRNA c
LOC_Os04g04020	transport;transmembrane transport
LOC_Os09g39670	phosphatidylcholine biosynthetic process;biosynthetic process
LOC_Os04g04020	proteolysis;ubiquitin-dependent protein catabolic process;embryon
LOC_Os03g57300	nuclear-transcribed mRNA catabolic process;fatty acid beta-oxidatic
LOC_Os04g04020	transport;transmembrane transport
LOC_Os04g04020 LOC	G2 phase of mitotic cell cycle;microtubule cytoskeleton organizatior
LOC_Os02g45250	na
LOC_Os02g45250	na

Do not distribute

LOC_Os04g04020	na
LOC_Os04g04020	transport;transmembrane transport
LOC_Os04g04020	actin filament depolymerization
LOC_Os06g36850	na
LOC_Os03g57300	na
LOC_Os04g04020	transport;transmembrane transport
LOC_Os02g45250	transcription, DNA-dependent;regulation of transcription, DNA-dep
LOC_Os06g36850	oxidation reduction;methylation
LOC_Os04g04020	na
LOC_Os04g04020	translational initiation
LOC_Os04g04020	na
LOC_Os04g04020	protein modification process
LOC_Os04g04020	na
LOC_Os03g57300	cellular process;vegetative to reproductive phase transition of meri
LOC_Os02g45250	regulation of transcription, DNA-dependent
LOC_Os02g45250	transcription, DNA-dependent;regulation of transcription, DNA-dep
LOC_Os02g45250	na
LOC_Os04g04020	intracellular protein transport;vesicle-mediated transport;protein ta
LOC_Os04g04020	protein targeting to vacuole;intracellular protein transport;vesicle-r
LOC_Os04g04020	transport;transmembrane transport
LOC_Os04g04020	transport;transmembrane transport;monosaccharide transport
LOC_Os03g57300	phosphorylation;phosphoinositide phosphorylation;phosphoinositic
LOC_Os04g04020	na
LOC_Os04g04020	phytosphingosine metabolic process;activation of protein kinase C a
LOC_Os04g04020	actin filament depolymerization;actin filament polymerization
LOC_Os04g04020	na
LOC_Os04g04020	transport;transmembrane transport
LOC_Os04g04020	na
LOC_Os02g45250	transcription, DNA-dependent;regulation of transcription, DNA-dep
LOC_Os02g45250	regulation of transcription, DNA-dependent
LOC_Os02g45250	na
LOC_Os04g04020	na
LOC_Os03g57300	protein amino acid glycosylation
LOC_Os04g04020 LOC	carbohydrate metabolic process;tricarboxylic acid cycle;malate met
LOC_Os09g39670	vernalization response
LOC_Os04g04020	na
LOC_Os04g04020	na
LOC_Os03g57300	na
LOC_Os04g04020	ubiquitin-dependent protein catabolic process;glucuronoxylan meta
LOC_Os04g04020	actin filament depolymerization
LOC_Os03g57300	glycerol ether metabolic process;response to oxidative stress;respo
LOC_Os06g36850	cysteine biosynthetic process from serine;cellular amino acid biosyr
LOC_Os04g04020	actin filament depolymerization;response to stress
LOC_Os03g57300	na
LOC_Os02g45250	regulation of transcription, DNA-dependent
LOC_Os02g45250	na

Do not distribute

LOC_Os06g36850	metabolic process
LOC_Os06g36850	ATP hydrolysis coupled proton transport;response to cold;plant-typ
LOC_Os06g36850	gluconeogenesis;GTP catabolic process;cytoskeleton organization;m
LOC_Os04g04020	na
LOC_Os03g57300	purine nucleotide biosynthetic process;mRNA export from nucleus;
LOC_Os04g04020	ATP catabolic process;transport;anion transport;intra-Golgi vesicle-
LOC_Os04g04020	response to cadmium ion;oxidation reduction
LOC_Os04g04020	vesicle-mediated transport
LOC_Os02g45250	na
LOC_Os04g04020	na
LOC_Os02g45250	na
LOC_Os04g04020	na
LOC_Os04g04020	regulation of transcription, DNA-dependent;histone acetylation;pro
LOC_Os04g04020	regulation of gene expression by genetic imprinting;negative regula
LOC_Os03g57300	protein sumoylation
LOC_Os04g04020	na
LOC_Os06g36850	pentose-phosphate shunt;ER to Golgi vesicle-mediated transport;re
LOC_Os06g36850	pentose-phosphate shunt;response to salt stress;response to sucros
LOC_Os09g39670	metabolic process;jasmonic acid biosynthetic process
LOC_Os02g45250	transcription, DNA-dependent;regulation of transcription, DNA-dep
LOC_Os04g04020	protein amino acid phosphorylation
LOC_Os04g04020	very-long-chain fatty acid metabolic process;fatty acid biosynthetic
LOC_Os02g45250	na
LOC_Os04g04020	na
LOC_Os04g04020	DNA replication;DNA repair;DNA recombination;response to DNA d
LOC_Os04g04020	regulation of Rab GTPase activity;positive regulation of Rab GTPase
LOC_Os09g39670	L-phenylalanine catabolic process;tyrosine metabolic process;tyrosi
LOC_Os04g04020	small GTPase mediated signal transduction
LOC_Os02g45250	regulation of transcription, DNA-dependent
LOC_Os04g04020	megagametogenesis;regulation of ARF protein signal transduction;v
LOC_Os04g04020	virus induced gene silencing;response to auxin stimulus;embryonic
LOC_Os12g36430	na
LOC_Os04g04020	cellular metabolic process
LOC_Os03g57300	response to DNA damage stimulus;reciprocal meiotic recombinatio
LOC_Os12g36430	na
LOC_Os02g45250	na
LOC_Os04g04020	double-strand break repair via homologous recombination;DNA rep
LOC_Os04g04020	folic acid and derivative biosynthetic process;oxidation reduction
LOC_Os06g36850	cysteine metabolic process;metabolic process;iron-sulfur cluster ass
LOC_Os04g04020	ubiquitin-dependent protein catabolic process;response to red or fa
LOC_Os03g57300	transcription, DNA-dependent;regulation of transcription, DNA-dep
LOC_Os06g36850	translation;tRNA aminoacylation for protein translation;glutamyl-tR
LOC_Os02g45250	na
LOC_Os02g45250	na
LOC_Os02g45250	maltose metabolic process;photosynthetic electron transport chain
LOC_Os03g57300	nuclear-transcribed mRNA catabolic process, nonsense-mediated d

Do not distribute

LOC_Os02g45250	na
LOC_Os02g45250	lipid metabolic process
LOC_Os09g39670	leaf morphogenesis
LOC_Os02g45250	regulation of transcription, DNA-dependent
LOC_Os02g45250	microtubule cytoskeleton organization;cytokinesis by cell plate forn
LOC_Os02g45250	na
LOC_Os02g45250	response to salicylic acid stimulus;response to jasmonic acid stimulu
LOC_Os02g45250	actin filament bundle assembly
LOC_Os04g04020	protein geranylgeranylation
LOC_Os12g36430	na
LOC_Os12g36430	na
LOC_Os02g45250	na
LOC_Os02g45250	metabolic process
LOC_Os06g36850	translation;tRNA aminoacylation for protein translation;tRNA aminc
LOC_Os04g04020	na
LOC_Os03g57300	glycerol ether metabolic process;response to oxidative stress;respo
LOC_Os02g45250	transcription, DNA-dependent;regulation of transcription, DNA-dep
LOC_Os09g39670	cellular aldehyde metabolic process;metabolic process;response to
LOC_Os03g57300	calcium ion transport;cellular zinc ion homeostasis;response to nem
LOC_Os02g45250	regulation of transcription, DNA-dependent
LOC_Os02g45250	regulation of transcription, DNA-dependent;response to heat;transc
LOC_Os02g45250	response to stress;response to water deprivation;response to salt st
LOC_Os12g36430	regulation of transcription, DNA-dependent;pollen development;an
LOC_Os04g04020	gluconeogenesis;cytoskeleton organization;response to heat;heat a
LOC_Os02g45250	transcription, DNA-dependent;regulation of transcription, DNA-dep
LOC_Os04g04020	na
LOC_Os02g45250	na
LOC_Os03g57300	na
LOC_Os02g45250	photosynthesis
LOC_Os03g57300	na
LOC_Os12g36430	glycine catabolic process;glycine decarboxylation via glycine cleavag
LOC_Os04g04020	protein transport
LOC_Os03g57300	formation of translation preinitiation complex;translation;translatio
LOC_Os02g45250	na
LOC_Os03g57300	proteolysis
LOC_Os09g39670	protein peptidyl-prolyl isomerization;protein folding
LOC_Os04g04020	response to salt stress
LOC_Os02g45250	regulation of transcription, DNA-dependent
LOC_Os04g04020	protein transport
LOC_Os04g04020	protein transport
LOC_Os04g04020	circumnutation;negative gravitropism;detection of gravity
LOC_Os04g04020	protein amino acid dephosphorylation;response to abscisic acid stin
LOC_Os04g04020	transport
LOC_Os04g04020	protein targeting to membrane;epidermal cell fate specification;pos
LOC_Os09g39670	protein amino acid phosphorylation
LOC_Os02g45250	transcription, DNA-dependent;regulation of transcription, DNA-dep

Do not distribute

LOC_Os02g45250	na
LOC_Os04g04020	na
LOC_Os04g04020	na
LOC_Os02g45250	metabolic process;oxidation reduction
LOC_Os02g45250	regulation of transcription, DNA-dependent
LOC_Os02g45250	regulation of transcription, DNA-dependent
LOC_Os12g36430	folic acid and derivative metabolic process
LOC_Os02g45250	na
LOC_Os03g57300	protein amino acid N-linked glycosylation;glucuronoxylan metabolic
LOC_Os04g04020	negative regulation of transcription factor activity;protein sumoylat
LOC_Os02g45250	na
LOC_Os04g04020	ubiquitin-dependent protein catabolic process;response to red or fa
LOC_Os02g45250	regulation of transcription, DNA-dependent
LOC_Os02g45250	transcription, DNA-dependent;regulation of transcription, DNA-dep
LOC_Os02g45250	transcription, DNA-dependent;regulation of transcription, DNA-dep
LOC_Os09g39670	intra-Golgi vesicle-mediated transport
LOC_Os04g04020	na
LOC_Os03g57300	na
LOC_Os02g45250	na
LOC_Os02g45250	na
LOC_Os04g04020	small GTPase mediated signal transduction
LOC_Os04g04020	protein modification process;protein transport;trichome branching;
LOC_Os06g36850	DNA replication;DNA repair;proteolysis;DNA duplex unwinding
LOC_Os04g04020	very-long-chain fatty acid metabolic process;fatty acid biosynthetic
LOC_Os02g45250	na
LOC_Os03g57300	carbohydrate metabolic process;tricarboxylic acid cycle;malate met
LOC_Os04g04020	translational initiation
LOC_Os02g45250	na
LOC_Os02g45250	na
LOC_Os04g04020	protein targeting;intracellular protein transport;protein transport
LOC_Os02g45250	na
LOC_Os02g45250	na
LOC_Os04g04020	metabolic process;cell-cell signaling;virus induced gene silencing;pr
LOC_Os04g04020	transport
LOC_Os06g36850	cellular amino acid metabolic process;response to salt stress;respor
LOC_Os06g36850	cellular amino acid metabolic process;nitrogen compound metaboli
LOC_Os06g36850	cysteine biosynthetic process from serine;cellular amino acid biosyr
LOC_Os04g04020	metabolic process
LOC_Os03g57300	lipid metabolic process;sphingolipid metabolic process;biosynthetic
LOC_Os04g04020	MAPKKK cascade;protein amino acid phosphorylation;phosphorylat
LOC_Os02g45250	na
LOC_Os02g45250	na
LOC_Os03g57300	autophagy
LOC_Os03g57300	RNA processing
LOC_Os09g39670	cellular aldehyde metabolic process;metabolic process;response to
LOC_Os04g04020	na

Do not distribute

LOC_Os04g04020	protein targeting;intracellular protein transport;protein transport;tr
LOC_Os04g04020	na
LOC_Os03g57300	na
LOC_Os02g45250	na
LOC_Os04g04020	na
LOC_Os06g36850	na
LOC_Os09g39670	purine nucleotide biosynthetic process;'de novo' IMP biosynthetic p
LOC_Os04g04020	protein amino acid phosphorylation
LOC_Os04g04020	phosphorylation;protein amino acid phosphorylation
LOC_Os03g57300	transcription, DNA-dependent;brassinosteroid mediated signaling p
LOC_Os04g04020	G2 phase of mitotic cell cycle;regulation of transcription, DNA-depe
LOC_Os12g36430	type I hypersensitivity
LOC_Os02g45250	transcription, DNA-dependent;regulation of transcription, DNA-dep
LOC_Os02g45250	regulation of transcription, DNA-dependent
LOC_Os04g04020	na
LOC_Os02g45250	regulation of transcription, DNA-dependent
LOC_Os12g36430	transport;phosphate transport;transmembrane transport
LOC_Os03g57300	cell communication
LOC_Os03g57300	cell redox homeostasis
LOC_Os04g04020	virus induced gene silencing;response to auxin stimulus;embryonic
LOC_Os06g36850	cellular amino acid metabolic process;response to salt stress;respor
LOC_Os06g36850	intracellular protein transport
LOC_Os03g57300	exocytosis
LOC_Os04g04020	regulation of translation;regulation of RNA splicing
LOC_Os02g45250	transcription, DNA-dependent;regulation of transcription, DNA-dep
LOC_Os09g39670	one-carbon metabolic process
LOC_Os09g39670	one-carbon metabolic process
LOC_Os09g39670	one-carbon metabolic process
LOC_Os03g57300	na
LOC_Os04g04020	phosphate metabolic process;defense response to bacterium;respo
LOC_Os04g04020	protein amino acid dephosphorylation;protein amino acid N-linked
LOC_Os02g45250	transcription, DNA-dependent;regulation of transcription, DNA-dep
LOC_Os12g36430	na
LOC_Os03g57300	nuclear-transcribed mRNA catabolic process;embryonic developme
LOC_Os04g04020	regulation of transcription, DNA-dependent
LOC_Os04g04020	intra-Golgi vesicle-mediated transport;small GTPase mediated signa
LOC_Os04g04020	small GTPase mediated signal transduction;protein transport
LOC_Os04g04020	DNA replication;DNA repair;DNA recombination;response to DNA d
LOC_Os09g39670	one-carbon metabolic process
LOC_Os02g45250	transcription, DNA-dependent;regulation of transcription, DNA-dep
LOC_Os04g04020	protein amino acid phosphorylation;multicellular organismal develc
LOC_Os02g45250	na
LOC_Os04g04020	intracellular protein transport;vesicle-mediated transport
LOC_Os02g45250	transcription, DNA-dependent;regulation of transcription, DNA-dep
LOC_Os12g36430	na
LOC_Os04g04020	na

Do not distribute

LOC_Os04g04020	MAPKKK cascade;protein amino acid phosphorylation;phosphorylat
LOC_Os04g04020	DNA replication;DNA repair;DNA recombination;response to DNA d
LOC_Os09g39670	ATP hydrolysis coupled proton transport;transport;ion transport;pr
LOC_Os09g39670	protein modification process;transcription factor import into nucleu
LOC_Os02g45250	transcription, DNA-dependent;regulation of transcription, DNA-dep
LOC_Os09g39670	metabolic process;starch biosynthetic process;oxidation reduction
LOC_Os03g57300	na
LOC_Os09g39670	oxidation reduction
LOC_Os09g39670	oxidation reduction
LOC_Os09g39670	oxidation reduction
LOC_Os09g39670	oxidation reduction
LOC_Os09g39670	oxidation reduction
LOC_Os04g04020	intracellular protein transport;vesicle-mediated transport;transport
LOC_Os04g04020	protein transport;transport
LOC_Os12g36430	na
LOC_Os03g57300	protein peptidyl-prolyl isomerization;protein folding
LOC_Os04g04020	na
LOC_Os04g04020	protein amino acid dephosphorylation;defense response to bacteriu
LOC_Os04g04020	response to salt stress
LOC_Os04g04020	response to bacterium;phloem or xylem histogenesis;cell-cell adhes
LOC_Os04g04020	na
LOC_Os04g04020	lipid metabolic process;signal transduction
LOC_Os04g04020	lipid metabolic process;signal transduction
LOC_Os03g57300	oxidation reduction
LOC_Os03g57300	na
LOC_Os04g04020	na
LOC_Os02g45250	regulation of transcription, DNA-dependent
LOC_Os06g36850	response to oxidative stress;folic acid and derivative biosynthetic pr
LOC_Os04g04020	na
LOC_Os04g04020	amino acid transmembrane transport;fatty acid beta-oxidation;tran
LOC_Os03g57300	na
LOC_Os03g57300	cation transport;calcium ion transmembrane transport;response to
LOC_Os12g36430	na
LOC_Os12g36430	shade avoidance;indoleacetic acid biosynthetic process;response to
LOC_Os12g36430	transcription, DNA-dependent;regulation of transcription, DNA-dep
LOC_Os09g39670	mitotic cell cycle;cytokinesis;protein amino acid phosphorylation;ce
LOC_Os04g04020	protein amino acid phosphorylation;phosphorylation;protein amino
LOC_Os12g36430	na
LOC_Os03g57300	na
LOC_Os04g04020	retrograde vesicle-mediated transport, Golgi to ER;maintenance of
LOC_Os04g04020	cellular protein localization;chloroplast avoidance movement;protei
LOC_Os04g04020	response to salt stress
LOC_Os12g36430	anion transport;cellular response to boron levels;boron transport
LOC_Os09g39670	cellular aldehyde metabolic process;metabolic process;oxidation re
LOC_Os03g57300	na
LOC_Os03g57300	protein peptidyl-prolyl isomerization;protein folding

Do not distribute

LOC_Os02g45250	na
LOC_Os03g57300	cation transport;response to osmotic stress;response to nematode;
LOC_Os02g45250	transcription, DNA-dependent;regulation of transcription, DNA-dep
LOC_Os03g57300	intracellular protein transport
LOC_Os12g36430	na
LOC_Os06g36850	cysteine biosynthetic process from serine;cellular amino acid biosyr
LOC_Os04g04020	intracellular protein transport
LOC_Os04g04020	protein amino acid dephosphorylation
LOC_Os04g04020	RNA metabolic process
LOC_Os04g04020	protein amino acid phosphorylation;response to brassinosteroid sti
LOC_Os04g04020	transport;cation transport;transmembrane transport
LOC_Os03g57300	protein targeting to membrane;cation transport;anion transport;nu
LOC_Os03g57300	vacuolar transport;response to salt stress
LOC_Os04g04020	protein amino acid phosphorylation
LOC_Os09g39670	one-carbon metabolic process
LOC_Os09g39670	cellular aldehyde metabolic process;metabolic process;oxidation re
LOC_Os03g57300	pseudouridine synthesis;RNA modification
LOC_Os04g04020	response to ethylene stimulus;positive regulation of abscisic acid m
LOC_Os02g45250	transcription, DNA-dependent;regulation of transcription, DNA-dep
LOC_Os03g57300	phosphorylation
LOC_Os03g57300	transport;ion transport;cation transport;calcium ion transport;calciu
LOC_Os09g39670	transport;seed development;root development;adenine nucleotide
LOC_Os12g36430	na
LOC_Os04g04020	protein amino acid dephosphorylation;defense response to bacteriu
LOC_Os03g57300	mRNA modification
LOC_Os04g04020	auxin biosynthetic process;positive regulation of flower developme
LOC_Os04g04020	na
LOC_Os12g36430	xylem and phloem pattern formation;steroid metabolic process;res
LOC_Os03g57300	intra-Golgi vesicle-mediated transport
LOC_Os04g04020	response to salicylic acid stimulus
LOC_Os04g04020	response to UV-B
LOC_Os04g04020	na
LOC_Os09g39670	metabolic process
LOC_Os04g04020	phenylalanyl-tRNA aminoacylation;tRNA aminoacylation
LOC_Os02g45250	na
LOC_Os02g45250	regulation of transcription, DNA-dependent
LOC_Os04g04020	na
LOC_Os12g36430	na
LOC_Os04g04020	na
LOC_Os04g04020	metabolic process;oxidation reduction;pollen tube growth
LOC_Os04g04020	metabolic process;oxidation reduction
LOC_Os04g04020	microtubule-based movement
LOC_Os06g36850	cellular amino acid metabolic process;one-carbon metabolic proces
LOC_Os02g45250	regulation of transcription, DNA-dependent
LOC_Os12g36430	na
LOC_Os04g04020	RNA processing

Do not distribute

LOC_Os04g04020	protein amino acid phosphorylation;phosphorylation;protein amino
LOC_Os03g57300	na
LOC_Os03g57300	protein amino acid phosphorylation;methylation-dependent chrom
LOC_Os04g04020	nuclear-transcribed mRNA catabolic process;protein amino acid gly
LOC_Os04g04020	translation;tRNA aminoacylation for protein translation;aspartyl-tR
LOC_Os04g04020	na
LOC_Os09g39670	protein amino acid phosphorylation;MAPKKK cascade;activation of
LOC_Os04g04020	mRNA processing;protein amino acid phosphorylation;response to
LOC_Os04g04020	protein amino acid phosphorylation;protein amino acid autophosph
LOC_Os12g36430	carbohydrate metabolic process;response to abscisic acid stimulus;
LOC_Os03g57300	regulation of ARF GTPase activity;GTP catabolic process;nucleocyto
LOC_Os04g04020	protein amino acid phosphorylation;phosphorylation
LOC_Os04g04020	nucleosome assembly;response to water deprivation
LOC_Os04g04020	nucleosome assembly;response to water deprivation
LOC_Os04g04020	nucleosome assembly;response to water deprivation
LOC_Os04g04020	nucleosome assembly;response to water deprivation
LOC_Os04g04020	nucleosome assembly;response to water deprivation
LOC_Os04g04020	nucleosome assembly;response to water deprivation
LOC_Os04g04020	nucleosome assembly;response to water deprivation
LOC_Os04g04020	nucleosome assembly;response to water deprivation
LOC_Os04g04020	nucleosome assembly;response to water deprivation
LOC_Os04g04020	nucleosome assembly;response to water deprivation
LOC_Os04g04020	na
LOC_Os12g36430	response to oxidative stress;oxidation reduction
LOC_Os04g04020	na
LOC_Os04g04020	lipid metabolic process;signal transduction;response to cold;respon
LOC_Os04g04020	protein amino acid phosphorylation;phosphorylation
LOC_Os09g39670	na
LOC_Os04g04020	tRNA aminoacylation for protein translation;prolyl-tRNA aminoacyl
LOC_Os03g57300	DNA repair;chromatin remodeling;regulation of transcription, DNA-
LOC_Os04g04020	carbohydrate metabolic process;galactose metabolic process;respo
LOC_Os04g04020	regulation of transcription, DNA-dependent;transport;ion transport
LOC_Os04g04020	oxidation reduction;N-terminal protein myristoylation;ER to Golgi v
LOC_Os04g04020	na
LOC_Os04g04020	tRNA aminoacylation for protein translation;glycyl-tRNA aminoacyl
LOC_Os09g39670	cell redox homeostasis;oxidation reduction;regulation of pollen tub
LOC_Os04g04020	coenzyme A biosynthetic process
LOC_Os04g04020	regulation of Rab GTPase activity;positive regulation of Rab GTPase
LOC_Os03g57300	na
LOC_Os03g57300	negative regulation of transcription from RNA polymerase II promot
LOC_Os03g57300	na
LOC_Os04g04020	proteolysis;ubiquitin-dependent protein catabolic process;proteoly
LOC_Os03g57300	polar nucleus fusion;pollen tube development
LOC_Os04g04020	dephosphorylation;protein amino acid dephosphorylation
LOC_Os04g04020	ER-associated protein catabolic process;hyperosmotic salinity respo
LOC_Os04g04020	na

Do not distribute

LOC_Os04g04020	na
LOC_Os02g45250	na
LOC_Os04g04020	vesicle docking during exocytosis;vesicle-mediated transport;transp
LOC_Os04g04020	cation transport;stem cell fate determination;cellular metal ion hon
LOC_Os04g04020	na
LOC_Os03g57300	cation transport;calcium ion transmembrane transport;transport;io
LOC_Os06g36850	regulation of transcription, DNA-dependent
LOC_Os04g04020	protein amino acid phosphorylation;phosphorylation;protein aminc
LOC_Os06g36850	DNA replication;regulation of DNA replication;error-prone translesi
LOC_Os09g23200	na
LOC_Os06g36850	nuclear-transcribed mRNA catabolic process, nonsense-mediated de
LOC_Os04g04020	mitochondrial fission;mitochondrion organization;peroxisome fissio
LOC_Os06g36850	translation;tRNA aminoacylation for protein translation;glutamyl-tR
LOC_Os06g36850	small GTPase mediated signal transduction
LOC_Os04g04020	transport;ion transport;ATP hydrolysis coupled proton transport
LOC_Os04g04020	carbohydrate metabolic process;galactose metabolic process;respo
LOC_Os06g36850	response to stress;response to heat;response to cadmium ion
LOC_Os06g36850	ubiquitin-dependent protein catabolic process
LOC_Os06g36850	DNA replication;deoxyribonucleoside triphosphate biosynthetic pro
LOC_Os04g04020	defense response to oomycetes;carbohydrate metabolic process;pe
LOC_Os03g57300	na
LOC_Os09g39670	metabolic process
LOC_Os06g36850	DNA replication;deoxyribonucleoside triphosphate biosynthetic pro
LOC_Os06g36850	response to stress;response to heat;response to cadmium ion
LOC_Os04g04020	na
LOC_Os04g04020	vesicle-mediated transport;methylation
LOC_Os06g36850	glycolysis;trichome morphogenesis
LOC_Os03g57300	na
LOC_Os12g36430	proteolysis
LOC_Os04g04020	proteolysis;sterol biosynthetic process;sphingoid biosynthetic proce
LOC_Os03g57300	oxidation reduction
LOC_Os04g04020	na
LOC_Os04g04020	na
LOC_Os04g04020	proteolysis;ubiquitin-dependent protein catabolic process;proteolys
LOC_Os02g45250	tRNA processing;tRNA 3'-trailer cleavage;tRNA 3'-end processing
LOC_Os04g04020	na
LOC_Os03g57300	COPII vesicle coating
LOC_Os04g04020	protein amino acid phosphorylation;phosphorylation
LOC_Os04g04020	transcription, DNA-dependent;regulation of transcription, DNA-dep
LOC_Os04g04020	na
LOC_Os04g04020	protein amino acid phosphorylation
LOC_Os04g04020	G2 phase of mitotic cell cycle;mitotic cell cycle;nuclear-transcribed r
LOC_Os12g36430	exocytosis
LOC_Os12g36430	na
LOC_Os09g39670	proteolysis
LOC_Os09g39670	proteolysis

Do not distribute

LOC_Os12g36430	oxidation reduction
LOC_Os03g57300	na
LOC_Os03g57300	glycerol ether metabolic process;plastid organization;hydrogen perc
LOC_Os03g57300	mitotic cell cycle;nuclear-transcribed mRNA catabolic process;RNA p
LOC_Os03g57300	protein amino acid glycosylation;cell adhesion;organ morphogenesi
LOC_Os09g39670	metabolic process
LOC_Os03g57300	na
LOC_Os03g57300	regulation of transcription, DNA-dependent;flower development;lea
LOC_Os04g04020	na
LOC_Os09g39670	translation
LOC_Os09g39670	translation
LOC_Os09g39670	translation
LOC_Os12g36430	na
LOC_Os04g04020	virus induced gene silencing;response to auxin stimulus;embryonic
LOC_Os04g04020	metabolic process
LOC_Os04g04020	ribosome biogenesis;translational elongation;response to cold;resp
LOC_Os02g45250	symbiosis, encompassing mutualism through parasitism;response to
LOC_Os12g36430	pollen development
LOC_Os04g04020	mRNA processing;protein amino acid phosphorylation;response to
LOC_Os06g36850	proteolysis
LOC_Os04g04020	virus induced gene silencing;response to auxin stimulus;embryonic
LOC_Os04g04020	ribosome biogenesis;translational elongation;response to cold;resp
LOC_Os04g04020	na
LOC_Os06g36850	metabolic process;para-aminobenzoic acid biosynthetic process;bio
LOC_Os06g36850	proteolysis
LOC_Os12g36430	na
LOC_Os04g04020	mitotic cell cycle;nuclear-transcribed mRNA catabolic process;meth
LOC_Os03g57300	na
LOC_Os04g04020	translational elongation;response to cold;response to salt stress;res
LOC_Os03g57300	na
LOC_Os04g04020	proteolysis
LOC_Os12g36430	protein amino acid phosphorylation;phosphorylation
LOC_Os02g45250	lipid metabolic process;fatty acid biosynthetic process;oxidation red
LOC_Os03g57300	respiratory burst during defense response;glutamine biosynthetic p
LOC_Os04g04020	GTP catabolic process;SRP-dependent cotranslational protein target
LOC_Os03g57300	translation
LOC_Os03g57300	protein amino acid phosphorylation;phosphorylation;protein amino
LOC_Os12g36430	na
LOC_Os06g36850	metabolic process
LOC_Os03g57300	na
LOC_Os09g39670	na
LOC_Os04g04020	na
LOC_Os09g39670	na
LOC_Os04g04020	protein amino acid glycosylation;signal transduction
LOC_Os06g36850	transcription, DNA-dependent;regulation of transcription, DNA-dep
LOC_Os09g39670	one-carbon metabolic process

Do not distribute

LOC_Os02g45250	protein amino acid phosphorylation
LOC_Os02g45250	na
LOC_Os02g45250	phosphorylation
LOC_Os02g45250	protein amino acid phosphorylation;phosphorylation
LOC_Os02g45250	na
LOC_Os02g45250	protein amino acid phosphorylation
LOC_Os02g45250	na
LOC_Os09g39670	oxidation reduction
LOC_Os09g39670	na
LOC_Os12g36430	na
LOC_Os04g04020	protein amino acid dephosphorylation
LOC_Os03g57300	oxidation reduction
LOC_Os03g57300	protein peptidyl-prolyl isomerization;peptidyl-proline modification;(
LOC_Os12g36430	na
LOC_Os12g36430	pollination;anther development
LOC_Os09g39670	proteolysis;cellular process
LOC_Os04g04020	transport;response to oxidative stress;oxidation reduction
LOC_Os03g57300	na
LOC_Os03g57300	GPI anchor biosynthetic process;methylation
LOC_Os03g57300	na
LOC_Os03g57300	na
LOC_Os09g39670	regulation of catalytic activity
LOC_Os04g04020	protein amino acid dephosphorylation
LOC_Os03g57300	regulation of transcription from RNA polymerase II promoter;regula
LOC_Os03g57300	leaf senescence;cell death;ethylene biosynthetic process;defense re
LOC_Os03g57300	na
LOC_Os04g04020	na
LOC_Os12g36430	metabolic process
LOC_Os02g45250	methylation
LOC_Os04g04020	na
LOC_Os04g04020	RNA splicing, via endonucleolytic cleavage and ligation;transcriptior
LOC_Os04g04020	na
LOC_Os12g36430	iron ion transport;response to nematode;response to zinc ion;meta
LOC_Os03g57300	na
LOC_Os04g04020	na
LOC_Os04g04020	mitochondrial fission;mitochondrion organization;peroxisome fissio
LOC_Os12g36430	na
LOC_Os03g57300	sister chromatid cohesion;reciprocal meiotic recombination;chroma
LOC_Os03g57300	lipid transport
LOC_Os03g57300	na
LOC_Os04g04020	regulation of transcription, DNA-dependent;defense response;respo
LOC_Os04g04020	carbohydrate metabolic process;metabolic process
LOC_Os02g45250	regulation of transcription, DNA-dependent;cuticle development;ar
LOC_Os03g57300	na
LOC_Os04g04020	ATP catabolic process;proteolysis;misfolded or incompletely synthe:
LOC_Os09g39670	metabolic process

Do not distribute

LOC_Os03g57300	RNA processing;mRNA processing;regulation of timing of transition
LOC_Os04g04020	na
LOC_Os09g39670	tRNA aminoacylation for protein translation;threonyl-tRNA aminoac
LOC_Os03g57300	na
LOC_Os04g04020	na
LOC_Os12g36430	protein amino acid phosphorylation
LOC_Os03g57300	na
LOC_Os04g04020	na
LOC_Os03g57300	na
LOC_Os09g39670	metabolic process
LOC_Os04g04020	root hair cell tip growth;response to cytokinin stimulus;root epiderr
LOC_Os12g36430	na
LOC_Os04g04020	pollen tube growth;sexual reproduction
LOC_Os12g36430	na
LOC_Os09g39670	metabolic process
LOC_Os03g57300	protein import into nucleus;transport;protein transport
LOC_Os12g36430	oxidation reduction
LOC_Os04g04020	protein folding
LOC_Os06g36850	metabolic process
LOC_Os04g04020	metabolic process
LOC_Os04g04020	metabolic process;response to anoxia
LOC_Os12g36430	translation
LOC_Os02g45250	protein amino acid phosphorylation;phosphorylation
LOC_Os04g04020	RNA splicing, via endonucleolytic cleavage and ligation;transcriptior
LOC_Os04g04020	metabolic process;response to hypoxia
LOC_Os04g04020	RNA methylation;rRNA processing;tRNA processing
LOC_Os04g04020	root hair cell tip growth;response to salt stress
LOC_Os04g04020	na
LOC_Os06g36850	carbohydrate metabolic process;glucose metabolic process;pentose
LOC_Os06g36850	carbohydrate metabolic process;glucose metabolic process;pentose
LOC_Os06g36850	carbohydrate metabolic process;glucose metabolic process;pentose
LOC_Os06g36850	carbohydrate metabolic process;glucose metabolic process;pentose
LOC_Os06g36850	carbohydrate metabolic process;glucose metabolic process;pentose
LOC_Os04g04020	protein amino acid N-linked glycosylation;GPI anchor biosynthetic p
LOC_Os09g39670	metabolic process
LOC_Os12g36430	metabolic process
LOC_Os12g36430	protein amino acid phosphorylation
LOC_Os03g57300	flower development;vegetative to reproductive phase transition of
LOC_Os04g04020	1,3-beta-glucan biosynthetic process;microsporogenesis;pollen gerr
LOC_Os03g57300	na
LOC_Os04g04020	mitochondrial fission;mitochondrion organization;peroxisome fissio
LOC_Os03g57300	na
LOC_Os04g04020	transport;cation transport;transmembrane transport
LOC_Os03g57300	transport;intracellular protein transport;protein import into nucleus
LOC_Os06g36850	translation;tRNA aminoacylation for protein translation;tryptophan
LOC_Os03g57300	translation

Do not distribute

LOC_Os09g39670	xenobiotic metabolic process;metabolic process;response to toxin;r
LOC_Os06g36850	RNA processing;tRNA processing
LOC_Os09g39670	D-ribose metabolic process;phosphorylation
LOC_Os04g04020	post-embryonic development;vegetative to reproductive phase trar
LOC_Os09g39670	metabolic process
LOC_Os03g57300	transport
LOC_Os04g04020	na
LOC_Os09g39670	na
LOC_Os12g36430	plant-type cell wall modification;pollen germination;pollen tube gro
LOC_Os09g39670	na
LOC_Os09g39670	metabolic process;xenobiotic metabolic process;response to toxin;r
LOC_Os12g36430	metabolic process
LOC_Os06g36850	response to high light intensity;cell differentiation;oxidation reducti
LOC_Os06g36850	response to high light intensity;cell differentiation;oxidation reducti
LOC_Os12g36430	na
LOC_Os02g45250	GTP catabolic process
LOC_Os04g04020	na
LOC_Os04g04020	metabolic process
LOC_Os06g36850	metabolic process
LOC_Os03g57300	protein amino acid glycosylation;chromosome segregation;sister ch
LOC_Os12g36430	na
LOC_Os03g57300	signal peptide processing;proteolysis
LOC_Os12g36430	UDP-glucuronate biosynthetic process;oxidation reduction
LOC_Os03g57300	na
LOC_Os09g39670	metabolic process
LOC_Os06g36850	na
LOC_Os03g57300	na
LOC_Os06g36850	metabolic process
LOC_Os09g39670	metabolic process
LOC_Os04g04020	translation;cell proliferation;adaxial/abaxial pattern formation;leaf
LOC_Os03g57300	ATP catabolic process;transport;transmembrane transport;cell adhe
LOC_Os03g57300	protein folding;response to stress;response to chlorate;protein imp
LOC_Os09g39670	metabolic process
LOC_Os12g36430	na
LOC_Os03g57300	mitotic cell cycle;DNA metabolic process;RNA processing;sister chro
LOC_Os03g57300	microtubule-based movement
LOC_Os06g36850	histidine biosynthetic process
LOC_Os03g57300	translational initiation;protein targeting to mitochondrion
LOC_Os04g04020	nucleotide biosynthetic process;ribosome biogenesis
LOC_Os03g57300	mRNA export from nucleus;protein import into nucleus
LOC_Os04g04020	carbohydrate metabolic process;glucose 6-phosphate metabolic pro
LOC_Os04g04020	carbohydrate metabolic process;glycolysis;hexokinase-dependent si
LOC_Os04g04020	carbohydrate metabolic process;glycolysis;phosphorylation;respons
LOC_Os06g36850	gluconeogenesis;glycolysis;protein targeting to vacuole;response to
LOC_Os12g36430	na
LOC_Os12g36430	oxidation reduction

Do not distribute

LOC_Os03g57300	pseudouridine synthesis;protein folding;response to heat;RNA mod
LOC_Os04g04020	protein amino acid phosphorylation;multicellular organismal develo
LOC_Os12g36430	defense response
LOC_Os03g57300	protein import into nucleus
LOC_Os04g04020	na
LOC_Os06g36850	sulfur amino acid metabolic process;glycine catabolic process;unsat
LOC_Os06g36850	lipoate biosynthetic process;protein lipoylation
LOC_Os06g36850	na
LOC_Os04g04020	RNA methylation;rRNA processing;mRNA export from nucleus;prote
LOC_Os03g57300	na
LOC_Os02g45250	GTP catabolic process;translational elongation
LOC_Os04g04020	transport;cation transport;transmembrane transport;regulation of
LOC_Os09g39670	metabolic process
LOC_Os06g36850	sulfate assimilation;response to stress;metabolic process;cellular ar
LOC_Os04g04020	protein amino acid dephosphorylation
LOC_Os04g04020	rRNA processing;tRNA processing;methylation;RNA methylation
LOC_Os09g39670	flower development;meristem structural organization;phloem or xy
LOC_Os04g04020	na
LOC_Os03g57300	plastid organization;carotenoid biosynthetic process;oxidation redu
LOC_Os04g04020	protein amino acid phosphorylation
LOC_Os12g36430	na
LOC_Os06g36850	rRNA processing;tRNA metabolic process;tRNA aminoacylation for
LOC_Os09g39670	phosphoinositide dephosphorylation;response to auxin stimulus;res
LOC_Os09g39670	na
LOC_Os12g36430	na
LOC_Os04g04020	cell redox homeostasis;nuclear division;nucleolus organization;cellu
LOC_Os04g04020	na
LOC_Os04g04020	root epidermal cell differentiation;root hair elongation;response to
LOC_Os06g36850	maltose metabolic process;pentose-phosphate shunt;thiamin biosy
LOC_Os04g04020	protein amino acid phosphorylation;phosphorylation
LOC_Os04g04020	sister chromatid cohesion;reciprocal meiotic recombination;gravitro
LOC_Os09g39670	metabolic process
LOC_Os12g36430	xylem and phloem pattern formation;glucuronoxylan biosynthetic p
LOC_Os02g45250	double-strand break repair via homologous recombination;DNA rep
LOC_Os03g57300	response to abscisic acid stimulus;cell division
LOC_Os09g39670	metabolic process
LOC_Os03g57300	histone methylation;methylation;histone lysine methylation
LOC_Os09g39670	carbohydrate metabolic process;pentose-phosphate shunt;metabol
LOC_Os04g04020	translation
LOC_Os06g36850	tricarboxylic acid cycle;fumarate metabolic process;response to oxi
LOC_Os03g57300	carbohydrate metabolic process;glycolysis;pentose-phosphate shun
LOC_Os09g39670	metabolic process
LOC_Os12g36430	na
LOC_Os06g36850	tRNA aminoacylation for protein translation;lysyl-tRNA aminoacylat
LOC_Os04g04020	vesicle docking during exocytosis;vesicle-mediated transport;protei
LOC_Os04g04020	na

Do not distribute

LOC_Os04g04020	protein amino acid phosphorylation;phosphorylation
LOC_Os09g39670	metabolic process
LOC_Os12g36430	polysaccharide catabolic process;carbohydrate metabolic process;cl
LOC_Os03g57300	na
LOC_Os02g45250	mismatch repair;negative regulation of reciprocal meiotic recombin
LOC_Os09g39670	metabolic process
LOC_Os12g36430	na
LOC_Os03g57300	na
LOC_Os02g45250	na
LOC_Os03g57300	na
LOC_Os03g57300	mRNA export from nucleus;production of ta-siRNAs involved in RNA
LOC_Os04g04020	transport;sulfate transport;transmembrane transport
LOC_Os04g04020	sulfate transport;transmembrane transport
LOC_Os04g04020	mitotic cell cycle;cytokinesis by cell plate formation;establishment c
LOC_Os03g57300	na
LOC_Os04g04020	translation;ribosomal small subunit assembly;translational elongati
LOC_Os04g04020	na
LOC_Os03g57300	rRNA processing;tRNA metabolic process;translation;tRNA aminoac
LOC_Os09g39670	metabolic process
LOC_Os04g04020	translation;response to fungus
LOC_Os04g04020	protein amino acid dephosphorylation
LOC_Os09g39670	metabolic process
LOC_Os03g57300	na
LOC_Os02g45250	DNA topological change;chromosome segregation;resolution of mei
LOC_Os09g39670	metabolic process
LOC_Os03g57300	na
LOC_Os02g45250	protein amino acid glycosylation;cell-matrix adhesion
LOC_Os04g04020	small GTPase mediated signal transduction;response to cadmium io
LOC_Os06g36850	purine nucleotide biosynthetic process;cellular amino acid metaboli
LOC_Os04g04020	na
LOC_Os12g36430	na
LOC_Os04g04020	protein amino acid phosphorylation;auxin polar transport;root deve
LOC_Os04g04020	na
LOC_Os09g39670	tricarboxylic acid cycle;response to oxidative stress;metabolic proce
LOC_Os02g45250	DNA metabolic process;double-strand break repair;DNA repair;resp
LOC_Os03g57300	na
LOC_Os09g39670	metabolic process
LOC_Os03g57300	oxidation reduction
LOC_Os04g04020	na
LOC_Os02g45250	na
LOC_Os03g57300	transport;intracellular protein transport;ER to Golgi vesicle-mediate
LOC_Os04g04020	protein amino acid phosphorylation;phosphorylation
LOC_Os03g57300	protein amino acid glycosylation
LOC_Os03g57300	microtubule cytoskeleton organization;cytokinesis by cell plate forn
LOC_Os03g57300	protein amino acid phosphorylation
LOC_Os12g36430	na

Do not distribute

LOC_Os04g04020	sodium ion transport;transmembrane transport;malate transmemb
LOC_Os09g39670	branched chain family amino acid biosynthetic process;leucine bios
LOC_Os04g04020	proteolysis;fatty acid biosynthetic process;cellular process;oxidatior
LOC_Os09g39670	na
LOC_Os09g39670	na
LOC_Os12g36430	na
LOC_Os04g04020	multidimensional cell growth;vacuole organization;trichome morph
LOC_Os04g04020	proteolysis;cellular process;vernalization response;protein processi
LOC_Os04g04020	GTP catabolic process;SRP-dependent cotranslational protein target
LOC_Os09g23200	na
LOC_Os04g04020	glycyl-tRNA aminoacylation;response to cadmium ion
LOC_Os02g45250	DNA repair;protein ubiquitination
LOC_Os06g36850	acetyl-CoA metabolic process;metabolic process;embryonic develop
LOC_Os04g04020	na
LOC_Os06g36850	maltose catabolic process;carbohydrate metabolic process;starch ca
LOC_Os04g04020	long-day photoperiodism
LOC_Os04g04020	protein amino acid dephosphorylation
LOC_Os03g57300	regulation of cyclin-dependent protein kinase activity;regulation of
LOC_Os04g04020	na
LOC_Os04g04020	ubiquitin-dependent protein catabolic process
LOC_Os04g04020	phenylpropanoid metabolic process
LOC_Os12g36430	na
LOC_Os09g39670	metabolic process
LOC_Os03g57300	na
LOC_Os04g04020	na
LOC_Os03g57300	na
LOC_Os03g57300	protein amino acid phosphorylation;phosphorylation;protein amino
LOC_Os04g04020	na
LOC_Os12g36430	na
LOC_Os09g39670	na
LOC_Os09g39670	oxidation reduction
LOC_Os12g36430	transport;ion transport;potassium ion transport;response to extern
LOC_Os02g45250	na
LOC_Os09g39670	oxidation reduction;tricarboxylic acid cycle;electron transport chain
LOC_Os12g36430	na
LOC_Os06g36850	translation;translational elongation;embryonic development ending
LOC_Os04g04020	translation
LOC_Os09g39670	protein folding
LOC_Os06g36850	metabolic process
LOC_Os04g04020	tRNA wobble uridine modification;metabolic process;tRNA methyla
LOC_Os12g36430	na
LOC_Os03g57300	RNA splicing
LOC_Os02g45250	transcription, DNA-dependent
LOC_Os02g45250	na
LOC_Os09g39670	DNA replication;DNA methylation;proteolysis;cell proliferation;leuk
LOC_Os12g36430	very-long-chain fatty acid metabolic process;lipid metabolic process

Do not distribute

LOC_Os04g04020	translation
LOC_Os09g39670	organ growth;protein complex disassembly
LOC_Os04g04020	'de novo' IMP biosynthetic process;pollen development
LOC_Os09g39670	metabolic process;salicylic acid metabolic process;shoot morphoger
LOC_Os04g04020	response to oxidative stress;oxidation reduction
LOC_Os04g04020	response to oxidative stress;oxidation reduction;establishment of p
LOC_Os04g04020	metabolic process;oxidation reduction;pollen tube growth
LOC_Os09g39670	purine nucleotide biosynthetic process;nucleotide biosynthetic proc
LOC_Os09g39670	proline biosynthetic process;threonine catabolic process;cellular am
LOC_Os09g39670	glycolysis;metabolic process;response to salt stress;response to cad
LOC_Os09g39670	transport;intracellular protein transport;retrograde vesicle-mediate
LOC_Os03g57300	GPI anchor metabolic process;intracellular protein transport
LOC_Os04g04020	small GTPase mediated signal transduction;response to cadmium io
LOC_Os04g04020	protein amino acid glycosylation;cellulose biosynthetic process;Golg
LOC_Os09g39670	metabolic process;salicylic acid metabolic process;shoot morphoger
LOC_Os04g04020	na
LOC_Os12g36430	na
LOC_Os04g04020	na
LOC_Os04g04020	na
LOC_Os06g36850	tRNA modification;tRNA processing;queuosine biosynthetic process
LOC_Os09g39670	metabolic process
LOC_Os03g57300	na
LOC_Os09g39670	metabolic process
LOC_Os04g04020	na
LOC_Os09g39670	chromatin modification;histone deacetylation;root development;his
LOC_Os12g36430	na
LOC_Os04g04020	biosynthetic process;biotin biosynthetic process
LOC_Os04g04020	na
LOC_Os04g04020	DNA methylation;methylation
LOC_Os04g04020	na
LOC_Os12g36430	na
LOC_Os03g57300	na
LOC_Os04g04020	na
LOC_Os12g36430	na
LOC_Os12g36430	na
LOC_Os04g04020	na
LOC_Os12g36430	metabolic process;long-chain fatty acid metabolic process;defense r
LOC_Os02g45250	na
LOC_Os06g36850	carbohydrate metabolic process
LOC_Os06g36850	carbohydrate metabolic process
LOC_Os06g36850	carbohydrate metabolic process
LOC_Os06g36850	carbohydrate metabolic process
LOC_Os06g36850	carbohydrate metabolic process
LOC_Os06g36850	carbohydrate metabolic process
LOC_Os09g39670	acetyl-CoA metabolic process;polyamine catabolic process;steroid b
LOC_Os02g45250	na

Do not distribute

LOC_Os12g36430	na
LOC_Os03g57300	na
LOC_Os04g04020	na
LOC_Os02g45250	RNA processing
LOC_Os02g45250	na
LOC_Os02g45250	na
LOC_Os12g36430	na
LOC_Os09g39670	na
LOC_Os12g36430	protein amino acid phosphorylation;phosphorylation
LOC_Os09g39670	metabolic process
LOC_Os02g45250	protein amino acid glycosylation
LOC_Os09g39670	aromatic amino acid family metabolic process;vitamin E biosyntheti
LOC_Os09g39670	metabolic process
LOC_Os04g04020	protein folding;oxidation reduction
LOC_Os03g57300	response to cadmium ion
LOC_Os03g57300	cation transport;single fertilization;pollen development;response to
LOC_Os09g39670	protein folding
LOC_Os03g57300	na
LOC_Os12g36430	protein amino acid phosphorylation;response to stress;phosphoryla
LOC_Os09g39670	ATP catabolic process;porphyrin biosynthetic process;photosynthes
LOC_Os02g45250	oxidation reduction
LOC_Os02g45250	protein amino acid glycosylation;transport
LOC_Os02g45250	transcription, DNA-dependent;regulation of transcription, DNA-dep
LOC_Os09g23200	na
LOC_Os09g39670	metabolic process
LOC_Os09g39670	GPI anchor biosynthetic process
LOC_Os04g04020	translation
LOC_Os02g45250	na
LOC_Os12g36430	na
LOC_Os06g36850	ubiquinone biosynthetic process;methylation
LOC_Os04g04020	carbohydrate metabolic process;glycolysis;hexokinase-dependent si
LOC_Os04g04020	metabolic process;oxidation reduction
LOC_Os02g45250	cytoskeleton organization;actin filament severing;actin filament bur
LOC_Os09g39670	na
LOC_Os09g39670	metabolic process
LOC_Os12g36430	protein amino acid phosphorylation;root development;calcium-mec
LOC_Os12g36430	na
LOC_Os04g04020	na
LOC_Os04g04020	na
LOC_Os03g57300	na
LOC_Os03g57300	na
LOC_Os06g36850	tRNA aminoacylation for protein translation;methionyl-tRNA amino
LOC_Os12g36430	regulation of transcription, DNA-dependent
LOC_Os04g04020	metabolic process;oxidation reduction
LOC_Os03g57300	na
LOC_Os04g04020	protein targeting to vacuole;transport;cation transport;regulation o

Do not distribute

LOC_Os03g57300	transcription, DNA-dependent;regulation of transcription, DNA-dep
LOC_Os03g57300	na
LOC_Os04g04020	translation
LOC_Os09g39670	metabolic process
LOC_Os09g39670	translation
LOC_Os09g39670	cation transport;manganese ion transport;response to manganese i
LOC_Os06g36850	iron-sulfur cluster assembly
LOC_Os03g57300	na
LOC_Os04g04020	regulation of Rab GTPase activity;positive regulation of Rab GTPase
LOC_Os12g36430	na
LOC_Os04g04020	metabolic process
LOC_Os03g57300	protein amino acid glycosylation;plant-type hypersensitive response
LOC_Os03g57300	mRNA processing
LOC_Os03g57300	metal ion transport;transmembrane transport
LOC_Os03g57300	carbohydrate metabolic process
LOC_Os09g39670	phosphorylation;glycerophospholipid biosynthetic process
LOC_Os09g39670	phosphorylation;glycerophospholipid biosynthetic process
LOC_Os09g39670	formation of translation preinitiation complex;translation;translatio
LOC_Os09g39670	malate metabolic process;oxidation reduction
LOC_Os09g39670	proteolysis involved in cellular protein catabolic process;proteolysis
LOC_Os09g39670	na
LOC_Os09g39670	na
LOC_Os04g04020	translation
LOC_Os06g36850	na
LOC_Os03g57300	na
LOC_Os03g57300	na
LOC_Os06g36850	na
LOC_Os09g39670	iron-sulfur cluster assembly
LOC_Os09g39670	metabolic process
LOC_Os04g04020	protein targeting to vacuole;signal transduction;vesicle-mediated tr
LOC_Os09g39670	small GTPase mediated signal transduction;protein transport;respo
LOC_Os03g57300	response to auxin stimulus;jasmonic acid mediated signaling pathw
LOC_Os04g04020	cell morphogenesis;cell growth;Golgi vesicle transport
LOC_Os06g36850	na
LOC_Os06g36850	rRNA processing;tRNA metabolic process;tRNA aminoacylation for p
LOC_Os06g36850	tRNA aminoacylation for protein translation;valyl-tRNA aminoacylat
LOC_Os06g36850	translation;tRNA aminoacylation for protein translation;valyl-tRNA a
LOC_Os06g36850	tRNA aminoacylation for protein translation;valyl-tRNA aminoacylat
LOC_Os09g39670	na
LOC_Os04g04020	nucleosome assembly;regulation of DNA methylation;regulation of
LOC_Os04g04020	na
LOC_Os09g39670	metabolic process;methylation
LOC_Os09g39670	protein folding
LOC_Os06g36850	protein folding
LOC_Os09g39670	protein folding;response to heat;regulation of ATPase activity;positi
LOC_Os06g36850	translation;tRNA aminoacylation for protein translation;glutaminyl-

Do not distribute

LOC_Os06g36850	translation;tRNA aminoacylation for protein translation;glutaminyl-t
LOC_Os02g45250	na
LOC_Os04g04020	metabolic process;oxidation reduction
LOC_Os03g57300	na
LOC_Os04g04020	na
LOC_Os04g04020	na
LOC_Os06g36850	proteolysis
LOC_Os04g04020	protein amino acid dephosphorylation
LOC_Os04g04020	intra-Golgi vesicle-mediated transport
LOC_Os12g36430	na
LOC_Os04g04020	intra-Golgi vesicle-mediated transport;cellulose biosynthetic proces
LOC_Os06g36850	na
LOC_Os03g57300	MAPKKK cascade;GTP catabolic process;regulation of transcription,
LOC_Os09g39670	intracellular protein transport;retrograde vesicle-mediated transpoi
LOC_Os09g39670	isoprenoid biosynthetic process;isopentenyl diphosphate biosynthe
LOC_Os04g04020	defense response to oomycetes;carbohydrate metabolic process;pe
LOC_Os04g04020	steroid metabolic process
LOC_Os03g57300	transcription, DNA-dependent;regulation of transcription, DNA-dep
LOC_Os12g36430	na
LOC_Os03g57300	reproduction;G2 phase of mitotic cell cycle;mitotic cell cycle;proteir
LOC_Os12g36430	defense response
LOC_Os03g57300	intracellular protein transport;vesicle-mediated transport
LOC_Os09g39670	peptidyl-lysine modification to hypusine;oxidation reduction
LOC_Os04g04020	mRNA catabolic process;leaf morphogenesis;deadenylation-indeper
LOC_Os12g36430	na
LOC_Os04g04020	translation
LOC_Os03g57300	na
LOC_Os03g57300	RNA splicing;regulation of viral protein levels in host cell
LOC_Os03g57300	transcription, DNA-dependent
LOC_Os09g39670	proteolysis;ubiquitin-dependent protein catabolic process;response
LOC_Os03g57300	na
LOC_Os06g36850	dUTP metabolic process;dUMP biosynthetic process;DNA-depender
LOC_Os12g36430	na
LOC_Os06g36850	iron-sulfur cluster assembly
LOC_Os03g57300	RNA splicing, via endonucleolytic cleavage and ligation;N-glycan prc
LOC_Os12g36430	na
LOC_Os04g04020	na
LOC_Os03g57300	regulation of transcription, DNA-dependent;response to osmotic str
LOC_Os04g04020	regulation of transcription, DNA-dependent;autophagy;response to
LOC_Os03g57300	RNA splicing, via endonucleolytic cleavage and ligation;mRNA expor
LOC_Os06g36850	na
LOC_Os06g36850	embryo sac development;pollen development;glucose mediated sig
LOC_Os06g36850	protein catabolic process
LOC_Os06g36850	proteolysis;protein catabolic process
LOC_Os06g36850	proteolysis;protein catabolic process;ATP catabolic process;respons
LOC_Os06g36850	proteolysis;photoinhibition;protein catabolic process;cell cycle;cell (

Do not distribute

LOC_Os06g36850	protein catabolic process
LOC_Os06g36850	ATP catabolic process
LOC_Os06g36850	protein catabolic process
LOC_Os06g36850	proteolysis;cell division;protein catabolic process
LOC_Os06g36850	proteolysis;protein catabolic process
LOC_Os06g36850	proteolysis;response to heat;meristem structural organization;leaf r
LOC_Os06g36850	proteolysis;protein catabolic process;ATP catabolic process;protein
LOC_Os06g36850	proteolysis;protein catabolic process
LOC_Os06g36850	protein catabolic process
LOC_Os06g36850	proteolysis;thylakoid membrane organization;photoinhibition;prote
LOC_Os06g36850	protein catabolic process;gluconeogenesis;ATP catabolic process;ub
LOC_Os06g36850	embryo sac development;pollen development;glucose mediated sig
LOC_Os06g36850	response to heat;meristem structural organization;leaf morphogene
LOC_Os06g36850	proteolysis;protein catabolic process
LOC_Os04g04020	transport;cation transport;transmembrane transport
LOC_Os03g57300	activation of protein kinase C activity by G-protein coupled receptor
LOC_Os03g57300	glucose catabolic process
LOC_Os04g04020	hyperosmotic salinity response;response to cold;response to water
LOC_Os12g36430	ubiquitin-dependent protein catabolic process
LOC_Os04g04020	regulation of transcription, DNA-dependent;histone acetylation;tran
LOC_Os04g04020	ribosomal small subunit assembly;translation;translational elongati
LOC_Os04g04020	transport;ion transport;potassium ion transport;calcium ion transpo
LOC_Os04g04020	na
LOC_Os09g39670	formation of translation preinitiation complex;translation;translatio
LOC_Os04g04020	D-xylose metabolic process;cellular metabolic process
LOC_Os03g57300	chromatin organization;developmental process;multicellular organi
LOC_Os12g36430	defense response;metabolic process
LOC_Os04g04020	retrograde vesicle-mediated transport, Golgi to ER
LOC_Os04g04020	na
LOC_Os03g57300	na
LOC_Os04g04020	na
LOC_Os04g04020	formation of translation preinitiation complex;translation;translatio
LOC_Os03g57300	GTP catabolic process
LOC_Os04g04020	na
LOC_Os04g04020	transport;cation transport;transmembrane transport
LOC_Os09g39670	glycolysis;metabolic process;response to salt stress;response to cad
LOC_Os12g36430	metabolic process
LOC_Os09g23200	na
LOC_Os12g36430	proteolysis
LOC_Os04g04020	metabolic process;oxidation reduction
LOC_Os04g04020	retrograde vesicle-mediated transport, Golgi to ER
LOC_Os06g36850	DNA-dependent DNA replication;metabolic process;toxin catabolic
LOC_Os09g39670	formation of translation preinitiation complex;translation;translatio

Do not distribute

ed transport;protein transport
ed transport;response to oxidative stress;protein transport
rt;transport;protein transport
rt

nediated transport
nediated transport
nediated transport
nediated transport
drolysis coupled proton transport;proton transport;ATP metabolic process;response to cac

n reduction
n reduction

gi vesicle transport
nediated transport
oton transport;ATP metabolic process;negative regulation of actin filament depolymerizatio

gravitropism

ty

n catabolic process

wering
ulation of transcription, DNA-dependent;positive regulation of cell proliferation;gravitropism
oRNA processing;shoot development;regulation of meristem development
oRNA processing;shoot development;regulation of meristem development

omosome segregation;post-embryonic development;embryonic development ending in se
nediated transport

Do not distribute

transport;protein secretion;Golgi organization

sponse to water deprivation;response to abscisic acid stimulus;regulation of stomatal move
gulation of protein kinase activity;protein amino acid autophosphorylation
e;response to heat;response to bacterium;response to temperature stimulus;response to h

nediated transport

sponse to salt stress
cadmium ion

sponse to salt stress
sponse to salt stress

sponse to water deprivation;response to abscisic acid stimulus;regulation of stomatal move

nediated transport
bacterium;response to cadmium ion
stimulus;unidimensional cell growth;protein polymerization
nium ion;protein polymerization
sponse to water deprivation;response to abscisic acid stimulus;regulation of stomatal move
nium ion;protein polymerization
c process;phosphatidylinositol metabolic process;phosphoinositide phosphorylation;phospl
e;telomere maintenance;cytokinesis by cell plate formation;double-strand break repair;mitc
zation;unidimensional cell growth;response to light stimulus
etabolic process

zation;response to cold;response to salt stress
zation;response to salt stress;response to cadmium ion
response to salt stress;protein polymerization
nicrotubule-based process;microtubule-based movement;response to salt stress;proteason
sponse to water deprivation;response to abscisic acid stimulus;regulation of stomatal move

cidification;vacuole organization

activity;response to cadmium ion

Do not distribute

combination;regulation of flower development;histone modification;methylglyoxal catabol

organization;vacuole organization;amyloplast organization;embryonic development endin
endent;epidermal cell differentiation;cotyledon development

thetic process;cysteine biosynthetic process;aging;response to cadmium ion

vacuole;Golgi to vacuole transport;cell communication;endosome to lysosome transport;pl

cycle
;tissue development;positive regulation of organelle organization;vesicle-mediated transpo

ediated transport

xidative stress
e transport;carbohydrate transport

biosynthetic process;response to zinc ion;cysteine biosynthetic process;response to cadmi

n targeting to vacuole;defense response to fungus

nthetic process;metabolic process

ell growth;protein amino acid N-linked glycosylation via asparagine;cellulose biosynthetic p

endent;shade avoidance;response to hormone stimulus;response to far red light;shoot mo
n transport;calcium ion transport
lopment

zation
zation;cortical microtubule organization;cell growth
thetic process;cysteine biosynthetic process;pollen tube development

Do not distribute

zation;cellular response to gravity;microtubule cytoskeleton organization;response to salt s

nse to UV-B
:y;regulation of cell cycle;response to salicylic acid stimulus
nium ion;protein polymerization;cellular response to gravity

sphorylation

:y;regulation of cell cycle
:y
tion of ta-siRNAs involved in RNA interference;maintenance of shoot apical meristem ident
pendent;leaf development;carpel development;stamen development;maintenance of floral

response to salt stress;xenobiotic catabolic process

sponse to virus;response to heat

nutrient;reproductive developmental process;primary root development;vegetative phase c
um ion transmembrane transport

;

se;pollen development;response to fungus;salicylic acid mediated signaling pathway;defens

Do not distribute

-mediated transport;response to abscisic acid stimulus;protein transport;vesicle-mediated

c process;phosphatidylinositol metabolic process;phosphoinositide phosphorylation;phospl

ıthetic process;cysteine biosynthetic process;photosynthetic acclimation;regulation of hydı

nse to UV-B

ess;regulation of transcription from RNA polymerase II promoter;regulation of RNA elongat

ıendent;anthocyanin accumulation in tissues in response to UV light;root development;mai

um

ɔRNA processing;shoot development;regulation of meristem development
ıe organization

oton transport

amage stimulus

ediated transport;regulation of intracellular pH

Do not distribute

ıediated transport

ɔacylation;response to cadmium ion
ı;hydrogen peroxide catabolic process

ıg

ıtion of flower development

duction
ı ion;translation
ı ion;translation
ı ion;translation

atin silencing;arginine biosynthetic process;nitrogen compound metabolic process;cytoske
lar response to sulfate starvation;cyanide metabolic process;response to cadmium ion;detc

ıembrane transport
n secretion;transport;protein transport
ɟependent protein catabolic process

le transport

ɟependent protein catabolic process

ve regulation of cellular response to phosphate starvation

id stimulus;negative regulation of transcription, DNA-dependent

Do not distribute

n catabolic process;DNA repair;response to DNA damage stimulus

ependent

ynthetic process;metabolic process;fatty acid elongation;shoot development;response to cy

s
membrane transport

cle;cytokinesis by cell plate formation;chromatin organization;RNA processing;fatty acid be
ransport;Golgi organization;vacuole organization;response to salt stress;ATP hydrolysis cou

Do not distribute

ar red light;embryonic development ending in seed dormancy;positive regulation of flower

nation;translation;translational initiation;regulation of translation;response to virus;gravitr

ponse to cadmium ion
s;ER to Golgi vesicle-mediated transport;hyperosmotic response;hyperosmotic salinity resp

cription, DNA-dependent;response to stress

tion, DNA-dependent;cell adhesion;trichome morphogenesis;vegetative to reproductive ph

rt

:hloroplast relocation;thylakoid membrane organization;ncRNA metabolic process;protein i

ponse to cadmium ion

pendent;defense response to bacterium;positive regulation of transcription, DNA-depender
pendent
pendent
tion, DNA-dependent;response to heat;transcription, DNA-dependent;response to stress

pendent;response to abscisic acid stimulus;response to sucrose stimulus;positive regulation

pendent;regulation of developmental process;response to ethylene stimulus
nscription, DNA-dependent;maintenance of floral organ identity
lar response to sulfate starvation;cyanide metabolic process;response to cadmium ion;detc

process;very-long-chain fatty acid metabolic process;response to cold;response to light stir

mination;response to abscisic acid stimulus

nembrane transport
nembrane transport

:in mediated signaling pathway;auxin polar transport;positive gravitropism;phosphorylatior
nembrane transport
n transport;defense response to fungus
coat development;regulation of gene expression;trichome morphogenesis
pendent

Do not distribute

endent;auxin mediated signaling pathway;response to auxin stimulus
endent;response to cytokinin stimulus

l adhesion;plant-type cell wall biogenesis;organ morphogenesis;tissue development;tricho

endent;negative regulation of transcription, DNA-dependent;response to abscisic acid stim

hogenesis;vegetative to reproductive phase transition of meristem;histone H2B ubiquitina
ance;protein refolding;cellular protein metabolic process;chaperone mediated protein fold
rotein refolding;cellular protein metabolic process
netabolic process;response to cadmium ion
onse to heat;response to high light intensity;embryonic development ending in seed dorm
netabolic process;response to cadmium ion
;response to cadmium ion
otein metabolic process;chaperone mediated protein folding requiring cofactor;protein fol
ization;embryonic development

ive regulation of flower development;leaf morphogenesis;vegetative to reproductive phase

cription, DNA-dependent;response to stress

endent
endent;auxin mediated signaling pathway;cotyledon vascular tissue pattern formation;sko
structural organization;petal development;petal morphogenesis
hogenesis;vegetative to reproductive phase transition of meristem;histone H2B ubiquitina

embrane transport

r protein signaling pathway;metabolic process;phosphorylation;cellular response to abscisi
to abscisic acid stimulus

endent;auxin mediated signaling pathway;response to auxin stimulus

Do not distribute

transcription, DNA-dependent;ovule development;seed development

endent;response to cold;systemic acquired resistance, salicylic acid mediated signaling pat

endent;ethylene mediated signaling pathway
endent;auxin mediated signaling pathway

ependent;embryo sac egg cell differentiation;xylem development
;intracellular protein transport;vesicle-mediated transport

Do not distribute

embrane transport

to cadmium ion

nsport;cellulose biosynthetic process;response to cadmium ion;Golgi vesicle transport

bohydrate transmembrane transport;amino acid import;response to salt stress;response tc
ansport;transmembrane transport
abolic process;response to cold;response to salt stress;defense response to bacterium;cell

endent;multicellular organismal development
endent
oliferation;specification of carpel identity;specification of stamen identity;cell differentiatic
catabolic process;methylation-dependent chromatin silencing;virus induced gene silencing;

ic development ending in seed dormancy;protein deubiquitination;root hair elongation
on;protein import into peroxisome matrix;calcium-mediated signaling;photoperiodism, flov

n;mitotic cell cycle;cytokinesis by cell plate formation;chromatin silencing;protein amino ac

ɪendent

stem;DNA replication;DNA repair;transcription, DNA-dependent;regulation of transcription

ɪendent;response to water deprivation;response to wounding;gravitropism;auxin mediated

argeting to vacuole;gravitropism
nediated transport

de-mediated signaling;protein amino acid phosphorylation

activity by G-protein coupled receptor protein signaling pathway;metabolic process;respon

ɪendent;auxin mediated signaling pathway

abolic process;response to cold;response to salt stress;defense response to bacterium;cell

abolic process;protein ubiquitination;xylan biosynthetic process

nse to cold;positive regulation of catalytic activity;cell redox homeostasis;oxidation reducti
nthetic process;cysteine biosynthetic process

Do not distribute

ɔe cell wall biogenesis;defense response to bacterium;response to salt stress;Golgi organiza
nicrotubule-based process;microtubule-based movement;proteasomal protein catabolic pr

protein amino acid methylation;embryo sac egg cell differentiation;photomorphogenesis;e
mediated transport;proteasomal ubiquitin-dependent protein catabolic process;proteasom

ɔtein amino acid acetylation;flower development
ıtion of flower development

ɛsponse to salt stress;response to sucrose stimulus;response to glucose stimulus;response t
se stimulus;response to glucose stimulus;response to fructose stimulus;response to cadmiu

ɔendent

process;negative regulation of programmed cell death;oxidation reduction

amage stimulus
activity
ine catabolic process;fatty acid beta-oxidation;response to sucrose stimulus;response to fru

vesicle-mediated transport
development ending in seed dormancy;auxin metabolic process;leaf morphogenesis;respoı

n;male meiosis;female meiosis

ɔlication;DNA repair;DNA recombination;mitotic recombination;regulation of transcription,

sembly
ar red light;embryonic development ending in seed dormancy;positive regulation of flower
ɔendent;trichome morphogenesis;DNA endoreduplication;regulation of cell division;cell cyc
ɾNA aminoacylation;tRNA aminoacylation

;photosynthesis;starch biosynthetic process;photosynthesis, light reaction;positive regulati
ecay;response to sucrose stimulus;hormone-mediated signaling pathway;sugar mediated s

Do not distribute

ɪnation;transcription, DNA-dependent;regulation of transcription, DNA-dependent;petal for

ɪs;regulation of flower development

ɔacylation;glutamyl-tRNA aminoacylation

nse to cold;positive regulation of catalytic activity;cell redox homeostasis;oxidation reducti
ɔendent;auxin mediated signaling pathway;response to auxin stimulus
desiccation;response to water deprivation;response to salt stress;response to abscisic acid
ɪatode;cell division

cription, DNA-dependent;response to stress
tress;response to ethylene stimulus;response to auxin stimulus;response to abscisic acid st
ɪther development;anther morphogenesis;tapetal cell differentiation;tapetal layer developı
ɪcclimation;proteasomal protein catabolic process;protein sumoylation;negative regulation
ɔendent;regulation of cell shape;ovule development;seed development

ʒe system

ɔnal initiation;regulation of translational initiation;photomorphogenesis;response to salt stı

ɪnulus

sitive regulation of flavonoid biosynthetic process;trichome differentiation;regulation of plɛ

ɔendent;auxin mediated signaling pathway;response to auxin stimulus

Do not distribute

: process;xylan biosynthetic process
:ion;response to heat;heat acclimation

ar red light;embryonic development ending in seed dormancy;positive regulation of flower

)endent;auxin mediated signaling pathway;response to auxin stimulus
)endent;defense response

;cell division

process;biosynthetic process;negative regulation of programmed cell death;oxidation redu

:abolic process;response to salt stress;response to zinc ion;cellular carbohydrate metabolic

oduction of ta-siRNAs involved in RNA interference;production of miRNAs involved in gene

ıse to cadmium ion;oxidation reduction
ic process;response to absence of light;response to salt stress;response to cadmium ion;reg
ıthetic process;response to cold;cellular response to sulfate starvation;cysteine biosyntheti

: process;regulation of programmed cell death;cell growth
:ion;response to hydrogen peroxide

desiccation;response to water deprivation;response to salt stress;response to abscisic acid

Do not distribute

ransport

process;metabolic process;purine base biosynthetic process;nucleoside metabolic process;l

pathway
endent;response to xenobiotic stimulus;response to light stimulus;photomorphogenesis;flo

pendent

development ending in seed dormancy;auxin metabolic process;leaf morphogenesis;respo
nse to cadmium ion;oxidation reduction

pendent

nse to salt stress;response to cadmium ion
glycosylation;N-terminal protein myristoylation;ER to Golgi vesicle-mediated transport;res
pendent;regulation of timing of transition from vegetative to reproductive phase;anther de

nt ending in seed dormancy

al transduction;protein transport;secretion by cell

amage stimulus

pendent
opment;response to auxin stimulus;auxin mediated signaling pathway;flower development;

pendent

Do not distribute

:ion;response to hydrogen peroxide;response to stress
amage stimulus
oton transport
ıs
endent

:;protein transport

um;interspecies interaction between organisms

sion;innate immune response

rocess;pteridine and derivative metabolic process;cellular metabolic process;tetrahydrofola

nsport;protein import into peroxisome matrix;transmembrane transport

osmotic stress;response to nematode;response to salt stress;defense response to bacteriu

ethylene stimulus;embryonic development ending in seed dormancy;flower development
endent;multicellular organismal development;flower development;cell differentiation;spec
ellular response to nitrogen starvation;positive regulation of cell proliferation;asymmetric c
acid autophosphorylation

protein location
in amino acid dephosphorylation

duction

Do not distribute

:response to salt stress;defense response to bacterium;negative regulation of programmed
›endent

ıthetic process;cysteine biosynthetic process

mulus;unidimensional cell growth;auxin metabolic process;root development;phosphoryla

ıcleotide transport;ER to Golgi vesicle-mediated transport;regulation of plant-type hyperseı

duction

ediated signaling pathway;auxin polar transport;regulation of stomatal movement;respons
›endent

um ion transmembrane transport
transport;transmembrane transport;shoot development

um

nt;vegetative to reproductive phase transition of meristem;inflorescence development;hist

ponse to wounding

:s;methionine catabolic process via 2-oxobutanoate;protein homotetramerization;cellular r

Do not distribute

) acid autophosphorylation

atin silencing;cell-cell signaling;virus induced gene silencing;determination of bilateral sym
cosylation;intracellular protein transport;vacuole organization;response to red or far red lig
NA aminoacylation;response to cadmium ion

MAPK activity;defense response, incompatible interaction;induced systemic resistance, jas
virus;flower development;leaf development;carpel development;regulation of viral reprodu
horylation
response to gibberellin stimulus;starch catabolic process;sucrose catabolic process;metabo
plasmic transport;response to virus

Do not distribute

ise to water deprivation;response to salt stress;response to abscisic acid stimulus

ation
-dependent;histone H4 acetylation;histone H2A acetylation;negative regulation of transcrip
nse to stress;xyloglucan biosynthetic process;root epidermal cell differentiation;cell wall bi
:;cation transport;sodium ion transport;regulation of pH;transmembrane transport
esicle-mediated transport;amino acid import

ation;response to cadmium ion
e growth;pollen tube guidance;pollen tube growth;response to cadmium ion

activity

ter;protein ubiquitination

sis involved in cellular protein catabolic process

onse

ɔort;protein transport
neostasis;pollen maturation;pollen germination;meristem maintenance;cellular response t

ɪn transport;calcium ion transport

ɔ acid autophosphorylation
on synthesis

ecay;protein export from nucleus;endoplasmic reticulum organization;secondary cell wall b
ɔn
ℜNA aminoacylation;mitochondrion organization;chloroplast organization;tRNA aminoacyla

nse to stress;xyloglucan biosynthetic process;root epidermal cell differentiation;cell wall bi

ɔcess;response to cadmium ion;oxidation reduction
ɜntose-phosphate shunt;defense response to bacterium;cellular response to redox state

ɔcess;response to cadmium ion;oxidation reduction

ɜss;attachment of GPI anchor to protein

sis involved in cellular protein catabolic process

ɪendent;response to stress;positive regulation of transcription, DNA-dependent

mRNA catabolic process;regionalization;RNA processing;mRNA export from nucleus;protei

Do not distribute

oxide catabolic process;cell redox homeostasis;oxidation reduction
processing;phloem or xylem histogenesis;production of siRNA involved in RNA interference
is;tissue development;trichome morphogenesis;vegetative to reproductive phase transition

af morphogenesis;trichome branching;seed development;cotyledon morphogenesis;positiv

development ending in seed dormancy;auxin metabolic process;leaf morphogenesis;respo

onse to salt stress;response to zinc ion;response to cadmium ion
o symbiotic fungus

virus;flower development;leaf development;carpel development;regulation of viral reprodu

development ending in seed dormancy;auxin metabolic process;leaf morphogenesis;respo
onse to salt stress;response to zinc ion;response to cadmium ion

osynthetic process;folic acid and derivative biosynthetic process;embryonic development er

ylation-dependent chromatin silencing;regulation of transcription, DNA-dependent;RNA pr

sponse to zinc ion;ribosome biogenesis;response to cadmium ion

duction
rocess;tryptophan catabolic process;nitrogen compound metabolic process;metabolic proc
ting to membrane

acid autophosphorylation

endent;positive regulation of meiotic cell cycle;positive regulation of cell proliferation;resp

Do not distribute

cytosol to ER transport

ation of development, heterochronic;regulation of radial pattern formation
esponse

ı from RNA polymerase II promoter;negative regulation of abscisic acid mediated signaling

l ion transport;iron ion transmembrane transport;transmembrane transport;zinc ion transı

on

atin silencing by small RNA;regulation of chromosome organization;meiotic DNA double-str

onse to water deprivation;jasmonic acid mediated signaling pathway;stomatal movement;c

ıthocyanin accumulation in tissues in response to UV light;root hair cell differentiation;root

sized protein catabolic process;protein catabolic process;cellular response to oxidative stre

Do not distribute

from vegetative to reproductive phase

cylation;tRNA aminoacylation

nal cell differentiation;root hair elongation;response to red light;response to far red light;r

, DNA-dependent;transcription from RNA polymerase II promoter

e-phosphate shunt;oxidation reduction;pentose-phosphate shunt, oxidative branch;respons
e-phosphate shunt;pentose-phosphate shunt, oxidative branch;response to cadmium ion;o
e-phosphate shunt;pentose-phosphate shunt, oxidative branch;oxidation reduction
e-phosphate shunt;oxidation reduction
e-phosphate shunt;pentose-phosphate shunt, oxidative branch;oxidation reduction
process;ER-associated protein catabolic process

meristem;phyllome development
mination;pollen tube growth;pollen wall assembly;regulation of pollen tube growth

on

s;regulation of RNA splicing
yl-tRNA aminoacylation;ovule development

Do not distribute

response to salt stress;xenobiotic catabolic process

nsition of meristem;carpel development;stamen development;histone H3-K4 methylation

owth;regulation of ARF protein signal transduction

response to salt stress;xenobiotic catabolic process

ion
ion

romatid cohesion;synapsis;reciprocal meiotic recombination;organ morphogenesis;tissue c

morphogenesis;root morphogenesis
esion;regulation of cell size;response to blue light;response to red or far red light;photomor
ort into chloroplast stroma;de-etiolation;response to heat;response to salt stress;response

omatid cohesion;meiosis;synapsis;reciprocal meiotic recombination;positive regulation of c

ocess
ignaling;sugar mediated signaling pathway;glucose mediated signaling pathway;programm
se to cold;response to salt stress;response to osmotic stress
o salt stress;response to cadmium ion

Do not distribute

ification;response to high light intensity;response to endoplasmic reticulum stress;respons
opment;auxin mediated signaling pathway;phosphorylation;cotyledon development

turated fatty acid biosynthetic process;oxidoreduction coenzyme metabolic process;vitamir

ein import into nucleus;sister chromatid cohesion;photomorphogenesis;embryonic develop

pH

nino acid biosynthetic process;cysteine biosynthetic process;sulfate reduction;cell redox hc

/lem histogenesis;leaf vascular tissue pattern formation;cotyledon vascular tissue pattern fo

iction

protein translation;lysyl-tRNA aminoacylation;chloroplast organization;embryonic developr
sponse to abscisic acid stimulus;response to jasmonic acid stimulus;phosphoinositide phos

ulose biosynthetic process;Golgi vesicle transport

light stimulus;response to auxin stimulus;root development;response to wounding;seed ge
nthetic process;response to vitamin B1;detection of bacterium;starch biosynthetic process

opism;organ morphogenesis;tissue development;vegetative to reproductive phase transitic

process;multicellular organism reproduction;leaf development;cell wall thickening
plication;DNA-dependent DNA replication;DNA replication initiation;regulation of DNA repli

lic process;response to cold;response to nematode;embryonic development ending in seed

dative stress;response to salt stress;nitrate assimilation;pollen tube development
it;valine metabolic process;oxidation reduction

tion;translation
n targeting to membrane;protein targeting to vacuole;calcium ion transport;anion transpor

Do not distribute

hitin catabolic process;defense response;metabolic process;jasmonic acid and ethylene-de[illegible]

ıation;pyrimidine dimer repair

\ interference;gene silencing by RNA;defense response to fungus

of planar polarity;regionalization;gluconeogenesis;endocytosis;cytoskeleton organization;si

on

ylation for protein translation;chloroplast organization;ovule development

iotic recombination intermediates;meiosis;mitosis;DNA repair

ɔn;protein amino acid ADP-ribosylation;transport;intracellular protein transport;protein tra
ic process;ornithine metabolic process;ovule development

elopment;cotyledon development

ess
ɔonse to DNA damage stimulus;meiosis

ed transport;protein transport

nation;gravitropism

Do not distribute

ɔrane transport
ynthetic process;oxidation reduction;response to salt stress;glucosinolate biosynthetic proc
n reduction

ıogenesis;response to abscisic acid stimulus
ng;N-terminal protein amino acid modification;anthocyanin accumulation in tissues in resp
ting to membrane

pment ending in seed dormancy;sterol biosynthetic process;brassinosteroid biosynthetic pr

atabolic process;glucose metabolic process

transcription, DNA-dependent;RNA processing;response to salt stress;stomatal lineage pro

ɔ acid autophosphorylation

al stimulus

ı

ʒ in seed dormancy;chloroplast relocation;thylakoid membrane organization;carotenoid bic

tion;methylation

ɔtriene biosynthetic process;histone H3-K9 methylation
ɜ;cuticle development

Do not distribute

nesis;benzoate metabolic process;cellular response to water deprivation;para-aminobenzo

ılanar polarity;positive regulation of abscisic acid mediated signaling pathway;callose depos

cess;arginine biosynthetic process via ornithine
nino acid biosynthetic process;response to heat;response to salt stress;oxidation reduction;
lmium ion;oxidation reduction
ed transport, Golgi to ER;vesicle-mediated transport;protein transport

on;transport;protein transport;vesicle-mediated transport
gi vesicle transport;response to ammonium ion
nesis;benzoate metabolic process;glucosinolate biosynthetic process;cellular response to w

;

stone H3-K4 methylation;oxidation reduction

Do not distribute

response to insect;fatty acid metabolic process;fatty acid biosynthetic process;wax biosynth

biosynthetic process;metabolic process;response to wounding;phenylpropanoid metabolic

ic process;plastoquinone biosynthetic process;carotenoid biosynthetic process;oxidation re

› nematode;inflorescence morphogenesis;shoot development;calcium ion transmembrane

ation;pollen tube growth
sis;chlorophyll biosynthetic process

›endent

ignaling;sugar mediated signaling pathway;glucose mediated signaling pathway;programm

ndle assembly

diated signaling;seed germination;protein amino acid autophosphorylation

›acylation;ovule development

›f pH;cellular potassium ion homeostasis;water homeostasis;transmembrane transport

Do not distribute

ɪendent

ion;cellular manganese ion homeostasis;response to cadmium ion

activity

e;systemic acquired resistance;response to salicylic acid stimulus;defense response signalin

ɔnal initiation;regulation of translational initiation

;;ubiquitin-dependent protein catabolic process;response to cold;response to cadmium ion

ansport;positive regulation of Rho GTPase activity;Golgi vesicle transport
nse to auxin stimulus;GTP catabolic process
ay;xylem and phloem pattern formation;primary shoot apical meristem specification

ɔrotein translation;valyl-tRNA aminoacylation;regulation of translational fidelity;chloroplas
tion;regulation of translational fidelity;embryonic development ending in seed dormancy
aminoacylation;regulation of translational fidelity
tion;regulation of translational fidelity;embryonic development ending in seed dormancy

gene expression;regulation of flower development;detection of temperature stimulus;flow

ive regulation of flower development;photoperiodism, flowering;response to salt stress
tRNA aminoacylation;tRNA aminoacylation;ovule development

Do not distribute

tRNA aminoacylation;tRNA aminoacylation;ovule development

ss;Golgi vesicle transport

DNA-dependent;protein targeting to membrane;cell communication;response to cold;resp
rt, Golgi to ER;vesicle-mediated transport;transport;protein transport
etic process, mevalonate-independent pathway
entose-phosphate shunt;defense response to bacterium;cellular response to redox state

endent;chromatin modification

n peptidyl-prolyl isomerization;regionalization;DNA methylation;DNA recombination;mitoti

ndent decapping of nuclear-transcribed mRNA;root meristem specification;cellular respons

e to cold;response to cadmium ion;proteolysis involved in cellular protein catabolic process

nt DNA replication;DNA repair;nucleotide metabolic process;2'-deoxyribonucleotide metab

ocessing;methionine biosynthetic process;cellulose biosynthetic process;defense response t

ress;cell adhesion;cold acclimation;trichome morphogenesis;regulation of ethylene mediat
symbiotic fungus;response to light intensity;response to salt stress;salicylic acid biosynthe
rt from nucleus;protein import into nucleus;transport;methionine biosynthetic process;resp

gnaling pathway;proteasomal protein catabolic process;protein catabolic process

se to heat;PSII associated light-harvesting complex II catabolic process;glucosinolate biosyn
division;ATP catabolic process

Do not distribute

morphogenesis;root morphogenesis;maintenance of root meristem identity;protein catabc
folding;response to heat;response to high light intensity;PSII associated light-harvesting co

ein catabolic process;photosystem II repair
biquitin-dependent protein catabolic process;fatty acid beta-oxidation;ER to Golgi vesicle-m
gnaling pathway;proteasomal protein catabolic process;protein catabolic process
esis;root morphogenesis;maintenance of root meristem identity;protein catabolic process;

r protein signaling pathway;metabolic process;phosphorylation;sphingolipid biosynthetic p

deprivation;response to salt stress;response to abscisic acid stimulus

nscription, DNA-dependent;chromatin modification
on
ort;response to fungus;transmembrane transport

onal initiation;regulation of translational initiation;response to salt stress;response to cadm

smal development;chromatin modification;floral organ abscission;anatomical structure mc

onal initiation;regulation of translational initiation

lmium ion;oxidation reduction

process;response to cyclopentenone;methylation
onal initiation;regulation of translational initiation;response to salt stress;response to cadm

Do not distribute

dmium ion

on;actin filament bundle assembly;actin filament capping;glucose mediated signaling pathv

n;negative regulation of flower development;histone modification;cellulose biosynthetic p

ed dormancy;meristem structural organization;leaf morphogenesis;shoot morphogenesis;r

Do not distribute

ement;transport;ion transport;proton transport

igh light intensity;response to cadmium ion;protein ubiquitination

ement

ement

horylation
otic recombination;RNA processing;protein import into nucleus;cell-cell signaling;regulatio

nal protein catabolic process;response to cadmium ion;protein polymerization
ement

Do not distribute

lic process to D-lactate;heterochromatin formation;meiotic DNA double-strand break form

g in seed dormancy;negative gravitropism;response to starvation;cell division;response to

hotorespiration;positive gravitropism;auxin homeostasis;endosome transport;root develop

ort;regulation of chromosome organization;growth;defense response to bacterium

Do not distribute

um ion

process;cell wall modification;Golgi vesicle transport;protein amino acid glycosylation

orphogenesis;response to cytokinin stimulus;unidimensional cell growth;negative regulatio

stress

Do not distribute

tity;gene silencing by RNA;reproductive structure development;production of lsiRNA involv
organ identity;indeterminate inflorescence morphogenesis;ovule development

change;organ senescence;sugar mediated signaling pathway;abscisic acid mediated signalir

se response signaling pathway, resistance gene-dependent;leaf morphogenesis;leaf senesc

transport;cellulose biosynthetic process;Golgi vesicle transport

horylation;endomembrane system organization

rogen peroxide metabolic process;photosynthesis

tion

intenance of floral organ identity

Do not distribute

Do not distribute

eleton organization;metabolic process;cellular amino acid biosynthetic process;proteasomal
oxification of nitrogen compound

ytokinin stimulus;root development;primary shoot apical meristem specification;meristem

ta-oxidation;mitosis;synapsis;reciprocal meiotic recombination;gravitropism;organ morphc
upled proton transport

development;endosperm development

opism;response to abscisic acid stimulus;RNA metabolic process

onse

hase transition of meristem;protein desumoylation;cell wall biogenesis;cell wall macromole

Do not distribute

import into chloroplast stroma

nt

of seed germination;negative regulation of transcription, DNA-dependent

oxification of nitrogen compound

mulus;epidermal cell differentiation;cuticle development

n;root hair initiation;root hair elongation;cotyledon development;phyllome development

Do not distribute

me morphogenesis;vegetative to reproductive phase transition of meristem;glucuronoxylan

nulus;response to water deprivation

ation;ubiquitin-dependent protein catabolic process
ling requiring cofactor

ancy;response to endoplasmic reticulum stress;protein refolding;response to hydrogen per

ding;protein refolding

e transition of meristem;histone H2B ubiquitination

tomorphogenesis;gravitropism;root development;somatic embryogenesis

ation;ubiquitin-dependent protein catabolic process

c acid stimulus;sphingolipid metabolic process

Do not distribute

:hway;defense response to bacterium

Do not distribute

ɔ abscisic acid stimulus;response to water deprivation

ular carbohydrate metabolic process;response to cadmium ion;oxidation reduction

on;floral organ development
;vegetative phase change;production of ta-siRNAs involved in RNA interference;RNA interfe

wering

cid phosphorylation;photomorphogenesis;cullin deneddylation;phosphorylation;protein ub

ı, DNA-dependent;response to DNA damage stimulus

ɫ signaling pathway;response to jasmonic acid stimulus;lateral root morphogenesis;negativ‹

ıse to cold

ular carbohydrate metabolic process;response to cadmium ion;oxidation reduction

ion

Do not distribute

ıtion;embryonic development ending in seed dormancy
ːocess;protein polymerization

ːmbryonic development ending in seed dormancy;seed germination;regulation of flower de
ne assembly;protein insertion into ER membrane;response to misfolded protein

to fructose stimulus;regulation of anion channel activity;amino acid import;oxidation reduc
ım ion;oxidation reduction;response to cold;response to water deprivation;response to ab

Do not distribute

ıctose stimulus;chlorophyll catabolic process;protein import into peroxisome matrix;oxidat

ınse to far red light;RNA interference;posttranscriptional gene silencing;gene silencing by m

DNA-dependent;response to DNA damage stimulus;sister chromatid cohesion;synapsis;re

development;endosperm development
ːle

ion of catalytic activity
ːignaling pathway;RNA interference;defense response to bacterium;cotyledon developmen

rmation;sepal formation

ion;regulation of carbohydrate metabolic process;negative regulation of catalytic activity

l stimulus;oxidation reduction

imulus;response to gibberellin stimulus;response to salicylic acid stimulus;response to jasm
ment
of transcription factor activity

ress;flower development

ant-type hypersensitive response;regulation of protein localization;cell fate commitment;re

Do not distribute

development;endosperm development

Do not distribute

ıction

process;response to cadmium ion;oxidation reduction

silencing by miRNA

ɡulation of nitrogen compound metabolic process;oxidation reduction
ic process;response to cadmium ion

l stimulus;oxidation reduction

leaf morphogenesis;chloroplast organization

wer development;root morphogenesis;cullin deneddylation;protein ubiquitination;histone

nse to far red light;RNA interference;posttranscriptional gene silencing;gene silencing by m

ponse to osmotic stress;response to cold;response to salt stress;response to UV-B;respons
velopment

;auxin polar transport;positive gravitropism;phosphorylation;root development;shoot deve

Do not distribute

Do not distribute

ate biosynthetic process;phosphorylation

ım;negative regulation of programmed cell death;anion homeostasis;transport;ion transpo

:;positive gravitropism;maintenance of root meristem identity;phloem or xylem histogenesi
cification of floral organ number;cell fate specification;specification of floral organ identity;
:ell division;response to cold;pollen development;embryonic development ending in seed d

l cell death;anion homeostasis;calcium ion transmembrane transport

tion

nsitive response;ammonium transport;basic amino acid transport;phospholipid transport;v

se to cadmium ion;regulation of protein amino acid dephosphorylation

tone deacetylation;cotyledon development;histone H3-K4 methylation;oxidation reduction

esponse to sulfate starvation;cellular response to water deprivation

Do not distribute

metry;organ morphogenesis;meristem initiation;vernalization response;vegetative phase c
ght;post-embryonic development;protein exit from endoplasmic reticulum;Golgi vesicle tra

monic acid mediated signaling pathway;induced systemic resistance, ethylene mediated si
uction;phosphorylation

lic process

Do not distribute

tion, DNA-dependent
iogenesis;cellular metabolic process

:o phosphate starvation

ɔiogenesis

ıtion;ovule development

iogenesis;cellular metabolic process

Do not distribute

n monoubiquitination;sister chromatid cohesion;cell adhesion;regulation of mitotic cell cyc

e;production of miRNAs involved in gene silencing by miRNA
n of meristem;positive regulation of organelle organization;protein desumoylation;regulati

ve regulation of cell division

nse to far red light;RNA interference;posttranscriptional gene silencing;gene silencing by R

uction;phosphorylation

nse to far red light;RNA interference;posttranscriptional gene silencing;gene silencing by R

nding in seed dormancy;chorismate metabolic process;folic acid biosynthetic process

rocessing;cytokinin mediated signaling pathway;gibberellic acid mediated signaling pathwa

cess;nitrogen fixation;indoleacetic acid biosynthetic process;response to abscisic acid stimu

ponse to auxin stimulus;regulation of cell division;trichome morphogenesis;positive regulat

Do not distribute

Do not distribute

pathway;response to salt stress

membrane transport;transport;ion transport;zinc ion transport

rand break formation;meiotic chromosome segregation

defense response to fungus;response to abscisic acid stimulus

t development

ess;chaperone-mediated protein complex assembly;oxidation-dependent protein catabolic

esponse to high light intensity

Do not distribute

se to cadmium ion
xidation reduction

development;response to gamma radiation;glucuronoxylan metabolic process;positive regu

rphogenesis;response to auxin stimulus;embryonic development ending in seed dormancy;
: to water deprivation

:ell proliferation;response to gamma radiation;glucuronoxylan metabolic process;chromatin

ned cell death;phosphorylation;hexose catabolic process;glucose 6-phosphate metabolic pr

Do not distribute

ɜe to hydrogen peroxide

n metabolic process;cellular amino acid biosynthetic process;aromatic amino acid family m

pment ending in seed dormancy;seed germination;embryonic pattern specification;regulati

omeostasis;oxidation reduction

ormation;root development;leaf development;shoot development;post-embryonic root de

nent ending in seed dormancy;thylakoid membrane organization;vegetative to reproductiv
phorylation

ermination;cell division
;isopentenyl diphosphate biosynthetic process, mevalonate-independent pathway;glucosir

on of meristem;regulation of anion channel activity;glucuronoxylan metabolic process;posit

ication;DNA methylation;cell proliferation;regulation of flower development;regulation of

d dormancy;defense response to bacterium;reductive pentose-phosphate cycle

rt;nucleotide transport;cellular zinc ion homeostasis;intracellular protein transport;ER to G

Do not distribute

pendent systemic resistance, ethylene mediated signaling pathway;cell wall macromolecul

ister chromatid cohesion;cell adhesion;embryonic development ending in seed dormancy;u

nsport;vesicle-mediated transport

Do not distribute

cess

ɔonse to UV light;carpel development

rocess

ɔgression;post-translational protein modification;positive regulation of transcription, DNA-ɑ

Do not distribute

ɔsynthetic process;isopentenyl diphosphate biosynthetic process, mevalonate-independen

ic acid metabolic process;cellular response to hydrogen peroxide;cellular response to absci

sition in cell wall during defense response;leaf senescence;cellular response to iron ion;regu

;L-proline biosynthetic process

vater deprivation;defense response to bacterium;para-aminobenzoic acid metabolic proces

Do not distribute

hetic process;cutin biosynthetic process;lateral root formation;defense response to fungus

process;embryonic development ending in seed dormancy;coumarin biosynthetic process;

ɛduction

transport

ned cell death;phosphorylation;hexose catabolic process;glucose 6-phosphate metabolic pr[illegible]

Do not distribute

ng pathway, resistance gene-independent;carbohydrate biosynthetic process;response to e

Do not distribute

t organization;embryonic development ending in seed dormancy;thylakoid membrane orga

ver development;defense response to bacterium

ponse to bacterium;response to ethylene stimulus;response to abscisic acid stimulus;abscis

ic recombination;chromatin organization;regulation of transcription, DNA-dependent;RNA

se to auxin stimulus;post-embryonic development;primary shoot apical meristem specificat

;

olic process

to bacterium;Golgi vesicle transport;phosphorylation

ted signaling pathway;vegetative to reproductive phase transition of meristem;protein desu
etic process;response to microbial phytotoxin;regulation of hydrogen peroxide metabolic pr
ponse to auxin stimulus;defense response signaling pathway, resistance gene-dependent;re

thetic process;regulation of protein localization

Do not distribute

‌olic process;meristem growth;proteasomal ubiquitin-dependent protein catabolic process;p
‌omplex II catabolic process;response to hydrogen peroxide

‌nediated transport;cytoskeleton organization;toxin catabolic process;response to cytokinin

meristem growth;proteasomal ubiquitin-dependent protein catabolic process;proteasome

‌rocess

‌ium ion

‌orphogenesis;embryonic development ending in seed dormancy

‌ium ion

Do not distribute

Do not distribute

way;response to cadmium ion

rocess;production of siRNA involved in RNA interference;production of miRNAs involved in

regulation of meristem growth;stem cell maintenance;flower morphogenesis;organ format

Do not distribute

n of mitotic cell cycle;embryo sac egg cell differentiation;virus induced gene silencing;phot[illegible]

ıation;positive regulation of cell cycle;floral organ formation

stress;phototropism;embryonic axis specification;late endosome to vacuole transport

pment;response to misfolded protein;proteasome core complex assembly

Do not distribute

n of transcription, DNA-dependent;response to auxin stimulus;root development;cell prolif

Do not distribute

ved in RNA interference;maintenance of DNA methylation;defense response to virus;produ

ng pathway

:ence;defense response to bacterium;callose deposition during defense response;callose de

Do not distribute

Do not distribute

l protein catabolic process;'de novo' UMP biosynthetic process;histone H3-K9 methylation;

structural organization;embryonic development ending in seed dormancy

ogenesis;tissue development;trichome morphogenesis;regulation of cell cycle process;posi

Do not distribute

ecule metabolic process;actin nucleation;root hair cell differentiation;hydrogen peroxide bi

Do not distribute

n metabolic process;positive regulation of organelle organization;cell growth;protein desu

roxide;cellular protein metabolic process

Do not distribute

Do not distribute

erence;chromatin silencing by small RNA;production of miRNAs involved in gene silencing k

›iquitination;histone methylation;histone phosphorylation;protein deubiquitination;histone

e regulation of transcription, DNA-dependent

Do not distribute

evelopment;meristem structural organization;maintenance of meristem identity;seed dorm

ction;response to cold;response to water deprivation;response to abscisic acid stimulus;D-g
scisic acid stimulus;D-gluconate metabolic process

tion reduction

niRNA;innate immune response;adventitious root development;gene silencing by RNA

ciprocal meiotic recombination;male meiosis;male meiosis I;pyrimidine ribonucleotide bios

nt;long-day photoperiodism;salicylic acid mediated signaling pathway;jasmonic acid mediat

Do not distribute

Do not distribute

nonic acid stimulus;response to chitin;response to cadmium ion

esponse to ethylene stimulus;response to auxin stimulus

Do not distribute

: methylation;histone acetylation;protein deubiquitination;post-translational protein modif

ıiRNA;innate immune response;adventitious root development;gene silencing by RNA

e to UV-C;stomatal complex development;phosphorylation;dephosphorylation;peptidyl-tyr

elopment;root hair initiation;root hair elongation;cotyledon development;phyllome develo

Do not distribute

Do not distribute

ɔrt;calcium ion transport

is;cotyledon vascular tissue pattern formation;root development;leaf development;gynoec
;ovule development
ɫormancy;vernalization response;stomatal lineage progression;regulation of meiosis;DNA e

vesicle-mediated transport;negative regulation of programmed cell death;amino acid impor

ı;chromatin modification

Do not distribute

change;xylem and phloem pattern formation;meristem maintenance;production of ta-siRNA
ansport;vesicle fusion with Golgi apparatus

gnaling pathway

Do not distribute

Do not distribute

:le;photomorphogenesis;embryonic development ending in seed dormancy;defense respor

on of chromosome organization;actin nucleation;hydrogen peroxide biosynthetic process

.NA;gene silencing by miRNA;innate immune response;adventitious root development

.NA;gene silencing by miRNA;innate immune response;adventitious root development

ıy;negative regulation of gibberellic acid mediated signaling pathway;RNA interference;pos

ılus;response to chitin;lateral root formation;root cap development;response to bacterium

:ion of transcription, DNA-dependent

Do not distribute

Do not distribute

process

Do not distribute

Do not distribute

ulation of organelle organization;protein desumoylation;regulation of telomere maintenan

;auxin polar transport;positive gravitropism;vernalization response;trichome morphogenes

n silencing by small RNA;regulation of telomere maintenance;multicellular organism reproc

ocess

ıetabolic process;aromatic amino acid family biosynthetic process;lipoate metabolic proces

ion of flower development;meristem structural organization;primary shoot apical meristen

ɛvelopment

ve phase transition of meristem;iron-sulfur cluster assembly;ovule development

nolate metabolic process;positive regulation of catalytic activity

tive regulation of organelle organization;protein desumoylation;chromatin silencing by sma

G2/M transition of mitotic cell cycle;gene silencing;histone lysine methylation;histone H3-K

iolgi vesicle-mediated transport;protein secretion;response to nematode;regulation of plar

Do not distribute

le catabolic process;response to cadmium ion;defense response to fungus

unidimensional cell growth;embryonic pattern specification;organ morphogenesis;tissue de

Do not distribute

dependent;photoperiodism, flowering;regulation of cell cycle;cell cycle;cell division

Do not distribute

ıt pathway;ncRNA metabolic process;hydrogen peroxide catabolic process;response to cadı

isic acid stimulus;cellular hyperosmotic salinity response;indolebutyric acid metabolic proc

ulation of stomatal movement;auxin polar transport;response to heat;negative regulation

ss;callose deposition in cell wall during defense response;cellular response to hydrogen per

Do not distribute

;

sterol biosynthetic process;brassinosteroid biosynthetic process;pentacyclic triterpenoid b

Do not distribute

ocess

endoplasmic reticulum stress;anthocyanin metabolic process

Do not distribute

anization;stomatal complex morphogenesis;vegetative to reproductive phase transition of

sic acid mediated signaling pathway;negative regulation of abscisic acid mediated signaling

processing;mRNA export from nucleus;protein folding;protein import into nucleus;meiosis

tion

umoylation;circadian regulation of gene expression;actin nucleation;regulation of long-day
rocess;defense response to bacterium
egulation of flower development;negative regulation of flower development;maintenance

Do not distribute

proteasome assembly;response to cadmium ion;root development;shoot development;phy

stimulus;photorespiration;proteasomal protein catabolic process;amino acid import;prote

assembly;response to cadmium ion;root development;shoot development;phyllome devel

Do not distribute

Do not distribute

ı gene silencing by miRNA;post-translational protein modification;positive regulation of traı

:ion

omorphogenesis;embryonic development ending in seed dormancy;seed germination;regu

Do not distribute

Do not distribute

feration;fruit septum development;reproductive developmental process;floral meristem de

Do not distribute

ction of siRNA involved in RNA interference;vegetative phase change;virus induced gene sil

eposition in cell wall during defense response;defense response to fungus

Do not distribute

Do not distribute

;cellular response to phosphate starvation

Do not distribute

tive regulation of organelle organization;protein import into peroxisome matrix;regulation

iosynthetic process;cell wall organization

Do not distribute

moylation;regulation of chromosome organization;actin nucleation;xylan biosynthetic proc

Do not distribute

Do not distribute

by miRNA;histone H3-K9 methylation

e H3-S10 phosphorylation;histone H3-S28 phosphorylation;positive regulation of transcript

Do not distribute

nancy;sugar mediated signaling pathway;positive regulation of vernalization response;vege

gluconate metabolic process

Do not distribute

synthetic process;pollen development;embryo sac egg cell differentiation;photomorphoger

ed signaling pathway;response to wounding

Do not distribute

Do not distribute

fication;histone H3 acetylation;positive regulation of transcription, DNA-dependent;positive

Do not distribute

rosine dephosphorylation;amino acid import;ovule development

pment

Do not distribute

:ium development;cotyledon development;primary root development;shoot development;

:ndoreduplication;negative regulation of cyclin-dependent protein kinase activity;gametopl

rt;regulation of ion transport;Golgi vesicle transport;Golgi vesicle budding;root developme

Do not distribute

As involved in RNA interference;RNA interference;production of miRNAs involved in gene s

Do not distribute

Do not distribute

ıse to fungus, incompatible interaction;seed germination;embryonic pattern specification;ı

Do not distribute

:t-translational protein modification;positive regulation of transcription, DNA-dependent;pr

ı

Do not distribute

Do not distribute

Do not distribute

ıce;multicellular organism reproduction;regulation of chromosome organization;meiotic DN

sis;vegetative to reproductive phase transition of meristem;auxin efflux;anthocyanin accum

duction;regulation of chromosome organization;meiotic DNA double-strand break formatic

ɜs;lipoate biosynthetic process;coenzyme biosynthetic process;nucleotide metabolic proces

n specification;seed dormancy;sugar mediated signaling pathway;vegetative to reproductiv

Do not distribute

all RNA;regulation of chromosome organization;meiotic DNA double-strand break formatio

K9 methylation;regulation of cell cycle

ɿt-type hypersensitive response;ammonium transport;basic amino acid transport;negative

evelopment;longitudinal axis specification;meristem initiation;primary shoot apical meriste

Do not distribute

Do not distribute

mium ion

ess

of defense response;root hair cell differentiation;defense response to bacterium;response

roxide;cellular response to abscisic acid stimulus;cellular hyperosmotic salinity response;inc

Do not distribute

iosynthetic process;methylation;cellular amino acid derivative biosynthetic process;lipid m

Do not distribute

Do not distribute

meristem;iron-sulfur cluster assembly;mRNA modification;protein targeting to chloroplast;

pathway;defense response to fungus, incompatible interaction;seed germination;systemic

Do not distribute

;;reciprocal meiotic recombination;cell adhesion;embryo sac egg cell differentiation;photor

photoperiodism, flowering;root hair cell differentiation;hydrogen peroxide biosynthetic pr

of meristem identity

yllome development;root cap development;response to misfolded protein

easomal ubiquitin-dependent protein catabolic process;proteasome assembly;root hair elon

lopment;root cap development;response to misfolded protein;female gamete generation;r

Do not distribute

Do not distribute

nscription, DNA-dependent;Golgi vesicle transport;photoperiodism, flowering;chromosom

Do not distribute

ulation of flower development;meristem structural organization;vernalization response;see

Do not distribute

eterminacy;gynoecium development;meristem development;auxin mediated signaling path

Do not distribute

lencing;transcription termination

Do not distribute

Do not distribute

of chromosome organization;attachment of spindle microtubules to kinetochore during m

Do not distribute

Do not distribute

:ess;Golgi vesicle transport;root hair cell differentiation;hydrogen peroxide biosynthetic prc

Do not distribute

Do not distribute

tion, DNA-dependent;histone H3-K9 methylation

Do not distribute

etative to reproductive phase transition of meristem;protein ubiquitination;histone modific

Do not distribute

nesis;embryonic development ending in seed dormancy;seed germination;regulation of flov

Do not distribute

Do not distribute

e regulation of cellular process;transcription, DNA-dependent;chromatin modification

Do not distribute

Do not distribute

;defense response to bacterium

hyte development;phosphorylation

nt;shoot development;lateral root development

Do not distribute

silencing by miRNA;flower morphogenesis;negative regulation of biological process

Do not distribute

Do not distribute

regulation of flower development;meristem structural organization;leaf morphogenesis;me

Do not distribute

rotein amino acid glycosylation

Do not distribute

Do not distribute

Do not distribute

\NA double-strand break formation;telomere maintenance in response to DNA damage;meio

nulation in tissues in response to UV light;actin nucleation;root development;carpel develo

on;telomere maintenance in response to DNA damage;meiotic chromosome segregation;xy

ss;protein lipoylation;jasmonic acid biosynthetic process;leaf morphogenesis;chlorophyll bi

ve phase transition of meristem;seed maturation;regulation of cell cycle process;protein ub

Do not distribute

on;meiotic chromosome segregation;xylan biosynthetic process;hydrogen peroxide biosynt

regulation of programmed cell death;amino acid import;regulation of ion transport

em specification;phloem or xylem histogenesis;trichome morphogenesis;vegetative to repr

Do not distribute

Do not distribute

to salt stress;defense response to fungus;response to molecule of bacterial origin;respons[illegible]

dolebutyric acid metabolic process;auxin metabolic process

Do not distribute

ıetabolic process;steroid metabolic process

Do not distribute

Do not distribute

;ovule development

: acquired resistance, salicylic acid mediated signaling pathway;salicylic acid mediated signa

Do not distribute

norphogenesis;embryonic development ending in seed dormancy;seed germination;deterr

rocess;cell wall organization

ngation;response to misfolded protein;proteasome core complex assembly

male gamete generation;regulation of gene silencing;lateral root formation

Do not distribute

Do not distribute

e organization

Do not distribute

ɛd dormancy;sugar mediated signaling pathway;vegetative to reproductive phase transitior

Do not distribute

ıway

Do not distribute

Do not distribute

Do not distribute

ıeiosis I;meiotic sister chromatid cohesion, centromeric

Do not distribute

Do not distribute

ocess;copper ion homeostasis;cell wall organization;transcription, DNA-dependent

Do not distribute

Do not distribute

Do not distribute

:ation;lipid storage;floral organ formation;response to freezing;methylation;histone arginin

Do not distribute

wer development;meristem structural organization;seed dormancy;sugar mediated signali

Do not distribute

Do not distribute

Do not distribute

Do not distribute

Do not distribute

Do not distribute

Do not distribute

eristem initiation;primary shoot apical meristem specification;maintenance of meristem id

Do not distribute

Do not distribute

Do not distribute

otic chromosome segregation;xylan biosynthetic process

Do not distribute

ɔpment;stamen development;root hair cell differentiation;cell wall organization;basipetal a

ylan biosynthetic process;positive regulation of transcription, DNA-dependent

osynthetic process;isopentenyl diphosphate biosynthetic process, mevalonate-independen

oiquitination;lipid storage;regulation of cell differentiation;leaf development;cotyledon dev

Do not distribute

:hetic process

oductive phase transition of meristem;lateral root formation;seed maturation;proteasomal

Do not distribute

Do not distribute

e to osmotic stress

Do not distribute

Do not distribute

Do not distribute

aling pathway;jasmonic acid mediated signaling pathway;organ morphogenesis;response tc

mination of bilateral symmetry;organ morphogenesis;tissue development;regulation of flov

Do not distribute

Do not distribute

Do not distribute

Do not distribute

ı of meristem;production of ta-siRNAs involved in RNA interference;telomere capping;prot[illegible]

Do not distribute

Do not distribute

Do not distribute

Do not distribute

Do not distribute

Do not distribute

Do not distribute

Do not distribute

Do not distribute

ıe methylation

Do not distribute

ing pathway;vegetative to reproductive phase transition of meristem;response to gamma ra

Do not distribute

Do not distribute

Do not distribute

Do not distribute

Do not distribute

Do not distribute

Do not distribute

entity;trichome morphogenesis;seed dormancy;sugar mediated signaling pathway;vegetat

Do not distribute

Do not distribute

Do not distribute

Do not distribute

uxin transport;acropetal auxin transport;auxin influx;response to nematode

ıt pathway;cysteine biosynthetic process;secondary metabolic process;cell differentiation;s

/elopment;response to freezing;cell division

Do not distribute

l protein catabolic process;basipetal auxin transport;positive regulation of organelle organi

Do not distribute

Do not distribute

Do not distribute

Do not distribute

Do not distribute

ɔ extracellular stimulus;fruit development;response to chitin;regulation of hydrogen peroxi

Do not distribute

wer development;meristem structural organization;meristem initiation;meristem maintena

Do not distribute

Do not distribute

Do not distribute

ein ubiquitination;lipid storage;production of miRNAs involved in gene silencing by miRNA;

Do not distribute

Do not distribute

Do not distribute

Do not distribute

Do not distribute

Do not distribute

Do not distribute

Do not distribute

Do not distribute

Do not distribute

radiation;somatic cell DNA recombination;protein ubiquitination;lipid storage;regulation of

Do not distribute

Do not distribute

Do not distribute

Do not distribute

Do not distribute

Do not distribute

Do not distribute

:ive to reproductive phase transition of meristem;cullin deneddylation;regulation of G2/M t

Do not distribute

Do not distribute

Do not distribute

Do not distribute

sulfur compound biosynthetic process;positive regulation of transcription, DNA-dependent

Do not distribute

ization;cell growth;vesicle-mediated transport;cellulose biosynthetic process;regulation of

Do not distribute

Do not distribute

Do not distribute

Do not distribute

Do not distribute

ide metabolic process;regulation of plant-type hypersensitive response;endoplasmic reticu

Do not distribute

ance;maintenance of meristem identity;regulation of root meristem growth;trichome morp

Do not distribute

Do not distribute

Do not distribute

;response to freezing

Do not distribute

Do not distribute

Do not distribute

Do not distribute

Do not distribute

Do not distribute

Do not distribute

Do not distribute

Do not distribute

Do not distribute

telomere maintenance;multicellular organism reproduction;meiotic DNA double-strand br

Do not distribute

Do not distribute

Do not distribute

Do not distribute

Do not distribute

Do not distribute

Do not distribute

transition of mitotic cell cycle;histone monoubiquitination;glucuronoxylan metabolic proce

Do not distribute

Do not distribute

Do not distribute

Do not distribute

:

Do not distribute

ARF protein signal transduction;endosome transport via multivesicular body sorting pathw

Do not distribute

Do not distribute

Do not distribute

Do not distribute

Do not distribute

ılum unfolded protein response;negative regulation of defense response;regulation of prot[illegible]

ɔhogenesis;seed dormancy;sugar mediated signaling pathway;vegetative to reproductive p

Do not distribute

Do not distribute

Do not distribute

Do not distribute

Do not distribute

Do not distribute

Do not distribute

Do not distribute

Do not distribute

Do not distribute

Do not distribute

Do not distribute

Do not distribute

Do not distribute

reak formation;telomere maintenance in response to DNA damage;post-translational prote

Do not distribute

Do not distribute

Do not distribute

Do not distribute

Do not distribute

Do not distribute

Do not distribute

ess;seed maturation;regulation of cell cycle process;cell growth;protein ubiquitination;histo

Do not distribute

Do not distribute

Do not distribute

Do not distribute

Do not distribute

/ay;regulation of organelle organization;regulation of chromosome organization;actin nucle

Do not distribute

Do not distribute

Do not distribute

Do not distribute

Do not distribute

tein amino acid dephosphorylation;hydrogen peroxide catabolic process;negative regulatior

Do not distribute

phase transition of meristem;leaf vascular tissue pattern formation;response to gamma radi

Do not distribute

Do not distribute

Do not distribute

Do not distribute

Do not distribute

Do not distribute

Do not distribute

Do not distribute

Do not distribute

Do not distribute

Do not distribute

Do not distribute

Do not distribute

ein modification;meiotic chromosome segregation;positive regulation of transcription, DNA

Do not distribute

Do not distribute

Do not distribute

Do not distribute

Do not distribute

Do not distribute

Do not distribute

one methylation;protein deubiquitination;protein desumoylation;lipid storage;regulation of

Do not distribute

Do not distribute

Do not distribute

Do not distribute

Do not distribute

eation;regulation of cell differentiation;Golgi vesicle transport;regulation of vesicle targetin

Do not distribute

Do not distribute

Do not distribute

Do not distribute

Do not distribute

n of programmed cell death;Golgi vesicle transport;root development;lateral root developr

iation;leaf formation;leaf shaping;cullin deneddylation;positive regulation of organelle orga

Do not distribute

Do not distribute

Do not distribute

Do not distribute

Do not distribute

Do not distribute

Do not distribute

Do not distribute

Do not distribute

Do not distribute

Do not distribute

Do not distribute

Do not distribute

Do not distribute

\-dependent;male gamete generation;response to freezing;chiasma assembly

Do not distribute

Do not distribute

Do not distribute

Do not distribute

Do not distribute

Do not distribute

Do not distribute

f organelle organization;histone H2B ubiquitination;actin nucleation;xylan biosynthetic pro

Do not distribute

Do not distribute

Do not distribute

Do not distribute

Do not distribute

ıg, to, from or within Golgi;floral organ formation;developmental growth;root hair cell diffe

Do not distribute

Do not distribute

Do not distribute

Do not distribute

Do not distribute

ment;defense response to fungus;response to other organism;signal transduction;G-protei

anization;protein ubiquitination;histone modification;histone methylation;protein deubiqu

Do not distribute

Do not distribute

Do not distribute

Do not distribute

Do not distribute

Do not distribute

Do not distribute

Do not distribute

Do not distribute

Do not distribute

Do not distribute

Do not distribute

Do not distribute

Do not distribute

Do not distribute

Do not distribute

Do not distribute

Do not distribute

Do not distribute

Do not distribute

Do not distribute

ocess;regulation of cell differentiation;positive regulation of transcription, DNA-dependent;

Do not distribute

Do not distribute

Do not distribute

Do not distribute

Do not distribute

erentiation;regulation of transport;cell division;cell wall organization;ER body organization

Do not distribute

Do not distribute

Do not distribute

Do not distribute

Do not distribute

n coupled receptor protein signaling pathway

ıitination;lipid storage;gene silencing by RNA;regulation of histone methylation;regulation (

Do not distribute

Do not distribute

Do not distribute

Do not distribute

Do not distribute

Do not distribute

Do not distribute

Do not distribute

Do not distribute

Do not distribute

Do not distribute

Do not distribute

Do not distribute

Do not distribute

Do not distribute

Do not distribute

Do not distribute

Do not distribute

Do not distribute

Do not distribute

Do not distribute

leaf development;floral organ formation;developmental growth;cotyledon development;h

Do not distribute

Do not distribute

Do not distribute

Do not distribute

Do not distribute

Do not distribute

Do not distribute

Do not distribute

Do not distribute

Do not distribute

Do not distribute

of telomere maintenance;regulation of organelle organization;regulation of chromosome c

Do not distribute

Do not distribute

Do not distribute

Do not distribute

Do not distribute

Do not distribute

Do not distribute

Do not distribute

Do not distribute

Do not distribute

Do not distribute

Do not distribute

Do not distribute

Do not distribute

Do not distribute

Do not distribute

Do not distribute

Do not distribute

Do not distribute

Do not distribute

ydrogen peroxide biosynthetic process;response to freezing;cell division;chromatin modific

Do not distribute

Do not distribute

Do not distribute

Do not distribute

Do not distribute

Do not distribute

Do not distribute

Do not distribute

Do not distribute

Do not distribute

organization;telomere maintenance in response to DNA damage;post-translational protein

Do not distribute

Do not distribute

Do not distribute

Do not distribute

Do not distribute

Do not distribute

Do not distribute

Do not distribute

Do not distribute

Do not distribute

Do not distribute

Do not distribute

Do not distribute

Do not distribute

Do not distribute

Do not distribute

Do not distribute

Do not distribute

Do not distribute

Do not distribute

Do not distribute

cation

Do not distribute

Do not distribute

Do not distribute

Do not distribute

Do not distribute

Do not distribute

Do not distribute

Do not distribute

Do not distribute

Do not distribute

Do not distribute

modification;actin nucleation;positive regulation of transcription, DNA-dependent;carpel c

Do not distribute

Do not distribute

Do not distribute

Do not distribute

Do not distribute

Do not distribute

Do not distribute

Do not distribute

Do not distribute

Do not distribute

Do not distribute

Do not distribute

Do not distribute

Do not distribute

Do not distribute

Do not distribute

Do not distribute

Do not distribute

Do not distribute

Do not distribute

Do not distribute

Do not distribute

Do not distribute

Do not distribute

Do not distribute

Do not distribute

Do not distribute

Do not distribute

Do not distribute

Do not distribute

development;stamen development;floral organ formation;petal formation;sepal formation;

Do not distribute

Do not distribute

Do not distribute

Do not distribute

Do not distribute

Do not distribute

Do not distribute

Do not distribute

Do not distribute

Do not distribute

Do not distribute

Do not distribute

Do not distribute

Do not distribute

Do not distribute

Do not distribute

Do not distribute

Do not distribute

Do not distribute

Do not distribute

Do not distribute

Do not distribute

Do not distribute

Do not distribute

Do not distribute

Do not distribute

Do not distribute

Do not distribute

Do not distribute

Do not distribute

Do not distribute

;root hair cell differentiation;response to freezing;histone H3-K9 methylation;cell wall orga

Do not distribute

Do not distribute

Do not distribute

Do not distribute

Do not distribute

Do not distribute

Do not distribute

Do not distribute

Do not distribute

Do not distribute

Do not distribute

Do not distribute

Do not distribute

Do not distribute

Do not distribute

Do not distribute

Do not distribute

Do not distribute

Do not distribute

Do not distribute

Do not distribute

Do not distribute

Do not distribute

Do not distribute

Do not distribute

Do not distribute

Do not distribute

Do not distribute

Do not distribute

Do not distribute

Do not distribute

ınization

Do not distribute

Supp. Table S18. Loci from the tropical japonica panel predicted to be involved in networ

[Rank]	[ORF_ID]	[Paralogs]	[Symbol]	[score]	[Evidences(	[#_linked_query/#_val
1	LOC_Os03g	no_paralog	Os03g0223	12.5	HS-LC:0.27	1/11
2	LOC_Os10g	no_paralog	Os10g0483	8.79	OS-CX:0.53	1/11
3	LOC_Os02g	no_paralog	Os02g0785	8.58	AT-CX:0.52	1/11
4	LOC_Os07g	LOC_Os03g	Os07g0184	8.29	OS-CX:0.53	1/11
5	LOC_Os02g	no_paralog	Os02g0478	8.15	AT-CX:0.57	1/11
6	LOC_Os08g	no_paralog	Os08g0117	8	AT-CX:0.57	1/11
7	LOC_Os04g	no_paralog	Os04g0501	7.94	AT-CX:0.53	1/11
8	LOC_Os03g	no_paralog	Os03g0598	7.7	AT-CX:0.54	1/11
9	LOC_Os03g	no_paralog	Os03g0851	7.7	AT-CX:0.45	2/11
10	LOC_Os10g	no_paralog	Os10g0564	7.51	AT-CX:0.58	1/11
11	LOC_Os08g	no_paralog	Os08g0326	7.46	AT-CX:0.58	1/11
12	LOC_Os08g	no_paralog	Os08g0109	7.42	SC-CC:0.45	1/11
13	LOC_Os10g	no_paralog	Os10g0563	7.1	AT-CX:0.44	2/11
14	LOC_Os01g	LOC_Os01g	Os01g0276	7.09	OS-CX:0.53	1/11
15	LOC_Os09g	no_paralog	Os09g0258	7.03	AT-CX:0.59	1/11
16	LOC_Os09g	no_paralog	Os09g0485	7.01	AT-CX:0.56	1/11
17	LOC_Os05g	LOC_Os03g	Os05g0346	7	AT-CX:0.53	1/11
18	LOC_Os07g	LOC_Os03g	Os07g0674	6.95	AT-CX:0.61	1/11
19	LOC_Os02g	no_paralog	Os02g0503	6.95	AT-CX:0.64	1/11
20	LOC_Os08g	LOC_Os04g	Os08g0234	6.89	OS-CX:0.52	1/11
21	LOC_Os03g	no_paralog	Os03g0234	6.82	AT-CX:0.67	1/11
22	LOC_Os03g	no_paralog	Os03g0576	6.81	AT-CX:0.63	1/11
23	LOC_Os02g	no_paralog	Os02g0791	6.8	SC-LC:0.39	1/11
24	LOC_Os03g	no_paralog	Os03g0297	6.76	AT-CX:0.61	1/11
25	LOC_Os02g	no_paralog	Os02g0821	6.7	AT-CX:0.66	1/11
26	LOC_Os02g	no_paralog	Os02g0622	6.63	AT-CX:0.69	1/11
27	LOC_Os01g	LOC_Os05g	Os01g0815	6.61	AT-CX:0.67	1/11
28	LOC_Os08g	no_paralog	Os08g0505	6.61	AT-CX:0.65	1/11
29	LOC_Os05g	no_paralog	Os05g0274	6.6	AT-CX:0.63	1/11
30	LOC_Os02g	no_paralog	Os02g0111	6.56	AT-CX:0.71	1/11
31	LOC_Os11g	no_paralog	Os11g0585	6.55	AT-CX:0.37	2/11
32	LOC_Os05g	no_paralog	Os05g0350	6.55	AT-CX:0.67	1/11
33	LOC_Os01g	LOC_Os01g	Os01g0666	6.52	AT-CX:0.56	1/11
34	LOC_Os05g	no_paralog	Os05g0314	6.47	OS-CX:0.51	1/11
35	LOC_Os11g	no_paralog	Os11g0433	6.45	AT-CX:0.61	1/11
36	LOC_Os05g	no_paralog	Os05g0145	6.44	AT-CX:0.51	1/11
37	LOC_Os03g	no_paralog	Os03g0337	6.43	AT-CX:0.60	1/11
38	LOC_Os03g	no_paralog	Os03g0626	6.41	OS-CX:0.68	1/11
39	LOC_Os01g	no_paralog	Os01g0821	6.41	AT-CX:0.60	1/11
40	LOC_Os05g	no_paralog	Os05g0155	6.38	AT-CX:0.51	1/11
41	LOC_Os07g	no_paralog	Os07g0150	6.33	AT-CX:0.74	1/11
42	LOC_Os08g	no_paralog	Os08g0156	6.33	AT-CX:0.72	1/11
43	LOC_Os01g	LOC_Os01g	Os01g0752	6.32	AT-CX:0.60	1/11
44	LOC_Os10g	LOC_Os03g	Os10g0170	6.3	AT-CX:0.64	1/11

Do not distribute

45	LOC_Os03gno_paralogOs03g0350	6.27	OS-CX:0.6[illegible]	1/11
46	LOC_Os08gno_paralogOs08g0558	6.25	AT-CX:0.71	1/11
47	LOC_Os06gLOC_Os02gOs06g0274	6.24	AT-CX:0.7[illegible]	1/11
48	LOC_Os05gno_paralogOs05g0541	6.23	AT-CX:0.61	1/11
49	LOC_Os03gLOC_Os07gOs03g0343	6.21	AT-CX:0.5[illegible]	1/11
50	LOC_Os07gLOC_Os03gOs07g0674	6.19	AT-CX:0.6[illegible]	1/11
51	LOC_Os09gno_paralogOs09g0568	6.18	OS-CX:0.5[illegible]	1/11
52	LOC_Os08gno_paralogOs08g0310	6.15	AT-CX:0.6[illegible]	1/11
53	LOC_Os02gno_paralogOs02g0822	6.15	SC-CC:0.54	1/11
54	LOC_Os01gno_paralogOs01g0896	6.11	AT-CX:0.6[illegible]	1/11
55	LOC_Os04gLOC_Os02gOs04g0473	6.1	AT-CX:0.6[illegible]	1/11
56	LOC_Os06gno_paralogOs06g0115	6.09	AT-CX:0.7[illegible]	1/11
57	LOC_Os04gLOC_Os08gOs04g0613	6.08	AT-CX:0.7[illegible]	1/11
58	LOC_Os10gLOC_Os02gOs10g0466	6.07	AT-CX:0.71	1/11
59	LOC_Os01gno_paralogOs01g0348	6.07	AT-CX:0.6[illegible]	1/11
60	LOC_Os02gno_paralogOs02g0103	6.06	AT-CX:0.6[illegible]	1/11
61	LOC_Os03gno_paralogOs03g0794	6.06	AT-CX:0.7[illegible]	1/11
62	LOC_Os02gno_paralogOs02g0321	6.05	AT-CX:0.7[illegible]	1/11
63	LOC_Os11gno_paralogOs11g0579	6.03	AT-CX:0.7[illegible]	1/11
64	LOC_Os07gLOC_Os03gOs07g0622	6.01	AT-CX:0.6[illegible]	1/11
65	LOC_Os03gLOC_Os07gOs03g0390	5.99	AT-CX:0.51	1/11
66	LOC_Os03gLOC_Os10gOs03g0139	5.98	AT-CX:0.7[illegible]	1/11
67	LOC_Os06gLOC_Os02gOs06g0172	5.96	AT-CX:0.7[illegible]	1/11
68	LOC_Os04gno_paralogOs04g0320	5.91	OS-CX:0.6[illegible]	1/11
69	LOC_Os02gno_paralogOs02g0175	5.86	OS-CX:0.7[illegible]	1/11
70	LOC_Os04gno_paralogOs04g0117	5.82	OS-CX:0.3[illegible]	2/11
71	LOC_Os05gLOC_Os01gOs05g0477	5.78	AT-CX:0.5[illegible]	1/11
72	LOC_Os02gLOC_Os04gOs02g0586	5.77	AT-CX:0.6[illegible]	1/11
73	LOC_Os02gno_paralogOs02g0284	5.72	AT-CX:0.7[illegible]	1/11
74	LOC_Os03gno_paralogOs03g0200	5.72	AT-CX:0.6[illegible]	1/11
75	LOC_Os03gno_paralogOs03g0756	5.71	AT-CX:0.61	1/11
76	LOC_Os01gno_paralogOs01g0140	5.7	AT-CX:0.6[illegible]	1/11
77	LOC_Os03gno_paralogOs03g0699	5.7	OS-CX:0.5[illegible]	1/11
78	LOC_Os03gno_paralogOs03g0207	5.69	AT-CX:0.6[illegible]	1/11
79	LOC_Os03gno_paralogOs03g0579	5.64	AT-CX:0.6[illegible]	1/11
80	LOC_Os03gno_paralogOs03g0109	5.63	AT-CX:0.6[illegible]	1/11
81	LOC_Os07gno_paralogOs07g0603	5.6	AT-CX:0.6[illegible]	1/11
82	LOC_Os02gno_paralogOs02g0287	5.59	AT-CX:0.6[illegible]	1/11
83	LOC_Os04gno_paralogOs04g0349	5.59	OS-CX:0.6[illegible]	1/11
84	LOC_Os03gLOC_Os07gOs03g0798	5.58	AT-CX:0.5[illegible]	1/11
85	LOC_Os08gno_paralogOs08g0559	5.58	AT-CX:0.5[illegible]	1/11
86	LOC_Os02gno_paralogOs02g0604	5.54	AT-CX:0.6[illegible]	1/11
87	LOC_Os04gLOC_Os02gOs04g0598	5.54	AT-CX:0.6[illegible]	1/11
88	LOC_Os03gLOC_Os07gOs03g0807	5.43	OS-CX:0.6[illegible]	1/11
89	LOC_Os07gno_paralogOs07g0662	5.39	AT-CX:0.5[illegible]	1/11
90	LOC_Os12gLOC_Os11gOs12g0124	5.35	OS-CX:0.6[illegible]	1/11

Do not distribute

91	LOC_Os02gLOC_Os04gOs02g0699	5.35	AT-CX:0.70	1/11
92	LOC_Os03gLOC_Os07gOs03g0366	5.35	AT-CX:0.64	1/11
93	LOC_Os03gLOC_Os07gOs03g0410	5.34	AT-CX:0.62	1/11
94	LOC_Os04gLOC_Os02gOs04g0430	5.34	AT-CX:0.67	1/11
95	LOC_Os02gno_paralogOs02g0158	5.31	AT-CX:0.70	1/11
96	LOC_Os11gno_paralogOs11g0602	5.28	OS-CX:0.58	1/11
97	LOC_Os07gno_paralogOs07g0673	5.24	OS-CX:0.66	1/11
98	LOC_Os10gno_paralogOs10g0411	5.18	AT-CX:0.58	1/11
99	LOC_Os12gLOC_Os11gOs12g0508	5.17	AT-CX:0.63	1/11
100	LOC_Os08gLOC_Os04gOs08g0207	5.16	AT-CX:0.68	1/11
101	LOC_Os01gno_paralogOs01g0723	5.15	AT-CX:0.63	1/11
102	LOC_Os06gLOC_Os02gOs06g0555	5.09	AT-CX:0.67	1/11
103	LOC_Os09gno_paralogOs09g0359	5.09	AT-CX:0.65	1/11
104	LOC_Os03gno_paralogOs03g0240	5.08	AT-CX:0.53	1/11
105	LOC_Os03gno_paralogOs03g0801	5.08	CE-CC:0.56	1/11
106	LOC_Os01gno_paralogOs01g0514	5.06	AT-CX:0.57	1/11
107	LOC_Os01gLOC_Os05gOs01g0938	5.04	AT-CX:0.62	1/11
108	LOC_Os01gno_paralogOs01g0814	5	OS-GN:0.5	2/11
109	LOC_Os03gLOC_Os07gOs03g0182	4.98	AT-CX:0.53	1/11
110	LOC_Os01gno_paralogOs01g0962	4.97	AT-CX:0.61	1/11
111	LOC_Os11gno_paralogOs11g0220	4.96	AT-CX:0.61	1/11
112	LOC_Os07gLOC_Os03gOs07g0636	4.86	OS-CX:0.51	1/11
113	LOC_Os07gLOC_Os03gOs07g0207	4.81	AT-CX:0.56	2/11
114	LOC_Os06gno_paralogOs06g0133	4.8	OS-CX:1.00	1/11
115	LOC_Os07gLOC_Os03gOs07g0641	4.79	AT-CX:0.58	1/11
116	LOC_Os01gno_paralogOs01g0227	4.75	CE-CC:0.70	1/11
117	LOC_Os01gno_paralogOs01g0551	4.75	AT-CX:0.60	1/11
118	LOC_Os07gno_paralogOs07g0615	4.71	OS-CX:1.00	1/11
119	LOC_Os04gno_paralogOs04g0502	4.69	AT-CX:0.53	1/11
120	LOC_Os09gLOC_Os08gOs09g0413	4.67	AT-CX:1.00	1/11
121	LOC_Os03gno_paralogOs03g0154	4.67	OS-CX:0.53	1/11
122	LOC_Os02gno_paralogOs02g0815	4.63	AT-CX:1.00	1/11
123	LOC_Os08gno_paralogOs08g0117	4.62	AT-CX:1.00	1/11
124	LOC_Os08gno_paralogOs08g0128	4.6	AT-CX:1.00	1/11
125	LOC_Os02gLOC_Os01gOs02g0235	4.58	AT-CX:1.00	1/11
126	LOC_Os11gLOC_Os12gOs11g0127	4.58	OS-CX:0.62	1/11
127	LOC_Os09gno_paralogOs09g0568	4.56	AT-CX:1.00	1/11
128	LOC_Os07gno_paralogOs07g0450	4.56	AT-CX:1.00	1/11
129	LOC_Os07gno_paralogOs07g0523	4.56	AT-CX:1.00	1/11
130	LOC_Os07gno_paralogOs07g0523	4.56	AT-CX:1.00	1/11
131	LOC_Os09gno_paralogOs09g0571	4.53	AT-CX:1.00	1/11
132	LOC_Os02gno_paralogOs02g0591	4.53	AT-CX:1.00	1/11
133	LOC_Os08gLOC_Os09gOs08g0436	4.51	AT-CX:1.00	1/11
134	LOC_Os12gno_paralogOs12g0634	4.5	OS-CX:0.60	1/11
135	LOC_Os01gLOC_Os05gOs01g0839	4.5	AT-CX:0.54	2/11
136	LOC_Os03gno_paralogOs03g0109	4.48	AT-CX:1.00	1/11

Do not distribute

137	LOC_Os05gno_paralogOs05g0556	4.47	AT-CX:1.0(	1/11
138	LOC_Os05gno_paralogOs05g0555	4.47	AT-CX:1.0(	1/11
139	LOC_Os05gLOC_Os01gOs05g0355	4.47	AT-CX:1.0(	1/11
140	LOC_Os01gLOC_Os05gOs01g0304	4.47	AT-CX:1.0(	1/11
141	LOC_Os10gno_paralogOs10g0561	4.47	OS-CX:1.0(	1/11
142	LOC_Os01gno_paralogOs01g0596	4.47	OS-CX:1.0(	1/11
143	LOC_Os07gno_paralogOs07g0296	4.46	OS-CX:1.0(	1/11
144	LOC_Os08gno_paralogOs08g0535	4.45	AT-CX:1.0(	1/11
145	LOC_Os02gno_paralogOs02g0162	4.45	AT-CX:1.0(	1/11
146	LOC_Os07gno_paralogOs07g0208	4.45	AT-CX:1.0(	1/11
147	LOC_Os07gno_paralogOs07g0173	4.44	AT-CX:1.0(	1/11
148	LOC_Os02gLOC_Os04gOs02g0682	4.43	AT-CX:1.0(	1/11
149	LOC_Os11gLOC_Os12gOs11g0168	4.42	OS-CX:0.51	1/11
150	LOC_Os06gLOC_Os01gOs06g0550	4.42	AT-CX:1.0(	1/11
151	LOC_Os05gLOC_Os01gOs05g0207	4.42	AT-CX:1.0(	1/11
152	LOC_Os03gno_paralogOs03g0586	4.41	OS-CX:0.5!	2/11
153	LOC_Os03gno_paralogOs03g0385	4.41	AT-CX:0.5(	2/11
154	LOC_Os05gLOC_Os01gOs05g0486	4.41	AT-CX:1.0(	1/11
155	LOC_Os12gno_paralogOs12g0166	4.39	AT-CX:0.61	1/11
156	LOC_Os05gno_paralogOs05g0176	4.39	AT-CX:1.0(	1/11
157	LOC_Os07gno_paralogOs07g0523	4.39	AT-CX:1.0(	1/11
158	LOC_Os02gno_paralogOs02g0618	4.39	AT-CX:1.0(	1/11
159	LOC_Os05gno_paralogOs05g0367	4.38	AT-CX:0.61	1/11
160	LOC_Os12gno_paralogOs12g0609	4.36	OS-CX:0.61	1/11
161	LOC_Os04gLOC_Os08gOs04g0641	4.36	OS-CX:0.5z	2/11
162	LOC_Os01gLOC_Os02gOs01g0205	4.35	AT-CX:1.0(	1/11
163	LOC_Os01gno_paralogOs01g0358	4.35	AT-CX:1.0(	1/11
164	LOC_Os02gno_paralogOs02g0105	4.35	AT-CX:1.0(	1/11
165	LOC_Os02gno_paralogOs02g0610	4.35	OS-CX:0.61	1/11
166	LOC_Os10gno_paralogOs10g0191	4.34	OS-CX:1.0(	1/11
167	LOC_Os05gno_paralogOs05g0147	4.33	OS-CX:0.57	1/11
168	LOC_Os02gLOC_Os06gOs02g0804	4.33	AT-CX:1.0(	1/11
169	LOC_Os06gno_paralogOs06g0701	4.33	AT-CX:1.0(	1/11
170	LOC_Os09gLOC_Os01gOs09g0507	4.33	AT-CX:1.0(	1/11
171	LOC_Os12gno_paralogOs12g0506	4.32	OS-CX:1.0(	1/11
172	LOC_Os02gno_paralogOs02g0186	4.32	OS-CX:0.5:	1/11
173	LOC_Os04gno_paralogOs04g0520	4.31	AT-CX:0.5z	1/11
174	LOC_Os02gLOC_Os10gOs02g0814	4.31	AT-CX:1.0(	1/11
175	LOC_Os10gno_paralogOs10g0411	4.31	AT-CX:1.0(	1/11
176	LOC_Os12gno_paralogOs12g0170	4.31	AT-CX:1.0(	1/11
177	LOC_Os12gno_paralogOs12g0571	4.28	AT-CX:1.0(	1/11
178	LOC_Os01gno_paralogOs01g0706	4.28	AT-CX:1.0(	1/11
179	LOC_Os02gno_paralogOs02g0796	4.26	AT-CX:1.0(	1/11
180	LOC_Os03gLOC_Os10gOs03g0249	4.24	AT-CX:1.0(	1/11
181	LOC_Os01gno_paralogOs01g0231	4.24	AT-CX:1.0(	1/11
182	LOC_Os11gLOC_Os12gOs11g0105	4.23	AT-CX:1.0(	1/11

Do not distribute

183	LOC_Os01g	no_paralog	Os01g0280	4.23	AT-CX:1.0(	1/11
184	LOC_Os11g	no_paralog	Os11g0512	4.2	OS-CX:1.0(	1/11
185	LOC_Os04g	no_paralog	Os04g0642	4.19	SC-LC:0.62	1/11
186	LOC_Os05g	LOC_Os01g	Os05g0460	4.18	AT-CX:0.51	2/11
187	LOC_Os02g	no_paralog	Os02g0487	4.18	OS-CX:1.0(	1/11
188	LOC_Os06g	no_paralog	Os06g0649	4.18	SC-LC:0.61	1/11
189	LOC_Os02g	no_paralog	Os02g0214	4.18	OS-CX:1.0(	1/11
190	LOC_Os03g	LOC_Os07g	Os03g0341	4.17	AT-CX:1.0(	1/11
191	LOC_Os03g	LOC_Os12g	Os03g0654	4.17	OS-CX:1.0(	1/11
192	LOC_Os12g	LOC_Os11g	Os12g0105	4.17	AT-CX:1.0(	1/11
193	LOC_Os02g	no_paralog	Os02g0252	4.16	OS-CX:1.0(	1/11
194	LOC_Os02g	no_paralog	Os02g0496	4.16	AT-CX:1.0(	1/11
195	LOC_Os02g	no_paralog	Os02g0242	4.15	OS-CX:1.0(	1/11
196	LOC_Os04g	LOC_Os02g	Os04g0429	4.14	OS-CX:1.0(	1/11
197	LOC_Os02g	LOC_Os06g	Os02g0728	4.11	AT-CX:1.0(	1/11
198	LOC_Os02g	no_paralog	Os02g0249	4.11	OS-CX:1.0(	1/11
199	LOC_Os03g	LOC_Os05g	Os03g0297	4.1	AT-CX:1.0(	1/11
200	LOC_Os06g	no_paralog	Os06g0116	4.08	SC-LC:0.65	1/11
201	LOC_Os03g	no_paralog	Os03g0168	4.06	OS-CX:1.0(	1/11
202	LOC_Os10g	LOC_Os04g	Os10g0492	4.06	OS-CX:1.0(	1/11
203	LOC_Os06g	no_paralog	Os06g0143	4.05	OS-CX:0.61	1/11
204	LOC_Os08g	no_paralog	Os08g0558	4.05	AT-CX:1.0(	1/11
205	LOC_Os06g	no_paralog	Os06g0664	4.05	AT-CX:1.0(	1/11
206	LOC_Os05g	LOC_Os01g	Os05g0459	4.05	AT-CX:1.0(	1/11
207	LOC_Os02g	no_paralog	Os02g0230	4.05	AT-CX:0.61	1/11
208	LOC_Os04g	no_paralog	Os04g0661	4.03	SC-LC:0.64	1/11
209	LOC_Os04g	LOC_Os02g	na	4.03	AT-CX:1.0(	1/11
210	LOC_Os10g	no_paralog	Os10g0464	4.02	OS-CX:1.0(	1/11
211	LOC_Os03g	no_paralog	Os03g0427	4.01	OS-CX:1.0(	1/11
212	LOC_Os08g	LOC_Os09g	Os08g0517	4	AT-CX:0.59	1/11
213	LOC_Os07g	no_paralog	Os07g0224	4	AT-CX:1.0(	1/11
214	LOC_Os11g	no_paralog	Os11g0657	3.99	AT-CX:1.0(	1/11
215	LOC_Os09g	LOC_Os08g	Os09g0331	3.99	OS-CX:1.0(	1/11
216	LOC_Os01g	no_paralog	Os01g0505	3.99	OS-CX:0.52	2/11
217	LOC_Os01g	no_paralog	Os01g0691	3.99	SC-LC:0.65	1/11
218	LOC_Os02g	no_paralog	Os02g0102	3.99	AT-CX:1.0(	1/11
219	LOC_Os02g	no_paralog	Os02g0249	3.98	OS-CX:1.0(	1/11
220	LOC_Os01g	no_paralog	Os01g0393	3.98	OS-CX:1.0(	1/11
221	LOC_Os02g	no_paralog	Os02g0268	3.98	OS-CX:1.0(	1/11
222	LOC_Os01g	LOC_Os05g	Os01g0165	3.98	HS-LC:0.58	1/11
223	LOC_Os02g	no_paralog	Os02g0714	3.97	AT-CX:1.0(	1/11
224	LOC_Os03g	LOC_Os10g	Os03g0241	3.97	AT-CX:1.0(	1/11
225	LOC_Os11g	no_paralog	Os11g0148	3.96	OS-CX:1.0(	1/11
226	LOC_Os03g	no_paralog	Os03g0667	3.96	AT-CX:1.0(	1/11
227	LOC_Os06g	no_paralog	Os06g0110	3.96	OS-CX:1.0(	1/11
228	LOC_Os09g	LOC_Os02g	Os09g0427	3.94	OS-CX:1.0(	1/11

Do not distribute

229	LOC_Os05g	no_paralog	Os05g0559	3.93	OS-CX:1.0(	1/11
230	LOC_Os02g	no_paralog	Os02g0534	3.93	AT-CX:1.0(	1/11
231	LOC_Os08g	no_paralog	Os08g0248	3.92	OS-CX:0.5;	1/11
232	LOC_Os02g	no_paralog	Os02g0821	3.91	AT-CX:1.0(	1/11
233	LOC_Os07g	no_paralog	Os07g0214	3.91	OS-CX:1.0(	1/11
234	LOC_Os02g	no_paralog	Os02g0268	3.91	OS-CX:1.0(	1/11
235	LOC_Os10g	no_paralog	Os10g0400	3.9	OS-CX:1.0(	1/11
236	LOC_Os07g	LOC_Os03g	Os07g0616	3.89	AT-CX:1.0(	1/11
237	LOC_Os01g	no_paralog	Os01g0104	3.89	OS-CX:1.0(	1/11
238	LOC_Os02g	no_paralog	Os02g0725	3.89	OS-CX:1.0(	1/11
239	LOC_Os08g	LOC_Os09g	Os08g0550	3.89	SC-LC:0.66	1/11
240	LOC_Os09g	LOC_Os08g	Os09g0541	3.89	SC-LC:0.66	1/11
241	LOC_Os08g	no_paralog	Os08g0326	3.88	AT-CX:1.0(	1/11
242	LOC_Os01g	no_paralog	Os01g0860	3.88	AT-CX:1.0(	1/11
243	LOC_Os02g	no_paralog	Os02g0824	3.87	AT-CX:1.0(	1/11
244	LOC_Os01g	no_paralog	Os01g0917	3.87	OS-CX:1.0(	1/11
245	LOC_Os05g	LOC_Os01g	Os05g0160	3.87	OS-CX:0.5;	1/11
246	LOC_Os07g	no_paralog	Os07g0216	3.87	OS-CX:1.0(	1/11
247	LOC_Os07g	LOC_Os03g	Os07g0613	3.87	OS-CX:0.6!	1/11
248	LOC_Os05g	no_paralog	Os05g0331	3.87	OS-CX:1.0(	1/11
249	LOC_Os01g	no_paralog	Os01g0762	3.87	OS-CX:1.0(	1/11
250	LOC_Os07g	no_paralog	Os07g0604	3.87	OS-CX:1.0(	1/11
251	LOC_Os01g	no_paralog	Os01g0102	3.86	OS-CX:1.0(	1/11
252	LOC_Os02g	no_paralog	Os02g0453	3.86	OS-CX:1.0(	1/11
253	LOC_Os01g	LOC_Os05g	Os01g0633	3.85	OS-CX:1.0(	1/11
254	LOC_Os02g	no_paralog	Os02g0249	3.85	OS-CX:1.0(	1/11
255	LOC_Os08g	no_paralog	Os08g0378	3.84	OS-CX:1.0(	1/11
256	LOC_Os12g	no_paralog	Os12g0556	3.84	AT-CX:1.0(	1/11
257	LOC_Os05g	no_paralog	Os05g0499	3.84	OS-CX:1.0(	1/11
258	LOC_Os04g	no_paralog	Os04g0548	3.84	AT-CX:1.0(	1/11
259	LOC_Os02g	no_paralog	Os02g0139	3.83	AT-CX:1.0(	1/11
260	LOC_Os06g	no_paralog	Os06g0726	3.82	OS-CX:1.0(	1/11
261	LOC_Os03g	no_paralog	Os03g0766	3.82	OS-CX:1.0(	1/11
262	LOC_Os08g	no_paralog	Os08g0107	3.81	OS-CX:1.0(	1/11
263	LOC_Os05g	LOC_Os01g	Os05g0565	3.81	AT-CX:1.0(	1/11
264	LOC_Os02g	LOC_Os04g	Os02g0591	3.8	AT-CX:1.0(	1/11
265	LOC_Os03g	LOC_Os07g	Os03g0352	3.8	AT-CX:1.0(	1/11
266	LOC_Os03g	LOC_Os07g	Os03g0825	3.8	AT-CX:1.0(	1/11
267	LOC_Os07g	no_paralog	Os07g0213	3.79	OS-CX:1.0(	1/11
268	LOC_Os06g	no_paralog	Os06g0507	3.79	OS-CX:1.0(	1/11
269	LOC_Os08g	no_paralog	Os08g0196	3.79	OS-CX:1.0(	1/11
270	LOC_Os04g	no_paralog	Os04g0676	3.79	AT-CX:0.5⁄	1/11
271	LOC_Os04g	no_paralog	Os04g0429	3.79	OS-CX:1.0(	1/11
272	LOC_Os12g	LOC_Os11g	Os12g0150	3.77	AT-CX:1.0(	1/11
273	LOC_Os07g	no_paralog	Os07g0658	3.76	AT-CX:1.0(	1/11
274	LOC_Os03g	LOC_Os03g	Os03g0276	3.76	SC-LC:0.67	1/11

Do not distribute

275	LOC_Os05g	LOC_Os01gOs05g0460	3.76	SC-LC:0.67	1/11
276	LOC_Os07g	no_paralogOs07g0222	3.76	OS-CX:1.0(	1/11
277	LOC_Os02g	no_paralogOs02g0249	3.75	OS-CX:1.0(	1/11
278	LOC_Os12g	no_paralogOs12g0269	3.75	OS-CX:1.0(	1/11
279	LOC_Os12g	no_paralogOs12g0604	3.73	AT-CX:1.0(	1/11
280	LOC_Os08g	no_paralogOs08g0412	3.73	OS-CX:0.57	1/11
281	LOC_Os11g	LOC_Os12gOs11g0634	3.73	AT-CX:1.0(	1/11
282	LOC_Os03g	no_paralogOs03g0773	3.73	AT-CX:1.0(	1/11
283	LOC_Os01g	LOC_Os05gOs01g0840	3.73	SC-LC:0.67	1/11
284	LOC_Os08g	no_paralogOs08g0377	3.72	OS-CX:1.0(	1/11
285	LOC_Os06g	no_paralogOs06g0564	3.71	OS-GN:1.0	1/11
286	LOC_Os05g	LOC_Os01gOs05g0574	3.71	OS-CX:1.0(	1/11
287	LOC_Os12g	no_paralogOs12g0269	3.71	OS-CX:1.0(	1/11
288	LOC_Os12g	no_paralogOs12g0625	3.71	OS-GN:1.0	1/11
289	LOC_Os04g	LOC_Os08gOs04g0605	3.7	AT-CX:1.0(	1/11
290	LOC_Os11g	LOC_Os12gOs11g0703	3.7	SC-LC:0.67	1/11
291	LOC_Os03g	LOC_Os03gOs03g0821	3.69	SC-LC:0.67	1/11
292	LOC_Os12g	no_paralogOs12g0406	3.67	AT-CX:1.0(	1/11
293	LOC_Os01g	no_paralogOs01g0530	3.66	AT-CX:1.0(	1/11
294	LOC_Os03g	no_paralogOs03g0277	3.65	SC-LC:0.68	1/11
295	LOC_Os06g	no_paralogOs06g0128	3.65	OS-CX:0.54	1/11
296	LOC_Os08g	no_paralogOs08g0422	3.65	OS-CX:0.52	1/11
297	LOC_Os03g	no_paralogOs03g0804	3.64	AT-CX:1.0(	1/11
298	LOC_Os07g	no_paralogOs07g0219	3.64	OS-CX:1.0(	1/11
299	LOC_Os12g	no_paralogOs12g0168	3.64	SC-LC:0.69	1/11
300	LOC_Os01g	no_paralogOs01g0977	3.63	OS-CX:1.0(	1/11
301	LOC_Os04g	no_paralogOs04g0685	3.63	AT-CX:1.0(	1/11
302	LOC_Os05g	no_paralogOs05g0551	3.62	OS-CX:1.0(	1/11
303	LOC_Os01g	no_paralogOs01g0785	3.62	AT-CX:1.0(	1/11
304	LOC_Os10g	no_paralogOs10g0139	3.61	OS-CX:1.0(	1/11
305	LOC_Os03g	no_paralogOs03g0619	3.6	OS-CX:0.55	1/11
306	LOC_Os01g	no_paralogOs01g0180	3.6	OS-CX:1.0(	1/11
307	LOC_Os06g	no_paralogOs06g0115	3.6	OS-CX:1.0(	1/11
308	LOC_Os07g	no_paralogOs07g0214	3.6	OS-CX:1.0(	1/11
309	LOC_Os07g	no_paralogOs07g0668	3.59	OS-CX:1.0(	1/11
310	LOC_Os01g	no_paralogOs01g0365	3.59	OS-CX:1.0(	1/11
311	LOC_Os03g	LOC_Os05gOs03g0352	3.59	AT-CX:0.56	1/11
312	LOC_Os01g	no_paralogOs01g0144	3.59	AT-CX:0.51	1/11
313	LOC_Os12g	no_paralogOs12g0621	3.58	OS-CX:1.0(	1/11
314	LOC_Os03g	no_paralogOs03g0401	3.57	OS-CX:1.0(	1/11
315	LOC_Os04g	no_paralogOs04g0127	3.57	OS-CX:1.0(	1/11
316	LOC_Os04g	no_paralogOs04g0380	3.57	OS-CX:1.0(	1/11
317	LOC_Os02g	no_paralogOs02g0248	3.56	OS-CX:1.0(	1/11
318	LOC_Os07g	no_paralogOs07g0600	3.56	OS-CX:1.0(	1/11
319	LOC_Os01g	LOC_Os05gOs01g0356	3.54	OS-CX:1.0(	1/11
320	LOC_Os07g	no_paralogOs07g0203	3.54	OS-CX:1.0(	1/11

Do not distribute

321	LOC_Os02gno_paralogOs02g0131	3.54	AT-CX:1.00	1/11
322	LOC_Os05gno_paralogOs05g0329	3.53	OS-CX:1.00	1/11
323	LOC_Os07gno_paralogOs07g0206	3.52	OS-CX:1.00	1/11
324	LOC_Os02gno_paralogOs02g0332	3.51	OS-CX:0.53	1/11
325	LOC_Os03gno_paralogOs03g0187	3.5	AT-CX:1.00	1/11
326	LOC_Os02gLOC_Os03gOs02g0761	3.5	OS-CX:1.00	1/11
327	LOC_Os05gLOC_Os01gOs05g0170	3.49	OS-CX:1.00	1/11
328	LOC_Os11gno_paralogOs11g0512	3.49	OS-CX:1.00	1/11
329	LOC_Os06gLOC_Os02gOs06g0319	3.47	AT-CX:1.00	1/11
330	LOC_Os03gno_paralogOs03g0601	3.47	OS-CX:1.00	1/11
331	LOC_Os09gno_paralogOs09g0324	3.46	OS-CX:1.00	1/11
332	LOC_Os04gno_paralogOs04g0346	3.46	AT-CX:1.00	1/11
333	LOC_Os01gno_paralogOs01g0897	3.44	AT-CX:1.00	1/11
334	LOC_Os01gno_paralogOs01g0793	3.43	AT-CX:0.51	1/11
335	LOC_Os07gno_paralogOs07g0202	3.43	OS-CX:1.00	1/11
336	LOC_Os12gno_paralogOs12g0163	3.42	OS-CX:0.51	1/11
337	LOC_Os01gno_paralogOs01g0276	3.42	OS-CX:0.58	1/11
338	LOC_Os02gno_paralogOs02g0697	3.42	AT-CX:0.52	1/11
339	LOC_Os02gLOC_Os04gOs02g0528	3.4	OS-CX:1.00	1/11
340	LOC_Os08gno_paralogOs08g0142	3.4	OS-CX:1.00	1/11
341	LOC_Os01gLOC_Os05gOs01g0823	3.39	AT-CX:1.00	1/11
342	LOC_Os02gno_paralogOs02g0827	3.39	AT-CX:0.50	1/11
343	LOC_Os01gLOC_Os03gOs01g0303	3.38	OS-CX:1.00	1/11
344	LOC_Os03gno_paralogOs03g0835	3.37	HS-LC:1.00	1/11
345	LOC_Os12gno_paralogOs12g0540	3.37	HS-LC:1.00	1/11
346	LOC_Os08gno_paralogOs08g0513	3.37	OS-CX:1.00	1/11
347	LOC_Os02gno_paralogOs02g0576	3.36	OS-CX:1.00	1/11
348	LOC_Os05gLOC_Os02gOs05g0445	3.35	AT-CX:1.00	1/11
349	LOC_Os01gno_paralogOs01g0299	3.33	OS-CX:0.57	1/11
350	LOC_Os03gno_paralogOs03g0111	3.33	OS-CX:1.00	1/11
351	LOC_Os07gno_paralogOs07g0162	3.33	OS-CX:1.00	1/11
352	LOC_Os04gno_paralogOs04g0661	3.32	AT-CX:1.00	1/11
353	LOC_Os01gno_paralogOs01g0593	3.32	AT-CX:1.00	1/11
354	LOC_Os01gLOC_Os01gOs01g0812	3.31	AT-CX:1.00	1/11
355	LOC_Os05gLOC_Os01gOs05g0490	3.31	AT-CX:1.00	1/11
356	LOC_Os05gno_paralogOs05g0328	3.31	OS-CX:1.00	1/11
357	LOC_Os06gno_paralogOs06g0186	3.31	AT-CX:0.51	1/11
358	LOC_Os07gno_paralogOs07g0668	3.31	AT-CX:0.51	1/11
359	LOC_Os12gno_paralogOs12g0607	3.31	AT-CX:1.00	1/11
360	LOC_Os01gno_paralogOs01g0580	3.3	OS-CX:1.00	1/11
361	LOC_Os08gno_paralogOs08g0272	3.28	AT-CX:1.00	1/11
362	LOC_Os07gLOC_Os03gOs07g0180	3.27	AT-CX:1.00	1/11
363	LOC_Os08gno_paralogOs08g0139	3.27	AT-CX:0.51	1/11
364	LOC_Os12gno_paralogOs12g0269	3.26	OS-CX:1.00	1/11
365	LOC_Os02gLOC_Os06gOs02g0229	3.26	AT-CX:1.00	1/11
366	LOC_Os02gLOC_Os04gOs02g0580	3.26	AT-CX:1.00	1/11

Do not distribute

367	LOC_Os01g	no_paralog	Os01g0911	3.24	OS-CX:1.0(	1/11
368	LOC_Os01g	LOC_Os05g	Os01g0840	3.23	AT-CX:1.0(	1/11
369	LOC_Os01g	no_paralog	Os01g0296	3.23	OS-CX:1.0(	1/11
370	LOC_Os12g	no_paralog	Os12g0176	3.23	AT-CX:1.0(	1/11
371	LOC_Os02g	LOC_Os06g	Os02g0717	3.22	AT-CX:1.0(	1/11
372	LOC_Os08g	no_paralog	Os08g0521	3.22	OS-CX:1.0(	1/11
373	LOC_Os05g	no_paralog	Os05g0557	3.21	AT-CX:1.0(	1/11
374	LOC_Os08g	no_paralog	Os08g0162	3.2	AT-CX:1.0(	1/11
375	LOC_Os03g	no_paralog	Os03g0852	3.19	AT-CX:1.0(	1/11
376	LOC_Os04g	no_paralog	Os04g0650	3.16	OS-CX:1.0(	1/11
377	LOC_Os05g	LOC_Os01g	Os05g0358	3.16	CE-CC:1.0(	1/11
378	LOC_Os02g	no_paralog	Os02g0296	3.16	OS-CX:1.0(	1/11
379	LOC_Os05g	no_paralog	Os05g0304	3.15	OS-CX:1.0(	1/11
380	LOC_Os10g	no_paralog	Os10g0552	3.13	AT-CX:1.0(	1/11
381	LOC_Os02g	no_paralog	Os02g0172	3.11	OS-CX:1.0(	1/11
382	LOC_Os08g	no_paralog	Os08g0530	3.1	OS-CX:1.0(	1/11
383	LOC_Os09g	LOC_Os08g	Os09g0520	3.1	OS-CX:1.0(	1/11
384	LOC_Os07g	no_paralog	Os07g0670	3.1	AT-CX:1.0(	1/11
385	LOC_Os02g	no_paralog	Os02g0706	3.09	AT-CX:1.0(	1/11
386	LOC_Os05g	no_paralog	Os05g0329	3.08	OS-CX:1.0(	1/11
387	LOC_Os01g	LOC_Os05g	Os01g0687	3.06	OS-CX:1.0(	1/11
388	LOC_Os08g	no_paralog	Os08g0116	3.05	AT-CX:1.0(	1/11
389	LOC_Os09g	no_paralog	Os09g0489	3.05	OS-CX:1.0(	1/11
390	LOC_Os01g	no_paralog	Os01g0550	3.02	SC-GT:0.5(	1/11
391	LOC_Os10g	LOC_Os04g	Os10g0497	3.02	OS-CX:1.0(	1/11
392	LOC_Os07g	no_paralog	Os07g0421	3.02	OS-CX:1.0(	1/11
393	LOC_Os02g	no_paralog	Os02g0519	3.01	AT-CX:1.0(	1/11
394	LOC_Os05g	no_paralog	Os05g0270	3.01	AT-CX:1.0(	1/11
395	LOC_Os03g	no_paralog	Os03g0835	3	HS-LC:1.0(	1/11
396	LOC_Os07g	no_paralog	Os07g0292	3	AT-CX:1.0(	1/11
397	LOC_Os01g	no_paralog	Os01g0915	3	OS-CX:1.0(	1/11
398	LOC_Os02g	no_paralog	Os02g0456	2.98	OS-CX:1.0(	1/11
399	LOC_Os09g	LOC_Os08g	Os09g0471	2.98	AT-CX:1.0(	1/11
400	LOC_Os11g	LOC_Os12g	Os11g0151	2.98	AT-CX:1.0(	1/11
401	LOC_Os05g	no_paralog	Os05g0452	2.98	AT-CX:1.0(	1/11
402	LOC_Os04g	no_paralog	Os04g0589	2.97	OS-CX:1.0(	1/11
403	LOC_Os01g	no_paralog	Os01g0891	2.96	AT-CX:1.0(	1/11
404	LOC_Os07g	no_paralog	Os07g0671	2.96	AT-CX:1.0(	1/11
405	LOC_Os07g	no_paralog	Os07g0549	2.95	OS-CX:1.0(	1/11
406	LOC_Os10g	no_paralog	Os10g0198	2.95	OS-CX:1.0(	1/11
407	LOC_Os09g	no_paralog	Os09g0556	2.94	OS-CX:1.0(	1/11
408	LOC_Os01g	no_paralog	Os01g0661	2.94	AT-CX:1.0(	1/11
409	LOC_Os06g	no_paralog	Os06g0103	2.94	OS-GN:1.0	1/11
410	LOC_Os01g	no_paralog	Os01g0380	2.93	OS-CX:1.0(	1/11
411	LOC_Os12g	no_paralog	Os12g0575	2.93	AT-CX:1.0(	1/11
412	LOC_Os05g	no_paralog	Os05g0516	2.93	CE-CC:1.0(	1/11

Do not distribute

413	LOC_Os03gno_paralogOs03g0197	2.93	OS-CX:1.0(	1/11
414	LOC_Os08gno_paralogOs08g0129	2.92	OS-CX:1.0(	1/11
415	LOC_Os07gno_paralogOs07g0219	2.92	OS-CX:1.0(	1/11
416	LOC_Os05gno_paralogOs05g0329	2.9	OS-CX:1.0(	1/11
417	LOC_Os02gLOC_Os06gOs02g0222	2.89	OS-GN:1.0	1/11
418	LOC_Os04gno_paralogOs04g0436	2.87	OS-CX:1.0(	1/11
419	LOC_Os05gLOC_Os01gOs05g0156	2.87	OS-CX:1.0(	1/11
420	LOC_Os03gno_paralogOs03g0760	2.87	AT-CX:1.0(	1/11
421	LOC_Os06gno_paralogOs06g0564	2.87	OS-GN:1.0	1/11
422	LOC_Os03gLOC_Os03gOs03g0275	2.87	OS-CX:1.0(	1/11
423	LOC_Os10gno_paralogOs10g0138	2.87	OS-CX:1.0(	1/11
424	LOC_Os09gLOC_Os08gOs09g0498	2.87	AT-CX:1.0(	1/11
425	LOC_Os01gno_paralogOs01g0978	2.87	OS-GN:1.0	1/11
426	LOC_Os06gno_paralogOs06g0564	2.86	OS-GN:1.0	1/11
427	LOC_Os01gno_paralogOs01g0862	2.86	CE-CC:1.0(	1/11
428	LOC_Os12gno_paralogOs12g0194	2.86	AT-CX:1.0(	1/11
429	LOC_Os03gLOC_Os10gOs03g0143	2.86	OS-GN:1.0	1/11
430	LOC_Os05gLOC_Os01gOs05g0110	2.85	OS-CX:1.0(	1/11
431	LOC_Os10gLOC_Os03gOs10g0463	2.85	OS-GN:1.0	1/11
432	LOC_Os06gno_paralogOs06g0149	2.85	OS-GN:1.0	1/11
433	LOC_Os09gno_paralogOs09g0556	2.85	OS-GN:1.0	1/11
434	LOC_Os05gno_paralogOs05g0571	2.85	OS-CX:1.0(	1/11
435	LOC_Os04gno_paralogOs04g0165	2.85	OS-GN:1.0	1/11
436	LOC_Os06gno_paralogOs06g0149	2.85	OS-GN:1.0	1/11
437	LOC_Os02gno_paralogOs02g0192	2.85	OS-CX:1.0(	1/11
438	LOC_Os12gno_paralogOs12g0244	2.85	AT-CX:1.0(	1/11
439	LOC_Os02gno_paralogOs02g0822	2.84	HS-LC:1.0(	1/11
440	LOC_Os06gno_paralogOs06g0498	2.84	OS-CX:1.0(	1/11
441	LOC_Os03gno_paralogOs03g0747	2.84	OS-GN:1.0	1/11
442	LOC_Os01gno_paralogOs01g0823	2.83	OS-CX:1.0(	1/11
443	LOC_Os03gno_paralogOs03g0577	2.83	AT-CX:1.0(	1/11
444	LOC_Os07gno_paralogOs07g0608	2.83	AT-CX:1.0(	1/11
445	LOC_Os04gno_paralogOs04g0446	2.82	AT-CX:1.0(	1/11
446	LOC_Os05gno_paralogOs05g0311	2.82	OS-CX:1.0(	1/11
447	LOC_Os01gno_paralogOs01g0518	2.82	OS-CX:1.0(	1/11
448	LOC_Os06gno_paralogOs06g0564	2.81	OS-GN:1.0	1/11
449	LOC_Os01gno_paralogOs01g0859	2.8	OS-CX:1.0(	1/11
450	LOC_Os03gLOC_Os03gOs03g0280	2.8	OS-CX:1.0(	1/11
451	LOC_Os01gLOC_Os05gOs01g0310	2.8	CE-CC:1.0(	1/11
452	LOC_Os03gno_paralogOs03g0300	2.8	AT-CX:1.0(	1/11
453	LOC_Os01gno_paralogOs01g0271	2.79	AT-CX:1.0(	1/11
454	LOC_Os05gno_paralogOs05g0180	2.79	SC-CC:1.0(	1/11
455	LOC_Os05gno_paralogOs05g0594	2.79	AT-CX:1.0(	1/11
456	LOC_Os11gno_paralogOs11g0255	2.79	OS-CX:1.0(	1/11
457	LOC_Os02gLOC_Os01gOs02g0273	2.79	CE-CC:1.0(	1/11
458	LOC_Os06gLOC_Os01gOs06g0332	2.79	CE-CC:1.0(	1/11

Do not distribute

459	LOC_Os11g	no_paralog	Os11g0512	2.78	OS-CX:1.0(	1/11
460	LOC_Os01g	LOC_Os01g	Os01g0721	2.77	AT-CX:1.0(	1/11
461	LOC_Os03g	no_paralog	Os03g0831	2.77	AT-CX:1.0(	1/11
462	LOC_Os09g	LOC_Os08g	Os09g0555	2.76	OS-CX:1.0(	1/11
463	LOC_Os05g	no_paralog	Os05g0415	2.76	OS-CX:1.0(	1/11
464	LOC_Os03g	no_paralog	Os03g0669	2.76	CE-CC:1.0(	1/11
465	LOC_Os06g	no_paralog	Os06g0144	2.75	OS-CX:1.0(	1/11
466	LOC_Os06g	no_paralog	Os06g0257	2.74	AT-CX:1.0(	1/11
467	LOC_Os02g	no_paralog	Os02g0102	2.74	AT-CX:1.0(	1/11
468	LOC_Os09g	no_paralog	Os09g0115	2.74	OS-CX:1.0(	1/11
469	LOC_Os02g	LOC_Os10g	Os02g0684	2.74	OS-CX:1.0(	1/11
470	LOC_Os03g	LOC_Os03g	Os03g0663	2.74	OS-CX:1.0(	1/11
471	LOC_Os07g	no_paralog	Os07g0155	2.73	OS-CX:1.0(	1/11
472	LOC_Os02g	no_paralog	Os02g0177	2.73	OS-CX:1.0(	1/11
473	LOC_Os02g	no_paralog	Os02g0680	2.73	OS-GN:1.0	1/11
474	LOC_Os01g	no_paralog	Os01g0375	2.73	AT-CX:1.0(	1/11
475	LOC_Os05g	no_paralog	Os05g0104	2.73	CE-CC:1.0(	1/11
476	LOC_Os12g	no_paralog	Os12g0278	2.73	CE-CC:1.0(	1/11
477	LOC_Os08g	no_paralog	Os08g0556	2.73	CE-CC:1.0(	1/11
478	LOC_Os01g	no_paralog	Os01g0112	2.72	AT-CX:1.0(	1/11
479	LOC_Os01g	LOC_Os01g	Os01g0743	2.72	OS-CX:1.0(	1/11
480	LOC_Os03g	no_paralog	Os03g0369	2.72	OS-CX:1.0(	1/11
481	LOC_Os12g	no_paralog	Os12g0538	2.72	HS-LC:1.0(	1/11
482	LOC_Os06g	LOC_Os02g	Os06g0285	2.72	OS-CX:1.0(	1/11
483	LOC_Os01g	no_paralog	Os01g0393	2.71	OS-CX:1.0(	1/11
484	LOC_Os11g	no_paralog	Os11g0620	2.71	OS-CX:1.0(	1/11
485	LOC_Os08g	no_paralog	Os08g0244	2.7	OS-CX:1.0(	1/11
486	LOC_Os02g	LOC_Os04g	Os02g0625	2.69	AT-CX:1.0(	1/11
487	LOC_Os02g	no_paralog	Os02g0169	2.69	OS-GN:1.0	1/11
488	LOC_Os09g	no_paralog	Os09g0368	2.68	OS-CX:1.0(	1/11
489	LOC_Os10g	LOC_Os03g	Os10g0561	2.67	OS-GN:1.0	1/11
490	LOC_Os03g	no_paralog	Os03g0587	2.67	OS-GN:1.0	1/11
491	LOC_Os03g	LOC_Os07g	Os03g0796	2.67	AT-CX:1.0(	1/11
492	LOC_Os07g	no_paralog	Os07g0555	2.66	OS-CX:1.0(	1/11
493	LOC_Os01g	no_paralog	Os01g0887	2.66	OS-GN:1.0	1/11
494	LOC_Os06g	no_paralog	Os06g0156	2.65	OS-CX:1.0(	1/11
495	LOC_Os04g	no_paralog	Os04g0458	2.65	OS-CX:1.0(	1/11
496	LOC_Os06g	LOC_Os02g	Os06g0105	2.65	OS-GN:1.0	1/11
497	LOC_Os08g	no_paralog	Os08g0130	2.65	AT-CX:1.0(	1/11
498	LOC_Os01g	no_paralog	na	2.64	OS-CX:1.0(	1/11
499	LOC_Os04g	no_paralog	Os04g0620	2.63	SC-LC:1.00	1/11
500	LOC_Os03g	no_paralog	Os03g0430	2.63	OS-CX:1.0(	1/11
501	LOC_Os02g	no_paralog	Os02g0122	2.63	OS-CX:1.0(	1/11
502	LOC_Os02g	no_paralog	Os02g0185	2.62	OS-CX:1.0(	1/11
503	LOC_Os04g	no_paralog	Os04g0289	2.62	OS-CX:1.0(	1/11
504	LOC_Os02g	no_paralog	Os02g0266	2.62	OS-CX:1.0(	1/11

Do not distribute

505	LOC_Os11gno_paralogOs11g0256	2.62	AT-CX:1.0(	1/11
506	LOC_Os07gLOC_Os03gOs07g0109	2.61	AT-CX:1.0(	1/11
507	LOC_Os03gno_paralogOs03g0363	2.61	OS-CX:1.0(	1/11
508	LOC_Os05gno_paralogOs05g0243	2.61	AT-CX:1.0(	1/11
509	LOC_Os09gno_paralogOs09g0480	2.6	OS-CX:1.0(	1/11
510	LOC_Os07gno_paralogOs07g0539	2.6	OS-CX:1.0(	1/11
511	LOC_Os02gno_paralogOs02g0708	2.58	HS-LC:1.0C	1/11
512	LOC_Os12gno_paralogOs12g0235	2.58	OS-CX:1.0(	1/11
513	LOC_Os02gno_paralogOs02g0708	2.57	AT-CX:1.0(	1/11
514	LOC_Os03gno_paralogOs03g0831	2.57	OS-CX:1.0(	1/11
515	LOC_Os09gno_paralogOs09g0338	2.57	OS-GN:1.0	1/11
516	LOC_Os03gno_paralogOs03g0622	2.57	AT-CX:1.0(	1/11
517	LOC_Os11gLOC_Os12gOs11g0135	2.57	AT-CX:1.0(	1/11
518	LOC_Os09gLOC_Os04gOs09g0526	2.57	OS-CX:1.0(	1/11
519	LOC_Os03gno_paralogOs03g0701	2.56	SC-CC:1.0C	1/11
520	LOC_Os02gno_paralogOs02g0462	2.56	AT-CX:1.0(	1/11
521	LOC_Os02gno_paralogOs02g0255	2.56	AT-CX:1.0(	1/11
522	LOC_Os12gno_paralogOs12g0578	2.55	OS-CX:1.0(	1/11
523	LOC_Os12gLOC_Os11gOs12g0133	2.55	AT-CX:1.0(	1/11
524	LOC_Os10gno_paralogOs10g0389	2.55	OS-CX:1.0(	1/11
525	LOC_Os10gno_paralogOs10g0145	2.55	OS-CX:1.0(	1/11
526	LOC_Os11gno_paralogOs11g0134	2.55	OS-CX:1.0(	1/11
527	LOC_Os03gno_paralogOs03g0718	2.55	HS-LC:1.0C	1/11
528	LOC_Os01gLOC_Os05gOs01g0964	2.55	HS-LC:1.0C	1/11
529	LOC_Os02gLOC_Os04gOs02g0576	2.54	AT-CX:1.0(	1/11
530	LOC_Os05gLOC_Os01gOs05g0106	2.54	HS-LC:1.0C	1/11
531	LOC_Os08gno_paralogOs08g0345	2.54	OS-CX:1.0(	1/11
532	LOC_Os03gLOC_Os03gOs03g0793	2.54	OS-CX:1.0(	1/11
533	LOC_Os08gno_paralogOs08g0155	2.54	AT-CX:1.0(	1/11
534	LOC_Os09gLOC_Os08gOs09g0482	2.53	HS-LC:1.0C	1/11
535	LOC_Os09gno_paralogOs09g0482	2.53	HS-LC:1.0C	1/11
536	LOC_Os02gno_paralogOs02g0746	2.53	HS-LC:1.0C	1/11
537	LOC_Os08gLOC_Os09gOs08g0500	2.53	HS-LC:1.0C	1/11
538	LOC_Os09gno_paralogOs09g0509	2.52	AT-CX:1.0(	1/11
539	LOC_Os07gno_paralogOs07g0415	2.52	OS-CX:1.0(	1/11
540	LOC_Os06gno_paralogOs06g0151	2.52	OS-GN:1.0	1/11
541	LOC_Os01gno_paralogOs01g0617	2.51	AT-CX:1.0(	1/11
542	LOC_Os01gLOC_Os05gOs01g0643	2.51	OS-CX:1.0(	1/11
543	LOC_Os03gno_paralogOs03g0797	2.51	OS-CX:1.0(	1/11
544	LOC_Os04gLOC_Os03gOs04g0580	2.5	OS-CX:1.0(	1/11
545	LOC_Os01gLOC_Os05gOs01g0679	2.5	OS-CX:1.0(	1/11
546	LOC_Os10gno_paralogOs10g0498	2.49	SC-LC:1.00	1/11
547	LOC_Os08gno_paralogOs08g0508	2.49	AT-CX:1.0(	1/11
548	LOC_Os04gLOC_Os03gOs04g0498	2.49	OS-CX:1.0(	1/11
549	LOC_Os11gno_paralogOs11g0598	2.49	OS-CX:1.0(	1/11
550	LOC_Os01gno_paralogOs01g0505	2.48	OS-CX:1.0(	1/11

Do not distribute

551	LOC_Os08gno_paralogOs08g0542	2.48	AT-CX:1.0(	1/11
552	LOC_Os08gno_paralogOs08g0151	2.48	AT-CX:1.0(	1/11
553	LOC_Os01gLOC_Os05gOs01g0662	2.47	OS-GN:1.0	1/11
554	LOC_Os06gno_paralogOs06g0527	2.47	OS-CX:1.0(	1/11
555	LOC_Os03gLOC_Os10gOs03g0182	2.47	SC-LC:1.00	1/11
556	LOC_Os01gno_paralogOs01g0235	2.47	OS-CX:1.0(	1/11
557	LOC_Os07gLOC_Os03gOs07g0659	2.47	OS-CX:1.0(	1/11
558	LOC_Os06gLOC_Os02gOs06g0195	2.46	SC-LC:1.00	1/11
559	LOC_Os01gno_paralogOs01g0788	2.46	AT-CX:1.0(	1/11
560	LOC_Os03gLOC_Os10gOs03g0251	2.46	OS-CX:1.0(	1/11
561	LOC_Os10gLOC_Os03gOs10g0390	2.46	OS-CX:1.0(	1/11
562	LOC_Os01gno_paralogOs01g0143	2.46	AT-CX:1.0(	1/11
563	LOC_Os02gno_paralogOs02g0324	2.45	OS-CX:1.0(	1/11
564	LOC_Os03gno_paralogOs03g0699	2.45	AT-CX:1.0(	1/11
565	LOC_Os05gno_paralogOs05g0368	2.45	AT-CX:1.0(	1/11
566	LOC_Os04gno_paralogOs04g0390	2.44	OS-CX:1.0(	1/11
567	LOC_Os07gno_paralogOs07g0627	2.44	OS-CX:1.0(	1/11
568	LOC_Os06gno_paralogOs06g0141	2.44	OS-CX:1.0(	1/11
569	LOC_Os02gno_paralogOs02g0121	2.44	OS-GN:1.0	1/11
570	LOC_Os05gLOC_Os01gOs05g0568	2.43	OS-GN:1.0	1/11
571	LOC_Os05gno_paralogOs05g0540	2.43	AT-CX:1.0(	1/11
572	LOC_Os03gno_paralogOs03g0240	2.43	AT-CX:1.0(	1/11
573	LOC_Os10gno_paralogOs10g0525	2.42	OS-CX:1.0(	1/11
574	LOC_Os03gno_paralogOs03g0122	2.42	OS-GN:1.0	1/11
575	LOC_Os07gno_paralogOs07g0206	2.42	OS-CX:1.0(	1/11
576	LOC_Os10gLOC_Os03gOs10g0170	2.41	AT-CX:1.0(	1/11
577	LOC_Os02gno_paralogOs02g0705	2.41	OS-CX:1.0(	1/11
578	LOC_Os03gno_paralogOs03g0166	2.41	OS-CX:1.0(	1/11
579	LOC_Os06gno_paralogOs06g0195	2.41	AT-CX:1.0(	1/11
580	LOC_Os12gno_paralogOs12g0443	2.4	AT-CX:1.0(	1/11
581	LOC_Os09gno_paralogOs09g0493	2.4	OS-CX:1.0(	1/11
582	LOC_Os07gno_paralogOs07g0214	2.4	OS-CX:1.0(	1/11
583	LOC_Os01gno_paralogOs01g0141	2.4	OS-CX:1.0(	1/11
584	LOC_Os08gno_paralogOs08g0307	2.39	SC-CC:1.0C	1/11
585	LOC_Os07gno_paralogOs07g0599	2.39	AT-CX:1.0(	1/11
586	LOC_Os10gno_paralogOs10g0140	2.38	OS-CX:1.0(	1/11
587	LOC_Os11gno_paralogOs11g0221	2.38	OS-CX:1.0(	1/11
588	LOC_Os04gno_paralogOs04g0625	2.38	AT-CX:1.0(	1/11
589	LOC_Os03gno_paralogOs03g0313	2.38	OS-CX:1.0(	1/11
590	LOC_Os08gLOC_Os09gOs08g0453	2.38	OS-CX:1.0(	1/11
591	LOC_Os07gno_paralogOs07g0146	2.37	OS-CX:1.0(	1/11
592	LOC_Os03gno_paralogOs03g0277	2.37	OS-CX:1.0(	1/11
593	LOC_Os07gno_paralogOs07g0577	2.37	AT-CX:1.0(	1/11
594	LOC_Os05gno_paralogOs05g0388	2.37	OS-GN:1.0	1/11
595	LOC_Os06gno_paralogOs06g0546	2.37	OS-GN:1.0	1/11
596	LOC_Os07gLOC_Os03gOs07g0658	2.37	SC-CC:1.0C	1/11

Do not distribute

597 LOC_Os08g no_paralog Os08g0506	2.37	OS-GN:1.0	1/11
598 LOC_Os05g LOC_Os01g Os05g0482	2.37	OS-GN:1.0	1/11
599 LOC_Os07g no_paralog Os07g0215	2.37	OS-CX:1.0(	1/11
600 LOC_Os06g no_paralog Os06g0155	2.36	OS-CX:1.0(	1/11
601 LOC_Os07g no_paralog Os07g0110	2.36	AT-CX:1.0(	1/11
602 LOC_Os01g no_paralog Os01g0813	2.36	SC-CC:1.0C	1/11
603 LOC_Os12g no_paralog Os12g0189	2.36	OS-CX:1.0(	1/11
604 LOC_Os04g no_paralog Os04g0608	2.36	OS-CX:1.0(	1/11
605 LOC_Os01g no_paralog Os01g0850	2.35	OS-CX:1.0(	1/11
606 LOC_Os07g no_paralog Os07g0603	2.35	AT-CX:1.0(	1/11
607 LOC_Os12g no_paralog Os12g0506	2.35	OS-CX:1.0(	1/11
608 LOC_Os02g no_paralog Os02g0658	2.35	AT-CX:1.0(	1/11
609 LOC_Os11g no_paralog Os11g0185	2.35	OS-CX:1.0(	1/11
610 LOC_Os06g no_paralog Os06g0137	2.35	AT-CX:1.0(	1/11
611 LOC_Os04g no_paralog Os04g0551	2.35	AT-CX:1.0(	1/11
612 LOC_Os11g no_paralog Os11g0482	2.35	OS-CX:1.0(	1/11
613 LOC_Os03g no_paralog Os03g0665	2.35	OS-CX:1.0(	1/11
614 LOC_Os04g no_paralog Os04g0195	2.34	SC-CC:1.0C	1/11
615 LOC_Os05g LOC_Os01g Os05g0497	2.34	OS-CX:1.0(	1/11
616 LOC_Os06g no_paralog Os06g0213	2.34	AT-CX:1.0(	1/11
617 LOC_Os06g LOC_Os02g Os06g0571	2.34	OS-CX:1.0(	1/11
618 LOC_Os03g no_paralog Os03g0699	2.34	OS-CX:1.0(	1/11
619 LOC_Os11g LOC_Os12g Os11g0145	2.34	SC-CC:1.0C	1/11
620 LOC_Os01g no_paralog Os01g0834	2.33	OS-CX:1.0(	1/11
621 LOC_Os12g no_paralog Os12g0465	2.33	OS-CX:1.0(	1/11
622 LOC_Os08g no_paralog Os08g0320	2.33	OS-CX:1.0(	1/11
623 LOC_Os02g no_paralog Os02g0525	2.33	OS-CX:1.0(	1/11
624 LOC_Os02g no_paralog Os02g0181	2.33	OS-GN:1.0	1/11
625 LOC_Os01g no_paralog Os01g0666	2.33	SC-CC:1.0C	1/11
626 LOC_Os12g LOC_Os11g Os12g0235	2.32	AT-CX:1.0(	1/11
627 LOC_Os08g no_paralog Os08g0427	2.32	AT-CX:1.0(	1/11
628 LOC_Os12g LOC_Os11g Os12g0141	2.32	SC-CC:1.0C	1/11
629 LOC_Os08g no_paralog Os08g0458	2.32	OS-CX:1.0(	1/11
630 LOC_Os08g no_paralog Os08g0129	2.32	OS-CX:1.0(	1/11
631 LOC_Os03g LOC_Os01g Os03g0330	2.31	OS-GN:1.0	1/11
632 LOC_Os01g no_paralog Os01g0143	2.31	OS-CX:1.0(	1/11
633 LOC_Os07g no_paralog Os07g0480	2.31	OS-CX:1.0(	1/11
634 LOC_Os06g LOC_Os02g Os06g0229	2.31	OS-CX:1.0(	1/11
635 LOC_Os02g no_paralog Os02g0164	2.31	AT-CX:1.0(	1/11
636 LOC_Os06g no_paralog Os06g0647	2.31	OS-CX:1.0(	1/11
637 LOC_Os03g no_paralog Os03g0115	2.31	AT-CX:1.0(	1/11
638 LOC_Os10g no_paralog Os10g0548	2.3	OS-CX:1.0(	1/11
639 LOC_Os01g LOC_Os03g Os01g0817	2.3	OS-GN:1.0	1/11
640 LOC_Os02g no_paralog Os02g0771	2.3	OS-CX:1.0(	1/11
641 LOC_Os11g LOC_Os12g Os11g0267	2.3	OS-GN:1.0	1/11
642 LOC_Os04g no_paralog Os04g0643	2.3	HS-LC:1.0C	1/11

Do not distribute

643	LOC_Os05g	LOC_Os01g	Os05g0438	2.3	HS-LC:1.00	1/11
644	LOC_Os03g	no_paralog	Os03g0836	2.3	HS-LC:1.00	1/11
645	LOC_Os04g	no_paralog	Os04g0107	2.3	HS-LC:1.00	1/11
646	LOC_Os01g	LOC_Os11g	Os01g0866	2.3	HS-LC:1.00	1/11
647	LOC_Os10g	no_paralog	Os10g0388	2.3	HS-LC:1.00	1/11
648	LOC_Os11g	LOC_Os01g	Os11g0163	2.3	HS-LC:1.00	1/11
649	LOC_Os10g	no_paralog	Os10g0510	2.3	HS-LC:1.00	1/11
650	LOC_Os12g	LOC_Os11g	Os12g0163	2.3	HS-LC:1.00	1/11
651	LOC_Os08g	LOC_Os04g	Os08g0170	2.3	HS-LC:1.00	1/11
652	LOC_Os03g	no_paralog	Os03g0669	2.3	AT-CX:1.00	1/11
653	LOC_Os05g	LOC_Os01g	Os05g0163	2.3	AT-CX:1.00	1/11
654	LOC_Os11g	no_paralog	Os11g0701	2.3	OS-CX:1.00	1/11
655	LOC_Os10g	LOC_Os02g	Os10g0472	2.3	OS-CX:1.00	1/11
656	LOC_Os03g	no_paralog	Os03g0185	2.3	AT-CX:1.00	1/11
657	LOC_Os10g	no_paralog	Os10g0521	2.29	SC-CC:1.00	1/11
658	LOC_Os08g	no_paralog	Os08g0538	2.29	OS-CX:1.00	1/11
659	LOC_Os04g	LOC_Os02g	Os04g0405	2.29	OS-GN:1.0	1/11
660	LOC_Os05g	no_paralog	Os05g0169	2.29	AT-CX:1.00	1/11
661	LOC_Os09g	no_paralog	Os09g0132	2.29	AT-CX:1.00	1/11
662	LOC_Os09g	no_paralog	Os09g0253	2.29	OS-CX:1.00	1/11
663	LOC_Os09g	no_paralog	Os09g0352	2.29	AT-CX:1.00	1/11
664	LOC_Os03g	no_paralog	Os03g0426	2.28	OS-GN:1.0	1/11
665	LOC_Os02g	LOC_Os04g	Os02g0526	2.28	OS-GN:1.0	1/11
666	LOC_Os07g	no_paralog	Os07g0562	2.28	AT-CX:1.00	1/11
667	LOC_Os04g	no_paralog	na	2.27	OS-CX:1.00	1/11
668	LOC_Os02g	no_paralog	Os02g0537	2.27	OS-GN:1.0	1/11
669	LOC_Os05g	no_paralog	Os05g0405	2.27	OS-CX:1.00	1/11
670	LOC_Os03g	LOC_Os03g	Os03g0705	2.27	SC-CC:1.00	1/11
671	LOC_Os08g	LOC_Os04g	Os08g0192	2.27	OS-CX:1.00	1/11
672	LOC_Os04g	LOC_Os02g	Os04g0405	2.27	OS-CX:1.00	1/11
673	LOC_Os03g	no_paralog	Os03g0559	2.27	OS-CX:1.00	1/11
674	LOC_Os05g	LOC_Os01g	Os05g0533	2.27	AT-CX:1.00	1/11
675	LOC_Os03g	LOC_Os07g	Os03g0286	2.27	OS-CX:1.00	1/11
676	LOC_Os05g	no_paralog	Os05g0293	2.26	OS-GN:1.0	1/11
677	LOC_Os04g	no_paralog	Os04g0546	2.26	OS-CX:1.00	1/11
678	LOC_Os09g	no_paralog	Os09g0525	2.26	OS-CX:1.00	1/11
679	LOC_Os04g	no_paralog	Os04g0585	2.26	AT-CX:1.00	1/11
680	LOC_Os05g	no_paralog	Os05g0104	2.25	AT-CX:1.00	1/11
681	LOC_Os01g	no_paralog	Os01g0322	2.25	OS-CX:1.00	1/11
682	LOC_Os04g	LOC_Os02g	na	2.25	OS-GN:1.0	1/11
683	LOC_Os10g	LOC_Os02g	na	2.25	OS-GN:1.0	1/11
684	LOC_Os08g	LOC_Os04g	na	2.25	OS-GN:1.0	1/11
685	LOC_Os07g	LOC_Os04g	na	2.25	OS-GN:1.0	1/11
686	LOC_Os04g	LOC_Os10g	na	2.25	OS-GN:1.0	1/11
687	LOC_Os03g	no_paralog	Os03g0646	2.25	OS-GN:1.0	1/11
688	LOC_Os11g	no_paralog	Os11g0269	2.25	OS-GN:1.0	1/11

Do not distribute

689	LOC_Os03g	no_paralog	Os03g0622	2.25	AT-CX:1.0(	1/11
690	LOC_Os04g	no_paralog	na	2.25	OS-GN:1.0	1/11
691	LOC_Os05g	no_paralog	na	2.25	OS-GN:1.0	1/11
692	LOC_Os03g	LOC_Os07g	Os03g0356	2.25	SC-CC:1.0(	1/11
693	LOC_Os01g	no_paralog	Os01g0513	2.25	AT-CX:1.0(	1/11
694	LOC_Os07g	no_paralog	Os07g0229	2.24	OS-GN:1.0	1/11
695	LOC_Os01g	no_paralog	Os01g0183	2.24	AT-CX:1.0(	1/11
696	LOC_Os06g	LOC_Os03g	Os06g0728	2.24	SC-CC:1.0(	1/11
697	LOC_Os08g	no_paralog	Os08g0559	2.24	OS-CX:1.0(	1/11
698	LOC_Os02g	no_paralog	Os02g0290	2.24	AT-CX:1.0(	1/11
699	LOC_Os10g	no_paralog	Os10g0495	2.23	OS-GN:1.0	1/11
700	LOC_Os04g	no_paralog	Os04g0492	2.23	OS-GN:1.0	1/11
701	LOC_Os04g	LOC_Os10g	na	2.23	OS-GN:1.0	1/11
702	LOC_Os04g	no_paralog	na	2.23	OS-GN:1.0	1/11
703	LOC_Os02g	no_paralog	na	2.23	OS-GN:1.0	1/11
704	LOC_Os03g	no_paralog	Os03g0345	2.22	OS-CX:1.0(	1/11
705	LOC_Os07g	LOC_Os03g	Os07g0181	2.22	OS-CX:1.0(	1/11
706	LOC_Os03g	no_paralog	Os03g0116	2.22	OS-CX:1.0(	1/11
707	LOC_Os12g	LOC_Os11g	Os12g0167	2.22	AT-CX:1.0(	1/11
708	LOC_Os11g	no_paralog	Os11g0175	2.22	OS-CX:1.0(	1/11
709	LOC_Os06g	no_paralog	Os06g0158	2.22	AT-CX:1.0(	1/11
710	LOC_Os04g	LOC_Os02g	Os04g0389	2.22	OS-GN:1.0	1/11
711	LOC_Os02g	no_paralog	Os02g0784	2.22	OS-CX:1.0(	1/11
712	LOC_Os04g	no_paralog	na	2.22	OS-GN:1.0	1/11
713	LOC_Os01g	no_paralog	Os01g0583	2.22	AT-CX:1.0(	1/11
714	LOC_Os12g	no_paralog	na	2.21	OS-GN:1.0	1/11
715	LOC_Os03g	no_paralog	Os03g0725	2.21	AT-CX:1.0(	1/11
716	LOC_Os04g	no_paralog	Os04g0389	2.21	OS-GN:1.0	1/11
717	LOC_Os02g	LOC_Os10g	Os02g0629	2.21	OS-CX:1.0(	1/11
718	LOC_Os09g	no_paralog	Os09g0275	2.21	OS-CX:1.0(	1/11
719	LOC_Os10g	no_paralog	Os10g0488	2.21	AT-CX:1.0(	1/11
720	LOC_Os03g	no_paralog	Os03g0350	2.21	AT-CX:1.0(	1/11
721	LOC_Os03g	no_paralog	Os03g0728	2.2	OS-CX:1.0(	1/11
722	LOC_Os02g	LOC_Os06g	Os02g0745	2.2	OS-CX:1.0(	1/11
723	LOC_Os02g	LOC_Os10g	na	2.2	OS-GN:1.0	1/11
724	LOC_Os08g	no_paralog	Os08g0295	2.2	OS-CX:1.0(	1/11
725	LOC_Os05g	no_paralog	Os05g0582	2.2	OS-CX:1.0(	1/11
726	LOC_Os02g	LOC_Os04g	Os02g0510	2.2	OS-GN:1.0	1/11
727	LOC_Os08g	LOC_Os04g	Os08g0171	2.19	OS-GN:1.0	1/11
728	LOC_Os01g	no_paralog	Os01g0722	2.19	OS-GN:1.0	1/11
729	LOC_Os03g	no_paralog	Os03g0308	2.19	OS-CX:1.0(	1/11
730	LOC_Os11g	LOC_Os12g	Os11g0131	2.19	OS-CX:1.0(	1/11
731	LOC_Os09g	no_paralog	Os09g0560	2.19	AT-CX:1.0(	1/11
732	LOC_Os02g	no_paralog	Os02g0761	2.19	OS-GN:1.0	1/11
733	LOC_Os10g	no_paralog	Os10g0400	2.18	AT-CX:1.0(	1/11
734	LOC_Os01g	no_paralog	Os01g0932	2.18	OS-CX:1.0(	1/11

Do not distribute

735	LOC_Os05g	no_paralog	Os05g0558	2.18	AT-CX:1.00	1/11
736	LOC_Os04g	no_paralog	Os04g0654	2.18	OS-CX:1.00	1/11
737	LOC_Os12g	no_paralog	Os12g0488	2.18	OS-GN:1.0	1/11
738	LOC_Os05g	no_paralog	Os05g0105	2.18	OS-CX:1.00	1/11
739	LOC_Os12g	no_paralog	Os12g0562	2.17	AT-CX:1.00	1/11
740	LOC_Os01g	no_paralog	Os01g0570	2.17	AT-CX:1.00	1/11
741	LOC_Os02g	no_paralog	Os02g0782	2.17	AT-CX:1.00	1/11
742	LOC_Os03g	no_paralog	Os03g0688	2.17	AT-CX:1.00	1/11
743	LOC_Os03g	no_paralog	Os03g0685	2.17	OS-CX:1.00	1/11
744	LOC_Os03g	no_paralog	Os03g0343	2.17	AT-CX:1.00	1/11
745	LOC_Os02g	no_paralog	Os02g0565	2.17	OS-GN:1.0	1/11
746	LOC_Os01g	no_paralog	Os01g0251	2.17	OS-GN:1.0	1/11
747	LOC_Os02g	no_paralog	Os02g0137	2.17	OS-GN:1.0	1/11
748	LOC_Os11g	no_paralog	Os11g0546	2.17	OS-CX:1.00	1/11
749	LOC_Os07g	LOC_Os03g	Os07g0616	2.16	OS-CX:1.00	1/11
750	LOC_Os01g	no_paralog	Os01g0901	2.16	OS-CX:1.00	1/11
751	LOC_Os03g	no_paralog	Os03g0336	2.16	OS-CX:1.00	1/11
752	LOC_Os07g	no_paralog	Os07g0150	2.16	OS-CX:1.00	1/11
753	LOC_Os12g	no_paralog	Os12g0561	2.16	AT-CX:1.00	1/11
754	LOC_Os08g	no_paralog	Os08g0133	2.16	OS-CX:1.00	1/11
755	LOC_Os09g	no_paralog	Os09g0116	2.16	AT-CX:1.00	1/11
756	LOC_Os02g	no_paralog	Os02g0202	2.16	OS-CX:1.00	1/11
757	LOC_Os08g	LOC_Os09g	Os08g0524	2.16	OS-CX:1.00	1/11
758	LOC_Os10g	no_paralog	Os10g0446	2.16	OS-CX:1.00	1/11
759	LOC_Os03g	no_paralog	Os03g0612	2.15	OS-CX:1.00	1/11
760	LOC_Os03g	no_paralog	Os03g0265	2.15	OS-GN:1.0	1/11
761	LOC_Os01g	LOC_Os05g	Os01g0315	2.15	OS-CX:1.00	1/11
762	LOC_Os06g	no_paralog	Os06g0635	2.15	OS-CX:1.00	1/11
763	LOC_Os04g	no_paralog	Os04g0244	2.15	AT-CX:1.00	1/11
764	LOC_Os03g	no_paralog	Os03g0565	2.15	OS-GN:1.0	1/11
765	LOC_Os03g	LOC_Os07g	Os03g0815	2.14	OS-CX:1.00	1/11
766	LOC_Os09g	LOC_Os08g	Os09g0499	2.14	OS-CX:1.00	1/11
767	LOC_Os08g	no_paralog	Os08g0206	2.14	AT-CX:1.00	1/11
768	LOC_Os02g	LOC_Os06g	Os02g0184	2.14	OS-CX:1.00	1/11
769	LOC_Os03g	no_paralog	Os03g0196	2.14	OS-GN:1.0	1/11
770	LOC_Os04g	no_paralog	Os04g0397	2.14	AT-CX:1.00	1/11
771	LOC_Os03g	no_paralog	Os03g0323	2.14	AT-CX:1.00	1/11
772	LOC_Os05g	no_paralog	Os05g0519	2.14	OS-GN:1.0	1/11
773	LOC_Os06g	no_paralog	Os06g0149	2.14	AT-CX:1.00	1/11
774	LOC_Os03g	no_paralog	Os03g0403	2.13	OS-GN:1.0	1/11
775	LOC_Os01g	no_paralog	Os01g0385	2.13	OS-CX:1.00	1/11
776	LOC_Os03g	no_paralog	Os03g0633	2.13	OS-CX:1.00	1/11
777	LOC_Os02g	no_paralog	Os02g0595	2.13	OS-GN:1.0	1/11
778	LOC_Os10g	no_paralog	Os10g0397	2.13	OS-CX:1.00	1/11
779	LOC_Os02g	no_paralog	Os02g0297	2.12	OS-GN:1.0	1/11
780	LOC_Os02g	no_paralog	Os02g0762	2.12	OS-GN:1.0	1/11

Do not distribute

781	LOC_Os02gno_paralogOs02g0120	2.12	AT-CX:1.0(	1/11
782	LOC_Os12gno_paralogOs12g0169	2.12	OS-GN:1.0	1/11
783	LOC_Os07gno_paralogOs07g0124	2.12	AT-CX:1.0(	1/11
784	LOC_Os03gno_paralogOs03g0832	2.11	AT-CX:1.0(	1/11
785	LOC_Os07gno_paralogOs07g0642	2.11	OS-GN:1.0	1/11
786	LOC_Os03gLOC_Os10gOs03g0101	2.11	OS-GN:1.0	1/11
787	LOC_Os02gLOC_Os04gOs02g0537	2.11	AT-CX:1.0(	1/11
788	LOC_Os06gno_paralogOs06g0712	2.11	OS-GN:1.0	1/11
789	LOC_Os06gLOC_Os02gOs06g0196	2.11	AT-CX:1.0(	1/11
790	LOC_Os07gno_paralogOs07g0685	2.11	OS-CX:1.0(	1/11
791	LOC_Os06gLOC_Os02gOs06g0193	2.1	OS-CX:1.0(	1/11
792	LOC_Os02gno_paralogOs02g0595	2.1	AT-CX:1.0(	1/11
793	LOC_Os04gLOC_Os02gOs04g0587	2.1	OS-CX:1.0(	1/11
794	LOC_Os06gno_paralogOs06g0498	2.1	AT-CX:1.0(	1/11
795	LOC_Os03gno_paralogOs03g0831	2.1	AT-CX:1.0(	1/11
796	LOC_Os06gno_paralogOs06g0708	2.1	AT-CX:1.0(	1/11
797	LOC_Os01gno_paralogOs01g0253	2.09	AT-CX:1.0(	1/11
798	LOC_Os01gno_paralogOs01g0841	2.09	OS-GN:1.0	1/11
799	LOC_Os01gno_paralogOs01g0742	2.09	OS-GN:1.0	1/11
800	LOC_Os01gno_paralogOs01g0283	2.09	OS-GN:1.0	1/11
801	LOC_Os03gno_paralogOs03g0704	2.09	OS-GN:1.0	1/11
802	LOC_Os01gno_paralogOs01g0642	2.09	OS-CX:1.0(	1/11
803	LOC_Os02gno_paralogOs02g0282	2.08	OS-CX:1.0(	1/11
804	LOC_Os02gno_paralogOs02g0817	2.08	OS-CX:1.0(	1/11
805	LOC_Os03gno_paralogOs03g0343	2.08	AT-CX:1.0(	1/11
806	LOC_Os03gno_paralogOs03g0296	2.08	AT-CX:1.0(	1/11
807	LOC_Os04gno_paralogOs04g0640	2.08	OS-CX:1.0(	1/11
808	LOC_Os06gno_paralogOs06g0236	2.08	AT-CX:1.0(	1/11
809	LOC_Os04gno_paralogOs04g0538	2.08	OS-GN:1.0	1/11
810	LOC_Os01gno_paralogOs01g0738	2.07	AT-CX:1.0(	1/11
811	LOC_Os04gno_paralogOs04g0151	2.07	OS-CX:1.0(	1/11
812	LOC_Os09gno_paralogOs09g0502	2.07	OS-CX:1.0(	1/11
813	LOC_Os12gno_paralogOs12g0530	2.07	AT-CX:1.0(	1/11
814	LOC_Os04gno_paralogOs04g0376	2.07	OS-CX:1.0(	1/11
815	LOC_Os06gno_paralogOs06g0265	2.06	AT-CX:1.0(	1/11
816	LOC_Os09gno_paralogOs09g0535	2.06	OS-GN:1.0	1/11
817	LOC_Os01gno_paralogOs01g0616	2.06	OS-CX:1.0(	1/11
818	LOC_Os01gno_paralogOs01g0147	2.06	OS-GN:1.0	1/11
819	LOC_Os03gno_paralogOs03g0151	2.05	OS-CX:1.0(	1/11
820	LOC_Os12gno_paralogna	2.05	OS-GN:1.0	1/11
821	LOC_Os12gno_paralogna	2.05	OS-GN:1.0	1/11
822	LOC_Os03gno_paralogOs03g0588	2.05	OS-CX:1.0(	1/11
823	LOC_Os01gno_paralogOs01g0857	2.05	OS-GN:1.0	1/11
824	LOC_Os04gno_paralogOs04g0670	2.05	OS-CX:1.0(	1/11
825	LOC_Os01gno_paralogOs01g0840	2.04	OS-CX:1.0(	1/11
826	LOC_Os10gLOC_Os02gOs10g0390	2.04	OS-CX:1.0(	1/11

Do not distribute

827	LOC_Os05g	no_paralog	Os05g0573	2.04	OS-CX:1.0(	1/11
828	LOC_Os03g	no_paralog	Os03g0815	2.03	OS-GN:1.0	1/11
829	LOC_Os03g	no_paralog	Os03g0761	2.03	AT-CX:1.0(	1/11
830	LOC_Os07g	no_paralog	Os07g0222	2.03	AT-CX:1.0(	1/11
831	LOC_Os11g	no_paralog	Os11g0153	2.03	AT-CX:1.0(	1/11
832	LOC_Os06g	LOC_Os10g	na	2.03	OS-GN:1.0	1/11
833	LOC_Os01g	LOC_Os04g	na	2.03	OS-GN:1.0	1/11
834	LOC_Os05g	LOC_Os04g	na	2.03	OS-GN:1.0	1/11
835	LOC_Os10g	LOC_Os04g	na	2.03	OS-GN:1.0	1/11
836	LOC_Os09g	no_paralog	na	2.03	OS-GN:1.0	1/11
837	LOC_Os04g	LOC_Os01g	na	2.03	OS-GN:1.0	1/11
838	LOC_Os04g	no_paralog	Os04g0662	2.03	AT-CX:1.0(	1/11
839	LOC_Os10g	LOC_Os04g	na	2.03	OS-GN:1.0	1/11
840	LOC_Os08g	LOC_Os04g	na	2.03	OS-GN:1.0	1/11
841	LOC_Os04g	LOC_Os01g	na	2.03	OS-GN:1.0	1/11
842	LOC_Os01g	LOC_Os04g	na	2.03	OS-GN:1.0	1/11
843	LOC_Os06g	no_paralog	Os06g0158	2.03	AT-CX:1.0(	1/11
844	LOC_Os01g	no_paralog	Os01g0174	2.02	OS-CX:1.0(	1/11
845	LOC_Os08g	no_paralog	Os08g0564	2.02	OS-CX:1.0(	1/11
846	LOC_Os04g	LOC_Os10g	na	2.02	OS-GN:1.0	1/11
847	LOC_Os09g	no_paralog	na	2.02	OS-GN:1.0	1/11
848	LOC_Os10g	LOC_Os04g	na	2.02	OS-GN:1.0	1/11
849	LOC_Os08g	LOC_Os04g	na	2.02	OS-GN:1.0	1/11
850	LOC_Os04g	LOC_Os05g	na	2.02	OS-GN:1.0	1/11
851	LOC_Os05g	LOC_Os04g	na	2.02	OS-GN:1.0	1/11
852	LOC_Os08g	LOC_Os04g	na	2.02	OS-GN:1.0	1/11
853	LOC_Os04g	LOC_Os10g	Os04g0528	2.02	OS-CX:1.0(	1/11
854	LOC_Os01g	no_paralog	Os01g0238	2.01	AT-CX:1.0(	1/11
855	LOC_Os07g	LOC_Os03g	Os07g0624	2.01	OS-CX:1.0(	1/11
856	LOC_Os10g	no_paralog	Os10g0369	2.01	AT-CX:1.0(	1/11
857	LOC_Os01g	no_paralog	Os01g0752	2.01	OS-CX:1.0(	1/11
858	LOC_Os09g	LOC_Os02g	Os09g0438	2.01	OS-CX:1.0(	1/11
859	LOC_Os05g	LOC_Os01g	Os05g0373	2.01	AT-CX:1.0(	1/11
860	LOC_Os12g	no_paralog	Os12g0415	2.01	OS-CX:1.0(	1/11
861	LOC_Os05g	LOC_Os01g	Os05g0487	2	OS-CX:1.0(	1/11
862	LOC_Os01g	no_paralog	Os01g0250	1.99	AT-CX:1.0(	1/11
863	LOC_Os07g	no_paralog	Os07g0158	1.99	OS-GN:1.0	1/11
864	LOC_Os06g	no_paralog	Os06g0247	1.99	AT-CX:1.0(	1/11
865	LOC_Os02g	LOC_Os06g	Os02g0105	1.99	OS-GN:1.0	1/11
866	LOC_Os06g	no_paralog	Os06g0499	1.99	OS-GN:1.0	1/11
867	LOC_Os08g	LOC_Os09g	Os08g0431	1.99	OS-GN:1.0	1/11
868	LOC_Os07g	no_paralog	Os07g0410	1.99	OS-GN:1.0	1/11
869	LOC_Os02g	no_paralog	Os02g0461	1.99	OS-CX:1.0(	1/11
870	LOC_Os12g	LOC_Os11g	Os12g0230	1.99	OS-GN:1.0	1/11
871	LOC_Os10g	LOC_Os03g	Os10g0441	1.99	OS-CX:1.0(	1/11
872	LOC_Os02g	no_paralog	Os02g0805	1.98	AT-CX:1.0(	1/11

Do not distribute

873	LOC_Os02g	no_paralog	Os02g0175	1.98	AT-CX:1.0C	1/11
874	LOC_Os08g	no_paralog	Os08g0137	1.98	AT-CX:1.0C	1/11
875	LOC_Os08g	LOC_Os04g	na	1.98	OS-GN:1.0	1/11
876	LOC_Os05g	LOC_Os04g	na	1.98	OS-GN:1.0	1/11
877	LOC_Os04g	LOC_Os01g	na	1.98	OS-GN:1.0	1/11
878	LOC_Os01g	LOC_Os04g	na	1.98	OS-GN:1.0	1/11
879	LOC_Os10g	LOC_Os04g	na	1.98	OS-GN:1.0	1/11
880	LOC_Os01g	no_paralog	Os01g0151	1.98	OS-GN:1.0	1/11
881	LOC_Os04g	LOC_Os02g	Os04g0497	1.98	OS-CX:1.0C	1/11
882	LOC_Os02g	no_paralog	Os02g0780	1.98	OS-CX:1.0C	1/11
883	LOC_Os10g	no_paralog	Os10g0495	1.97	AT-CX:1.0C	1/11
884	LOC_Os12g	no_paralog	Os12g0548	1.97	OS-GN:1.0	1/11
885	LOC_Os04g	no_paralog	Os04g0203	1.97	OS-CX:1.0C	1/11
886	LOC_Os03g	no_paralog	Os03g0413	1.97	OS-CX:1.0C	1/11
887	LOC_Os02g	no_paralog	Os02g0623	1.97	OS-GN:1.0	1/11
888	LOC_Os07g	no_paralog	Os07g0545	1.97	AT-CX:1.0C	1/11
889	LOC_Os07g	no_paralog	Os07g0640	1.97	AT-CX:1.0C	1/11
890	LOC_Os01g	no_paralog	Os01g0948	1.97	AT-CX:1.0C	1/11
891	LOC_Os05g	no_paralog	Os05g0279	1.97	OS-GN:1.0	1/11
892	LOC_Os05g	no_paralog	Os05g0595	1.96	OS-GN:1.0	1/11
893	LOC_Os06g	no_paralog	Os06g0300	1.96	AT-CX:1.0C	1/11
894	LOC_Os04g	no_paralog	Os04g0422	1.96	OS-CX:1.0C	1/11
895	LOC_Os03g	no_paralog	Os03g0125	1.96	OS-GN:1.0	1/11
896	LOC_Os11g	LOC_Os12g	Os11g0128	1.96	AT-CX:1.0C	1/11
897	LOC_Os03g	LOC_Os10g	Os03g0252	1.96	OS-GN:1.0	1/11
898	LOC_Os02g	no_paralog	Os02g0620	1.95	OS-CX:1.0C	1/11
899	LOC_Os05g	no_paralog	Os05g0332	1.95	OS-CX:1.0C	1/11
900	LOC_Os09g	LOC_Os08g	Os09g0308	1.95	OS-CX:1.0C	1/11
901	LOC_Os10g	no_paralog	Os10g0457	1.94	AT-CX:1.0C	1/11
902	LOC_Os02g	no_paralog	Os02g0436	1.94	AT-CX:1.0C	1/11
903	LOC_Os01g	no_paralog	Os01g0524	1.94	OS-CX:1.0C	1/11
904	LOC_Os07g	no_paralog	Os07g0134	1.94	OS-GN:1.0	1/11
905	LOC_Os04g	no_paralog	Os04g0480	1.94	OS-CX:1.0C	1/11
906	LOC_Os12g	no_paralog	Os12g0554	1.94	OS-CX:1.0C	1/11
907	LOC_Os05g	no_paralog	Os05g0366	1.94	AT-CX:1.0C	1/11
908	LOC_Os09g	LOC_Os08g	Os09g0408	1.93	OS-GN:1.0	1/11
909	LOC_Os09g	LOC_Os08g	Os09g0474	1.93	AT-CX:1.0C	1/11
910	LOC_Os04g	no_paralog	Os04g0665	1.93	AT-CX:1.0C	1/11
911	LOC_Os05g	no_paralog	Os05g0417	1.93	OS-CX:1.0C	1/11
912	LOC_Os08g	no_paralog	Os08g0191	1.93	AT-CX:1.0C	1/11
913	LOC_Os08g	no_paralog	Os08g0110	1.93	OS-CX:1.0C	1/11
914	LOC_Os03g	LOC_Os07g	Os03g0755	1.93	OS-CX:1.0C	1/11
915	LOC_Os08g	no_paralog	Os08g0559	1.93	AT-CX:1.0C	1/11
916	LOC_Os04g	no_paralog	Os04g0476	1.93	OS-CX:1.0C	1/11
917	LOC_Os12g	LOC_Os11g	Os12g0125	1.93	AT-CX:1.0C	1/11
918	LOC_Os03g	LOC_Os07g	Os03g0370	1.92	AT-CX:1.0C	1/11

Do not distribute

919	LOC_Os03g	no_paralog	Os03g0598	1.92	AT-CX:1.0(	1/11
920	LOC_Os02g	no_paralog	Os02g0131	1.92	OS-CX:1.0(	1/11
921	LOC_Os04g	no_paralog	Os04g0311	1.92	OS-CX:1.0(	1/11
922	LOC_Os08g	LOC_Os09g	Os08g0359	1.92	OS-CX:1.0(	1/11
923	LOC_Os04g	no_paralog	Os04g0397	1.92	OS-GN:1.0	1/11
924	LOC_Os07g	no_paralog	Os07g0178	1.92	OS-CX:1.0(	1/11
925	LOC_Os12g	no_paralog	Os12g0170	1.92	AT-CX:1.0(	1/11
926	LOC_Os03g	no_paralog	Os03g0828	1.92	OS-GN:1.0	1/11
927	LOC_Os07g	no_paralog	Os07g0492	1.91	OS-GN:1.0	1/11
928	LOC_Os11g	no_paralog	Os11g0133	1.91	AT-CX:1.0(	1/11
929	LOC_Os08g	no_paralog	Os08g0254	1.91	OS-GN:1.0	1/11
930	LOC_Os06g	no_paralog	Os06g0687	1.91	OS-CX:1.0(	1/11
931	LOC_Os02g	no_paralog	Os02g0633	1.91	OS-CX:1.0(	1/11
932	LOC_Os12g	no_paralog	Os12g0193	1.91	OS-CX:1.0(	1/11
933	LOC_Os05g	no_paralog	Os05g0302	1.9	OS-CX:1.0(	1/11
934	LOC_Os06g	no_paralog	Os06g0103	1.9	OS-CX:1.0(	1/11
935	LOC_Os10g	no_paralog	Os10g0366	1.9	OS-CX:1.0(	1/11
936	LOC_Os02g	LOC_Os06g	Os02g0745	1.9	OS-CX:1.0(	1/11
937	LOC_Os10g	LOC_Os04g	na	1.9	OS-GN:1.0	1/11
938	LOC_Os05g	LOC_Os04g	na	1.9	OS-GN:1.0	1/11
939	LOC_Os04g	LOC_Os10g	na	1.9	OS-GN:1.0	1/11
940	LOC_Os02g	no_paralog	Os02g0806	1.9	OS-GN:1.0	1/11
941	LOC_Os04g	no_paralog	Os04g0531	1.9	OS-GN:1.0	1/11
942	LOC_Os03g	no_paralog	Os03g0355	1.9	AT-CX:1.0(	1/11
943	LOC_Os03g	no_paralog	Os03g0225	1.89	OS-CX:1.0(	1/11
944	LOC_Os10g	LOC_Os03g	Os10g0465	1.89	OS-CX:1.0(	1/11
945	LOC_Os06g	no_paralog	Os06g0116	1.89	OS-CX:1.0(	1/11
946	LOC_Os12g	LOC_Os11g	Os12g0114	1.89	OS-CX:1.0(	1/11
947	LOC_Os08g	no_paralog	Os08g0199	1.89	AT-CX:1.0(	1/11
948	LOC_Os11g	no_paralog	Os11g0615	1.89	AT-CX:1.0(	1/11
949	LOC_Os06g	no_paralog	Os06g0131	1.89	OS-GN:1.0	1/11
950	LOC_Os12g	no_paralog	Os12g0528	1.89	AT-CX:1.0(	1/11
951	LOC_Os04g	no_paralog	Os04g0107	1.89	OS-GN:1.0	1/11
952	LOC_Os12g	no_paralog	Os12g0613	1.88	OS-CX:1.0(	1/11
953	LOC_Os03g	no_paralog	Os03g0179	1.88	OS-CX:1.0(	1/11
954	LOC_Os06g	no_paralog	Os06g0714	1.88	OS-CX:1.0(	1/11
955	LOC_Os03g	no_paralog	Os03g0711	1.88	AT-CX:1.0(	1/11
956	LOC_Os03g	no_paralog	Os03g0751	1.87	OS-GN:1.0	1/11
957	LOC_Os03g	no_paralog	Os03g0356	1.87	OS-GN:1.0	1/11
958	LOC_Os09g	no_paralog	Os09g0364	1.87	OS-CX:1.0(	1/11
959	LOC_Os02g	no_paralog	Os02g0197	1.87	OS-CX:1.0(	1/11
960	LOC_Os06g	LOC_Os02g	Os06g0168	1.87	AT-CX:1.0(	1/11
961	LOC_Os02g	no_paralog	Os02g0728	1.87	AT-CX:1.0(	1/11
962	LOC_Os11g	no_paralog	Os11g0545	1.87	OS-CX:1.0(	1/11
963	LOC_Os09g	no_paralog	Os09g0567	1.87	OS-CX:1.0(	1/11
964	LOC_Os02g	no_paralog	Os02g0234	1.87	OS-CX:1.0(	1/11

Do not distribute

965	LOC_Os09gLOC_Os02gOs09g0470	1.87	OS-CX:1.0(	1/11
966	LOC_Os12gno_paralogOs12g0560	1.86	AT-CX:1.0(	1/11
967	LOC_Os12gno_paralogOs12g0514	1.86	OS-CX:1.0(	1/11
968	LOC_Os12gno_paralogOs12g0613	1.86	AT-CX:1.0(	1/11
969	LOC_Os08gno_paralogOs08g0278	1.86	AT-CX:1.0(	1/11
970	LOC_Os01gLOC_Os05gOs01g0875	1.86	AT-CX:1.0(	1/11
971	LOC_Os09gno_paralogOs09g0467	1.86	AT-CX:1.0(	1/11
972	LOC_Os04gno_paralogOs04g0636	1.86	OS-CX:1.0(	1/11
973	LOC_Os03gno_paralogOs03g0273	1.86	OS-CX:1.0(	1/11
974	LOC_Os05gno_paralogOs05g0486	1.86	OS-CX:1.0(	1/11
975	LOC_Os02gno_paralogOs02g0277	1.86	OS-CX:1.0(	1/11
976	LOC_Os09gno_paralogOs09g0428	1.86	OS-CX:1.0(	1/11
977	LOC_Os02gno_paralogOs02g0684	1.86	OS-CX:1.0(	1/11
978	LOC_Os03gno_paralogOs03g0344	1.85	OS-CX:1.0(	1/11
979	LOC_Os11gLOC_Os12gOs11g0139	1.85	OS-CX:1.0(	1/11
980	LOC_Os10gLOC_Os03gOs10g0189	1.85	OS-GN:1.0	1/11
981	LOC_Os03gno_paralogOs03g0198	1.85	AT-CX:1.0(	1/11
982	LOC_Os07gno_paralogOs07g0123	1.85	SC-GT:1.0(	1/11
983	LOC_Os05gLOC_Os01gOs05g0438	1.85	AT-CX:1.0(	1/11
984	LOC_Os06gno_paralogOs06g0159	1.85	AT-CX:1.0(	1/11
985	LOC_Os06gno_paralogOs06g0160	1.85	AT-CX:1.0(	1/11
986	LOC_Os11gno_paralogOs11g0155	1.85	AT-CX:1.0(	1/11
987	LOC_Os01gLOC_Os04gOs01g0866	1.85	AT-CX:1.0(	1/11
988	LOC_Os02gno_paralogOs02g0815	1.85	AT-CX:1.0(	1/11
989	LOC_Os05gno_paralogOs05g0567	1.85	AT-CX:1.0(	1/11
990	LOC_Os05gno_paralogOs05g0528	1.84	OS-CX:1.0(	1/11
991	LOC_Os01gno_paralogOs01g0918	1.84	AT-CX:1.0(	1/11
992	LOC_Os09gLOC_Os02gOs09g0434	1.84	OS-CX:1.0(	1/11
993	LOC_Os07gno_paralogOs07g0623	1.84	AT-CX:1.0(	1/11
994	LOC_Os01gLOC_Os05gOs01g0328	1.84	AT-CX:1.0(	1/11
995	LOC_Os02gLOC_Os04gOs02g0549	1.84	AT-CX:1.0(	1/11
996	LOC_Os12gno_paralogOs12g0265	1.84	OS-CX:1.0(	1/11
997	LOC_Os03gno_paralogOs03g0115	1.84	OS-GN:1.0	1/11
998	LOC_Os01gno_paralogOs01g0116	1.84	OS-GN:1.0	1/11
999	LOC_Os04gLOC_Os02gOs04g0518	1.84	AT-CX:1.0(	1/11
1000	LOC_Os02gno_paralogOs02g0468	1.84	AT-CX:1.0(	1/11
1001	LOC_Os01gno_paralogOs01g0559	1.84	AT-CX:1.0(	1/11
1002	LOC_Os02gno_paralogOs02g0816	1.84	SC-GT:1.0(	1/11
1003	LOC_Os02gno_paralogOs02g0189	1.83	OS-CX:1.0(	1/11
1004	LOC_Os04gno_paralogOs04g0269	1.83	OS-CX:1.0(	1/11
1005	LOC_Os09gno_paralogOs09g0455	1.83	OS-CX:1.0(	1/11
1006	LOC_Os01gLOC_Os05gOs01g0350	1.83	OS-CX:1.0(	1/11
1007	LOC_Os05gno_paralogOs05g0435	1.83	AT-CX:1.0(	1/11
1008	LOC_Os08gno_paralogOs08g0225	1.83	AT-CX:1.0(	1/11
1009	LOC_Os02gno_paralogOs02g0768	1.83	AT-CX:1.0(	1/11
1010	LOC_Os04gLOC_Os02gOs04g0508	1.83	AT-CX:1.0(	1/11

Do not distribute

1011	LOC_Os07g	no_paralog	Os07g0638	1.83	OS-CX:1.0(	1/11
1012	LOC_Os04g	no_paralog	Os04g0683	1.83	OS-CX:1.0(	1/11
1013	LOC_Os08g	no_paralog	Os08g0142	1.83	OS-CX:1.0(	1/11
1014	LOC_Os12g	no_paralog	Os12g0176	1.83	AT-CX:1.0(	1/11
1015	LOC_Os04g	no_paralog	Os04g0118	1.82	OS-GN:1.0	1/11
1016	LOC_Os04g	no_paralog	Os04g0619	1.82	AT-CX:1.0(	1/11
1017	LOC_Os04g	LOC_Os03g	Os04g0559	1.82	OS-GN:1.0	1/11
1018	LOC_Os01g	no_paralog	Os01g0276	1.82	AT-CX:1.0(	1/11
1019	LOC_Os08g	no_paralog	Os08g0359	1.82	OS-CX:1.0(	1/11
1020	LOC_Os02g	no_paralog	Os02g0198	1.82	AT-CX:1.0(	1/11
1021	LOC_Os05g	no_paralog	Os05g0579	1.81	OS-GN:1.0	1/11
1022	LOC_Os07g	no_paralog	Os07g0626	1.81	AT-CX:1.0(	1/11
1023	LOC_Os03g	no_paralog	Os03g0726	1.81	OS-CX:1.0(	1/11
1024	LOC_Os06g	no_paralog	Os06g0168	1.81	AT-CX:1.0(	1/11
1025	LOC_Os03g	no_paralog	Os03g0307	1.81	OS-CX:1.0(	1/11
1026	LOC_Os07g	no_paralog	Os07g0644	1.81	OS-CX:1.0(	1/11
1027	LOC_Os01g	no_paralog	Os01g0105	1.81	OS-GN:1.0	1/11
1028	LOC_Os06g	no_paralog	Os06g0545	1.81	OS-CX:1.0(	1/11
1029	LOC_Os01g	LOC_Os01g	Os01g0584	1.81	OS-CX:1.0(	1/11
1030	LOC_Os01g	no_paralog	Os01g0312	1.81	OS-CX:1.0(	1/11
1031	LOC_Os03g	no_paralog	Os03g0734	1.81	OS-CX:1.0(	1/11
1032	LOC_Os02g	no_paralog	Os02g0244	1.8	OS-GN:1.0	1/11
1033	LOC_Os08g	LOC_Os09g	Os08g0366	1.8	OS-GN:1.0	1/11
1034	LOC_Os02g	LOC_Os01g	Os02g0516	1.8	OS-CX:1.0(	1/11
1035	LOC_Os08g	LOC_Os09g	Os08g0484	1.8	AT-CX:1.0(	1/11
1036	LOC_Os03g	LOC_Os07g	Os03g0800	1.8	OS-CX:1.0(	1/11
1037	LOC_Os03g	no_paralog	Os03g0844	1.8	OS-GN:1.0	1/11
1038	LOC_Os05g	LOC_Os01g	Os05g0419	1.8	AT-CX:1.0(	1/11
1039	LOC_Os02g	no_paralog	Os02g0754	1.8	OS-GN:1.0	1/11
1040	LOC_Os07g	no_paralog	Os07g0110	1.8	OS-GN:1.0	1/11
1041	LOC_Os09g	no_paralog	Os09g0437	1.8	OS-GN:1.0	1/11
1042	LOC_Os07g	no_paralog	Os07g0693	1.8	AT-CX:1.0(	1/11
1043	LOC_Os05g	no_paralog	Os05g0349	1.8	OS-CX:1.0(	1/11
1044	LOC_Os02g	no_paralog	Os02g0257	1.8	OS-CX:1.0(	1/11
1045	LOC_Os01g	no_paralog	Os01g0911	1.79	AT-CX:1.0(	1/11
1046	LOC_Os04g	LOC_Os02g	Os04g0462	1.79	AT-CX:1.0(	1/11
1047	LOC_Os10g	LOC_Os03g	Os10g0549	1.79	OS-GN:1.0	1/11
1048	LOC_Os06g	no_paralog	Os06g0728	1.79	AT-CX:1.0(	1/11
1049	LOC_Os11g	no_paralog	Os11g0523	1.79	OS-CX:1.0(	1/11
1050	LOC_Os02g	LOC_Os05g	Os02g0529	1.79	AT-CX:1.0(	1/11
1051	LOC_Os01g	no_paralog	Os01g0256	1.79	AT-CX:1.0(	1/11
1052	LOC_Os01g	LOC_Os01g	Os01g0208	1.79	OS-GN:1.0	1/11
1053	LOC_Os09g	LOC_Os08g	Os09g0315	1.79	OS-GN:1.0	1/11
1054	LOC_Os01g	no_paralog	Os01g0110	1.79	OS-GN:1.0	1/11
1055	LOC_Os01g	LOC_Os01g	Os01g0758	1.79	OS-GN:1.0	1/11
1056	LOC_Os10g	LOC_Os03g	Os10g0462	1.79	AT-CX:1.0(	1/11

Do not distribute

1057	LOC_Os07ɡno_paralogOs07g0459	1.79	OS-GN:1.0	1/11
1058	LOC_Os02ɡno_paralogOs02g0575	1.79	OS-GN:1.0	1/11
1059	LOC_Os04ɡno_paralogOs04g0640	1.79	OS-GN:1.0	1/11
1060	LOC_Os02ɡno_paralogOs02g0805	1.79	OS-GN:1.0	1/11
1061	LOC_Os05ɡno_paralogOs05g0323	1.79	OS-GN:1.0	1/11
1062	LOC_Os02ɡno_paralogOs02g0816	1.79	OS-GN:1.0	1/11
1063	LOC_Os11ɡno_paralogOs11g0216	1.79	OS-GN:1.0	1/11
1064	LOC_Os09ɡno_paralogOs09g0250	1.79	OS-GN:1.0	1/11
1065	LOC_Os03ɡLOC_Os07ɡOs03g0406	1.79	OS-CX:1.0(	1/11
1066	LOC_Os02ɡno_paralogOs02g0608	1.79	OS-GN:1.0	1/11
1067	LOC_Os01ɡno_paralogOs01g0640	1.78	OS-CX:1.0(	1/11
1068	LOC_Os03ɡLOC_Os07ɡOs03g0815	1.78	SC-GT:1.0(	1/11
1069	LOC_Os12ɡno_paralogOs12g0592	1.78	AT-CX:1.0(	1/11
1070	LOC_Os03ɡno_paralogOs03g0811	1.78	AT-CX:1.0(	1/11
1071	LOC_Os02ɡno_paralogOs02g0761	1.78	OS-GN:1.0	1/11
1072	LOC_Os02ɡLOC_Os04ɡOs02g0644	1.78	AT-CX:1.0(	1/11
1073	LOC_Os01ɡno_paralogOs01g0511	1.78	AT-CX:1.0(	1/11
1074	LOC_Os08ɡno_paralogOs08g0177	1.78	OS-CX:1.0(	1/11
1075	LOC_Os12ɡLOC_Os03ɡOs12g0618	1.78	AT-CX:1.0(	1/11
1076	LOC_Os01ɡno_paralogOs01g0259	1.77	AT-CX:1.0(	1/11
1077	LOC_Os03ɡno_paralogOs03g0215	1.77	OS-GN:1.0	1/11
1078	LOC_Os10ɡLOC_Os03ɡOs10g0409	1.77	OS-CX:1.0(	1/11
1079	LOC_Os02ɡno_paralogOs02g0123	1.77	OS-CX:1.0(	1/11
1080	LOC_Os03ɡno_paralogOs03g0257	1.77	AT-CX:1.0(	1/11
1081	LOC_Os04ɡno_paralogOs04g0105	1.77	AT-CX:1.0(	1/11
1082	LOC_Os06ɡno_paralogOs06g0187	1.77	OS-CX:1.0(	1/11
1083	LOC_Os05ɡno_paralogOs05g0583	1.77	AT-CX:1.0(	1/11
1084	LOC_Os04ɡno_paralogOs04g0464	1.76	SC-GT:1.0(	1/11
1085	LOC_Os05ɡno_paralogOs05g0558	1.76	AT-CX:1.0(	1/11
1086	LOC_Os01ɡno_paralogna	1.76	AT-CX:1.0(	1/11
1087	LOC_Os01ɡno_paralogOs01g0152	1.76	AT-CX:1.0(	1/11
1088	LOC_Os02ɡno_paralogOs02g0606	1.76	AT-CX:1.0(	1/11
1089	LOC_Os11ɡno_paralogOs11g0594	1.76	OS-CX:1.0(	1/11
1090	LOC_Os11ɡno_paralogOs11g0267	1.76	OS-CX:1.0(	1/11
1091	LOC_Os06ɡno_paralogOs06g0154	1.76	OS-GN:1.0	1/11
1092	LOC_Os03ɡLOC_Os10ɡOs03g0162	1.76	AT-CX:1.0(	1/11
1093	LOC_Os12ɡno_paralogOs12g0485	1.76	OS-GN:1.0	1/11
1094	LOC_Os02ɡno_paralogOs02g0519	1.76	OS-GN:1.0	1/11
1095	LOC_Os04ɡno_paralogOs04g0412	1.75	OS-CX:1.0(	1/11
1096	LOC_Os03ɡno_paralogOs03g0129	1.75	OS-GN:1.0	1/11
1097	LOC_Os07ɡno_paralogOs07g0157	1.75	OS-CX:1.0(	1/11
1098	LOC_Os03ɡno_paralogOs03g0735	1.75	OS-CX:1.0(	1/11
1099	LOC_Os12ɡno_paralogOs12g0182	1.75	OS-GN:1.0	1/11
1100	LOC_Os01ɡLOC_Os05ɡOs01g0762	1.75	AT-CX:1.0(	1/11
1101	LOC_Os05ɡno_paralogOs05g0397	1.75	OS-GN:1.0	1/11
1102	LOC_Os07ɡno_paralogOs07g0270	1.75	AT-CX:1.0(	1/11

Do not distribute

1103	LOC_Os03gno_paralogOs03g0622	1.75	OS-CX:1.0(	1/11
1104	LOC_Os02gno_paralogOs02g0489	1.75	AT-CX:1.0(	1/11
1105	LOC_Os09gno_paralogOs09g0557	1.75	OS-CX:1.0(	1/11
1106	LOC_Os06gno_paralogOs06g0186	1.75	OS-CX:1.0(	1/11
1107	LOC_Os07gno_paralogOs07g0643	1.75	OS-CX:1.0(	1/11
1108	LOC_Os06gno_paralogOs06g0137	1.75	OS-CX:1.0(	1/11
1109	LOC_Os12gLOC_Os03gOs12g0636	1.75	OS-CX:1.0(	1/11
1110	LOC_Os01gLOC_Os04gOs01g0920	1.75	OS-CX:1.0(	1/11
1111	LOC_Os10gno_paralogOs10g0494	1.75	OS-GN:1.0	1/11
1112	LOC_Os06gno_paralogOs06g0508	1.75	OS-CX:1.0(	1/11
1113	LOC_Os02gLOC_Os04gOs02g0580	1.75	AT-CX:1.0(	1/11
1114	LOC_Os01gno_paralogOs01g0923	1.74	OS-CX:1.0(	1/11
1115	LOC_Os05gno_paralogOs05g0176	1.74	OS-CX:1.0(	1/11
1116	LOC_Os06gno_paralogOs06g0224	1.74	OS-CX:1.0(	1/11
1117	LOC_Os09gno_paralogOs09g0453	1.74	OS-CX:1.0(	1/11
1118	LOC_Os01gno_paralogOs01g0266	1.74	AT-CX:1.0(	1/11
1119	LOC_Os01gno_paralogOs01g0265	1.74	OS-CX:1.0(	1/11
1120	LOC_Os07gno_paralogOs07g0408	1.74	OS-CX:1.0(	1/11
1121	LOC_Os05gno_paralogOs05g0457	1.74	OS-CX:1.0(	1/11
1122	LOC_Os09gno_paralogOs09g0382	1.74	OS-CX:1.0(	1/11
1123	LOC_Os02gLOC_Os04gOs02g0565	1.74	OS-CX:1.0(	1/11
1124	LOC_Os03gLOC_Os07gOs03g0408	1.74	AT-CX:1.0(	1/11
1125	LOC_Os03gno_paralogOs03g0183	1.74	AT-CX:1.0(	1/11

Do not distribute

rks related leaf rolling under drought

[Linked_qu	[GO_descriptions]
LOC_Os06¦	positive regulation of phosphorylation
LOC_Os02¦	response to salt stress
LOC_Os02¦	translation
LOC_Os02¦	na
LOC_Os02¦	translation
LOC_Os02¦	translation;cytokinesis by cell plate formation;leaf morphogenesis;trichome mc
LOC_Os02¦	translation;response to oxidative stress;response to cold;response to high light
LOC_Os02¦	translation
LOC_Os02¦	GTP catabolic process;translational elongation;pyrimidine ribonucleotide biosy
LOC_Os02¦	translation
LOC_Os02¦	ribosome biogenesis
LOC_Os06¦	cytoskeleton organization;unidimensional cell growth;plant-type cell wall bioge
LOC_Os02¦	nucleoside diphosphate phosphorylation;GTP biosynthetic process;UTP biosynt
LOC_Os02¦	translation;response to fungus
LOC_Os02¦	translation
LOC_Os02¦	translation;adaxial/abaxial pattern formation
LOC_Os02¦	translation;rRNA processing;ribosomal small subunit biogenesis
LOC_Os02¦	translation
LOC_Os02¦	translation
LOC_Os02¦	na
LOC_Os02¦	translation
LOC_Os02¦	translation
LOC_Os06¦	autophagic vacuole assembly
LOC_Os02¦	rRNA processing;translation;ribosomal small subunit biogenesis
LOC_Os02¦	rRNA processing;tRNA processing;methylation;RNA methylation
LOC_Os02¦	translation
LOC_Os02¦	auxin mediated signaling pathway;gynoecium development
LOC_Os02¦	translation
LOC_Os02¦	translation
LOC_Os02¦	translation
LOC_Os02¦	translation
LOC_Os02¦	proteolysis;cellular process;G2 phase of mitotic cell cycle;RNA methylation;puri
LOC_Os02¦	na
LOC_Os02¦	RNA splicing
LOC_Os02¦	translation
LOC_Os02¦	G2 phase of mitotic cell cycle;pyrimidine ribonucleotide biosynthetic process;pl
LOC_Os02¦	translation
LOC_Os02¦	na
LOC_Os02¦	G2 phase of mitotic cell cycle;pyrimidine ribonucleotide biosynthetic process;pl
LOC_Os02¦	translation
LOC_Os02¦	translation;response to salt stress;response to cadmium ion
LOC_Os02¦	translation
LOC_Os02¦	translation
LOC_Os02¦	translation

Do not distribute

LOC_Os02	na
LOC_Os02	translation
LOC_Os02	na
LOC_Os02	translation
LOC_Os02	translation
LOC_Os02	translation
LOC_Os02	na
LOC_Os02	RNA elongation;translation;pyrimidine ribonucleotide biosynthetic process;ribo
LOC_Os06	carbohydrate metabolic process;cellular amino acid metabolic process;phospho
LOC_Os02	translation;cell proliferation;adaxial/abaxial pattern formation;leaf morphogen
LOC_Os02	translation
LOC_Os02	translation
LOC_Os02	translation
LOC_Os02	translation
LOC_Os02	translation;response to oxidative stress;response to cold;response to high light
LOC_Os02	translation;adaxial/abaxial pattern formation
LOC_Os02	translation;ribosome biogenesis
LOC_Os02	translation;adaxial/abaxial pattern formation;post-embryonic organ developm
LOC_Os02	pseudouridine synthesis;ribosome biogenesis
LOC_Os02	rRNA processing;translation;growth;ribosomal small subunit biogenesis
LOC_Os02	rRNA processing;translation;growth;ribosomal small subunit biogenesis
LOC_Os02	translation
LOC_Os02	translation
LOC_Os02	na
LOC_Os02	translation
LOC_Os02	tRNA processing;oxidation reduction
LOC_Os02	translation
LOC_Os02	na
LOC_Os02	translation
LOC_Os02	translation
LOC_Os02	translation
LOC_Os02	translation
LOC_Os02	rRNA processing;protein import into nucleus;ribosome biogenesis;protein matu
LOC_Os02	na
LOC_Os02	translation
LOC_Os02	response to salt stress
LOC_Os02	G2 phase of mitotic cell cycle;pyrimidine ribonucleotide biosynthetic process;p
LOC_Os02	translation
LOC_Os02	maturation of SSU-rRNA from tricistronic rRNA transcript (SSU-rRNA, 5.8S rRNA
LOC_Os02	translation
LOC_Os02	na
LOC_Os02	na
LOC_Os02	translation;response to cold
LOC_Os02	translation
LOC_Os02	translation;translational elongation;defense response to bacterium
LOC_Os02	translation

Do not distribute

LOC_Os02	translation
LOC_Os02	protein folding;response to heat
LOC_Os02	na
LOC_Os02	na
LOC_Os02	RNA splicing, via endonucleolytic cleavage and ligation;DNA repair;regulation o
LOC_Os02	translation
LOC_Os02	nuclear mRNA splicing, via spliceosome;translation;ribosome biogenesis;protei
LOC_Os02	translation;ribosomal small subunit assembly;translational elongation
LOC_Os02	translation
LOC_Os02	translation
LOC_Os02	translation
LOC_Os02	translation
LOC_Os02	protein amino acid methylation;methylation;peptidyl-arginine N-methylation
LOC_Os02	na
LOC_Os06	intracellular protein transport
LOC_Os02	na
LOC_Os02	na
LOC_Os02	cysteine biosynthetic process from serine;cellular amino acid biosynthetic proc
LOC_Os02	translation;ribosomal small subunit assembly;response to osmotic stress;respo
LOC_Os02	auxin homeostasis
LOC_Os02	translation;response to UV-B;developmental process;cellular response to UV-B
LOC_Os02	pseudouridine synthesis;RNA processing;RNA modification
LOC_Os02	translation
LOC_Os02	biosynthetic process;glucan biosynthetic process;starch metabolic process;star
LOC_Os02	protein folding;response to heat
LOC_Os06	small GTPase mediated signal transduction;protein transport
LOC_Os02	na
LOC_Os02	na
LOC_Os02	translation
LOC_Os02	translation
LOC_Os02	translation
LOC_Os02	translation
LOC_Os02	cytokinesis by cell plate formation;translation;leaf morphogenesis;trichome mo
LOC_Os02	translation
LOC_Os02	translation
LOC_Os02	translation
LOC_Os02	translation
LOC_Os02	translation
LOC_Os02	translation
LOC_Os02	translation
LOC_Os02	protein peptidyl-prolyl isomerization;RNA methylation;rRNA processing;protein
LOC_Os02	na
LOC_Os02	translation
LOC_Os02	na
LOC_Os02	translation
LOC_Os02	ribosomal small subunit assembly;translation;translational elongation

Do not distribute

LOC_Os02¦translation
LOC_Os02¦translation
LOC_Os02¦translation
LOC_Os02¦translation
LOC_Os02¦na
LOC_Os02¦na
LOC_Os02¦na
LOC_Os02¦transport;protein transport
LOC_Os02¦translation
LOC_Os02¦translation
LOC_Os02¦translation;ribosome biogenesis
LOC_Os02¦RNA methylation;protein targeting to mitochondrion;transport;protein transpo
LOC_Os02¦translation
LOC_Os02¦translation
LOC_Os02¦translation
LOC_Os02¦tRNA aminoacylation for protein translation;lysyl-tRNA aminoacylation;translat
LOC_Os02¦translation
LOC_Os02¦auxin mediated signaling pathway;gynoecium development
LOC_Os02¦na
LOC_Os02¦RNA methylation;rRNA processing;tRNA processing
LOC_Os02¦translation
LOC_Os02¦RNA methylation;pseudouridine synthesis;rRNA processing;polar nucleus fusio
LOC_Os02¦na
LOC_Os02¦na
LOC_Os02¦DNA methylation;transcription, DNA-dependent;production of siRNA involved i
LOC_Os02¦translation
LOC_Os02¦translation
LOC_Os02¦translation
LOC_Os02¦nuclear mRNA splicing, via spliceosome;mRNA processing;RNA splicing
LOC_Os02¦regulation of transcription, DNA-dependent
LOC_Os02¦protein folding;response to zinc ion;cellular protein metabolic process;respons
LOC_Os02¦translation
LOC_Os02¦translational elongation
LOC_Os02¦ribosome biogenesis
LOC_Os02¦na
LOC_Os02¦embryo sac egg cell differentiation
LOC_Os02¦translation;megagametogenesis
LOC_Os02¦translation
LOC_Os02¦translation;rRNA export from nucleus
LOC_Os02¦na
LOC_Os02¦transport;protein transport
LOC_Os02¦translation
LOC_Os02¦translation
LOC_Os02¦translation
LOC_Os02¦translational elongation
LOC_Os02¦ribosome biogenesis

Do not distribute

LOC_Os02	na
LOC_Os02	regulation of transcription, DNA-dependent
LOC_Os06	thigmotropism;positive gravitropism
LOC_Os02	translation
LOC_Os02	na
LOC_Os06	regulation of transcription, DNA-dependent
LOC_Os02	regulation of transcription, DNA-dependent
LOC_Os02	translation
LOC_Os02	oxidation reduction
LOC_Os02	ribosome biogenesis
LOC_Os02	regulation of transcription, DNA-dependent
LOC_Os02	intracellular protein transport
LOC_Os02	na
LOC_Os02	na
LOC_Os02	na
LOC_Os02	na
LOC_Os02	translation;rRNA processing;ribosomal small subunit biogenesis
LOC_Os06	nuclear membrane fusion;karyogamy;protein folding;response to heat;embryo
LOC_Os02	cell redox homeostasis
LOC_Os02	transport
LOC_Os02	superoxide metabolic process;removal of superoxide radicals;oxidation reducti
LOC_Os02	translation;post-embryonic organ development;adaxial/abaxial pattern formati
LOC_Os02	G2 phase of mitotic cell cycle;pyrimidine ribonucleotide biosynthetic process;pl
LOC_Os02	translation
LOC_Os02	defense response to bacterium;root development;defense response to fungus;
LOC_Os06	regulation of transcription from RNA polymerase II promoter
LOC_Os02	na
LOC_Os02	na
LOC_Os02	na
LOC_Os02	translation
LOC_Os02	na
LOC_Os02	RNA processing
LOC_Os02	na
LOC_Os02	metabolic process;toxin catabolic process;response to cyclopentenone;para-an
LOC_Os06	negative regulation of flower development
LOC_Os02	translation;pyrimidine ribonucleotide biosynthetic process
LOC_Os02	na
LOC_Os02	regulation of transcription, DNA-dependent
LOC_Os02	defense response
LOC_Os02	na
LOC_Os02	heme biosynthetic process;nucleoside metabolic process;nucleotide biosynthet
LOC_Os02	ribosome biogenesis
LOC_Os06	na
LOC_Os02	translation;endonucleolytic cleavage in ITS1 to separate SSU-rRNA from 5.8S rR
LOC_Os02	protein amino acid dephosphorylation;multicellular organismal development
LOC_Os02	trehalose biosynthetic process;metabolic process

Do not distribute

LOC_Os02¦na
LOC_Os02¦translation
LOC_Os02¦purine nucleotide biosynthetic process;'de novo' pyrimidine base biosynthetic ¦
LOC_Os02¦translation
LOC_Os02¦type I hypersensitivity
LOC_Os02¦defense response
LOC_Os02¦na
LOC_Os02¦ribosomal small subunit assembly;endonucleolytic cleavage in ITS1 to separate
LOC_Os02¦regulation of transcription, DNA-dependent
LOC_Os02¦transcription, DNA-dependent;regulation of transcription, DNA-dependent
LOC_Os06¦regulation of protein catabolic process;regulation of catalytic activity
LOC_Os06¦regulation of protein catabolic process;regulation of catalytic activity
LOC_Os02¦RNA methylation;purine nucleotide biosynthetic process;GMP biosynthetic pro
LOC_Os02¦translation;adaxial/abaxial pattern formation;post-embryonic organ developm
LOC_Os02¦na
LOC_Os02¦na
LOC_Os02¦translation
LOC_Os02¦na
LOC_Os02¦translation
LOC_Os02¦na
LOC_Os02¦na
LOC_Os02¦na
LOC_Os02¦regulation of transcription, DNA-dependent
LOC_Os02¦na
LOC_Os02¦glycogen biosynthetic process;metabolic process;biosynthetic process;starch bi
LOC_Os02¦na
LOC_Os02¦rRNA processing;tRNA metabolic process;glycerol ether metabolic process;plas
LOC_Os02¦na
LOC_Os02¦na
LOC_Os02¦na
LOC_Os02¦purine nucleotide biosynthetic process;mRNA export from nucleus;protein ami
LOC_Os02¦carbohydrate metabolic process;glycogen biosynthetic process;starch metaboli
LOC_Os02¦na
LOC_Os02¦positive regulation of gene expression;pollen tube guidance;embryonic develo
LOC_Os02¦translation
LOC_Os02¦translation
LOC_Os02¦na
LOC_Os02¦transport;protein transport;protein import into mitochondrial inner membrane
LOC_Os02¦type I hypersensitivity
LOC_Os02¦na
LOC_Os02¦na
LOC_Os02¦purine nucleotide biosynthetic process;'de novo' pyrimidine base biosynthetic ¦
LOC_Os02¦embryonic development
LOC_Os02¦translation;response to cold
LOC_Os02¦RNA elongation;translation;pyrimidine ribonucleotide biosynthetic process
LOC_Os06¦response to stress;response to virus;response to hydrogen peroxide;response t

Do not distribute

LOC_Os06	na
LOC_Os02	na
LOC_Os02	na
LOC_Os02	na
LOC_Os02	G2 phase of mitotic cell cycle;protein import into nucleus;photomorphogenesis
LOC_Os02	RNA methylation;chromatin assembly or disassembly;protein import into nucle
LOC_Os02	translation
LOC_Os02	translation
LOC_Os06	response to stress
LOC_Os02	na
LOC_Os02	cysteine biosynthetic process from serine
LOC_Os02	transcription, DNA-dependent;regulation of transcription, DNA-dependent
LOC_Os02	proteolysis
LOC_Os02	cysteine biosynthetic process from serine;aging;cellular amino acid biosyntheti
LOC_Os02	na
LOC_Os06	response to stress;response to heat;response to cadmium ion
LOC_Os06	response to stress;response to virus;response to heat;response to cadmium ior
LOC_Os02	translation
LOC_Os02	transcription, DNA-dependent;pollen tube growth
LOC_Os06	response to stress;response to virus;response to heat;response to bacterium;re
LOC_Os02	RNA elongation;translation
LOC_Os02	na
LOC_Os02	protein folding;cellular protein metabolic process;G2 phase of mitotic cell cycle
LOC_Os02	na
LOC_Os06	protein folding;response to heat
LOC_Os02	na
LOC_Os02	G2 phase of mitotic cell cycle;endonucleolytic cleavages during rRNA processin
LOC_Os02	na
LOC_Os02	na
LOC_Os02	na
LOC_Os02	protein folding;cellular protein metabolic process
LOC_Os02	na
LOC_Os02	na
LOC_Os02	type I hypersensitivity
LOC_Os02	na
LOC_Os02	na
LOC_Os02	fatty acid biosynthetic process
LOC_Os02	na
LOC_Os02	na
LOC_Os06	protein amino acid phosphorylation;phosphorylation
LOC_Os02	regulation of transcription, DNA-dependent
LOC_Os02	na
LOC_Os02	na
LOC_Os02	endonucleolytic cleavages during rRNA processing;RNA methylation;protein im
LOC_Os02	na
LOC_Os02	RNA methylation;rRNA processing;RNA processing;mRNA processing;protein in

Do not distribute

LOC_Os02¦intracellular protein transport
LOC_Os02¦na
LOC_Os02¦na
LOC_Os02¦protein folding;cellular protein metabolic process;response to cadmium ion
LOC_Os02¦G2 phase of mitotic cell cycle;karyogamy;protein import into nucleus;amine me
LOC_Os02¦transcription, DNA-dependent;regulation of transcription, DNA-dependent;resp
LOC_Os02¦na
LOC_Os02¦regulation of transcription, DNA-dependent
LOC_Os02¦translation
LOC_Os02¦na
LOC_Os02¦na
LOC_Os02¦translation
LOC_Os02¦G2 phase of mitotic cell cycle;pyrimidine ribonucleotide biosynthetic process;pl
LOC_Os02¦na
LOC_Os02¦purine nucleotide biosynthetic process;pyrimidine ribonucleotide biosynthetic
LOC_Os02¦RNA methylation;pyrimidine ribonucleotide biosynthetic process;floral meriste
LOC_Os02¦transmembrane transport
LOC_Os02¦na
LOC_Os02¦carbohydrate metabolic process;starch metabolic process;cellular response to
LOC_Os02¦negative regulation of catalytic activity
LOC_Os02¦translation
LOC_Os02¦photosystem II assembly
LOC_Os02¦amino acid transmembrane transport
LOC_Os06¦protein amino acid glycosylation;lipid transport
LOC_Os06¦lipid transport
LOC_Os02¦na
LOC_Os02¦na
LOC_Os02¦translational elongation
LOC_Os02¦na
LOC_Os02¦transport
LOC_Os02¦lipid metabolic process
LOC_Os02¦na
LOC_Os02¦translation
LOC_Os02¦translation
LOC_Os02¦translation
LOC_Os02¦na
LOC_Os02¦DNA duplex unwinding
LOC_Os02¦na
LOC_Os02¦GTP catabolic process;translational initiation
LOC_Os02¦regulation of transcription, DNA-dependent
LOC_Os02¦na
LOC_Os02¦translation
LOC_Os02¦rRNA processing;tRNA metabolic process;aromatic amino acid family biosynthe
LOC_Os02¦na
LOC_Os02¦translation
LOC_Os02¦response to stress;mitochondrion organization;response to salt stress;response

Do not distribute

LOC_Os02 transcription, DNA-dependent;regulation of transcription, DNA-dependent;mul
LOC_Os02 translation
LOC_Os02 protein amino acid phosphorylation;phosphorylation
LOC_Os02 GTP catabolic process;translational initiation
LOC_Os02 na
LOC_Os02 transcription, DNA-dependent;regulation of transcription, DNA-dependent
LOC_Os02 karyogamy;RNA methylation;chromatin silencing;translation;protein import int
LOC_Os02 translation
LOC_Os02 rRNA processing
LOC_Os06 na
LOC_Os06 root development
LOC_Os02 na
LOC_Os02 regulation of transcription, DNA-dependent
LOC_Os02 G2 phase of mitotic cell cycle;fatty acid beta-oxidation;metabolic process;pyrim
LOC_Os02 defense response
LOC_Os02 translation
LOC_Os02 regulation of transcription, DNA-dependent
LOC_Os02 RNA methylation;protein targeting to mitochondrion;ribosome biogenesis;ribo
LOC_Os02 na
LOC_Os02 na
LOC_Os02 na
LOC_Os02 translational elongation
LOC_Os02 transcription, DNA-dependent;regulation of transcription, DNA-dependent;pro
LOC_Os06 proteolysis;ubiquitin-dependent protein catabolic process
LOC_Os02 regulation of abscisic acid mediated signaling pathway;maintenance of seed do
LOC_Os06 carbohydrate metabolic process
LOC_Os02 heat acclimation
LOC_Os02 translation
LOC_Os06 lipid transport
LOC_Os02 na
LOC_Os02 negative regulation of peptidase activity;defense response
LOC_Os02 na
LOC_Os02 methylation
LOC_Os02 translation
LOC_Os02 translation
LOC_Os02 na
LOC_Os02 translation
LOC_Os02 protein amino acid methylation;methylation;transcription, DNA-dependent;reg
LOC_Os02 na
LOC_Os02 na
LOC_Os02 na
LOC_Os02 translation
LOC_Os02 response to ethylene stimulus;peptidyl-pyroglutamic acid biosynthetic process,
LOC_Os02 na
LOC_Os02 na
LOC_Os06 response to oxidative stress;small GTPase mediated signal transduction;respon

Do not distribute

LOC_Os02	na
LOC_Os02	na
LOC_Os02	na
LOC_Os02	na
LOC_Os02	na
LOC_Os02	na
LOC_Os02	proton transport
LOC_Os02	purine nucleotide biosynthetic process;cellular amino acid metabolic process;c
LOC_Os02	na
LOC_Os02	metabolic process;leaf development
LOC_Os02	na
LOC_Os02	translation
LOC_Os02	cysteine biosynthetic process from serine;cellular amino acid biosynthetic proc
LOC_Os02	cysteine biosynthetic process from serine
LOC_Os06	ubiquitin-dependent protein catabolic process;protein targeting to vacuole;Gol
LOC_Os02	transcription, DNA-dependent
LOC_Os02	na
LOC_Os02	na
LOC_Os02	cysteinyl-tRNA aminoacylation;response to cadmium ion
LOC_Os02	cysteine biosynthetic process from serine
LOC_Os02	tRNA aminoacylation for protein translation;cysteinyl-tRNA aminoacylation;chl
LOC_Os02	na
LOC_Os02	cysteine biosynthetic process from serine;cyanide catabolic process
LOC_Os02	cysteine biosynthetic process from serine;cysteine homeostasis
LOC_Os02	na
LOC_Os02	na
LOC_Os06	lipid transport
LOC_Os02	GTP catabolic process;brassinosteroid mediated signaling pathway;mRNA modi
LOC_Os02	cysteine biosynthetic process from serine;cellular amino acid biosynthetic proc
LOC_Os02	chloroplast organization;positive regulation of transcription, DNA-dependent
LOC_Os02	translation;response to abiotic stimulus;response to salt stress
LOC_Os02	translation;response to abiotic stimulus;response to salt stress
LOC_Os02	histone peptidyl-prolyl isomerization;protein folding;peptidyl-proline modificat
LOC_Os06	na
LOC_Os02	na
LOC_Os02	response to cadmium ion
LOC_Os02	regulation of transcription, DNA-dependent;response to cold;response to wate
LOC_Os02	na
LOC_Os06	na
LOC_Os02	endonucleolytic cleavages during rRNA processing;pyrimidine ribonucleotide bi
LOC_Os02	na
LOC_Os06	endocytosis;response to salt stress;phosphorylation;phosphoinositide phospho
LOC_Os02	na
LOC_Os02	na
LOC_Os06	intracellular protein transport
LOC_Os06	intracellular protein transport

Do not distribute

LOC_Os02	regulation of transcription, DNA-dependent
LOC_Os02	na
LOC_Os02	RNA methylation;mRNA export from nucleus;protein import into nucleus
LOC_Os02	na
LOC_Os02	regulation of transcription, DNA-dependent
LOC_Os06	protein amino acid glycosylation;signal transduction
LOC_Os02	GTP catabolic process;translation;translational elongation
LOC_Os02	na
LOC_Os02	na
LOC_Os02	na
LOC_Os02	nucleosome assembly;response to water deprivation
LOC_Os02	na
LOC_Os02	oxidation reduction
LOC_Os02	RNA processing
LOC_Os02	pentose-phosphate shunt;rRNA processing;unsaturated fatty acid biosynthetic
LOC_Os02	RNA methylation;GTP catabolic process;maintenance of inflorescence meristen
LOC_Os06	intracellular protein transport;vesicle-mediated transport
LOC_Os06	na
LOC_Os06	dephosphorylation;peptidyl-tyrosine dephosphorylation
LOC_Os02	RNA methylation;protein targeting to mitochondrion
LOC_Os02	malate metabolic process;oxidation reduction
LOC_Os02	na
LOC_Os06	na
LOC_Os02	regulation of transcription, DNA-dependent
LOC_Os02	na
LOC_Os02	na
LOC_Os06	carbohydrate metabolic process
LOC_Os02	purine ribonucleoside salvage;AMP biosynthetic process;phosphorylation;respo
LOC_Os02	maltose metabolic process;sulfur metabolic process;methylglyoxal catabolic pr
LOC_Os02	na
LOC_Os02	response to high light intensity;cell differentiation;oxidation reduction
LOC_Os02	inositol biosynthetic process;sulfur metabolic process;response to cold;myo-inc
LOC_Os02	translation
LOC_Os02	na
LOC_Os02	isoprenoid biosynthetic process;isopentenyl diphosphate biosynthetic process,
LOC_Os02	lipid metabolic process
LOC_Os02	na
LOC_Os02	pyruvate metabolic process;metabolic process
LOC_Os02	translational elongation;response to cold;response to salt stress;response to zi
LOC_Os02	na
LOC_Os06	ATP catabolic process;transport;transmembrane transport;arsenite transport;r
LOC_Os02	proteolysis;negative regulation of catalytic activity
LOC_Os02	fatty acid catabolic process;response to salt stress;response to abscisic acid stir
LOC_Os02	oxidation reduction
LOC_Os02	DNA methylation on cytosine;centromeric heterochromatin assembly;DNA met
LOC_Os02	spliceosomal snRNP assembly;nuclear mRNA 5'-splice site recognition;nuclear r

Do not distribute

LOC_Os02; na
LOC_Os02; translation
LOC_Os02; nuclear mRNA splicing, via spliceosome;chromosome segregation;sister chroma
LOC_Os02; translation
LOC_Os06; na
LOC_Os06; carbohydrate metabolic process
LOC_Os02; na
LOC_Os02; na
LOC_Os02; RNA methylation;purine nucleotide biosynthetic process;glutamine catabolic pr
LOC_Os02; na
LOC_Os02; cysteine metabolic process;metabolic process;iron-sulfur cluster assembly
LOC_Os02; na
LOC_Os02; ribosome biogenesis;translational elongation;response to cold;response to salt
LOC_Os06; transcription, DNA-dependent;regulation of transcription, DNA-dependent;resp
LOC_Os06; phosphatidylinositol metabolic process;phosphoinositide phosphorylation;phos
LOC_Os02; protein folding
LOC_Os02; translation
LOC_Os02; aromatic amino acid family biosynthetic process;chorismate metabolic process
LOC_Os02; ribosome biogenesis;translational elongation;response to cold;response to salt
LOC_Os06; DNA repair
LOC_Os02; na
LOC_Os02; na
LOC_Os02; na
LOC_Os02; root hair cell tip growth;response to cytokinin stimulus;root epidermal cell diffe
LOC_Os02; nucleosome assembly;cell proliferation;lateral root formation;cell differentiatic
LOC_Os02; root hair cell tip growth;response to salt stress
LOC_Os02; glycogen biosynthetic process;metabolic process;biosynthetic process;starch bi
LOC_Os02; na
LOC_Os02; endonucleolytic cleavages during rRNA processing;RNA methylation;rRNA proc
LOC_Os02; protein folding;response to stress;defense response;response to heat;response
LOC_Os02; protein folding;response to stress;defense response;response to heat;response
LOC_Os02; ubiquitin-dependent protein catabolic process;response to red or far red light;e
LOC_Os02; protein folding;response to stress;defense response;response to heat;response
LOC_Os02; cytidine to uridine editing;RNA modification
LOC_Os02; RNA elongation;translation;pyrimidine ribonucleotide biosynthetic process;ribc
LOC_Os02; DNA replication;DNA repair;proteolysis;DNA duplex unwinding
LOC_Os02; na
LOC_Os02; na
LOC_Os02; tryptophan biosynthetic process;tryptophan metabolic process;metabolic proc
LOC_Os02; na
LOC_Os02; translation
LOC_Os06; mitotic cell cycle;thigmotropism;vegetative to reproductive phase transition of
LOC_Os02; na
LOC_Os02; na
LOC_Os02; na
LOC_Os02; na

Do not distribute

LOC_Os02 RNA methylation;translation
LOC_Os02 nuclear mRNA splicing, via spliceosome
LOC_Os02 iron-sulfur cluster assembly
LOC_Os02 na
LOC_Os06 na
LOC_Os02 protein import into nucleus, docking;gluconeogenesis;intracellular protein tran
LOC_Os06 cell redox homeostasis
LOC_Os06 na
LOC_Os02 na
LOC_Os02 ATP hydrolysis coupled proton transport;cellular response to nutrient levels;va
LOC_Os02 biosynthetic process
LOC_Os02 na
LOC_Os02 translation
LOC_Os02 purine nucleotide biosynthetic process;AMP biosynthetic process;'de novo' AM
LOC_Os02 translation
LOC_Os02 metabolic process;oxidation reduction
LOC_Os02 GTP catabolic process
LOC_Os02 na
LOC_Os02 translation;tRNA aminoacylation for protein translation;glutamyl-tRNA aminoa
LOC_Os02 iron-sulfur cluster assembly
LOC_Os02 protein folding;protein refolding;cellular protein metabolic process;response to
LOC_Os02 GTP catabolic process;intracellular protein transport
LOC_Os06 de-etiolation;response to cadmium ion;lateral root development;response to g
LOC_Os02 translation
LOC_Os02 na
LOC_Os02 chloride transport;cell volume homeostasis
LOC_Os02 na
LOC_Os02 na
LOC_Os02 na
LOC_Os02 tRNA aminoacylation for protein translation;prolyl-tRNA aminoacylation
LOC_Os02 phosphatidylglycerol biosynthetic process;aromatic amino acid family biosynth
LOC_Os02 type I hypersensitivity
LOC_Os02 na
LOC_Os06 endocytosis;response to salt stress;phosphorylation;phosphoinositide phospho
LOC_Os02 pyrimidine ribonucleotide biosynthetic process
LOC_Os06 carbohydrate metabolic process;mannose metabolic process
LOC_Os02 transcription, DNA-dependent;regulation of transcription, DNA-dependent;aux
LOC_Os02 na
LOC_Os02 transcription, DNA-dependent;regulation of transcription, DNA-dependent;aux
LOC_Os06 oxidation reduction
LOC_Os02 na
LOC_Os02 metabolic process
LOC_Os02 tricarboxylic acid cycle;metabolic process;response to cadmium ion
LOC_Os02 pentose-phosphate shunt;RNA elongation;translation;aromatic amino acid fam
LOC_Os02 RNA elongation;translation
LOC_Os06 phosphorylation;phosphatidylinositol metabolic process;phosphoinositide pho

Do not distribute

LOC_Os02	RNA processing
LOC_Os02	glucose catabolic process;metabolic process;response to cold;response to cadn
LOC_Os02	type I hypersensitivity
LOC_Os02	na
LOC_Os02	ribosome biogenesis
LOC_Os06	vesicle-mediated transport
LOC_Os02	regulation of anatomical structure morphogenesis;oxidation reduction
LOC_Os02	microtubule cytoskeleton organization;cytokinesis by cell plate formation;prote
LOC_Os02	red or far-red light signaling pathway
LOC_Os02	purine nucleotide biosynthetic process;pyrimidine ribonucleotide biosynthetic
LOC_Os02	na
LOC_Os02	na
LOC_Os02	na
LOC_Os02	protein targeting to mitochondrion;photomorphogenesis;embryonic developm
LOC_Os02	G2 phase of mitotic cell cycle;RNA methylation;gluconeogenesis;protein folding
LOC_Os02	translation
LOC_Os02	na
LOC_Os06	protein targeting to vacuole;intracellular protein transport;gravitropism;vesicle
LOC_Os02	protein amino acid methylation
LOC_Os02	na
LOC_Os02	translation;translational elongation;response to zinc ion;response to cadmium
LOC_Os02	seed oilbody biogenesis;response to freezing;lipid storage;seed germination
LOC_Os06	phosphatidylinositol metabolic process;pollen germination;pollen tube growth;
LOC_Os02	translation
LOC_Os02	na
LOC_Os02	na
LOC_Os02	metabolic process
LOC_Os02	ATP catabolic process;protein folding;response to heat;response to high light ir
LOC_Os06	intracellular protein transport
LOC_Os02	arginine biosynthetic process
LOC_Os02	nucleosome assembly;DNA mediated transformation;response to wounding;re:
LOC_Os06	endocytosis;establishment of tissue polarity;plant-type cell wall modification;p
LOC_Os02	two-component signal transduction system (phosphorelay);regulation of transc
LOC_Os02	na
LOC_Os02	glucose catabolic process;metabolic process
LOC_Os02	na
LOC_Os02	proteolysis
LOC_Os02	biosynthetic process;glucan biosynthetic process;starch biosynthetic process
LOC_Os02	na
LOC_Os02	proteolysis;glucosinolate biosynthetic process
LOC_Os02	na
LOC_Os02	regulation of cell shape;biosynthetic process;cell division;rRNA processing;tRN/
LOC_Os02	glucose catabolic process;metabolic process;response to cold;response to cadn
LOC_Os02	na
LOC_Os02	protein metabolic process
LOC_Os02	ubiquitin-dependent protein catabolic process;response to red or far red light;

Do not distribute

LOC_Os02| na
LOC_Os02| na
LOC_Os02| protein folding;response to stress;defense response to bacterium, incompatible
LOC_Os02| developmental growth;root hair elongation
LOC_Os02| fatty acid catabolic process
LOC_Os02| root epidermal cell differentiation;root hair elongation;response to light stimul
LOC_Os02| na
LOC_Os02| na
LOC_Os02| ubiquitin-dependent protein catabolic process;response to red or far red light;
LOC_Os02| dUTP metabolic process;dUMP biosynthetic process;DNA-dependent DNA repli
LOC_Os02| translation;tRNA aminoacylation for protein translation;arginyl-tRNA aminoacy
LOC_Os02| carbohydrate metabolic process;defense response
LOC_Os06| na
LOC_Os02| na
LOC_Os06| trehalose metabolic process;trehalose catabolic process;response to stress;me
LOC_Os02| tRNA aminoacylation for protein translation;glycyl-tRNA aminoacylation;respor
LOC_Os02| na
LOC_Os02| translation;response to UV-B;developmental process;cellular response to UV-B
LOC_Os02| na
LOC_Os06| response to nitrate;Golgi vesicle transport
LOC_Os02| rRNA processing;nucleotide biosynthetic process;pyrimidine ribonucleotide bio
LOC_Os02| ATP catabolic process;response to heat;chloroplast organization;protein proces
LOC_Os02| protein metabolic process;response to stress;cellular response to heat;protein
LOC_Os02| na
LOC_Os02| na
LOC_Os02| na
LOC_Os06| response to starvation
LOC_Os06| reproductive developmental process;phosphorylation;growth;phosphatidylinos
LOC_Os02| endonucleolytic cleavages during rRNA processing;rRNA processing;leaf vascula
LOC_Os02| na
LOC_Os06| na
LOC_Os02| na
LOC_Os06| metabolic process;L-phenylalanine biosynthetic process
LOC_Os02| na
LOC_Os02| seed oilbody biogenesis;response to freezing;lipid storage;seed germination
LOC_Os02| nuclear mRNA splicing, via spliceosome;rRNA processing;ribosome biogenesis;
LOC_Os02| endonucleolytic cleavages during rRNA processing;rRNA processing;pyrimidine
LOC_Os02| formation of translation preinitiation complex;translation;translational initiatio
LOC_Os02| RNA splicing
LOC_Os02| na
LOC_Os02| na
LOC_Os02| na
LOC_Os02| na
LOC_Os02| na
LOC_Os02| transcription, DNA-dependent
LOC_Os02| na

Do not distribute

LOC_Os02g na
LOC_Os02g na
LOC_Os02g na
LOC_Os06g phosphorylation;phosphatidylinositol metabolic process;phosphoinositide pho:
LOC_Os02g na
LOC_Os02g carbohydrate metabolic process;glycerol-3-phosphate metabolic process;glyce
LOC_Os02g pyrimidine ribonucleotide biosynthetic process
LOC_Os06g na
LOC_Os02g na
LOC_Os02g G2 phase of mitotic cell cycle;protein targeting to mitochondrion;photomorph
LOC_Os02g transcription, DNA-dependent
LOC_Os02g mitotic cell cycle;double-strand break repair;DNA recombination;mitotic recom
LOC_Os02g na
LOC_Os02g na
LOC_Os02g na
LOC_Os02g translation;endonucleolytic cleavage in ITS1 to separate SSU-rRNA from 5.8S rR
LOC_Os06g na
LOC_Os02g reproduction;G2 phase of mitotic cell cycle;protein deneddylation;chromatin o
LOC_Os02g translation
LOC_Os02g na
LOC_Os02g na
LOC_Os02g na
LOC_Os02g na
LOC_Os02g na
LOC_Os02g na
LOC_Os02g na
LOC_Os02g RNA elongation;translation
LOC_Os02g branched chain family amino acid biosynthetic process;cellular amino acid bios
LOC_Os06g transport;water transport
LOC_Os02g na
LOC_Os02g protein folding
LOC_Os02g na
LOC_Os02g na
LOC_Os06g transport;silicate transport
LOC_Os02g na
LOC_Os02g tRNA aminoacylation for protein translation;threonyl-tRNA aminoacylation;tRN
LOC_Os02g isoprenoid biosynthetic process;photosynthesis;ubiquinone biosynthetic proce
LOC_Os02g na
LOC_Os02g DNA methylation;transcription, DNA-dependent;production of siRNA involved i
LOC_Os02g methylation;regulation of RNA metabolic process
LOC_Os02g maltose metabolic process;proteolysis;starch biosynthetic process
LOC_Os06g xylan catabolic process;L-arabinose metabolic process
LOC_Os02g ubiquitin-dependent protein catabolic process;photorespiration;response to m
LOC_Os02g methylation;regulation of RNA metabolic process
LOC_Os02g tRNA aminoacylation for protein translation;phenylalanyl-tRNA aminoacylation
LOC_Os02g defense response

Do not distribute

LOC_Os02| phenylalanyl-tRNA aminoacylation;tRNA aminoacylation
LOC_Os02| G2 phase of mitotic cell cycle;protein deneddylation;response to auxin stimulus
LOC_Os02| na
LOC_Os06| GTP catabolic process;intracellular protein transport;ER to Golgi vesicle-mediat
LOC_Os02| protein folding
LOC_Os02| gluconeogenesis;pyrimidine nucleotide biosynthetic process;chromatin silencin
LOC_Os02| mRNA export from nucleus;protein import into nucleus
LOC_Os02| na
LOC_Os02| na
LOC_Os02| rRNA processing
LOC_Os02| nucleoside diphosphate phosphorylation;GTP biosynthetic process;UTP biosynt
LOC_Os02| translation
LOC_Os02| rRNA processing;translation;photosystem II assembly;chlorophyll biosynthetic p
LOC_Os02| ATP catabolic process
LOC_Os02| sucrose metabolic process;response to osmotic stress;biosynthetic process;resp
LOC_Os02| DNA metabolic process;DNA repair;DNA recombination;response to DNA dama
LOC_Os02| na
LOC_Os06| protein amino acid phosphorylation;signal transduction;phosphorylation;cytoki
LOC_Os02| na
LOC_Os02| oxidation reduction
LOC_Os02| embryo sac development;polar nucleus fusion
LOC_Os02| na
LOC_Os02| translation
LOC_Os02| na
LOC_Os02| translation
LOC_Os02| translation;isopentenyl diphosphate biosynthetic process, mevalonate-indeper
LOC_Os06| D-xylose metabolic process;cellular metabolic process
LOC_Os02| transcription, DNA-dependent;regulation of transcription, DNA-dependent
LOC_Os02| karyogamy;protein targeting to mitochondrion;embryo sac egg cell differentiat
LOC_Os02| GTP catabolic process;translational elongation;response to cadmium ion;transl
LOC_Os02| regulation of transcription, DNA-dependent
LOC_Os02| na
LOC_Os02| purine nucleotide biosynthetic process;pyrimidine ribonucleotide biosynthetic
LOC_Os02| oxidation reduction
LOC_Os02| rRNA processing;translation;chloroplast relocation;thylakoid membrane organi
LOC_Os02| protein amino acid glycosylation;apoptosis;negative regulation of programmed
LOC_Os02| transmembrane transport
LOC_Os02| ATP catabolic process;protein folding;response to stress;response to heat;resp
LOC_Os02| DNA repair;nucleotide-excision repair;nucleosome assembly;DNA mediated tra
LOC_Os02| transcription, DNA-dependent;embryo sac egg cell differentiation
LOC_Os02| na
LOC_Os02| DNA replication
LOC_Os02| GTP catabolic process;translational elongation;very-long-chain fatty acid metab
LOC_Os02| polysaccharide biosynthetic process;polysaccharide catabolic process;starch m
LOC_Os02| protein metabolic process
LOC_Os02| regulation of RNA metabolic process;methylation

Do not distribute

LOC_Os02	folic acid and derivative biosynthetic process;oxidation reduction
LOC_Os02	response to salt stress;anther dehiscence;protein homotrimerization
LOC_Os02	formation of translation preinitiation complex;translation;translational initiatio
LOC_Os02	na
LOC_Os02	response to salt stress;anther dehiscence
LOC_Os02	response to high light intensity;cell differentiation;oxidation reduction
LOC_Os02	response to cold;defense response to bacterium;oxidation reduction;hydrogen
LOC_Os02	regulation of nitrogen utilization;tRNA processing;oxidation reduction
LOC_Os02	protein folding;response to cold;response to cadmium ion
LOC_Os02	sister chromatid cohesion;reciprocal meiotic recombination;embryonic develop
LOC_Os06	na
LOC_Os02	na
LOC_Os02	negative regulation of catalytic activity
LOC_Os02	mRNA export from nucleus;protein import into nucleus;nucleotide biosynthetic
LOC_Os02	'de novo' IMP biosynthetic process;purine nucleotide biosynthetic process;ubic
LOC_Os02	na
LOC_Os02	DNA replication;DNA repair;DNA recombination;response to DNA damage stim
LOC_Os02	gluconeogenesis;glycolysis;pentose-phosphate shunt;ubiquitin-dependent prot
LOC_Os02	GTP catabolic process;translational elongation;response to cold
LOC_Os02	na
LOC_Os02	translation
LOC_Os02	DNA replication
LOC_Os02	RNA splicing, via endonucleolytic cleavage and ligation;methionine biosynthetic
LOC_Os02	maltose metabolic process;unsaturated fatty acid biosynthetic process;phosph
LOC_Os02	na
LOC_Os02	embryonic development ending in seed dormancy
LOC_Os06	na
LOC_Os02	tRNA modification;protein targeting to mitochondrion;nucleotide biosynthetic
LOC_Os02	GTP catabolic process;translational elongation;maltose metabolic process;pent
LOC_Os02	na
LOC_Os02	DNA methylation;chromatin silencing;defense response to bacterium, incompa
LOC_Os02	translation
LOC_Os02	nucleosome assembly
LOC_Os02	G2 phase of mitotic cell cycle;formation of translation preinitiation complex;tra
LOC_Os02	asparagine biosynthetic process;glutamine metabolic process;metabolic proces
LOC_Os02	glycolysis;glycine catabolic process;tryptophan catabolic process;triglyceride m
LOC_Os02	translation
LOC_Os02	glycolysis;metabolic process;response to salt stress;response to zinc ion;cellula
LOC_Os02	ornithine metabolic process;response to jasmonic acid stimulus
LOC_Os02	na
LOC_Os02	na
LOC_Os02	na
LOC_Os02	biosynthetic process;methylglyoxal catabolic process to D-lactate;photoperiodi
LOC_Os02	na
LOC_Os02	regulation of transcription, DNA-dependent;transcription, DNA-dependent;mul
LOC_Os02	na

Do not distribute

LOC_Os02 lipid metabolic process;lipid catabolic process
LOC_Os02 translation;isopentenyl diphosphate biosynthetic process, mevalonate-indeper
LOC_Os02 metabolic process;nucleotide biosynthetic process;methylation;peptidyl-arginii
LOC_Os02 formation of translation preinitiation complex;translation;translational initiatio
LOC_Os02 na
LOC_Os02 na
LOC_Os02 na
LOC_Os02 na
LOC_Os02 na
LOC_Os02 na
LOC_Os02 na
LOC_Os02 na
LOC_Os02 na
LOC_Os02 na
LOC_Os02 na
LOC_Os02 na
LOC_Os02 na
LOC_Os02 na
LOC_Os02 protein maturation
LOC_Os02 na
LOC_Os02 na
LOC_Os02 na
LOC_Os02 na
LOC_Os02 na
LOC_Os02 na
LOC_Os02 na
LOC_Os02 na
LOC_Os02 na
LOC_Os02 trehalose biosynthetic process;response to stress;metabolic process;response t
LOC_Os02 translation;tRNA aminoacylation for protein translation;tRNA aminoacylation;g
LOC_Os02 GTP catabolic process
LOC_Os02 protein folding;response to cold;response to cadmium ion
LOC_Os02 response to salt stress
LOC_Os02 na
LOC_Os02 na
LOC_Os02 na
LOC_Os02 na
LOC_Os02 fructose 6-phosphate metabolic process;glycolysis;embryonic development enc
LOC_Os02 pyruvate metabolic process;metabolic process
LOC_Os02 pyruvate metabolic process;metabolic process
LOC_Os02 metabolic process
LOC_Os02 pyruvate metabolic process;metabolic process
LOC_Os02 petal formation;sepal formation
LOC_Os02 protein metabolic process
LOC_Os02 small GTPase mediated signal transduction;protein transport;early endosome t
LOC_Os02 DNA replication;regulation of DNA replication;error-prone translesion synthesi

Do not distribute

LOC_Os02	glycolysis;ubiquitin-dependent protein catabolic process;aerobic respiration;ph
LOC_Os02	multicellular organismal development;chromatin modification;pollen sperm ce
LOC_Os02	na
LOC_Os02	na
LOC_Os02	na
LOC_Os02	na
LOC_Os02	na
LOC_Os02	protein insertion into membrane;protein import into chloroplast thylakoid mer
LOC_Os02	na
LOC_Os06	proteolysis;negative regulation of catalytic activity
LOC_Os02	pyrimidine ribonucleotide biosynthetic process;transcription factor import into
LOC_Os02	nucleoside diphosphate phosphorylation;GTP biosynthetic process;UTP biosynt
LOC_Os02	na
LOC_Os02	metabolic process;response to cytokinin stimulus
LOC_Os02	rRNA processing;tRNA metabolic process;tRNA aminoacylation for protein tran
LOC_Os02	nucleosome assembly;DNA mediated transformation;response to wounding;re
LOC_Os02	karyogamy;RNA methylation;protein amino acid methylation;protein import in
LOC_Os02	proline biosynthetic process;threonine catabolic process;cellular amino acid bi
LOC_Os02	pseudouridine synthesis;tRNA processing;RNA modification
LOC_Os02	nucleoside diphosphate phosphorylation;GTP biosynthetic process;UTP biosynt
LOC_Os02	regulation of flower development;histone lysine methylation
LOC_Os02	na
LOC_Os02	translation;isopentenyl diphosphate biosynthetic process, mevalonate-indeper
LOC_Os02	cellular amino acid metabolic process;cellular amino acid biosynthetic process;
LOC_Os02	lipid metabolic process;sphingolipid metabolic process;biosynthetic process;re
LOC_Os02	na
LOC_Os02	na
LOC_Os06	metabolic process
LOC_Os02	nucleotide metabolic process;nucleoside triphosphate catabolic process;deoxy
LOC_Os02	lysine biosynthetic process via diaminopimelate;oxidation reduction;cellular an
LOC_Os02	protein folding
LOC_Os02	na
LOC_Os02	na
LOC_Os06	na
LOC_Os02	na
LOC_Os02	metabolic process
LOC_Os02	protein folding;response to stress;response to chlorate;protein import into chl
LOC_Os02	GTP catabolic process;microtubule-based process;protein polymerization;pyrim
LOC_Os02	na
LOC_Os02	purine nucleotide biosynthetic process;purine base biosynthetic process
LOC_Os02	na
LOC_Os02	translation
LOC_Os02	metabolic process;branched chain family amino acid biosynthetic process;pyrin
LOC_Os02	na
LOC_Os02	cellular amino acid metabolic process;cellular amino acid biosynthetic process;
LOC_Os02	pseudouridine synthesis;RNA processing;RNA modification

Do not distribute

LOC_Os02| na
LOC_Os06| plant-type cell wall cellulose metabolic process;cell wall pectin metabolic proce
LOC_Os06| proteolysis
LOC_Os02| metabolic process
LOC_Os02| protein hexamerization;ATP catabolic process;protein import into chloroplast s
LOC_Os02| DNA duplex unwinding;meristem development
LOC_Os02| na
LOC_Os02| translation;pattern specification process;regulation of cell size;plant-type cell w
LOC_Os02| nucleoside diphosphate phosphorylation;GTP biosynthetic process;UTP biosynt
LOC_Os02| na
LOC_Os02| RNA elongation;transport;protein secretion;thylakoid membrane organization;
LOC_Os02| gluconeogenesis;glycolysis;protein folding;response to salt stress;plant-type ce
LOC_Os02| na
LOC_Os06| na
LOC_Os02| transport;purine nucleotide transport;transmembrane transport
LOC_Os02| na
LOC_Os02| na
LOC_Os06| fatty acid biosynthetic process;photomorphogenesis;sphingoid biosynthetic pro
LOC_Os02| na
LOC_Os02| na
LOC_Os02| na
LOC_Os02| lipid metabolic process;sphingolipid metabolic process;biosynthetic process;cel
LOC_Os02| regulation of nitrogen utilization;tRNA processing;oxidation reduction
LOC_Os02| metabolic process;pyrimidine ribonucleotide biosynthetic process;peptidyl-dipl
LOC_Os02| embryo sac egg cell differentiation
LOC_Os02| translation
LOC_Os02| endonucleolytic cleavage of tetracistronic rRNA transcript (SSU-rRNA, LSU-rRNA
LOC_Os06| protein amino acid phosphorylation;photoperiodism, flowering;phosphorylatio
LOC_Os02| gluconeogenesis;glycolysis;protein targeting to vacuole;response to salt stress;
LOC_Os02| transcription, DNA-dependent
LOC_Os02| arginine catabolic process;polyamine biosynthetic process;response to osmotic
LOC_Os02| chromatin assembly or disassembly;protein import into nucleus
LOC_Os02| arginine catabolic process;spermidine biosynthetic process;putrescine biosynth
LOC_Os02| nucleosome assembly;response to salt stress;response to ethylene stimulus;res
LOC_Os02| protein modification process;transcription factor import into nucleus
LOC_Os06| na
LOC_Os02| transcription, DNA-dependent;RNA elongation;regulation of transcription, DNA
LOC_Os02| translation
LOC_Os02| translation
LOC_Os02| nuclear-transcribed mRNA catabolic process
LOC_Os02| na
LOC_Os02| DNA replication;deoxyribonucleoside triphosphate biosynthetic process;respor
LOC_Os02| protein targeting to mitochondrion;embryo sac egg cell differentiation
LOC_Os02| na
LOC_Os02| na
LOC_Os02| nuclear mRNA splicing, via spliceosome;RNA methylation;response to cold

Do not distribute

Do not distribute

LOC_Os06	transcription, DNA-dependent;regulation of transcription, DNA-dependent;neg
LOC_Os02	transcription, DNA-dependent
LOC_Os02	na
LOC_Os02	na
LOC_Os02	na
LOC_Os02	response to stress
LOC_Os02	purine nucleotide biosynthetic process;'de novo' IMP biosynthetic process;toxi
LOC_Os02	na
LOC_Os06	metabolic process
LOC_Os02	na
LOC_Os02	hydrogen peroxide catabolic process;oxidation reduction
LOC_Os02	na
LOC_Os02	na
LOC_Os02	proteolysis;isopentenyl diphosphate biosynthetic process, mevalonate-indepe
LOC_Os02	translation
LOC_Os02	lipid metabolic process;sphingolipid metabolic process;biosynthetic process;ce
LOC_Os02	tRNA aminoacylation for protein translation;seryl-tRNA aminoacylation;respon
LOC_Os06	na
LOC_Os02	na
LOC_Os02	na
LOC_Os02	nucleosome assembly
LOC_Os02	nucleosome assembly
LOC_Os02	na
LOC_Os02	translation;protein targeting to mitochondrion
LOC_Os02	RNA methylation;rRNA processing;methylation
LOC_Os02	translation;ribosome biogenesis
LOC_Os02	negative regulation of transcription factor activity;protein sumoylation;respons
LOC_Os06	transcription, DNA-dependent;regulation of transcription, DNA-dependent
LOC_Os02	organ growth;regulation of cell proliferation;regulation of cell cycle
LOC_Os02	translation
LOC_Os02	na
LOC_Os02	lignan biosynthetic process
LOC_Os02	pyridoxine biosynthetic process;pyridoxal phosphate biosynthetic process;oxid
LOC_Os02	GTP catabolic process;translational elongation
LOC_Os02	purine ribonucleoside salvage;AMP biosynthetic process;response to cadmium
LOC_Os02	pyrimidine ribonucleotide biosynthetic process;embryonic development endin
LOC_Os02	tRNA aminoacylation for protein translation;seryl-tRNA aminoacylation
LOC_Os06	tubulin complex assembly
LOC_Os02	response to salicylic acid stimulus;response to ethylene stimulus;response to s
LOC_Os06	amine metabolic process;oxidation reduction
LOC_Os02	metabolic process
LOC_Os02	na
LOC_Os02	fatty acid biosynthetic process;defense response to fungus, incompatible inter
LOC_Os02	fatty acid biosynthetic process;defense response to fungus, incompatible inter
LOC_Os02	phosphate metabolic process;defense response to bacterium;response to salt s
LOC_Os02	cell redox homeostasis;oxidation reduction

LOC_Os02¦oxidation reduction;response to stress
LOC_Os06¦metabolic process;positive regulation of seed germination;response to nitrate;
LOC_Os02¦negative regulation of catalytic activity
LOC_Os02¦RNA methylation;ATP catabolic process;DNA replication;pyrimidine ribonucleot
LOC_Os02¦GTP catabolic process;translational elongation;response to cold
LOC_Os02¦protein import into nucleus
LOC_Os02¦metabolic process;branched chain family amino acid metabolic process
LOC_Os02¦histidine biosynthetic process;nucleotide biosynthetic process
LOC_Os02¦translation
LOC_Os02¦RNA elongation;translation
LOC_Os02¦DNA repair;base-excision repair
LOC_Os02¦RNA elongation;translation
LOC_Os02¦GTP catabolic process;microtubule-based process;protein polymerization;cellul
LOC_Os02¦transcription, DNA-dependent
LOC_Os02¦nicotianamine biosynthetic process;phloem transport;cellular response to iron
LOC_Os02¦proton-transporting ATP synthase complex assembly
LOC_Os02¦iron-sulfur cluster assembly
LOC_Os06¦na
LOC_Os06¦na
LOC_Os02¦carbohydrate metabolic process;polysaccharide catabolic process;metabolic pr
LOC_Os02¦translation
LOC_Os02¦tricarboxylic acid cycle;carbon fixation
LOC_Os02¦tricarboxylic acid cycle;carbon fixation
LOC_Os06¦na
LOC_Os02¦methylation
LOC_Os02¦na
LOC_Os02¦protein insertion into membrane;signal transduction;chloroplast organization;c
LOC_Os02¦na
LOC_Os02¦na
LOC_Os02¦pollen tube development
LOC_Os02¦pollen tube development
LOC_Os02¦IMP biosynthetic process;purine ribonucleoside monophosphate biosynthetic p
LOC_Os02¦seed development;acquisition of desiccation tolerance;response to abscisic aci
LOC_Os02¦na
LOC_Os02¦na
LOC_Os02¦response to stress;mitochondrion organization;response to salt stress;response
LOC_Os02¦metabolic process;branched chain family amino acid metabolic process
LOC_Os02¦na
LOC_Os02¦response to cold;stomatal lineage progression;response to freezing;positive reg
LOC_Os02¦translational elongation;response to cold
LOC_Os02¦translation
LOC_Os02¦tricarboxylic acid cycle;carbon fixation
LOC_Os02¦tricarboxylic acid cycle;carbon fixation;protein tetramerization;cellular respons
LOC_Os02¦tricarboxylic acid cycle;carbon fixation
LOC_Os02¦tricarboxylic acid cycle;carbon fixation
LOC_Os02¦protein folding;response to heat;protein refolding;cellular protein metabolic pr

Do not distribute

LOC_Os02 protein amino acid phosphorylation
LOC_Os02 transport
LOC_Os02 protein amino acid phosphorylation
LOC_Os02 protein amino acid phosphorylation;transport
LOC_Os02 protein amino acid phosphorylation;protein stabilization
LOC_Os02 protein amino acid phosphorylation;response to sucrose stimulus;flavonoid bic
LOC_Os02 protein amino acid phosphorylation;chlorophyll catabolic process
LOC_Os02 na
LOC_Os02 translation;translational elongation;response to cadmium ion
LOC_Os02 branched chain family amino acid biosynthetic process
LOC_Os02 rRNA processing;tRNA methylation;rRNA base methylation;RNA methylation
LOC_Os06 transcription, DNA-dependent;regulation of transcription, DNA-dependent;regu
LOC_Os02 na
LOC_Os02 RNA elongation;translation;ribosome biogenesis
LOC_Os02 proteolysis;cellular process;response to abscisic acid stimulus;dolichol biosynth
LOC_Os02 response to heat;response to high light intensity;response to hydrogen peroxid
LOC_Os02 na
LOC_Os02 na
LOC_Os02 GTP catabolic process;ribosome biogenesis
LOC_Os02 na
LOC_Os02 cellular amino acid metabolic process;cysteine biosynthetic process from serine
LOC_Os02 na
LOC_Os02 mitotic cell cycle;cytokinesis;protein amino acid phosphorylation;cellular respo
LOC_Os02 na
LOC_Os02 RNA methylation;RNA processing
LOC_Os02 na
LOC_Os02 protein import into mitochondrial matrix
LOC_Os06 na
LOC_Os02 translation
LOC_Os02 na
LOC_Os02 nucleosome assembly
LOC_Os02 na
LOC_Os02 embryo sac development
LOC_Os02 DNA repair;base-excision repair
LOC_Os02 folic acid and derivative metabolic process
LOC_Os02 nucleosome assembly
LOC_Os02 iron-sulfur cluster assembly
LOC_Os02 GTP catabolic process;translational elongation;response to cold
LOC_Os02 cell redox homeostasis;anther development
LOC_Os02 protein amino acid glycosylation
LOC_Os02 na
LOC_Os02 na
LOC_Os02 acetyl-CoA metabolic process;metabolic process;embryonic development endi
LOC_Os02 protein transport
LOC_Os02 RNA elongation;pyrimidine ribonucleotide biosynthetic process;protein secreti
LOC_Os02 na

Do not distribute

LOC_Os02	transcription, DNA-dependent;regulation of transcription, DNA-dependent
LOC_Os02	translation;maturation of SSU-rRNA from tricistronic rRNA transcript (SSU-rRNA
LOC_Os02	na
LOC_Os02	na
LOC_Os06	metabolic process;pollen tube growth
LOC_Os02	rRNA processing;embryonic development ending in seed dormancy;thylakoid m
LOC_Os06	regulation of transcription, DNA-dependent
LOC_Os02	na
LOC_Os02	arginine biosynthetic process;cellular amino acid biosynthetic process;response
LOC_Os02	na
LOC_Os02	microtubule-based process
LOC_Os06	two-component signal transduction system (phosphorelay);response to supero
LOC_Os02	mitochondrial proton-transporting ATP synthase complex assembly
LOC_Os02	na
LOC_Os02	na
LOC_Os02	na
LOC_Os02	na
LOC_Os02	metabolic process;spermidine biosynthetic process
LOC_Os02	metabolic process;suberin biosynthetic process
LOC_Os02	response to light intensity;regulation of gene expression
LOC_Os02	signal peptide processing
LOC_Os02	translation;inflorescence development;flower development;post-embryonic de
LOC_Os02	transcription termination;cation transport;regulation of growth

Do not distribute

orphogenesis
: intensity

nthetic process;response to cadmium ion

enesis
thetic process;CTP biosynthetic process;phosphorylation

Do not distribute

ine nucleotide biosynthetic process;protein import into nucleus;pyrimidine ribonucleotide

hotomorphogenesis;regulation of flower development;cullin deneddylation;histone lysine

hotomorphogenesis;regulation of flower development;cullin deneddylation;histone lysine

ɔsome biogenesis
orylation;phosphatidylinositol metabolic process;phosphoinositide phosphorylation
ıesis;root morphogenesis

: intensity

ent

Do not distribute

uration

ɪhotomorphogenesis;cullin deneddylation

ʌ, LSU-rRNA);translation;translational elongation

ɔf transcription, DNA-dependent;defense response;methionine biosynthetic process

in maturation

:ess;cysteine biosynthetic process;photosynthetic acclimation;regulation of hydrogen pero›
ɔnse to salt stress;mature ribosome assembly

;

:ch biosynthetic process

ɔrphogenesis

n folding;signal transduction;response to cadmium ion

Do not distribute

ort;protein import into mitochondrial inner membrane

tion

n;ribosome biogenesis

in RNA interference;gene silencing by RNA;posttranscriptional gene silencing by RNA;regul

e to cadmium ion

Do not distribute

Do not distribute

› sac development;cellularization of the embryo sac;embryo sac egg cell differentiation;pola

ion
tion
›hotomorphogenesis;cullin deneddylation

;cell wall repair

ninobenzoic acid metabolic process

tic process;pyrimidine ribonucleotide biosynthetic process;phosphorylation

RNA and LSU-rRNA from tricistronic rRNA transcript (SSU-rRNA, 5.8S rRNA, LSU-rRNA);endo

process;pyrimidine nucleotide biosynthetic process;cellular amino acid metabolic process;r

SSU-rRNA from 5.8S rRNA and LSU-rRNA from tricistronic rRNA transcript (SSU-rRNA, 5.8S

ocess;asparagine biosynthetic process;protein import into nucleus;pyrimidine ribonucleotid
ent

Do not distribute

iosynthetic process

stid organization;chloroplast organization;transcription from plastid promoter;protein targe

ino acid methylation;embryo sac egg cell differentiation;photomorphogenesis;embryonic d
ic process;starch biosynthetic process

pment ending in seed dormancy

e

process;UMP biosynthetic process;regulation of transcription, DNA-dependent;pyrimidine

to heat;response to bacterium;response to temperature stimulus;response to high light int

s;regulation of flower development;cullin deneddylation;histone lysine methylation
eus;protein maturation

ic process;response to zinc ion;cysteine biosynthetic process;response to cadmium ion

n

esponse to cadmium ion

e;RNA methylation;gluconeogenesis;cytoskeleton organization;pyrimidine ribonucleotide bi

g;RNA methylation;mRNA export from nucleus;protein import into nucleus;protein targetir

nport into nucleus;protein targeting to mitochondrion;ribosome biogenesis;protein maturat

nport into nucleus;embryo sac development

Do not distribute

etabolic process;embryo sac egg cell differentiation;photomorphogenesis;embryonic devel
ponse to temperature stimulus;negative regulation of flower development;maintenance of

hotomorphogenesis;regulation of flower development;cullin deneddylation;histone lysine

process
m determinacy

fructose stimulus;cellular response to sucrose stimulus;cellular response to light stimulus;c

Do not distribute

etic process;chloroplast organization;embryonic development ending in seed dormancy;thy

e to auxin stimulus;cell growth;lateral root development;cell division;response to ethylene

lticellular organismal development;plastid organization;gibberellin biosynthetic process;res

to nucleus;embryo sac development;pollen development;embryo sac egg cell differentiatio

nidine ribonucleotide biosynthetic process;photomorphogenesis;regulation of flower devel

some assembly

tein amino acid phosphorylation;signal transduction;response to xenobiotic stimulus;endo

rmancy

gulation of transcription, DNA-dependent;chromatin modification;histone arginine methyla

, using glutaminyl-peptide cyclotransferase;negative regulation of defense response

ise to salt stress;protein transport

Do not distribute

:ellular amino acid biosynthetic process;methionine biosynthetic process;threonine biosynt

:ess;cysteine biosynthetic process;pollen tube development

lgi to vacuole transport;cell communication;endosome to lysosome transport;photorespira

oroplast organization;polar nucleus fusion;cristae formation;regulation of programmed cel

ification;developmental process;ribosome biogenesis
:ess;cysteine biosynthetic process;aging;response to cadmium ion

tion;nucleosome assembly

:r deprivation;response to salt stress;response to abscisic acid stimulus;pollen maturation

iosynthetic process;regulation of flower development

orylation;phosphoinositide-mediated signaling;microgametogenesis

Do not distribute

process;phosphatidylglycerol biosynthetic process;ubiquinone biosynthetic process;isopre
n identity;ribosome biogenesis;negative regulation of transcription, DNA-dependent;floral

onse to cadmium ion;response to trehalose-6-phosphate stimulus
ocess to D-lactate;starch biosynthetic process;phosphoinositide phosphorylation

ositol hexakisphosphate biosynthetic process;methylglyoxal catabolic process to D-lactate;

mevalonate-independent pathway

nc ion;ribosome biogenesis;response to cadmium ion

esponse to arsenic

mulus

thylation on cytosine within a CG sequence;chromatin organization;cell division;vegetative
mRNA splicing, via spliceosome

Do not distribute

atid cohesion;synapsis;reciprocal meiotic recombination;regulation of chromosome organi:

rocess;pyrimidine ribonucleotide biosynthetic process;cellular response to phosphate starv

: stress;response to zinc ion;response to cadmium ion
ponse to stress;response to heat
sphorylation;multicellular organismal development;flower development;cell differentiatior

;
: stress;response to zinc ion;response to cadmium ion

erentiation;root hair elongation;response to red light;response to far red light;response to
on;response to cadmium ion;somatic cell DNA recombination

iosynthetic process;photoperiodism, flowering;starch metabolic process

:essing
ɜ to water deprivation;response to salt stress;flower development;heat acclimation;leaf de
ɜ to water deprivation;response to salt stress;flower development;heat acclimation;leaf de
embryonic development ending in seed dormancy;positive regulation of flower developme
ɜ to water deprivation;response to salt stress;flower development;heat acclimation;leaf de

ɔsome biogenesis

:ess;defense response to bacterium;callose deposition in cell wall during defense response;

' meristem;histone phosphorylation;cortical cytoskeleton organization

Do not distribute

ısport;cytoskeleton organization;response to wounding;response to fungus;jasmonic acid n

cuolar proton-transporting V-type ATPase complex assembly;vacuolar sequestering;seques

ⅠP biosynthetic process;response to cadmium ion

cylation;mitochondrion organization;chloroplast organization;tRNA aminoacylation;ovule c

ɔ cadmium ion

;rowth hormone stimulus;glutathione metabolic process;response to salt stress

ıetic process;stomatal complex morphogenesis;iron-sulfur cluster assembly;isopentenyl dip

ɔrylation;phosphoinositide-mediated signaling;microgametogenesis

‹in mediated signaling pathway

‹in mediated signaling pathway involved in phyllotactic patterning;phyllotactic patterning;n

ıily biosynthetic process;chloroplast relocation;thylakoid membrane organization;photosyn

sphorylation

Do not distribute

nium ion;stomatal movement;pollen development

ein export from nucleus;systemic acquired resistance;innate immune response

process

nent ending in seed dormancy;seed germination;regulation of flower development;meriste
g;cytoskeleton organization;pyrimidine ribonucleotide biosynthetic process;photomorphog

e-mediated transport

ion

;plant-type cell wall modification

ntensity;protein processing;protein metabolic process;response to hydrogen peroxide

sponse to bacterium
ollen germination;pollen tube growth;stomatal movement;phosphatidylinositol metabolic
cription, DNA-dependent

A metabolic process;peptidoglycan biosynthetic process;peptidoglycan-based cell wall biog
nium ion

embryonic development ending in seed dormancy;positive regulation of flower developme

Do not distribute

e interaction

lus;response to auxin stimulus;root development;response to wounding;seed germination;

embryonic development ending in seed dormancy;positive regulation of flower developme
ication;DNA repair;nucleotide metabolic process;2'-deoxyribonucleotide metabolic process
lation

tabolic process
nse to cadmium ion

[illegible]

osynthetic process;embryo sac development;pollen development
ssing;protein metabolic process
hexamerization;ATP catabolic process

sitol metabolic process;phosphoinositide phosphorylation;root hair elongation;root hair ce
ar tissue pattern formation;cotyledon vascular tissue pattern formation;root development;

protein maturation
ribonucleotide biosynthetic process;embryonic development ending in seed dormancy;rib
on;regulation of translational initiation;embryonic development;pollen germination

Do not distribute

sphorylation

rol-3-phosphate catabolic process;oxidation reduction;lipid metabolic process;glycerophos

ɔgenesis;regulation of flower development;cullin deneddylation;histone lysine methylation

ıbination;transcription, DNA-dependent;RNA elongation;RNA processing;sister chromatid c

RNA and LSU-rRNA from tricistronic rRNA transcript (SSU-rRNA, 5.8S rRNA, LSU-rRNA);endo

ırganization;protein complex assembly;hyperosmotic response;sister chromatid cohesion;r

Do not distribute

ynthetic process;isoleucine biosynthetic process;valine biosynthetic process;response to h

IA aminoacylation
ess

in RNA interference;gene silencing by RNA;posttranscriptional gene silencing by RNA

iisfolded protein;proteasome core complex assembly

ı;nucleotide biosynthetic process;tRNA aminoacylation

s;specification of floral organ identity;negative regulation of photomorphogenesis;signalos

ted transport;nucleocytoplasmic transport;signal transduction;small GTPase mediated signa

ng;arginine biosynthetic process;nitrogen compound metabolic process;cytoskeleton organ

thetic process;CTP biosynthetic process;phosphorylation

process;isopentenyl diphosphate biosynthetic process, mevalonate-independent pathway;

ponse to cold;response to flooding;response to cadmium ion
age stimulus;SOS response

inesis by cell plate formation;response to nutrient;response to water deprivation;response

Do not distribute

ndent pathway;cysteine biosynthetic process

tion;megagametogenesis
ation

process;response to cold

ization;photosynthesis;chlorophyll biosynthetic process;isopentenyl diphosphate biosynthe
d cell death

onse to high light intensity;heat acclimation;protein metabolic process;cellular response to
ansformation;response to cadmium ion

bolic process;cuticle development
etabolic process;acetyl-CoA metabolic process;steroid biosynthetic process;response to lig

ɔn;regulation of translational initiation

ı peroxide catabolic process

pment;embryonic development ending in seed dormancy;zygote asymmetric cell division;r[illegible]

c process;pyrimidine ribonucleotide biosynthetic process;embryo sac egg cell differentiatio
quinone biosynthetic process;nucleotide biosynthetic process;pyrimidine ribonucleotide bi[illegible]

ıulus
tein catabolic process;water transport;hyperosmotic response;Golgi organization;metaboli[illegible]

c process
atidylglycerol biosynthetic process;chlorophyll biosynthetic process;carotenoid biosyntheti

process;tRNA processing;methylation
tose-phosphate shunt;RNA elongation;rRNA processing;chloroplast organization;post-embr

ıtible interaction;negative regulation of transposition;production of siRNA involved in RNA

ınslation;translational initiation;regulation of translational initiation;photomorphogenesis;[illegible]
ss;cellular amino acid biosynthetic process;L-asparagine biosynthetic process
ıobilization;calcium ion transport;Golgi organization;metabolic process;response to salt stre

ır carbohydrate metabolic process;response to cadmium ion;gluconeogenesis;pentose-pho[illegible]

ism, flowering

lticellular organismal development;positive regulation of cell proliferation;embryonic patte

Do not distribute

ndent pathway
ne N-methylation
on;regulation of translational initiation;photomorphogenesis;response to salt stress;flower

Do not distribute

to cadmium ion
glutamyl-tRNA aminoacylation

ding in seed dormancy;photosynthesis;phosphorylation;response to cadmium ion

to late endosome transport
s

ıotorespiration;response to cadmium ion;response to misfolded protein;proteasome core (
:ll differentiation;long-day photoperiodism, flowering

ımbrane;thylakoid membrane organization

ı nucleus
thetic process;CTP biosynthetic process;response to UV;red, far-red light phototransductioı

ıslation;lysyl-tRNA aminoacylation;chloroplast organization;embryonic development endinĮ
:sponse to bacterium
ıto nucleus;pyrimidine ribonucleotide biosynthetic process;embryo sac egg cell differentiati
osynthetic process;response to heat;response to salt stress;oxidation reduction;L-proline b

thetic process;CTP biosynthetic process;response to oxidative stress;phosphorylation

ıdent pathway;cysteine biosynthetic process
branched chain family amino acid biosynthetic process;methionine biosynthetic process;th
gulation of programmed cell death;cell growth

:ribonucleoside triphosphate catabolic process
nino acid biosynthetic process;lysine biosynthetic process;diaminopimelate biosynthetic pr

oroplast stroma;de-etiolation;response to heat;response to salt stress;response to water d
nidine ribonucleotide biosynthetic process;response to blue light;chloroplast organization;c

nidine ribonucleotide biosynthetic process;pollen development

branched chain family amino acid biosynthetic process;methionine biosynthetic process;th

Do not distribute

ess

stroma;regulation of chlorophyll biosynthetic process;protein targeting to chloroplast;chlor

vall organization;plant-type cell wall biogenesis;auxin polar transport;root morphogenesis;r
thetic process;CTP biosynthetic process;response to salt stress;phosphorylation;response t

protein transport
ll wall organization;cell wall modification;cellular protein metabolic process;response to ca

ocess;oxidation reduction

ll growth

hthamide biosynthetic process from peptidyl-histidine;methylation

A, 4.5S-rRNA, 5S-rRNA);plastid organization
on
response to cadmium ion

stress;response to oxidative stress;spermidine biosynthetic process;response to cold;putr

netic process
sponse to auxin stimulus;response to abscisic acid stimulus;response to gibberellin stimulus

A-dependent

nse to cadmium ion;oxidation reduction

Do not distribute

;ative regulation of abscisic acid mediated signaling pathway;positive regulation of transcri[illegible]

n catabolic process;response to auxin stimulus

ndent pathway

ll growth
se to cadmium ion

se to heat;heat acclimation

ation reduction;pyridoxal 5'-phosphate salvage

ion;response to trehalose-6-phosphate stimulus
g in seed dormancy

alt stress;response to jasmonic acid stimulus;response to cadmium ion;response to abscisic

action
action
stress;response to cadmium ion

Do not distribute

seed coat development;nitrate transport;oxalate catabolic process;para-aminobenzoic acic

tide biosynthetic process;embryonic development ending in seed dormancy;regulation of f

lar response to gravity;microtubule cytoskeleton organization;response to salt stress

ion;cellular response to ethylene stimulus;response to zinc ion;pollen tube growth;pollen c

rocess;cellulose catabolic process

chloroplast relocation;thylakoid membrane organization;ncRNA metabolic process

process;response to abscisic acid stimulus;embryonic development ending in seed dormanc
d stimulus

e to auxin stimulus;cell growth;lateral root development;cell division

gulation of transcription, DNA-dependent

se to phosphate starvation

rocess;response to cadmium ion

Do not distribute

›synthetic process;response to UV-B

ulation of transcription from RNA polymerase II promoter;translational elongation;negative

ıetic process;N-terminal protein amino acid modification
de;response to cadmium ion

e;metabolic process;cellular amino acid biosynthetic process;cysteine biosynthetic process;

›nse to nitrogen starvation;positive regulation of cell proliferation;asymmetric cell division;

ng in seed dormancy;sterol biosynthetic process;brassinosteroid biosynthetic process

on;embryonic development ending in seed dormancy;protein transport

Do not distribute

A, 5.8S rRNA, LSU-rRNA);translational elongation

nembrane organization;vegetative to reproductive phase transition of meristem;iron-sulfur

e to cadmium ion;oxidation reduction

oxide;regulation of transcription, DNA-dependent;fatty acid beta-oxidation;response to osm

evelopment

Do not distribute

Do not distribute

biosynthetic process;photomorphogenesis;cullin deneddylation

methylation

methylation

Do not distribute

xide metabolic process;photosynthesis

Do not distribute

ation of immune response;defense response to fungus

Do not distribute

Do not distribute

ar nucleus fusion;synergid death;chaperone mediated protein folding requiring cofactor

nucleolytic cleavage to generate mature 3'-end of SSU-rRNA from (SSU-rRNA, 5.8S rRNA, LS

metabolic process;pyrimidine ribonucleotide biosynthetic process;cellular response to pho[illegible]

rRNA, LSU-rRNA);endonucleolytic cleavage to generate mature 3'-end of SSU-rRNA from ([illegible]

[illegible]le biosynthetic process

Do not distribute

[illegible]eting to chloroplast;cell redox homeostasis;positive regulation of transcription, DNA-depen[illegible]

[illegible]levelopment ending in seed dormancy;seed germination;regulation of flower development

ribonucleotide biosynthetic process;transcription factor import into nucleus;post-translati[illegible]

[illegible]ensity;response to cadmium ion;protein ubiquitination

Do not distribute

iosynthetic process;photomorphogenesis;regulation of flower development;cullin deneddy

ng to mitochondrion;photomorphogenesis;regulation of flower development;cullin denedc

tion

lopment;cullin deneddylation;nucleologenesis;cotyledon development;cell division;regulati
f floral meristem identity;maintenance of inflorescence meristem identity;floral meristem c

methylation

Do not distribute

cellular response to glucose stimulus

ylakoid membrane organization;iron-sulfur cluster assembly;protein targeting to chloroplas

stimulus

sponse to auxin stimulus;response to abscisic acid stimulus;abscisic acid mediated signaling

on;double fertilization forming a zygote and endosperm;histone phosphorylation;histone H[illegible]

lopment;cullin deneddylation;histone lysine methylation

plasmic reticulum unfolded protein response;positive regulation of GTPase activity

ation;histone H3-R26 methylation;histone H3-R17 methylation;histone H3-R2 methylation;[illegible]

Do not distribute

:hetic process;lysine biosynthetic process via diaminopimelate;isoleucine biosynthetic proc

ation;positive gravitropism;auxin homeostasis;endosome transport;root development;resp

ll death;ovule development

Do not distribute

:enoid biosynthetic process;aromatic amino acid family biosynthetic process;response to blu
organ morphogenesis

Do not distribute

glucosinolate biosynthetic process;L-ascorbic acid biosynthetic process;phosphoinositide p

to reproductive phase transition of meristem

ization;meiotic DNA double-strand break formation;meiotic chromosome segregation

vation;carbamoyl phosphate biosynthetic process;pyrimidine nucleotide biosynthetic proce

n

high light intensity

velopment;protein stabilization;cellular response to calcium ion
velopment;protein stabilization;cellular response to calcium ion
ent;endosperm development
velopment;protein stabilization;cellular response to calcium ion

;gravitropism

Do not distribute

netabolic process;jasmonic acid biosynthetic process;response to jasmonic acid stimulus;pr

stering of zinc ion;ATP synthesis coupled proton transport

development

Do not distribute

phosphate biosynthetic process, mevalonate-independent pathway;protein targeting to chl

naintenance of shoot apical meristem identity;leaf phyllotactic patterning

nthesis;iron-sulfur cluster assembly;isopentenyl diphosphate biosynthetic process, mevalon

m structural organization;seed dormancy;sugar mediated signaling pathway;vegetative to [illegible]
genesis;regulation of flower development;cullin deneddylation;proteasomal protein catabo

Do not distribute

process;phosphoinositide phosphorylation;phosphorylation

genesis;chloroplast organization;chloroplast relocation;chloroplast fission;thylakoid membr

ent;endosperm development

:cell division

ent;endosperm development
s

Do not distribute

ell tip growth;positive gravitropism;lateral root formation
;leaf development;shoot development;petal vascular tissue pattern formation;sepal vascula

osome biogenesis

;pholipid metabolic process;phospholipid biosynthetic process;systemic acquired resistance

ı

cohesion;meiosis;synapsis;reciprocal meiotic recombination;embryo sac egg cell differentia

›nucleolytic cleavage to generate mature 3'-end of SSU-rRNA from (SSU-rRNA, 5.8S rRNA, L[illegible]

eciprocal meiotic recombination;photomorphogenesis;response to absence of light;respon

erbicide

Do not distribute

ome assembly;cullin deneddylation;positive regulation of G2/M transition of mitotic cell cy

al transduction;protein transport

ization;metabolic process;cellular amino acid biosynthetic process;proteasomal protein ca

protein targeting to chloroplast

to ethylene stimulus;abscisic acid mediated signaling pathway;potassium ion import;stom

Do not distribute

etic process, mevalonate-independent pathway;ncRNA metabolic process

heat;response to endoplasmic reticulum stress;response to hydrogen peroxide;protein un

ht stimulus;anatomical structure morphogenesis;plant-type cell wall organization;lignin me

egulation of chromosome organization;meiotic DNA double-strand break formation;meioti

n;transcription factor import into nucleus
osynthetic process

c process;aerobic respiration;response to temperature stimulus;response to salt stress;phc

ic process;xanthophyll biosynthetic process;starch biosynthetic process;isopentenyl diphos

ryonic development;embryonic development ending in seed dormancy;seed germination;c

interference;gene silencing by RNA;ovule development;histone H3-K9 methylation

cullin deneddylation

ess;chloroplast organization;indoleacetic acid biosynthetic process;reductive pentose-phos

sphate shunt

ern specification;longitudinal axis specification;apical cell fate commitment;cotyledon deve

Do not distribute

development

Do not distribute

complex assembly

n;auxin mediated signaling pathway;phosphorylation;isopentenyl diphosphate biosyntheti

g in seed dormancy;thylakoid membrane organization;vegetative to reproductive phase tra

ion;methylation;transcription factor import into nucleus
iosynthetic process

reonine biosynthetic process;isoleucine biosynthetic process;oxidation reduction

ocess

eprivation
chloroplast relocation;chloroplast fission;plastid fission

reonine biosynthetic process;isoleucine biosynthetic process;oxidation reduction

Do not distribute

·oplast organization

regulation of meristem growth;growth;ribosome biogenesis;anther development
:o cadmium ion;nucleotide metabolic process

ıdmium ion

Do not distribute

·escine biosynthetic process;response to wounding;response to salt stress;response to absc

s;response to salicylic acid stimulus;response to jasmonic acid stimulus;response to cadmiu

ption, DNA-dependent;response to water deprivation;negative regulation of cell growth;ph

Do not distribute

c acid stimulus

d metabolic process;response to cadmium ion;defense response to fungus

flower development;histone modification;floral organ formation

development

Do not distribute

cy;nucleotide metabolic process;IMP salvage

e regulation of flower development;seed dormancy;regulation of RNA elongation;regulatio

;photoperiodism, flowering

response to cold;pollen development;embryonic development ending in seed dormancy;ve

Do not distribute

r cluster assembly;positive regulation of transcription, DNA-dependent;ovule development

notic stress;signal transduction;circadian rhythm;cell death;response to cold;response to w

Do not distribute

Do not distribute

Do not distribute

Do not distribute

Do not distribute

Do not distribute

SU-rRNA);translational elongation;ribosomal small subunit biogenesis

sphate starvation;'de novo' UMP biosynthetic process

SSU-rRNA, 5.8S rRNA, LSU-rRNA);rRNA export from nucleus;translation;response to osmotic

ident;oxidation reduction

:;meristem structural organization;maintenance of meristem identity;seed dormancy;sugar

onal protein modification;'de novo' UMP biosynthetic process;positive regulation of transcr

Do not distribute

Do not distribute

ylation;proteasomal protein catabolic process;histone lysine methylation

dylation;ribosome biogenesis

ion of cell division
determinacy;floral whorl development;regulation of timing of transition from vegetative to

Do not distribute

st;positive regulation of transcription, DNA-dependent

g pathway;gibberellic acid mediated signaling pathway;embryonic development;terpenoid

3-K9 methylation

Do not distribute

peptidyl-arginine methylation, to asymmetrical-dimethyl arginine;vegetative to reproductiv

ess;oxidation reduction

onse to misfolded protein;proteasome core complex assembly

Do not distribute

ue light;response to high light intensity;response to sucrose stimulus;leaf morphogenesis;th

Do not distribute

hosphorylation

ess;arginine biosynthetic process;glutamine metabolic process;cellular amino acid biosynth

Do not distribute

roteasomal protein catabolic process

Do not distribute

loroplast

ıate-independent pathway;ncRNA metabolic process;transcription from plastid promoter;p

reproductive phase transition of meristem;regulation of cell cycle process;protein ubiquitir
›lic process;histone lysine methylation;cellular protein metabolic process

Do not distribute

·ane organization;ncRNA metabolic process;transcription from plastid promoter;positive re

Do not distribute

ar tissue pattern formation

e;glycerolipid biosynthetic process;glycerolipid metabolic process;anther development

ation;response to gamma radiation;regulation of cell cycle process;protein ubiquitination;re

SU-rRNA);translational elongation;ribosomal small subunit biogenesis

ıse to salt stress;embryonic development ending in seed dormancy;seed germination;embr

Do not distribute

ycle;regulation of defense response;positive regulation of protein catabolic process

tabolic process;'de novo' UMP biosynthetic process;histone H3-K9 methylation;cellular res

natal movement;cellular response to phosphate starvation;galactolipid biosynthetic process

Do not distribute

nfolding;positive regulation of translation

etabolic process;multidimensional cell growth;unidimensional cell growth;plant-type cell w

ic chromosome segregation

otorespiration;response to zinc ion;cysteine biosynthetic process;proteasomal ubiquitin-de

sphate biosynthetic process, mevalonate-independent pathway;positive regulation of catal

chloroplast relocation;thylakoid membrane organization;photosystem II assembly;vegetativ

sphate cycle;isopentenyl diphosphate biosynthetic process, mevalonate-independent pathv

elopment

Do not distribute

Do not distribute

c process, mevalonate-independent pathway;response to hydrogen peroxide

ansition of meristem;iron-sulfur cluster assembly;ovule development

Do not distribute

Do not distribute

cisic acid stimulus;response to jasmonic acid stimulus;seed development

um ion

hotoperiodism, flowering;regulation of timing of transition from vegetative to reproductive

Do not distribute

Do not distribute

on of RNA elongation from RNA polymerase II promoter;response to gibberellin stimulus

ernalization response;stomatal lineage progression;regulation of meiosis;DNA endoredupli

Do not distribute

t

/ater deprivation;response to salt stress;cytokinin mediated signaling pathway;hormone-m

Do not distribute

Do not distribute

Do not distribute

Do not distribute

Do not distribute

Do not distribute

c stress;response to salt stress;mature ribosome assembly

Do not distribute

mediated signaling pathway;positive regulation of vernalization response;vegetative to re

ription, DNA-dependent;oxidation reduction

Do not distribute

› reproductive phase;multicellular organismal development;flower development;cell differe

Do not distribute

biosynthetic process;mitochondrial signaling pathway;positive regulation of transcription, [illegible]

Do not distribute

ve phase transition of meristem;regulation of flower development

Do not distribute

ıylakoid membrane organization;response to red light;regulation of proton transport;respo

Do not distribute

ıetic process;'de novo' UMP biosynthetic process

Do not distribute

Do not distribute

protein targeting to chloroplast

nation;lipid storage;leaf development;cotyledon development;response to freezing;cell divi

Do not distribute

egulation of transcription, DNA-dependent

Do not distribute

egulation of telomere maintenance;meiotic DNA double-strand break formation;telomere

ryonic pattern specification;regulation of flower development;meristem structural organiza

Do not distribute

sponse to phosphate starvation

s;calcium-mediated signaling;cellular response to water deprivation

Do not distribute

vall biogenesis;secondary cell wall biogenesis;cell tip growth;regulation of meristem growth

Do not distribute

:pendent protein catabolic process;cellular carbohydrate metabolic process;response to ca[illegible]

ytic activity;oxidation reduction

/e to reproductive phase transition of meristem;iron-sulfur cluster assembly;starch biosynt[illegible]

way;cysteine biosynthetic process;glycerol catabolic process;multicellular organism reprod[illegible]

Do not distribute

Do not distribute

Do not distribute

e phase;response to blue light

Do not distribute

Do not distribute

Do not distribute

cation;negative regulation of cyclin-dependent protein kinase activity;gametophyte develo

ıediated signaling pathway;salicylic acid mediated signaling pathway;ethylene mediated sig

Do not distribute

Do not distribute

Do not distribute

Do not distribute

Do not distribute

Do not distribute

Do not distribute

eproductive phase transition of meristem;protein ubiquitination;histone modification;lipid s

Do not distribute

entiation

Do not distribute

DNA-dependent;cotyledon development;cell division;transcription from RNA polymerase II

Do not distribute

Do not distribute

›nse to far red light;chlorophyll biosynthetic process;terpenoid biosynthetic process;carote

Do not distribute

Do not distribute

Do not distribute

ision

Do not distribute

Do not distribute

maintenance in response to DNA damage;meiotic chromosome segregation

ation;primary shoot apical meristem specification;trichome morphogenesis;seed dormancy

Do not distribute

Do not distribute

ı;regulation of hormone levels;sterol biosynthetic process;brassinosteroid biosynthetic proı

dmium ion;response to misfolded protein;proteasome core complex assembly

hetic process;ncRNA metabolic process;regulation of protein amino acid dephosphorylatio[illegible]

uction;regulation of protein localization;glyceraldehyde-3-phosphate biosynthetic process;

Do not distribute

Do not distribute

Do not distribute

Do not distribute

Do not distribute

Do not distribute

ɔpment;phosphorylation

Do not distribute

;naling pathway;regulation of flower development;regulation of seed germination;phloem [illegible]

Do not distribute

Do not distribute

Do not distribute

Do not distribute

Do not distribute

Do not distribute

Do not distribute

storage;floral organ formation;response to freezing;methylation;histone arginine methylati

Do not distribute

Do not distribute

I promoter;regulation of transcription from RNA polymerase II promoter

Do not distribute

Do not distribute

enoid biosynthetic process;isopentenyl diphosphate biosynthetic process, mevalonate-inde

Do not distribute

Do not distribute

Do not distribute

Do not distribute

Do not distribute

/;sugar mediated signaling pathway;vegetative to reproductive phase transition of merister

Do not distribute

Do not distribute

cess;cysteine biosynthetic process;anthocyanin accumulation in tissues in response to UV l

Do not distribute

n;transcription from plastid promoter;positive regulation of catalytic activity;positive regul

;primary root development

Do not distribute

Do not distribute

Do not distribute

Do not distribute

Do not distribute

Do not distribute

or xylem histogenesis;leaf senescence;regulation of chlorophyll catabolic process;cellular r

Do not distribute

Do not distribute

Do not distribute

Do not distribute

Do not distribute

Do not distribute

Do not distribute

ion

Do not distribute

Do not distribute

Do not distribute

Do not distribute

pendent pathway;cysteine biosynthetic process;cell differentiation;positive regulation of tr

Do not distribute

Do not distribute

Do not distribute

Do not distribute

Do not distribute

m;cullin deneddylation;seed maturation;regulation of cell cycle process;positive regulation

Do not distribute

Do not distribute

light;root development;anther development;root hair elongation;cellular developmental pr

Do not distribute

ation of transcription, DNA-dependent;ovule development

Do not distribute

Do not distribute

Do not distribute

Do not distribute

Do not distribute

Do not distribute

'esponse to phosphate starvation;phosphorylation;protein import into peroxisome matrix;p

Do not distribute

Do not distribute

Do not distribute

Do not distribute

Do not distribute

Do not distribute

Do not distribute

Do not distribute

Do not distribute

Do not distribute

Do not distribute

ranscription, DNA-dependent

Do not distribute

Do not distribute

Do not distribute

Do not distribute

Do not distribute

of organelle organization;protein ubiquitination;histone methylation;protein deubiquitinat

Do not distribute

Do not distribute

rocess;oxidation reduction;cell wall organization

Do not distribute

Do not distribute

Do not distribute

Do not distribute

Do not distribute

Do not distribute

Do not distribute

peptidyl-histidine phosphorylation;signal transmission via phosphorylation event;regulatior

Do not distribute

Do not distribute

Do not distribute

Do not distribute

Do not distribute

Do not distribute

Do not distribute

Do not distribute

Do not distribute

Do not distribute

Do not distribute

Do not distribute

Do not distribute

Do not distribute

Do not distribute

Do not distribute

tion;lipid storage;regulation of chromosome organization;regulation of cell differentiation;

Do not distribute

Do not distribute

Do not distribute

Do not distribute

Do not distribute

Do not distribute

Do not distribute

Do not distribute

Do not distribute

n of anthocyanin metabolic process;negative regulation of iron ion transport;defense respc

Do not distribute

Do not distribute

Do not distribute

Do not distribute

Do not distribute

Do not distribute

Do not distribute

Do not distribute

Do not distribute

Do not distribute

Do not distribute

Do not distribute

Do not distribute

Do not distribute

Do not distribute

Do not distribute

:positive regulation of transcription, DNA-dependent;leaf development;cotyledon developm

Do not distribute

Do not distribute

Do not distribute

Do not distribute

Do not distribute

Do not distribute

Do not distribute

Do not distribute

Do not distribute

ɔnse to bacterium;regulation of meristem development;regulation of shoot development;c

Do not distribute

Do not distribute

Do not distribute

Do not distribute

Do not distribute

Do not distribute

Do not distribute

Do not distribute

Do not distribute

Do not distribute

Do not distribute

Do not distribute

Do not distribute

Do not distribute

Do not distribute

Do not distribute

ment;response to freezing;cell division

Do not distribute

Do not distribute

Do not distribute

Do not distribute

Do not distribute

Do not distribute

Do not distribute

Do not distribute

Do not distribute

:ellular response to cold;cellular response to abscisic acid stimulus;cellular response to sucr

Do not distribute

Do not distribute

Do not distribute

Do not distribute

Do not distribute

Do not distribute

Do not distribute

Do not distribute

Do not distribute

Do not distribute

Do not distribute

Do not distribute

Do not distribute

Do not distribute

Do not distribute

Do not distribute

Do not distribute

Do not distribute

Do not distribute

Do not distribute

Do not distribute

Do not distribute

Do not distribute

Do not distribute

Do not distribute

rose stimulus;secondary growth

Do not distribute
