# Supplementary material for: Leaf morphology, rather than plant water status, underlies genetic variation of rice leaf rolling under drought
Source: Plant Cell Environ. 2019 Feb 20;42(5):1532–44. doi: 10.1111/pce.13514 (PMC6487826; doi:10.1111/pce.13514)
Supplement: Supplementary file 1 — Table S1. Genotypes included in the aus experiments in the field and greenhouse. Table S2. Genotypes included in the tropical japonica greenhouse experiment. Table S3. Leaf anatomical parameters measured in eight selected aus genotypes in field drought stress and well‐watered treatments. Table S4. Correlations between the change in normalized difference vegetation index (ΔNDVI) and leaf rolling score (LRS) in the 2010 and 2012 field experiments, based on Spearman's rank correlation. Table S5. Relationships among canopy temperature (CT) and the change in normalized difference vegetation index (ΔNDVI) or and shoot biomass, based on ANOVA on a panel of 226 aus rice genotypes under drought conditions in 3 field studies during the dry season of 2010, 2011, 2012. Table S6. Relationships among leaf rolling (leaf rolling score ΔNDVI) with maintenance of biomass and grain yield under drought in the aus field and greenhouse experiments, based on correlation (Spearman's for leaf rolling traits, Pearson for ΔNDVI). Table S7. Sclerenchyma cell area and number in six selected aus genotypes in the 2018DS field well‐watered treatment. Letter groups indicate significant differences among genotypes (p < 0.05). Table S8. Bulliform cell size and number, as well as stomatal density, in eight selected aus genotypes in field drought stress and well‐watered treatments. Table S9. The most contrasting genotypes from the japonica panel in terms of leaf rolling score and maintenance of shoot biomass under drought as compared to that under well‐watered conditions (SDWratio, calculated as (DS‐WW/WW)). Table S10. Traits for which association analysis was conducted on genotypes with available sequence data. Table S11. List of top markers (−log10(P‐value) > 4.0) from association mapping using EMMAX model for leaf rolling scores and ΔNDVI from different experiments. Table S12. List of markers with annotations from gene models following the Rice Genome Annotation Project (Kawahara et al., 2013) and O [file PCE-42-1532-s001.zip › pce13514_Supp_Figures.docx]

Supp. Fig. S1. Rainfall and soil water potential in the drought treatment (30 cm depth) from the drought treatments in the aus field studies.

Supp. Fig. S2. Distributions of mean leaf rolling scores of aus genotypes in the field and greenhouse lysimeter experiments.

Supp. Fig. S3. Leaf morphology distributions among 26 selected genotypes in the aus panel grown in the greenhouse lysimeter study. The youngest fully expanded leaf at 71 DAS was measured to determine A) leaf area, B) leaf length, C) leaf width, and D) specific leaf area.

Supp. Fig. S4. Distribution of mean leaf rolling score of 172 tropical japonica genotypes at the end of the greenhouse study, when the soil moisture level reached FTSW of 0.2.

Supp. Fig. S5. Leaf morphology distributions among tropical japonical panel genotypes grown in the greenhouse.The last ligulated leaf was measured when the soil dried to FTSW=0.2 to determine A) leaf area, B) leaf length, C) leaf width, and D) specific leaf area.

Supp. Fig. S6. Relationship between mean leaf rolling score of 156 japonica genotypes at the end of the greenhouse study and the time required for the soil moisture level of each genotype to reach FTSW of 0.2.

Supp. Fig. S7. Manhattan plots and Quantile-Quantile plots of genome-wide association analysis in the aus panel for A-B) leaf rolling score in the 2010 field drought trial; C-D) leaf rolling score in the 2012 field drought trial on medium-duration genotypes (74 DAS); E-F) change in NDVI in the 2010 field drought trial; G-H) change in NDVI in the 2012 field drought trial on medium-duration genotypes.

Supp Fig S8. Manhattan plot and Quantile-Quantile plot of genome-wide association analysis of leaf rolling score in the greenhouse study of 172 tropical japonica genotypes.

Supp. Fig. S9. Quantile-Quantile plot of genome-wide association analysis in the aus panel leaf rolling score in the 2012 field drought trial on medium-duration genotypes (106 DAS), adjusted for effects of marker density and correlation.
