# Supplementary material for: Leaf morphology, rather than plant water status, underlies genetic variation of rice leaf rolling under drought
Source: Plant Cell Environ. 2019 Feb 20;42(5):1532–44. doi: 10.1111/pce.13514 (PMC6487826; doi:10.1111/pce.13514)
Supplement: Supplementary file 1 — Table S1. Genotypes included in the aus experiments in the field and greenhouse. Table S2. Genotypes included in the tropical japonica greenhouse experiment. Table S3. Leaf anatomical parameters measured in eight selected aus genotypes in field drought stress and well‐watered treatments. Table S4. Correlations between the change in normalized difference vegetation index (ΔNDVI) and leaf rolling score (LRS) in the 2010 and 2012 field experiments, based on Spearman's rank correlation. Table S5. Relationships among canopy temperature (CT) and the change in normalized difference vegetation index (ΔNDVI) or and shoot biomass, based on ANOVA on a panel of 226 aus rice genotypes under drought conditions in 3 field studies during the dry season of 2010, 2011, 2012. Table S6. Relationships among leaf rolling (leaf rolling score ΔNDVI) with maintenance of biomass and grain yield under drought in the aus field and greenhouse experiments, based on correlation (Spearman's for leaf rolling traits, Pearson for ΔNDVI). Table S7. Sclerenchyma cell area and number in six selected aus genotypes in the 2018DS field well‐watered treatment. Letter groups indicate significant differences among genotypes (p < 0.05). Table S8. Bulliform cell size and number, as well as stomatal density, in eight selected aus genotypes in field drought stress and well‐watered treatments. Table S9. The most contrasting genotypes from the japonica panel in terms of leaf rolling score and maintenance of shoot biomass under drought as compared to that under well‐watered conditions (SDWratio, calculated as (DS‐WW/WW)). Table S10. Traits for which association analysis was conducted on genotypes with available sequence data. Table S11. List of top markers (−log10(P‐value) > 4.0) from association mapping using EMMAX model for leaf rolling scores and ΔNDVI from different experiments. Table S12. List of markers with annotations from gene models following the Rice Genome Annotation Project (Kawahara et al., 2013) and O [file PCE-42-1532-s001.zip › Supp Table S3-S13, S17-S18..docx]

Supp. Table S3. Leaf anatomical parameters measured in eight selected aus genotypes in field drought stress and well-watered treatments.

Supp. Table S4. Correlations between the change in normalized difference vegetation index (ΔNDVI) and leaf rolling score (LRS) in the 2010 and 2012 field experiments, based on Spearman’s rank correlation.

Supp. Table S5. Relationships among canopy temperature (CT) and the change in normalized difference vegetation index (ΔNDVI) or and shoot biomass, based on ANOVA on a panel of 226 aus rice genotypes under drought conditions in 3 field studies during the dry season of 2010, 2011, 2012.

Supplementary Table S6. Relationships among leaf rolling (leaf rolling score ΔNDVI) with maintenance of biomass and grain yield under drought in the aus field and greenhouse experiments, based on correlation (Spearman’s for leaf rolling traits, Pearson for ΔNDVI).

Supp. Table S7. Sclerenchyma cell area and number in six selected aus genotypes in the 2018DS field well-watered treatment. Letter groups indicate significant differences among genotypes (p<0.05).

Supp. Table S8. Bulliform cell size and number, as well as stomatal density, in eight selected aus genotypes in field drought stress and well-watered treatments.

Supp. Table S9. The most contrasting genotypes from the japonica panel in terms of leaf rolling score and maintenance of shoot biomass under drought as compared to that under well-watered conditions (SDWratio, calculated as (DS-WW/WW)).

Supp. Table S10. Traits for which association analysis was conducted on genotypes with available sequence data.

Supp. Table S11. List of top markers (−log10(P-value) > 4.0) from association mapping using EMMAX model for leaf rolling scores and ΔNDVI from different experiments.

Supp. Table S12. List of markers with annotations from gene models following the Rice Genome Annotation Project (Kawahara *et al.*, 2013) and OGRO/ Q-TARO database (Yamamoto *et al.*, 2012).

Supp. Table S13. Aus candidate loci for leaf rolling under drought and their annotations.

Supp. Table S17. Loci from the aus panel predicted by RiceNet2 to be involved in networks related leaf rolling under drought.

Supp. Table S18. Loci from the tropical japonica panel predicted to be involved in networks related leaf rolling under drought.
